# Spatial disparities in zero-dose vaccination coverage for children aged 12–23 months in Ethiopia: A geographically weighted regression analysis

**DOI:** 10.1371/journal.pone.0332162

**Published:** 2025-09-11

**Authors:** Berhanu Fikadie Endehabtu, Kassahun Alemu, Shegaw Angaw Mengiste, Mesert Zelalem, Monika Knudsen Gullslett, Biniyam Tilahun

**Affiliations:** 1 Department of Health Informatics, Institute of Public Health, College of Medicine and Health Sciences, University of Gondar, Gondar, Ethiopia; 2 Center for Digital Health and Implementation Science, University of Gondar, Gondar, Ethiopia; 3 Department of Epidemiology and Biostatistics, Institute of Public Health, College of Medicine and Health Sciences, University of Gondar, Gondar, Ethiopia; 4 School of Business, University of South-Eastern Norway, Drammen, Norway; 5 Adjunct Assistant Professor of Pediatrics and Child Health, University of Gondar, Gondar, Ethiopia; 6 Chief of Programs and Research African Center for Early Childhood Development, Addis Ababa, Ethiopia; 7 Norwegian Centre for E-health Research, University Hospital of North Norway, Tromsø, Norway; Harvard University T H Chan School of Public Health, ETHIOPIA

## Abstract

**Background:**

Though Ethiopia has made significant progress in childhood vaccination, many children remain unvaccinated, making it the third largest contributor to the global burden of zero-dose children. Zero-dose children are those who doesn’t receive the first dose of diphtheria, tetanus and pertussis containing vaccine. Identifying geographic inequities of zero-dose prevalence and the factors influencing it could help to effectively reach and identify at-risk children and to design tailored intervention.

**Objectives:**

This study aimed to assess the geographical inequities and predicting factors of zero-dose children aged 12–23 month in Ethiopia.

**Methods:**

We used a population-based survey data. A total of 6,212 children aged 12–23 were included. The spatial autocorrelation was employed to examine geographic variations in zero-dose children. Getis-Ord Gi* statistics was used for hotspot analyses. A Kriging interpolation technique used to estimate values of zero-dose at unmeasured locations based on known values of zero-dose at observed locations. The Geographic Weighted Regression (GWR) analysis was used to elicit determinants of geographic difference in zero-dose children. Adjusted R2 and Akaike Information Criteria (AICc) were used to compare the models.

**Results:**

The prevalence of zero-dose children was 24.8% [CI: 23.7%−25.8%] ranged from 0.9% in Addis Ababa to 40.7% in Somali region. The zero-dose prevalence varied across the study area (Moran’s I = 0.193; P-value<0.0001). Significantly higher proportions of zero doses (hotspot areas) were identified in the north and south Somali, northwest Afar, East Amhara, and southern Oromia regions. GWR analysis showed that no ANC utilization, no TT/Td vaccination, poor perceptions on immunization, and far distance to healthcare facilities contributed to these geographic variations.

**Conclusion:**

This study revealed that the prevalence of Zero-dose is unacceptably high, with geographic inequities varying across the country. Factors such as ANC utilization, TT/Td vaccination, perceptions of immunization, and distance to healthcare facilities contributed to these geographic differences. This underscores the importance of designing and implementing tailored interventions to identify and reach zero-dose children. Such an approach could help achieve the national and global immunization goal of leaving no one behind by providing equitable access to immunization.

## Introduction

Immunization is one of the most successful and cost-effective interventions saving millions of live every year and ensuring global wellbeing [1]. Between 2021 and 2030, routine immunization is projected to avert an estimated 5.1 million child death globally [2]. Despite a remarkable increase in childhood immunization coverage globally, an estimated 14.5 million children did not receive the first dose of DPT containing vaccines (zero-dose children) in 2023 [3]. Approximately 60% of these zero-dose children were found to be living in ten low-and middle-income countries (LMICs), including Ethiopia [3,4].

Zero-dose children, defined as those who have not received a first dose of Diphtheria Tetanus and Pertussis (DTP-1) vaccine [4,5] are a key focus for global health policy makers. Although, Ethiopia has made commendable progress in immunization coverage [6], it remains one of the ten countries with the highest number of for unvaccinated and under-vaccinated children globally. In Ethiopia, approximately 917,000 children haven’t received the first dose of DPT containing vaccines, accounting for 6% of the global burden of zero-dose children [7]. These zero-dose children are at high risk of mortality from vaccine preventable disease (VPDS) and a potential source of vaccine preventable disease outbreaks [8–11]. These unvaccinated children are also often disadvantaged of other basic services and suffer from rooted inequality [12–14]. These children mainly reside in marginalized or disadvantaged communities marked by poverty, limited access to essential health services, urban slums, inadequate sanitation, and conflict affected areas [1,13,15,16]. These factors, along with socioeconomic, demographic, and health service related factors, contribute significantly to the uneven distribution of zero-dose children within countries [13].

Addressing the issue of zero-dose children is vital for meeting both the national and global agenda in reducing the number of zero-dose children by 50% by the year 2030. This effort aligns with the commitment to “leave no one behind” [1] and Gavi, the Vaccine Alliance’s strategy for 2021–2025 [17]. Realizing equitable access to child health services is the priority for international development [18]. Reducing socioeconomic and geographic inequities in access to health care is a key for countries to achieve Universal Health Coverage [19]. Closing the immunization equity gap must start with zero-dose children and missed communities [12].

The geographic inequities of immunization coverage can obstruct the fight against VPDs [20]. Existing evidence showed the presence of geographic disparities in immunization Coverage [21–30]. Spatial analysis, as a statistical tool, helps to pinpoint clusters of events, and identify areas that are more susceptible to health risks [30–32]. Spatial analyses of data from various surveys conducted in the Ethiopia [33–37] have shown a persistent regional divide in immunization coverage. Recent interest in studying spatial inequities in childhood immunization seeks to uncover gaps in immunization coverage, facilitating targeted efforts to effectively close these gaps [38–40]. In addition, aggregate-level analysis masks inequities in coverage, granular analysis can help identify persistently missed hot spots (areas with high unvaccinated zero-dose) [24,41,42].

However, there is limited evidence revealing the geographic inequities of zero-dose children at the national level and the factors contributing to these inequities. Identifying the geographic disparities in zero-dose coverage and the factors influencing them could help effectively reach and identify at-risk children, as well as design tailored interventions. Therefore, this study aimed to examine the geographic inequities and local predictors of zero-dose children aged 12–23 months in Ethiopia.

## Methods and materials

### Study design and setting

The data for this study were gathered in Ethiopia using a cross-sectional household survey method. Ethiopia is located at (3^o^-14^o^N and 33^o^ – 48°E) the horn of Africa. Administratively, the country is divided into eleven regions and two city administrations. Further, each region is divided into zones, woredas and kebeles (the smallest administrative unit). This study included all regions and two city administrations of the country, with the exception of the Tigray region due to internal conflicts.

### Data sources and data type

This study involved a secondary analysis of the national immunization program evaluation conducted in 2023 [43]. We analyzed nationally representative data from a recent immunization program assessment survey in Ethiopia, which covers 10 regions and two city administrations. The survey included a nationally representative sample of 15,158 households with children aged 12–35 months. The sample sizes were calculated using a single population proportion formula. The assumptions were 95% confidence level, 2% margin of errors, a design effect of 3, and 10% expected nonresponse rate. A two-stage stratified sampling technique was used to select the survey population. In the first stage, enumeration areas (EAs) were randomly selected from stratified (urban and rural) EA sampling frames, which were prepared for each region and city administration by the Ethiopian Statistical Services (ESS). The number of EAs required per region and city administration was determined based on the estimated sample size for each area, with the goal of allocating a maximum of 30 households per EA. The required number of EAs was then allocated to the urban and rural strata based on the proportion of the national population living in urban (21.4%) and rural (78.6%) areas.

The survey, was conducted from May to July 2023 by five Capacity Building and Mentorship Partnership (CBMP) universities: Addis Ababa, Jimma, Haramaya, Hawassa, and University of Gondar. In addition, The Immunization Inter-agency Coordinating Committee (ICC), the National Immunization Technical Advisory Group (NITAG), and the Immunization Technical Working Group (TWG) were involved in the study process. The data owned by Ministry of health was shared on May 20, 2024 for this research purpose.

This national immunization program evaluation survey aimed to estimate the performance of the immunization program. Various maternal/care giver and child health-related characteristics including **S**ocioeconomic and Demographic variables(residency, religion, marital status, mothers/caregivers educational status, Occupation of mothers’/caregivers’, Birth order, wealth Index), health service utilization(ANC follow up, History of maternal tetanus diphtheria (Td) vaccine, Place of delivery, Postnatal care (PNC), Mother/caregiver’s perceived benefits on Immunization and trust on Healthcare providers and distance to vaccination sites were among the collected variables. Immunization cards/health facility registration books/ mothers or caregivers’ recall for the most recent birth were used to assess vaccination status. If a mother reported a child is vaccinated but could not provide the immunization card, health facility records were reviewed. As a last resort, caregiver recall was used. Geographic location data was gathered at the household level, but for analysis purposes, we computed the centroid latitude and longitude for each enumeration area or cluster. Data on zero-dose status was extracted for children aged 12–23 months.

### Measurements

The outcome variable zero-dose was assessed for children born within 1–2 years prior to the survey. Zero-dose was defined as children aged 12–23 months who did not receive the first dose of the DPT containing vaccine [5,44]. Respondents who received the first dose of DPT containing vaccine were categorized as “Yes” or else as “No.” The explanatory variables such as wealth index, region, religion, residency, marital status, mother/caregivers’ educational status, occupation of mothers’/caregivers’, birth order, ANC follow up, history of maternal tetanus diphtheria (Td) vaccine, distance to facility, mother/care givers perceived benefits on immunization, postnatal care (PNC) and place of delivery were included in the analysis.

### Data analysis

The collected dataset was cleaned and preprocessed to ensuring compatibility with geographic analysis. All variables included in this analysis were weighted to handle the effect of selection, and non-response bias. After weighting, the analysis included a total of 6,212 children aged 12–23 months from 463 enumeration areas (EAs), comprising 99 EAs from urban settings and 364 EAs from rural areas. Descriptive statistics like frequency, and percentage were used to describe participants’ socio-demographic characteristics and zero-dose prevalence.

The outcome variable of interest was whether a child aged 12–23 months received the first dose of the pentavalent vaccine or not. The predictor variables included in this study were chosen based on established correlations and predictive factors found in existing literature, along with their biological relevance. Wealth index was computed as a composite index of living standard [6]. It was developed based on ownership of valuable assets and livestock, size of land for agriculture and housing purposes, and materials used for house construction.

The spatial dependency of the outcome was assessed at the cluster level using the global Moran’s Index. This spatial autocorrelation analysis calculated the Moran’s Index, z-score, and associated p-values for both observed and expected index values, taking into account the number of features and their variance. A positive Moran’s Index indicates a tendency toward clustering, whereas a negative Moran’s Index suggests a tendency toward dispersion and value closer to 0 it implicates a random pattern [45]. Anselin Local Moran’s I was used to identify the presence of local level cluster locations of zero-dose children and it measures whether there were positively correlated (high-high and low-low) clusters or negatively correlated (high-low and low-high) clusters which are called outliers [46,47].

In addition, an optimized hot spot analysis technique was employed to pinpoint areas with statistically significant clusters of zero-dose children, both high (hot spots) and low (cold spots). The Optimized Hot Spot Analysis, which builds upon the traditional Hot Spot Analysis (Getis-Ord Gi* statistic), focuses on identifying spatial clustering by analyzing the distribution of features alongside their neighboring counterparts. Furthermore, it incorporates adjustments for multiple testing and spatial dependency through the False Discovery Rate (FDR) correction method [48].

The spatial scan statistical method identified clusters of zero-dose children using Kuldorff’s SaTScan 9.7 software. It analyzed unvaccinated children as cases and vaccinated ones as controls under the Bernoulli model. The maximum scanning window was based on a percentage of the at-risk population, ensuring no small or large clusters were overlooked. Clusters were identified using p-values and likelihood ratio tests. The cluster demonstrating the highest likelihood ratio was designated as the most likely cluster [49].

Also, spatial interpolation technique was applied to predict zero-dose at unknown (non-sampled) locations using values at the measured (sampled) locations. The Kriging interpolation method was chosen because it is an optimal interpolator that minimizes both the mean error (ME) and the root mean square error (RMSE) [50].

To examine the factors explaining zero-dose children, we ran three different regression models. The conventional Ordinary Least Square (OLS) was used as baseline model with classical assumptions of linear regression such as no multicollinearity (VIF < 10 [51,52]. Koenker statistics was significant (p-value of 0.05), indicating the non-stationarity which means the associations between the dependent and independent variables varies across locations. Ordinary Least Squares (OLS) models often overlook the spatial autocorrelation that frequently exists in spatial data, which limits their effectiveness in explaining spatial phenomena. To overcome this limitation, Spatial Regression Models, such as Spatial Lag Models (SLM) and Spatial Error Models (SEM), was used to incorporate spatial autocorrelation, either within the dependent variables (SLM) or in the residuals (SEM). We employed the same set of explanatory variables to run spatial regression models using GeoDa version 1.20 [53,54]. After comparing the R-squared and AIC values, we determined that Spatial Error Models were more suitable than Spatial Lag Models for the final comparison between OLS and spatial regression models.

Finally, Geographic weighted regression (GWR) was employed to identify and quantify the spatial correlates of an independent variable based on the spatial proximity or distance among observations. GWR is particularly useful for addressing spatial non-stationarity, which refers to the presence of different relationships at various locations in space [55]. To evaluate the models, the adjusted R-squared and corrected Akaike Information Criteria (AICc) were used to compare the Ordinary Least Squares (OLS) global model with the GWR local model. The model that best fit the data was determined by the lowest AICc value and the highest adjusted R-squared value [56].

### Ethics approval and consent to participants

The protocol of the study was reviewed and approved by the Institutional Ethical Review Board of University of Gondar (Reference №: CMHSSH-UOG IRERC/3/7/2024). Written informed consent was obtained from each mothers/ caregiver during the survey. We used de-identified data (summary data without individuals’ identity) to ensure confidentiality. In addition, to address the ethical issues associated with the geographic locations of study participants, we computed the centroid of the latitude and longitude data. We followed the international standard of strengthening the reporting of cross-sectional studies in epidemiology

## Results

**Fig 1:**
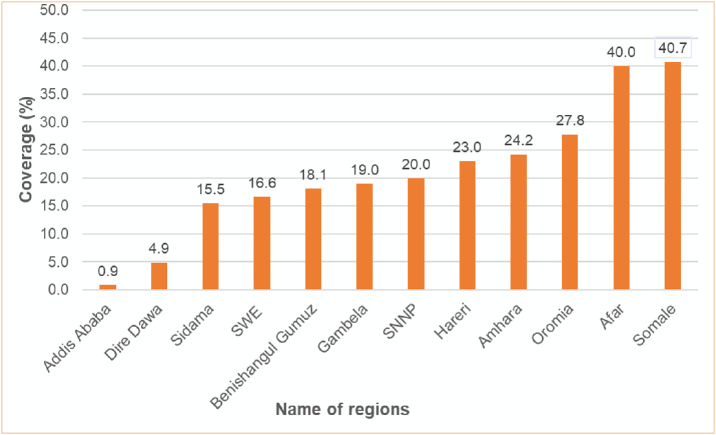
The prevalence of Zero-dose children aged 12-23 months in Ethiopia, 2023 (n = 6212).

A total of 6,212 of children aged 12–23 months were included in the analysis. The overall prevalence of zero-dose in Ethiopia was 24.8% (95%CI: 23.7–25.8%). There were regional variations in the prevalence of zero-dose children. The higher prevalence was observed in Somali (40.7%), Afar (40%) and followed by Oromia (27.8%) and the lowest observed in Addis Ababa (0.9%) and Dire Dawa (4.9%)(Fig 1). High proportion of zero-dose children were found from the rural residents (83.7%) and not educated mother/care givers (60.2%). Majority (93.5%) of children from mother who had ANC visit received first dose of pentavalent vaccine (Table 1).

**Table 1:** Prevalence of Zero-dose stratified by socio-demographic and economic characteristics of mothers/caregivers of children aged 12–23 months in Ethiopia, 2023 (n = 6,212).

Variables	Zero-dose children	
	Yes, number (%)	No, number (%)
**Place of residency**		
Rural	1,286(83.7)	3,439(73.6)
Urban	251(16.3)	1,236(26.4)
**Religion**		
Orthodox	512(33.3)	1830(39.1)
Muslim	800(52.0)	1,822(40.0)
Protestant	208(13.6)	998(21.4)
Others*	17(1.1)	25(0.5)
**Educational status**		
No education	924(60.2)	2,041(43.7)
Primary	401(26.0)	1,508(32.2)
Secondary	151(9.80)	737(15.8)
College and above	61(4.0)	388(8.3)
**Wealth status**		
Poor	499(32.5)	1394(29.8)
Middle	542 (35,2)	1,600(34.2)
Richer	496(32.3)	1,681(36.0)
**Marital status**		
Married and living together	1,449(94.2)	4,403(94.2)
Married but not living together	35(2.3))	116(42.5)
Not in marital union	53(3.5)	157(3.3)
**Birth order**		
1^st^	409(26.6)	1835(39.3)
2^nd^	460(29.9)	1,586(33.9)
3^rd^	289(18.8)	610(13.0)
4^th^ and above	379(24.7)	644(13.8)
**Parity**		
Primipara	308(22.4)	1,028(23.9)
Multipara (2–4)	589(42.8)	2,334(54.3)
Grand multipara (5^+^)	480(34.8)	940(21.8)
**Perceived distance to vaccination site**		
Big problem	1047(68.1)	1402(30.0)
No problem	490(31.9)	3273(70.0)
**Perceived benefit on immunization(n = 5,855)**		
Poor	781(57.5)	275(6.1)
Good	578(42.5)	4221(93.9)
**Antenatal care visits(n = 5,679)**		
Yes	777(56.5)	4023(93.5)
No	600(43.5)	279(6.5)
**Place of delivery(n = 5,679)**		
Home	733(53.2)	942(21.8)
Health facility	644(44.8)	3360(78.1)
**Postnatal care (n = 5,679)**		
Yes	440(32.0)	2030(47.2)
No	937(68.0)	2271(52.8)

Others*: catholic, traditional, others.

### Geographic patterns of zero-dose children

**Fig 2:**
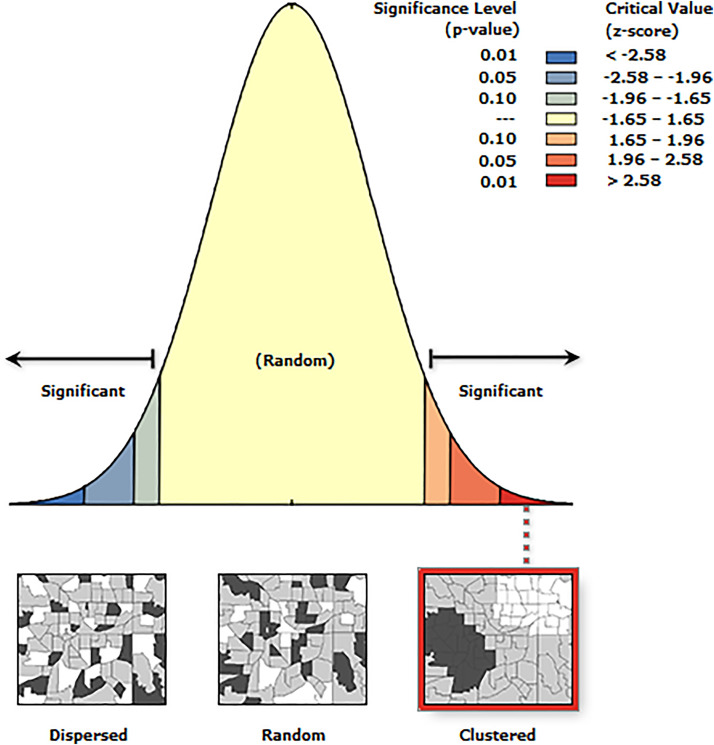
Spatial autocorrelations of Zero-dose children aged 12-23 months in Ethiopia, 2023.

In the spatial autocorrelation analysis, the Global Moran’s I value (I = 0.19), z-score of 3.67 and a p-value = 0.0002 indicated that the prevalence of zero-dose children at cluster level were close to each other. There was less than 1% (99% degree of confidence) likelihood that this clustering could be by chance (Fig 2).

**Fig 3:**
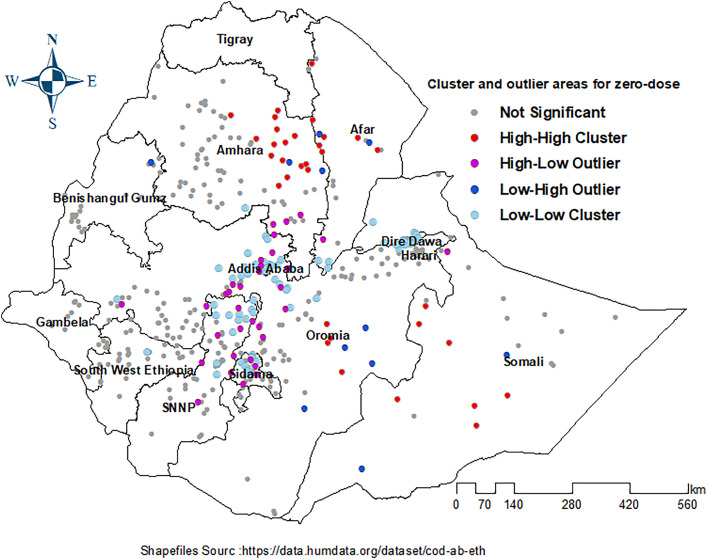
Spatial Cluster and outlier for Zero-dose prevalence among12-23 months children in Ethiopia, 2023.

The Local Moran’s I analysis identified clusters and outliers among children who received no first dose of DTP containing vaccine. The results highlighted both high-high and low-low clusters, indicating areas with significant excess of zero-dose children. Notably, a substantial number of unvaccinated children were concentrated in various regions, including much of Somali, northern and southern Afar, Eastern Amhara, and southern Oromia. Conversely, outliers(high-low), characterized by areas with a large number of zero-dose children surrounded by areas with fewer unvaccinated children, were primarily found in southern Amhara, central and southwestern Oromia, northeastern Somali, Sidama, and the Southern Nations, Nationalities, and Peoples’ Region (SNNP). In contrast, low-high outliers denote clusters where there is a low percentage of zero-dose children encircled by areas with a higher percentage of zero-dose children are detected southwest Somali and Oromia, South-east Afar, northeast Benishangul Gumuz and Eastern Amhara (Fig 3).

**Fig 4:**
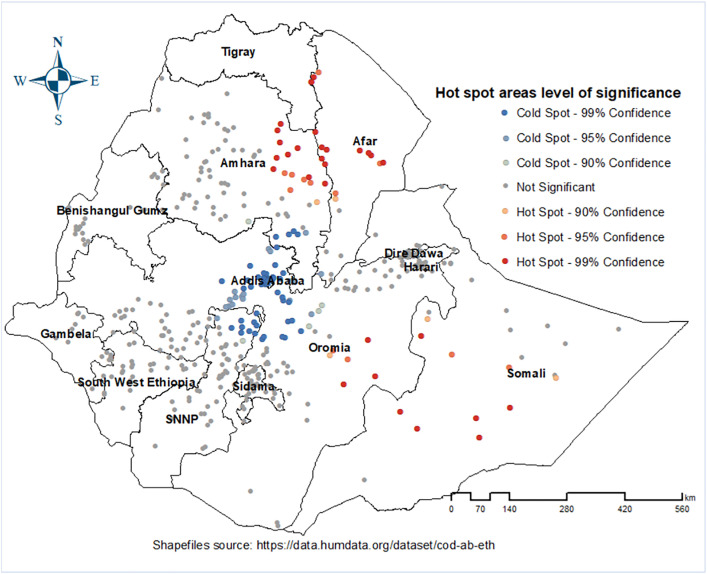
Hotspot areas for Zero-dose children aged 12-23 months in Ethiopia, 2023.

The hotspot analysis detects high proportion of zero-dose children (hot spots) and clusters with a low proportion of zero-dose (cold spot). The higher proportion of zero-dose children (red dots) were prevalent in most parts of Somali, north and southwest Afar, Eastern part of Amhara, and southwest Oromia. Conversely, low proportion of zero-dose (blue dots) was detected in Centeral and northern Oromia, southern Amhara, Addis Ababa, Southern Afara, and northern parts of SNNP (Fig 4).

**Fig 5:**
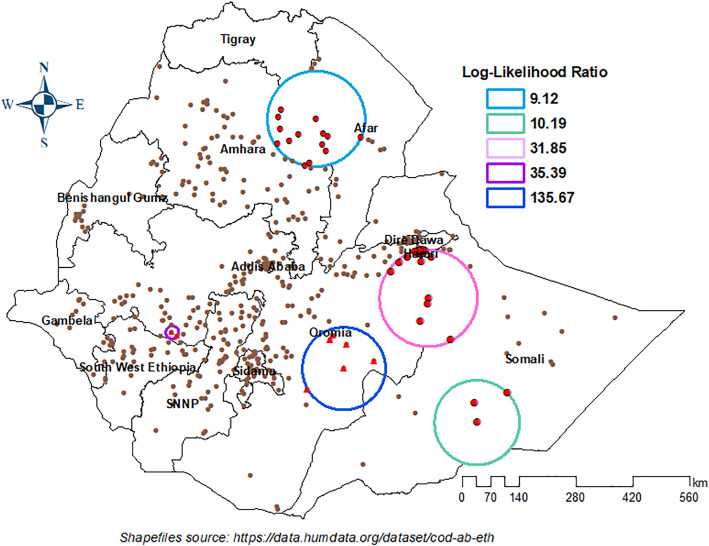
Primary and secondary clusters of zero-dose children aged 12-23 months in Ethiopia, 2023.

The spatial scan statistics analysis identified six statistically significant clusters for zero-dose children. The most likely clusters, also called the primary cluster with at total enumeration area of 6 was found in the southern Oromia region. The primary cluster was pinpointed with Log-Likelihood Ratio of 135.7 spanning an area with a radius of 101.63 km (Table 2). In addition, five most likely secondary clusters circled with a pink, purple, light blue and green color were found in the easter, southern, southwest and southeast parts of the country (Fig 5).

**Table 2:** Significant clusters for zero-dose children aged 12-23 months in Ethiopia, 2023.

Cluster	N	Population	Cases	RR	LLR	P-value	Radius
Primary	6	214	165	3.39	135.7	<0.0001	101.63 km
Secondary 1	2	51	41	3.33	35.39	<0.0001	15.97 km
Secondary 2	23	142	79	2.33	31.85	<0.0001	119.14 km
Secondary 3	3	21	15	2.92	10.2	<0.0098	104.19 km
Secondary 4	121	20	53	1.72	9.1	<0.029	117.53 km

Note: N = number of clusters (EA), RR = relative risk, LLR = log-likelihood ratio.

### Predicted prevalence of zero-dose children

**Fig 6:**
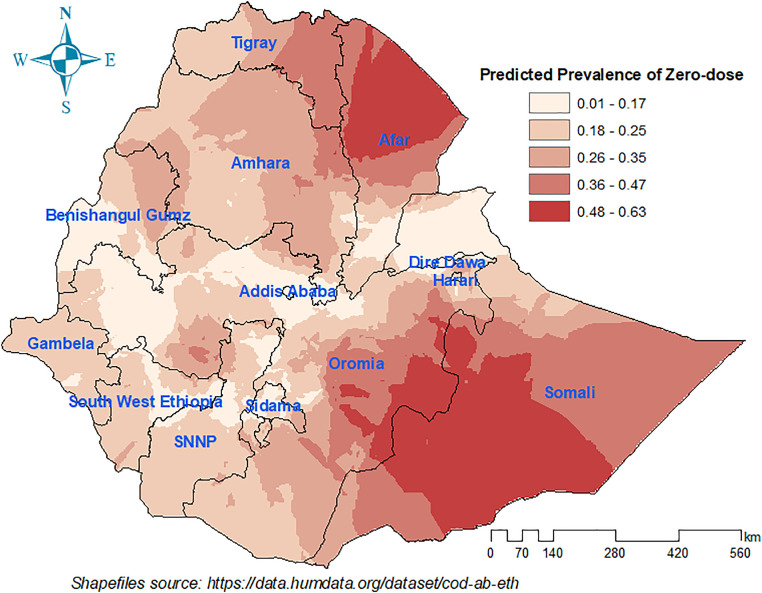
Predicted prevalence of zero-dose children aged 12-23 months using Kriging interpolation in Ethiopia, 2023.

The predicted map evident that most parts of Afar and Somali, southern and southeastern Oromia, as well as eastern Amhara had a relatively high zero-dose prevalence. On the other hand, Addis Ababa, Dire Dawa, the Kefa and Konta special zones in southwestern Ethiopia, the Sidama zone in the Sidama region, Kamashi zone of Benishangul Gumuz region and certain parts of Oromia were identified as low-risk regions for zero-dose children (Fig 6).

The color pattern shows she spatial difference in the prevalence of zero-dose. The deeper red color indicates a higher prevalence of zero-dose children, the lighter indicates the lower prevalence.

### Factors associated with geographic variabilities of zero-dose children

The OLS analysis identified four variables associated with zero-dose children: Lack of ANC utilization, poor perception of the benefit of immunization, no maternal TT/Td vaccination, and distance to facility. There was no indication of multicollinearity detected among the selected independent variables evidenced by a variance inflation factor (VIF) ranged from 1.28–2.98. The OLS model (adjusted R^2^ = 0.646) explained 64.6% of the variation in zero-dose children by the four explanatory variables (Table 3).

**Table 3:** Ordinary least square egression (OLS) results summary.

Variable	Coefficient	Robust t-statistics	Robust probability	VIF
Intercept	0.032	4.211872	<0.0001	--------
No ANC utilization	0.303	4.505514	<0.0001	2.98
poor perception on benefit of immunization	0.379	7.471412	<0.0001	1.75
no maternal TT vaccination	0.151	2.206580	0.028	2.65
and distance to facility as a big problem	0.113	3.796852	<0.0001	1.28
**Ordinary Least Square Regression (OLS) diagnostic**				
Number of observations	463	**P-Value**		
*Joint F-statistics*	212	<0.0001		
*Joint Wald statistics*	682	<0.0001		
*Koenker (BP) statistics*	58	<0.0001		
*Jarque- Bera*	48.4	<0.0001		
*Moran’s I*	0.172	0.0012		
**Model performance**				
Adjusted R-Squared		0.646		
Akaike’s Information Criterion (AICc)		−542.36		

The Koenker statistics in the model is statistically significant indicating that the regression model is inconsistent across the study area (with the change in geographic location the relationship of variables will also change) implies there is the non-stationarity of the variance. The results obtained from the OLS method prompt us to utilize spatial regression analysis alongside the GWR model, which we deem more suitable for estimating the model parameters (Table 3).

The Spatial Error (SER) model demonstrated superior performance with a more robust overall fit, achieving an adjusted R² of 66.5%, which surpasses the Ordinary Least Squares (OLS) model’s 64.9% and the Spatial Lag model’s 65%. The AICc (−558) and BIC (537.9) of the SEM is lower than the OLS and SLM. The local GWR model with a highest Adjusted R2 (0.77) and a lowest AICc(−628) better performed than the OLS, SLM and SER. The GWR model explained 77% of the variation of zero-dose children prevalence, significantly outperforming than baseline OLS model. In addition, the spatial autocorrelation observed from the GWR output (Moran’s I = 0.01, P-value = 0.179) suggests that the GWR model is fitting the data well, as the residuals do not exhibit significant spatial patterns (Table 4). The mean coefficient of all explanatory variables were positive. Indicating a positive association between zero-dose children and the four explanatory variables (no ANC utilization, poor perception on immunization, No maternal Td vaccine and perceived distance to facility as far).

**Table 4:** Model comparisons among the ordinary least square, spatial error, SEM-Spatial error and geographical weighted regression models.

Predictors	Zero-dose children			
	OLS (coef, p-value)	SLM (coef, p-value)	SEM (coef, p-value)	GWR
No ANC utilization	0.303(0.000)	0.304(0.000)	0.301(0.000)	
poor perception on immunization	0.379(0.001)	0.15(0.001)	0.39(0.000)	
no maternal TT vaccination	0.151(0.000)	0.37(0.000)	0.16(0.000)	
Distance to facility as a big problem	0.113(0.000)	0.11(0.000)	0.12(0.000)	
LL	277.5	278.7	284.3	
Moran’s I (value, P-value)-Residuals	0.172(0.001)	0.09(0.79)	0.002(0.48)	0.01(0.18)
BIC	−524.3	−520.6	−537.9	
AIC	−542.4	−545.4	−558	−628
Adjusted R²	0.646	0.65	0.67	0.77

***OLS-Ordinary Least Square Model, SLM-Spatial Lag Model, SEM-Spatial Error Model, GWR-Geographic Weighted Regression.***

**Fig 7:**
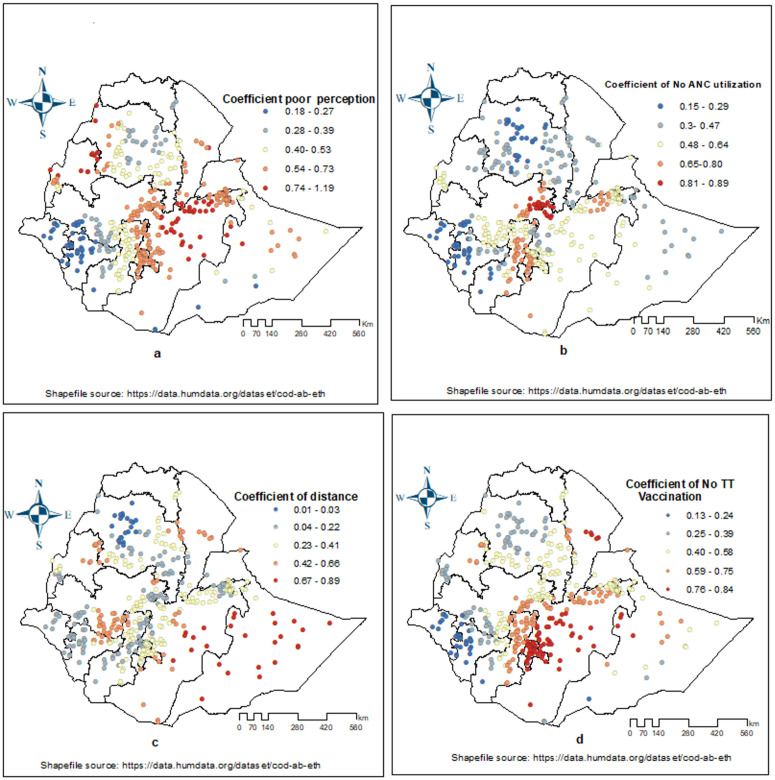
The spatial mapping of local regression coefficients of explanatory variables on Zero-dose children aged 12-23 months in Ethiopia, 2023.

Fig 7 (a, b, c and d) display the GWR coefficients for each explanatory variable. These coefficients reveal that the relationship between independent variables and the prevalence of zero-dose children varies significantly by location. Areas marked with red, orange, and yellow dotted points demonstrate a considerable impact of these variables on zero-dose children. The risk of a child being unvaccinated increases with the number of mothers who do not utilize antenatal care (ANC) varies across regions. The higher coefficient found in Addis Ababa, Oromia, Somali, Sidama, Benishangul Gumuz, SNNP, Dire Dawa, Harari, and Amhara (Fig 7a).

Perception on benefits of immunization was strongly linked with zero-dose children and causes higher in most parts of the country mainly eastern and southeastern Amhara, the majority of Oromia, Sidama, Sidam, Somali, SNNP and Afar (Fig 7b).

The coefficient of mother/care givers who perceived distance to immunization site is far reveals a positive correlation predominantly observed in the Afar, Somali, Benishangul Gumuz, Oromia, SNNP and some parts of Amhara (Fig 7c). Similarly, a notable association of maternal TT/Td vaccination and prevalence zero-dose children was identified across the country and the strong relation was observed in most parts of Oromia, Somali, SNNP, and Afar (Fig 7d).

## Discussion

This study aimed to assess the geographic inequities of zero-dose children and its determinants among children aged 12–23 months. In this study, we found that one in four children didn’t take the first dose of DPT containing vaccines. This finding slightly surpasses the joint estimates of immunization Coverage for 2023 as reported by the World Health Organization (WHO) and the United Nations International Children’s Emergency Fund (UNICEF) [57]. This is also higher than the mini EDHs report of 2019 [6]. The result suggests that the country has lagged behind the target in reducing the number of the number of zero-dose children. This highlights the urgent need for a proactive and comprehensive strategy to address the significant issue of unvaccinated children.

This study identified sub-national disparities in the prevalence of zero-dose children across Ethiopia. We found distinct hotspots of high zero-dose prevalence and cold spots where prevalence was low. Notably, many hotspots were concentrated in the pastoralist regions, Specifically Afar and Somali. In contrast, cold spots were observed in central and northern Oromia, southern Amhara, Addis Ababa, and the northern parts of Southern Nations, Nationalities, and Peoples regions. These findings underscore significant regional inequality in zero-dose prevalence.

This spatial pattern is consistent with previous studies, which also points to marked regional disparities in zero-dose prevalence and immunization coverage. A high burden of the unvaccinated children concentrated in the pastoralist regions of Afar and Somali [58]. Similarly, other studies on geographical disparities in incomplete immunization have identified these same areas as major hotspot regions [59,60]. These regions face considerable challenges with the accessibility and availability of health services, compounded by persistent quality gaps in healthcare delivery [61,62].

This stark geographic disparity highlights not only existing inequities but also raises important questions about the underlying factors contributing to this variation. Such inequalities can be attributed to a combination of socio-economic and healthcare related factors. These include, but are not limited to, place of residence, maternal health utilization, and the accessibility of health facilities [1,13,15,16].

This study also sought to identify the predictor variables linked to zero-dose children. Antenatal care (ANC) service utilization, perceptions on immunization benefits, maternal tetanus toxoid (TT) vaccination, and the distance to immunization sites were significant factors contributing to the prevalence of zero-dose children in Ethiopia. The findings of this study indicated that lack of antenatal care (ANC) utilization significantly correlated with a high prevalence of zero-dose children. This is in agreement with previous study examined the prevalence, spatial variation, and determinants of zero-dose children [58]. Children whose mothers did not attend ANC were more likely to be zero-dose. This is likely becaues ANC visits provides mothers to access integrated health services, more likely to receive information on immunization schedules, build trust in the health system and improve adherence to health services [63–65]. Furthermore, thes study reaveld that the influence of ANC utilization on zero-dose children varies geographically. The correlation was strongest in Oromia, the SNNP, Addis Ababa, and Afar regions underscoring the importance of localized strategies to address barriers to child immunization.

This study also revealed that maternal TT/Td vaccination significantly influence the prevalence of zero-dose children, consistent with previous study [64,65]. One possible explanation is that maternal vaccination increases access to healthcare, fostering a trusting relationship between mothers and healthcare providers. This trust can enhance communication regarding immunization schedules and the importance of vaccinating children. The strongest correlations for TT/Td vaccination were observed in the Oromia, Somali, Afar, and SNNP regions, showing its effects geographically.

Another significant finding was the strong link between mothers’ and caregivers’ perceptions of vaccinations and the prevalence of zero-dose children. Consistently, Previous studies [66,67] has shown that individuals with negative perceptions on immunization benefits are less likely to vaccinate their children. This relationship underscores the critical role beliefs and attitudes play in healthcare decision-making. This tendency can lead to significant gaps in immunization coverage, ultimately contributing to higher rates of vaccine preventable diseases. Our analysis revealed that the highest coefficient, indicating a strong negative impact of poor perceptions regarding the benefits of immunization, was found in regions such as Somali, eastern and southern Amhara, Sidama, Afar, and some parts of Oromia. These areas face unique challenges, including cultural beliefs, misinformation, and limited access to accurate health information, which can intensify negative attitudes towards vaccinations. Addressing perception as a factor for zero-dose children may require a multifaceted and local specific tailored approach. This should combine education, and community engagement aiming to increase the awareness of mother/care givers on the benefits of immunization.

Additionally, our study found that the distance to vaccination site significantly influences the prevalence of zero-dose, aligning with previous studies highlighting geographical barriers as primary factors to immunization coverage [68,69]. The highest coefficients were observed in the Somali, Afar, Benishangul and Some parts of Oromia region. The Somali and Afar regions are known for significant challenges in accessing and utilizing health services [61,62]underscoring persistent disparities in the healthcare services. To address this, context-specific strategies are important for these pastoralist regions to identify and reach zero-dose children. Existing vaccination strategies should be strengthened and evaluated to determine their suitability for these areas.

This study had several strengths worth mentioning. We used national level survey data from 463 enumeration areas ensuring generalizability across the country and providing a current a current snapshot of the zero-dose situation. A key strength is the use of advanced geo-statistics to identify the most risk areas and the risk factors considering the spatial distribution and dependence of zero-dose prevalence. The spatial heterogeneity of the model parameters has been accounted using the spatial regression analysis and GWR. At the same time, this study identified the risk factors of zero-dose which may help policy makers and planners to design tailored interventions to identify and reach the unvaccinated children in the geographically high where the zero-dose is eventually high compared to the other parts of the country.

This study was subject to some limitations. Firstly, though we used a national level data and we predicted the prevalence of zero-dose for the unsampled sites, we didn’t included data from Tigray region which is one of the administrative regions of the country data due to security issues. Secondly, distance was assessed through subjective measures. By asking participants about distance as far or near to immunization sites. Objective measure of distance, such actual distance from home to immunization site or actual travel times(reference), may provide a more accurate representation of accessibility and its impact on zero-dose prevalence. Lastly, the perceptions of mothers and caregivers regarding the benefits of immunization were measured using binary yes/no questions. However, this approach may oversimplify their views and could result in failing to capture a more subtle understanding of their attitudes towards immunization.

## Conclusion

Despite significant efforts in Ethiopia’s immunization program, the proportion of unvaccinated children remains unacceptably high, with one-fourth of children still not immunized. Our analysis indicated notable regional disparities in the prevalence of zero-dose children. The results also highlight associations between zero-dose prevalence and factors such as ANC utilization, perceptions of immunization benefits, TT/Td vaccination, and distance to immunization sites. These associations vary across regions, underscoring the need for tailored interventions to identify and reach zero-dose children. This approach can help fulfill the national and global immunization agenda of leaving no one behind by ensuring equitable access to vaccinations. Additionally, regional governments should strengthen maternal health services through various health promotion activities, which can also raise awareness about the benefits of immunization.

## Supporting information

S1 Dataset Dataset of Spatial Disparities in Zero-Dose Vaccination Coverage for Children Aged 12–23 Months in Ethiopia: A Geographically Weighted Regression Analysis. (DTA)
