# Supplementary material for: Spatial disparities in zero-dose vaccination coverage for children aged 12–23 months in Ethiopia: A geographically weighted regression analysis
Source: PLoS One. 2025 Sep 11;20(9):e0332162. doi: 10.1371/journal.pone.0332162 (PMC12425285; doi:10.1371/journal.pone.0332162)

PLOS ONE

Spatial Disparities in Zero-Dose Vaccination Coverage for Children Aged 12-23 Months in Ethiopia: A Geographically Weighted Regression Analysis

--Manuscript Draft--

**Manuscript Number:**	PONE-D-24-59668
**Article Type:**	Research Article
**Full Title:**	Spatial Disparities in Zero-Dose Vaccination Coverage for Children Aged 12-23 Months in Ethiopia: A Geographically Weighted Regression Analysis
**Short Title:**	Spatial Disparities in Zero-Dose Children Aged 12-23 Months in Ethiopia: Geographically Weighted Regression
**Corresponding Author:**	Berhanu Endehabtu<br>University of Gondar College of Medicine and Health Sciences<br>6200-Gondar, ETHIOPIA
**Keywords:**	Geographic inequity; zero-dose children; Spatial Analysis; Ethiopia
**Abstract:**	Background<br><br>Though Ethiopia has made significant progress in childhood vaccination, many children remain unvaccinated, making it the third largest contributor to the global burden of zero-dose children those who doesn't receive the first dose of diphtheria, tetanus and pertussis containing vaccine. Identifying geographic inequities of zero-dose prevalence and the factors influencing it could help to effectively reach and identify at-risk children and to design tailored intervention.<br><br>Objectives<br><br>This study aimed to assess the geographical inequities and predicting factors of zero-dose children aged 12-23 month in Ethiopia.<br><br>Methods<br><br>We used a population-based survey data. A total of 6,212 children aged 12-23 were included. The spatial autocorrelation was employed to examine geographic variations in zero-dose children. Getis-Ord Gi* statistics was used for hotspot analyses. A Kriging interpolation technique used to estimate values of zero-dose at unmeasured locations based on known values of zero-dose at observed locations. The Geographic Weighted Regression (GWR) analysis was used to elicit determinants of geographic difference in zero-dose children. Adjusted R2 and Akaike Information Criteria (AICc) were used to compare the models.<br><br>Results<br><br>The prevalence of zero-dose children was 24.8% [CI: 23.7%-25.8%] ranged from 0.9% in Addis Ababa to 40.7% in Somali region. The zero-dose prevalence varied across the study area (Moran's I=0.193; P-value<0.0001). Significantly higher proportions of zero doses (hotspot areas) were identified in the north and south Somali, northwest Afar, East Amhara, and southern Oromia regions. GWR analysis showed that no ANC utilization, no TT/Td vaccination, poor perceptions on immunization, and far distance to healthcare facilities contributed to these geographic variations.<br><br>Conclusion<br><br>This study revealed that the prevalence of Zero-dose is unacceptably high, with geographic inequities varying across the country. Factors such as ANC utilization, TT/Td vaccination, perceptions of immunization, and distance to healthcare facilities contributed to these geographic differences. This underscores the importance of designing and implementing tailored interventions to identify and reach zero-dose children. Such an approach could help achieve the national and global immunization goal of leaving no one behind by providing equitable access to immunization.

Powered by Editorial Manager® and ProduXion Manager® from Aries Systems Corporation

Order of Authors:	Berhanu Fikadie Endehabtu, MPH
	Kassahun Alemu
	Shegaw Angaw Mengiste
	Mesert Zelalem
	Monika Knudsen Gullslett
	Biniyam Tilahun
Additional Information:	
Question	Response
**Financial Disclosure**<br><br>Enter a financial disclosure statement that describes the sources of funding for the work included in this submission. Review the submission guidelines for detailed requirements. View published research articles from *PLOS ONE* for specific examples.<br><br>This statement is required for submission and **will appear in the published article** if the submission is accepted. Please make sure it is accurate.<br><br>**Funded studies**<br>Enter a statement with the following details:<br>• Initials of the authors who received each award<br>• Grant numbers awarded to each author<br>• The full name of each funder<br>• URL of each funder website<br>• Did the sponsors or funders play any role in the study design, data collection and analysis, decision to publish, or preparation of the manuscript?<br><br>Did you receive funding for this work?	Yes
Please add funding details.<br>as follow-up to "**Financial Disclosure**<br><br>Enter a financial disclosure statement that describes the sources of funding for the work included in this submission. Review the submission guidelines for detailed requirements. View published research articles from *PLOS ONE* for specific examples.	This study was done using a grant awarded by University of Gondar, Ethiopia (Reference № AC/V/P/C/02/1775/2024). The funder had no role in the study design, data collection and analysis, decision to publish, or preparation of the manuscript.

This statement is required for submission and **will appear in the published article** if the submission is accepted. Please make sure it is accurate.<br><br>**Funded studies**<br>Enter a statement with the following details:<br>• Initials of the authors who received each award<br>• Grant numbers awarded to each author<br>• The full name of each funder<br>• URL of each funder website<br>• Did the sponsors or funders play any role in the study design, data collection and analysis, decision to publish, or preparation of the manuscript?<br><br>Did you receive funding for this work?"	
Please select the country of your main research funder (please select carefully as in some cases this is used in fee calculation).<br>as follow-up to "**Financial Disclosure**<br><br>Enter a financial disclosure statement that describes the sources of funding for the work included in this submission. Review the submission guidelines for detailed requirements. View published research articles from *PLOS ONE* for specific examples.<br><br>This statement is required for submission and **will appear in the published article** if the submission is accepted. Please make sure it is accurate.	ETHIOPIA - ET

**Funded studies**<br>Enter a statement with the following details:<br>• Initials of the authors who received each award<br>• Grant numbers awarded to each author<br>• The full name of each funder<br>• URL of each funder website<br>• Did the sponsors or funders play any role in the study design, data collection and analysis, decision to publish, or preparation of the manuscript?<br><br>Did you receive funding for this work?"	
**Competing Interests**<br><br>Use the instructions below to enter a competing interest statement for this submission. On behalf of all authors, disclose any competing interests that could be perceived to bias this work—acknowledging all financial support and any other relevant financial or non-financial competing interests.<br><br>This statement is required for submission and **will appear in the published article** if the submission is accepted. Please make sure it is accurate and that any funding sources listed in your Funding Information later in the submission form are also declared in your Financial Disclosure statement.<br><br>View published research articles from *PLOS ONE* for specific examples.	The authors declare that they have no competing interests.

**NO authors have competing interests**<br><br>Enter: *The authors have declared that no competing interests exist.*<br><br>**Authors with competing interests**<br><br>Enter competing interest details beginning with this statement:<br><br>*I have read the journal's policy and the authors of this manuscript have the following competing interests: [insert competing interests here]*<br><br>* typeset	
**Ethics Statement**<br><br>Enter an ethics statement for this submission. This statement is required if the study involved:<br><br>• Human participants<br>• Human specimens or tissue<br>• Vertebrate animals or cephalopods<br>• Vertebrate embryos or tissues<br>• Field research<br><br>Write "N/A" if the submission does not require an ethics statement.<br><br>General guidance is provided below. Consult the submission guidelines for detailed instructions. **Make sure that all information entered here is included in the Methods section of the manuscript.**	Yes. The ethical approval uploaded. The protocol of the study was reviewed and approved by the Institutional Ethical Review Board of University of Gondar (Reference №: CMHSSH-UOG IRERC/3/7/2024). We used de-identified data (summary data without individuals' identity) to ensure confidentiality. Written informed consent was obtained from each mothers/ caregiver during the survey. We followed the international standard of strengthening the reporting of cross-sectional studies in epidemiology.

Powered by Editorial Manager® and ProduXion Manager® from Aries Systems Corporation

**Format for specific study types**<br><br>**Human Subject Research (involving human participants and/or tissue)**<br>• Give the name of the institutional review board or ethics committee that approved the study<br>• Include the approval number and/or a statement indicating approval of this research<br>• Indicate the form of consent obtained (written/oral) or the reason that consent was not obtained (e.g. the data were analyzed anonymously)<br><br>**Animal Research (involving vertebrate animals, embryos or tissues)**<br>• Provide the name of the Institutional Animal Care and Use Committee (IACUC) or other relevant ethics board that reviewed the study protocol, and indicate whether they approved this research or granted a formal waiver of ethical approval<br>• Include an approval number if one was obtained<br>• If the study involved *non-human primates*, add *additional details* about animal welfare and steps taken to ameliorate suffering<br>• If anesthesia, euthanasia, or any kind of animal sacrifice is part of the study, include briefly which substances and/or methods were applied<br><br>**Field Research**<br><br>Include the following details if this study involves the collection of plant, animal, or other materials from a natural setting:<br>• Field permit number<br>• Name of the institution or relevant body that granted permission	
**Data Availability**<br><br>Authors are required to make all data underlying the findings described fully available, without restriction, and from the time of publication. PLOS allows rare exceptions to address legal and ethical concerns. See the PLOS Data Policy and FAQ for detailed information.	Yes - all data are fully available without restriction

A Data Availability Statement describing where the data can be found is required at submission. Your answers to this question constitute the Data Availability Statement and **will be published in the article**, if accepted.<br><br>**Important:** Stating 'data available on request from the author' is not sufficient. If your data are only available upon request, select 'No' for the first question and explain your exceptional situation in the text box.<br><br>Do the authors confirm that all data underlying the findings described in their manuscript are fully available without restriction?	
**Describe where the data may be found in full sentences. If you are copying our sample text, replace any instances of XXX with the appropriate details.**<br><br>• If the data are **held or will be held in a public repository**, include URLs, accession numbers or DOIs. If this information will only be available after acceptance, indicate this by ticking the box below. For example: *All XXX files are available from the XXX database (accession number(s) XXX, XXX.).*<br>• If the data are all contained **within the manuscript and/or Supporting Information files**, enter the following: *All relevant data are within the manuscript and its Supporting Information files.*<br>• If neither of these applies but you are able to provide **details of access elsewhere**, with or without limitations, please do so. For example:<br><br>*Data cannot be shared publicly because of [XXX]. Data are available from the XXX Institutional Data Access / Ethics Committee (contact via XXX) for researchers who meet the criteria for access to confidential data.*<br><br>*The data underlying the results presented in the study are available from (include the name of the third party*	All relevant data are within the manuscript and its Supporting Information file.

and contact information or URL).<br>• This text is appropriate if the data are owned by a third party and authors do not have permission to share the data.<br><br>* typeset	
Additional data availability information:	

Manuscript-Revised

Click here to access/download;Manuscript;Manuscript_Zero_Dose_Jan-

Spatial Disparities in Zero-Dose Vaccination Coverage for Children Aged 12-23 Months in Ethiopia: A Geographically Weighted Regression Analysis

Berhanu Fikadie Endehabtu[1,2]*, Kassahun Alemu[2,3], Shegaw Angaw Mengiste[4], Mesert Zelalem[5,6], Monika Knudsen Gullslett[7], Biniyam Tilahun[1,2]

Author`s Affiliation

[1]Department of Health Informatics, Institute of Public Health, College of Medicine and Health Sciences, University of Gondar, Gondar, Ethiopia

[2]Center for Digital Health and Implementation Science, University of Gondar, Gondar, Ethiopia

[3]Department of Epidemiology and Biostatistics, Institute of Public Health, College of Medicine and Health Sciences, University of Gondar, Gondar, Ethiopia

[4]School of Business, University of South-Eastern Norway, Drammen, Norway

[5]Adjunct Assistant Professor of Pediatrics and Child Health, University of Gondar, Gondar, Ethiopia

[6]Deputy Chief Executive Officer, Chief of Programs and Research African Center for Early Childhood Development, Addis Ababa, Ethiopia

[7]Norwegian Centre for E-health Research, University Hospital of North Norway, Tromsø, Norway

*** Corresponding author**

Berhanu Fikadie Endehabtu

Department of Health Informatics, Institute of Public Health, College of Medicine and Health Sciences, University of Gondar, Gondar, Ethiopia.

Email: berhanufikadie@gmail.com, berhanu.fikadie@cdhi-uog.edu.et

Abstract

Background: Though Ethiopia has made significant progress in childhood vaccination, many children remain unvaccinated, making it the third largest contributor to the global burden of zero-dose children those who doesn't receive the first dose of diphtheria, tetanus and pertussis containing vaccine. Identifying geographic inequities of zero-dose prevalence and the factors influencing it could help to effectively reach and identify at-risk children and to design tailored intervention.

Objectives: This study aimed to assess the geographical inequities and predicting factors of zero-dose children aged 12-23 month in Ethiopia.

Methods: We used a population-based survey data. A total of 6,212 children aged 12-23 were included. The spatial autocorrelation was employed to examine geographic variations in zero-dose children. Getis-Ord Gi* statistics was used for hotspot analyses. A Kriging interpolation technique used to estimate values of zero-dose at unmeasured locations based on known values of zero-dose at observed locations. The Geographic Weighted Regression (GWR) analysis was used to elicit determinants of geographic difference in zero-dose children. Adjusted R2 and Akaike Information Criteria (AICc) were used to compare the models.

Results: The prevalence of zero-dose children was 24.8% [CI: 23.7%-25.8%] ranged from 0.9% in Addis Ababa to 40.7% in Somali region. The zero-dose prevalence varied across the study area (Moran's I=0.193; P-value<0.0001). Significantly higher proportions of zero doses (hotspot areas) were identified in the north and south Somali, northwest Afar, East Amhara, and southern Oromia regions. GWR analysis showed that no ANC utilization, no TT/Td vaccination, poor perceptions on immunization, and far distance to healthcare facilities contributed to these geographic variations.

Conclusion: This study revealed that the prevalence of Zero-dose is unacceptably high, with geographic inequities varying across the country. Factors such as ANC utilization, TT/Td vaccination, perceptions

of immunization, and distance to healthcare facilities contributed to these geographic differences. This underscores the importance of designing and implementing tailored interventions to identify and reach zero-dose children. Such an approach could help achieve the national and global immunization goal of leaving no one behind by providing equitable access to immunization.

Keywords: Geographic inequity, zero-dose children, spatial analysis, Ethiopia

Introduction

Immunization is one of the most successful and cost-effective interventions saving millions of live every year and ensuring global wellbeing (1). Between 2021 and 2030, routine immunization is projected to avert an estimated 5.1 million child death globally(2). Despite a remarkable increase in childhood immunization coverage globally, an estimated 14.5 million children did not receive the first dose of DPT containing vaccines (zero-dose children) in 2023 (3). Approximately 60% of these zero-dose children were found to be living in ten low-and middle-income countries (LMICs), including Ethiopia (3, 4).

Zero-dose children, defined as those who have not received a first dose of Diphtheria Tetanus and Pertussis (DTP-1) vaccine (4, 5) are a key focus for global health policy makers. Although, Ethiopia has made commendable progress in immunization coverage (6), it remains one of the ten countries with the highest number of for un- and under-vaccinated children globally. In Ethiopia, approximately 917,000 children are estimated to haven't received the first dose of DPT containing vaccines, accounting for 6% of the global burden of zero-dose children (7). These zero-dose children are at high risk of mortality from vaccine preventable disease (VPDS) and a potential source of vaccine preventable disease outbreaks (8-11). These unvaccinated children are also often disadvantaged of other basic services and suffer from rooted inequality (12-14). These children mainly reside in marginalized or disadvantaged communities marked by poverty, limited access to essential health services, urban slums, inadequate sanitation, and conflict affected areas (1, 13, 15, 16). These factors, along with socioeconomic, demographic, and health service related factors, contribute significantly to the uneven distribution of zero-dose children within countries (13).

Addressing the issue of zero-dose children is vital for meeting both the national and global agenda in reducing the number of zero-dose children by 50% by the year 2030. This effort aligns with the commitment to "leave no one behind" (1) and Gavi, the Vaccine Alliance's strategy for 2021–2025 (17).

Realizing equitable access to child health services is the priority for international development (18). Reducing socioeconomic and geographic inequities in access to health care is a key for countries to achieve Universal Health Coverage (19). Closing the immunization equity gap must start with zero-dose children and missed communities (12).

The geographic inequities of immunization coverage can obstruct the fight against VPDs (20). Existing evidences showed the presence of geographic disparities in immunization Coverage(21-30). Spatial analysis as a statistical tool helps to pinpoint clusters of events, helps to identify areas that are more susceptible to health risks (30-32). Spatial analyses of data from various surveys conducted in the Ethiopia (33-37) have shown a persistent regional divide in immunization coverage. Recent interest in studying spatial inequities in childhood immunization seeks to uncover gaps in immunization coverage, facilitating targeted efforts to effectively close these gaps (38-40). In addition, aggregate-level analysis masks inequities in coverage, granular analysis can help identify persistently missed hot spots (areas with high unvaccinated zero-dose) (24, 41, 42).

However, there is limited evidence revealing the geographic inequities of zero-dose children at the national level and the factors contributing to these inequities. Identifying the geographic disparities in zero-dose coverage and the factors influencing them could help effectively reach and identify at-risk children, as well as design tailored interventions. Therefore, this study aimed to examine the geographic inequities and local predictors of zero-dose children aged 12–23 months in Ethiopia.

Methods and Materials

Study design and setting

This study was a secondary analysis of a national immunization program evaluation 2023. Ethiopia is located at (3°-14^oN and 33° – 48°E) the horn of Africa. Administratively, the country is divided into eleven

regions and two city administrations. Further, each region is divided into zones, woredas and kebeles (the smallest administrative unit). This study included all regions and two city administrations of the country, with the exception of the Tigray region due to internal conflicts.

Data sources

We conducted an analysis using nationally representative data from a recent immunization program assessment survey in Ethiopia, covering 10 regions and two city administrations. The data was accessed for research purposes on May 20, 2024. This national immunization program evaluation survey aimed to estimate the performance of the immunization program. Various maternal and child health-related characteristics were collected. Geographic location data was gathered at the household level, but for analysis purposes, we computed the centroid latitude and longitude for each enumeration area or cluster. Data on zero-dose status was extracted for children aged 12–23 months.

Sampling and sample size

A two-stage stratified cluster sampling technique was employed using the 2007 Population and Housing Census as a sampling frame. Stratification was done by separating each region into urban and rural areas. Sampling frames were prepared for each region and city administrations by the Ethiopian Statistical Services (ESS). The number of EAs required per region and city administration was determined based on the size within the stratum (study regions) and proportion of the Ethiopia population living in urban and rural areas (21.4% urban and 78.6% rural). In the first stage, 468 EAs (100 from urban and 368 from rural areas) were chosen with probability sampling proportional to the size of the EAs, with independent selection in each sampling stratum. In the second stage, on average, 30 households were systematically selected. After weighting, the analysis included a total of 6,212 children aged 12-23 months from 463 enumeration areas (EAs), comprising 99 EAs from urban settings and 364 EAs from rural areas. This inclusion was necessary because five of the EAs lacked data for children aged 12-23 months.

Measurements

The outcome variable zero-dose was assessed for children born within 1-2 years prior to the survey. Zero-dose was defined as children aged 12-23 months who did not receive the first dose of the DPT containing vaccine (5, 43). Respondents who received the first dose of DPT containing vaccine were categorized as "Yes" or else as "No." The explanatory variables such as wealth index, region, religion, residency, marital status, mother/caregivers' educational status, occupation of mothers'/caregivers', birth order, ANC follow up, history of maternal tetanus diphtheria (Td) vaccine, distance to facility, mother/care givers perceived benefits on immunization, postnatal care (PNC) and place of delivery were included in the analysis.

Data analysis

The collected dataset was cleaned and preprocessed to ensuring compatibility with geographic analysis. All variables included in this analysis were weighted to handle the effect of selection, and non-response bias. Descriptive statistics like frequency, and percentage were used to describe participants' socio-demographic characteristics and zero-dose prevalence.

The outcome variable of interest was whether a child aged 12-23 months received the first dose of the pentavalent vaccine or not. The predictor variables included in this study were chosen based on established correlations and predictive factors found in existing literature, along with their biological relevance. Wealth index was computed as a composite index of living standard(6). It was developed based on ownership of valuable assets and livestock, size of land for agriculture and housing purposes, and materials used for house construction.

The spatial dependency of the outcome was assessed at the cluster level using the global Moran's Index. This spatial autocorrelation analysis calculated the Moran's Index, z-score, and associated p-values for both observed and expected index values, taking into account the number of features and their variance.

A positive Moran's Index indicates a tendency toward clustering, whereas a negative Moran's Index suggests a tendency toward dispersion and value closer to 0 it implicates a random pattern(44). Anselin Local Moran's I was used to identify the presence of local level cluster locations of zero-dose children and it measures whether there were positively correlated (high-high and low-low) clusters or negatively correlated (high-low and low-high) clusters which are called outliers (45, 46).

In addition, an optimized hot spot analysis technique was employed to pinpoint areas with statistically significant clusters of zero-dose children, both high (hot spots) and low (cold spots). The Optimized Hot Spot Analysis, which builds upon the traditional Hot Spot Analysis (Getis-Ord Gi* statistic), focuses on identifying spatial clustering by analyzing the distribution of features alongside their neighboring counterparts. Furthermore, it incorporates adjustments for multiple testing and spatial dependency through the False Discovery Rate (FDR) correction method (47).

The spatial scan statistical method identified clusters of zero-dose children using Kuldorff's SaTScan 9.7 software. It analyzed unvaccinated children as cases and vaccinated ones as controls under the Bernoulli model. The maximum scanning window was based on a percentage of the at-risk population, ensuring no small or large clusters were overlooked. Clusters were identified using p-values and likelihood ratio tests. The cluster demonstrating the highest likelihood ratio was designated as the most likely cluster (48).

Also, spatial interpolation technique was applied to predict zero-dose at unknown (non-sampled) locations using values at the measured (sampled) locations. The Kriging interpolation method was chosen because it is an optimal interpolator that minimizes both the mean error (ME) and the root mean square error (RMSE) (49).

To examine the factors explaining zero-dose children, we ran three different regression models. The conventional Ordinary Least Square (OLS) was used as baseline model with classical assumptions of

linear regression such as multicollinearity (VIF > 10 (50, 51). Koenker statistics was significance (p-value of 0.05), indicating the non-stationarity which means the associations between the dependent and independent variables varies across locations. Ordinary Least Squares (OLS) models often overlook the spatial autocorrelation that frequently exists in spatial data, which limits their effectiveness in explaining spatial phenomena. To overcome this limitation, Spatial Regression Models, such as Spatial Lag Models (SLM) and Spatial Error Models (SEM), was used to incorporate spatial autocorrelation, either within the dependent variables (SLM) or in the residuals (SEM). We employed the same set of explanatory variables to run spatial regression models using GeoDa version 1.20 (52, 53). After comparing the R-squared and AIC values, we determined that Spatial Error Models were more suitable than Spatial Lag Models for the final comparison between OLS and spatial regression models.

Finally, Geographic weighted regression (GWR) was employed to identify and quantify the spatial correlates of an independent variable based on the spatial proximity or distance among observations. GWR is particularly useful for addressing spatial non-stationarity, which refers to the presence of different relationships at various locations in space (54). To evaluate the models, the adjusted R-squared and corrected Akaike Information Criteria (AICc) were used to compare the Ordinary Least Squares (OLS) global model with the GWR local model. The model that best fit the data was determined by the lowest AICc value and the highest adjusted R-squared value (55).

Ethics approval and consent to participants

The protocol of the study was reviewed and approved by the Institutional Ethical Review Board of University of Gondar (Reference №: CMHSSH-UOG IRERC/3/7/2024). We used de-identified data (summary data without individuals' identity) to ensure confidentiality. Written informed consent was obtained from each mothers/ caregiver during the survey. We followed the international standard of strengthening the reporting of cross-sectional studies in epidemiology.

Results

A total of 6,212 of children aged 12-23 months were included in the analysis. The overall prevalence of zero-dose in Ethiopia was 24.8% (95%CI: 23.7–25.8%). There were regional variations in the prevalence of zero-dose children. The higher prevalence was observed in Somali (40.7%), Afar (40%) and followed by Oromia (27.8%) and the lowest observed in Addis Ababa (0.9%) and Dire Dawa (4.9 %) (**Error! Reference source not found.**). High proportion of zero-dose children were found from the rural residents (27.2%) and not educated mother/care givers (30.5%). Majority (68.3%) of children from mother who had no ANC visit didn't receive first dose of pentavalent vaccine (Table 1).

Table 1:Prevalence of Zero-dose stratified by socio-demographic and economic characteristics of mothers/caregivers of children aged 12–23 months in Ethiopia, 2023 (n=6,212)

**Variables**	**Zero-dose children**	
	**Yes, number (%)**	**No, number (%)**
**Place of residency**		
Rural	1,286(27.2)	3,439(72.8)
Urban	251(16.9)	1,236(83.1)
**Religion**		
Orthodox	512(22)	1830(78)
Muslim	800(30.5)	1,822(69.5)
Protestant	208(17.3)	998(82.7)
Others*	17(40.7)	25(59.3)
**Educational status**		
No education	925(31.2)	2,041(68.8)
Primary	401(21.0)	1,508(79.0)
Secondary	151(17.0)	737(83.0)
College and above	61(13.6)	388(86.4)
**Wealth status**		
Poor	499(26.4)	1394(73.6)
Middle	541(25.3)	1,600(74.7)
Richer	496(22.8)	1,681(77.2)
**Marital status**		
Married and living together	1,449(24.8)	4,403(75.2)
Married but not living together	35(23.4))	116(76.4)
Not in marital union	53(25.4)	157(74.6)
**Birth order**		
1st	409(18.2)	1835(81.8)
2nd	460(22.5)	1,586(77.5)
3rd	289(32.2)	610(67.8)
4th and above	379(37.1)	644(62.9)

**Parity**		
Primipara	308(23.1)	1,028(76.9)
Multipara (2–4)	589(20.1)	2,334(79.9)
Grand multipara (5^{+})	480(33.8)	940(66.2)
**Perceived distance to vaccination site**		
Big problem	490(13.0)	3273(87.0)
No problem	1047(42.8)	1,0402(57.2)
**Perceived benefit on immunization**		
Poor	781(74.0)	275(26.0)
Good	578(12.0)	4221(88.0)
**Antenatal care visits**		
Yes	777(16.2)	4023(83.8)
No	600(68.3)	279(31.2)
**Place of delivery**		
Home	723(43.8)	942(56.2)
Health facility	644(16.1)	3360(83.9)
**Postnatal care**		
Yes	440(17.8)	2030(82.2)
No	937(29.2)	2771(70.8)

Others*: catholic, traditional, others

Figure 1: The prevalence of Zero-dose children aged 12-23 months in Ethiopia, 2023 (n=6212)

Geographic patterns of zero-dose children

In the spatial autocorrelation analysis, the Global Moran's I value (I = 0.19), z-score of 3.67 and a p-value=0.0002 indicated that the prevalence of zero-dose children at cluster level were close to each other. There was less than 1% (99% degree of confidence) likelihood that this clustering could be by chance (Figure 2).

Figure 2:Spatial autocorrelations of Zero-dose children aged 12-23 months in Ethiopia, 2023

The Local Moran's I analysis identified clusters and outliers among children who received no first dose of DTP containing vaccine. The results highlighted both high-high and low-low clusters, indicating areas with significant excess of zero-dose children. Notably, a substantial number of unvaccinated children were high in various regions, including much of Somali, northern and southern Afar, Eastern Amhara, and southern Oromia. Conversely, outliers(high-low), characterized by areas with a large number of zero-dose children surrounded by areas with fewer unvaccinated children, were primarily found in southern Amhara,

central and southwestern Oromia, northeastern Somali, Sidama, and the Southern Nations, Nationalities, and Peoples' Region (SNNP). In contrast, low-high outliers denote clusters where there is a low percentage of zero-dose children encircled by areas with a higher percentage of zero-dose children are detected southwest Somali and Oromia, South-east Afar, northeast Benishangul Gumuz and Eastern Amhara (Figure 3).

Figure 3: Spatial Cluster and outlier for Zero-dose prevalence among12-23 months children in Ethiopia, 2023

The hotspot analysis detects high proportion of zero-dose children (hot spots) and clusters with a low proportion of zero-dose (cold spot). The higher proportion of zero-dose children (red dots) were prevalent in most parts of Somali, north and southwest Afar, Eastern part of Amhara, and southwest Oromia. Conversely, low proportion of zero-dose (blue dots) was detected in Centeral and northern Oromia, southern Amhara, Addis Ababa, Southern Afara, and northern parts of SNNP (Figure 4).

Figure 4: Hotspot areas for Zero-dose children aged 12-23 months in Ethiopia, 2023

The spatial scan statistics analysis identified six statistically significant clusters for zero-dose children. The most likely clusters, also called the primary cluster with at total enumeration area of 6 was found in the southern Oromia region. The primary cluster was pinpointed with Log-Likelihood Ratio of 135.7 spanning an area with a radius of 101.63km (Table 2). In addition, five most likely secondary clusters circled with a pink, purple, light blue and green color were found in the easter, southern, southwest and southeast parts of the country (figure 5).

Table 2: Significant clusters for zero-dose children aged 12-23 months in Ethiopia, 2023

**Cluster**	**N**	**Population**	**Cases**	**RR**	**LLR**	**P-value**	**Radius**
Primary	6	214	165	3.39	135.7	<0.0001	101.63 km
Secondary 1	2	51	41	3.33	35.39	<0.0001	15.97 km
Secondary 2	23	142	79	2.33	31.85	<0.0001	119.14 km
Secondary 3	3	21	15	2.92	10.2	<0.0098	104.19 km
Secondary 4	121	20	53	1.72	9.1	<0.029	117.53 km

Note: N = number of clusters (EA), RR = relative risk, LLR = log-likelihood ratio

Figure 5:Primary and secondary clusters of zero-dose children aged 12-23 months in Ethiopia, 2023

Predicted prevalence of zero-dose children

The predicted map evident that most parts of Afar and Somali, southern and southeastern Oromia, as well as eastern Amhara had a relatively high zero-dose prevalence. On the other hand, Addis Ababa, Dire Dawa, the Kefa and Konta special zones in southwestern Ethiopia, the Sidama zone in the Sidama region, Kamashi zone of Benishangul Gumuz region and certain parts of Oromia were identified as low-risk regions for zero-dose children (Figure 6).

Figure 6: Predicted prevalence of zero-dose children aged 12-23 months using Kriging interpolation in Ethiopia, 2023

The color pattern shows she spatial difference in the prevalence of zero-dose. The deeper red color indicates a higher prevalence of zero-dose children, the lighter indicates the lower prevalence.

Factors associated with geographic variabilities of zero-dose children

The OLS analysis identified four variables, no ANC utilization, poor perception on benefit of immunization, no maternal TT/Td vaccination, and distance to facility as a big problem were found associated with zero-dose children. There was no indication of multicollinearity detected among the selected independent variables evidenced by a variance inflation factor (VIF) ranged from 1.28 - 2.98. The OLS model (adjusted R^2 =0.646) explained 64.6% of the variation in zero-dose children by the four explanatory variables (Table 3).

The Koenker statistics in the model is statistically significant indicating that the regression model is inconsistent across the study area (with the change in geographic location the relationship of variables will also change) implies there is the non-stationarity of the variance. The results obtained from the OLS method prompt us to utilize spatial regression analysis alongside the GWR model, which we deem more suitable for estimating the model parameters (Table 3).

Table 3:Ordinary least square egression (OLS) results summary

Variable	Coefficient	Robust t-statistics	Robust probability	VIF
Intercept	0.032	4.211872	<0.0001	--------
No ANC utilization	0.303	4.505514	<0.0001	2.98
poor perception on benefit of immunization	0.379	7.471412	<0.0001	1.75
no maternal TT vaccination	0.151	2.206580	0.028	2.65
and distance to facility as a big problem	0.113	3.796852	<0.0001	1.28
**Ordinary Least Square Regression (OLS) diagnostic**				
Number of observations	463	**P-Value**		
Joint F-statistics	212	<0.0001		
Joint Wald statistics	682	<0.0001		
Koenker (BP) statistics	58	<0.0001		
Jarque- Bera	48.4	<0.0001		
Moran's I	0.172	0.0012		
**Model performance**				
Adjusted R-Squared		0.646		
Akaike's Information Criterion (AICc)		-542.36		

The Spatial Error (SER) model demonstrated superior performance with a more robust overall fit, achieving an adjusted R^2 of 66.5%, which surpasses the Ordinary Least Squares (OLS) model's 64.9% and the Spatial Lag model's 65%. The AICc (-558) and BIC (537.9) of the SEM is lower than the OLS and SLM. The local GWR model with a highest Adjusted R2 (0.77) and a lowest AICc(-628) better performed than the OLS, SLM and SER. The GWR model explained 77% of the variation of zero-dose children prevalence, significantly outperforming than baseline OLS model. In addition, the spatial autocorrelation observed from the GWR output (Moran's I=0.01, P-value=0.179) suggests that the GWR model is fitting the data well, as the residuals do not exhibit significant spatial patterns (Table 4). The mean coefficient of all explanatory variables were positive. Indicating a positive association between zero-dose children and the four explanatory variables (no ANC utilization, poor perception on immunization, No maternal Td vaccine and perceived distance to facility as far).

Table 4: Model comparisons among the ordinary least square, spatial error, SEM-Spatial error and geographical weighted regression models

Predictors	Zero-dose children			
	**OLS** (coef, p-value)	**SLM** (coef, p-value)	**SEM** (coef, p-value)	**GWR**
No ANC utilization	0.303(0.000)	0.304(0.000)	0.301(0.000)	
poor perception on immunization	0.379(0.001)	0.15(0.001)	0.39(0.000)	
no maternal TT vaccination	0.151(0.000)	0.37(0.000)	0.16(0.000)	
Distance to facility as a big problem	0.113(0.000)	0.11(0.000)	0.12(0.000)	
LL	277.5	278.7	284.3	
Moran's I (value, P-value)-Residuals	0.172(0.001)	0.09(0.79)	0.002(0.48)	0.01(0.18)
BIC	-524.3	-520.6	-537.9	
AIC	-542.4	-545.4	-558	-628
Adjusted R^2	0.646	0.65	0.67	0.77

OLS-Ordinary Least Square Model, SLM-Spatial Lag Model, SEM-Spatial Error Model, GWR-Geographic Weighted Regression

Figure 7 (a, b, c and d) display the GWR coefficients for each explanatory variable. These coefficients reveal that the relationship between independent variables and the prevalence of zero-dose children varies significantly by location. Areas marked with red, orange, and yellow dotted points demonstrate a considerable impact of these variables on zero-dose children. The risk of a child being unvaccinated increases with the number of mothers who do not utilize antenatal care (ANC) varies across regions. The higher coefficient found in Addis Ababa, Oromia, Somali, Sidama, Benishangul Gumuz, SNNP, Dire Dawa, Harari, and Amhara (Figure 7a).

Perception on benefits of immunization was strongly linked with zero-dose children and causes higher in most parts of the country mainly eastern and southeastern Amhara, the majority of Oromia, Sidama, Sidam, Somali, SNNP and Afar (Figure 7b).

The coefficient of mother/care givers who perceived distance to immunization site is far reveals a positive correlation predominantly observed in the Afar, Somali, Benishangul Gumuz, Oromia, SNNP and some parts of Amhara (Figure 7c). Similarly, a notable association of maternal TT/Td vaccination and prevalence zero-dose children was identified across the country and the strong relation was observed in most parts of Oromia, Somali, SNNP, and Afar (Figure 7d).

Figure 7: The spatial mapping of local regression coefficients of explanatory variables on Zero-dose children aged 12-23 months in Ethiopia, 2023

Discussion

Using the data from the most recent national immunization survey of Ethiopia, this study aimed to assess the geographic inequities of zero-dose children and its determinants among children aged 12-23 months. In this study, we found that one in four children didn't take the first dose of DPT containing vaccines. This finding slightly surpasses the joint estimates of immunization Coverage for 2023 as reported by the World Health Organization (WHO) and the United Nations International Children's Emergency Fund (UNICEF) (56). This is also higher than the mini EDHs report of 2019(6). The result suggests that the country has lagged behind the target in reducing the number of the number of zero-dose children. This highlights the urgent need for a proactive and comprehensive strategy to address the significant issue of unvaccinated children.

The spatial analysis has greatly enhanced our understanding of the geographic distribution of zero-dose children (ages 12-23 months) using data from recent national immunization surveys and advanced geo-statistical techniques. This analysis offers a clear picture of unvaccinated children coverage disparities at sub-national levels, helping to prioritize areas that requires more attention.

The study revealed spatial dependence, disparities, and local determinants affecting zero-dose children across Ethiopia. It identified both high (hotspots) and low (cold spots) of zero-dose prevalence throughout the country. Notably, there are sharp subnational disparities, many hot spots located in the pastoralist

regions of the country, particularly in in Afar and Somali., while cold spots are found in the central and northern Oromia, southern Amhara, Addis Ababa, and the northern parts of Southern Nations, Nationalities, and Peoples regions. These findings underscore the regional inequality in zero-dose prevalence.

This finding aligns with previous studies, which point to regional disparities in zero-dose prevalence and immunization coverage indicating the high burden of the unvaccinated children concentrated in the pastoralist regions of the country mainly in Afar and Somali(57). Similarly, other studies on geographical disparities in incomplete immunization have identified that most hotspot regions are located within the Somali and Afar areas(58, 59). These regions face significant challenges related to the accessibility and availability of health services, compounded by persistent quality gaps in healthcare delivery (60, 61).

This stark geographic disparity not only highlights the inequities but also raises important questions about the underlying factors contributing to this variation. Such inequalities can be attributed to variations in socio-economic factors and healthcare related factors, including place of residence, maternal health utilization, and the accessibility of health facilities (1, 13, 15, 16).

This study also sought to identify the predictor variables linked to zero-dose children. Antenatal care (ANC) service utilization, perceptions on immunization benefits, maternal tetanus toxoid (TT) vaccination, and the distance to immunization sites were significant factors contributing to the prevalence of zero-dose children in Ethiopia. The findings of this study indicated that lack of antenatal care (ANC) utilization significantly correlated with a high prevalence of zero-dose children. This is in agreement with previous study examined the prevalence, spatial variation, and determinants of zero-dose children (57). Children born to mothers without ANC follow-up are more likely to be zero-dose compared to those whose mothers did seek ANC. This coorelation can be attributed to the fact that ANC visits enables

mothers to access integrated health service, more likely to receive information on immunization schedules, build trust on the health system and improve adherence to health services(62-64). The coefficients from your GWR model indicate that the influence of lack of ANC utilization on zero-dose children varies geographically, stronger correlation observed in Oromia, the SNNP, Addis Ababa and Afar regions. This highlights the importance of localized strategies to address barriers for child immunization.

This study also revealed that maternal TT/Td vaccination significantly influence the prevalence of zero-dose children, consistent with previous study (64, 65). One possible explanation is that maternal vaccination increases access to healthcare, fostering a trusting relationship between mothers and healthcare providers. This trust can enhance communication regarding immunization schedules and the importance of vaccinating children. Notably, the highest correlations for TT/Td vaccination were observed in the Oromia, Somali, Afar, and SNNP regions, indicating that the effects of maternal vaccination vary across different areas of the country.

Another important finding was that mothers' and caregivers' perceptions on vaccinations was closely linked to the prevalence of zero-dose children. Previous studies have consistently shown that individuals who has negative perceptions about the benefits of immunization are less likely to pursue vaccinations for their children(65, 66). This relationship underscores the critical role that beliefs and attitudes play in healthcare decision-making. This tendency can lead to significant gaps in immunization coverage, ultimately contributing to higher rates of vaccine preventable diseases. The analysis revealed that the highest coefficient, indicating a strong negative impact of poor perceptions regarding the benefits of immunization, was found in regions such as Somali, eastern and southern Amhara, Sidama, Afar, and some parts of Oromia. These areas may face unique challenges, including cultural beliefs, misinformation, and limited access to accurate health information, which can intensify negative attitudes towards vaccinations. Addressing perception as a factor for zero-dose children may require a multifaceted and

local specific tailored approach that combines education, and community engagement aiming to increase the awareness of mother/care givers on the benefits of immunization.

Additionally, the study found that distance to immunizations site significantly influences the prevalence of zero-dose. This finding is consistent with previous studies evidenced that geographical barriers that affect accessibility and the distance to healthcare facilities were the predominant barriers for immunization coverage(67, 68). The high coefficients were observed in Somali, Afar, Benishangul and Some parts of Oromia region. The Somali and Afar regions have been highlighted as areas in which accessing and utilizing health services pose significant challenge(60, 61). This finding underscores that disparities in the health services remain a persistent issue across various regions in Ethiopia. Design and implement context specific appropriate strategy for this pastoralist regions are needed to identify and reach zero-dose children. The existing vaccination strategies should be strengthened and assess which better fits for the areas.

This study had several strengths worth mentioning. We used a recent national level survey data involving representative samples from 463 enumeration areas in Ethiopia and thus the result obtained from this analysis could be generalizable across the country. The study produced the picture of the most recent zero-dose situation in Ethiopia. Additionally, this study assessed the spatial heterogeneity of zero-dose prevalence using advanced geo-statistics to identify the most risk areas and the risk factors considering the spatial distribution and dependence of zero-dose prevalence. The spatial heterogeneity of the model parameters has been accounted using the spatial regression analysis and GWR. At the same time, this study identified the risk factors of zero-dose which may help policy makers and planners to design tailored interventions to identify and reach the unvaccinated children in the geographically high where the zero-dose is eventually high compared to the other parts of the country

This study was subject to some limitations. Firstly, though we used a national level data and we predicted the prevalence of zero-dose for the unsampled sites, we didn’t included data from Tigray region which is one of the administrative regions of the country data due to security issues. Secondly, distance was assessed through subjective measures. By asking participants about distance as far or near to immunization sites. Objective measure of distance, such actual distance from home to immunization site or actual travel times(reference), may provide a more accurate representation of accessibility and its impact on zero-dose prevalence. Lastly, the perceptions of mothers and caregivers regarding the benefits of immunization were measured using binary yes/no questions. However, this approach may oversimplify their views and could result in failing to capture a more subtle understanding of their attitudes towards immunization.

Conclusion

Despite significant efforts in Ethiopia's immunization program, the proportion of unvaccinated children remains unacceptably high, with one-fourth of children still not immunized. Our analysis indicated notable regional disparities in the prevalence of zero-dose children. The results also highlight associations between zero-dose prevalence and factors such as ANC utilization, perceptions of immunization benefits, TT/Td vaccination, and distance to immunization sites. These associations vary across regions, underscoring the need for tailored interventions to identify and reach zero-dose children. This approach can help fulfill the national and global immunization agenda of leaving no one behind by ensuring equitable access to vaccinations. Additionally, regional governments should strengthen maternal health services through various health promotion activities, which can also raise awareness about the benefits of immunization.

Acknowledgment

The authors would like to thank the University of Gondar for the unwavering support. Additionally, we extend our sincere thanks to the Ministry of Health of Ethiopia for granting us access to the data that was critical for our study.

References

1. WHO. Immunization agenda 2030: a global strategy to leave no one behind World Health Organization, Geneva, Switzerland

2020 [1-29]. Available from: https://www.who.int/publications/m/item/immunization-agenda-2030-a-global-strategy-to-leave-no-one-behind.

2. Carter A, Msemburi W, Sim SY, Gaythorpe KA, Lambach P, Lindstrand A, et al. Modeling the impact of vaccination for the immunization Agenda 2030: Deaths averted due to vaccination against 14 pathogens in 194 countries from 2021 to 2030. Vaccine. 2024;42:S28-S37.

3. WHO. Immunization Coverage 2024 [updated July 15/2024. Available from: https://www.who.int/news-room/fact-sheets/detail/immunization-coverage.

4. Galles NC, Liu PY, Updike RL, Fullman N, Nguyen J, Rolfe S, et al. Measuring routine childhood vaccination coverage in 204 countries and territories, 1980–2019: a systematic analysis for the Global Burden of Disease Study 2020, Release 1. The Lancet. 2021;398(10299):503-21.

5. GAVI. Zero Dose children and missed communities 2021 [Available from: https://www.gavi.org/our-alliance/strategy/phase-5-2021-2025/equity-goal/zero-dose-children-missed-communities.

6. Ethiopian public health Institute (EPHI)[Ethiopia] and ICF. 2021. Ethiopia Mini Demographic and Health Survey 2019: Final Report. Rockville, Maryland, USA: EPHI and ICF.

7. Jones CE. Routine Vaccination Coverage—Worldwide, 2023. MMWR Morbidity and Mortality Weekly Report. 2024;73.

8. Resolution GA. Transforming our world: the 2030 Agenda for Sustainable Development. UN Doc A/RES/70/1 (September 25, 2015). 2015.

9. Bosch-Capblanch X, Banerjee K, Burton A. Unvaccinated children in years of increasing coverage: how many and who are they? Evidence from 96 low-and middle-income countries. Tropical Medicine & International Health. 2012;17(6):697-710.

10. Muhoza P, Danovaro-Holliday MC, Diallo MS, Murphy P, Sodha SV, Requejo JH, et al. Routine vaccination coverage—worldwide, 2020. Morbidity and Mortality Weekly Report. 2021;70(43):1495.

11. WHO. WHO/UNICEF estimates of national immunization coverage. 2019. 2019.

12. Hogan D, Gupta A. Why Reaching Zero-Dose Children Holds the Key to Achieving the Sustainable Development Goals. Vaccines. 2023;11(4):781.

13. Wigley A, Lorin J, Hogan D, Utazi CE, Hagedorn B, Dansereau E, et al. Estimates of the number and distribution of zero-dose and under-immunised children across remote-rural, urban, and conflict-affected settings in low and middle-income countries. PLOS Global Public Health. 2022;2(10):e0001126.

14. Lee BX, Kjaerulf F, Turner S, Cohen L, Donnelly PD, Muggah R, et al. Transforming our world: implementing the 2030 agenda through sustainable development goal indicators. Journal of public health policy. 2016;37:13-31.

15. Farrenkopf BA, Zhou X, Shet A, Olayinka F, Carr K, Patenaude B, et al. Understanding household-level risk factors for zero dose immunization in 82 low-and middle-income countries. Plos one. 2023;18(12):e0287459.

16. Utazi CE, Pannell O, Aheto JM, Wigley A, Tejedor-Garavito N, Wunderlich J, et al. Assessing the characteristics of un-and under-vaccinated children in low-and middle-income countries: A multi-level cross-sectional study. PLOS Global Public Health. 2022;2(4):e0000244.

17. Gavi TVA. Gavi Strategy 5.0, 2021–2025 2020 [updated April 22, 2024. Available from: https://www.gavi.org/our-alliance/strategy/phase-5-2021-2025.

18. Dadari I, Belt RV, Iyengar A, Ray A, Hossain I, Ali D, et al. Achieving the IA2030 coverage and equity goals through a renewed focus on urban immunization. Vaccines. 2023;11(4):809.

19. Boyce T, Gudorf A, de Kat C, Muscat M, Butler R, Habersaat KB. Towards equity in immunisation. Eurosurveillance. 2019;24(2):1800204.

20. Glasser JW, Feng Z, Omer SB, Smith PJ, Rodewald LE. The effect of heterogeneity in uptake of the measles, mumps, and rubella vaccine on the potential for outbreaks of measles: a modelling study. The Lancet Infectious Diseases. 2016;16(5):599-605.
21. Marek L, Hobbs M, McCarthy J, Wiki J, Tomintz M, Campbell M, et al. Investigating spatial variation and change (2006–2017) in childhood immunisation coverage in New Zealand. Social Science & Medicine. 2020;264:113292.
22. Brownwright TK, Dodson ZM, van Panhuis WG. Spatial clustering of measles vaccination coverage among children in sub-Saharan Africa. BMC public health. 2017;17(1):1-7.
23. Utazi CE, Wagai J, Pannell O, Cutts FT, Rhoda DA, Ferrari MJ, et al. Geospatial variation in measles vaccine coverage through routine and campaign strategies in Nigeria: Analysis of recent household surveys. Vaccine. 2020;38(14):3062-71.
24. Dimitrova A, Carrasco-Escobar G, Richardson R, Benmarhnia T. Essential childhood immunization in 43 low-and middle-income countries: Analysis of spatial trends and socioeconomic inequalities in vaccine coverage. PLoS Medicine. 2023;20(1):e1004166.
25. Atalell KA, Liyew AM, Alene KA. Spatial distribution of rotavirus immunization coverage in Ethiopia: a geospatial analysis using the Bayesian approach. BMC Infectious Diseases. 2022;22(1):830.
26. Kamal A, Waseem A, Siddiqa M, Ijaz M, Shakeel A, Iftikhar S. Contextual factors influencing incomplete immunization and investigation of its geospatial heterogeneity in Pakistan: a cross-sectional study based on PDHS (2017–18). BMC Public Health. 2023;23(1):1620.
27. Siddique M, Iftikhar S, Dharma VK, Shah MT, Siddiqi DA, Malik AA, et al. Using geographic information system to track children and optimize immunization coverage and equity in Karachi, Pakistan. Vaccine. 2023;41(18):2922-31.
28. Ikilezi G, Augusto OJ, Sbarra A, Sherr K, Dieleman JL, Lim SS. Determinants of geographical inequalities for DTP3 vaccine coverage in sub-Saharan Africa. Vaccine. 2020;38(18):3447-54.
29. WHO. Immunization coverage. 2023.
30. Mosser JF, Gagne-Maynard W, Rao PC, Osgood-Zimmerman A, Fullman N, Graetz N, et al. Mapping diphtheria-pertussis-tetanus vaccine coverage in Africa, 2000–2016: a spatial and temporal modelling study. The Lancet. 2019;393(10183):1843-55.
31. Jiang Y, Fan F, Holly Zanoni I, Li Y. Global diffusion pattern and hot spot analysis of vaccine-preventable diseases. ISPRS Annals of the Photogrammetry, Remote Sensing and Spatial Information Sciences. 2017;4:167-73.
32. Moore DA, Carpenter TE. Spatial analytical methods and geographic information systems: use in health research and epidemiology. Epidemiologic reviews. 1999;21(2):143-61.
33. Atalell KA, Techane MA, Wubneh CA, Assimamaw NT, Belay GM, Tamir TT, et al. Spatiotemporal distributions of immunization coverage in Ethiopia from 2000 to 2019. Vaccine. 2022;40(10):1413-20.
34. Awol M, Alemu ZA, Moges NA, Jemal K. Geographical variations and associated factors of defaulting from immunization among children aged 12 to 23 months in Ethiopia: using spatial and multilevel analysis of 2016 Ethiopian Demographic and Health Survey. Environmental Health and Preventive Medicine. 2021;26(1):1-17.
35. Geremew TT, Gezie LD, Abejie AN. Geographical variation and associated factors of childhood measles vaccination in Ethiopia: a spatial and multilevel analysis. BMC public health. 2019;19:1-15.
36. Melaku MS, Nigatu AM, Mewosha WZ. Spatial distribution of incomplete immunization among under-five children in Ethiopia: evidence from 2005, 2011, and 2016 Ethiopian demographic and health survey data. BMC Public Health. 2020;20:1-22.
37. Teshale AB, Amare T. Exploring spatial variations and the individual and contextual factors of uptake of measles-containing second dose vaccine among children aged 24 to 35 months in Ethiopia. Plos one. 2023;18(1):e0280083.
38. Takahashi S, Metcalf CJE, Ferrari MJ, Tatem AJ, Lessler J. The geography of measles vaccination in the African Great Lakes region. Nature communications. 2017;8(1):15585.

39. Utazi CE, Thorley J, Alegana VA, Ferrari MJ, Takahashi S, Metcalf CJE, et al. Mapping vaccination coverage to explore the effects of delivery mechanisms and inform vaccination strategies. Nature communications. 2019;10(1):1633.
40. Utazi CE, Thorley J, Alegana VA, Ferrari MJ, Takahashi S, Metcalf CJE, et al. High resolution age-structured mapping of childhood vaccination coverage in low and middle income countries. Vaccine. 2018;36(12):1583-91.
41. Yourkavitch J, Burgert-Brucker C, Assaf S, Delgado S. Using geographical analysis to identify child health inequality in sub-Saharan Africa. PLoS One. 2018;13(8):e0201870.
42. Chopra M, Bhutta Z, Blanc DC, Checchi F, Gupta A, Lemango ET, et al. Addressing the persistent inequities in immunization coverage. Bulletin of the World Health Organization. 2020;98(2):146.
43. Wonodi C, Farrenkopf BA. Defining the Zero Dose Child: A Comparative Analysis of Two Approaches and Their Impact on Assessing the Zero Dose Burden and Vulnerability Profiles across 82 Low-and Middle-Income Countries. Vaccines. 2023;11(10):1543.
44. Getis A. Spatial autocorrelation. Handbook of applied spatial analysis: Software tools, methods and applications: Springer; 2009. p. 255-78.
45. Anselin L. Spatial econometrics: Methods and modelskluwer academic. Boston, MA. 1988.
46. Anselin L. Local indicators of spatial association—LISA. Geographical analysis. 1995;27(2):93-115.
47. Optimized Hot Spot Analysis (Spatial Statistics): ESRI; [Available from: https://pro.arcgis.com/en/pro-app/latest/tool-reference/spatial-statistics/optimized-hot-spot-analysis.htm.
48. Kulldorff M. A spatial scan statistic. Communications in Statistics-Theory and methods. 1997;26(6):1481-96.
49. Meng Q, Liu Z, Borders BE. Assessment of regression kriging for spatial interpolation–comparisons of seven GIS interpolation methods. Cartography and geographic information science. 2013;40(1):28-39.
50. Akinwande MO, Dikko HG, Samson A. Variance inflation factor: as a condition for the inclusion of suppressor variable (s) in regression analysis. Open journal of statistics. 2015;5(07):754.
51. Salmeron R, García C, García J. Variance inflation factor and condition number in multiple linear regression. Journal of statistical computation and simulation. 2018;88(12):2365-84.
52. Anselin L. GeoDa (Spatial Statistical Program). The Encyclopedia of Research Methods in Criminology and Criminal Justice. 2021;2:839-41.
53. Ward MD, Gleditsch KS. Spatial regression models: Sage Publications; 2018.
54. Wheeler DC. Geographically weighted regression. Handbook of regional science: Springer; 2021. p. 1895-921.
55. Akaike H. Information theory and an extension of the maximum likelihood principle. Selected papers of hirotugu akaike: Springer; 1998. p. 199-213.
56. WHO U. WHO/UNICEF estimates of national immunization coverage 2024 [Available from: https://www.who.int/news-room/questions-and-answers/item/who-unicef-estimates-of-national-immunization-coverage.
57. Tamir TT, Zegeye AF, Mekonen EG, Tekeba B, Ali MS, Gonete AT, et al. Prevalence, spatial variation and determinants of zero-dose children in Ethiopia: Spatial and multilevel analyses. Public Health. 2024;236:365-72.
58. Bantie B, Atnafu Gebeyehu N, Adella GA, Ambaw Kassie G, Mengstie MA, Abebe EC, et al. Mapping geographical inequalities of incomplete immunization in Ethiopia: a spatial with multilevel analysis. Frontiers in Public Health. 2024;12:1339539.
59. Ketsela A, Gebreyesus SH, Deressa W. Spatial distribution of under immunization among children 12–23 months old in Butajira HDSS, southern Ethiopia. BMC pediatrics. 2021;21(1):226.
60. Hierink F, Oladeji O, Robins A, Muñiz MF, Ayalew Y, Ray N. A geospatial analysis of accessibility and availability to implement the primary healthcare roadmap in Ethiopia. Communications Medicine. 2023;3(1):140.

61. Misganaw A, Naghavi M, Walker A, Mirkuzie AH, Giref AZ, Berheto TM, et al. Progress in health among regions of Ethiopia, 1990–2019: a subnational country analysis for the Global Burden of Disease Study 2019. The Lancet. 2022;399(10332):1322-35.
62. Dixit P, Dwivedi LK, Ram F. Strategies to improve child immunization via antenatal care visits in India: a propensity score matching analysis. PloS one. 2013;8(6):e66175.
63. Jiao B, Iversen I, Sato R, Pecenka C, Khan S, Baral R, et al. Association between achieving adequate antenatal care and health-seeking behaviors: A study of Demographic and Health Surveys in 47 low-and middle-income countries. Plos Medicine. 2024;21(7):e1004421.
64. Krishnamoorthy Y, Rehman T. Impact of antenatal care visits on childhood immunization: a propensity score-matched analysis using nationally representative survey. Family Practice. 2022;39(4):603-9.
65. Chambongo PE, Nguku P, Wasswa P, Semali I. Community vaccine perceptions and its role on vaccination uptake among children aged 12-23 months in the Ileje District, Tanzania: a cross section study. Pan African Medical Journal. 2016;23(1).
66. Puri YE, Murti B, Dermatoto A. Analysis of the effect of maternal perception on completeness of child immunization status with health belief model. Journal of Health Promotion and Behavior. 2016;1(3):211-22.
67. Girmay A, Dadi AF. Full immunization coverage and associated factors among children aged 12-23 months in a hard-to-reach areas of Ethiopia. International journal of pediatrics. 2019;2019.
68. Nour TY, Farah AM, Ali OM, Abate KH. Immunization coverage in Ethiopia among 12–23 month old children: systematic review and meta-analysis. BMC Public Health. 2020;20(1):1-12.

Figure 1

Click here to access/download;Figure;Figure 1 Prevalence_zero_dose.png

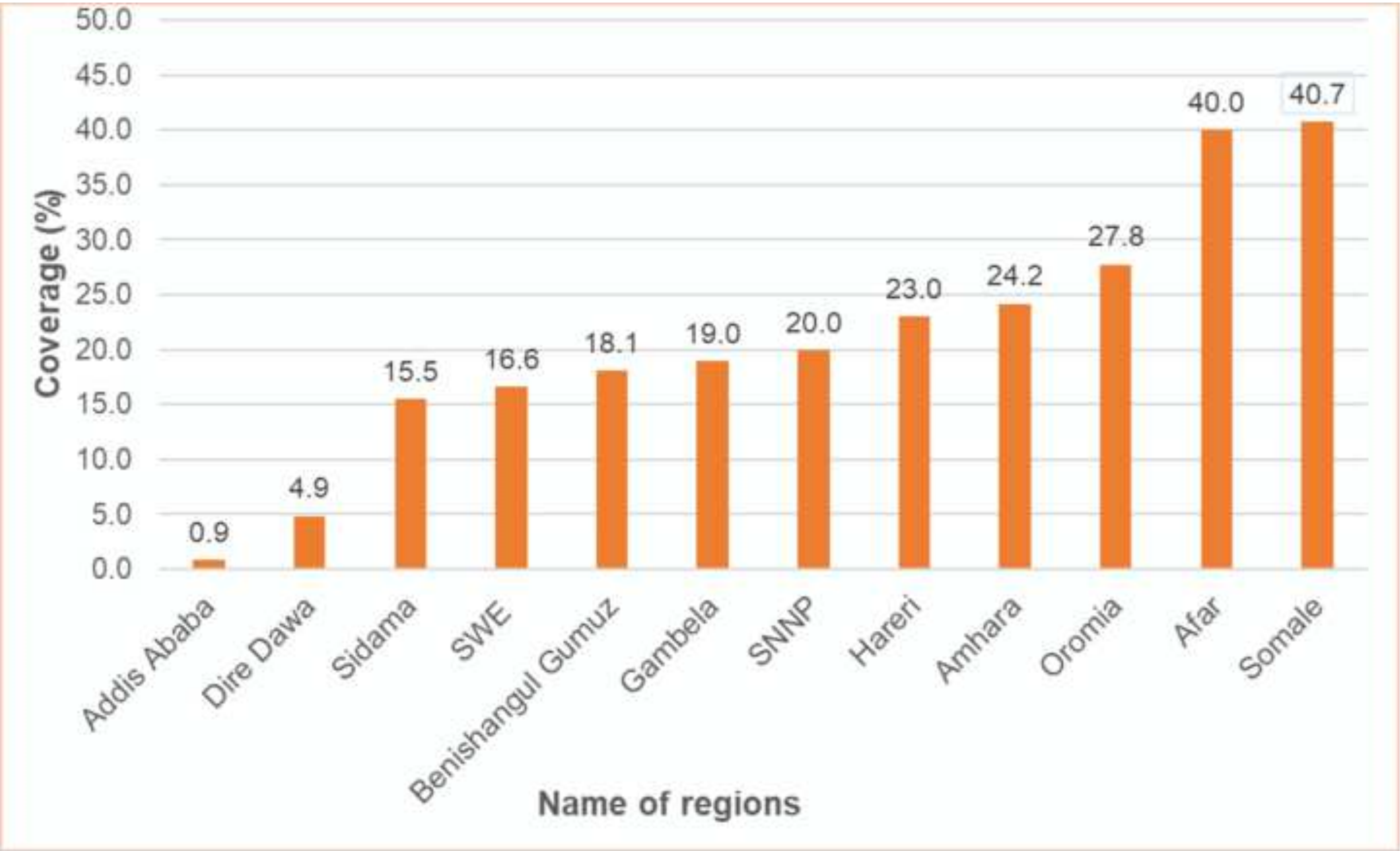

Figure 2

Click here to access/download;Figure;Figure2_Spatial autocolration.png

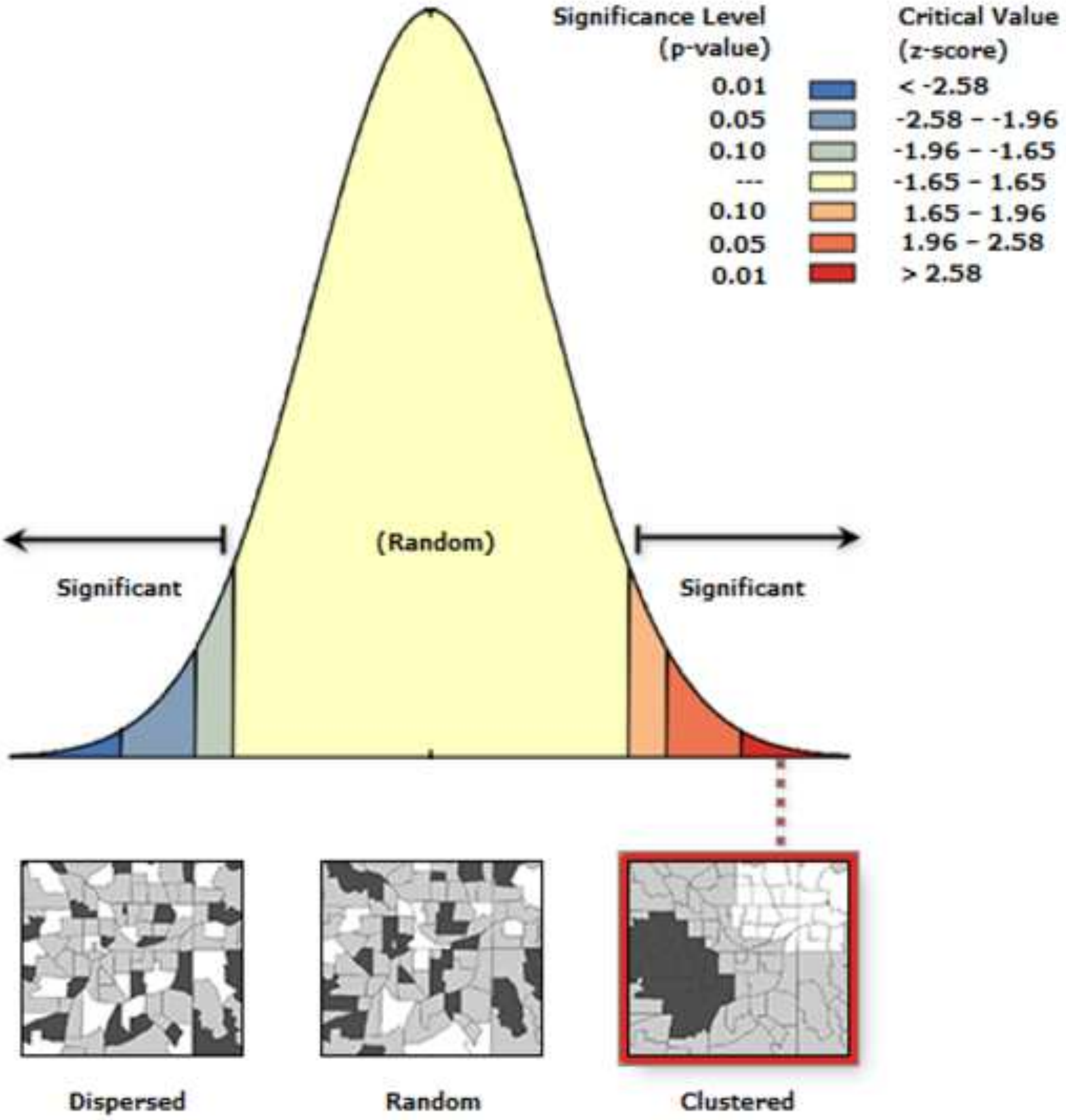

Figure 3

Click here to access/download;Figure;Figure 3_Cluster and outlier of zerodose.tif

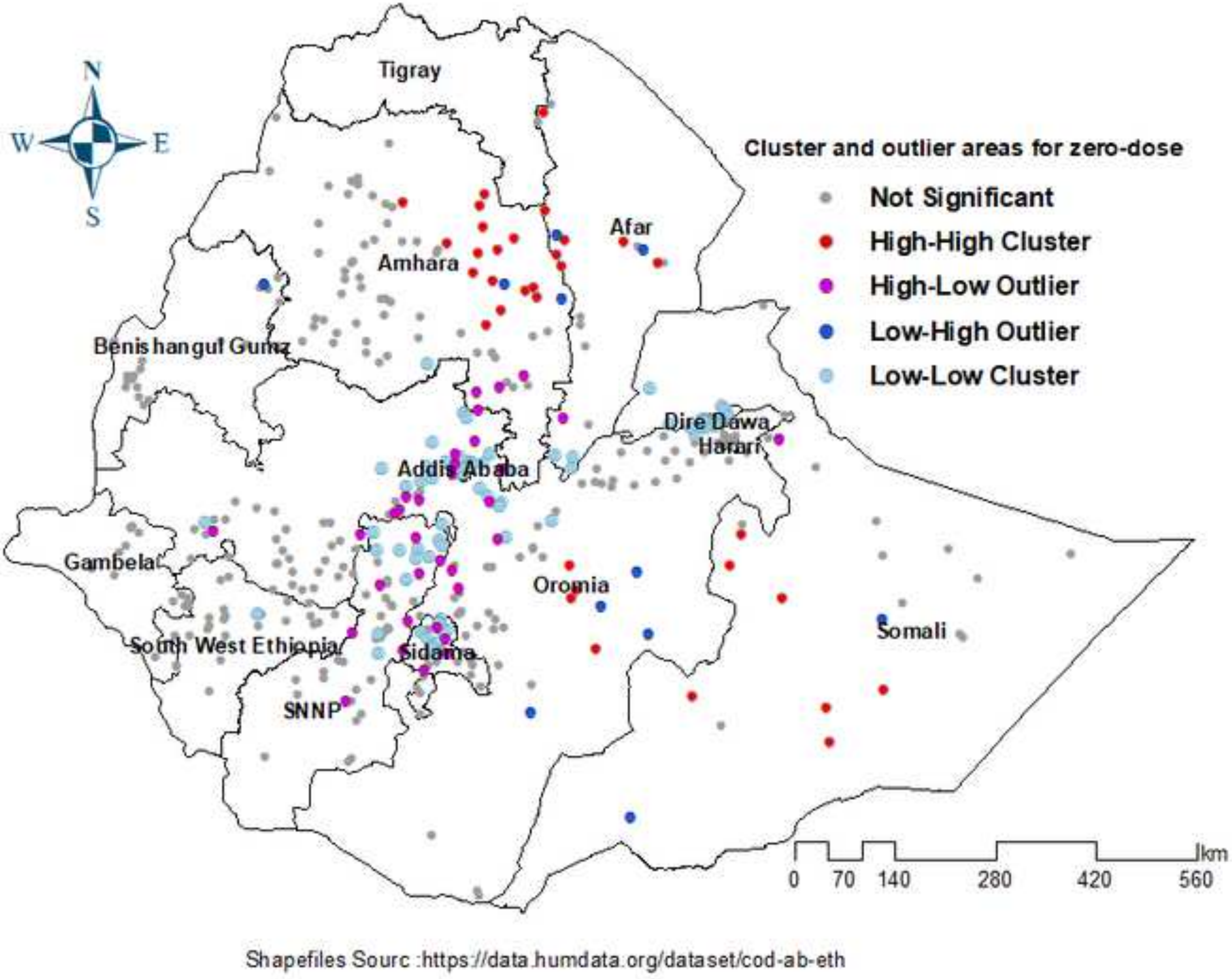

Figure 4

Click here to access/download;Figure;Figure 4_Hotspot analysis.png

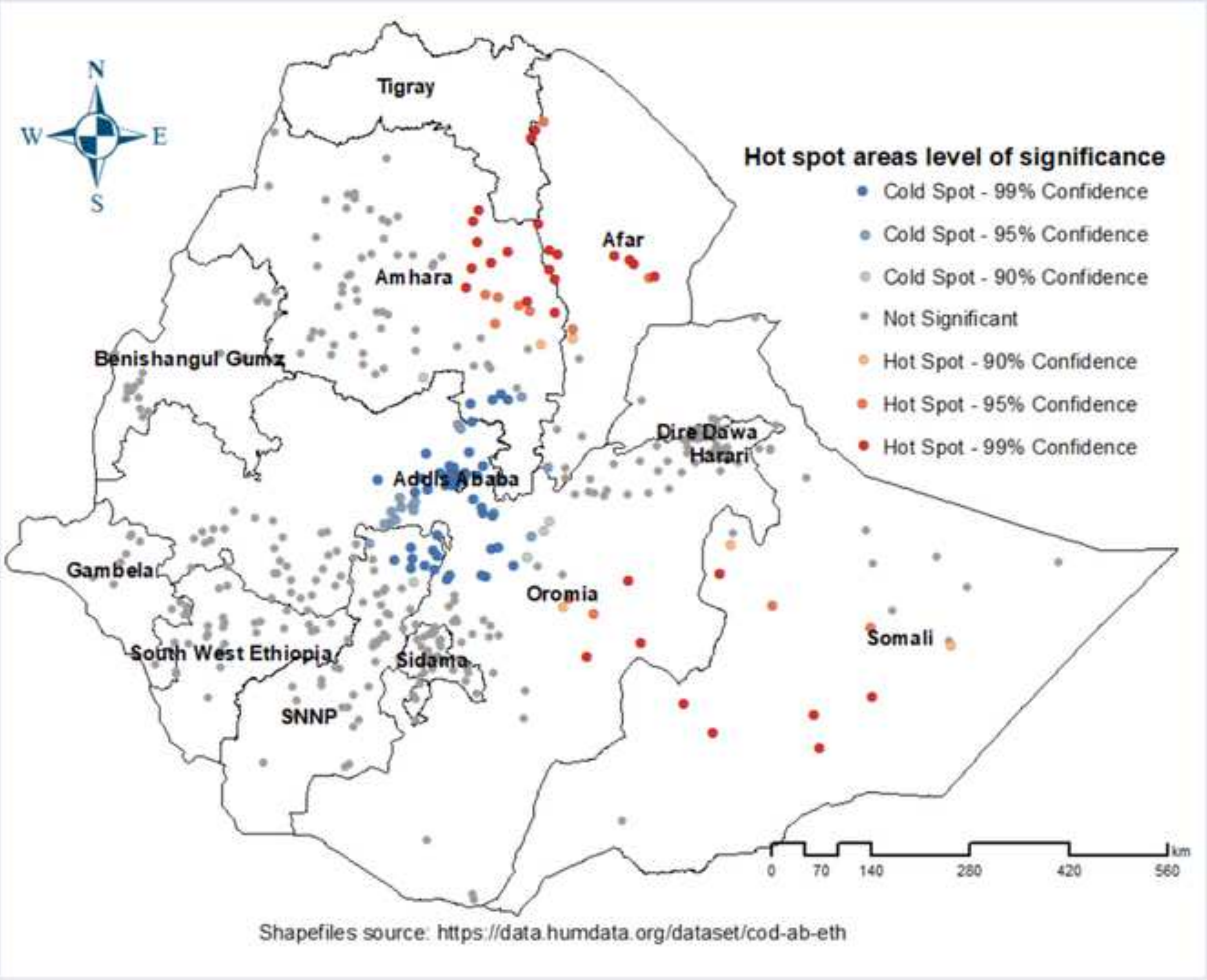

Figure 5 Click here to access/download;Figure;Figure 5_SaTScan analyis of zerodose.tif

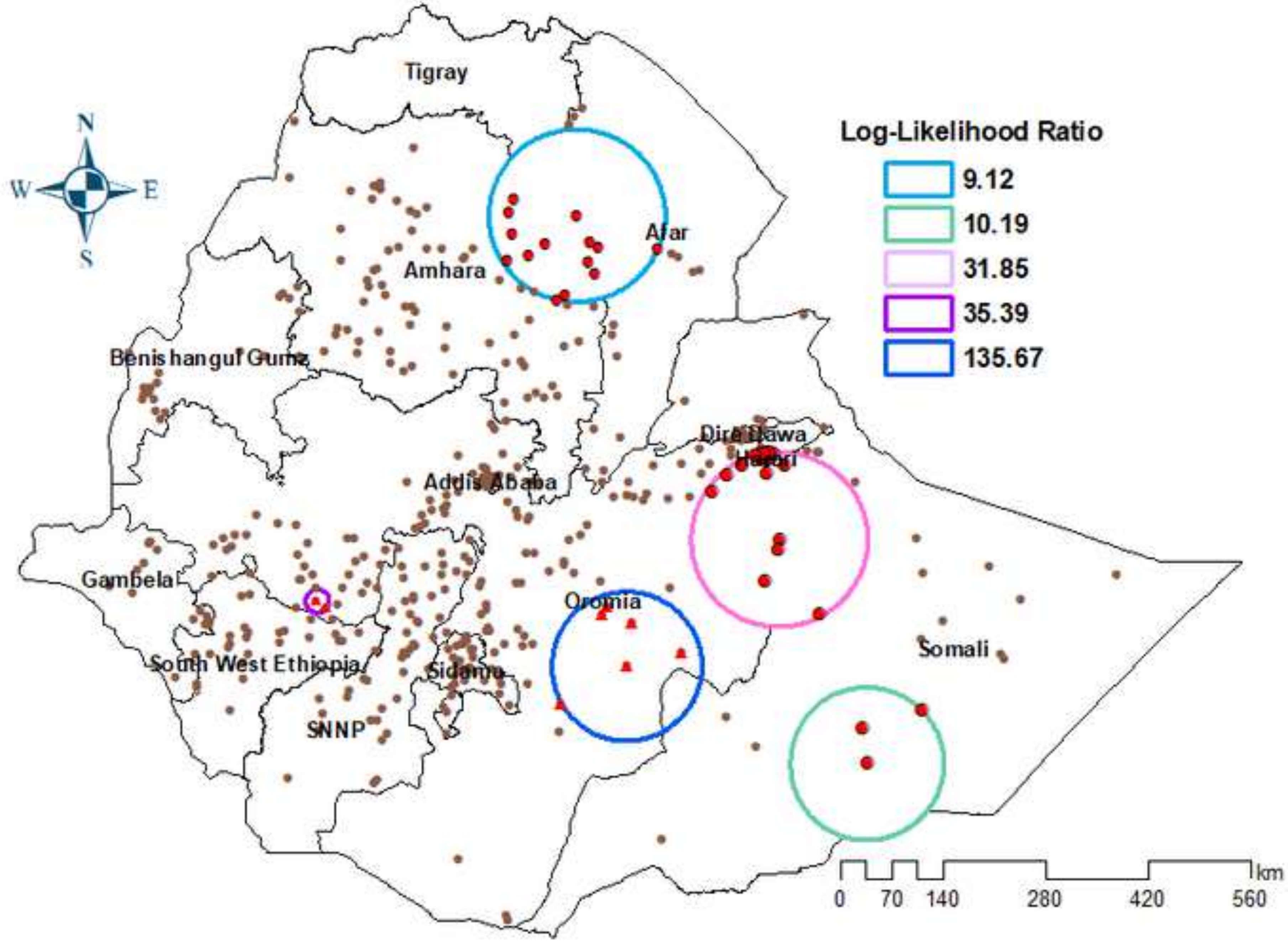

Shapefiles source: https://data.humdata.org/dataset/cod-ab-eth

Figure 6

Click here to access/download;Figure;Figure 6_Zerod_interpolated.png

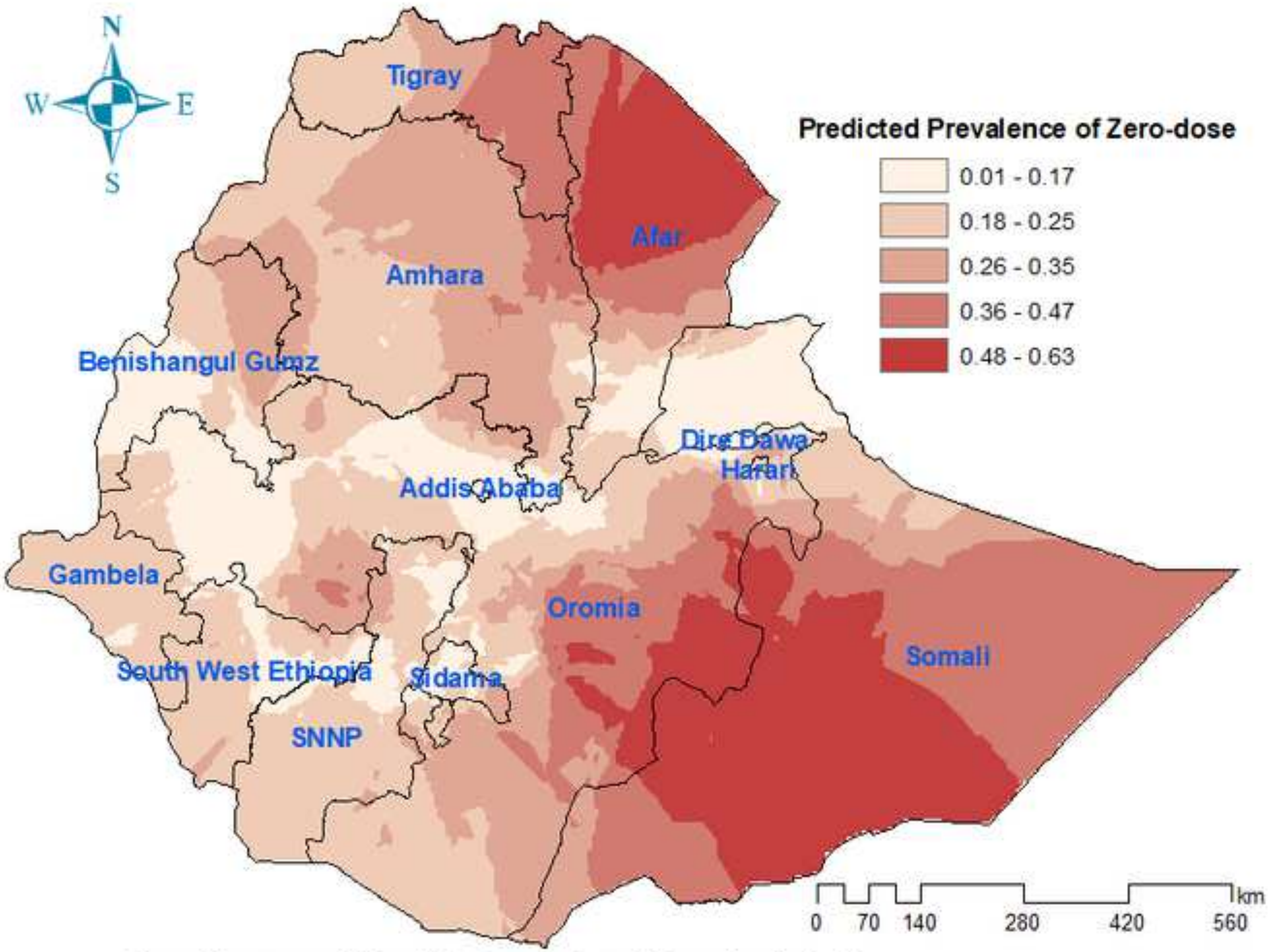

Shapefiles source: https://data.humdata.org/dataset/cod-ab-eth

Figure 7 Click here to access/download;Figure;Figure 7_GWR_.tif

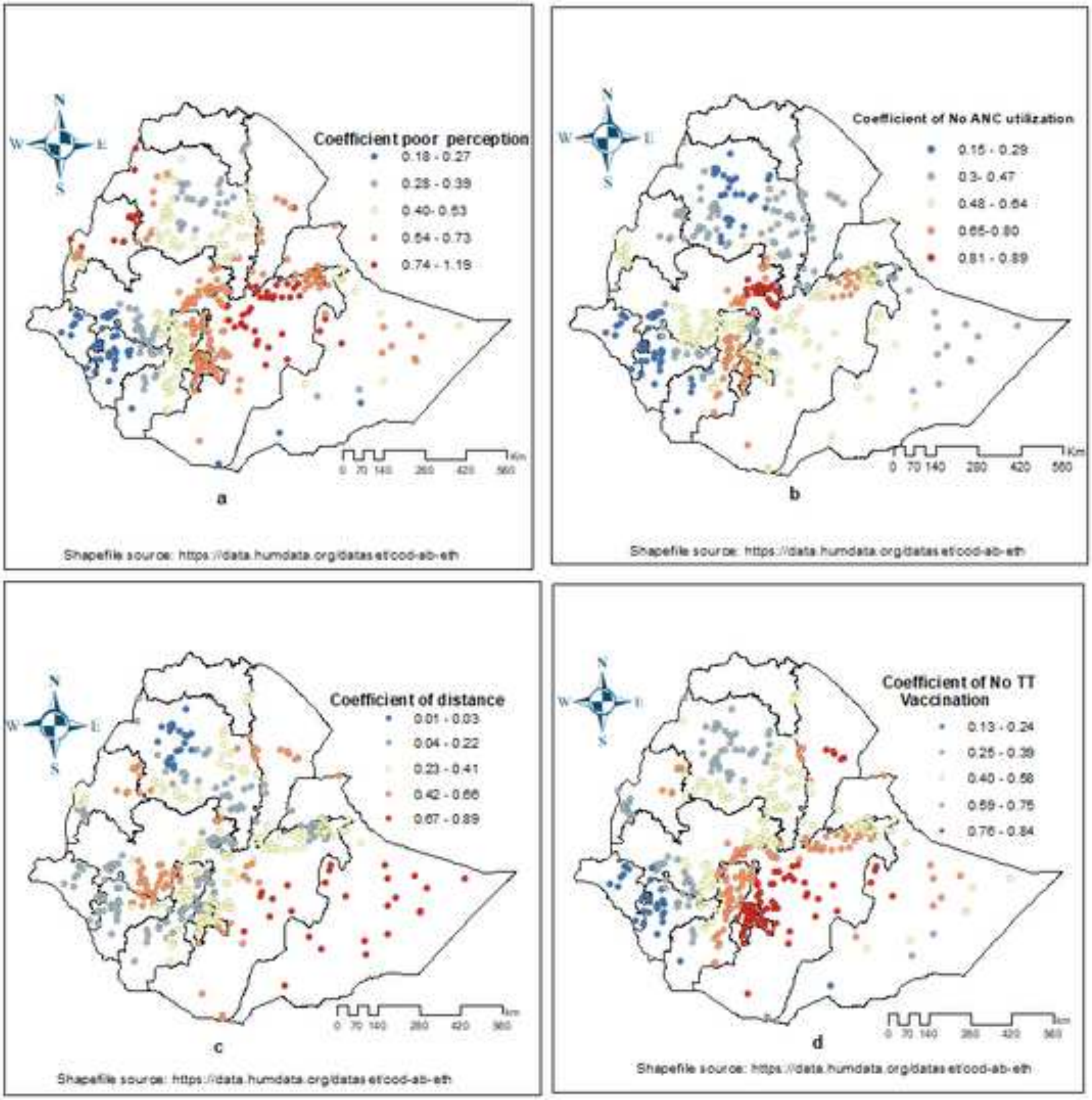

Supplement: Attachment — Submitted filename: PONE-D-24-59668 (1).pdf [file pone.0332162.s003.pdf]
