# Supplementary material for: Spatial disparities in zero-dose vaccination coverage for children aged 12–23 months in Ethiopia: A geographically weighted regression analysis
Source: PLoS One. 2025 Sep 11;20(9):e0332162. doi: 10.1371/journal.pone.0332162 (PMC12425285; doi:10.1371/journal.pone.0332162)
Supplement: Attachment — Submitted filename: National_immunization_program_evaluation 2023-ETHIOPIA.pdf [file pone.0332162.s005.pdf]

National Immunization Program Evaluation Research in Ethiopia

Consortium of CBMP Universities in Ethiopia

Addis Ababa University
Haramaya University
Hawassa University
Jimma University
University of Gondar

May, 2024

Addis Ababa, Ethiopia.

National Immunization Program Evaluation Steering Committee and Co-Principal Investigators

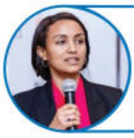

Dr. Meseret Zelalem
MCAH LEO
Chair

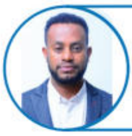

Mr. Melkamu Ayalew
EPI Manager
Secretory

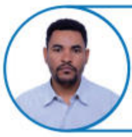

Mr. Yohannes Lakew
Senior Advisor
Member

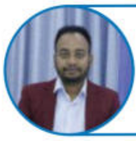

Mr. Naod Wondrad
Strategic Affairs EO
Member

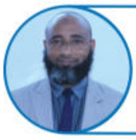

Mr. Mesoud Mohammed
Strategic Affairs A/EO
Member

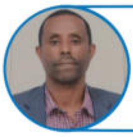

Dr. Kasahun Negash
Project Manager
Member

National Immunization Program Evaluation Technical Coordinating Committee

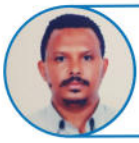

Mr. Sileshi Solomon
National Immunization Advisor
Chair and Co-PI

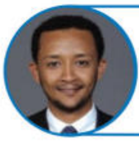

Mr. Admas Abera
Assistant Professor of Epidemiology,
Haramaya University – Secretary and Co-PI

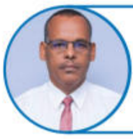

Dr. Keneni Gutema – Associate Professor of
Health Systems, Management and Economics,
Hawassa University – Member and Co-PI

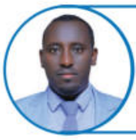

Mr. Berhanu Fikadie
Assistant Professor in public Health and
Health Informatics,
University of Gondar: Member and Co-PI

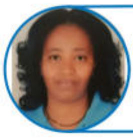

Dr. Mulumebet Abera
Associate Professor of Reproductive Health
Jimma University: Member and Co-PI

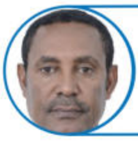

Dr. Girma Taye
Associate Professor of Biostatistics
Addis Ababa University: Member and Co-PI

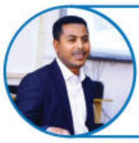

Dr. Binyam Tilahun
Associate Professor of
Health Information Systems
University of Gondar: Member and Co-PI

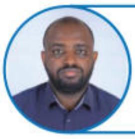

Dr. Mikiyas Teferi
(MD)
Member and Co-PI

List of Research Teams

Name	Organization	Name	Organization
Admas Abera	Haramaya University	Dr. Melaku Haile	Hawassa University
Dr. Keneni Gutema	Hawassa University	Netsanet Abera	Hawassa University
Dr. Girma Taye	Addis Ababa University	Ayantu Melke	Arbaminch University
Dr. Mulumebet Abera	Jimma University	Mustefa Gelagn	Arbaminch University
Dr. Binyam Tilahun	University of Gondar	Mastewal Kerebih	Ministry of Health
Berhanu Fikadie	University of Gondar	Dawit Wolde	Jimma University
Dr. Meseret Zelalem	Ministry of Health	Dr. Yibelta Kifle	Jimma University
Melkamu Ayalew	Ministry of Health	Professor Zewdie Berhanu	Jimma University
Yohannes Lakew	Ministry of Health	Chaltu Fikru	Jimma University
Sileshi Solomon	Ministry of Health	Gelila Abraham	Jimma University
Naod Wondirad	Ministry of Health	Asaye Birhanu	Jimma University
Mesoud Mohammed	Ministry of Health	Feyissa Tolosa	Jimma University
Dr. Mulugeta Tamire	Addis Ababa University	Ketema Lemma	Jimma University
Dr. Meaza Gezu	Addis Ababa University	Girma Hailemariam	Ministry of Health
Yimer Seid	Addis Ababa University	Mekdelawit Mengesha	Ministry of Health
Tesfamichael Awoke	Addis Ababa University	Professor Kassahun Alemu	University of Gondar
Awgichew Teshome	Addis Ababa University	Getasew Amare	University of Gondar
Tigist Habtamu	Addis Ababa University	Dr. Lemma Derseh	University of Gondar
Ephrem Biruk	Addis Ababa University	Dr. Bikes Destaw	University of Gondar
Tekalign Admasu	Ministry of Health	Dr. Yohannis Habtu	University of Gondar
Habtamu Alemay	Ministry of Health	Mulat Nigus	Ministry of Health
Behailu Hawulte	Haramaya University	Tamirat Awol	Ministry of Health
Dr. Merga Dheressa	Haramaya University	Prof. Gurmesa Tura	Jimma University
Dr. Tesfaye Gobena	Haramaya University	Dr. Misra Abdulahi	Jimma University
Lemma Demissie	Haramaya University	Muluneh Getachew	Jimma University
Saba Hailu	Haramaya University	Fitsum Yeneneh	Haramaya University
Kibrom Abraham	Ministry of Health	Dr. Tesfahun Melese	University of Gondar
Yakob Wondirad	Ministry of Health	Dr.Ahmed Mohammed	Haramaya University
Alemu Tamiso	Hawassa University		
Tomas Yeheyis	Hawassa University		

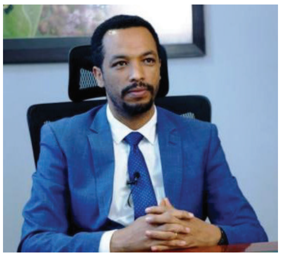

Forward

Ethiopia has so far achieved tremendous results in reducing deaths among children in the last two decades including a decline in under-5 mortality from 123 deaths per 1,000 live births in 2005 to 59 deaths per 1,000 live births in 2019, and a decline in infant mortality from 77 to 47 deaths per 1,000 live births and the key achievements and success comes in reality with the implementation immunization program and live saving interventions at the community and facility level. The national Expanded Program on Immunization (EPI) in Ethiopia started in 1980 G.C. with the aim of reducing mortality, morbidity, and disability among children, mothers and adults. It has substantially reduced deaths and illnesses from vaccine preventable diseases. The program operates within the context of the national health system, in alignment with global and regional strategies. In this regard, children under one year of age, children in their second year of life, adolescent girls (9- 14 years of age), women of reproductive age group (15- 49 years of age), and adults above 12 years of age are the targets for the currently available vaccines in the immunization program of the country.

Moreover, successes, previous achievements and recognitions shall be appraised in the lenses of global landscape and international standards. These gives more room for improvement and lots of cup to fill the growing demand of the communities for health care system, new vaccine introduction, VPD outbreaks and newly emerged pandemics. These needs robust evidences for immunization program and the health system improvement and needs research institutes, academia's, individual researchers, innovators, Partners, CSA to generate evidences used as a baseline for program implementation and reduction of morbidity and mortality.

With the lead of Ministry of Health (MOH) and Capacity Building and Mentorship (CBMP) Universities in collaboration with AMREF-ACDC-SLL Project, WHO, UNICEF, JSI and PATH had conducted a National Immunization Program Evaluation and it's the first in the history of immunization program implementation since 1980 G.C. The evaluation used as WHO EPI review guide and recommendation as its reference for the country's immunization program evaluation aimed at addressing the success and gaps of the immunization program through the lenses of leadership, management, and governance, immunization, service delivery, the supply chain management, vaccine safety and quality, VPD surveillance, demand generation and communication, and monitoring and evaluation pillars of immunization program. Throughout the survey period multiple stake holders engaged in all levels of the health system including national and sub-national health administration, agencies, health facilities, health posts and the community.

Ministry of Health greatly acknowledges the principal survey coordinators, EPI and CBMP universities research team, AMREF-ACDC-SLL Project, WHO, UNICEF, JSI and PATH for their technical, financial and logistics contribution to generate the evidence for immunization program improvement. The ministry also thanks MCAHS LEO, Strategic Affairs and Immunization Service Desk for the great coordination and dedication of using evidence as strategy to improve the program. Finally, we call upon all health sector stakeholders especially health system leaders, donors and partners to use this evidence as a baseline for a responsive and resilient health system.

H.E. Dr. Dereje Duguma, (MD, MPH)

State Minster, Minstiry of Health, Ethiopia

Acknowledgements

This national level study was conducted by the CBMP universities consortium in Ethiopia: Addis Ababa University, Haramaya University, Hawassa University, Jimma University, and University of Gondar. This work is the culmination of a collaborative effort by a dedicated team of researchers and stakeholders.

We, the CBMP consortium, would like to express our sincere gratitude to the following individuals and organizations for their invaluable contributions to this national-level study. We are particularly grateful to the Ministry of Health of Ethiopia for providing the opportunity and the necessary permissions to conduct this important study. We deeply appreciate the technical guidance and support offered by the MoH staff throughout the research process.

This research was made possible by the financial support of Amref Health Africa. Their commitment to fund this study as well their technical contributions is deeply appreciated. Moreover, we are grateful to the World Health Organization (WHO), UNICEF, JSI, and PATH, for providing logistical support during data collection.

Our sincere thanks goes to the regional health bureaus, zonal health departments, woreda health offices, health facilities, and health posts across the country. Their willingness to share data and facilitate fieldwork was instrumental to the success of this study. We express our heartfelt gratitude to the communities who willingly participated and provided valuable data for this study.

Last but not least, we would like to express our sincere gratitude to our dedicated field team for their tireless efforts in collecting data across the country. This includes our field supervisors who provided invaluable guidance and support, facility level data collectors who ensured accurate data collection at health facilities, and field data collectors who worked diligently in the communities.

Acronyms

ACSM	Advocacy, Communication and Social Mobilization
AEFI	Adverse Event Following Immunization
CBHI	Community Based Health Insurance
CEIP	Current expenditure for Immunization Programs
CSO	Civil Service Organizations
CTMS	Computerized Temperature Monitoring System
DOR	Drop Out Rate
DPT	Diphtheria Pertussis Tetanus
EA	Enumeration Area
EDHS	Ethiopia Demographic and Health Survey
EFDA	Ethiopian Food and Drug Authority
EFY	Ethiopian Fiscal Year
EPI	Expanded Program on Immunization
EPSS	Ethiopian Pharmaceutical Supply Service
ESS	Ethiopian Statistical Services
EVM	Effective Vaccine Management
FGD	Focus Group Discussion
HEW	Health Extension Worker
HF	Health Facility
HIT	Health Information Technicians
HP	Health Post
HPV	Human Papilloma Virus
HR	Human Resource
HSTP	Health Sector Transformation Plan
IPC	Inter Personal Communication
IRB	Institutional Review Board
KII	Key Informant Interview
LD	Lower Distribution
LMG	Leadership, Management and Governance
LMICs	Lower and Middle Income Countries
LMIS	Logistics Management Information System
MCH	Maternal and Child Health
MCV	Measles antigen-containing vaccines

MoH	Ministry of Health
MYP	Multi-Year Plan
NGO	Non-Governmental Organization
NNT	Neonatal Tetanus
ODK	Open Data Kit
OPV	Oral Polio Vaccine
PHCU	Primary Health Care Unit
PHEM	Public Health Emergency Management
PIRI	Periodically Intensified Routine Immunization
REC	Reach Every Child
RED	Reach Every District
RHB	Regional Health Bureau
SC	Sub-Central
SD	Standard Deviation
SDD	Solar Direct Device
SNNP	Southern Nations Nationalities and Peoples
SOP	Standard Operating Procedure
SP	Service Provision
Td	Tetanus diphtheria
TWG	Technical Working Group
UN	United Nations
UNICEF	United Nations Children's Fund
VPD	Vaccine-Preventable Disease
VVM	Vaccine Vial Monitor
WHO	World Health Organization
WoHO	Woreda Health Office
ZHD	Zonal Health Department

Table of Contents

**List of Contributors and Recommended Citations**	**II**
**Foreword**	**IV**
**Acknowledgements**	**V**
**Acronyms**	**VI**
**Table of Contents**	**VIII**
**List Figures**	**XI**
**List of Tables**	**XV**
**Executive Summary**	**XXIII**

**1.**	**Introduction**	**1**
**2.**	**Survey Methods**	**5**
2.1.	Overview	5
2.2.	Survey Design	5
2.3.	Study Populations	5
2.4.	Sample Size and Sampling Strategies	5
2.5.	Data Collection Methods and Instruments	9
2.6.	Recruitment and Training of Field Staff	11
2.7.	Field Work	11
2.8.	Pretest	11
2.9.	Data Management and Analysis	12
2.10.	Ethical Consideration	12
**3.**	**Leadership, Manegement and Governance**	**13**
3.1.	Planning, governance, and coordination	14
3.2.	Human resource	22
3.3.	Immunization program structure	25
3.4.	Micro-planning	28
3.5.	Resource mobilization and sustainability of financing	30
3.6.	New vaccine introduction	40
3.7.	Conclusions	44
3.8.	Recommendations and Implications	44
**4.**	**Supply Chain Management**	**45**
4.1.	Immunization Supply chain operations and management at central and sub-central levels	47
4.2.	Immunization supply chain operations and management at lowest dist0ribution (LD) and service provision (SP) levels	55
4.3.	Supply chain essentials at central and sub central level	75

4.4. Supply chain essentials at lower distribution and service provision levels 81
4.5. Effective vaccine management (EVM) summary report 85
4.6. Conclusions 88
4.7. Recommendations 89

5. Immunization Service Delivery 90

5.1. Immunization Service Availability 91
5.2. Immunization Service Readiness 94
5.3. Immunization Service Delivery Strategy and Integration Approaches 98
5.4. Immunization Session Quality 104
5.5. Conclusions 133
5.6. Recommendation 134

6. Adverse Events Following Immunization (AEFI) 136

6.1. Adverse Event Following Immunization (AEFI) 136
6.2. Types of Adverse Events Following Immunization 137
6.3. Measures taken for adverse effect following immunization by the community 140
6.4. Measures taken for adverse events following HPV vaccination 141
6.5. Compensations in place for AEFI 141
6.6. AEFI cases reported from health facilities 142
6.7. Roles of health facilities in investigating and managing AEFI cases 143
6.8. Pharmacovigilance characteristics 144
6.9. Availability of resources and inputs for AEFI surveillance and management 146
6.10. Regulatory Actions taken by the pharmacovigilance centers 147
6.11. AEFI Surveillance System challenges 148
6.12. Conclusions 154
6.13. Recommendations and Implications 154

7. Vaccine Preventable Disease (VPD) Surveillance 156

7.1. Case-based surveillance system 156
7.2. Human Resource and Governance of VPD Surveillance 158
7.3. VPD surveillance activities conducted by health facilities 159
7.4. VPD Surveillance Reporting 161
7.5. VPD Surveillance reporting frequency, timeliness and completeness 161
7.6. Vaccine Preventable Diseases surveillance implementation challenges 165
7.7. Conclusions 168
7.8. Recommendations and Implications 169

8. Demand Generation and Communication 172

8.1. Chldhood vaccinations: Awareness, acceptance/attitude, hesitance and mitigation 171
8.2. Community's perception and attitude towards childhood vaccination 177
8.3. Perceived access and level of satisfaction with vaccination services 178
8.4. Role of Gender in immunization service uptake/Gender inter-sectionality 181
8.5. COVID -19 infection and vaccine: Awareness, beliefs, acceptance and hesitations 182
8.6. COVID-19 vaccine uptake 191
8.7. HPV infection and vaccine: Awareness, acceptance/ attitude and hesitance/concerns 196
8.8. Socio-cultural factors and immunization services uptake 207
8.9. IPC skills of health workers in immunization service delivery (GATHER approach) 208
8.10. EPI Advocacy, Communication and Social Mobilization (ACSM) 217
8.11. Community structures for immunization demand creation 219
8.12. Challenges in EPI advocacy, communications and social mobilization (ACSM) 220
8.13. Comprehensive ACSM suggested strategies to improve vaccine uptake 222
8.14. Conclusions 228
8.15. Recommendations and Implications 229

9. Monitoring and Evaluation 230

9.1. Immunization M&E Structure 230
9.2. Immunization data quality 240
9.3. Immunization Data Use 241
9.4. Conclusions 267
9.5. Recommendations and implications 268

References 269

List of Figures

Figure 3.1: Percentage of governance practices in woreda health office of Ethiopia, 2023 19

Figure 3.2: The Immunization Management Structure of Ethiopia (MoH, 2022) 25

Figure 3.3: Presence of updated micro-planning at health posts in Ethiopian health care system by region 2023. 29

Figure 3.4: Percentage of critical activities of the immunization program canceled due to lack of budget at health posts in health care system of Ethiopia 2023 36

Figure 3.5: Annual total expenditure in USD for Immunizations in Ethiopia, 2016-2020 37

Figure 4.1: AVaccine stock record management practice of lower and Service Provision levels by health institution types. 60

Figure 4.2: Availability of shake test and multi-dose vial policy/procedure by health facility at the LD and SP levels in Ethiopia. 65

Figure 5.1: Percentage of facilities that offer immunization services in Ethiopia 2023. 91

Figure 5.2: Availability of tracer items (percent) for immunization services among health facilities in Ethiopia 2023. 94

Figure 5.3: Proportion of health facilities with at least one staff got immunization related training in the last two years prior to the survey by training types in Ethiopia. 2023 98

Figure 5.4:. Immunization service integration and guideline availability by the health facilities across regions in Ethiopia, 2023. 100

Figure 5.5: Percentage of health posts who had experience of missing scheduled vaccination sessions in Ethiopia, 2023. 103

Figure 5.6: Immunization service integration and guideline availability by health posts across regions in Ethiopia, 2023. 104

Figure 5.7: Mother's satisfaction on immunization services by regions in Ethiopia, 2023 111

Figure 5.8: National vaccination coverage on selected antigens, dropouts and zero-dose among children aged 12 to 23 months in Ethiopia, 2023. 112

Figure 5.9: Child vaccination verification approaches by antigens in Ethiopia, 2023 113

Figure 5.10: Pentavalent 3 coverage and drop-out rates by region in Ethiopia, 2023 117

Figure 5.11: Pentavalent 3 coverage by selected characteristics in Ethiopia, 2023 118

Figure 5.12: Full immunization/all vaccines coverage by regions in Ethiopia, 2023 119

Figure 5.13: Full immunization/all vaccines coverage by selected characteristics in Ethiopia,2023 120

Figure 5.14: Zero dose coverage b regions in Ethiopia, 2023 121

Figure 5.15: Zero dose coverage by selected characteristics in Ethiopia, 2023 122

Figure 5.16: MCV 2 coverage by regions in Ethiopia, 2023 123

Figure 5.17: Td vaccination coverage by different doses in Ethiopia, 2023. 125

Figure 5.18: HPV vaccination coverage by doses in Ethiopia, 2023 128

Figure 5.19: COVID-19 vaccination coverage by respective dose 131

Figure 6.1: Prevalence of adverse effect following immunization based on types of vaccine in Ethiopia, 2013 137

Figure 6.2: Types of adverse events following immunization among children received routine vaccine in Ethiopia, 2023 138

Figure 6.3: Common adverse effect following immunization among adult received COVID vaccine, in Ethiopia, 2023. 139

Figure 6.4: Common adverse events following immunization among adolescent girl who received Human Papilloma Virus vaccine in Ethiopia, 2023. 139

Figure 6.5: Number of AEFI cases reported from health facilities and health posts by regions in Ethiopia, 2023. 142

Figure 6.6: Roles of Health Facilities in AEFI investigation and Management by regions of Ethiopia, 2023. 143

Figure 6.7: Availability of pharmacovigilance center and resource availability among health facilities in Ethiopia, 2023. 145

Figure 6.8: Regulatory Actions taken by health facilities for AEFI by regions of Ethiopia, 2023. 148

Figure 7.1: Measles cases reported by health facilities in the 12 months preceding the survey in Ethiopia, 2023. 157

Figure 7.2: Reported rubella cases in the studied facilities 12 months preceding the survey in Ethiopia 2023. 157

Figure 7.3: Neonatal cases reported by studied health facilities in the 12 months preceding the survey, 2023. 158

Figure 7.4: Report Completeness of VPD surveillance data among health facilities by regions of Ethiopia, 2023. 164

Figure 8.1: Reasons for mothers/caregivers not vaccinated their children in Ethiopia, 2023 176

Figure 8.2: Relationship between local leaders' involvement and magnitude of Zero dose in Ethiopia 177

Figure 8.3: Percentage of respondents who ever heard about COVID -19 by region, Ethiopia 2023 185

Figure 8.4: Source of information for COVID-19 vaccine among Study participants, Ethiopia, 2023 185

Figure 8.5: COVID-19 disease concern among study participants in Ethiopia, 2023 188

Figure 8.6: Reason for not taking HPV vaccination among adolescents in Ethiopia 204

Figure 8.7: Motivators for uptake of HPV vaccination among adolescents in Ethiopia, 2023 206

Figure 8.8: Proportion of health workers who received EPI focused training (a) and supportive supervision (b), 2024, Ethiopia 210

Figure 8.9: IPC practice of health workers in Immunization service delivery by location (place of residence) and facility type, Ethiopia, 2023 215

Figure 8.10: IPC practice of health workers in Immunization service delivery by status on getting EPI focused training and supportive supervision, Ethiopia, 2023 216

Figure 9.1: Data accuracy of reports for the E.I. data at different health service levels in Ethiopia, 2023. 246

Figure 9.2: Distribution of health facilities EPI Data accuracy by facility type in Ethiopia, 2023 247

Figure 9.3: Frequency of the performance monitoring team meetings at district level held during the 3rd quarter of 2015 EFY. 251

Figure 9.4: Proportion of WoHOs that conducted performance monitoring based on key performance indicators and RHIS data in Ethiopia, 2023. 252

Figure 9.5: Type of decisions made based on the discussion of the district/sub-city and/or health facility's EPI program performance at district level in Ethiopia, 2023. 253

Figure 9.6: Proportions of Woredas conducted regular analysis and held data review meeting in Ethiopia, 2023. 260

Figure 9.7: Proportion of HPs that got supportive supervision in the 3rd quarter of 2015 EFY in Ethiopia, 2023. 262

Figure 9.8: Electronic systems used by the health facilities for EPI data entry and analysis in Ethiopia, 2023. 263

List of Tables

Table 1.1: Thematic areas of EPI (Adapted from WHO EPI Review Guide) 4

Table 2.1: Sample size for household survey by region and target population 6

Table 2.2: Number of Key informant interviews and FGDs at national, subnational, facility, and community levels in Ethiopia 2023. 8

Table 3.1: Availability of strategic plan, operational plan, updated population demographics, micro plan, stakeholder engagement, and joint meeting at Woreda level in Ethiopian health care system by region, 2023 15

Table 3.2: Availability of strategic plan, operational plan, updated population demographics, micro plan, joint meeting at Facility level in Ethiopian health care system by region, 2023 16

Table 3.3. Availability of governing bodies, national immunization guideline, technical immunization guidelines, rewarding system, and refraining at Woreda and health facility level in Ethiopia by region, 2023. 17

Table 3.4: Availability of job aids, rewarding system, new vaccine introducing guideline, HR adequacy and measures to poor performer at health facility level in Ethiopia by region, 2023 18

Table 3.5: Level of Multi-sectoral collaboration, public-private coordination, coordinating immunization with other health care programs and joint meetings for coordination of at Woreda level in Ethiopian health care system by region, 2023. 20

Table 3.6: Level of Multi-sectoral collaboration, public-private coordination, coordination with NGO and the community at facility level in Ethiopian health care system by region, 2023. 21

Table 3.7: Immunization days lost, training on EP and LMG, supervision, and interruption of immunization service in either HP or other health facilities in Ethiopia by region and location, 2023 23

Table 3.8: Human resource work load of immunization at health posts based on the population projection in Ethiopia, 2023. 24

Table 3.9: Availability of organizational chart at the woreda and facility level in Ethiopian health care system by region 2023. 26

Table 3.10: Availability of program budget for immunization, budget adequacy, and the cancellation of critical immunization activities at Woreda level in Ethiopian health care system by region, 2023 32

Table 3.11: Availability of program budget for immunization and the cancellation of critical immunization activities at the health facility level in the Ethiopian health care system by region, 2023 35

Table 3.12: The costs of immunization in USD, their sources, and per capita costs, 2016-2020, Ethiopia. 38

Table 3.13: Shares of CEIP from domestic and external sources of funding 39

Table 3.14: Forecasted Immunization cost using the ETS algorithm, Ethiopia 2023 39

Table 3.15: Availability selected items in the HPs, HFs, and woredas that started new vaccines in the last 2 years. 42

Table 4.1: Temperature management of vehicles at Central and sub-Central hubs, Ethiopia 2023. 48

Table 4.2: Storage capacity of cold rooms, refrigerators, and dry storage area at the Central and sub-Central EPSS hubs Ethiopia, 2023. 49

Table 4.3: Dry good storage at the Central and Sub Central EPSS hubs 50

Table 4.4: Maintenance of refrigerator's equipment of the Central and Sub-Central Hubs 51

Table 4.5: Vaccine stock record and arrangements of the Central and sub-Central EPSS hubs 52

Table 4.6: Temperature management of vaccine storage equipment by health institutions 55

Table 4.7: Vaccine storage management practice by health facility type 57

Table 4.8: Practice of maintenance & repair of buildings at the LD and SP level. 58

Table 4.9: Preventive maintenance and repair of refrigerators by health institution type at the LD and SP level. 59

Table 4.10: Stock management system by facility type at the LD and SP level. 61

Table 4.11: Stock record arrangements at lower and service provision levels by health institution type at the LD and SP level. 62

Table 4.12: Distribution of vaccines at lower distribution and service provision levels by health institutions type in Ethiopia. 63

Table 4.13: Vaccine management of the lower and service provision levels by health institution type in Ethiopia 2023. 66

Table 4.14: Vaccine injection practice by health institution at lower distribution and service provision levels 67

Table 4.15: Waste onsite storage by health institution type at lower distribution and service provision levels 69

Table 4.16: Waste disposal practice of lower and service provision levels by health institutions type in Ethiopia 2023. 71

Table 4.17: Immunization supply chain forecasting practice at the lower distribution and service provision levels by type of health institution 72

Table 4.18: Immunization supply chain work plan at lower distribution and service provisions level by health institutions in Ethiopia 2023. 73

Table 4.19: Immunization supply chain supervision at lower distribution and service provision levels by health institutions type. 74

Table 4.20: Immunization supply chain performance monitoring at lower distribution and service provision levels by health institution types 74

Table 4.21: Utilities and services in EPSS Central and sub-Central hubs 75

Table 4.22: Building amenities and conditions at the central and sub-central hubs 76

Table 4.23: Cold/freezer rooms at Central and sub-Central hubs in Ethiopia, 2023. 78

Table 4.24: Cold and other vaccine storage equipment in EPSS Central and Sub Central hubs in Ethiopia 2023. 80

Table 4.25: Information technology at lower distribution and service provision levels by health institution types in Ethiopia 2023 81

Table 4.26: Human Resource/Staff at lower distribution and service delivery level by facility type in Ethiopia, 2023. 82

Table 4.27: Staff knowledge on temperature management of vaccines at the lower distribution and service provision levels by health institutions type in Ethiopia 2023. 83

Table 4.28: Staff knowledge on stocks management of LD and SP levels by type of health institution in Ethiopia 2023. 84

Table 4.29: Availability of Standard Operating Procedures (SOPs) for routine operations at the LD and SP levels in Ethiopia 2023. 85

Table 4.30: Effective vaccine management Ethiopia 2023 facility operations and management score 86

Table 4.31: Comparison of Supply chain management findings from 2013, 2019 and 2023 EVM assessments in Ethiopia. 87

Table 5.1: Immunization service availability (percentage) by region, facility type and setting, Ethiopia 2023 92

Table 5.2: Percentage of facilities with tracer items for immunization services by region, facility type, and setting Ethiopia, 2023. 96

Table 5.3: Approaches used by health facilities to reach zero-dose and under-immunized children in Ethiopia, 2023. 99

Table 5.4: Percentage of immunization services delivery strategies by setting and approaches implemented among studied health posts 2023, Ethiopia. 102

Table 5.5: Vaccination session safety during the preparation/pre-administration phase across regions, facility types and settings in Ethiopia, 2023. 105

Table 5.6: Vaccination safety practice during administration in Ethiopia across regions, setting and facility type, 2023. 108

Table 5.7: Post-administration vaccination safety practice by region, setting and facility type, and service delivery approach in Ethiopia, 2023. 110

Table 5.8: Vaccination coverage aged 12-23 months across selected socio-demographic and economic characteristics in Ethiopia, 2023. 114

Table 5.9: MCV2 vaccination coverage aged 24-35 months across selected socio-demographic and economic characteristics in Ethiopia, 2023. 124

Table 5.10: Td maternal vaccination coverage across basic characteristics in Ethiopia, 2023. 126

Table 5.11: HPV vaccination coverage across different background characteristics in Ethiopia, 2023 129

Table 5.12: COVID-19 vaccination coverage across different socio-demographic characteristics in Ethiopia, 2023. 132

Table 6.1: Measures taken for adverse events following child immunization among children in Ethiopia 2023. 140

Table 6.2: Types of measures taken for adverse event following HPV vaccination among 15-18 girls in Ethiopia, 2023. 141

Table 6.3: Compensation for adverse event following human papilloma various vaccination among 15-18 years old adolescent girls in Ethiopia, 2023 141

Table 6.4: Roles of health facilities in managing and investigating adverse effect following immunization by type and location, in Ethiopia, 2023 144

Table 6.5: Pharmacovigilance Center Characteristics by type and location of health facilities in Ethiopia, 2023. 145

Table 6.6: Availability of inputs needed for adverse event following immunization surveillance and Management in Ethiopia, 2023. 146

Table 6.7: Availability of inputs needed for AEFI surveillance and Management in Ethiopia, 2023. 146

Table 6.8: Regulatory Actions taken by the pharmacovigilance center by type of facilities and region in Ethiopia, 2023. 147

Table 6.9: Summary of structural, technical and behavioral related problems of AEFI surveillance in Ethiopia 2023. 150

Table 7.1: Human Resource and Governance of VPD surveillance by type of facilities and location in Ethiopia, 2023. 159

Table 7.2: Vaccine preventable disease surveillance capacity building, communication and networking in Ethiopia 2023. 160

Table 7.3: Vaccine preventable disease surveillance reporting frequency by types and location of facilities in Ethiopia, 2023. 159

Table 7.4: Vaccine preventable diseases surveillance reporting frequency in Ethiopia, 2023. 162

Table 7.5: Vaccine preventable disease surveillance report timeliness and completeness in Ethiopia, 2023. 163

Table 7.6: Summary of VPD surveillance implementation structural, technical and behavioral challenges in Ethiopia 2023. 166

Table 8.1: Childhood vaccination information, source of information and contents of information among caregivers in Ethiopia, 2023. 172

Table 8.2: Knowledge of childhood vaccination among mothers/caregivers in Ethiopia, 2023. 174

Table 8.3: Childhood vaccination status and willingness of mothers to get their children to be vaccinated, Ethiopia 2023 175

Table 8.4: Community perception and attitude towards childhood vaccination in Ethiopia, 2023 177

Table 8.5: Perception of support and approval for childhood vaccination in Ethiopia, 2023 178

Table 8.6: Perception and experience of mothers with childhood vaccination services in Ethiopia, 2023 179

Table 8.7: Perceived access to vaccination services and level of satisfaction among mothers/caregivers in Ethiopia, 2023 180

Table 8.8: Reasons for dissatisfaction of caregivers/mothers with immunization service in Ethiopia 2023 181

Table 8.9: Participants' characteristics of COVID-19 disease and vaccine survey, Ethiopia, 2023. 183

Table 8.10: Awareness about COVID-19 among study participants, Ethiopia, 2023 184

Table 8.11: Perception of study participants about COVID-19 infection, risk group, where to get vaccine and vaccination available in Ethiopia, 2023. 187

Table 8.12: COVID-19 disease and vaccine awareness, and perception of vaccines safety and importance among study participants, Ethiopia, 2023 190

Table 8.13: Respondent perception about communities' acceptance of COVID-19 vaccines and supports in Ethiopia 2023 192

Table 8.14: Access to COVID-19 vaccine and satisfaction with vaccination services among respondents Ethiopia 2023. 194

Table 8.15: Attitude toward COVID-19 vaccine and other prevention measures among Study participants in Ethiopia, 2023 195

Table 8.16: Attitude toward COVID-19 vaccine among study participants by socio-demographic characteristics in Ethiopia, 2023 196

Table 8.17: HPV infection information, sources of information and knowledge about the infection among adolescents in Ethiopia, 2023. 198

Table 8.18: Perception and source of information relating to HPV vaccine 200

Table 8.19: Attitude, acceptance and believe about HPV infection and vaccination among adolescents in Ethiopia, 2023 202

Table 8.20: Attitude toward HPV vaccine among study participants by socio-demographic characteristics in Ethiopia, 2023 203

Table 8.21: Concerns of HPV vaccination among adolescents in Ethiopia,2023 187

Table 8.22: Demographic characteristics of IPC Skill assessment respondent, Ethiopia, 2023 210

Table 8.23: IPC process and skills practiced by health workers during immunization service delivery following GATHER approach in Ethiopia, 2023 212

Table 8.24: Other IPC activities practiced by health workers during immunization service delivery in Ethiopia, 2023 213

Table 8.25: Mean score of IPC practice among health workers proving Immunization service by region, 2024 214

Table 8.26: Barriers to practicing good IPC in immunization service delivery as reported by health workers in Ethiopia, 2023 217

Table 8.27: EPI communication or education settings/platforms and participants in Ethiopia, 2023 218

Table 8.28: Comprehensive suggestions to improve to uptake of vaccine: ACSM 223

Table 8.29: COVID-19 hesitation mitigation strategies, and suggested vaccine communication strategies in Ethiopia 227

Table 9.1: Available M&E Mechanisms for EPI program monitoring and evaluation at different health system levels in Ethiopia, 2023. 232

Table 9.2: Immunization data management responsible person at different health system level in Ethiopia, 2023 234

Table 9.3: Guidelines, formats, and logbooks for EPI data management at all health system level, 2023. 236

Table 9.4: Planning and demographic-related information of immunization by zonal health department, 2023 237

Table 9.5: Demographic and planning characteristics of Districts/Sub-cities in Ethiopia, 2023. 238

Table 9.6: Immunization planning and demographic characteristics at the health facilities in Ethiopia, 2023 239

Table 9.7: EPI annual plan and demographic characteristics of health Posts in Ethiopia, 2023 240

Table 9.8: Report completeness, timelines, and performance monitoring of Immunization at the regional level, Ethiopia 2023. 241

Table 9.9: Report completeness, timeless, and performance characteristics of Zonal health department, 2023 242

Table 9.10: Report completeness, timelines, and performance monitoring of immunization at the District/sub-city health offices, 2023 243

Table 9.11: EPI report completion and performance monitoring of Health facilities in Ethiopia, 2023 244

Table 9.12: Availability of reports, IQAS, report completeness, and timelines of health Posts in Ethiopia, 2023 245

Table 9.13: The proportion of health facilities with acceptable accuracy of reports (Weighted percent) in Ethiopia, 2023 247

Table 9.14: The proportion of health posts with acceptable accuracy of reports (Weighted percent) in Ethiopia, 2023 248

Table 9.15: Proportion of health facilities with complete and timeliness report by background characteristics in Ethiopia, 2023. 249

Table 9.16: Proportion of health facilities that conducted data quality assessment mechanism by to background characteristics in Ethiopia, 2023. 250

Table 9.17: Percentage of Woreda/sub-city that conducted performance monitoring at the district level in by region of Ethiopia, 2023. 253

Table 9.18: EPI performance monitoring practices at the health posts level by background characteristics, in Ethiopia 2023. 254

Table 9.19: EPI performance practice at the health post level by background characteristics, in Ethiopia 2023. 255

Table 9.20: EPI data analysis and utilization on EPI data management of immunization in Zonal health department, in Ethiopia 2023. 256

Table 9.21: EPI data analysis and utilization of immunization data by district/sub-city health offices in Ethiopia, 2023. 257

Table 9.22: Immunization supply recoding practice of the health facilities in Ethiopia, 2023. 258

Table 9.23: Immunization performance monitoring and vaccine balance recording of health Posts in Ethiopia, 2023. 259

Table 9.24: Percentage of Woreda/sub-city that practiced data use of the EPI program by region of Ethiopia, 2023. 261

Table 9.25: Proportion of health facilities that conducted data processing and analysis by background characteristics in Ethiopia, 2023 264

Table 9.26: Percentage of Woreda and sub-cities that visualized and utilized the routine EPI data by region of Ethiopia, 2023. 266

Table 9.27: Percentage of Woreda/sub-city that disseminated data outside the health sector by region of Ethiopia, 2023. 267

Executive Summary

Background: Immunization is a vital, cost-effective intervention intended to avert mortality rates among children, adolescent girls, and women. Ethiopia launched the Expanded Program of Immunization (EPI) with six antigens (BCG, DPT, Measles, OPV) in 1980 with the aim of reducing mortality, morbidity, and disability among children, and currently fourteen antigens are being offered including HPV and COVID-19 vaccines. Tremendous results were achieved in reducing deaths among children over the last two decades, including a 52% decline in under-5 mortality between 2005 and 2019. Over the same period, infant mortality declined from 77 to 47 deaths per 1,000 live births.

Despite these achievements, the immunization program suffers from multifaceted problems ranging from policy frameworks from the delivery side to community perception on the receiver end. In recent years, the immunization program of Ethiopia has faced challenges from natural and artificial catastrophes including the COVID-19 pandemic, drought, war, and economic crisis, which resulted in shifting government priorities, interruption of services, an overstretched health system, and movement restrictions, all of which have adversely affected both the supply and demand side determinants of the performance.

Aim: This national study, therefore, was aimed at exploring the structure and functions of the different components of the immunization program, including immunization service delivery; vaccine quality, safety, and vaccine preventable diseases surveillance; immunization supply chain management; demand generation and communication; the leadership, management, governance, and human resources; and the monitoring and evaluation of the program.

Methods: The study employed a mixed methods study with concurrent quantitative and qualitative approaches that includes cross-sectional survey of households, health facility and health post assessments, desk reviews, exit interviews, session observation, key informant interviews, and focus group discussions. The study included randomly selected 15,158 children aged 12-35 months and women 15-49 years, 6,900 adolescent girls aged 15-18 years, 14,825 adults from 471 enumeration areas after samples were proportionally allocated to size of regions of Ethiopia. Key informant interviews were carried out with purposively selected officials and EPI experts at at all levels of the health system, and FGDs were conducted with the community. Quantitative data were collected electronically using Open Data Kit (ODK) using face-to-face interviews. Data on vaccination status were obtained from three sources; 1) vaccination cards, 2) vaccination history from verbal responses, and 3) health facility records. The WHO Effective Vaccine Management (EVM) tool was used to collect data on the vaccine supply chain management 302 national and subnational hubs, lower distribution units and service providing institutions. Data were exported into the central server on daily basis, cleaned and analysed using STATA statistical software. Qualitative data were analyzed thematically after verbatim transcription and translation.

Leadership, Management and Governance: Despite an increase in demand for immunization services in recent years, the immunization program structure has not been expanded accordingly. This has resulted in poor functional alignment and coordination between different levels of the program. More than half (53.4%) of woredas' annual plans were not linked with the budget, 5.2% lacked strategic plans, and 3% lacked operational plans. There is inadequate staffing at all levels, with a significant under-representation of personnel trained on immunization in the last two years. This includes health post service providers, where only 57.4% health extension workers were trained on immunization in the last two years preceding the survey. Immunization officers use outdated census reports for population projection and target calculation leading to inaccurate planning and improper resource allocation. Personnel shortages and inadequate cold chain capacity were major bottlenecks, along with inconsistent guidelines, negative community perceptions, and funding issues, hindering the implementation of new vaccines introduction.

Supply Chain Management: The overall Ethiopian EVM-2023 assessment score was 69%, far less than the desired EVM standard of 80%. This study indicated critical gaps in the cold chain system, with a shortage of refrigerated vehicles and a shortage of temperature records at health facilities. Lack of well-equipped maintenance workshops, budget constraints, and shortage of skilled technicians remain significant barriers to ensuring the proper functioning of vaccine cold chain. Despite the improvements in on-demand, proximity-based, last-mile delivery; data visibility, transportation, and infrastructure shortages challenge persist. At lower distribution levels, only 18% of health institutions have VVMs beyond discard time and 16% storing expired vaccine vials.

Service Delivery: A critical gap exists in immunization service availability, with only 38% of facilities offering services every day. To reach zero dose and under-immunized children, campaign was practiced by most health institutions studied. Findings from session observation to assess service quality indicated that only two thirds of the vaccinators practiced proper BCG and measles preparation. Estimates from child immunization coverage revealed that one-in-four eligible children (24%) have not received any vaccine, whereas 44% of children received all vaccines. Two-third (66%) of the studied children received the third dose of Pentavalent, and 60% received the first dose of measles. The dropout rates of Pentavalent was 14.2% and it was 20% for MCV1. Overall, 85% of mothers received at least one dose of Td vaccine, and only 54% the adolescent girls received one dose of HPV vaccine.

Adverse Events Following Immunization (AEFI): The study found that about half (51%) of vaccinated children experienced at least one type of AEFI. Similarly, 44% of people vaccinated for COVID-19 and 20% of adolescent girls vaccinated for HPV reported AEFIs. While the significant majority of the AEFIs were wild cases, a total of 11 children were reported to have been hospitalized because of serious AEFI. Rural residents were found to have limited awareness about vaccine adverse events, and its management. These study also revealed that children who developed adverse events drop out the subsequent vaccination schedules. Regarding AEFI surveillance, a staggering 88% of health facilities did not report a single AEFI case in one year preceding the survey. This suggests significant underreporting of AEFIs, potentially compromising vaccine safety monitoring. AEFI surveillance are not being considered as part of the routine immunization activities in many health facilities particularly at health posts. Weak coordination between stakeholders, AEFI surveillance not integrated with HMIS, absence clear compensation policy, suboptimal detection and reporting system of AEFI, fear of blame to report AFEI, low awareness of the community were among the major challenges identified.

Vaccine Preventable Diseases Surveillance: Despite being a crucial activity for disease prevention, only 35% of health facilities conducted VPD surveillance activities in the year preceding the survey. This potentially hinders early detection and response to vaccine-preventable disease outbreaks. On the other hand, a quarter of health facilities, and half of health posts do not have a focal person to conduct VPD surveillance regularly. A significant gap exists in immediate VPD reporting, with only 56% of health centers & hospitals and 43% of health posts doing so. Inadequate skilled human resource at lower level and shortage of necessary inputs such as lab equipment and capacity building for health workers were the major challenges.

Demand Generation and Communication: While most mothers (97.3%) knew where to get vaccinations, a significantly lower proportion (26%) knew at least one type of childhood vaccine antigen provided in the national immunization program. Open multi vial policy, accessibility and availability of services, and gaps in interpersonal communication skills of providers were impediments to vaccination uptake.

A large majority (85%) of adolescent girls in the study were aware that the HPV vaccine helps prevent cervical cancer. This highlights a positive finding regarding knowledge about a specific vaccine. This study also indicated that only half (50.1%) of respondents have favorable attitude about COVID-19 vaccine. The study revealed significant regional variations in healthcare workers' IPC skills. Participants from several regions, including Oromia, Amhara, and Sidama, demonstrated higher proficiency compared to those in Afar and Gambella regions. Inadequacy and local insensitiveness of communication materials, weakening of community structures, growing perception of demand creation as a finished agenda for routine immunization, and inadequacy of behavioral change enablers were among the major challenges to EPI advocacy, communication and social mobilization.

Monitoring and Evaluation: While the health system has a structure for monitoring and evaluation (M&E), there is a noticeable absence of a specific structure for M&E of EPI. Data entry (HIT) staff are present in most regions, zones, districts, and facilities, but there is a shortage of staff trained in data management at all levels of the system. Independent M&E mechanisms for EPI are present in three quarters of RHBs and 82.4% of zones. The study further highlights varying levels of EPI data use culture across different health system levels, with some regions demonstrating strong practices in data utilization. Active monitoring practices for immunization performance and stock monitoring are observed at regional, zonal, and district levels. Disparities in data completeness and timeliness exist among regions, with some showing higher performance than others.

INTRODUCTION

1. Introduction

Immunization is a vital, cost-effective intervention intended to avert mortality rates among children, adolescent girls, and women. It is known as the most effective public health intervention in preventing infections that are highly associated with high morbidity and mortality (1-3). The vision for the decade of vaccines, outlined in the strategic document of Immunization Agenda 2030, outlines goals and suggestions for enhancing health and leaving no one behind, in any situation or at any stage of life that everyone receives the full advantages of immunization (4).

The World Health Organization (WHO) introduced the Expanded Program for Immunization (EPI) in 1974 to control Vaccine Preventable Diseases (VPDs). With the expansion of vaccination programs across low- and middle-income countries (LMICs) over the past two decades, morbidity and mortality` related to VPDs have shown a significant reduction.

Ethiopia launched EPI with six antigens (BCG, DPT, Measles, OPV) in 1980 with the aim of reducing mortality, morbidity, and disability among children and mothers. The program has substantially reduced deaths and illnesses from VPDs. It has added new vaccines over time and now offers fourteen antigens including HPV and COVID-19 vaccines(1, 5).

The EPI operates within the context of the national health system, in alignment with global, regional and national strategies, where children aged under a year, children in their second year of life, adolescent girls (9- 14 years of age), women of reproductive age group (15- 49 years of age), and adults above 15 years of age are the targets for the currently available vaccines in the immunization program of the country (6). The key approaches to immunization service delivery in Ethiopia include static, outreach, and mobile strategies (1). Currently, the EPI program delivers its services at all service delivery points at public and private ealth facilities, including 471 hospitals, 3,848 health centers, and 17,982 health posts. Moreover, the program operates with 14 national and regional laboratories, and one central and 15 Sub-central pharmaceutical supply hubs.

The national EPI program has received international recognition for some of its remarkable achievements so far. Ethiopia has received certification from WHO and UNICEF for eliminating maternal and neonatal tetanus nationwide in 2017 and for maintaining polio-free status by the WHO AFRO Certification. Committee since August 2020 (7, 8). The country has also made commendable progress in terms of immunization coverage. Between 2000 and 2019, the coverage of DPT3/Penta3 increased from 21.1% to 61% and the proportion of children fully immunized increased from 14.2% to 43%(9, 10).

As a result, Ethiopia has achieved tremendous results in reducing deaths among children over the last two decades, including a 52% decline in under-5 mortality from 123 deaths per 1,000 live births in 2005 to 59 deaths per 1,000 live births in 2019. Over the same period, infant mortality declined from 77 to 47 deaths per 1,000 live births (6, 11).

A well-functioning immunization system depends on the interaction of several components such as careful planning and management of human, financial, and logistics, effective communication strategies, quality service delivery, VPD surveillance, and program-level monitoring and evaluation (M&E). Moreover, policy support, global collaboration, and emergency preparedness are essential for the success of EPI (2, 12, 13). Ethiopia's national immunization program has been improved to be geared with a life course and integration approaches. However, the system suffers from multifaceted problems ranging from policy frameworks from the delivery side to community perception on the receiver end (14-19).

Vaccines are very important and sensitive health commodities that require proper supply chain system, which operates in a network of storage facilities and transportation system designed to

keep vaccines at a recommended temperature range. Around the millennium and earlier, the supply chain management system was reported to be "inefficient and unacceptable", and its distribution was disorderly. For this reason, a number of transformation and transition plans took place, in 2005, when Ethiopian Pharmaceuticals Fund and Supply Agency (EPFSA) was established; Vaccine Supply Chain Management Transition Plan of 2014, helped with relocation of existing cold rooms to PFSA hubs, design and implementation of a Logistic Management Information System (LMIS), development of Standard Operating Procedures (SOPs), and training and recruitment of staff; and development of an overall Immunization Supply Chain Management Strategy, 2018-2023. They also helped to acquire equipment such as Walk-in-cold rooms, Solar Direct Drive (SDD) refrigerators, and refrigerated vehicles. PFSA was created to take responsibility for vaccine availability at national level, while the health offices (FMOH, RHBs and woreda health offices) were to focus on policy, planning, and overseeing service delivery.

National Effective Vaccine Management Assessment (EVMA) was conducted in 2013 and 2019 using the WHO EVM 1.0 tool. The use of data related to supply chain management was facilitated by the deployment of features such as Vaccine Vial Monitors (VVM), and Health Commodity Management Information System (HCMIS). In response to GAVI's full portfolio planning (FPP), Ethiopia developed the five-year immunization Comprehensive Multi-Year Plan (cMYP) 2021-2025, aligned with the HSTP II (2020-2025) and global frameworks. Nevertheless, the sector is highly challenged by so many problems related to equipment, HR development, finance (1).

There are gaps in immunization coverage, quality, and equity due to barriers that are multifaceted and include inadequate leadership, management, and governance skills among health workers and managers (20). The improvement of the national EPI coverage is contrary to the outbreak of different VPDs in different parts of Ethiopia (21-23). In Ethiopia, 19.2% of eligible children have never been vaccinated for the required vaccines (9). The number of children defaulting from the vaccination program is also high (9, 14, 24).

Evidence shows that the national immunization program faces challenges related to health facilities readiness in terms of the availability of essential human and material resources. In addition, the engagement of private health facilities in the delivery of immunization services is very limited (25). Moreover, despite recommendations made in the guideline for immediate reporting of Adverse Events Following Immunization (AEFI), very few health facilities are actually reporting AEFI. A recent study revealed that 88% of the facilities have not reported a single AEFI case in one year preceding the survey (26).

The national immunization plan of action and budget misses demand generation and immunization communication activities at all levels of health system (29). According to evidence generated at sub-national levels, the quality of vaccination services is being neglected due to poor vaccine storage and handling, poor practice of vaccine administration/delivery, poor waste disposal and management, and inadequate knowledge of health care providers (30). Besides, constraints of unreliable electric power supply and inadequate and improperly set up storage facilities were important factors for the poor quality and safety of vaccines (31). Despite documented improvements in the monitoring and evaluation activities, reports showed huge discrepancies in the immunization report generated using different platforms like administrative reports, surveys, and WHO/UNICEF Estimates of National Immunization Coverage (WUENIC)(32-34).

In recent years, the national immunization program has faced challenges from natural and artificial catastrophes such as the COVID-19 pandemic, drought, war, and economic crisis (27, 28), which resulted in shifting government priorities, interruption of services, an overstretched

health system, and movement restrictions, all of which have adversely affected both the supply and demand side determinants of the performance of the national immunization program.

The above evidence implies that there may be a problem with the overall national immunization program implementation at different levels of the health system - national, regional, zonal, district, health facility, provider, and user levels. Further, previous achievements and recognition shall be appraised in the lenses of global landscape and international standards giving more room for improvement and lots of cup to fill. Hence, this study intends to evaluate Ethiopia's immunization program.

The overall objective of the national immunization program evaluation was to explore the structure and functions of the different components of the national immunization program, including immunization service delivery; vaccine quality, safety, and vaccine preventable diseases surveillance; immunization supply chain management; demand generation and communication; and the leadership, management, governance, and human resources; and the monitoring and evaluation of the program. The specific objectives of the evaluation include to:

1. Assess the existing leadership, manage ment, and governance of immunization service delivery systems.
2. Determine service coverage and delivery across all the health care delivery system.
3. Explore the supply chain management practice of the national immunization program.
4. Explore the demand generation and communication strategies and practices of the immunization program
5. Assess the practice of vaccine safety, management of adverse events follow ing immunization, and vaccine preventable diseases surveillance implementation.
6. Assess the monitoring and evaluation strategies and practices of the EPI.

In order to address these objectives, we conducted a national level assessment on the different components and sub-components of a national immunization program. Table 1.1 shows the key thematic areas and the sub-themes assessed.

Table 1.1: Thematic areas of EPI (Adapted from WHO EPI Review Guide)

Thematic areas	Sub-themes
Leadership, management, governance, and HR	Program Management and Financing<br>A. Policy & guidance<br>B. Governance & accountability<br>C. Planning & procurement<br>D. Partner coordination<br>E. Budgeting & financing<br>F. Structural challenges for EPI program at all levels<br>HR Management<br>A. HR planning<br>B. Capacity-building<br>C. Supervision & performance monitoring
Service delivery	A. Facility readiness, including HR and strategies.<br>B. Service delivery strategies (Access)<br>C. Session quality<br>D. Service integration<br>E. Coverage survey (utilization and equity)
Immunization Supply chain management	A. Cold chain<br>B. Supply management (including stock availability)<br>C. Transport<br>D. Waste management
Demand generation and communication	A. Demand<br>B. Advocacy & communication<br>C. Community engagement<br>D. Mapping of Vaccine Hesitance and mitigation strategies
Vaccine quality, safety, and VPD surveillance	Vaccine quality, safety<br>AEFI monitoring<br>VPD surveillance<br>A. HR & systems<br>B. Detection & response<br>C. Performance<br>D. Surveillance data use
Monitoring and evaluation	A. HR & systems<br>B. Recording & reporting<br>C. Data quality<br>D. Coverage monitoring (including the issue of denominators (existing challenge, opportunities , head count practice ...)<br>E. M&E information use

SURVEY METHODS

2. Survey Methods

2.1 Overview

This national immunization program was designed and conducted by a consortium of 5 Ethiopian universities, including Addis Ababa University, Jimma University, Haramaya University, Hawassa University, and University of Gondar. The evaluation process has been participatory. Under the leadership of the Ministry of Health, stakeholders of the national immunization program, including MoH, RHBs, federal and regional agencies, NGOs, and UN agencies participated in the evaluation process. The Immunization Interagency Coordinating Committee (ICC), the National Immunization Technical Advisory Group (NITAG), and the Immunization Technical Working Group (TWG) served as platforms for stakeholder engagement. The stakeholders were engaged in the finalization of evaluation objectives, refining the evaluation methods, guiding data collection processes, and validating key findings. Stakeholders also provided input in the formulation of recommendations. Data for this study were collected from March to July 2023.

2.2 Survey Design

A mixed methods study with concurrent quantitative and qualitative approach was used to evaluate the national immunization program at all levels of the health care system and the community. The study included cross-sectional survey of households, health facility assessments, desk reviews, self administered questionnaire for the health professionals to assess IPC skill key informant interviews, and focus group discussions.

2.3 Study Populations

The study included a nationally representative sample of households, health facilities of all types (health posts, health centers, hospitals, and private clinics), health administration institutions (MoH, RHBs, ZHDs, WoHOs), and federal and regional agencies (public health institutes, FDA, PSS). At the household level, children (12-35 months), women (15-49 years), adolescent girls (15-18 years), and persons 12 years and older were the study populations for data collection on child vaccination, TD, HPV, and COVID vaccine, respectively. In addition to assess interpersonal communication skills health care providers , who were working at immunization units included in the survey. The qualitative study participants were officials at all levels of health system and partners and stakeholders including MoH, federal and regional agencies, development partners and UN agencies, regional health bureaus, zonal health departments, woreda health offices, public and private health service providers, and the community.

2.4 Sample Size and Sampling Strategies

Sampling of Households

Sample size for the household survey was calculated for each of the regions and city administrations. The minimum sample sizes for estimating vaccination coverage for the target antigens (Penta3, zero dose, MCV2, HPV and COVID-19) were calculated using a single population proportion formula. Coverage estimates for each of the regions and city administrations reported in recent studies (mini-EDHS 2019; ICO/IARC Information Centre on HPV and Cancer (HPV Information Centre) were used as approximations of their corresponding population parameters. We assumed 95% confidence level, 2% margin of errors, a design effect of 3, and 10% expected nonresponse rate. Accordingly, the total sample size was 15,158 for child vaccination and women of reproductive age group, 14,825 adults, and 6,900 adolescent girls (Table 2). The calculated sample sizes also meet the minimum requirement suggested by WHO cluster survey guideline (34).

The household survey included all regions and the two city administrations, except Tigray region. Two stage stratified sampling technique was used to select the survey population. In the first stage enumeration areas (EAs) were randomly selected from stratified (urban and rural) EA sampling frames prepared for each region and city administration by the Ethiopian Statistical Services (ESS). The number of EAs required per region and city administration was determined based on the estimated sample size of each region/city administration and with the aim of allocating a maximum of 30 households per EA. The required number of EAs was allocated to urban and rural strata based on the proportion of the national population living in urban and rural areas (21.4% urban and 78.6% rural). EAs were finally selected randomly from each of the two strata under in each of the regions. A total of 505 EAs (127 EAs from urban, and 378 EAs from rural) were included in the survey Table 2.2. The field teams delineated the sampled EAs using EA maps obtained from ESS. Each team prepared sampling frames of households with at least one target population (children, women, adolescent girls, and adult household members). From each EA, 30 households with at least one eligible household member were selected using random sampling. If more than one eligible person was available in the household, one would be selected using simple random sampling. If the selected person was unavailable during data collection time, the data collector would revisit the household at least two times. If the selected individual refused to participate, the data collectors moved to the next selected household.

Table 2.1: Sample size for household survey by region and target population

Region	Estimated Sample Size (Number of study subjects)						Number of EAs		
	Children 12-35 months (Penta3)	Children 12-35 months (Zero-dose)	Children 12-35 months (MCV2)	Women 15-49 (Td)	Adolescent girls >14 years (HPV)	People ≥12 years (COVID-19)	Urban EAs	Rural EAs	Total
Afar	668	620	593	668	482	694	22	5	17
Amhara	2317	1600	1168	2317	696	2,315	77	16	61
Benishangul Gumuz	624	594	577	624	482	621	21	4	17
Gambella	567	560	577	567	482	570	19	4	15
Harari	550	550	552	550	482	552	18	4	14
Oromia	3781	2469	1678	3781	772	3,434	126	27	99
Sidama	897	756	672	897	482	859	30	6	24
SNNP	1630	1191	928	1630	612	1,518	54	12	42
Somali	1013	825	713	1013	482	768	34	7	27
Southwest	796	696	637	796	482	1,027	27	6	21
Tigray	995	814	707	995	482	971	33	7	26
Addis Ababa	748	667	620	748	482	922	25	25	0
Dire Dawa	571	562	559	571	482	576	19	4	15
National	15158	11903	9981	15158	6900	14,825	505	127	378

NB. The number of adolescent girls per region was determined based on the proportion or conversion factor of adolescent girls 14 years and above.

Sampling of Health Facilities

All health posts in the selected EAs and their corresponding catchment health centers were included in the survey. Assuming that the EAs were evenly distributed throughout each region, we anticipated the number of sampled health posts, health centers, and EAs to be equal. Additionally, hospitals, WoHOs, ZHDs, and private hospital and private clinics serving the selected EAs were included as they are part of the chain of EPI referral link even though they may not be physically located within the sampled EAs. From each sample health facility, 3 to 5 exit interviews were conducted to assess client satisfaction and adverse effect following childhood immunization. From each health facility 3-5 health care providers included in the survey to assess IPC skill, and barriers to communication.

Sampling for Qualitative Data Collection for the qualitative study, a total of 76 KIIs and 24 FGDs were planned to be included. The key informants were selected from representatives of stakeholders at national, regional, zonal, woreda, facility, and community levels. The FGD participants were community members. In one FGD, 5-7 participants were included. The sample size was optimal enough to assess the national EPI program and to bring potential information and point of saturation was also used to determine the final samples size of the survey.

Purposive sampling method was used to select key informants, who had direct or indirect involvement in the planning, implementation, monitoring, and evaluation of EPI at administrative and service delivery levels. Participants were identified from all levels of the health care system, community representatives and women development army.

The participants were selected using purposive mixed method sampling using intensity sampling (information rich) for the selection of key-informants regardless of the level of institution and personal position. In addition, maximum variation (one from each group) was used to recruit regions considering Penta 3 vaccination, or MCV2 coverage. The regions were divided into three categories to create maximum variation as indicated in Table 2.3. Whereas, to recruit key informants at zonal and woreda levels, we used extreme case/deviant sampling (two from each group i.e. the highest and lowest coverage of immunization service.

Table 2.2: Number of Key informant interviews and FGDs at national, subnational, facility, and community levels in Ethiopia 2023.

Regions	Federal level	Partners	RHB (EPI focal)	Zone/ wored (EPI focal)	Hospital/ PHCU	Private clinic EPI service provider	HEWs /HP	Community level FGD	Total
Addis Ababa	All (9)	BMGF, CDC	One	2 sub cities (H,L)	None	1 (Providing	Two (H,L)	1 Female & Male community reps	17 KII, 2 FGD
Dire Dawa		None	One	None	1 Hospital (H)	None	Two (H, L)	1 Female & Male Community reps	4 KII, 2FGD
Amhara		JSI	One	1 Waghmi ra zone	1 PHCU (H)	1(Non-providing)	Two (H, L)	1 Female & Male WDA leaders	7 KII, 2 FGD
Benishangul Gumuz		None	One	1 WrHO (I	1 PHCU (L)	None	Two (H,L)	1 Female & Male Community reps	6 KII, 2 FGD
Gambella		None	One	None	1 PHCU (L)	1 (Providing	Two (H,L)	1 Female & Male Community reps	5 KII, 2FGD
Harari		None	One	None	1 hospita (H)	None	Two (H, L)	1 Female & 1 Male WD/ leaders	4 KII, 2 FGD
Oromia		WHO	One	2 Zone (H, L)	None	1 (Non-providing)	Two (H,L)	1 Female & Male Community reps	7 KII, 2 FGD
Sidama		None	One	1 WrHO (I	None	1 (Providing	Two (H, L)	1 Female & Male Community reps	5 KII, 2 FGD
SNNPR		UNICEF /GAVI	One	1 S.Om Zone	1 PHCU (H)	None	Two (H, L)	1 Female & Male WDA leaders	6 KII, 2 FGD
South west		None	One	1 WrHO(L	1 PHCU (L)	None	Two (H, L)	1 Female & Male Community reps	5 KII, 2FGD
Afar		None	One	1 Afar (L)	None	1 (Non-providing)	Two (H,L)	2 WDA leaders	4 KII, 2FGD
Somali		None	One	2 WrHO (H&L)	1 PHCU (H)		Two (H,L)	1 Female & Male Community reps	6 KII, 2 FGD
Total KIIs/FGD	9	5	12	12	8	6	24	24	KII - 71 FGDs 24

H–High performing L–Low performing

2.5 Data Collection Methods and Instruments

For this evaluation, we used questionnaires, desk reviews, observation checklists, and key informant interview guide, and focus groups discussion guide questions were used for data collection.

Household Survey Questionnaire

The quantitative data was collected using face-to-face interviews with women, adolescent girls and persons 12 years or older in sampled households using a household survey questionnaire. The questionnaire included four subsections: household background, routine child immunization (BCG, Pentavalent, PCV, polio, Rota virus, measles and maternal TD2 vaccination), HPV, and COVID-19 vaccination related questions. The questionnaire was adapted from different guidelines and tools for assessing the performance of EPI from different organizations, including WHO, MEASURE evaluation, PRISM, and UNICEF. Adaptations were primarily intended at tailoring data collection tools to the national EPI guideline and previously conducted national assessments involving EPI. The questionnaire was translated into five local languages (Afar, Amharic, Afan Oromo, Sidama, and Somalia).

Effective Vaccine Management (EVM) System

The WHO Effective Vaccine Management (EVM) tool was used to collect data on the vaccine supply chain management system. The EVM system used nine thematic areas known as "criteria score" (Es), and has six entities, known as "Category Scores" and denoted by "C". There are four supply chain levels for assessment used by the system: Primary level (PR) (one), where the vaccine arrives after importation (national hub); the Sub-national Level (SN), the EPSS hubs (15); the Lower Distribution level (LD), Woreda Health Offices (90); and the Service provision level (SP), the health facilities (83 HPs, 101 HCs, and 12 Hospitals). There are about 400 indicators under PR, about 300 under SN, about 200 under LD and about 150 under SP, in total about 1050 indicators. The scoring indicators are grouped into 3 and measure availability, quality, and efficiency. The scoring begins from the bottom, scoring the indicators, then HFs, finally to the PR level, combining the scores at each level, arriving at aggregated national level score.

Vaccination Status Survey

Data on vaccination status of the sampled children were obtained from three sources, including 1) written vaccination records (including the vaccination card and other health cards), 2) vaccination history from verbal responses of mother/care givers, and 3) health facility records (vaccination registration books). The health facility vaccination registration book review was used to complete and verify vaccination history reported by mothers/care givers of children for whom vaccination cards were not available, but a health facility visits, or receipt of any vaccine was reported.

Data on vaccination coverage were obtained from health facility records in addition to information written on vaccination records, including the infant immunization card and other health cards, and information gathered from mothers' verbal reports. During the individual interview, mothers were asked to report vaccinations received by their children born in the last 3 years. For each child born in the 3 years before the survey, mothers were asked to show the interviewer the infant immunization card or health card used to record the child's immunizations. If the infant immunizations card or other health card was available, the interviewer copied the dates of each vaccination received in the respective section of the Questionnaire. If a vaccination was not recorded on the infant immunizations card or the health card, the mother was asked to recall whether that vaccination had been administered.

If the mother was not able to present the child's infant immunizations card, she was asked to recall whether the child had received the BCG, polio, DPT-HepBHib, measles, pneumococcal, and rotavirus vaccines. If she indicated that the child had received the polio, DPT-HepB-Hib, pneumococcal, measles, or rotavirus vaccine, she was asked the number of doses that the child received. If the mother did not have the infant immunizations card available and the child had visited a health facility, the field supervisor or the health facility expert went to the health facility to collect the relevant vaccination records. The purpose of obtaining information at the health facility was to complement the mother's report.

When the mother/caretaker did not present the immunization card and when they are asked to recall the vaccines the child has taken, the site of the injection and the date (schedule) of the vaccination were asked to reduce recall bias. For instance, for BCG vaccine a scar on the left upper arm were looked for; for Penta vaccine the mothers were asked if the child was vaccinated on his/her left outer thigh together with the date of the vaccination; similarly for PCV – right thigh; Rota – oral drops; polio vaccine – oral droplets; measles – upper left arm; HPV – left upper arm among adolescent girls were inquired.

Document Reviews

Administrative units and facilities were assessed using document/record reviews. The survey included health facility assessment where health facility experts reviewed the health facility for EPI program related questions. Separate checklists were developed and used to assess (1) regional Health bureaus/zonal health departments, (2) Woreda/Sub-city (3) hospital and health center, and (4) health posts.

Vaccination Session Observation and Client Exit Interview

Session observations were conducted at hospitals, health centers, and health posts during both static and outreach sessions. Five session observations were held in each health center and hospital, and three session observations were held in each health post. The health facility assessment experts observed service providers on vaccination safety, communication skills, and waste disposal practices. Exit interviews were conducted with mothers and care takers coming to health facilities and health posts to vaccinate their children. The exit interview questionnaires primarily focused on satisfaction with the service, and AEFI experience.-Self administered IPC tool was used to assess IPC skill of health workers and barriers affecting communication

FGD/Key Informant Interview Guides

Key informant interviews were conducted to evaluate the EPI all levels of the health system including, ministry of health, regional health bureaus, EPSS hubw, woreda health offices, non-governmental organizations, Ethiopian public health institute, EFDA, health centers, health posts, and private health facilities. Focus group discussions were held with informed community representatives.

The interview questions were mainly semi-structured, with some open-ended questions for further probing of responses. Supportive documents and observation of important sources of evidence (like temperature reading, record keeping, and meeting minutes) were made to ensure credibility of the data. All interviews were conducted in a noise-free environment at their offices using local languages and audio recorded while scribbles were maintained during the interviews. All the scribbles were expanded to field notes to catch unspoken parts of the interview and other emotional reactions of the participants. All audio-recorded data were transcribed and translated for analysis. Experienced qualitative survey assistants were used to lead the interviews. One experienced moderator, who can speak and listen to the local language and a note-taker were recruited to

facilitate FGDs at community level. In one FGD, 5-7 participants were included. The rigor of the qualitative data and its findings were assured by applying different approaches following Lincoln's and Guba's four criteria namely credibility, dependability, conformability, and transferability. We purposively selected information rich participants. Data were collected from all over the country to assure data triangulation by person and place. In addition, there were debriefing sessions every day during data collection. Efforts were made to ensure consistency between the data before and after translation by back translation of samples of files for each language. We involved at least three different data collectors and two independent coders to read three selected transcripts and identify categories as an effort to ensure the dependability of the findings. Data reflecting common views of FGD participants were quoted verbatim with references to the sources of the excerpts extracted from the data to assure conformability. We utilized thick description by providing adequate details on the survey site, the participants and recruitment, and data collection and analysis in the methods section to assure transferability.

2.6 Recruitment and Training of Field Staff

For the household survey and facility assessment, data collectors were recruited based on their experience on data collection, willingness to work in an assigned area, availability for the training period, and fluency in writing and speaking local languages of the survey area. A total of 468 data collectors (having a minimum of BSc degree), 115 facility level data collectors and 117 supervisors (having a minimum of MPH/MSc degree) were recruited for the household and facility assessments. Additionally, one field/local guide was assigned for every five EAs.

All household and health facility assessment data collectors and supervisors were trained for seven days on data collection tools and procedures before deployment. The training materials contained the six thematic areas of the EPI evaluation study, specific contents, administration of the interviews, supervision, communication and ethics, data confidentiality and participant privacy. The training also covered the application of electronic data collection methods, including ODK and EVM system.

For qualitative data collection, the facilitators and note takers had MPH/MSc degree and above in health sciences disciplines, had a good experience in qualitative data collection translation and transcription, and were willing to work in an assigned area and attend training. A total of 18 data collection facilitators and 9 field note-takers were recruited for the same. All qualitative data collectors and note takers were trained for seven days on the qualitative data collection tools and procedures.

2.7 Field Work

The field work activities had two phases: preparation and implementation phases. The preparation phase included mapping of resources, recruitment of data collectors, supervisors, health facility assessment experts, local guides, ODK tool development, training of data collectors and supervisors, conducting pretests, division of tasks among the survey team and field workers, and assigning of field workers to the data collection sites/facilities, identifying enumeration areas, delineation, household listing, sampling, and obtaining consent/assent. The implementation phase was the actual data collection and supervision. At the household level, data were collected through face-to-face interviews with eligible members of each sampled household. Health facilities and offices desk review, vaccination session observation, client exit interview, key informant interview and FGD were the other methods.

2.8 Pretest

Pre-testing was carried out outside the sampled woredas, where the community had similar

characteristics. Pre-testing of the questionnaire and survey implementation procedures were conducted in five selected kebeles from five regions. These kebeles were not included in the study. Around five questionnaires were completed in each pretesting kebele to check consistency and validity of the questions asked. The pretest was used to check clarity of each question, identify ambiguous questions, check skipping patterns and the flow of questions, and check the consistency and cultural sensitivity of questions. Comments and suggestions from the pretest were incorporated in the final data collection tools.

2.9 Data Management and Analysis

Quantitative Data Management and Analysis

Data were exported from the data collection sites into the central ODK server on daily basis. Descriptive statistics like frequency, percentage, median, mean, and standard deviation were computed using STATA software version 16. Additionally, maps, graphs, and tables were used to present the findings of the survey. All data presented in the results section are weighted values unless specified.

The EVM data was analyzed using two approaches: results obtained directly from the report generated by EVM system, and analysis of the EVM-Excel imported raw data. For the second approach, the raw data were exported to excel from EVM system which constituted about 1350 indicators. The questionnaire for the IPC section consisted of participants' profile; EPI focused training and supportive supervision; effective IPC practice in terms of IPC process, IPC skills, and other IPC activities; and barriers to practicing good IPC practices.

Qualitative Data Management and Analysis

The audio-recorded and field-noted data were checked for accuracy and completeness. Qualitative data were transcribed and translated verbatim from the local languages into English. The qualitative data was analyzed using the ATLAS.ti software and the contents were grouped into categories and then analyzed thematically to identify major themes. The first step was reading and re-reading of the transcripts and field notes before the coding and analysis. Then, guided by the objective of the survey, a codebook was prepared after coding two key informant interviews and one focus group discussion transcripts and reviewed by the qualitative study team before starting the coding. We applied thematic analysis.

The data-analysis process followed a sequence of interrelated steps, such as: frequent reading-coding grouping into categories displayingreducing and interpreting line by line. Inconsistencies of data were clarified by rechecking field records and reducing non-standard data and reviewing themes defining and reading the transcriptions.

2.10 Ethical Consideration

Ethical approval was obtained from the respective Institutional Review Board (IRB). Support letters were obtained from the Ministry of Health and Regional Health Bureaus after being notified of the purpose of the study. Official letters of support were submitted to zonal health departments, woreda health offices, and local administrators. Survey participants were given adequate information about the survey including the purpose of the study, benefits, risks, the nature of confidentiality of the study, and their right not to participate or withdraw at any time from the study. Participant consent/assent permission was obtained for participation and for audio records. Privacy during data collection time and confidentiality of the data was strictly maintained. The data was collected based on anonymously prepared electronic data collection devices. Furthermore, the team of researchers kept the principles of the Helsinki declaration.

LEADERSHIP, MANAGEMENT AND GOVERNANCE

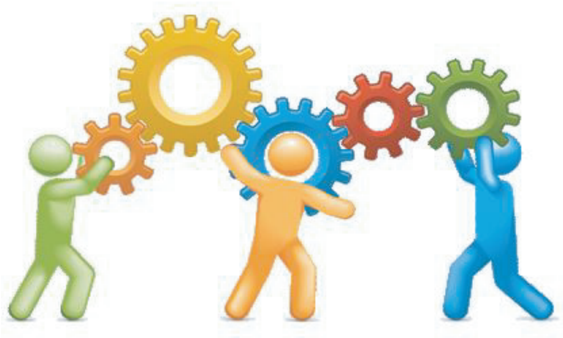

3. Leadership, Management and Governance

This section presents Leadership, Management, and Governance (LMG) current practices, challenges, and opportunities of immunization programs in Ethiopia, including LMG practices, immunization structure, resource mobilization and sustainability of financing, micro-planning process, human resource, new vaccine introduction, and coordination and collaboration among various sectors.

Immunization is a crucial component of public health, and its governance is essential for ensuring that vaccines are available, accessible, and effectively administered to all populations, particularly those who are most vulnerable. Good governance in immunization enables the government to fulfill its responsibility to protect public health while also promoting collaboration and coordination among different stakeholders.

Ethiopia's health policy is organized according to decentralized principles. The three guiding principles of strategic planning in the health industry are "one plan, one budget, and one report. Each year, top-down and bottom-up methods are used to produce woreda-based planning for all health initiatives. These plans include immunization, which is derived from the cyclical five-year plan. Every year, each woreda creates a comprehensive immunization micro plan based on the health care plan of the woreda. Precise population estimates and maps displaying HFs, village locations, and target populations are necessary for efficient vaccination micro-planning (MoH, 2022).

The data collected from health posts, health centers, hospitals, woreda health offices, zonal health departments, regional bureaus, and MoH were assessed using quantitative and qualitative methods.

Key Findings
• Despite increasing demands the immunization program structure has not grown accordingly resulting in poor functional alignment and coordination.<br>• 53.4% of Woredas' annual plans were not linked with the budget, 5.2% lacked strategic plans, and 3% lacked operational plans.<br>• There is inadequate staffing at all levels, with only 57.4% of health post service-providers trained on immunization in the last two years.<br>• Urban HCPs have a higher LMG training rate (43.8%) compared to rural areas (27.2%).<br>• Immunization officers use outdated census reports for population projection and target calculation leading to inaccurate planning and improper resource allocation.<br>• Only 44% of the health posts had updated micro plans.<br>• Coordination of immunization program between the public and private facilities is low at woreda and facility levels with 22.9% and 9.6% respectively.<br>• Insufficient funding, compounded by contextual factors like insecurity and transport limitations, hampers the execution of planned immunization activities.<br>• Implementation of new vaccines is hindered by inconsistent guidelines, negative community perceptions, funding issues, personnel shortages, and inadequate cold chain capacity.

3.1 Planning, governance, and coordination

In the context of the Immunization program, planning and governance involve setting vaccination targets, developing strategies to reach underserved populations, and establishing frameworks to ensure accountability and transparency. Effective planning and governance coordinate efforts, optimize resource allocation, and ensure the integrity and success of immunization programs.

Current Practice

In this study, it is reported that the Immunization Service executive office is responsible for vaccine-preventable diseases. The office is involved in planning, coordination, and M&E of the immunization program. The office also introduces and sustains new vaccines, and communication, ensures vaccine safety and compliance with national standards, and monitors the cold chain system from the global to the local level.

> *"Three working teams operate under a technical group: scientific, immunization logistics and supply, and social and communication. These teams, along with a task force of independent senior experts oversee the national immunization program. Additional support comes from academics, EPHI, AHARI, Partners, FDA, and EPSS. An inter-country coordination …connects the immunization program with the Global Vaccine Alliance."* (KII, MoH)

The regional and zonal health departments allocate resources (budgets, personnel, equipment) for immunization activities within their zone/region. The immunization focal is responsible for the management, leadership, and coordination of all immunization activities. They provide technical support and supervision to Woreda Health Offices and health facilities within their zone/region. They also horizontally integrate immunization with other health programs to maximize efficiency and resources.

> "…I coordinate all immunization activities; conduct supportive supervision, and collect reports from the woreda and report to the region." (KII, Zonal EPI focal)

The woreda health offices prepare immunization Micro planning by adapting regional immunization plans to local needs and resources. Track coverage, outbreaks, and program performance, they conduct integrated supportive supervision for the health facilities under their woreda. The immunization or MCH focal is responsible for managing and coordinating all immunization activities.

At the health facilities level the immunization management, leadership, and coordination are done by the immunization focal person. They administer recommended vaccines at specific ages and intervals as outlined by the national program. Follow-up vaccine storage and handling; data collection and reporting. They manage allocated vaccines, supplies, and equipment within the facility. In collaboration with HEWs identify unvaccinated children, support outreach campaigns, and promote social mobilization for immunization.

Planning

The finding of this evaluation shows that all regions have strategic, operational plan/annual plans for immunization and reported engagement of stakeholders on immunization strategic and operational planning development and joint meetings to monitor the implementation of plans. Regarding the availability of updated population demographic characteristics for planning none reported the availability of updated data.

Regarding the zonal health departments assessed nationally, 90.2% have a strategic plan, 94.1% have an immunization-annual plan, 70.6% reported stake holders were engaged in the development of planning, 43.9% reported having a program budget for immunization, and 78.4% had a joint meeting with stakeholders to monitor implementation of plans.

The finding shows national availability of strategic plans in Woredas was 94.8%, whereas the presence of immunization-specific annual plans ranged from 50% in Harari to 100 % in Addis Ababa. On the other hand the availability of national immunization guidelines ranged from zero percent in Afar and Benishangul Gumuz to 100% in Addis Ababa, Gambella, Sidama, SNNP and Southwest regions. Regarding engagement of stakeholder engagement in planning and monitoring through joint meetings at the national for the woredas is 63.9% and 100%. Linkage of the immunization plan with the financial plan was reported by 53.4% nationally, with Addis Ababa having the lowest proportion (18.2%) (Table 3.1).

Table 3.1: Availability of strategic plan, operational plan, updated population demographics, micro plan, stakeholder engagement, and joint meeting at Woreda level in Ethiopian health care system by region, 2023

Region	Strategic planning	Vision and mission	Operational plan	Population demographics	Micro plan implementation	immunization plan linked to the financial plan	Engagement of stakeholders in planning
National	94.8	94.8	97	94.8	81.7	53.4	63.9
Addis Ababa	92.3	92.3	100	84.6	100	18.1	84.6
Afar	92.8	92.9	92.8	100	92.8	76.9	71.4
Amhara	92.8	92.9	95.2	97.6	92.8	55.2	69
Benishangul Gumuz	75	75	87.5	87.5	100	62.5	87.5
Dire Dawa	NA	NA	NA	NA	NA	NA	NA
Gambella	100	100	100	100	100	50	100
Harari	100	100	50	100	50	33.3	100
Oromia	100	100	98.4	98.4	62.5	55.7	50
SNNP	100	100	100	90.3	80.6	50	74.2
Sidama	100	100	100	93.7	87.5	73.3	75
Somali	82.4	82.6	91.3	95.6	82.6	45.4	39.1
Southwest Ethiopia Peoples	92.8	92.8	100	85.7	50	41.7	57.1

Regarding planning in the health facilities, nationally 68.1% have a strategic plan, and 95% have immunization specific annual plan for immunization. Regarding a joint meeting with stakeholders to monitor the implementation of the immunization plan only 60.7% of the facilities reported having the meeting in the 2015 fiscal year. Regarding the location of facilities 61.4%, 98%, 97%, 85.3%, and 55.5% of the facilities in rural areas had a strategic plan, Immunization specific annual plan, updated population demographics for the catchment, micro plan, and joint meeting respectively. Regarding the facility type in terms of their planning performances public facilities have a higher proportion of Micro planning and Updated population characteristics 81.8 and 94.3%, whereas NGOs/faith-based facilities have a higher proportion of having strategic and annual plans (Table 3.2).

Table 3.2: Availability of strategic plan, operational plan, updated population demographics, micro plan, joint meeting at Facility level in Ethiopian health care system by region, 2023

	Strategic planning	Mission and vision	Operational plan	Updated population profile	Micro plan	Joint meeting
National	68.1	68.1	95.1	93.1	80.1	60.7
Region						
Addis Ababa	89.5	89.5	100	94.7	73.6	42.1
Afar	55.6	55.6	88.9	94.4	83.3	77.7
Amhara	70.7	70.6	94.6	93.3	84	49.3
Benishangul Gumuz	56.3	56.2	87.5	87.5	62.5	50
Dire Dawa	35.7	35.7	92.8	71.4	50	78.5
Gambella	70	70	100	90	100	70
Harari	100	100	100	90	50	50
Oromia	74.7	74.7	98	99	89.9	74.7
SNNP	69.2	69.2	100	94.2	78.8	71.1
Sidama	79.1	69.1	91.7	100	70.8	33.3
Somali	44.4	44.4	80.5	80.5	80.5	50
South West Ethiopia Peoples	59.1	59.1	95.4	95.4	90.9	59.1
Location						
Urban	73.7	73.7	93.3	90.2	77.7	50.4
Rural	61.4	61.4	98	97	85.3	55.5
Public Facility Type						
Referral/Specialized hospital	85.7	85.7	100	85.7	71.4	85.7
General Hospital	54.1	54.1	95.8	75	58.3	54.1
Primary hospital	80.5	80.5	90.2	82.9	68.9	51.2
Health Center	60.5	60.5	96.1	88	44	62.4

Governance and accountability

The national availability of the governing body in woreda health offices assessed in this study was 90.9%, while the availability of national immunization guideline ranged from 0% in Afar and Benishangul Gumuz to 100% in Addis Ababa, Gambella, and Oromia regions. A rewarding system for immunization performance was reported by 35.2% of woredas nationally, with the lowest proportion in Addis Ababa and Harari regions. At national level, certification (28.9%) was the most common form of reward while the least common reward was transfers (10.6%). Refrainment systems were reported by 33.5% of woredas nationally, with the highest proportion (100.0%) in the Benishangul Gumuz region.

Regarding assessed health facilities, nationally, 89.1% of health facilities reported having a governing body, while 80.2% had a national immunization guideline. Job aids for immunization were available in 73.6% of health facilities, and a rewarding system for immunization performance was reported by 40.4% of them. Refraining systems for poor performance were reported by 42.4% of health facilities. There were significant variations in the availability of these items across regions, with some regions reporting much higher or lower percentages than the national total (Table 3.3).

Table 3.3: Availability of governing bodies, national immunization guideline, technical immunization guidelines, rewarding system, and refraining at Woreda and health facility level in Ethiopia by region, 2023.

By woreda	Governing bodies	National immunization guideline	Technical immunization guidelines	Rewarding system	Refraining system
National	90.9	76.2	86.7	35.2	33.5
Addis Ababa	92.3	100.0	100.0	23.0	15.4
Afar	100.0	0.0	92.9	42.8	35.7
Amhara	88.1	60.0	83.3	40.5	40.5
Benishangul Gumuz	87.5	0.0	100.0	75.0	100.0
Gambella	100.0	100.0	100.0	75.0	75.0
Harari	100.0	75.0	75.0	0.0	0.0
Oromia	89.0	100.0	84.3	21.9	20.3
SNNP	100.0	88.1	87.1	45.2	42.0
Sidama	100.0	87.5	87.5	25.0	18.7
Somali	73.9	83.3	91.3	34.8	30.4
SW Ethiopia Peoples	92.9	71.5	71.4	50.0	50.0
By health facility	Governing bodies	Nationa l immunization guideline	Job aids for immunization	Rewarding system	Refraining system
National	89.1	80.2	73.6	40.4	42.4
Addis Ababa	63.2	100.0	73.7	63.2	42.1
Afar	77.8	72.2	61.1	16.7	44.4
Amhara	97.3	80.0	73.3	33.3	37.3
Benishangul Gumuz	100.0	56.2	81.3	6.3	18.8
Dire Dawa	92.9	100.0	64.3	64.3	78.6
Gambella	100.0	88.9	100.0	22.2	66.7
Harari	100.0	100.0	100.0	90.0	80.0
Oromia	94.9	81.8	82.8	42.4	55.8
SNNP	79.2	69.2	51.9	42.4	41.7
Sidama	92.3	91.7	75.0	58.3	55.8
Somali	66.7	80.6	72.2	36.1	36.1
So uthwest Ethiopia Peoples	57.0	68.2	72.7	36.4	31.8

Table 3.4 shows the percentage of health posts that reported the availability of guidelines and LMG practices in different regions of Ethiopia. Job aids for immunization were available in 56.6% of health posts nationally, with significant variations across regions. Afar had the highest availability at 81.8%, while Somali had the lowest at 30.8%. Rewarding systems were present in 28.4% of health posts nationally while refraining systems were found in 13.8% of health posts nationwide, with three regions reporting zero and six more reporting more than 27.3% availability. Belief in the adequacy of HEWs numbers varied widely across regions, with the national total being 40.0%. Gambella had the highest belief in the adequacy of HEWs numbers at 88.9%, while Oromia had the lowest at 23.5%.

Table 3.4: Availability of job aids, rewarding system, new vaccine introducing guideline, HR adequacy and measures to poor performer at health facility level in Ethiopia by region, 2023

Region	Job aids for immunization	Rewarding system	Refraining system	Believes HEWs number adequate
National	56.6	28.4	13.8	40.0%
Afar	81.8	27.2	27.3	36.4
Amhara	59.6	31.9	6.4	44.7
Benishangul Gumuz	33.3	0.0	0.0	50.0
Dire Dawa	75.0	12.5	12.5	62.5
Gambella	88.9	11.1	0.0	88.9
Harari	76.5	29.4	47.1	62.5
Oromia	61.8	39.7	17.6	23.5
SNNP	37.5	34.3	9.4	25.0
Sidama	45.0	35.0	25.0	50.0
Somali	30.8	11.5	0.0	50.0
Southwest Ethiopia Peoples	70.6	17.6	11.8	31.2

It is worth noting that of all assessed facilities 83.0% had governing bodies meet every quarter and 72.4% reported that they conducted a self-assessment of facilities' performance in the last six months while only 30.0% from Gambella and 41.7% from Addis Ababa conducted the assessment, being the lowest.

The findings of the study on the governance practices show Addis Ababa demonstrated about 40% having three and above of them while 50.0% in Afar, Benishangul-Gumuz, and Gambella practice only one of them likewise 56.5% from Somali region practice only one of them. In the overall response, a little more than one-fifth percent of them mentioned participatory budgeting and community score cards as a governance practice while community radio was the least (3.3%) (Figure 3.1).

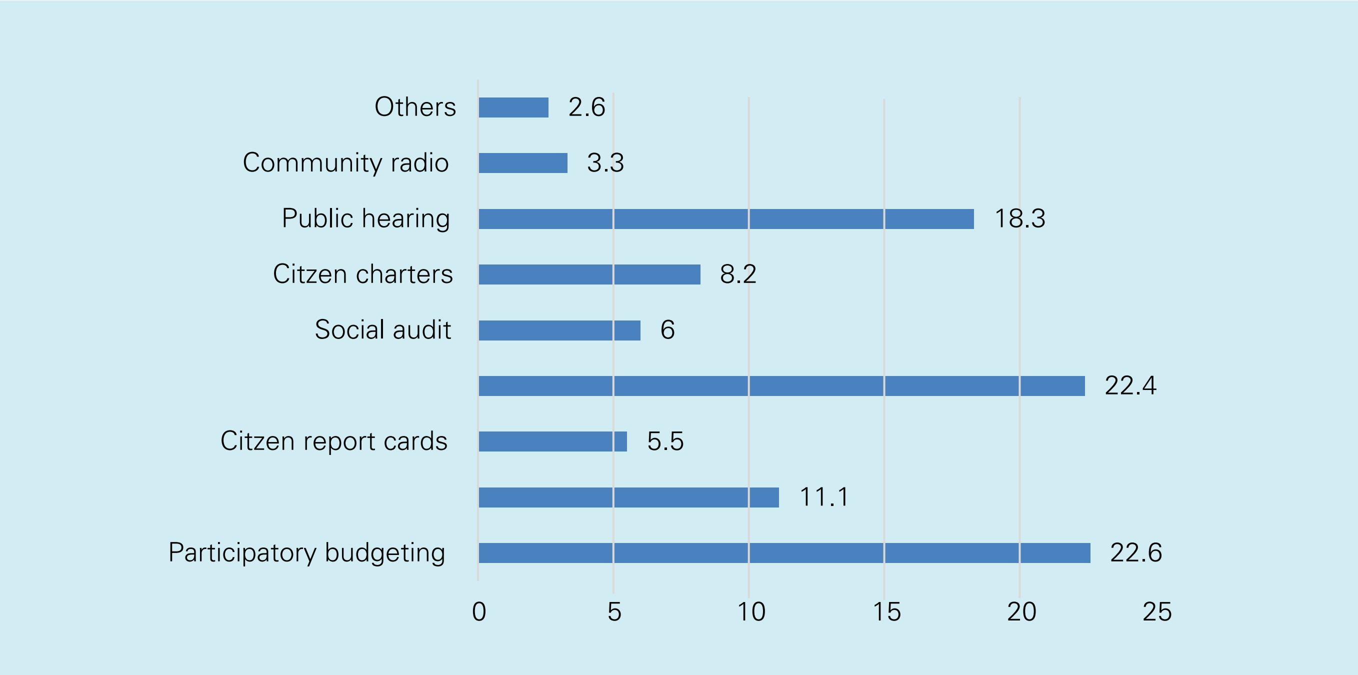

Figure 3.1: Percentage of governance practices in woreda health office of Ethiopia, 2023

Coordination and Collaboration

In Ethiopia at the national level, the coordination platforms include the Joint Steering Committee (JSC), The Executive Committee (EC) The Joint Core Coordinating Committee (JCCC) these platforms support the health sector by regularly reviewing and monitoring the performance against set targets and make timely decisions.

The other platform for collaboration is the National Immunization Task Force, which reports to the Inter-agency Coordinating Committee's technical arm and must provide briefings and written reports regularly (ICC).

The ministry has also ensured their participation in planning, review meetings, supportive supervision, and other health-related activities by collaborating closely with the private sector, professional associations, and Civil Society Organizations (CSOs). Additionally, it is essential to improve the accountability system to increase community ownership and engagement.

All regions and 72.5% of the zonal health offices have a multi-sectorial (governmental) coordination for the immunization program. All regions except Benishangul Gumuz, Somali, SNNPR, and Southwest Ethiopia, and 72.5% of the zonal health offices have a coordination mechanism of immunization program between the public and private facilities. All regions have a coordination between the immunization program and other health care initiatives.

Nationally at the woreda level the availability of governmental sectoral collaboration in the implementation of the activities in the immunization program was 72.9%, with Sidama having the highest

proportion (93.7%) and Harari and Afar having the lowest proportion of 50%. Regarding public-private collaboration, the national is 22.9 % with Gambella the lowest (0%) and Addis Ababa the highest (84.6%). On the other hand, 84.4% of the woredas national have reported having a coordination mechanism to link the immunization with other health care programs. In this regard, Harari has the highest proportion 100% and Gambella has the lowest 50% (Table 3.5).

Table 3.5: Level of Multi-sectoral collaboration, public-private coordination, coordinating immunization with other health care programs and joint meetings for coordination of at Woreda level in Ethiopian health care system by region, 2023.

Region	Multi-sectoral collaboration	Public-private collaboration	immunization with other health care programs	Joint meeting for coordination with stakeholders/partners
National	72.9	22.9	84.4	65.7
Addis Ababa	92.3	84.6	92.3	100
Afar	50	50	92.8	78.5
Amhara	64.3	9.8	90.2	69
Benishangul Gumuz	87.5	12.5	87.5	75
Dire Dawa	NA	NA	NA	NA
Gambella	75	0	50	100
Harari	50	25	100	100
Oromia	79.4	25.4	92.06	70.3
SNNP	74.2	19.4	80.6	61.3
Sidama	93.7	18.7	75	68.7
Somali	52.2	8.7	60.8	47.8
South West Ethiopia Peoples	78.5	14.3	78.5	64.3

The assessment at the level of health facilities shows that the national proportion for multi-sectoral (governmental) collaboration in immunization functions is 63% where Harari had the highest proportion (90%) and Gambella the lowest (20%). On the other hand, facility-level public-private coordination for immunization activities is only 9.6 % with Benishangul Gumuz reporting the lowest 0%. The presence of platforms for coordination with NGOs and the community for immunization activities was reported as 25.8% and 51.6% nationally.

Regarding the coordination of activities in locations of facilities public-private partnership was relatively higher in rural facilities (16.28%) compared to their urban counterparts (6.43%). Similarly, platforms were present in higher proportion in rural areas (56.14%) compared to the facilities in urban areas (48.21%). Except for coordination platforms with the community where private facilities have no coordination (0%), public facilities have lower coordination with stakeholders, NGOs, and private facilities (Table 3.6).

Table 3.6: Level of Multi-sectoral collaboration, public-private coordination, coordination with NGO and the community at facility level in Ethiopian health care system by region, 2023.

Region	Multi-sectoral collaboration	Public -private coordination	Coordination between the facility and NGO	Coordination between facility and community
National	63	9.6	25.8	51.6
Addis Ababa	57.9	15.7	15.8	31.5
Afar	50	27.8	50	27.7
Amhara	60	4	20	45.33
Benishangul Gumuz	43.7	0	37.5	37.5
Dire Dawa	71.4	14.3	7.2	35.7
Gambella	20	20	20	50
Harari	90	20	10	70
Oromia	75.7	13.1	33.3	59.6
SNNP	65.4	4.2	17.3	67.3
Sidama	58.3	9.6	4.1	54.1
Somali	58.3	2.8	52.78	47.2
South West Ethiopia Peoples	54.5	4.5	13.64	54.5
Location				
Urban	61.9	6.4	27.6	48.2
Rural	65.9	16.2	23.4	56.1
Type of public facility				
Referral/Specialized hospital	57.2	14.3	28.6	14.3
General Hospital	54.2	8.3	20.8	29.1
Primary hospital	70.7	7.3	26.8	39
Health center	83.8	8.9	25.5	56.7

New initiatives to improve the immunization program

All the regions except Dire Dawa and the southwest region reported that new initiatives/ projects were launched and implemented at the regional/ zonal/ city administration level to improve the immunization program performance/ coverage in the zone whereas only half 54.9% of the zones assessed reported the launch of new initiatives. Catch-up strategy, mobile health team, digitalization of supportive supervision, and standardization of private facility services are among the common initiatives used to improve immunization program performance/ coverage, PIRI, internally displaced people-focused immunization and intensive immunization programs are the commonly employed initiatives across regions and zones.

Challenges in planning, governance, and coordination

The qualitative findings reported that immunization guidelines are widely available, and are being updated frequently. However, there is no efficient delivery of it to a lower level. A key informant from the Woreda health office mentioned that it is not about guideline availability but rather its delivery that in the end leads to a gap in implementation.

"Regarding immunization-related guidelines at the Federal Ministry of Health, there are updated guidelines. However, the problem lies in the fact that these guidelines do not reach the lower levels…currently, there is a lack of efficient delivery of guidelines from the Federal Ministry of Health to the lower levels…leading to ineffective implementation." (KII, WrHO immunization focal)

The qualitative findings show insecurity, internal displacement, poor communication between leaders at different levels, poor reporting and record management, and lack of attention to the immunization program by the management team at the facilities are some of the challenges at woreda and health facility level besides the common challenges at all levels.

Lack of commitment to immunization program leadership skills and weak collaboration with sectors are some of the challenges at woreda and facilities.

"There is lack of attention for the immunization program by the management, Weak communication between woreda and facilities, and limited intersectoral collaboration." (KII, HC focal).

3.2 Human resource

Human resources in the Expanded Program on Immunization (immunization) are critical for its success. This involves recruiting, training, and retaining skilled healthcare workers who can effectively deliver vaccines, manage logistics, and educate communities. Adequate human resources ensure efficient program execution, high vaccination coverage, and the ability to respond to immunization challenges and outbreaks.

Current Practice and identified challenges

Shows data on various health facilities and their performance indicators across different regions in Ethiopia. The table includes data on the mean number of immunization days lost due to National Immunization Days (NIDs), training on immunization in the last two years, percentage of healthcare providers (HCPs) working in immunization units, and interruptions or missing planned immunization sessions among others. The region with the highest percentage of health posts receiving training in the last two years was Gambella (88.9%), least was Sidama followed by Amhara (20.0%). Harari and Afar had the highest percentage of interrupted or missed planned immunization sessions (50.0%), and lowest by Addis Ababa (5.3%). Urban areas had a higher percentage of HCPs trained on LMG compared to rural areas (43.8% vs 27.2%) (Table 3.7).

Table 3.7: Immunization days lost, training on EP and LMG, supervision, and interruption of immunization service in either HP or other health facilities in Ethiopia by region and location, 2023

	Health Posts			Health Facilities		
	Mean (SD) immunization days lost due to NIDs	Training on immunization in the last two years n (%)	HP receive supervision in the last six months %	Mean(SD) HCPs working in immunization Unit	% of HCPs trained on LMG	Interruptions/missing of the planned immunization sessions %
National	9.9 (16.4)	57.4	76.9	2.0(1.3)	43.5	25.3
Addis Ababa	--	--	--	3.4 (1.3)	52.6	5.3
Afar	5.64 (9.4)	7 (63.6)	72.3	1.9 (0.9)	66.7	50.0
Amhara	6.3 (11.7)	28 (59.6)	85.1	1.6 (0.8)	28.0	30.7
Benishangul Gumuz	15.2 (13.0)	4 (33.3)	75.0	1.9 (1.1)	50.0	12.5
Dire Dawa	4.6 (10.4)	4 (50.0)	87.5	2.1 (1.8)	42.9	7.1
Gambella	18.8 (20.8)	8 (88.9)	77.8	2.5 (0.8)	70.0	40.0
Harari	7.9 (5.9)	13 (81.3)	100.0	4.3 (2.3)	30.0	50.0
Oromia	14.2 (23.5)	49 (72.1)	73.5	1.8 (0.8)	41.4	26.3
Sidama	5.6 (10.7)	4 (20.0)	75.0	2.2 (1.0)	45.8	20.8
SNNPR	9.5 (18.4)	11 (34.4)	85.0	2.1 (1.3)	51.9	25.0
Somali	8.5 (8.9)	14 (53.8)	57.7	2.5 (2.3)	47.2	13.9
SW Ethiopia Peoples	9.6 (11.8)	10 (62.5)	68.7	1.5 (1.0)	40.9	27.3
Location						
Urban	9.9 (11.5)	9 (37.5)	54.1	2.3 (1.4)	43.8	27.2
Rural	9.9 (16.9)	143 (59.3)	79.2	1.8 (1.2)	43.3	22.8

HCP: Health Care Providers HP: Health Posts HEW: Health Extension Workers NID: National Immunization Day

Challenges in Human resource

Health workforce development and deployment increase responsiveness to the population's health need for vaccination. To have responsive health facilities and competent health professionals' capacity building and good planning for human resources is important. It is also critical to have an adequate number of well-qualified health workers with an appropriate mix of skills who are equitably distributed and motivated to enable universal access to vaccines in Ethiopia.

The current evaluation of immunization revealed inadequate human resources at all levels, and a lack of training or capacity-building activities, particularly at the lower level; those who took training no refresher training which significantly affected health care responsiveness and the quality of immunization service.

> *"...I suggest the basic immunization training would be better to incorporate with the existing health curriculum at university level thus the professionals would be able to deliver the epi service."* (KII, Regional immunization focal)

The other important issue raised by the key informant is the deployment mechanism doesn't consider the capacity of the health professionals like the professionals assigned to provide immunization service without induction training and or refresher training. The immunization focal is assigned for the position with no prior training in immunization. To address the gaps in human resource planning, capacity building, and

deployment: the participants suggested that there should be continuous refresher training, induction training for the newly deployed health professionals, and incorporating the basic immunization training in the curriculum.

> *"…For capacity building at the institution level, training is given to a focal person on immunization practice/implementation. However, there is no training on immunization updates for the remaining staff which makes service delivery difficult if there is focal person turnover or not available for some reasons. Thus, induction training would be effective."* (KII, Public HC immunization focal)

Another critical concern mentioned by key informants is employee retention and motivation. There are no retention mechanisms, and no incentive mechanisms (financial or non-financial) are in place. If incentive mechanisms are present, they are not consistently implemented at all levels (Region, zones, woreda, and facilities), including at the HEWs level. This contributes to high turnover rates and brain drain from remote rural areas to town health care facilities and private facilities. These have a significant impact on immunization service delivery at all levels. As a result, well-designed, structured, and consistent incentive mechanisms that include financial and non-financial packages must be implemented for all levels of health professionals.

Based on the current (2023) population projection about 109 million people live in Ethiopia. Which means there are 7392 people in one Keble. Based on this assumption and the current practice at health posts the immunization workload of health extension workers at the health post is explained as follows (Table 3.8).

Table 3.8: Human resource work load of immunization at health posts based on the population projection in Ethiopia, 2023.

Parameter	Value
Population in one kebele	7,392
Children (SI 3.2% of population)	237
Pregnant women (pw 5% of population)	370
Child visits needed (5 per child)	1,183
Mother visits needed (2 per mother)	740
Total visits needed	1,923
Visit distribution and staffing	
Visit addressed in static	50%(961 visits)
Visit addressed in out reach	50%(961 visits)
Visits needed per week	37
Visits at static per week	18
Visits at outreach per week	18
Average number of HEWs in one health post	2
Workload and service provision	
Days dedicated for immunization per week	2
Impact of new vaccines(hpv,covid -19) and population growth	Increase workload
The ability of 2 HEWs to provide other services on immunization days	No
Static immunization provision per week	Once
Therefore there is a critical need for additional staffing at health posts	Yes(at least 1)

3.3 Immunization program structure

A service provision structure outlines how a service gets delivered. It covers delivery methods, the responsible team, the steps involved, and how well it performs. This ensures consistent, efficient service that meets user needs.

Current structure

In terms of organization, the MoH's immunization programs are managed by the Reproductive, Maternal, Neonatal, Child, Adolescent Health, and Nutrition (RMNCAH-N) Directorate, which uses the current hierarchy down to the lowest level. The directorate works with the National Immunization Technical Advisory Group (E-NITAG), the Immunization Task Force, the Interagency Coordinating Committee (ICC), and the five technical working groups (TWGs) to coordinate and manage the immunization program (MoH, 2022). The immunization service Lead office is under the Maternal, Child, and Adolescent Health Service Executive Office, led the immunization program at the federal level.

Ethiopia's administrative system serves as the model for immunization's organizational structure at sub national level. Decentralized decision-making occurs at the district and PHCU levels, starting at the regional level. The organizational framework of a region influences the structure at both the regional and zonal levels. For instance, the largest region, Oromia, has two spots for the immunization program, but other regions only have one. Despite an increasing scope and objective for immunization, the immunization program's structure remained unchanged (MoH, 2022). The zonal and woreda level immunization structure is embedded under the MCH unit and one focal person is assigned to execute all immunization related activities. At the zonal level, there is no separate focal person for immunization instead the focal person is responsible for leading child health, nutrition, and immunization. Similarly, the focal person at the woreda level is responsible for coordinating all maternal and child health care services (Figure 3.2).

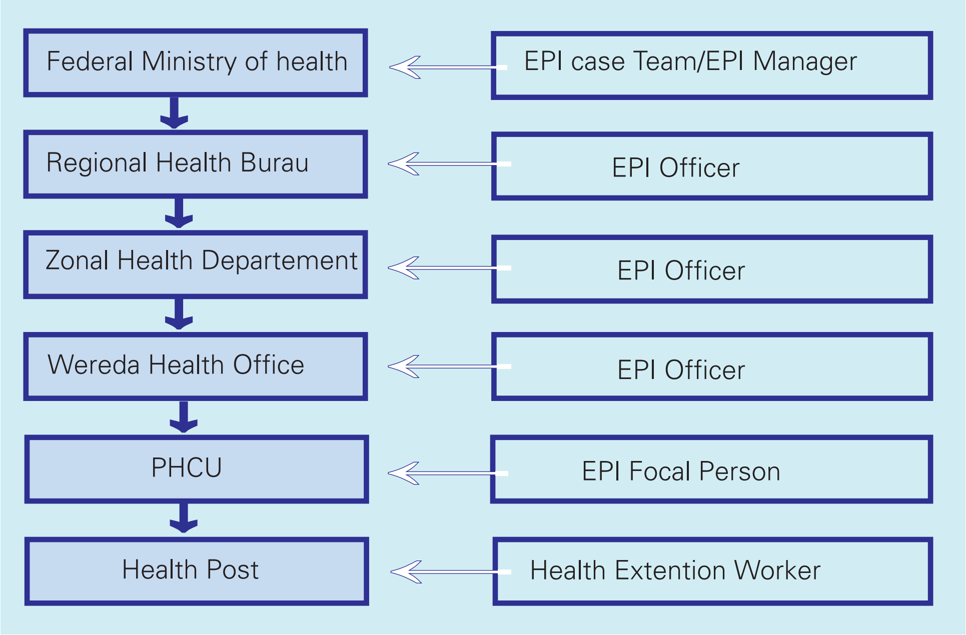

Figure 3.2: The Immunization Management Structure of Ethiopia (MoH, 2022)

Regarding organizational charts the assessment data shows all regions and 47% of the zonal health offices have organizational charts, 13.8% claimed to have the organizational chart and 39.2% do not have it. In all regions, only 62.5% of zonal health offices' immunization programs are placed in the organizational chart of the bureau of zonal health department.

The Woreda level assessment shows that 41.8% of woredas have the organizational chart of the Woreda health office, 15.5% claimed to have the organizational chart, and 42.6%) do not have it. Of those woredas that have an organizational chart, only 7.5% have an immunization program placed in the organizational chart of the woreda health office. At the level of health facilities, 55.2% have an organizational chart of the facility 11.1% claimed to have it but failed to show it and 18.9% do not have an organizational chart. Among those facilities that reported having an organizational chart, 66.7% placed the immunization program in the organizational chart (Table 3.9).

Table 3.9: Availability of organizational chart at the woreda and facility level in Ethiopian health care system by region 2023.

	Woreda		Health facility	
Name of Region	Yes observed	Yes but not observed	Yes observed	Yes but not observed
National	41.8	15.5	55.1	11.1
Addis Ababa	15.4	38.5	68.4	0
Afar	78.6	14.3	44.4	33.3
Amhara	33.3	9.5	57.3	6.6
Benishangul Gumuz	50	12.5	37.5	6.2
Dire Dawa			14.3	14.3
Gambella	50	0	60	0
Harari	25	0	50	20
SNNP	29	9.7	59.6	7.7
Sidama	37.5	25	58.3	16.7
Somali	43.5	26.1	38.9	19.4
South West Ethiopia Peoples	50	0	59.1	13.6

The qualitative findings further described the current immunization structure as reported in the below discussions. The administrative structure of Ethiopia's Expanded Program on Immunization (immunization is designed based on the administrative structure of the country. This decentralizes decision-making, empowering regional, district, and Primary Health Care Unit (PHCU) levels. However, regional and zonal variations exist depending on their specific organizational setups. The immunization structure at the regional level has one to three immunization focal with technical staff support. The zonal and woreda level immunization structure is embedded under the MCH unit and one focal person is assigned to execute all immunization related activities.

One of the Woreda KII stated that the immunization structure at the woreda and facility level is unclear

"The immunization structure at the facility, there are immunization focal at each health facility. However, the immunization focal is not only responsible for immunization activities but also assigned to other activities

but also assigned to other activities e.g. outpatient department. A participant from HC mentioned saying …I am assigned as a facility immunization focal person in charge of immunization service activities, although it is not considered a routine task ...Rather, I am usually assigned to OPD… "(KII, HC immunization focal).

This is supported by another key informant who strengthens the absence of a clear structure for the program. Moreover, he has highlighted that at the health post level, there are a limited number of HEWs who are engaged in addressing all the health extension packages.

"Majority of immunization service is provided by the HEWs. However, they are engaged in providing and working on the eighteen packages. The number of HEWs at health posts is not adequate to provide comprehensive immunization services. The existing community level structure is not effectively led e.g. Health development army." (KII, WrHOs immunization focal)

In general, there is no clear and independent immunization structure at the subnational level. However, the problem is worsening at zonal and lower levels. Immunization focal person at all levels are also responsible for reporting immunization-related activities to the higher hierarchy and giving supportive supervision and feedback to the lower levels.

Effect of current structure on immunization program implementation.

The organizational structure of Ethiopia's Expanded Program on Immunization (immunization) plays a critical role in shaping the implementation of immunization efforts. The current structure impacts the efficiency and effectiveness of immunization implementation efforts across different levels.

The absence of a clear and independent immunization structure at a lower level hinders effective coordination and collaboration with other health programs, potentially limiting synergies and integrated approaches to healthcare delivery. Ultimately, addressing this structural deficiency is essential for ensuring the efficient and equitable delivery of immunization services at all levels of the healthcare system as the current structure is not up to the WHOs recommendation of immunization structural framework (WHO, 2019).

On qualitative findings, it is emphasized that there is no clear structure at the lower level, which has a significant impact on service quality, lower client satisfaction, poor cold chain practice, poor supply chain management, a poor surveillance system, and difficulty maintaining vaccine safety. If there is no clear structure, immunization service delivery in general will be disrupted.

"The current system suffers from rapid personnel changes, a limited number of qualified individuals, vague structure, and a restrictive hierarchical structure." (KII, MoH)

Another result of the current immunization structure is increased staff workload due to insufficient human resources assigned to immunization service provision, which leads to turnover and transfer of trained personnel due to stress and burnout. A participant described how turnover and rotation affect service delivery saying

"The rotation of focal persons varies; some regions do it permanently, while others rotate every 3, 6, or 12 months. The impact of this on service delivery is readily apparent, particularly in vaccination centers. Since vaccine administration requires trained professionals, frequent personnel changes can create difficulties in maintaining service quality and ultimately lead to decreased customer satisfaction and potentially even higher turnover rates." (KII, MoH)

Challenges in the immunization structure

To make immunization programs effective, an independent immunization program structure with sufficient trained manpower and a defined scope of work is in bold print at all levels, particularly at subnational levels. The scope and target for immunization are growing, but the

immunization program is not expanding its structure accordingly.

"…these days there are a lot of campaigns, and a lot of antigens switches, and technology is growing and new vaccines are coming additionally. The human resources are not growing in line with the scope of work. So there must be an increased number of human resources by the government to fill the gap. Temporarily, yes (UNICEF) and other partners' deployed technical assistance but that will not be a lasting solution it's only to fill the gap and to transfer skills and knowledge in some areas. So, the human resources and structure should be well addressed at all levels: regional, zonal, and Woreda levels based on the scope of ever-increasing work." (KII, Federal level Partner).

3.4 Micro-planning

Micro-planning is a strategy in immunization that targets low-coverage areas. It uses maps, data, and community input to identify barriers and create plans for reaching every child with vaccines.

Current practice of micro-planning

In terms of the presence of updated population demographic characteristics for the catchment at the woreda level, 94.8% had it, while the implementation of a Micro Plan for the 2015 calendar year ranged from 50% in Southwest Ethiopia and Harari regions to 100% in Addis Ababa, Gambella, and Benishangul regions with a national percentage of 81.7%.

Less than three-fourths (72.5%) of zones had an updated immunization micro plan. Only one-third (33.3%) were reported in the Somali region. Three-fifths (60.7%) of zones used the same denominator for different programs. One-third of zones in Gambella and 30.7% in SNNP were using the same denominator for different programs. Less than half (45.10%) of the Zonal health departments had an updated 2015 EFY catchment map including the outreach site.

Less than three in four (70.5%) districts and sub-cities updated their immunization micro plan for the year 2015 EFY. Districts in Gambella (25%) and Somalia (38%) regions were the lowest on micro plan updating. More than half (57.58%) and (57.1%) of the districts and sub-cities used the same denominator for different programs and updated the 2015 EFY.

Moreover, in the qualitative study, it is reported that the current practice of micro-planning is characterized by government-focused and well-supported by partners. Federal, regional, zonal, and woreda level leaders work together to coordinate immunization micro-planning efforts and there are established channels for engagingrelevant stakeholders throughout the process. Moreover, there is general agreement that immunization micro-planning is an effective and efficient way to vaccinate every unvaccinated child or vaccinate as per schedule.

The woreda-level participants mentioned the current practice of micro planning is: there is a regional micro plan handed down to the zone and woreda, and the first step is to adapt the plan as needed based on the context to ensure continuous improvement and accountability. The micro plan outlines how immunizations will be conducted at static, outreach, and mobile levels. The other important consideration during micro-planning preparation is the involvement community and relevant stakeholders including funding partners. Most participants at all levels of the health system agreed on the vitality of micro-planning.

"There is a common agreement by participants at all levels was clear: micro-planning is essential. we are diligently collaborating with RHB authorities to develop micro plans. It is the backbone to provide vaccine services. Without well-drafted micro-plans, it is foolish to expect quality immunization." (KII, MoH).

It was further strengthened by another participant who reported implementation plan is done in collaboration with relevant stakeholders.More these stake holders play their own role in the implementation of the plan.

"Micro-planning at Woreda is conducted through integration and collaboration with various stakeholders, including the Office of Agriculture, Office of Education, and Office of Water…we strive to incorporate input and expertise from each stakeholder during the planning process and implement together." (KII, WrHOs immunization focal)

On the other hand, participants mentioned that micro-planning is strongly backed by partners' support logistically as well as through capacity building. Although not many mentioned that capacity building related to micro-planning has observed a decline in the last few years.

"An informant from equity zone reported saying… as part of micro-plan we highlight the plan as a list of activities, the resources needed to implement those activities comes from UNICEF." (KII, Zone immunization focal)

At the facility level, 93.2% reported having updated population demographics of their catchment area, 80.1% were implementing a micro plan with Gambella reporting the highest (100%), and on the contrary, Dire Dawa and Harari had the lowest proportion, (50%). In the case of Health posts, 88% reported having updated population demographic characteristics of the catchment area and only less than half (44%) had updated micro plan for the immunization program, the highest, (63.6%) reported in Afar and lowest in Benishangul Gumuz (16.7%) (Figure 3.3).

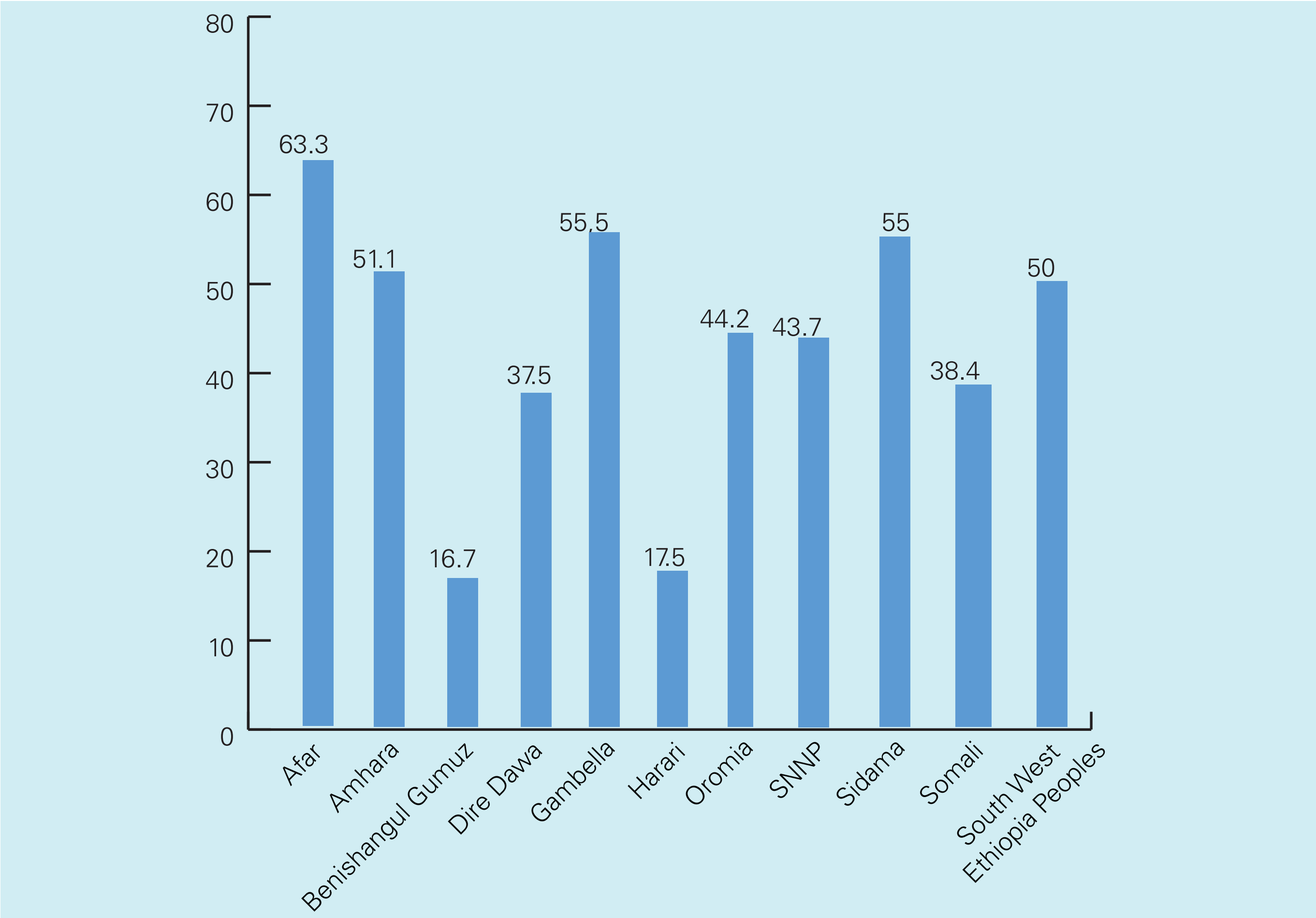

Figure 3.3: Presence of updated micro-planning at health posts in Ethiopian health care system by region 2023.

Challenges Micro-planning practice

Most participants consistently reported several challenges hindering the effectiveness of immunization micro-planning, for instance, outdated census reports to make population projections and to calculate eligible target groups, poor planning, lack of accountability for failure to meet the plan, population influx and knowledge and experience gaps in planning and executing micro-planning. Additionally, the success of immunization activities is often determined by the woreda-based plan rather than micro-planning is also mentioned as a challenge.

A participant from the immunization micro-plan supporting organization reported how the use of projected figures made micro-planning challenging and significantly affected resource allocation at all levels.

> *"There is a denominator issue when doing micro-plan, use of outdated demographic data such as 2007 census projection. This can lead to inaccurate planning assumptions and ultimately affect the effectiveness of micro plan…. then resources will be requested from GAVI with this wrong estimate leading to improper resource delivery."* (KII, partner)

The informants also consistently reported that failure to meet the micro-plans plan has no consequences or means of accountability. Moreover, immunization's performance is more dependent on woreda woreda-based plan than micro-plan planning.

> *"Most of the time we use estimation …the reality was not the same that's why few woredas have more than 100% performance. Even if the performance was above 100%, in reality, are have unvaccinated children."* (KII, RHB immunization focal)

An informant from RHB described how lack of accountability turns made things difficult to practically implement a micro-plan at the grassroots. Another informant supported the same issue saying

> *At the regional level, whether the lower districts work or not there is no accountability. For these reasons, the micro plan has become a challenge to practically enforce on the ground* (KII, RHB immunization focal)

The other issue raised by most participants relates to the population's sudden increase in number which might be due to any natural or manmade reasons, leading to poor performance. A participant from Addis Ababa reported how population influx overinflated their performance when in reality it is not.

> *"For instance, in Addis Ababa, the implementation may reach 140% to 180%. Initially, the estimated number of children may be 200,000, but the actual number may be 500,000-600,000. This indicates there is high flux and informal ways of living…So, somehow these factors may affect the micro plan."* (KII, Zonal immunization focal)

Moreover, lack of support from partners especially in remote areas, and deficiency in capacity of the lower level health care workers is leading to poor practice in planning.

> *"The other consistently reported challenge related to micro-planning is lack of capacity building especially in those areas where partners don't support. Also, there has been poor practice of planning especially at lower levels. An informant reported saying… there is the issue of lack of capacity building for implementers. Not so much training is being given at the level of health extension leading to poor planning and execution."* (KII, Zone immunization focal)

3.5 Resource mobilization and sustainability of financing

Securing long-term funding is crucial for the Expanded Programme on Immunization (immunization) to continue saving lives. Resource mobilization involves strategies to acquire funds from various sources, like governments, donors, and innovative financing mechanisms. The sustainability of these practices focuses on ensuring a consistent flow of resources over time. This might involve diversifying funding sources, advocating for increased government investment, and

exploring cost-effective service delivery methods. By effectively mobilizing and managing resources, immunization programs can maintain their vital role in protecting children from preventable diseases.

The WHO has set an objective to Build and sustain strong political and financial commitment to immunization at all levels to increase

exploring cost-effective service delivery methods. By effectively mobilizing and managing resources, immunization programs can maintain their vital role in protecting children from preventable diseases.

The WHO has set an objective to Build and sustain strong political and financial commitment to immunization at all levels to increase immunization expenditure from domestic resources in aid-dependent countries, and when transitioning away from aid, secure government funding to achieve and sustain high coverage for all vaccines In Immunization agenda 2030 (Lindstrand et al., 2021)

The Ethiopian healthcare financing strategies (1998 and 2020) emphasized the need to increase the resources for health through to improve health service coverage and quality. The strategies acknowledged that to maintain long-term sustainability, health care should be paid through a variety of financing channels. In addition to systematizing a fee-waiver system for the poor, introducing a private wing in public hospitals, outsourcing non-clinical services, setting, and revising user fees, and promoting health facility autonomy through the introduction of a governance system, the reforms brought revenue retention and utilization to the HF level (MoH, 2022).

The government implemented several interventions to protect people from financial hardship and mobilize resources for health, such as full subsidization for the very poor through fee waivers for both health services and CBHI premiums, implementation of CBHI schemes, subsidization of over 80% of the cost of care in public health facilities, and provision of a fee waiver for high-impact interventions through an exemptions program (MoH, 2022).

Funding for vaccinations enters the system through several avenues according to Ethiopia's current practices. One may show this by looking at resources from the Treasury and the rest of the world/aid resources. The government provides the MoH with resources from the treasury in the form of a regular budget, and the ministry may buy supplies for the immunization program and deliver them to HFs directly or through RHBs. Any kind of purchasing framework that the ministry uses could be used for this purchase. The immunization program benefits from subnational allocations of treasury resources in the form of budgets from regionaland woreda governments to the health sector, at least insofar as health service providers' salaries and the supply of facilities for health care are concerned (MoH, 2022).

The funding for the immunization program comes from a variety of sources. Money managed by the MoH may be sent directly to the HFs or RHBs in the form of financing or other inputs. Some partners provide direct financial or supply-side support to the RHBs. The remaining resources for the immunization program are overseen by non-governmental organizations (NGOs), with most of their funding coming from government expenditure channels (MoH, 2022).

Resource mobilization current practice

In this regard findings of this evaluation, study indicate that the majority (83.3%) of the region except afar and Harari have reported having inadequate budget from the government financing for the immunization program. Partners (43.5%), government (43.5%), private (8.7%), and community are the sources of budget for immunization activities

At the zonal level one third (68.6%) zones mentioned partners as a source of budget, followed by government (58.8%). Regarding the allocation of the budget for immunization activities at the lower levels mostly, the budget is allocated based on the national budget allocation (government direction). On adequacy of the budget majority (90.2%) of ZHDs reported that the budget for immunization activities allocated by the government is insufficient. Only 1 ZHD found it sufficient whereas 4 ZHD reps declined to say about adequacy of the budget.

The WoHo reported the sources of immunization budgets for the government were the most dominant source of budget followed by development partners. But Only 3.5% of the WoHOs reported the budget allocated for immunization by the government is adequate with Benishangul Gumuz being the highest (12.5%). About one-third of WoHOs replied that the budget is allocated to the lower-level facilities based on the national government direction. Others said it is allocated based on population size, performance-based funding, expenditure reports, and others (Table 3.10).

Table 3.10: Availability of program budget for immunization, budget adequacy, and the cancellation of critical immunization activities at Woreda level in Ethiopian health care system by region, 2023

Name of Region	Program budget for immunization	Budget adequacy	Critical activities canceled
National	72	3.4	73.3
Addis Ababa	92.3	0%	30.7
Afar	50	0%	42.8
Amhara	76.2	2.4	73.2
Benishangul Gumuz	37.5	12.5	62.5
Dire Dawa	NA	NA	NA
Gambella	100	0	75
Harari	75	0	75
Oromia	79.4	3.2	81
SNNP	54.8	3.2	83.8
Sidama	56.2	0	75
Somali	91.3	13	85.7
South West Ethiopia Peoples	64. 3	0	71.4

From the qualitative findings, informants reported that the government provides partial funding for vaccines through co-financing with partners but this only covers a portion of the total cost. Although there has been an increase in setting budget, it was deemed not enough to meet the demands of the program. It was noted that technical assistance from donors is crucial to the success of the immunization program, but the sustainability of its support is unclear given the huge investment need for immunization service delivery.

An informant from MoH reported how dependent the immunization program is, questioning its sustainability if main funding bodies don't support it despite a commitment from the government side.

> *"Despite the strong commitment, government's budget alone is insufficient to meet the needs of immunization...if external donors don't support the program, especially GAVI, the immunization program will be less sustained."* (KII, MoH)

Although resources and funding have been stated as one of the challenges, few also mentioned that there are situations whereby available resources might not even be used. A participant from the ministry reported that at all levels there are limitations in liquidating and timely taking funds, especially from partners that provide direct cash transfer.

> *"The key issue is even starting from the ministry, region, zones, and woredas are not utilizing the available resources and timely not reporting the liquidation. So because of that, they may lose some amount of money from a funder that provides direct cash transfers for some of the regions, zones, and woredas. Due to that, we can't use it for the intended purpose of micro-plan these are some of the weaknesses that need to be improved."* (KII, WrHO immunization focal).

As stated by the interviewee the routine immunization program did not get the due attention as other case teams, allocating the budget for a new vaccine like COVID-19 were regarded as helpful to divert the budget for the routine immunization services. Conducting a review meeting, supervision and training were some of the activities done by the budget diversion.

> *"There is no dedicated budget for the immunization program. Hence, we use the budget allocated for new vaccines as an opportunity, for instance, a budget for COVID-19 is used to cascade the routine vaccination, it also became a good opportunity to conduct review meetings, supervision, and training for the routine immunization services along with that of COVID 19 related activities." (KII, Zone immunization focal).*

It has not only facilitated capacity building but also traced out the service for children who drop out and those eligible children who never get vaccinated. This will not be achieved unless they use this approach. The other important issue was most of the supporting organizations were not interested in immunization service commonly nutrition and adolescent health were a focus area for the donors. AMREF was mentioned as the one supporting some districts on immunization service including mentorship and capacity-building activities.

Sustainability of financing practice and challenges

Limited domestic resources mobilization and dependence on donors significantly affect the program's sustainability.
Absence of immunization program dedicated budget at the zonal level which significantly affects performance monitoring.

> *"Because of the absence of a dedicated budget specific to immunization, it is difficult to conduct supportive supervision on a regular basis. Rather we use the COVID-19 vaccine campaign as an opportunity to oversee the routine immunization program."* (KII, Zonal immunization focal).

The finding of this study shows that there are cancellations of immunization activities at the regional level due to a lack of budget in the last three quarters of 2015 EFY. In this regard, five regions' Addis Ababa, Afar, Gambella, Harari, and Somali have reported cancellation of immunization activities in the aforementioned period. Mentorship, campaign, annual training, and outreach activities are reported as canceled in regions.

On the other hand, More than half (60.7%) of ZHDs reported that they did not cancel any immunization activities due to lack of funds while 20 reported canceling the activities. Those activities reported to have been canceled by the 20 ZHDs include review meetings, training and

mentorship,Supportive supervision, baby-friendly room preparation and transportation vehicle for immunization, and immunization campaigns.

Similarly, 73.4% of the WoHos reported canceling immunization activities highest (85.7%) in Somali and lowest in Addis Ababa (30.7%). The canceled activities were outreach programs, focused supervision, mentorship, reaching hard-to-reach areas, review meetings, immunization M&E activities, supportive supervisions, vaccine provision (temporarily), technical training,addressing immunization-prevented outbreaks such as measles outbreak, vaccine distribution, mobile vaccine programs, default tracing.

Nationally only 18.7% of the health facilities reported to have a program budget for the immunization. Despite the lack of budget majority (80.3%) of the HFs did not cancel any critical immunization activities because of lack of budget. Among the HFs that reported not having budgets, only 19.7% reported that they canceled critical immunization activities due to lack of funds. The most frequently canceled immunization services at HFs are vaccine transportation services to hard-to-reach areas, refrigerator maintenance, outreach, and mobile sessions, creating community awareness, and tracking loss-to-follow-up. The coping mechanisms used by HF for lack of budget were distributing the vaccines using community members and sharing resources with EPSA
.
From the dimension of facility type Private and NGO/faith-based facilities have cancelled no activities. Similarly, they have a better proportion in terms of having a program budget for immunization activities. Among the public facilities general hospitals have the highest proportion (25%) of cancelling immunization activities due to lack of budget (Table 3.11).

Table 3.11: Availability of program budget for immunization and the cancellation of critical immunization activities at the health facility level in the Ethiopian health care system by region, 2023

Region	Program budget for immunization	Critical activities canceled
National	18.7	19.7
Addis Ababa	15.7	0
Afar	27.8	38.9
Amhara	19	24
Benishangul Gumuz	0	25
Dire Dawa	50	14.3
Gambella	20	40
Harari	20	50
Oromia	47.7	17.2
SNNP	5.7	13.5
Sidama	0	4.2
Somali	12.5	25
South West Ethiopia Peoples	18.2	18.2
Location		
Urban	20.5	18.7
Rural	16.4	21.1
Public facility type		
Referral/specialized	14.3	14.3
General Hospital	12.5	25
Primary hospital	14.63	19.5
Health Center	19.43	20

At the health post level, only 12.4% reported that they canceled critical activities of the immunization program due to a lack of budget. Afar has the highest number of health posts that canceled critical activities of immunization due to a lack of budget. Outreach activities and campaigns are the commonest activities canceled due to lack of budget 59.2%, followed by social mobilization and education 18.4%, RED/REC-Reaching Every District/Community 12.2%, and Training for community members (WDA) 6.1% (Figure 3.4).

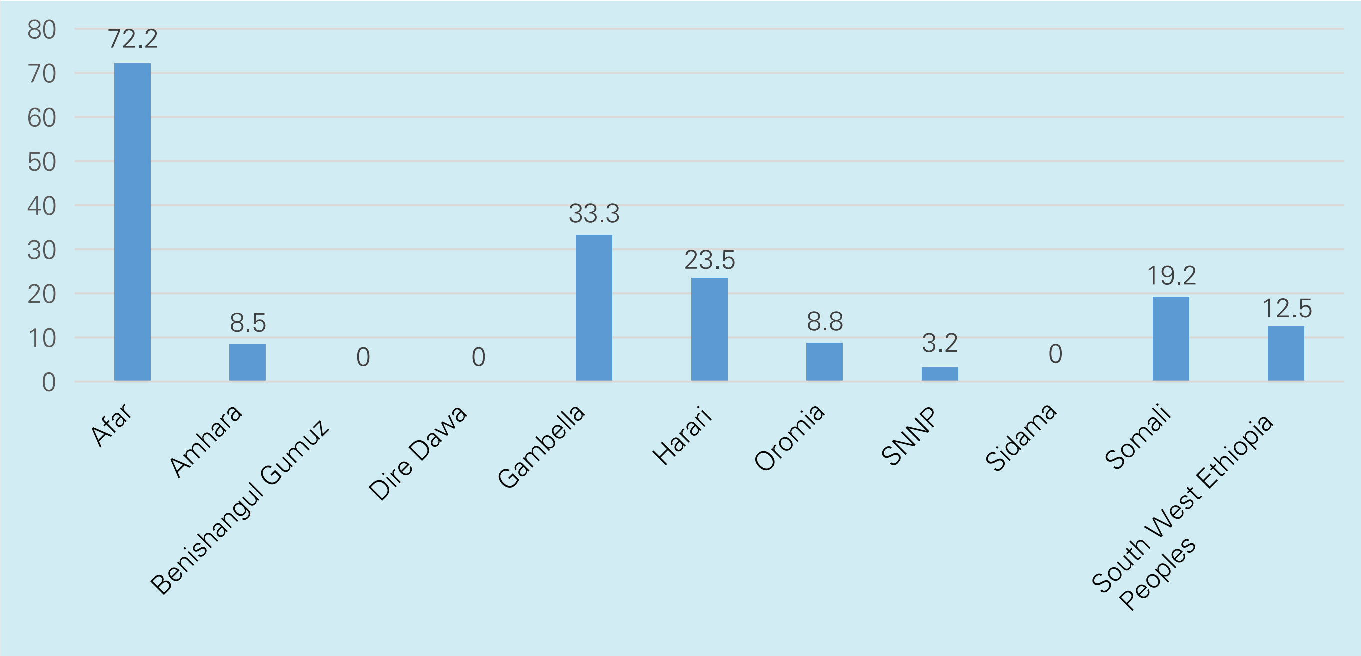

Figure 3.4: Percentage of critical activities of the immunization program canceled due to lack of budget at health posts in health care system of Ethiopia 2023

The qualitative findings further showed that the government fully funds three of the vaccines and co-finances the rest. The government has shown a strong commitment to its funding, and health insurance is expected to provide sustainable financing; hoping that demand from the users' side will aid in this regard and there are signs that current support might not continue as it is. An informant mentioned that despite government increases in funding its sustainability is questionable suggesting that national healthcare financing must address the sustainability issues.

> *"The government has increased vaccine funding to close 500 million ETB from last five years 300K …and aims to cover 70% of the immunization program by 2030, but domestic finance sustainability is a major concern…the national healthcare financing strategy should address these concerns."* (KII, MoH)

On the other hand, the key informants at Woreda and facility levels of the health system highlighted challenges faced in implementing immunization activities due to financial constraints and insufficient funding further compounded by contextual factors such as distance and transport-related limitations. They reported that various activities such as training, supportive supervision, and review meetings among others have not been conducted as planned. A participant from the WrHO office questioned how the immunization program would survive if campaigns stopped altogether and ended his say by suggesting that it needs attention.

> *"There is no budget allocation for vaccination, and we rely solely on campaigns like the COVID-19 campaign, measles campaign, and others to deliver immunization services. What if there were no such campaigns? How would we deliver regular immunization training, supervision, and follow-ups? That is a problem that should be emphasized."* (KII, WrHO)

Expenditure for Immunization Programs in Ethiopia

The data used in this analysis were extracted from the World Health Organization's (WHO) Global Health Expenditure Database. The costs related to the immunization program for Ethiopia are available from 2016 to 2020. Accordingly, five immunization expenditure related variables were considered. These are; Financing sources by domestic and external sources, Total expenditure for immunization, expenditure on immunization as % of Total Health Expenditure (THE), percapita immunization expenditure.

The total expenditure for immunization programs (TEIP) has consistently risen since the data for such programs was available (2016). In 2016, the TEIP for Ethiopia for all immunization programs available during the time was US$ 65.7 million, in 2021 constant and this figure increased by 45.6% to USD 114.2 million in 2019, in 2021 USD constant. However this has decreased to 72,100,238 in 2020 (Figure 3.5)

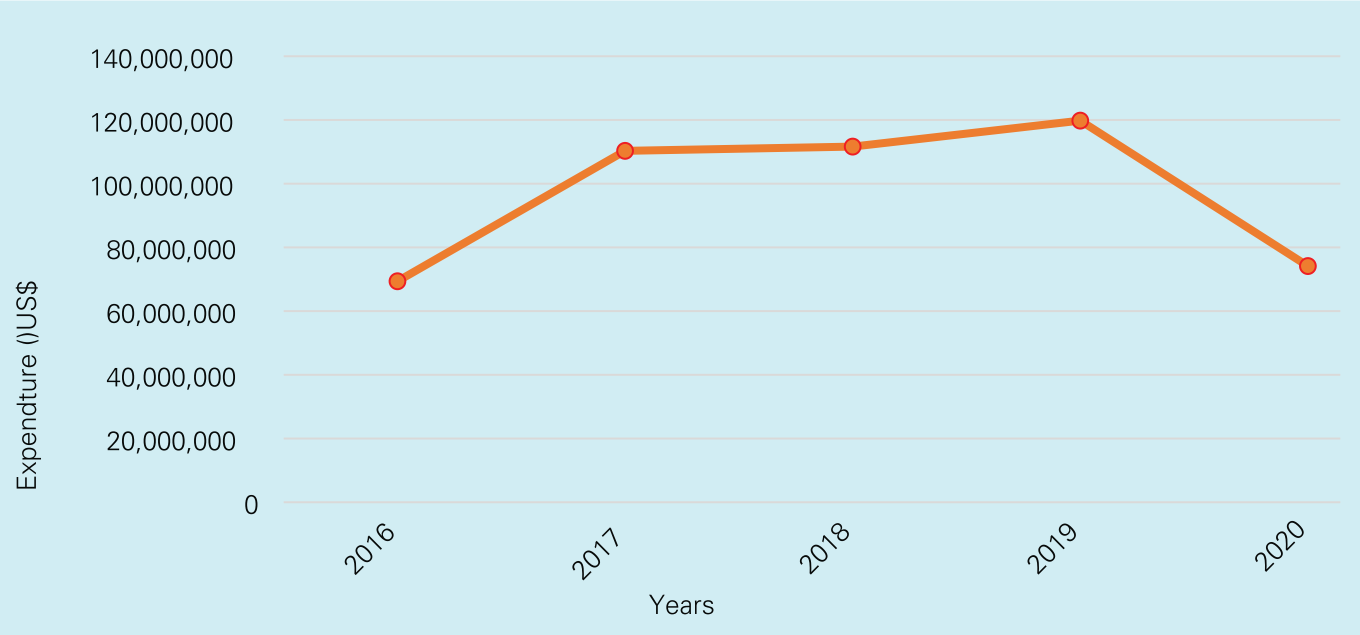

Figure 3.5: Annual total expenditure in USD for Immunizations in Ethiopia, 2016-2020

The share of government expenditure on immunization was higher (18%) in 2016. The government's commitment towards immunization reduced in the subsequent years until it bounced in 2020 to 17% when the COVID pandemic erupted and the flow of foreign aid and load declined due to the northern war. Similarly, the share of private finance in TEIP reduced from about US$ 4 million (6%) in 2016 to almost negligible size in 2020. The lion's share of TEIP has been coming from external sources. In 2016, the share of external sources for TEIP was US$ 49.7 million (76%), in 2021 constant which had been increasing sharply until it declined to US$ 59.6 million (83%) in 2020, in 2021 constant (Table 3.12)

Table 3.12: The costs of immunization in USD, their sources, and per capita costs, 2016-2020, Ethiopia.

Year	Current Expenditure for Immunization Programs (CEIP), US$					Expenditure on Immunization Prog as % THE	Per capita TEIP (US$)
	Domestic sources for CEIP			US$ external sources for TEIP (%)	TEIP (USD)		
	US$ government TEIP (%)	US$ private TEIP (%)	US$ total domestic TEIP, in 2021 constant (%)				
2016	11,801,386 (18)	4,141,035 (6)	15,944,437 (24)	49,715,668 (76)	65,660,105	2.7	0.661
2017	12,446,218 (12)	3,533,162 (3)	15,979,380 (15)	91,835,074 (85)	107,814,454	4.2	1.020
2018	11,928,737 (11)	3,738,916 (3)	15,667,653 (14)	96,736,312 (86)	112,403,965	4.2	1.004
2019	12,403,631 (11)	4,252,924 (4)	16,656,555 (15)	97,542,146 (85)	114,198,701	4.0	1.052
2020	12,513,000 [1] (17)	1,995 (0)	12,514,995 (17)	59,585,243 (83)	72,100,238	1.8	0.526

The TEIP as the share of the THE was 2.7%, 4.2%, 4.2%, 4.0%, and 1.8% in 2016-2020, respectively. The per capita TEIP also increased from US$ 0.7 in 2016 to US$ 1.052 in 2019, before declining to an all-time low (US$ 0.5 per capita) in 2021 since 2016.

Through 2016-2020, the domestic expenditure on immunization made up 1.93% of the domestic general government expenditure on health, whereas, 10% and 5.3% of the external sources of the health system went to immunization programs from 2016-2019, and 2020, respectively. The domestic resource for TEIP was US$ 0.119, 0.118, 0.107, and 0.114 per capita in 2016- 2019, respectively; and the external source for TEIP was US$ 0.501 in 2016 and US$ 0.9 per capita in 2016 and 2019, respectively but this number declined to US$ 0.5 in 2020. The private TEIP expenditure was almost negligible in those years (US$ 0.0402 in 2016) but zero in 2020 (Table 3.13)

Table 3.13: Shares of CEIP from domestic and external sources of funding

Year	Domestic general government expenditure on immunization program as % of domestic general government expenditure on health	External sources of funding on immunization program as % of total external health expenditure	Domestic general government expenditure on mmunization program, in current US$ per capita	External sources of funding on immunization program, in US$ per capita	Domestic private expenditure on immunization program, in US$ per capita
2016	1.9	10.0	0.119	0.501	0.042
2017	1.9	10.0	0.118	0.869	0.033
2018	1.9	10.0	0.107	0.864	0.033
2019	1.9	10.0	0.114	0.898	0.039
2020	1.9	5.3		0.526	0.000

Future resource estimate for immunization programs in Ethiopia

The future resources estimate for TEIP has been made using the AAA (additive error, additive trend, and additive seasonality) version of the exponential triple smoothing (ETS) algorithm of Excel as follows. First, we forecasted the total TEIP for 2021-2025 using the 2008-2020 CHE data. The 2020 and 2025 CHE data have been used as Forecast Start and Forecast End periods of the ETS. 2008-2020 have been used as the Timeline Ranges and the corresponding THE data as value ranges. Since the data available for TEIP (2016-2029) are not sufficient to make forecasts for 2021-2025, we used alternative forecasts of the highest (4.21) and lowest (1.8) per capita TEIP for the prediction of TEIP in US$ for the years (2021-2025) (Table 3.14)

Table 3.14: Forecasted Immunization cost using the ETS algorithm, Ethiopia 2023

Timeline	Forecast TCHE in US$)	Best TEIP (US$) if per capita TEIP= 4.21% of THE	Medium TEIP, if per capita TEIP= 3% of THE	Low TEIP, if per capita TEIP= 1.8% of THE
2021	3,184,776,097	134,079,074	95,543,283	57,325,970
2022	3,274,700,739	137,864,901	98,241,022	58,944,613
2023	3,686,527,972	155,202,828	110,595,839	66,357,503
2024	3,857,912,469	162,418,115	115,737,374	69,442,424
2025	4,029,296,967	169,633,402	120,878,909	72,527,345
Total		759,198,320	540,996,427	324,597,856

The forecasted cost is lower than the financial plan formulated by the MoH for the year 2021-2025 comprehensive mid-year plan. The total resource estimate in this forecast is about 760 million US$, whereas, that of the MoH-E is 1.16 billion USD. A possible explanation is that the forecast is based on the expenditure between 2016 and 2020 and other factors that probably raise the cost have not been considered. As a Limitation the WHO Healthcare Expenditure database we used is only till 2020.hence, in availability of current data has its own limitation in reflecting the existing situation. The other limitation of this report is that it is used from secondary source as there was limitation of time in collecting primary data.

3.6 New vaccine introduction

New vaccine introduction is the process of adding a safe and effective vaccine to a country's routine immunization program. It involves careful evaluation to ensure the vaccine fits the country's needs and resources. This process aims to protect people from a new disease or improve immunity against an existing one.

Current practice in introduction of new vaccine

All study units were asked whether they had introduced new vaccines in the past 2 years of the study times; almost all reported that they had introduced new vaccines. Among the health service provision units that introduced new vaccines, 90.05% HPs, 85.9% HFs, 6.6% WrHOs, and all assessed ZHDs reported using at least one type of media to promote the new vaccines and create awareness. The modalities used by the healthcare delivery units were the Women Development Army (WDA), brochures and leaflets, announcements at community gatherings, and religious institutions. Most of the healthcare delivery units used more than one media modality for the promotion of the new vaccines.

Availability of vaccine stock records in the study units while introducing the new vaccine.

Among the HPs that introduced new vaccines, 18.1% reported having vaccine stock records that were observable. The remaining HPs either did not have the records or were reported to have them but could not be observed. Similarly, 19.5% of HFs that introduced new vaccines in the last two years reported that they had stock records while introducing the vaccines. On the other hand, proportionally, a similar number of HFs in rural and urban areas had the stock registers and location had no significant influence on the availability of the vaccine stock records in the HFs that introduced new vaccines recently. Regionally, all the woredas from Addis-Ababa and Gambella had vaccine stock registers.

Availability of feedback logbook during supervisory visits for the new vaccines

Among the HPs that introduced new vaccines in the past 2 years, 13.6% had feedback logbooks that were observed during the supervision of the new vaccines. Similarly, it was revealed that 28.0% of HFs that introduced new vaccines in the last two years had site visit reports and feedback logbooks that were observed during the supervision. On the other hand, among the total assessed woredas with the new vaccines, 45.8% had supervisor’s books (site-visit reports) for the new vaccines that they could show during the supervision.

Regarding injection safety/waste management policy documents, 30.5% of the HPs that launched new vaccines had injection safety/waste-management policy documents for the vaccines. Of the total assessed woredas, 81.8%, that introduced the new vaccines had injection safety (waste management) policy documents.

Availability coverage and dropout rates of the new vaccines in the institutions

Of the total assessed HPs, only 18.6% reported that the coverage and dropout rate data existed in their facility. Similarly, 45.8% of the woredas had the dropout and coverage data verifiably. Woredas from Gambella Benishangul-Gumuz and SW Ethiopia performed better as all of their woredas that introduced new vaccines had the coverage and dropout rates data.

This study also revealed that all the 39 zones that introduced new vaccines had dropout rates and coverage data for the new vaccines.

Availability wastage reports for the new vaccines in the institutions

Of all HPs that introduced new vaccines in the last two years, 13.6% had wastage reports that were observed during the data collection. Also, it was shown that over 47% of woredas that introduced new vaccines either had no wastage reports for the new vaccines or reported to have them but could not be observed during the supervision. Woredas from Gambella, Benishangul-Gumuz, and South West Ethiopia performed better as all of their woredas that introduced new vaccines had wastage reports.

Similarly, 34 of the total zones that launched new vaccines in the last year said that they had vaccine wastage reports for the new vaccines.

Availability of adverse events following immunizations (AEFI) logbook for the new vaccines in the institutions

Among the HPs that introduced new vaccines, only 17.3% had logbooks to register AEFI. 70.9% of woredas had AEFI logbooks. Woredas from the Gambella region outperform others as all of them reported to have the AEFI logbooks. At the zone level, 31 of those who launched new vaccines in the last year said that they had AEFI logbook for the new vaccines (Table 3.15).

Table 3.15: Availability selected items in the HPs, HFs, and woredas that started new vaccines in the last 2 years.

Region/City admin	Availability of stock records for the new vaccines (observed)			Availability of feedback logbook for the new vaccines			Availability coverage and dropout rates of the new vaccines		Availability wastage reports for the new vaccines	
	HPs	HFs	WrHOs	HPs	HFs	WrHOs	HPs	WrHOs	HPs	WrHOs
Addis-Ababa	NA	0.0	66.7	NA	44.4	33.3	NA	50.0	NA	50.0
Afar	55.6	31.3	53.8	22.2	25.0	76.9	11.1	53.8	11.1	53.8
Amhara	10.0	7.2	62.1	25.0	20.3	37.9	32.5	48.3	17.5	51.7
Benishangul Gumuz	11.1	0.0	66.7	11.1	15.4	66.7	11.1	116.7	22.2	116.7
Dire-Dawa	0.0	7.7	NA	0.0	23.1	NA	12.5	NA	0.0	NA
Gambella	0.0	0.0	50.0	42.9	20.0	50.0	0.0	50.0	14.3	50.0
Harari	23.5	37.5	0.0	5.9	25.0	0.0	5.9	66.7	11.8	66.7
Oromia	13.8	17.4	65.3	8.6	37.0	46.9	25.9	59.2	15.5	51.0
Sidama	20.0	33.3	40.0	0.0	33.3	50.0	13.3	50.0	20.0	50.0
SNNPR	17.4	30.0	47.4	13.0	34.0	47.4	17.4	52.6	13.0	63.2
Somali	38.1	51.9	37.5	14.3	14.8	31.3	4.8	31.3	0.0	25.0
SW Ethiopia	21.4	17.6	27.3	14.3	11.8	54.5	14.3	45.5	14.3	54.5
Total	18.1	19.5	53.0	13.6	28.0	45.8	18.6	53.0	13.6	52.4

Opportunities to introduction of new vaccine

A number of Partners were supporting the implementation of new vaccines like measles 2, COVID 19, HPV, and IPV. The policies and the new implementation guideline have been updated and have been in use since the new vaccines were introduced in Ethiopia. In addition, different nongovernmental organizations have participated in the development of the implementation guideline. This has been described by a participant as follows:

> *"…I feel the ministry is very strong with engaging partners for guideline preparation and putting a timely strategic plan as well. Concerning documents apart from that the immunization team engaged every time for example on the updating of strategic plan, documents when the new vaccine introduction is required"* (KII, MoH)

CHIA was also involved following the start of the Rota vaccine and played a key role in introducing the vaccine in Ethiopia as well as supporting the system with a lot of activities like planning, capacity building, and budgeting. With the help of this partnership remarkable progress has been achieved in the reduction of under-five mortality from diarrhea and pneumonia.

Different mentions on collaboration were notably mentioned by the health extension workers underlining the significant role of schools during the introduction of the HPV vaccine. They described their view on

the possible suggestion for the new vaccine as emphasizing on need for collaboration with sectors like education. It would make the way easy if it addresses the influential person in the community like the religious leaders. Providing training for the women's development army has appeared as one of the strategies to increase awareness among the community.

The religious leaders were engaged in encouraging the community when the health facilities announced to implementation of the COVID-19 vaccine.

> *"Another opportunity for the immunization service is the collaboration with community and religious leaders. These influential figures actively educate community members about the importance of immunizing their children and foster a positive attitude towards vaccinations. This has been particularly valuable during the immunization campaign against COVID-19."*(KII WrHO immunization focal)

Moreover, the introduction of new vaccines like COVID-19 has brought an opportunity to strengthen the cold chain system for immunization in general at different sites and brought additional human and financial resources to the system. The other reported finding from most participants relates to COVID's role in strengthening cold chain capacity. A participant from ZHD mentioned saying.

> *"COVID-19 vaccine has brought an additional human and financial resource to the local area which in turn supported to strengthen the health system. It has also enhanced the cold chain capacity."* (KII, ZHD)

Challenges in new vaccine introduction

Implementing new vaccines in the region faces a multitude of challenges, including inconsistent guidelines, limited support from partners, negative community perceptions, insufficient funding, lack of trained personnel, and inadequate cold chain capacity for storing vaccines. It underlined the need for updated training due to the implementation of the new vaccine as well as the addition of the new HEWs in the previous year. However, only one module was included in an integrated ART refresher training course has highlighted a gap regarding the capacity building.

In a few study settings, participants reported that there was opposition from political leadership which undermined the effort to direct the importance of these new vaccines.

One participant from WrHO reported how political leaders neget the introduction of a new vaccine saying

> *"… The opposition political leadership seems to disregard the importance of these new vaccines. Some resistance towards vaccines arises due to the misconception of "vaccines being administered while giving birth campaign"* (KII, WrHO, Immunization focal)

As stated by the informants, it has emphasized the need for improvement in the implementation across the region, there is inconsistent implementation of the guideline for new vaccine introduction was seen as a major issue. In addition, partner support has also played its role while UNICEF and WHO were supporting only limited settings due to security issues in some regions. Beyond the logistic and financial support, it has also involved in the recruitment of technical assistance while developing the micro plan

> *"…. if not implemented uniformly across the regions because of the limited capacity, resources, because of security and other in some area."* (KII, MoH)

The limited capacity and resources of the Ministry of Health related to technical expertise, and the lack of external technical support from partners for planning and review meetings is one the challenges for the new vaccine. It has been suggested to capacitate the health professional, preparing guidelines and updating the HMIS system.

Most study participants had identified a lack of sufficient information and evidence for decision-making as a challenge for new vaccine introduction. An informant reported that the negative perception of the community towards the HPV vaccine has been challenged a lot while the ministry tries to introduce it, the community perceived as HPV vaccine will result in infertility.

> *"…. for example, when we introduced the HPV vaccine, it was challenging because different rumors were running in the community like it makes people infertile."* (KII, MoH

Another challenge reported was the inadequacy of human resources for the new vaccine and logistics like low cold chain capacity has been reported as a major challenge. It has been mentioned that the health facilities face financial constraints they are unable to pay incentives for health workers and to enhance the cold chain capacity.

3.7 Conclusions

The effectiveness of leadership, management, and governance (LMG) practices have a significant impact on the coverage of immunization programs, equity quality of service, acceptance, and sustainability in Ethiopia. Ethiopia has made remarkable strides in vaccine finance, moving from complete reliance on foreign help to self-funding for certain vaccines, according to the findings of this national immunization evaluation of the LMG sub-section. Strong collaborations, revised policies, the introduction of new vaccines, rising vaccination rates, and the use of micro-planning have all contributed to the nation's successes in the program.

However, several challenges including the disconnect between strategic planning and operational execution, structural limitations within the Expanded Program on Immunization (EPI), inadequate provider commitment, lack of recognition practices, weak coordination, difficulties in liquidating funds and timely reporting, poor leadership skills, frequent turnover of human resources, reliance on outdated and inaccurate population projections for planning, insufficient capacity development, and lack of accountability persist.

3.8 Recommendations and Implications

- Addressing structural limitations within immunization: change the structure to meet the growing demand for immunization program
- Bridging the gap between strategic planning and operational execution: ensure plans are realistic and feasible
- Increasing provider commitment and motivation: provide incentives, recognition, and opportunities for professional growth and focus on the primary healthcare level
- Improving population projection data: use accurate data from census or headcount
- Securing reliable funding and simplify the process of liquidating funds
- Sustaining micro-planning efforts: continuously monitor and evaluate plans and their execution
- Capacity building: equip immunization leaders and providers with existing evidence
- It is crucial to facilitate multi-stakeholder collaboration, establish clear policy frameworks, enhance capacity building, mobilize resources, engage the community, and strengthen monitoring and evaluation mechanisms to improve public-private partnership in immunization.

SUPPLY CHAIN MANAGEMENT

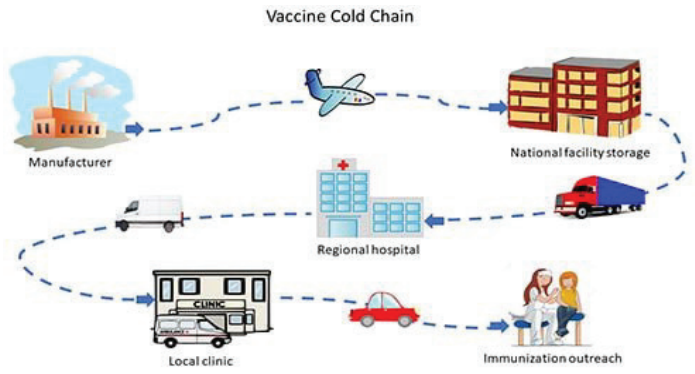

4. Supply Chain Management

Immunization Supply Chain (iSC) Management refers to the process of ensuring that vaccines are effectively stored, transported, and distributed from manufacturers to HFs. The iSC encompasses all the people, activities, infrastructure, resources and planning necessary to ensure that vaccines stay safe and effective and reach their destination. Effective iSC management is essential for maintaining the potency and quality of vaccines, minimizing wastage, and maximizing vaccine coverage to protect populations from vaccine-preventable diseases.

The WHO Effective Vaccine Management (EVM) tool was used to collect data on vaccine supply chain management system of the health system. The assessment covered one central hub (CH) and 15 Sub-central (SC) Ethiopian Pharmaceutical Supply Service (EPSS) hubs. The sub-central hubs were fairly distributed across various Ethiopian regions, including four in Oromia, three in Amhara, two in Addis Ababa, and one each in Somali, Sidama, Gambella, Dire Dawa, Benshangul Gumuz, and former SNNP. At lower distribution (LD) and service provision (SP) levels, 286 institutions were assessed. These included 101 (35.3%) health centers (HC), 90 (31.4%) Woreda Health Offices (WorHO), 83 (29%) health posts (HP), and the remaining 12 (4.2%) hospitals. The majority of institutions were from Oromia, 85 (29.7%) and Amhara regions, 52 (18%), followed by SNNP, 34 (11.8%). Only five HCs were assessed in Addis Ababa.

Key Findings

- The overall Ethiopian 2023 EVM assessment score was 69%, far less than the desired EVM standard 80% score.
- Vaccine forecasting is challenged by lack of denominators, city immigration, and cross-boundary movement.
- There is a serious shortage of refrigerated vehicles, functional 30-day temperature records, and proper temperature-controlled refrigerators.
- Collaborative efforts involving regional health bureaus and external partners have contributed to maintenance and repair of cold chain equipment.
- Lack of well -equipped maintenance workshops, budget constraints, and shortage of skilled technicians remain significant barriers to ensuring the proper functioning of cold chain equipment.
- Although stock records are well-organized and identified by vaccine type, they are mostly insecurely placed at the central hub and four sub-central hubs.
- Vaccine management is still challenged by late requests and a shortage of cold van transportation, leading to stock-outs at facility levels.
- The shift to on-demand, proximity-based, last-mile delivery has improved vaccine distribution, but data visibility, transportation, infrastructure and vehicle availability challenges persist, especially during rainy seasons at lower levels.
- Many health institutions lack adequate storage capacity and infrastructure, leading to vaccine stock-outs and wastage, with average storage capacities of 1433.9 liters for positive vaccines and 538 liters for negative vaccines.
- Institutions lack schedules or documentation for preventive maintenance of buildings and refrigeration equipment, with maintenance hindered by shortages of funds, spare parts, and skilled professionals.
- At lower distribution levels, shortages of ledger books, transportation, poor road conditions, and inadequate infrastructure cause stock-outs and EPI service interruptions, with 18% of health institutions having VVMs beyond discard time and 16% storing expired vaccine vials.
- Waste segregation was uncommon among institutions, with poor use of color-coded bins, and disposal methods leading to overfilled waste and risks of hazardous spills.

4.1 Immunization Supply chain operations and management at central and sub-central levels

Vaccine arrivals at Central and Sub-Central (SC) Hubs

The qualitative study revealed that vaccine arrival tasks including vaccine procurement, shipping, and delivery to the central store were conducted by the EPSS, collaborating with customs, shipping agencies, and port administration. The procurement process encompasses annual and micro-plans, ensuring continuous supply, adapting to evolving needs, and effectively vaccinating the eligible population.

Temperature management during transportation at central and sub-central hubs

Because of their high sensitivity, vaccines require proper temperature maintenance during transportation. The central hub has two refrigerated vehicles, each equipped with temperature-monitoring units in the driver's cabin, while the seven sub-central hubs have one refrigerated vehicle each; only two had functional 30-Day Temperature Records (30DTR) Table 4.1.

Findings from the qualitative data also indicated that at the central level, cold chain management involves an annual calibration of vaccines, utilizing refrigerated vans for transportation, and implementing a remote temperature monitoring device for information dissemination.

Table 4.1: Temperature management of vehicles at Central and sub-Central hubs, Ethiopia 2023.

Variables		Central	Sub - central	Total
Total number of refrigerated vehicles	Two vehicles	1	0	1
	One vehicle	0	7	7
	No refrigerated vehicles	0	8	8
Number of vehicles with a Temperature monitoring unit in the driver's cabin	2	1	0	1
	1	0	6	6
	0	0	1	1
Number of vehicles with a functional temperature monitoring unit	2	1	0	1
	1	0	4	4
	0	0	2	2
Number of vehicles with a functional 30-Day Temperature Record (30DTR) in	Yes	0	2	2
	No	1	5	6
Number of vehicles equipped with a geographic location transmitter	52	1	0	1
	8	0	1	1
	5	0	1	1
	4	0	2	2
	2	0	1	1
	1	0	8	8
	None	0	1	1

Storage and transport capacity at central and sub-central hubs

Understanding storage capacity and identifying gaps in vaccine supply chains are crucial for minimizing damage risks. Table 4.2 presents cold room and refrigerator capacities. The net storage capacity of refrigerated compartments of cold rooms in the central hub is 440.8 m3, while the storage capacity of freezer compartments is 40 m3. The net storage capacity of refrigerator compartment of cold rooms scored in SCHs was 798.2 m3 with a mean (SD) of 53(37) m3. Only four SCHs had freezer compartments in their cold rooms with aggregate capacity of 11.8 m3. Six SCHs have a total net capacity of 2430 liters of refrigerators; thirteen hubs have a total net vaccine storage freezer capacity of 13930 liters. The total storage capacity of the dry goods was 90000 m3 for the CH. The net storage capacity of the dry goods was 16206 for all SCHs. The CH's refrigerated vehicle total transportation capacity was 41 m3, while nine SCHs had a total capacity of 85.3 m3 (mean [SD]: 17.1 [11.2] m3). Non-refrigerated vehicles at the Central

hub hold 1746 m3, compared to 1004.1 m3 for the nine SCHs (mean [SD]: 111.6 [76.6] m3). The CH possess a net storage capacity of 1969.8 liters in insulated containers, while SCHs average 763.6 liters (mean [SD]: 63.6 [27.6] liters).

The challenges regarding cold chain management at CH and SC level include inadequate number and capacity of cold vans affecting the transportation of vaccines from the airport to regional hubs and from the head office to branches leading to delays in vaccine delivery, impacting the overall efficiency of the cold chain.

> *"…there are not enough cold vans to clear vaccines from the airport. Since the distance is close, sometimes other vans are used. Another thing is that there is not enough cold van to deliver even from the head office to the branch. So sometimes while we are waiting in line, one car will go to a certain branch and refill them. Therefore, our delivery will also be delayed. The biggest shortage is the shortage of refrigerator vehicles".* KII, Central EPSS

Table 4.2: Storage capacity of cold rooms, refrigerators, and dry storage area at the Central and sub-Central EPSS hubs Ethiopia, 2023.

Variables	Central	Sub-Central		
	n=1	N	Total	Mean (SD)
Cold rooms storage capacity in m^3				
Gross storage capacity of the refrigerator compartment	2249	15	2224	148(69)
Net storage capacity of the refrigerator compartment	441	15	798	53(37)
Gross storage capacity of the freezer compartment	201	4	72	18.(3)
Net storage capacity of the freezer compartment	40	4	12	3(1)
Refrigerators capacity in Litres				
Net vaccine storage capacity of the refrigerator in	-	6	2430	405(278)
Net vaccine storage capacity of the freezer	-	13	13930	1071(401)
Net cool water-pack storage capacity of the refrigerator	600	8	3613	451(252)
Net ice-pack storage capacity of the freezer	435	13	11404	877(565)
Total storage capacity: Vaccines, positive	440768	15	779304	51953(38666)
Total storage capacity: Vaccines, negative	40000	13	25743	1980(3504)
Total storage capacity: Cool packs	44677	13	37555	2888(1627)
Total storage capacity: Icepacks	40000	13	25743	1980(557)
Total storage capacity: Coolant packs	4435	15	12501	961(3403)
SD=Standard deviation, n=sample size				

The central hub and sub-central hubs stored dry goods in containers or on racks. The central hub had a total storage capacity of 9000 m^3 for dry goods and net storage capacity of 1,969 liters, while 15 sub-central hub had 7,758 m^3 total storage capacity of dry goods and 12 had 763 liters of net storage capacity of the insulated container. Nine sub-central hubs and the central hub had air conditioning. Fourteen SCHs and the CH reported no direct sunlight exposure (Table 4.3).

Table 4.3: Dry good storage at the Central and Sub Central EPSS hubs

Dry Goods storage of hubs in m	Central (n=1)	Sub Central (n=15) Observed	Total	Mean (SD)
Gross storage capacity of the dry goods	98	15	24987	1665(3762)
Net storage capacity of the dry goods	90	15	16206	1157(2665)
Total storage capacity: Dry goods (m^3)	9000	15	7758	517(876)
Net storage capacity of the insulated container (in litres)	1969	12	763	63(27)

Maintenance and repair of building at central and sub-central hubs

Very few responded to building maintenance questions. A few (7) sub-central hubs recorded preventative maintenance schedules. Among the seven sub-central hubs with maintenance records, 6 adhered to the schedule, and had documented procedures for reporting fault and damages. Although 12 hubs had repairs completed in the past year, only seven have documentation. In eight hubs, including the Central, facility handles building maintenance, while in four repairs are conducted by the facility and NGO or the facility and government.

Maintenance and repair of temperature management equipment at central and sub-central hubs

Regional Health Bureaus, along with Partners, and GHSC-PSM, oversee cold equipment maintenance, installation, and monitoring. This involves scheduled visits, monthly inventory, and coordination to address technical issues. The Central Hub and 13 Sub-central Hubs have conducted preventive maintenance and repaired refrigerators in the past year. At central hub maintenance of refrigerator's equipment were conducted by facilities themselves and NGOs (Table 4.4).

Table 4.4: Maintenance of refrigerator's equipment of the Central and Sub-Central Hubs

Variables		EPSS hubs		Total
		Central n=1	Sub Central n=15	
Responsible body for refrigeration maintenance	NGOs	0	3	3
	Governmental & NGOs	0	2	2
	Facility	0	2	2
	Facility and NGOs	1	4	5
	Facility and Governmental organizations	0	1	1
	Facility/Governmental and NGOs	0	3	3
EPSS= Ethiopian Pharmaceutical Supply Services, NGO=Nongovernmental Organisation				

Maintenance and repair of vehicles at central and sub-central hubs

Twelve sub-central hubs had vehicle preventive maintenance schedules, 10 having records of completed work. Ten hubs had vehicle maintenance by the facility, while two had repairs by the facility and NGO, including the Central hub. Private Service providers handle repairs in two hubs, and government offices handle maintenance in one hub. Lack of a well-equipped workshop, budget constraints, and a shortage of skilled technicians are barriers to maintaining cold chain equipment.

> *"But there are lots of challenges in distribution, especially transportation. The cold vehicles have not been adequately managed. The cold vehicles and their cold rooms have not been regularly maintained, the staff who were working at the warehouse have not been capacitated and engaged, and the rooms are not properly maintained and managed. At the country level, it is expected to have a workshop, but unfortunately, we do not have one. In addition, for the staff who are working at the warehouse, their career structures have not been explicitly defined. Even in our project, there are 19 cold vehicles, but half of them need maintenance service. The cars are managing commodities, not vaccines, because their cold room system is not working. There is support from partners in designing protocols, capacitating the staff, and the like."* KII, Partner at the Central level

Stock management at central and sub-central hubs

The central hub and all sub-central hubs had computerized stock records, the maximum levels recorded at the central hub and 14 sub-central hubs. Stock records were well-organized at the central hub and four sub-central hubs. The vaccine stock in all hubs was arranged by vaccine type and expiry lot. Stock records were securely placed at the central and four sub-central hubs. The central hub and two sub-central hubs kept vaccine stock records for at least 3 years.   The central-hub and one sub-central hub conducted four physical stock counts in the last 12 months, while others varied. Seven sub-central hubs had the highest number of physical stock counts, with 12 records (Table 4.5).

Table 4.5: Vaccine management of the lower and service provision levels by health institution type in Ethiopia 2023.

Variables		Central	Sub-Central	Total
Presence of expired vials in the cold/freezer rooms		0	1	1
Facility has stock records for each vaccine on the vaccination schedule		1	14	15
Stock record completeness		1	14	15
Facility has diluent stock records		1	11	12
Well organized vaccine stock records		1	5	6
Vaccine stock records stored in a secure place		1	4	5
Number of physical stock counts made in last 12 months	12 Times	0	7	7
	11 Times	0	1	1
	9 Times	0	1	1
	4 Times	1	4	5
	3 Times	0	1	1
	1 time	0	1	1

Effective vaccine management relies on proper storage, stock control, inventory, and handling. The EPSS system ensures Central-level compliance with cold chain standards through inspections and documentation. Routine vaccinations are held 1 to 4 months at branch levels and 3 to 6 months at Central levels. Stock management includes reporting levels, submitting requests, and using platforms like WhatsApp for communication.

> *"Regarding stock management, we usually set the minimum and maximum plan. We monitor monthly stock, and branches send their report quarterly, but they send their monthly vaccine stock in total too. Then we take stock from the head office and check if that stock is within the range."* KII, EPSS Central level

Challenges related to either not timely sending the request by the lower levels or to a shortage of cold van transportation, where the regional hubs could not deliver the vaccines lead to stock-outs at facility levels. All sub-central hubs electronically submit vaccine requests to the central hubs, with 14 hubs recording these requests. All the sub-central hubs and the central hub receive vaccine requests and maintain records of vaccine release. In the central hub and 13 sub-central hubs, vaccine stocks are replenished on a regular schedule.

Over the past 12 months, hubs submitted varying quantities of requests: three hubs each made 12, 10, and 7 requests, while 11 hubs made four requests in total. Some hubs made unscheduled emergency requests: one hub made seven, three hubs made two, and one hub made one. Only four hubs included some or all of the following data items in their report: temperature alarm data, functionality of cold chain

equipment, vaccine consumption data, vaccine stock data, closed vial wastage data, and vaccination data.

Key informants stress the importance of data management and visibility at all levels of the vaccination supply chain, advocating for automated data visibility to support decision-making. Regional health institutions submit vaccine report forms for timely delivery, essential for managing stock-outs and forecasting. However, data visibility is inconsistent across healthcare systems, with hospitals having better visibility than facilities below the zonal level. Lack of automated data access hampers efficiency, and inadequate tools at the branch level contribute to incomplete end-to-end data management.

Transportation and distribution of vaccines and dry goods at central and sub-central hubs

Ethiopia's vaccine distribution has shifted towards last-mile delivery, with coordination through the EPSS system transforming the hierarchical ministry system to a district level. Ten sub-central hubs collect vaccines from the central hubs, which then distributes to five sub-central hubs. All hubs, including a Central and 15 sub-central hubs, distribute vaccines, with eight sub-central hubs allowing other institutions to collect vaccines. Adequate space in the vaccine packing area facilitates timely transportation and distribution.

Of all hub vehicles, 52 vehicles at central and 31 vehicles at sub-central hubs are equipped with geographic location transmitters, while fire extinguishers are present in 52 vehicles at central and 28 vehicles at sub-central hubs, three lacking them. Contingency plans for transportation emergencies exist in the central hub and 10 sub-central hubs, with vaccine transportation logbooks available in the central and nine sub-central hubs.

Under the current system, EPSS hubs directly deliver vaccines to WorHOs or to health facilities equipped with refrigerators or storage capabilities, bypassing the zonal health offices or regional bureaus. However, travel duration was influenced by road conditions, potentially affecting vaccine quality; and problem of compliance with WHO-recommended temperature ranges during transportation.

Vaccines are collected, distributed, or provided to recipients using road vehicles by the central and all sub-central hubs. Despite efforts for last-mile delivery, Ethiopia's vaccine distribution faces challenges, including poorly managed cold chain vehicles, insufficient staff, challenging terrains, and conflict zones, leading to delays and interruptions in vaccination campaigns.

Various strategies have been attempted to address conflict-related challenges, such as involving faith-based institutions in vaccine delivery in certain areas and implementing hit-and-run practices in conflict zones. Catch-up campaigns are conducted after conflicts end. Limited local humanitarian involvement complicates addressing challenges, potentially increasing zero-dose cases. Suggestions include improving cold chain capacity to enhance last-mile delivery to health institutions.

> *"…EPSSA should maximize direct supply site and minimize indirect supply approach for vaccine supply distribution."* KII, Regional EPI focal person

Waste management at central and sub-central (SC) hubs

Waste in immunization is inevitable, necessitating proper waste management as an integral part of immunization services. The readiness of the supply chain system is crucial for implementing an effective waste management system.

Waste management practices include disposal procedures, though regular waste is rare at the Central EPSS and its branches. However, disposal may occur for expired Pfizer COVID-19 vaccines, conducted according to a schedule in the presence of the controlling body for transparency.

> *"At the national level, vaccine wastage is rare, and I can even say that it does not exist… There is not much regular vaccine wastage at branches as well. However, there is some wastage around the COVID section, and there is some wastage of Pfizer… If there is wastage, the vaccine will be disposed according to the schedule, along with other items.The disposal is done in the presence of the controlling body. The list of items to be disposed of is sent to this body and then a certificate for the disposal of the items will be sent back."*

National EPSS Nine hubs had waste removed by the facility, three by both the facility and the government waste collection agency, and one hub requires assistance from the supplying store for waste removal. The CH and 13 SCHs have scheduled waste removal, ranging from annually to biannually.

Annual forecast for vaccine needs at central and sub-central hubs

Centrally, the vaccine forecasting process, led by EPSS, involves predicting health protection needs depending on eligible population size and quantifying regular vaccinations based on annual vaccine requests from each regional hub. This entails compiling micro-plans from each woreda. Based on such forecasts, EPSS collaborates with UNICEF and other donors for procurement of supplies.

The Visibility of vaccine platform is utilized to monitor shipment timelines and vaccine clearance through UNICEF procurement. A focal person is designated to receive an advice document detailing anticipated arrival at the airport. A pre-permit report is obtained through the Ethiopian Drug and Food Authority, ensuring quality tests. Upon arrival, vaccines undergo physical inspection and are recorded on the Vitas system. Special situations like Central campaigns or emergency outbreaks prompt tailored responses, including micro-plans and distribution coordination.

At the regional level, vaccine forecasting is integrated into the vaccine micro-plan, conducted at the beginning of each year. This involves a detailed estimation of vaccine needs, syringes, and other supplies, with agreements made with the Ethiopian Pharmaceuticals Supply Service (EPSS) to facilitate the plan.

EPSS then provides vaccines to health institutions based on the forecast, ensuring a predetermined schedule that aligns with refrigerator capacity. Additionally, training is conducted at the regional level to empower woredas (districts) on proper forecasting methods, calculating vaccine wastage, and requesting reserve vaccines.

> *"At the very beginning while we conduct our vaccine micro-plan, we forecast our vaccine needed for that specific year. We forecast how many vaccines, how many syringes and other supplies do we need for that year in our micro-plan then we agree with EPSA on our plan. All woredas and Health facilities also conduct their own vaccine forecast. So, using that vaccine forecast EPSA provide vaccine for each health facilities in predetermined schedule. For example, all Health facilities need a vaccine for only one month based on their refrigerator capacity, so EPSA provide the vaccine to them every month." KII, Regional EPI focal*

Regional participants highlighted challenges in vaccine forecasting, including the lack of recent census reports, conversion factor issues, and security concerns. In cities like Addis Ababa, population discrepancies due to rural to urban migration or internal displacement also pose challenges. The transition from central distribution to distributing vaccines to each health facility further improved accessibility, but forecasting remains a challenge.

"The denominator issue challenges us; the conversion factor challenges us to get the actual number of eligible groups to make proper forecasting. The other issues are security and budget problems hindering us from travel to the villages or home to home in some areas to get the actual number of pregnant mothers and eligible children" KII, Regional EPI focal.

4.2 Immunization supply chain operations and management at lowest distribution (LD) and service provision (SP) levels

Temperature management at lowest distribution and service provision level

At regional levels, 72% of institutions in Sidama lack functional refrigerators with a 30-Day Temperature Record (30DTR), while in SNNP and Southwest Ethiopia, the figures are 59 % and 56%, respectively. All institutions across the regions have very few refrigerators with proper temperature control. None of the refrigerators were functional in 50% of Sidama's institutions, 24 % in SNNP, and 22.2% in Southwest Ethiopia. Additionally, all health institutions in Amhara, Dire Dawa, Harari, and Sidama lack refrigerators or freezers with computerized temperature monitoring systems (CTMS) (Annex Table 1).

Of 30 health institutions with CTMS, 17% reported that it does not display temperature trends. Specifically, 8% of HCs, 67% of HPs, none of the hospitals, and 15% of WorHOs reported this issue (Table 4.6). To reduce vaccine wastage due to storage issues, institutions in some regions conduct daily and monthly temperature checks during performance reviews.

Table 4.6: Temperature management of vaccine storage equipment by health institutions

Variables		Health Institution type				
		Health Center n(%)	Health Post n(%)	Hospital n(%)	WorHO n(%)	Total n(%)
Computerized Temperature Monitoring System (CTMS) display temperature trends	Yes	11 (92)	1(33)	2(100)	11(85)	25(83)
Downtime incidents affecting storage conditions during last 12 months	Yes	8 (8)	6(9)	0(0)	11(14)	25(10)
Number of refrigerators/freezers with a Computerized Temperature Monitoring System	None	89(88)	61(94)	10(83)	67(85)	227(88)
	One	9(9)	4(6)	2(17)	7(9)	22(9)
	Two	3(3)	0(0)	0(0)	3(4)	6(2)
	Three	0(0)	0(0)	0(0)	2(3)	2(1)

WorHO=Woreda Health Office

Refrigerator/Freezer and insulated container storage capacity at lowest distribution and service provision levels

To assess cold chain storage adequacy, facilities calculate vaccine storage needs and their existing cold chain equipment capacity. The average (SD) storage capacity for vaccines positive (2-8 Degree Celsius) was 1434 (11243) liters in 286 facilities. The average (SD) storage capacity of vaccines negatives (-25 to -15 Degree Celsius) was 538 (5732) liters in 286 facilities. Average storage capacity for cool-packs, ice-packs, and coolant packs, was 593 (7580), 141 (1446), and 733 (8913), respectively for these facilities.
The average net vaccine storage capacity of refrigerators was 176 liters (n=254 institutions). The average net storage capacity of cool water-packs in refrigerators and icepacks in freezers was 16 and 12 liters, respectively. Freezers had an average net vaccine storage capacity of 16 liters (n=253). The average (n=286) net storage capacity of insulated containers 16 liters (57).Dry goods capacity was assessed in 50 WorHOs, with mean gross and net storage capacities of 34 (57) cubic meters and 20 (37) cubic meters, respectively.Dry goods storage capacity was recorded in 89 WorHOs, averaging 11243 (29341) liters (Annex Table 2).

Utilization of available capacity at lowest distribution and service provision levels

Vaccine storage and supply in health institutions are influenced by factors such as facility type, storage method, transportation mode, and insulated containers, leading to disparities between higher and lower-level institutions both in quantity and quality. Challenges include insufficient active storage devices and reliance on supplying stores, necessitating improvements for vaccine effectiveness and safety. Active storage methods are less common among facilities and WorHOs. Among the 286 health institutions, only 42 (15%) utilize long-term passive storage devices for vaccines. The distribution of long-term passive storage users among HCs, HPs, hospitals, and WorHOs is 16 (15.8%), 8 (9.6%), 2 (16.7%), and 16 (17.9%) respectively. A similar proportion (15.8% to 17.8%) of HCs, hospitals, and WorHOs adopt long-term passive storage, while only 9.6% of HPs do so, possibly explaining the non-significant association test. About 42% institutions retrieve vaccines from the supplying store.

Transportation and storage capacity challenges disproportionately affect pastoralist regions due to limited transportation options and the nomadic nature of the population. Health workers often trek long distances on foot in harsh weather to reach remote health facilities

Vaccine storage and management practices are vital for the success of the vaccine supply chain, ensuring availability, potency, and safety, thereby protecting individuals and communities from vaccine-preventable diseases. Out of 240 institutions, 70% arrange vaccine stock by type, while 139 out of 239 institutions (58%) organize stocks by lot and expiry date (EEFO).Variations exist, ranging from 29% for HPs to 83% for hospitals, leading to a significant association between institutions and responses.

Very few institutions (9.86%) reported vaccine storage emergencies, with hospitals having none, followed by HPs (9.64%), HCs (13.13%), and WorHOs (7.78%). About 89% of institutions took appropriate actions for vaccine storage emergencies; all WorHo taking the right steps, followed by HPs (87.50%) and HCs (84.62%). Only 9.7% of institutions experienced downtime incidents affecting storage conditions in the last 12 months (Table 4.7).

Proportions of institutions in Somali (86%), Dire Dawa (75%), Harari (66.67%), Sidama (40%), and Benishangul (40%) regions didn't arrange vaccine stocks by type. Likewise, Harari (100%), Dire Dawa (87.5%), Somalia (85.71%), and

Sidama (70%) regions didn't organize it by lot and expiry date (EEFO). However, 80% of institutions in Addis Ababa and Benishangul arranged by EEFO (Annex Table 3).

Key informants report that vaccine storage involves categorization by vaccine types, utilizing designated compartments within the refrigerator for separate storage by trained staff. Temperature regulation occurs twice daily throughout the storage process to maintain the cold chain.

> *"We keep safety of vaccine according to cold chain management systems for example: which antigens are heat /cold sensitive and assigning place accordingly the fridge compartments and fridge tag monitoring systems twice a day and if high arm maintaining, by doing shake test method we can evaluate the antigens in the facility. In our facility there is a selected room for only cold chain and locked to protect untrained professionals."* KII, HCEPI Focal_SD

Maintenance and repair of cold chain equipment at lowest distribution and service provision levels

Some partners offer training on open vial policy, with a focus on high-dose vaccines like measles and BCG.Critical aspects include established policies, guidelines, and temperature monitoring as key performance indicators. Some regions prioritize daily and monthly temperature checks during performance reviews to prevent waste due to inadequate temperature monitoring.

> *"There is an open vial policy, and we give training on that. For example, there are vaccines that have a high dose, like measles and BCG. For that, there are policies, guides, and temperature monitoring, which are critical issues and key performance indicators."* KII, National level partner

Table 4.7: Vaccine storage management practice by health facility type

		Health Institution type				
		Hea lth centre n(%)	Health Post n(%)	Hospital n (%)	WorHO n(%)	Total n(%)
Vaccine stocks arranged by vaccine type	Yes	73(73)	25(46)	10(83)	60(82)	168(70)
Vaccine stocks arranged by lot and expiry date	Yes	61(61)	16(29)	10(83)	52(72)	139(58)
Vaccine stora ge emergencies in the last 12 month	Yes	13(13)	8(10)	0(0)	7(8)	28(10)
Appropriate actions taken during the most recent vaccine storage emergency	Yes	11(85)	7(88)	NA	7(100)	25(89)
WorHO=Woreda Health Office, NA: Not Applicable						

Maintenance and repair of cold chain equipment at lowest distribution and service provision levels

Effective immunization supply chain management relies heavily on building maintenance. However, of 285 institutions, only 30 (10.5%) had a documented schedule for preventive maintenance tasks. Thirty-one (11%) facilities had records on maintenance work carried out and only 27 (9.5%) had a documented procedure for reporting faults or damage in buildings.

Over the past 12 months, repairs were conducted on only 60 (21%) of institutions' buildings. The government was primarily responsible for maintaining the buildings in 118 (41.4%) institutions, with all hospitals reporting no maintenance or repair-related work on their buildings. About 90% of institutions in Sidama, Benishangul, Gambella, Oromia, Somali, and southwest regions, and over 75% in Afar, Amhara, Dire Dawa, and Harari, lacked a documented preventive maintenance schedule for buildings. Table 4.8 and Annex Table 4 provide details on maintenance and repair practices of buildings at the LD and SP levels by facility type and region, respectively.

Table 4.8: Practice of maintenance & repair of buildings at the LD and SP level.

		Health Institution type				
		Health Center n(%)	Health Post n (%)	Hospital n (%)	WorHO n(%)	Total n(%)
Vaccine stocks arranged by vaccine type	Yes	73(73)	25(46)	10(83)	60(82)	168(70)
Vaccine stocks arranged by lot and expiry date	Yes	61(61)	16(29)	10(83)	52(72)	139(58)
vaccine storage emergencies in the last 12 month	Yes	13(13)	8(10)	0(0)	7(8)	28(10)
Appropriate actions taken during the most recent vaccine storage emergency	Yes	11(85)	7(88)		7(100)	25(89)

WorHO=Woreda Health Office

Maintenance and repair of cold chain equipment at lowest distribution and service provision levels

Majority of institutions, 214(82.95%), lacked a documented schedule for preventive maintenance of refrigeration equipment. The availability of such a schedule varied by type of health institution, HPs with the highest percentage (95.45%) without a schedule, followed by HCs (86.14%), WorHOs (70.89%), and hospitals (66.67%).

Of the 258 institutions, 207 (80.23%) reported no repairs on refrigeration equipment in the past 12 months. Repair services differed among institutions, most HCs (87.13%) and HPs (90.91%) not having any repair services (Table 4.9). The government primarily maintains the facility's refrigeration equipment, followed by the facility itself, with contributions from other actors. Only seven (20%) institutions kept records of preventive maintenance work (n=35). The percentage of those with records varied by type,

ranging from 11% for HCs to 50% for hospitals. This indicates a common problem among health institutions regarding documentation of work performed.

At lower levels, challenges persist, including the absence of refrigerators in health posts, shortages of spare parts, and skilled professionals for refrigerator maintenance. This jeopardizes vaccine viability as they cannot remain outside the fridge for more than 24 hours, hindering last-mile delivery goals. Additionally, lack of skilled personnel for immediate refrigerator maintenance can result in delayed repairs, potentially causing vaccine damage due to prolonged notification processes from institutions to higher levels.

Table 4.9: Preventive maintenance and repair of refrigerators by health institution type at the

Variables		Health Institution type				
		Health Center n(%)	Health Post n (%)	Hospital n (%)	WorHO n(%)	Total n(%)
Availability of documented schedule(s) of the preventive maintenance tasks for building	Yes	10 (10)	10(12)	0(0)	10(11)	30(11)
Availability of records of the preventive maintenance work carried out	Yes	9 (9)	10(12)	0(0)	12(13)	31 (11)
Presence of documented procedure for reporting fault/damage in the buildings?	Yes	10 (10)	6 (7)	1 (8)	10 (11)	27 (10)
Building re paired in the past 12 months	Yes	19 (19)	17 (20)	1 (8)	23 (26)	60 (21)
WorHO						

Stock management equipment at lowest distribution and service provision levels

Effective vaccine stock management is essential for ensuring a smooth vaccine supply chain. Proper management minimizes the risk of stock-outs or wastage (Figure 4.1). Of 285 institutions, 98 (34.39%), do not refill vaccine stocks regularly. This tendency is more pronounced in HPs, likely due to their lack of mandate for regular replenishment. Furthermore, 49 (17.19%) of the institutions do not submit vaccine requests, HPs being less likely to do so, as it is not part of their SOP.

Out of the 236 institutions that submit vaccine requests, 175 (74.15%) do not do so electronically. However, among those that do, 192 (81.36%) keep records of vaccine requests. Hospitals have the highest proportion of records (83.33%), followed by WorHO (93.98%), HCs (94.68%), and HPs (31.91%). Records of vaccine requests are lowest in HPs (31.91%), followed by hospitals (16.67%), WorHO (6.02%), and HCs (5.32%).

Of 285 institutions, 113 (39.65%) lack vaccine stock records, with a significant majority (92.77%) of HPs falling into this category. In addition, 158 of 172 institutions (91.86%) with vaccination stock records reported not having computerized records.

Regarding vaccine receipts, the majority (61.05%) maintain records. HCs lead in this aspect (81.2%), followed by WorHOs (83.15%), hospitals (66.67%), and HPs (12.05%). Physical damage receipts for vaccines are minimal, with 98.84% of institutions reporting zero instances; Only WorHOs reported such receipts.

Regarding documented maximum stock levels for each vaccine, over three-fourths (76.16%) of institutions reported having them. HCs have the highest proportion (78.82%), followed by WorHOs (74.32%), hospitals (71.43%), and HPs (66.67%).

Stock management involves various steps such as forecasting, reserving 25%, making emergency requests, and updating ledger stock balances to minimize wastage. Health institutions submit supply requests to Woreda, sometimes utilizing direct communication technologies. A standardized vaccine request form is necessary for sustainable inventory tracking, procurement, storage, administration, and billing. Monthly requests are based on eligible children or population members in each kebele, ensuring accurate refill amounts with a mandatory ending balance and considering existing or previous month's stock for effective resource utilization.

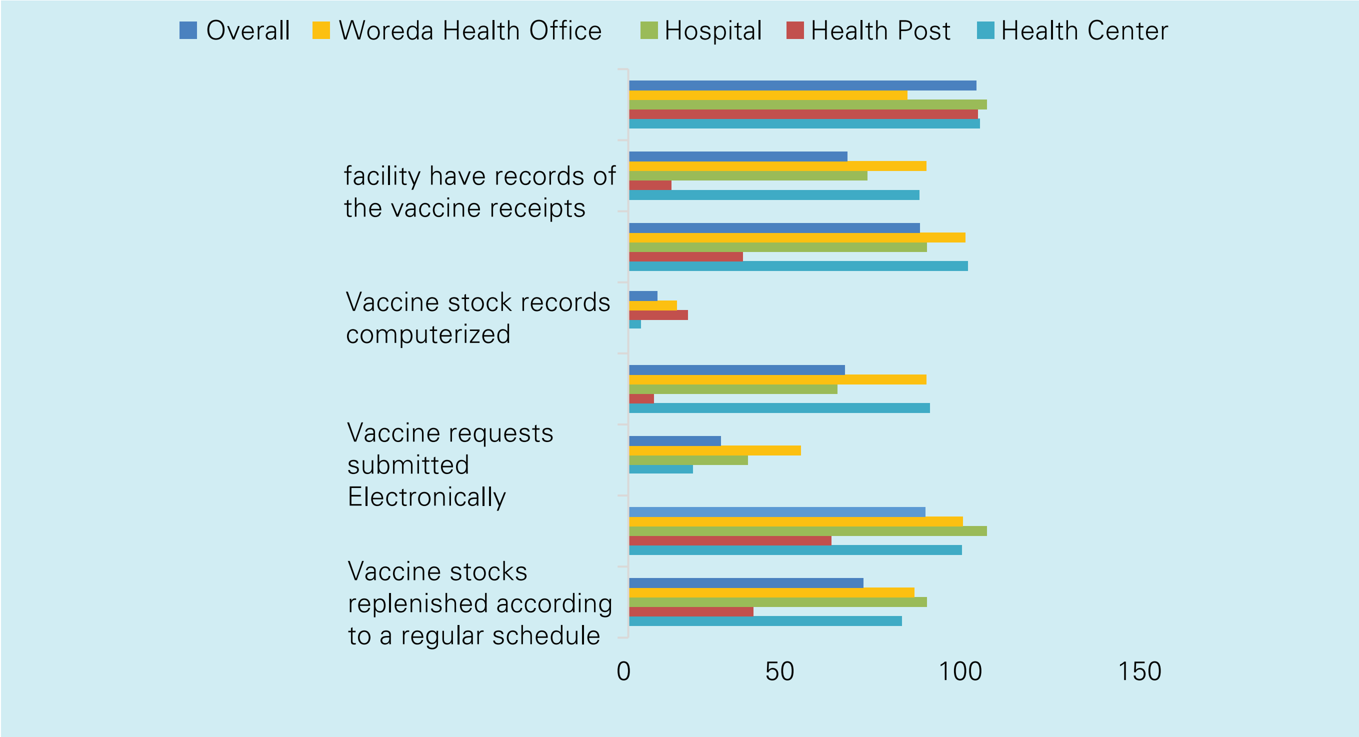

Figure 4.1: Vaccine stock record management practice of lower and Service Provision levels by health institution types.

Table 4.10 displays the vaccine stock management system and distribution by type of health institution. Vaccine stock management for vaccine needs per receiving store was highest among WorHOss (76%), and HPs (70%). Vaccine quantity distribution deviated from allocation in the following order: HCs (95.45%), HPs (95.24%), WorHo (84.21%), and hospitals (75%). The majority of those evaluated health

institutions (84.85%) maintain complete distribution and allocation records. Completeness of distribution and allocation records varies from 68.42.00% for WorHo to 95.24% for HPs. The stock management system indicates whether vaccine stock levels fall between the established minimum and maximum, ranging from 57% for hospitals to 73% for health posts.

Table 4.10: Stock management system by facility type at the LD and SP level.

Variable		Health Institution type				
		Health Center	Health Post	Hospital	WorHO	Total
Stock management system show vaccine needs per receiving store	Yes	22 (59)	21(70)	4(67)	19(76)	66(67)
Stock management system show the quantities distributed against those allocated per supply period for each receiving store	Yes	21(93)	20(95)	3(75)	16(84)	60(91)
Complete distribution and allocation records	Yes	20(91)	20(95)	3(75)	13(68)	56(85)
Stock management system show vaccine stock levels are between the established minimum and maximum	Yes	42 (69)	38(73)	4(57)	32(55)	116(65)

Stock records at lowest distribution and service provision levels

Table 4.11 and Annex Table 5 presents stock records at lowest distribution and service provision levels at facility and regional level, respectively. Accurate vaccine stock records are vital for efficient immunization supply chain management, ensuring availability, preventing stock-outs, optimizing storage and distribution, minimizing wastage, and facilitating monitoring and evaluation of immunization programs. Comprehensive records aid in planning and forecasting vaccine needs. All hospitals maintained diluent stock records, with 88.2% of HCs and 75.0% of HPs also having diluent stock records. Dilutant stock records were available in 144 (88.9%) institutions, with regions such as Addis Ababa, Gambela, Harari, Sidama, and Southwest showing 100% availability. Well-organized stock records were observed in 119 (67.23%) institutions, with records securely kept in 103 (70.7%) institutions. HPs and hospitals kept complete stock records, followed by WorHOss (89.83%) and HCs (81.58%). Vaccine stock record availability by regions shows Addis Ababa and Dire Dawa Cities, had fully completed records.

The majority of health institutions (67.23%) reported having well-organized vaccine stock records, which include records filled by vaccine type and transaction type. HPs had the highest proportion (72.0%), followed by HCs (67.21%), WorHOs (67.80%), and hospitals (28.57%).

Retention of vaccination stock records for a minimum of three years was required, with 55% of HCs, 46.0% of HPs, 45.8% of WorHOs, and 28.6% of hospitals meeting this requirements. Over the last 12 months, vaccine storage emergencies occurred in 28 (9.86%) institutions, with records of unopened vial wastage available in 89 (31.23%) institutions. Additionally, 103 (70.07%) of health institutions stored their vaccine stock records securely, such as in a locked filing cabinet or cap board, with a high proportion (76.92%) among health posts. Distribution and allocation records were updated monthly for 54 (81%) of the institutions, with all hospitals adhering to monthly updates (Table 4.11).

Table 4.11: Stock record arrangements at lower and service provision levels by health institution type at the LD and SP level.

Variable		Health Institution type				
		Health Center n(%)	Health Post n(%)	Hospital n(%)	WorHO n(%)	Total n(%)
Vaccine stock records well organized	Yes	41(67)	36(72)	2(27)	40(68)	119(67)
Stock management system show if vaccine stock levels are between minimum and maximum	Yes	42 (69)	38(73)	4(57)	322(55)	116 (65)
Stock records complete	Yes	62(81.8)	4(100)	5(100)	53(90)	124(86)
Presence of diluent stock records	Yes	67(88.2)	3(75	5(100)	52(88)	127(88)
Vaccine stock records stored in a secure place	Yes	33(68.7)	30(77)	3(43)	37(70)	103(70)
How often are the distribution and allocation records updated?	Annually	1 (5)	0 (0)	0(0)	0(0)	1(2)
	Bi-annual.	1(5)	0 (0)	0(0)	0(0)	1(2)
	Daily	3(14)	1(5)	0(0)	0(0)	4(6)
	Monthly	15(68)	19(90)	4(100)	16(84)	54(82)
	Quarterly	0 (0)	0(0)	0(0)	1(5)	1(2)
	Weekly	2(9)	1(5)	0(0)	2(11)	5(8)
	Total	22	21	4	19	66

Stock management challenges vary across facility levels, with lower-level institutions and newly established regions experiencing technology shortages causing submission delays. A shortage of ledger books at the woreda level significantly affects documentation and the effectiveness of stock management. Health posts in regions like Afar resort to providing copied versions of ledgers, which are considered inadequate. Stock outs of vaccines such as OPV, Rota, and BCG from EPSS at the country level are also noted, possibly linked to a shortage of cold vans and related issues.

"At the health center level, they have a standardized ledger book. They don't have it at the health post level, so we have given them a copy of the ledger book. We don't have enough ledger books, so we are making copies for health posts. There is no proper monitoring and evaluation of the EPI process." KII, Regional EPI focal

"We register vaccine supplies on stock management ledger book monthly as collected from woreda health office and distribute it to health posts of our catchment even it is currently interrupted due not trained/ experienced new EPI focal." KII, HC_ EPI focal

Stock out challenges in vaccine distribution are often linked to transportation shortages, such as the inability to transport enough vaccines using motorcycles. Additionally, newly appointed EPI focal persons without prior training can lead to inadequate stock management in some HPs, affecting vaccination service provision. Bordering institutions also face challenges due to mobile populations traveling to neighboring countries, causing sporadic dropouts and artificial stock outs due to fluctuating population flow.

"The other challenge is those of people who are dropout from the program, they are not in the camp. Maybe they are going to South Sudan, or they are going to the border or else they are going to gold mining. This dropout rate is not going to be traced on time. They came after nine months something, something." KII, GM HF EPI focal

Distribution of vaccines and dry goods at lower distribution and service provision levels

In many regions, health institutions receive vaccines directly from EPSS regional hubs or supply stores at woreda rather than collecting themselves. All HCs in Addis Ababa, 91% in Gambella, 83.3% in Sidama, 72.2% in South West, and 71.4% in Harari have vaccines delivered directly to their facilities.

Overall, 56% of HCs receive vaccines from WorHO, while 44% collect vaccines from the supply store themselves. About 20% of institutions use road vehicles for vaccine collection or distribution, with respective percentages of 20%, 25%, and 41% for HCs, hospitals, and WorHOs. HPs, lacking vehicles, can only accept deliveries. Health centers and hospitals may use vehicles to collect from WorHOs, while WorHOs use vehicles for distribution to institutions. Despite the scarcity of road vehicles, 34 (26.2%) institutions reported that roads were either in poor condition or unusable year-round (Table 4.12).

Table 4.12: Distribution of vaccines at lower distribution and service provision levels by health institutions type in Ethiopia.

		Health Institution type				
		HC	HP	Hosp.	WorHO	Total
Collect vaccines from a supplying store	Yes	44(44)	31(37)	4(33)	40(44)	119(42)
Road vehicles used to collect, distribute vaccines or to provide	Yes	20 (20)	1(1)	3(25)	37(41)	61(21)
Access road in good condition	Yes	14 (70)	1(100)	2(67)	28(76)	45(74)
Road can be used year -round	Yes	13(65)	1(100)	3(100)	26(70)	43(71)

HC = HP Hosp.WorHO

The challenge of transportation and inadequate infrastructure not only hinders last-mile delivery implementation but also leads to EPI service interruptions, particularly in some districts during the rainy season.

Key informant interviews revealed that the ultimate goal of last-mile delivery remains unfulfilled in many health institutions due to various factors, primarily lack of storage facilities. However, transitioning to last-mile delivery is recognized as a significant improvement in the immunization supply chain. Direct delivery by EPSS to health institutions, achieving 100% effectiveness in certain regions like Sidama and Addis Ababa, enhances vaccine distribution efficiency. Shifting from monthly distribution to on-demand, proximity-based, and scheduled delivery further streamlines the process, reducing the need for intermediaries.

> *"Ethiopia's immunization supply chain has undergone a transformation over the years. In the past, it was a vertical system where resources were distributed from health protection to region, from region to zone, and from zone to district. However, according to the Ethiopian pharmaceutical master plan, resources should go through EPSS in a coordinated manner… resources will be delivered to health facilities in two ways from 2018. One is that our EPSS branches will deliver direct resources to certain health facilities as EPSS branches in the districts. After that, the resource is transferred to the district, for example, if a district has four or five health centers. Then the health centers will distribute to health posts. The health institutions that EPSS delivers directly use their own."* KII, Partner National level

In areas where last-mile delivery is not practiced, WorHOs resort to using ambulances and/or motorcycles for supply delivery to health institutions within their catchments. However, issues such as the absence or malfunction of vehicles, coupled with inadequate road infrastructure, especially during the rainy season, pose significant challenges to effective vaccine distribution management.

> *"As I have said we are bringing vaccines by using motorcycle and we may not bring sufficient number of vaccines to be maintained in the stock. The stock may be maintained for not more than two weeks. Had it been a vehicle we may maintain for longer period of time (for a month or more)."* KII, AMH HF EPI focal

Vaccine management and quality at lower distribution and service provision levels

Overall, the quality of vaccine management and handling in Ethiopia is generally good. Approximately 77.4% of facilities had a shake test guideline and procedure in place. However, two out of five hospitals and 13 out of 48 HCs lacked this guideline and procedure.

Safely utilizing multi-dose vials is crucial to prevent contamination, wastage and potential harm to individuals. Adherence to the multi-dose vial policy is therefore essential. Among the 84 HFs, 14 (16.6%) did not have a multi-dose vial policy in place (Figure 4.2). In terms of wastage minimization, the first action taken by all institutions was reducing the wastage rate by sharing vaccines between nearby health facilities.

Maintaining a high client flow is another crucial strategy for minimizing vaccine wastage. For BCG and measles, opening these vaccines twice a week has been effective in applying strategic vial opening.

> *"…… Recently, after discussion has been conducted, vaccine wastage has been reduced from 10% to 5%. The expected BCG is 50%, which means if we have 10 children, we can dilute the BCG vaccine. But now we arrange a nearby health post, which is about 30 minutes away. They were coordinated to work together to give vaccines by traveling to one place. …which is important to reduce vaccine wastage for BCG and measles."* KII, HC_EPI focal _ BG

There have also been some institutions that indicated no wastage because of the high flow of clients during the appointed day.

> *"There is no vaccine wastage because we have a high client flow. Wastage may happen only for two vaccines when we open BCG and measles vaccines."* KII, HC_EPI focal DD

Adhering to recommended protocols for vaccine storage, handling, preparation, and administration helps maintain the potency and effectiveness of vaccines, minimizes the risk of contamination or adverse events, and ensures that individuals receive the intended protection.

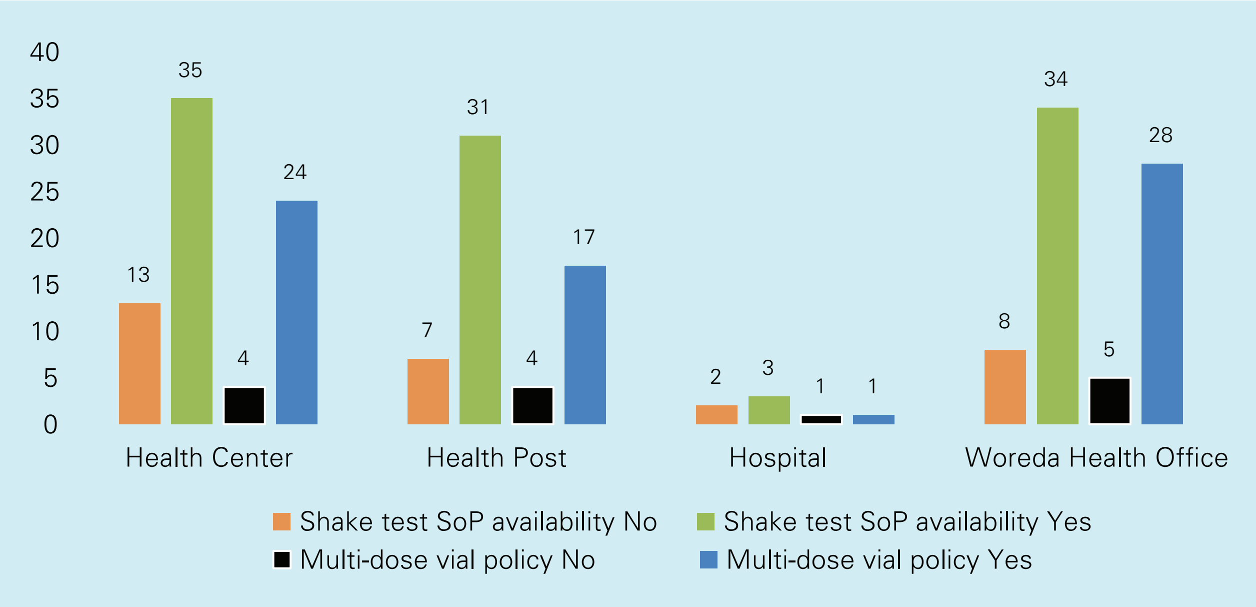

Figure 4.2:. Availability of shake test and multi-dose vial policy/procedure by health facility at the LD and SP levels in Ethiopia.

Almost all institutions across all regions correctly used appropriate diluents to reconstitute freeze-dried vaccines, except for two institutions—one in Amhara and the other in Oromia (Annex Table 6). Approximately 20% of institutions in Amhara and Harari, and 18.92% in Oromia, were using expired vaccine vials. However, none of the institutions in Addis Ababa, Dire Dawa, Gambella, or southwest used such expired vaccine vials.

Eighty-six percent of institutions had no expired vaccine vials, with 93.33% of HCs, 87.18% of HPs, 66.67% of hospitals, and 80.39% of WorHO having no expired vials. Additionally, 82% of institutions had no vials with Vaccine Vial Monitors (VVMs) beyond the discard point, with the figures being 86.36% for HCs, 82.05% for HPs, 66.67% for hospitals, and 78.00% for WorHOs. Furthermore, 74% and 78% of the respondents has knowledge on how to conduct and when to conduct shake test respectively. However, 38% of the health institutions had a shake test practice, with 93% of HCs, and 36% of hospitals having shake-test practice. (Table 4.13)

Table 4.13: Vaccine management of the lower and service provision levels by health institution type in Ethiopia 2023.

		Health Institution type				
		HC, n (%)	HP n (%)	Hosp. n (%)	WorHO, n (%)	Total n (%)
The correct diluents are used to reconstitute freeze dried vaccines	No	0 (0)	0 (0)	0 (0)	2 (5)	2 (2)
	Yes	40 (100)	32 (100)	3 (100)	35 (95)	110 (98)
Any expired vaccine vials to be used.	No	42 (93)	34 (87)	2 (67)	41 (80)	119 (86)
	Yes	3 (7)	5 (13)	1 (33)	10 (20)	19 (14)
Vaccine Vial Monitors (VVMs) beyond the discard point	No	38 (86)	32 (82)	2 (67)	39 (78)	111 (82)
	Yes	6 (14)	7 (18)	1 (33)	11 (22)	25 (18)
Shake test	No	14 (27.5)	8 (36)	1 (33)	7 (12)	31 (21)
	Yes	37(72.5)	14(64)	2 (37)	50(88)	117(79)
Responsible staff know when to conduct the shake test	No	14 (14)	36 (43)	5 (42)	9 (10)	67 (22)
	Yes	87(86)	47(57)	7 (58)	81(90)	235(78)
Responsible staff know how to conduct the shake test	No	20 (20)	40 (48)	6 (50)	10 (11)	78 (26)
	Yes	81(80)	43(52)	6(50)	80(89)	224(74)

HC=Health Centres, HP=Health Posts, Hosp=Hospitals, WorHO=Woreda Health Office

Waste management and handling of syringes after use at lower distribution and service provision levels

The number of safety boxes in stock serves as a critical indicator of safe injection practice in health institutions. Among 203 respondents, 197 health institutions utilize safety boxes to store used syringes, with an average of 26.73 (SD = 7.55) safety boxes in stock. Safety boxes in stock ranges from 1 to 58, with a 95% CI (25.67 - 27.79).

Proper utilization of safety boxes is essential for preventing needle stick injuries to both providers and the community. However, only 14% of institutions admitted to having filled safety boxes alongside general waste on their premises, with hospitals reporting none. In terms of regional distribution, 41% of the safety boxes are filled with general waste in Afar, while all other regions recorded much lower values. The most common type of syringe used for injections was the auto-disabled syringe (ADS), accounting for 76.09% (n=138). All institutions in Addis Ababa and Dire Dawa used only the AD syringe type for injection, the most widely used type in all regions.

The most common type of syringe used for dilution was the standard disposable syringe, which accounted for 138 (92%) of health institutions. All institutions in Addis Ababa, Benishangul, Gambella, SNNP, and Sidama used the standard disposable syringe type for vaccine dilution.. Most health workers, 138 (89.13%), did not recap syringes after use. Similarly, 100 (61.00%), did not de-fang syringes immediately

after use. Most health workers place used syringes into safety boxes immediately after use. Health workers in two HPs in Harari, two HCs in SNNP, one HP and HC in Sidama, one HC in South West, and one HP in Amhara recap syringes after use. In the remaining regions, none of the health workers return needles after use (Table 4.14 and Annex Table 7).

Table 4.14: Vaccine injection practice by health institution at lower distribution and service provision levels.

Variables		Health Institution type				
		HC, n(%)	HP, n (%)	Hosp, n(%)	WorHO, n (%)	Total, n(%)
Types of syringes used for injections	ADS	33 (73)	29 (74)	1 (33)	42 (82)	105 (77)
	ADS and SDS	12 (27)	9 (23)	1 (33)	8 (16)	30 (22)
	SDS	0 (0)	1(3)	1 (33)	1 (2)	3 (2)
Types of syringes used for dilution	Other	0 (0)	1 (3)	0 (0)	1 (2)	2 (1)
	RUP	4 (9)	4 (11)	0 (0)	0 (0)	8 (6)
	SDS	40 (89)	34 (87)	3 (100)	50 (98)	127 (92)
	SDS and RUP	1 (2)	0 (0)	0 (0)	0 (0)	1(1)
Do health workers recap syringes after use?	No	38 (84)	36(92)	3 (100)	46 (90)	123 (89)
	Yes	7 (16)	3 (8)	0 (0)	5 (10)	15 (11)
Health workers de-fang syringes practice immediately after use?	No	20 (63)	17 (57)	1 (50)	23 (64)	61 (61)
	Yes	12 (38)	13 (43)	1 (50)	13 (36)	39 (39)
Health workers place used syringes into safety boxes immediately after use	No	1 (2)	1 (3)	0 (0)	0 (0)	2 (1)
	Yes	43 (98)	38 (97)	3 (100)	50 (100)	134 (98)

ADS=Auto - disabled syringe, SDS=Standard disposable syringes, RUP = Reuse prevention syringe, HC = Health Centres, HP = Health Posts, Hosp = Hospitals, WorHO = Woreda Health Office

HPs and WorHOs performed less than 50%. Nevertheless, 80 (52.3%) of the institutions reported securing the waste storage areas from pests. Only 130 (45%) of the institutions stored waste in bins, with HCs and hospitals demonstrating better performance at 66 (65%) and 28 (83%), respectively. However, WorHOs performed poorly at 29% due to their limited involvement in onsite service delivery. Among those using bins, only 54 (41%) employed color-coded bins for different waste types, indicating ongoing challenges in segregation and proper waste disposal. Additionally, 51 (17.9%) of health institutions had overfilled waste bins, posing risks of hazardous or infectious waste spills, endangering the health of both health workers and the community.

Waste collection from health institutions typically occurs either daily or weekly, with a combined frequency of 86%. Hospitals demonstrate higher daily collection rates at 42%, while WorHOs exhibit poorer performance, with only 18% conducting annual collections. Given the number of institutions reporting non-isolated storage, lack of bin usage, and uncovered waste containment, frequent daily or weekly collection significantly aids the system (Table 4.15).

Table 4.15: Waste onsite storage by health institution type at lower distribution and service provision levels

		Health Institution type				
		HC, n(%)	HP, n(%)	Hospital n(%)	WorHO, n (%)	Total, n(%)
Waste stored in a segregated area	No	31 (31)	45 (54)	3 (25)	53 (60)	132 (46)
	Yes	70 (69)	38 (46)	9 (75)	36 (40)	153 (54)
Waste storage area secured from pests	No	36 (51)	23 (60)	5 (56)	16 (44)	80 (52)
	Yes	34 (48)	15 (39)	4 (44)	20 (56)	73 (48)
Waste storage area covered	No	42 (60)	27 (71)	5 (56)	21(58)	95 (62)
	Yes	28 (40)	11 (29)	4(44)	15 (42)	58 (38)
Waste stored in bins	No	35 (35)	55 (66)	2 (17)	64 (71)	156 (54)
	Yes	66 (65)	28 (34)	10 (83)	26 (29)	130 (45)
Bins colour-coded for each type of waste	No	27 (41)	27 (96)	2 (20)	20 (77)	76 (58)
	Yes	39 (59)	1 (4)	8 (80)	6(23)	54 (45)
Frequency of waste removal from the facility	Annually	0 (0)	0 (0)	0 (0)	2 (18)	2 (4)
	Daily	9 (38)	1 (33)	3 (43)	0 (0)	13 (29)
	Monthly	0 (0)	0 (0)	1 (14)	3 (27)	4 (9)
	Weekly	15 (63)	2 (67)	3 (43)	6 (54)	26 (58)
Bin liners in bins	No	41 (62)	25 (89)	5 (50)	22 (85)	93 (71)
	Yes	25 (38)	3 (11)	5 (50)	4 (15)	37 (28)
Over-filled waste storage area with waste	No	75 (74)	68 (82)	10 (83)	81 (91)	234 (82)
	Yes	26 (26)	15 (18)	2 (17)	8 (9)	51(18)
Any used syringes, needles/vials on the floor	No	68 (68)	67 (81)	11 (92)	81 (91)	227 (80)
	Yes	32 (32)	16 (19)	1 (8)	8 (9)	57 (20)
Any unsealed bags, open boxes of waste in the waste storage area	No	72 (71)	67 (81)	12 (100)	77 (86)	228 (80)
	Yes	29 (29)	16 (19)	0 (0)	12 (13)	57 (20)
Any filled safety boxes together with the general waste	No	81 (80)	68 (82)	12 (100)	84 (94)	245 (86)
	Yes	20 (20)	15 (18)	0 (0)	5 (6)	40 (14)

HC,=Health Centres, HP=Health Posts, Hosp=Hospitals, WorHO=Woreda Health Office

Waste segregation practices vary across regions, with a significant portion of institutions in Harari and the Somali region not storing in segregated areas (71.4% and 60%, respectively). The storage areas lacked pest security in most institutions, particularly in Benishangul (100%), Sidama (85.7%), and Dire Dawa (88.9%). A majority of institutions in Afar (66.7%), SNNP (73.5%), and Sidama (88.9%) did not utilize waste bins. During assessment, used syringes, needles, or vials were found on the floor near 50% of institutions in Afar, 40% in Addis Ababa, 36.3% in Gambella, and 33.3% in southwest Ethiopia. Over 50% of institutions in all regions lacked color-coded waste storage bins for different types of waste, with more pronounced issues in SNNP (77.8%), Somalia (71.43%), and Dire Dawa (66.7%). Sixty-seven percent of institutions in Sidama and 25% in Afar disposed of their immunization waste monthly. Conversely, all institutions in Gambella, Harari, SNNP, and Somalia disposed of immunization waste weekly (Annex Table 8).

Disposal of immunization waste at lower distribution and service provision levels

According to WHO, used syringes should be classified as infectious waste and disinfected via non-incineration methods. For disposing of such waste, different waste management equipment systems are used. About 57% of institutions treat or dispose of immunization waste within the vicinity of their facility. Health centers and hospitals mostly dispose of on-site or treat, while WorHOs rarely do so (27%). Thirty-one percent of the institutions use incineration as the main waste management method. Health institutions' preferences for the type of waste management differ from facility to facility: 50% of hospitals use a combination of incineration and burial; only one WorHO uses a needle pit. However, although incineration is a popular method among the institutions, it is either not working or requires repair in 39 (32%) of the cases (Table 4.16). Immunization waste was not treated or disposed of on-site at 58.8% of SNNP institutions, 52% of Somalia, and 46% of Amhara sites. Only 40% of the assessed incinerators are operating effectively in the Afar, 50% in Benishangul, and 60% in Addis Ababa. The remnants either needed repairs or weren't functioning at all; in the other region, a large number of well-working incinerators were reported. At the time of assessment, the waste storage area was overfilled with waste in 50% of the institutions in Afar, 29.4% in SNNP, and 27.8% in south-west Ethiopia (Annex Table 9).

Table 4.16: Waste disposal practice of lower and service provision levels by health institutions type in Ethiopia 2023.

Variables		Health Institution type				
		HC, n(%)	HP, n(%)	Hosp n(%)'	WorHO, n (%)	Total, n(%)
Immunization waste is treated or disposed of on-site	No	10 (10)	46 (55)	2 (17)	65 (72)	123 (43)
	Yes	91 (90)	37 (45)	10 (83)	25 (28)	163 (57)
Type of waste management equipment used at the facility	Burial	2 (2)	8 (22)	0 (0)	7 (29)	17 (11)
	Burial and Needle pit	1(1)	1(3)	0 (0)	0 (0)	2 (1)
	Burial and Other	0 (0)	0 (0)	0 (0)	1(4)	1(1)
	Incineration	31 (34)	10 (28)	3 (30)	6 (25)	50 (31)
	Incineration and Burial	35 (38)	6 (17)	5 (50)	3 (12)	49 (30)
	Incineration and Burial and Needle pit	15 (16)	1(3)	2 (20)	2 (8)	20 (12)
	Incineration and Burial and Other	1(1)	0 (0)	0 (0)	0 (0)	1 (1)
	Incineration and Needle pit	1(1)	1(3)	0 (0)	2 (8)	4 (3)
	Incineration and Other	3 (3)	0 (0)	0 (0)	1 (4)	4 (3)
	Needle pit	0 (0)	0 (0)	0 (0)	1 (4)	1 (1)
	Other	2 (2)	9 (25)	0 (0)	1 (4)	12 (7)
Functional status of the incinerator	Not working	4 (5)	1 (6)	0 (0)	0 (0)	5 (4)
	Working However needs repair	25 (29)	4 (22)	1 (10)	4 (29)	34 (27)
	Working well	57 (66)	13 (72)	9 (90)	10 (71)	89 (69)

HC=Health Centres, HP=Health Posts, Hosp=Hospitals, WorHO=Woreda Health Office

Forecasting vaccine needs at lower distribution and service provision levels

Immunization supply chain forecasting is crucial for effective vaccine supply management. By considering factors such as population demographics, disease prevalence, vaccination schedules, and campaign plans, forecasting models can project the demand for vaccines over a specific time. Fifty-eight (20%) of health institutions reported that they do not forecast their vaccine needs. Nineteen (21%) of Woredas and 20 (20%) of health centers do not forecast their vaccine needs (Table 4.17).

When viewed by region, all institutions in Afar, Benishangul, Dire Dawa, and Harari forecast their vaccine needs. However, only 50% in the Sidama Region, 57.58% in SNNP, 72.22% in Southwest Ethiopia, and 77.65% in Oromia forecast their vaccine needs (Annex Table 10).

Of 125 health institutions, only 12 (9.6%) do not track the immunization supply chain performance. Among HCs, HPs, hospitals, and WorHOs, 6 (11.11%), 2 (14.29%), 1 (50%), and 3 (5.45%), respectively, failed to monitor the immunization supply chain performance. Majority, 194 (85.46%), of institutions have done forecasts for the previous year. The availability of vaccine forecasts for the previous year ranges from 6 (60.00%) for hospitals to 4 (90.14%) for WorHOs. Few health institutions (40.00%) use computerized systems to monitor vaccine forecast accuracy; HCs performed well (54%), while HPs performed poorly (27%) (Table 4.17).

Table 4.17: Immunization supply chain forecasting practice at the lower distribution and service provision levels by type of health institution

		Health Institution type				
		HC n(%)	HP n(%)	Hosp. n(%)	WorHO, n (%)	Total, n (%)
Availability of iSC guidance materials for facility managers	No	46 (46)	69 (83)	10 (83)	35 (38.89)	160 (56)
	Yes	54 (54)	14 (17)	2 (17)	55 (61.11)	125 (44)
Monitoring the (iSC) performance	No	6 (11)	2 (14)	1 (50)	3 (5.45)	12 (10)
	Yes	48 (89)	12 (86)	1(50)	52 (94.55)	113 (90)
Forecasting vaccine needs	No	20 (20)	17 (21)	2 (17)	19 (21.11)	58 (20)
	Yes	80 (80)	66 (80)	10 (83)	71 (78.89)	227 (80)
Method Used to forecast vaccine needs	Consumption & target methods	15 (19)	15 (23)	1 (10)	14 (19.72)	45 (20)
	Past consumption method	5 (6)	2 (3)	1 (10)	6 (8.45)	14 (6)
	Target population method	59 (74)	49(74.24)	8(80)	51(71.83)	167(74)
	Other method	1(1)	0(0)	0(0)	0(0)	1(0)
Having vaccine needs forecasts for all vaccines for current year	No	0 (0)	1 (2)	1 (10)	3 (4)	5 (2)
	Yes	80 (100)	65 (99)	9 (90)	68 (96)	222 (98)
Having vaccine needs forecasts for all vaccines for the previous year	No	12 (15)	10 (15)	4 (40)	7 (10)	33 (15)
	Yes	68 (85)	56 (85)	6 (60)	64 (90)	194 (85)
Accuracy of computerized system for monitoring vaccine forecast	No	6 (46)	8 (73)	4 (67)	18 (60)	36 (60)
	Yes	7 (54)	3 (27)	2 (33)	12 (40)	24 (40)
HC = Health Centres, HP = Health Posts, Hosp = Hospitals, WorHO=Woreda Health Office						

Annual work planning on iSC at the lower distribution and service provision levels

Most health institutions, 266 (93.33%), have an annual work plan, ranging from all hospitals to 77 (92.77%) HPs. On the other hand, 92 (35%) institutions budget their work plan activities, 25% for hospitals to 38% for HCs. Feedback was received by 95% of WorHOs, HPs (89.13%), HCs (86.96%), and hospitals (85.71%) (Table 4.18).

Table 4.18: immunization supply chain work plan at lower distribution and service provisions level by health institutions in Ethiopia 2023.

		Health Institution type				
		HC, n(%)	HP, n (%)	Hosp. n(%)	WorHO, n (%)	Total, n (%)
Facility have an annual work plan	No	4 (4)	6 (7)	0 (0)	9 (10)	19 (7)
	Yes	96 (96)	77 (93)	12 (100)	81 (90)	266 (93)
Activities of the work plan budgeted	No	60 (63)	53 (69)	9 (75)	52 (64)	174 (65)
	Yes	36 (38)	24 (31)	3 (25)	29 (36)	92 (35)
Implementation of the work plan on scheduled	No	12 (16)	11 (18)	2 (20)	12 (18)	37 (17)
	Yes	64 (84)	51(82)	8 (80)	55 (82)	178(83)
Facility have records of the latest visit	No	39 (46)	33 (42)	5 (42)	36 (46)	113 (44)
	Yes	46 (54)	46 (58)	7 (58)	42 (54)	141 (55)
Report immunization supply chain data	No	19 (19)	16 (19)	3 (25)	17 (19)	55 (10)
	Yes	81 (81)	67 (80)	9 (75)	73 (81)	230 (81)
HC=Health Centres, HP=Health Posts, Hosp=Hospitals, WorHO=Woreda Health Office						

Table 4.19: Immunization supply chain supervision at lower distribution and service provision levels by health institutions type.

		Health Institution type				
		HC, n (%)	HP, n(%)	Hospital, n (%)	WorHO, n (%)	Total, n (%)
Supervisor leave written feedback	No	6 (134)	5 (11)	1 (14)	2 (5)	14 (10)
	Yes	40 (87)	41 (89)	6 (86)	40 (95)	127 (90)
Facility reported immunization supply chain data to higher level	No	19 (19)	16 (19)	3 (25)	17 (19)	55 (19)
	Yes	81 (81)	67 (81)	9 (75)	73 (81)	230 (81)
Facility receive any feedback about its report during the past 12 months	No	33 (41)	33 (49)	5 (56)	29 (39)	100 (43)
	Yes	48 (59)	34 (51)	4 (44)	44 (60)	130 (56)
HC=Health Centres, HP=Health Posts, Hosp=Hospitals, WorHO=Woreda Health Office,						

Immunization supply chain performance monitoring at lower distribution and service provision levels

The majority of health institutions (71.64%) lacked a supply chain performance dashboard, hospitals being the worst; and their iSC performance not reviewed in a year for about half of them (Table 4.20). None of the institutions in Dire Dawa and Gambella had a supply chain performance dashboard, while such dashboards were available in all institutions in Harari and in 44% of institutions in the Somali regions (Annex Table 11).

Table 4.20: Immunization supply chain performance monitoring at lower distribution and service provision levels by health institution types

		Health Institution type				
		HC, n (%)	HP, n (%)	Hosp., n %)	WorHO, n (%)	Total, n (%)
Availability of "supply chain performance dashboard"	No	34 (70)	24 (63)	8 (100)	30 (77)	(72)
	Yes	15 (31)	14 (37)	0 (0)	9 (23)	38 (28)
Facility reviewed iSC performance within the last year	No	44 (44)	36 (43)	7 (58)	48 (53)	135 (48)
	Yes	55 (56)	47 (57)	5 (42)	42 (47)	149 (52)
HC = Health Centres, HP = Health Posts, Hosp = Hospitals, WorHO = Woreda Health Office,						

4.3 Supply chain essentials at central and sub central level

Utilities and services (electricity, water, and generator) infrastructure at central and sub central level

All hubs had sufficient water supply systems, with only four sub-central hubs lacking a hand washing facility. All supply chain equipment in the hubs were connected to electric power, except one hub that had electricity for only 8 hours a day.None of the hubs utilized onsite solar power for their cold chain equipment. All 16 hubs are equipped with electricity generators, although one non-functional (Table 4.21).

Table 4.21: Utilities and services in EPSS Central and sub-Central hubs

Variable		EPSS hubs		Total
		Central	Sub Central	
Facilities supply chain equipment connected to electricity.	Yes	1	15	16
Hours of grid electricity the facility received	24 hours	1	14	15
	8 hours	0	1	1
Incident of power cuts of more than 4 hours in the past 12 months	Yes	-	6	6
Facility's cold chain equipment powered by on-site solar system	No	1	15	16
Availability of 24-hour surveillance	Yes	1	14	15
Functional status of the generator	Functional	1	14	15
	Not Functional	0	1	1
	Total	1	15	16
EPSS=Ethiopian Pharmaceutical Supply Service				

Building amenities and conditions of central and sub-central hubs

The Central hub seem to be better off in terms of building amenities and conditions, though leaks in the ceiling and no emergency exit were observed. Yet, some of the sub-Central hubs had several problems: leaks in the ceiling, cracks on the ceiling or walls, potholes on the floor and broken windowpanes, absence of functional smoke alarms, etc. Their strength is that floors were clean and dry for all of them, and windows had locks/grill (Table 4.22).

Table 4.22: Building amenities and conditions at the central and sub-central hubs

Building condition		CH (n=1)	SCH(n=15)	Total
Leaks in the ceilings	Yes	1	5	6
	No	0	10	10
Cracks on the ceilings or walls	Yes	0	4	4
	No	1	11	12
Potholes on the floors	Yes	0	2	2
	No	1	13	14
Clean and dry floors	No	1	0	1
	Yes	0	15	15
Any broken windowpanes	No	1	11	12
	Yes	0	2	2
	NA	0	2	2
Windows have locks or grills	Yes	1	12	13
	NA	0	3	3
Building have functional manual fire alarm	Yes	1	12	13
	No	0	3	3
Building have a functional smoke alarm	No	0	7	7
	Yes	1	8	9
Building have a fire extinguisher	Yes	1	15	16
Building have an emergency exit	No	1	7	8
	Yes	0	8	8
NA = Not Applicable, CH = Central Hubs, SCH = Sub-Central Hubs				

Storage equipment (cold and other storage equipment) at the central and sub-central hubs

One of the critical components of effective vaccine management is ensuring all vaccines and diluents are stored in the proper temperature zone. Thus, the EPSS is one actor in the immunization supply. The installation of cold rooms and training programs, supported by UNICEF, GAVI, and the Ministry of Health, aids in optimizing cold chain capacity and deploying refrigerators to health institutions, including health posts. Focal persons have been employed at the regional hubs to oversee cold chain control. However, EPSS perceives this as a challenge due to the perception of it being a single-person activity.

"We installed the cold room there and provided training to EPSS technicians on controlling the cold chain system. EPSS also has 19 regional Hubs, distributed geographical, so they possess a large cold room. However, there is now a designated vaccine focal managing maintenance, expiration times, and communication with both of us (MOH) and EPSS." KII, Central MOH

All EPSS hubs store vaccines in long-term passive storage devices and have at least one refrigerator or freezer. There were 58 cold rooms across all hubs, with three lacking automatic deforestation and 25 missing door-open alarms. Distribution of cold rooms among hubs was as follows: 12 in central hub, 3 sub-central hubs have 5 each, 4 hubs have 2 each, 3 sub-central hubs have 3 each, and 2 sub-central hubs have 1 each. All hubs were equipped with refrigerators or freezers, only 4 having combined units. None of the refrigerators in the hubs had computerized temperature monitoring. However, there were no reported downtime incidents affecting the vaccine supply in the last 12 months. One sub-central hubs has 8 refrigerators in the store, compared to the central hubs with only 6. Nonetheless, the absence of computerized temperature monitoring poses a potential risk of vaccine wastage or damage (Table 4.23 , Table 4.24).

Table 4.23: Cold/freezer rooms at Central and sub-Central hubs in Ethiopia, 2023.

Variables		Regional		Total hubs
		Central	Sub-Central	
Facility have any cold rooms and/or freezer rooms	Yes	1	15	16
Well ventilated rooms containing the condensers	Yes	1	15	16
Total number of cold rooms in the hub	12	1	0	1
	5	0	3	3
	4	0	2	2
	3	0	5	5
	2	0	3	3
	1	0	2	2
Cold rooms with automatic deforestation	12	1	0	1
	5	0	2	2
	4	0	3	3
	3	0	5	5
	2	0	2	2
	1	0	2	2
	No deforestation	0	1	1
Number of cold/freezer rooms with a door-open alarm	6	0	1	1
	5	0	1	1
	4	0	2	2
	3	0	2	2
	2	0	2	2
	1	1	2	3
	0	0	5	5

The qualitative findings reveal that EPSS hubs in Ethiopia have a strong cold chain system for storing vaccines. However, some cold chain equipment, such as automatic defrosting and door-open alarms, were missing or not working in certain hubs. A key informant highlighted the potential of COVID-19 vaccination to expand storage capacity with freezers, especially at Central and regional hubs. Yet, this initiative lacks

support for maintenance, space, and skilled personnel, underscoring the need for effective warehouse management involving personnel.

> *"Though there has been improvement in terms of the storage capacity, we are good in cold rooms and freezer rooms, especially in EPSS hubs. Due to the introduction of some vaccines that need ultra-freezers, such as Pfizer, we have good freezer room capacity."* KII, Central Partner

Though there have been improvements compared to the past, there are still challenges in terms of human resources, even at central and regional hubs, where one person-activity is dominant including warehouse and cold-chain management.

> *"… in the warehouse, there is only one vaccine focal. If he is not available, someone else should own and work in that position. That person is the representative because there are many special things in the area. Sometimes we face such problems."* KII, Central EPSS

Partners such as CHAI have trained technicians and conducted maintenance campaigns periodically, employing mid-level and higher-level technicians. This aims to address challenges in maintaining the cold chain and providing solar refrigerators, which require less frequent maintenance and are unaffected by power interruptions

> *"Currently, we have some problems in some areas, one of which is the refrigerator, which works with electricity. It needs a lot of Spare parts, and they have become out of service because of electric fluctuations,...we got a solar fridge, which is better than the previous one, and it brought evolution to the Ethiopian cold chain system it works with solar, is good for rural areas because it has limited maintenance, and has good energy supplies, so this stabilizes so many things."* KII, Central level Partner

.

Table 4.24: Cold and other vaccine storage equipment in EPSS Central and Sub Central hubs in Ethiopia 2023.

Variables		EPSS hubs CH, n=1	SCH, n=15	Total Hubs
Facility have any refrigerators and/or freezers	Yes	1	15	16
Total number of refrigerators in the store	8	0	1	1
	6	1	0	1
	4	0	1	1
	3	0	4	4
	2	0	3	3
	1	0	2	2
	0	0	4	4
Total number of combined refrigerator freezers in the store	0	1	11	12
	1	0	2	2
	2	0	1	1
	3	0	1	1
Number of refrigerators with a functional 30-Da	0	1	11	12
Temperature Record (30DTR)	1	0	1	1
	2	0	2	2
	3	0	1	1
Number of refrigerators with good functional status regarding temperature control	14	0	1	1
	13	0	3	3
	12	0	2	2
	9	1	3	4
	8	0	3	3
	6	0	1	1
	5	0	1	1
	3	0	1	1
Any downtime incidents that affected the supply during the past 12 months	No	1	15	16
Refrigerators/freezers with a computerized temperature Monitoring system	No	1	15	16
EPSS=Ethiopian Pharmaceutical Supply Service, CH = Central Hubs, SCH = Sub-Central Hubs				

Transportation equipment (vehicles & insulated containers) at central and sub-central hubs

Thirteen sub-central hubs and the central hub use insulated containers for vaccine distribution. Vaccine transportation containers were packed within their building in the central hub and in fourteen sub-central hubs.

4.4 Supply chain essentials at lower distribution and service provision levels

Information Technology: General IT equipment (Telephone, Computers, Printers) at lower distribution and service provision levels.

Information technology plays a crucial role in supply chain management, influencing its effectiveness, quality, safety, and performance. However, the presence of computers in health institution offices appears to be limited, with only around 40% having computers. Furthermore, not all computers are utilized to support the supply chain, with only about 71% involved in supply chain operations. Of those with computers, only 61% are supported by IT experts (Table 4.25).

This scarcity or underutilization of technology poses challenges for data visibility and stock management. It hampers communication for requests between health facilities, regional hubs, and the Central EPSS. This situation is particularly evident at lower levels, where manual request forms are still prevalent despite the goal of targeted last-mile delivery and improved data visibility, including temperature monitoring across all levels.

> *"For example, if a branch requests an amount of stock from a health center under its supervision, it is difficult to get information except for a monthly manual report. So, priority should be given to improving automated data visibility. If you do not have data, you cannot proceed to the other system."* KII, Federal level

Table 4.25: Information technology at lower distribution and service provision levels by health institution types in Ethiopia 2023

Variables		Health Institution type				
		HC, n(%)	HP, n(%)	Hosp., n(%)	WorHO, n(%)	Total
Functional computer in the facility	Yes	57(56)	0(0)	8(67)	48(54)	113(40)
Facilities computer used to support supply chain operations	Yes	37 (65)	NA	5(63)	39(81)	81(72)
Facility have IT support	Yes	31(54)	NA	8(100)	30(62)	69(61)
HC=Health Centres, HP=Health Posts, Hosp=Hospitals, WorHO=Woreda Health Office, NA: Not Applicable						

Human resources: Staffing (availability and quality) at lower distribution and service provision levels.

The majority of institutions (44.76%) had only one supply chain staff, with about 83% having a maximum of three immunization supply chain staff, while very few have four, and only two have no such staff. (Table 4.26).

Table 4.26: Human Resource/Staff at lower distribution and service delivery level by facility type in Ethiopia, 2023.

		Health Institution type				
		HC, n(%)	HP, n(%)	Hosp., n(%)	WorHO, n(%)	Total
Total number of immunization Supply Chain (iSC) staff in the facility	Zero	0 (0)	15(1)	0 (0)	1(1)	2(1)
	One	53(53)	29(35)	6(50)	40(44)	128(45)
	Two	34(34)	34(41)	5(42)	37(41)	110(44)
	Three	8(8)	16(19)	1(8)	11(12)	36(13)
	Four	6(6)	3(4)	0 (0)	1(1)	10(3)

HC=Health Centres, HP=Health Posts, Hosp=Hospitals, WorHO=Woreda Health Office

Knowledge & understanding for staff at lower distribution and service delivery level

Most health institutions have staff that are knowledgeable about preventing freezing during transportation in sub-zero temperatures (94%) and packing insulated containers (94%), with the majority (92%) also equipped with staff knowledgeable about when and how to pack freeze indicators (Table 4.27).

Table 4.27: Staff knowledge on temperature management of vaccines at the lower distribution and service provision levels by health institutions type in Ethiopia 2023.

Variables	Health Institution type				
	HC, n(%)	HP, n(%)	Hosp. n(%)	WorHO, n(%)	Total
Staff know how to prevent freezing during transportation in sub-zero temperature	23 (96)	19 (100)	2 (50)	16 (94)	60 (94)
Staff know how to pack insulated containers for transportation	26 (100)	21 (95)	4 (80)	16 (89)	67 (94)
Staff know when to pack freeze indicators	13 (100)	10 (91)	1 (50)	11 (92)	35 (92)
Staff know how to pack freeze indicators	13 (100)	10 (91)	2 (100)	11 (92)	36 (95)
Responsible staff know what to do with vaccines in the event of vehicle breakdown	19 (100)	15 (88)	2 (67)	12 (92)	48 (92)

HC=Health Centres, HP=Health Posts, Hosp=Hospitals, WorHO=Woreda Health Office

Staff knowledge on stock management is generally good. Apart from how to use the stock management system (56%) and how to calculate unopened vial wastage rate (58%), the majority (ranging from 83.16% to 98.25%) are highly knowledgeable about various aspects including VVM use, diluent interchangeability, vaccine discard time, and unopened vial wastage (Table 4.28).

Table 4.28: Staff knowledge on stocks management of LD and SP levels by type of health institution in Ethiopia 2023.

	Health Institution type				
	HC, n(%)	HP, n(%)	Hosp. n(%)	WorHO, n(%)	Total
Responsible staff know how to use the manual/computerized stock management system	60 (60)	45 (54)	5 (42)	49 (54)	159 (56)
Staff know how to use the Vaccine Vial Monitor (VVM) in relation to the expiry	97 (97)	77 (93)	10 (83)	83 (92)	267 (94)
Responsible staff know that diluents are not inter-changeable	93 (93)	82 (99)	11 (92)	86 (96)	272 (95)
Responsible staff know the discard time of MDVP tracer vaccine	99 (99)	81 (98)	12 (100)	88 (98)	280 (98)
Responsible staff know the main types of unopened vial wastage	80 (80)	72 (87)	11 (92%)	74 (82)	237 (83)
Responsible staff know how to calculate the unopened vial wastage rate	61 (61)	52 (63)	6 (50)	48 (53)	167 (59)
Responsible staff know the main types of open vial wastage	82 (82)	70 (84)	11 (92)	74 (82)	237 (83)
HC=Health Centres, HP=Health Posts, Hosp=Hospitals, WorHO=Woreda Health Office					

Standard operating procedures (SOPs) for routine operations at lower distribution and service delivery level

SOPs are vital for routine operations in immunization supply chain management. Among the 191 health institutions assessed, 119 (62%) lacked safe injection SOPs in the immunization area; 133 of 285 (47%) had SOPs or guidance materials for immunization supply chain operations, with 99 (74%) written in a language understandable by the staff. About 160 of 285 (56%) lacked immunization supply chain (iSC) guidance materials for facility managers (Table 4.29).

Table 4.29: Availability of Standard Operating Procedures (SOPs) for routine operations at the LD and SP levels in Ethiopia 2023.

	Health Institution type				
	HC, n(%)	HP, n(%)	Hosp., n(%)	WorHO, n(%)	Total
Availability of SOPs for safe injection in the immunization room/area	27 (41)	15 (29)	2 (27)	28 (42)	72 (38)
Availability of SOPs/guidance material for iSC Operation	48 (47)	38 (46)	5 (42)	42 (47)	133 (47)
SOPs /guidance materials written in a language that can be understood by facility's staff	34 (71)	31 (82)	5 (100)	29 (69)	99 (74)
Availability of immunization Supply Chain (iSC) guidance materials for facility managers	42 (4)	36 (43)	5 (42)	42 (47)	125 (44)
HC=Health Centres, HP=Health Posts, Hosp=Hospitals, WorHO=Woreda Health Office					

4.5 Effective vaccine management (EVM) summary report

The EVM output result presents various scores (Es, Ms and Cs) for each of the four levels of operation: Central hub (CH), sub-central (SC), Lowest Distribution (LD) and Service Provision (SP) levels.

According to EVM standard an area of vaccine management is considered “Effective” if its criterion score is greater than or equal to 80%. Table 4.30 shows the overall Ethiopian EVM assessment score in 2023. Accordingly, the overall EVM score was 69%, far less than the desired 80% score. Among the criteria scores (E’s), vaccine management received the greatest score of 80%, while storage and transportation capacity received the lowest score of 64%. Policies and procedures (61%), and financial resources (80%) had the lowest and maximum category scores (C’s). The iSC performance monitoring parameter had the lowest overall score (57%), this may be due to the majority of assessed health institutions (71.64%) lacked a supply chain performance dashboard.

Table 4.30: Effective vaccine management Ethiopia 2023 facility operations and management score

		Infrastructure	Equipment	Information technology	Human resources	Policies & procedures	Financial resources			TOTAL
		C1	C2	C3	C4	C5	C6	OU TPU T	PERFORM ANCE	
Vaccine arrivals	E1			50	100	37	100	67		67
Temperature management	E2			54	80	56		51	85	66
Storage and transportation capacity	E3	19	38		94	67	83	73	89	64
Facility infrastructure and equipment	E4	66	67	50			62	76		67
Maintenance and repair	E5			54	80	61	69	59	89	69
Stock management	E6			84	75	69		64	32	70
Distribution of vaccines and dry goods	E7		83	26	66	60	70	69	91	69
Vacci ne management	E8				88	63		68		80
Waste management	E9		58		77	47	75	58	72	75
Annual needs forecasting	M1				83	69		97	37	78
Annual work planning	M2				82	65	83	71	81	76
Supportive supervision	M3	95	96	88	77	81	18	66		72
iSC performance monitoring	M4			83	72	59		49		57
TOTAL		66	64	67	78	61	80	66	75	69

Previously, EVM assessment was done in 2013 and 2019 and the results are presented here with the current, EVM 2023 study to help compare them in order to judge if progresses are made (Table 4.31 compared the EVM assessment output among 2013, 2019, and 2023. The timely arrival of vaccines is a critical factor in ensuring uninterrupted immunization programs. Vaccine arrival at the Central hub, however, performed unsatisfactorily in 2023 (score 67%) and a declining situation compared to 2019 (score 74%).

The EVM assessment output on temperature management of the three years shows a consistent value less than the target scores. Except for the sub-central level, temperature management greatly deteriorated over the years. However, there is an improvement between 2013 and 2023 for central and sub-Central hubs, from a score of 40% to 73% for the former and 54% to 76% for the latter. The indicator showed a decrease in the score for WorHOs and service provision level from 2013 to 2023. Temperature management is important during storage and transportation to prevent vaccine wastage. This shows that the cold chain had problem of storing and transporting vaccine while maintaining the required temperature level. So, the findings from the assessment tools call for immediate intervention to enhancing proper temperature management, including enhancements to functional information technology systems and monitoring proper temperature management in storage and during transportation.

The central hub was involved in vaccine arrival activities and the comparison showed an increment in score from the 2013 to 2023 but the score is lower compared to 2019. Overall, the scores in all the three years are lower than the WHO EVM standard score (80%).

The central hub demonstrated commendable improvement in stock management, vaccine management, maintenance and repair, and distribution of vaccines and dry goods between 2013 and 2023, scoring 90% and above 90% in all.

Some indicators, surprisingly, are getting worse in 2023 compared to 2013 assessment; this includes storage and transportation capacity at all levels and facility infrastructure and equipment in lower distribution and service provision facilities. This could be attributed to a lack of maintenance for storage equipment, which also resulted in a reduction in score for lower distribution and service provision facilities. Lack of timely maintenance or replacement of storage equipment can lead to damage, which hampers the storage capacities of facilities. As a result, the reduction in score for lower distribution and service provision facilities can be attributed to a decrease in functional storage equipment.

In almost all indicators the score for lower distribution and service provision facilities is much lower than the minimum the WHO-EVM standard score and mostly gets worse than previous years. Thus, there is a need to focus on lower distribution and service provision facilities to improve the overall supply chain management of the country.

Table 4.31: Comparison of Supply chain management findings from 2013, 2019 and 2023 EVM assessments in Ethiopia.

	Central hub			Sub-Central hubs			Woreda Health offices (LD)			Service Provision (health facilities)		
EVM Assessment Years	2013	2019	2023	2013	2019	2023	2013	2019	2023	2013	2019	2023
Vaccine arrival (E1)	57	74	67	-	-	-	-	-	-	-	-	-
Temperature management (E2)	40	75	73	54	63	76	71	71	65	69	70	54
Storage and Transportation Capacity (E3)	89	71	62	80	90	72	76	76	60	82	76	63
Facility infrastructure and equipment (E4)	91	96	89	80	82	82	69	71	50	76	74	53
Maintenance and repair (E5)	65	64	90	68	72	84	71	51	59	67	56	50
Stock management (E6)	64	77	97	54	80	89	51	57	62	53	50	45
DistriHoweverion of vaccines and dry goods (E7)	60	81	90	43	77	75	44	65	60	84	59	56
Vaccine management (E8)	50	80	100	68	84	88	67	77	71	80	82	65
Waste management (E9)	59	92	96	80	59	84	75	48	65	NA	45	60

4.6 Conclusions

Effectiveness of the Ethiopian immunization supply chain management was far less than the desired EVM standard 80% score, indicating a need for improvement in supply chain efficiency, logistics and coordination. Efforts in vaccine procurement based on forecasting and micro-planning face challenges such as lack of denominators, migration/internal displacement, and cross-boundary movement from neighboring countries, impacting forecast effectiveness, stock management, and vaccine wastage. Implementation of computerized stock management systems at central and sub-central hubs has improved organization, yet issues persist, including late requests and shortages of cold van transportation which could lead to delays in vaccine delivery, potentially resulting in vaccine stock-outs and disruptions in immunization services.

Documentation at lower distribution and service provision levels is encouraging but lacks a computerized management system, hindering effective vaccine stock management. While stock management knowledge among staff is generally good, challenges persist among skilled personnel, particularly in effective warehouse management and calculating unopened vial waste rates. Additionally, an inadequate number of supply chain staff was observed, with supply chain activities often performed by other EPI staff. This implies potential inefficiencies in inventory control and distribution, leading to inaccuracies in stock records.

While central and sub-central hubs exhibit adequate storage capacities, many health institutions lack sufficient infrastructure, leading to vaccine stock-outs and wastage. The net storage capacity of refrigerator compartments on the central hub was 440.8 m3, and the sub-national hubs had 798.2 m3. The average institutional storage capacity at LD and SP was 1433.9 liters for vaccines positive and 538 liters for vaccines negative. Inconsistent funding exacerbates these challenges, highlighting the need for sustainable investment in storage facilities.

The country faces a significant shortage of refrigerators with proper temperature control and computerized monitoring systems, posing a threat to vaccine potency. Interruptions in electricity and lack of transportation further compromise temperature management, necessitating improvements in infrastructure and equipment. Scheduled maintenance of supply chain equipment is lacking, compounded by shortages of funds, spare parts, and skilled technicians.

The transition to last-mile delivery has improved distribution efficiency, but poor transportation infrastructure and inadequate storage equipment hinder effective distribution, especially at lower levels. Vehicle unavailability hampers effective distribution, especially at lower levels. This may affect ensuring timely and efficient vaccine distribution, particularly at lower levels, resulting in potential delays in immunization services and compromised vaccine coverage.

Lack of a supply chain performance dashboard and delays in vaccine reporting hinder data visibility and utilization and may affect decision-making and optimizing vaccine distribution. Inadequate waste management practices, including improper segregation and disposal methods, pose risks of hazardous spills and needle stick injuries posing safety challenges to the healthcare providers and communities.

4.7 Recommendations

The following actions are suggested to enhance the effectiveness of the immunization supply chain management system:

- Implement measures to improve vaccine forecasting at the service provision level based on updated head counts and eligible population at the micro level through the health extension workers and considering migration and cross-boundary movement.
- Cascading implementation of a computerized stock management systems at all levels of the supply chain and use of standardized leisure books at the lower distribution and service provision levels to improve organization, reduce stock-outs, and minimize vaccine wastage.
- Strengthening the availability of cold chain equipment and ensuring its functionality through scheduled and regular maintenance and repair
- Allocate sustainable funding for the construction and maintenance of storage facilities with proper temperature control and monitoring systems to safeguard vaccine potency.
- To ensure effectiveness of last-mile delivery, address challenges related to vehicle availability and transportation infrastructure to ensure timely and efficient vaccine delivery, especially at lower levels.
- Develop a supply chain performance dashboard to improve data visibility and reporting, enabling better decision-making and optimization of vaccine distribution.
- Implement proper waste management protocols to reduce risks of hazardous spills and injuries, ensuring the safety of healthcare providers and communities.
- The system should consider independent unit through hiring dedicated immunization supply chain staff for efficient and effective vaccine supply chain management.

IMMUNIZATION SERVICE DELIVERY

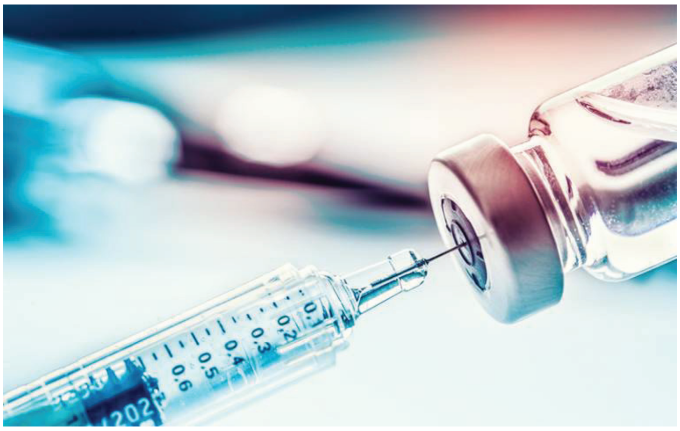

5. IMMUNIZATION SERVICE DELIVERY

This section includes the availability and readiness of immunization services, the strategies used for service delivery, and the integration of immunization services with other health programs. The study further assessed immunization coverage among various population groups, including children aged 12-35 months, adolescent girls aged 15-18 years, women of childbearing age, and adults over 12 years old. The assessment of service availability and readiness involved evaluating the presence of essential items and the overall availability of services in health facilities. Similarly, the study examined the different strategies employed by health facilities for immunization delivery such as static, mobile, outreach, campaign, PIRI and other strategies. Furthermore, the integration of immunization services with other health programs was also assessed to understand the extent to which child and adult immunization services are combined with other health services. The quality of immunization sessions was evaluated through observations of vaccinators' practices before, during, and after administering vaccines, based on specific monitoring indicators.

Key findings

Service availability and readiness

- Merely four in ten (38%) of the health facilities provided immunization services everyday.
- One in ten (12%) of the health facilities had all the required tracer items.
- Nearly seven in ten (73%) health facilities had the average tracer items (15 out of 21 tracer items)

Service delivery strategies

- Majority of the health facilities used static as the main immunization service delivery strategy.
- Pastoral regions used mobile approach as one of their key strategies.
- Campaign was used as the main strategy to reach zero dose and under immunized children.

Session quality

- Only two thirds of the vaccinators practiced proper BCG and measles preparation.
- Only 7% of the vaccinators provide all immunization key messages to mothers/caregivers.
- Proper infant assessment was also properly practiced at only 4% of the observations.

Child immunization coverage

- The coverage of third dose of Pentavalent was 64.7% and MCV1 was 60.3%.
- Only 43.9% of children received all vaccines.
- One in four children didn't take any dose of vaccines.
- The dropout rates of Pentavalent were 14.1% and MCV1 was 19.9%.
- The coverage of MCV2 was 54.7%.

Td, HPV and COVID -19

- 85% of mothers received at least one dose of Td vaccine
- 54% the adolescent girls received at least one dose of HPV vaccine.
- The coverage of COVID-19 vaccine for at least one dose was 55.9%.

5.1 Immunization Service Availability

Of the health facilities, 95% offered immunization services. Merely four in ten (38%) of the health facilities offered immunization services daily. Eight in ten health facilities in Addis Ababa and Harari and only one in five health facilities in Sidama and Southwest Ethiopia Peoples regions offered daily bases immunization services. Of health facilities offering immunization services, only 60% offered birth, 62% infant, and 59% adult vaccines. HPV and COVID-19 vaccines were delivered by 44% of the health facilities Figure 5.1.

Lack of refrigerators, and security/conflict were the common reasons for not offering immunization services. The reasons of not offering immunization services daily were, lack of refrigerators, workload, security, vaccine wastage for multi-dose vials like BCG and measles, low cluster demand and eligible population, and distance/geographic accessibility were reported.

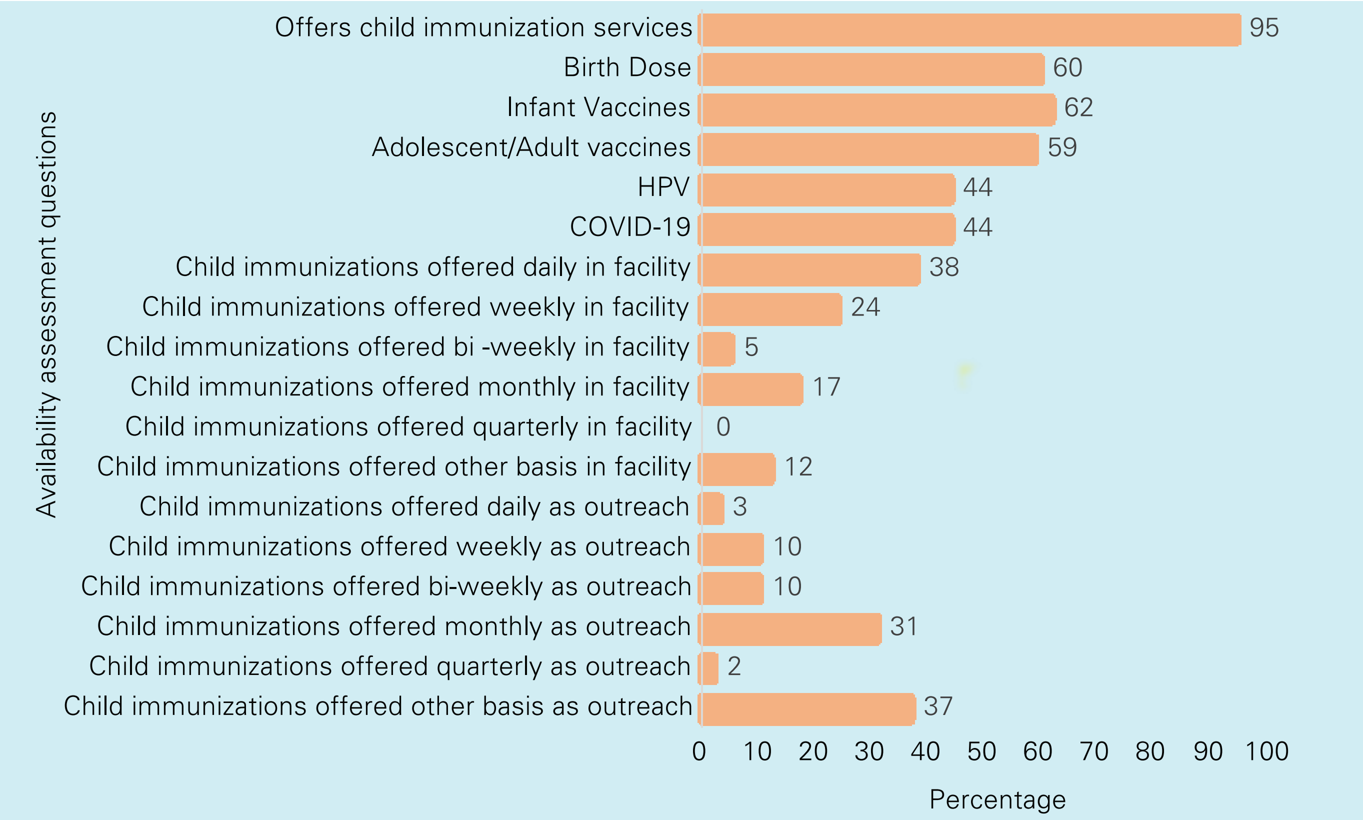

Figure 5.1: Percentage of facilities that offer immunization services in Ethiopia 2023.

Though most health facilities offered immunization services, there is variation in the availability of antigens and frequency of service provision across regions, facility types, and settings. 80% of Addis Ababa and Harari region health facilities provided immunization services on daily bases. However, 50 % of health facilities from the rest of the regions offered immunization service daily. 54 % of the urban health facilities offered immunization service daily followed by rural 27 %. Only 13 % of the health posts offered daily immunization service. 70% urban health facilities had birth, infant, and adult doses whereas only 50% of rural health facilities had these vaccines. The availability of these doses at the health posts was below 50%. In regions such as Sidama, SNNP, Gambela, and southwest Ethiopia Peoples lower than 50% of health facilities had these doses; however, health facilities in Addis Ababa (above 90%), Harari, and and Dire Dawa (above 80%) and Somali (above 75%) had these vaccine doses Table 5.1.

Table 5.1: Immunization service availability (percentage) by region, facility type and setting, Ethiopia 2023

	Offers child immunization services	Birth Dose	Infant Vaccines	Td Vaccines	HPV	COVID-19	Child immunizations offered daily in the facility	Child immunizations offered weekly in the facility	Child immunizations offered bimonthly in the facility	Child immunizations offered monthly in the facility	Child immunizations offered quarterly in the facility	Child immunizations offered onother[1] basis in the facility	Child immunizations offered daily as an outreach	Child immunizations offered weekly as outreach	Child immunizations offered bi-monthly as outreach	Child immunizations offered monthly as a outreach	Child immunizations offered quarterly as outreach	Child immunizations offered on other[2] basis as outreach	Total number of studied facilities
Region																			
Addis Ababa	100	96	96	96	59	41	85	15	0	0	0	0	0	0	0	0	89	0	17
Afar	95	63	63	60	45	57	33	41	2	13	0	5	3	8	0	69	15	5	16
Amhara	94	66	66	63	38	40	38	14	6	15	0	6	4	6	8	24	51	6	159
Benishangul Gumuz	96	64	50	45	34	40	33	5	3	4	0	4	3	0	0	41	54	4	15
Dire Dawa	100	82	82	82	73	82	53	12	0	35	0	0	0	25	0	22	53	0	2
Gambela	89	43	43	36	22	32	40	16	0	0	0	11	4	0	0	48	37	11	7
Harari	100	80	80	80	50	60	80	18	0	2	0	0	0	15	40	23	23	0	2
Oromia	96	60	62	59	48	47	42	25	6	10	0	4	4	12	12	29	38	4	270
SNNP	95	51	55	50	49	49	33	29	6	7	0	5	0	25	10	33	24	5	26
Sidama	100	51	53	53	49	43	19	45	5	13	0	4	0	27	17	32	18	0	98
Somali	96	77	75	77	46	49	54	20	5	12	0	4	2	22	7	45	19	4	45
Southwest Ethiopia Peoples	92	29	32	32	16	16	22	23	3	23	0	8	2	10	3	53	22	8	27

Facility type																			
Referral Hospital	100	98	98	98	40	27	90	0	2	0	0	7	0	0	0	2	0	98	14
General Hospital	92	79	78	71	23	55	64	11	0	0	0	17	5	0	0	5	0	82	19
Primary Hospital	94	55	55	55	23	36	53	14	0	4	0	24	3	15	1	0	1	74	43
Health Center	99	69	70	69	54	53	55	24	2	6	0	12	4	12	8	26	4	45	317
Health Post	94	48	51	48	39	35	13	29	10	32	0	10	2	16	14	45	1	16	291
Setting																			
Urban	93	72	71	70	46	50	54	19	2	4	0	15	4	11	6	15	2	56	161
Rural	98	53	56	53	43	40	27	28	7	25	0	10	2	15	13	42	2	24	523
Total	95	60	62	59	44	44	38	24	5	17	0	12	3	13	10	31	2	37	684

[1]Others for static session frequency notes bi-weekly, three times a week, and four times a week; Other[2] for outreach session frequency notes every six months, every two months, and during campaign times without a specific date; SNNP=Southern Nations, Nationalities and People's region; HPV= Human Papilloma Virus

5.2. Immunization Service Readiness

The readiness of the health facilities to deliver quality vaccination services was assessed by checking the availability of adequate human resources, vaccination equipment, and vaccination medicines and commodities. Its service was assessed using the availability of 21 tracer items.

Of the health facilities, only 12% of them had all tracer items. The mean availability of tracer items in health facilities was 73% (15 items from the 21 tracer items). Sixty-six percent of the facilities had at least one trained staff on immunization in the last two years before this survey. The availability of national immunization guidelines was observed at only 48% of the health facilities. Among the equipment assessed, cold boxes, Ad-syringes, and safety boxes were available at more than 90% of the health facilities. However, the stock balance was observed only at 57% of the health facilities implies low vaccine demand forecasting, requesting, and assessment practices. Of health facilities offered immunization services, the availability of vaccination antigens at the time of the assessment ranges from 61-71% Figure 5.2.

High staff turnover, high workload, lack of infrastructure, including road problems during the summer season, lack of waiting rooms at health facilities, security problems, shortage of EPI supplies, distance, poor record of full doses or drop out of immunization were among were the challenges reported by key informants. Besides, organizational structure or governance of the immunization program and shortage of human resources were also main barriers to the readiness of the facilities to deliver immunization services effectively. Immunization programs personnel had multiple responsibilities such as nutrition and maternal and child health services and affects the focus to the immunization service provision, monitoring, and evaluation. Community engagement during the planning and delivering immunization services were the coping strategies applied.

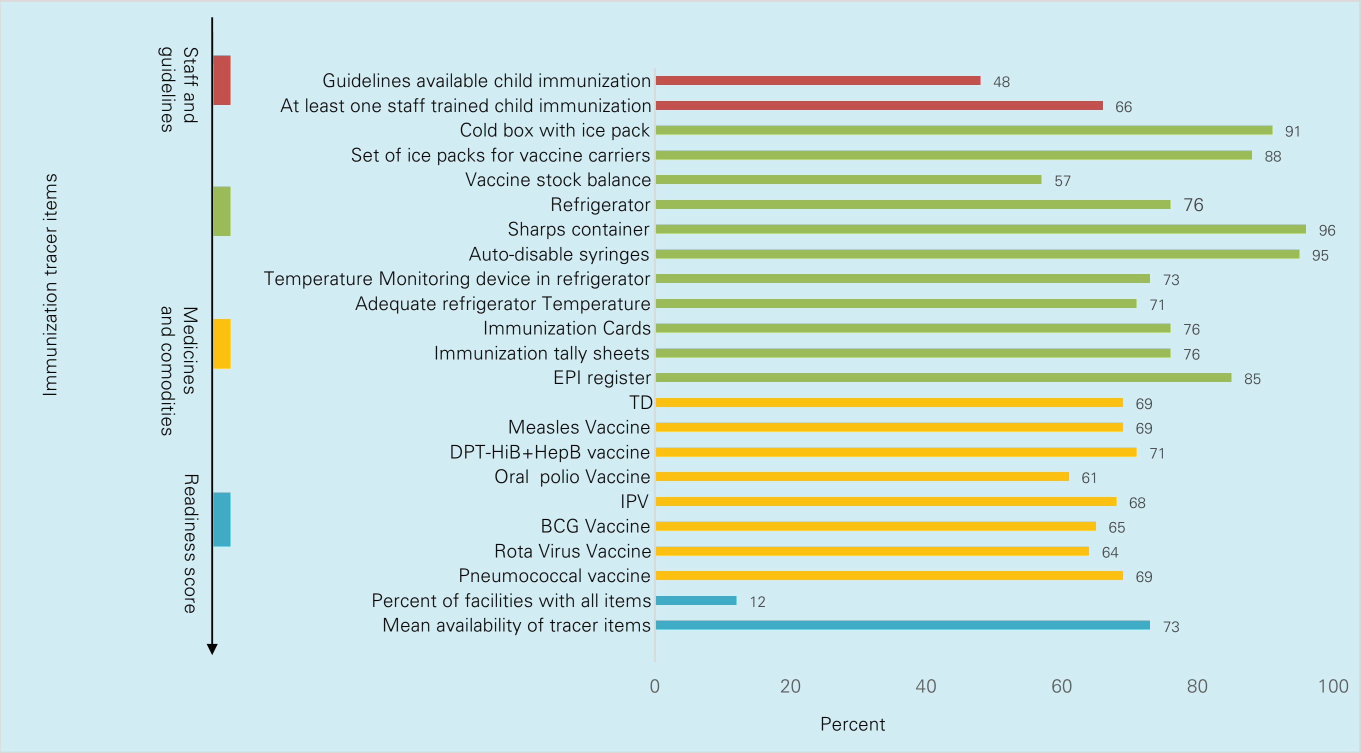

Figure 5.2: Availability of tracer items (percent) for immunization services among health facilities in Ethiopia 2023

Approximately 50% of the health facilities in Addis Ababa were discovered to comply with all the required tracer items. Surprisingly, none of the facilities located in the southwestern regions of Ethiopia met the criteria. On the other hand, the readiness score of facilities in Somali, Afar, Sidama, and Amhara wasquite concerning, with less than 10% of them meeting all the requirements for readiness tracer items. Additionally, only 7% of rural facilities were able to fulfil all the tracer items, while 19% of urban facilities did so. Although the majority of immunization services are provided at health posts, the availability of tracer items is alarmingly low, with only 1% of health posts meeting all the requirements compared to health centers (18%) and hospitals (30%). The average availability of tracer items at health posts is noticeably lower than in other health facilities Table 5.2.

Table 5.2: Percentage of facilities with tracer items for immunization services by region, facility type, and setting Ethiopia, 2023.

	Guidelines available for child immunization	At least one staff trained child immunization	Cold box with an ice pack	Set of ice packs for vaccine carriers	Vaccine stock balance	Refrigerator	Sharps container	Auto-disable syringes	Temperature Monitoring device in refrigerator	Adequate refrigerator Temperature	Immunization Cards	Immunization tally sheets	EPI register	Td	Measles Vaccine	Pentavalent vaccine	Oral polio Vaccine	Inactivated poliovirus vaccine	BCG Vaccine	Rota Virus Vaccine	Pneumococcal vaccine	Percent of facilities with all items	Mean availability of tracer items	Total number of facilities
Regions																								
Addis Ababa	93	89	100	100	100	100	100	100	100	93	100	100	100	100	96	100	89	100	100	74	100	48	97	16
Afar	41	69	94	90	90	87	94	97	76	76	90	80	90	81	77	83	79	87	87	83	83	8	83	15
Amhara	52	70	82	87	87	79	99	98	83	68	61	77	93	77	79	77	66	79	71	69	78	10	78	149
Benishangul Gumuz	33	47	95	95	95	77	95	95	72	78	58	81	100	70	68	65	62	61	49	59	70	14	73	15
Dire Dawa	42	89	100	100	100	100	97	100	97	87	57	100	92	95	95	97	84	100	90	90	100	19	91	3
Gambela	66	89	100	100	100	100	100	100	87	80	88	96	100	96	89	96	89	91	74	96	89	24	92	6
Harari	65	62	85	77	77	98	90	100	97	95	80	93	75	95	93	93	73	88	93	95	95	10	87	1
Oromia	54	77	93	93	93	71	98	96	69	73	84	82	85	63	62	65	55	59	59	58	61	13	74	256
SNNP	34	58	87	87	87	80	96	96	74	70	67	59	75	68	67	68	55	68	68	58	69	10	71	24
Sidama	36	59	86	79	79	73	91	90	73	70	81	64	75	64	65	69	63	69	64	60	64	9	70	98
Somali	31	59	83	83	83	86	86	80	49	65	74	62	79	78	82	81	70	72	76	83	80	6	73	43
Southwest Ethiopia Peoples	27	59	84	76	76	57	97	91	49	57	68	68	77	46	43	49	44	41	42	49	49	0	59	24

Facility type																								
Ref/hospital	93	93	100	100	100	100	100	90	90	90	93	100	100	100	100	100	93	100	100	60	100	52	95	19
Gen/ hospital	38	56	89	97	73	97	100	97	93	75	70	100	95	93	96	96	89	85	97	96	92	3	87	14
Prim/hospital	60	70	85	87	69	93	85	88	79	79	73	84	86	88	86	88	82	85	85	81	88	27	82	43
Health center	63	72	99	95	87	95	98	98	90	78	82	87	97	90	88	90	78	88	85	80	88	18	87	304
Health Post	27	57	82	80	17	48	95	92	28	51	70	60	70	39	40	43	36	39	35	41	40	1	52	270
Setting																								
Urban	60	73	91	89	79	88	96	95	89	80	81	88	93	84	83	84	75	81	81	77	83	19	83	249
Rural	40	61	90	87	43	68	95	94	60	64	73	69	80	60	60	62	53	60	55	55	60	7	66	401
Total	48	66	91	88	57	76	96	95	73	71	76	76	85	69	69	71	61	68	65	64	69	12	73	650

SNNP=Southern Nations, Nationalities and People's region, Td=Tetanus-Diphtheria Vaccine, BCG=Bacillus Calmette-Guérin, DPT - HiB+HepB=Diphtheria, Pertussis, Tetanus, Homophiles Influenza, and Hepatitis B vaccines

Immunization-related capacity-building training

Figure 5.3 describes the training provision performance-related immunization in the last two years before the assessment. It revealed that almost half of the staff working in the immunization unit didn't get the training related to immunization. The potential reasons stated were a shortage of resources, a staff rotation platform that shifts the trained health professionals from immunization to others, and leaving the trained staff to other programs because the immunization unit didn't have any incentive to retain the health professionals. It was also described as most trained health professionals diverting to other programs, transferring to other health facilities, or quitting their jobs before completing expected service 1 or 2 years after training. So, continuous training of health professionals is important, but they couldn't train due to budget scarcity.

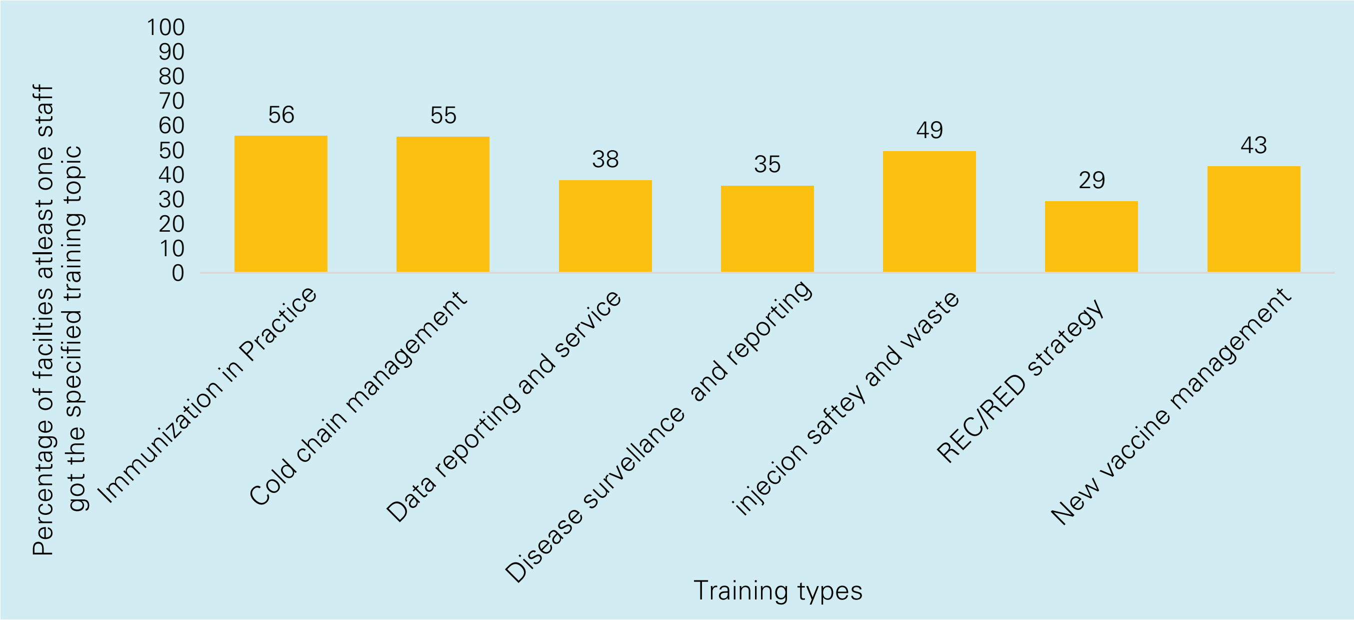

Figure 5.3: Proportion of health facilities with at least one staff got immunization related training in the last two years prior to the survey by training types in Ethiopia. 2023

5.3 Immunization Service Delivery Strategy and Integration Approaches

Immunization service delivery strategy by health facilities (health centers and hospitals)

The Ethiopian national guidelines for immunization emphasize the importance of tailored delivery strategies based on the country's diverse regional landscape. The guidelines highlight various approaches such as static, mobile, campaign, and outreach methods as key strategies for immunization service delivery. Furthermore, strategies like Catch-up, PIRI, mop-up, and hit-and-run are also utilized to reach missed and underserved populations.

The study found that 99% of health facilities surveyed utilize the static immunization service delivery strategy. Moreover, all regions with the exception of Addis Ababa have adopted an outreach strategy to enhance immunization coverage. Facilities in Somali, Afar, Gambela, and Oromia regions have also incorporated the mobile strategy as part of their service delivery framework.

Approaches to reach zero dose and under-immunized children by health facilities (health centers and hospitals)

Health facilities implemented various strategies to ensure that all children receive the necessary immunizations. These strategies included the Periodically Intensified Routine Immunization (PIRI), the Reach Every Child/Reach Every District (REC/RED) plan, targeted campaigns, local social mobilizations involving community leaders and women's groups, leveraging family connections, and conducting house-to-house surveys to identify children who were not up to date on their vaccinations. The REC/RED approach was particularly popular among health facilities in regions like southwest Ethiopia (59%), Somalia (62.1%), Sidama (55.8%), SNNP (55.2%), and Benishangul Gumuz (48.3%), as highlighted in Table 5.3.

Table 5.3: Approaches used by health facilities to reach zero-dose and under-immunized children in Ethiopia, 2023

Characteristics	REC/RED strategy	PIRI	Campaigns	Others*	Total facilities visited
Region					
Addis Ababa	10.3	0	27.5	75.9	17
Afar	40.7	40.6	53.2	0	10
Amhara	40.9	35.5	35.2	31.1	97
Ben/Gumuz	48.3	13.8	34.5	24.1	7
Dire Dawa	32.6	16.3	38	31.2	2
Gambela	53.9	21.6	89.2	0	4
Harari	0	0	46	54	2
Oromia	34.2	23.4	52.7	28	149
SNNP	55.2	23.2	49.1	13.5	16
Sidama	55.8	29.4	44.2	14.7	54
Somali	62.1	26.7	51.4	8.1	22
Southwest Ethiopia Peop	59	22.9	62.8	3.6	13
Facility type					
Referral Hospital	2.2	11.9	0	88.9	14
General Hospital	2.1	32.7	6.8	60.6	19
Primary Hospital	36.5	13.2	14.1	42.5	43
Health Center	46.2	28.5	54.6	18.9	317
Setting					
Urban	33.3	24.5	44.2	33.7	117
Agrarian	44.9	27.3	45.7	24.4	243
Pastoralist	46.9	28.4	55.9	6	33
Total	41.6	26.5	46	25.8	393

PIRI=Periodically Intensified Routine Immunization, REC/RED=Reach Every Child/Reach Every District,SNNP=Southern Nations, Nationalities and People's region,
Phone calling, house-to house survey, mobilization via women development army,mobilization using kebele leaders, & family health team link

Missed immunization sessions by the health facilities (health centers and hospitals)

The assessment also revealed that among the assessed health facilities, 24.2% of them reported that they had the experience of missing scheduled immunization sessions in the previous six months. Workload, different campaign activities, staff shortage, security problems, vaccine supply shortage, refrigerator failure, holy days, meetings, and other overlapping tasks were reported as the reasons for missing scheduled immunization sessions.

Immunization Service Integration by the health facilities (health centers and hospitals)

The immunization service integration approaches were assessed from the plan and experience of facilities in providing the immunization service by integration with other maternal child and other essential health services.

The assessment revealed that around 82% of health facilities reported offering immunization by integrating with other services. Health facilities from Afar (90.7%), Dire Dawa (100%), Harari (93.4%), and SNNP (93.7%) regions reported that they are integrating the immunization services with newborn and child health (IMNCI), Maternal (ANC, Delivery and PNC), Family Planning, Nutrition (Screening, Vit.A & Deworming), and other health services like TB/ART, chronic services during routine and campaign programs. The immunization service integration guideline was observed only at half of the health facilities during the Campaigns. The guideline was observed at above 80% of the Harari and Dire Dawa facilities Figure 5.4.

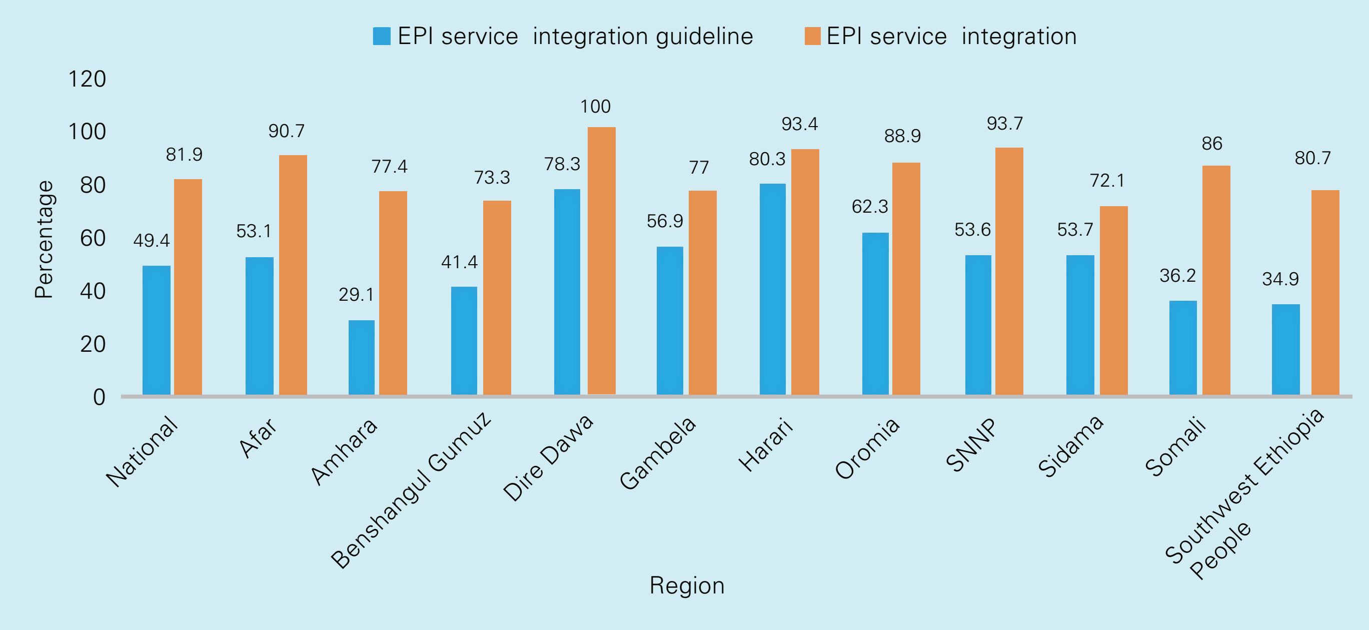

Figure 5.4: Immunization service integration and guideline availability by the health facilities across regions in Ethiopia, 2023.

Immunization delivery strategies used by health posts

The health posts also reported as using different strategies for immunization service delivery. Static, mobiles, and campaign activities were the reported strategies. Among the 291 Health posts in the survey, 79% use the static approach. Regarding regions, 88% of the Harari Health posts implemented the static strategy. Only 56% of the health posts in South West Ethiopia Peoples use the static approach. Besides, 85% of the health posts are agrarian, 81% are pastoralists, and only.

The mobile strategy is implemented in 10% of the health posts included in this survey. The mobile strategy was reported as one strategy by the health posts from Afar, Somali, Gambela and Oromia regions. The outreach strategy is implemented in 74% of the health posts included in the study. Eighty-eight percent of the Health posts from Harari and Dire Dawa regions implemented the outreach strategy. In contrast, only 46% of the health posts from the Benishangul Gumuz region implemented this strategy. The outreach strategy is implemented among 85% of the health posts from the agrarian, 59% from the Pastoralist, and only 33% from the Urban Health posts.

To implement the outreach strategy, health posts should use different settings. Common site villages are used among 65% of the health posts included in the study. Besides, common site villages are used among 88% of the health posts from the Dire Dawa region, 83% from Afar, and only 36% from the Gambela region. Religious institutions were also used as an outreach delivery setting for 26% of the health posts included in the survey Table 5.4.

Approaches to Reach Disadvantaged Children by health posts

The PIRI is implemented among 16% of the health posts included in the study to reach the disadvantaged children. Twenty-seven percent of the Health posts from Gambela, 2% from Benishangul Gumuz, only 5% from the Oromia region, and none of the health posts from the Somali region used the PIRI approach. The PIRI is also implemented among 29% of the health posts from the pastoralists, 15% from the agrarian, and only 9% from the urban settings Table 5.4.

The RED/REC is implemented in 31% of the health posts included in the survey. Half (50%) of the health posts from Dire Dawa, 46% from Gambela, only 25% from Afar, and none of the health posts from Harari are implementing the RED/REC approach. Thirty-five percent of the health posts were from pastoralist settings, 33% were from agrarian settings, and 16% were from urban settings that implemented the RED/REC approach as indicated in Table 5.4.

Table 5.4: Percentage of immunization services delivery strategies by setting and approaches implemented among studied health posts 2023, Ethiopia.

	Immunization services delivery strategies				Settings where outreaches are implemente				Approaches used to reach the disadvantaged					Number of Health posts included
	Static	Mobile	Outreach	All	Common site villages	Religious institutions	Remote villages	Schools	PIRI	REC/RED	Outreach	Child day	Others *	
Region														
Afar	83	17	83	0	83	0	33	25	8	25	67	17	0	6
Amhara	62	7	71	6	60	35	20	42	16	36	64	0	11	61
Ben/Gumuz	77	8	46	8	46	23	15	23	23	23	46	0	8	9
Gambela	82	9	55	0	36	27	9	18	27	46	64	36	0	3
Oromia	86	24	77	7	69	17	20	41	5	26	73	6	14	121
SNNP	81	14	73	8	73	27	30	27	10	30	68	5	5	9
Sidama	85	5	85	10	70	55	15	40	21	30	80	0	5	45
Somali	86	14	57	4	46	4	25	25	0	43	61	4	4	23
Southwest Ethiopia Peoples	56	11	78	0	67	22	28	17	16	39	83	0	0	14
Setting														
Urban	44	5	33	5	36	20	12	24	9	16	27	0	23	43
Agrarian	85	12	85	5	72	30	20	41	15	33	80	3	7	212
Pastoralist	81	15	59	17	55	12	35	31	29	35	64	8	8	37
Total	79	10	74	7	65	26	20	37	16	31	70	4	10	291

*HDAs, campaign, house-to-house survey, registering targets in the village, community mobilization.

PIRI=Periodically Intensified Routine Immunization, REC/RED=Reach Every Child/Reach Every District, SNNP=Southern Nations, Nationalities and People's region

Missed immunization sessions by health posts

The assessment also indicated that among the assessed health posts, 19.5% reported that they had experienced missing scheduled vaccination sessions in the last six months. A higher percentage of health posts from Afar (50%) and Harari (41.2%) reported a history of session interruption. The respondents stated workload, campaign activities (COVID-19, CBHI), staff shortage, security problems, vaccine supply shortage, holy days, training programs, and unscheduled meetings as the reasons for missing scheduled immunization sessions. The respondents also explained that most missed immunization sessions were not compensated Figure 5.5.

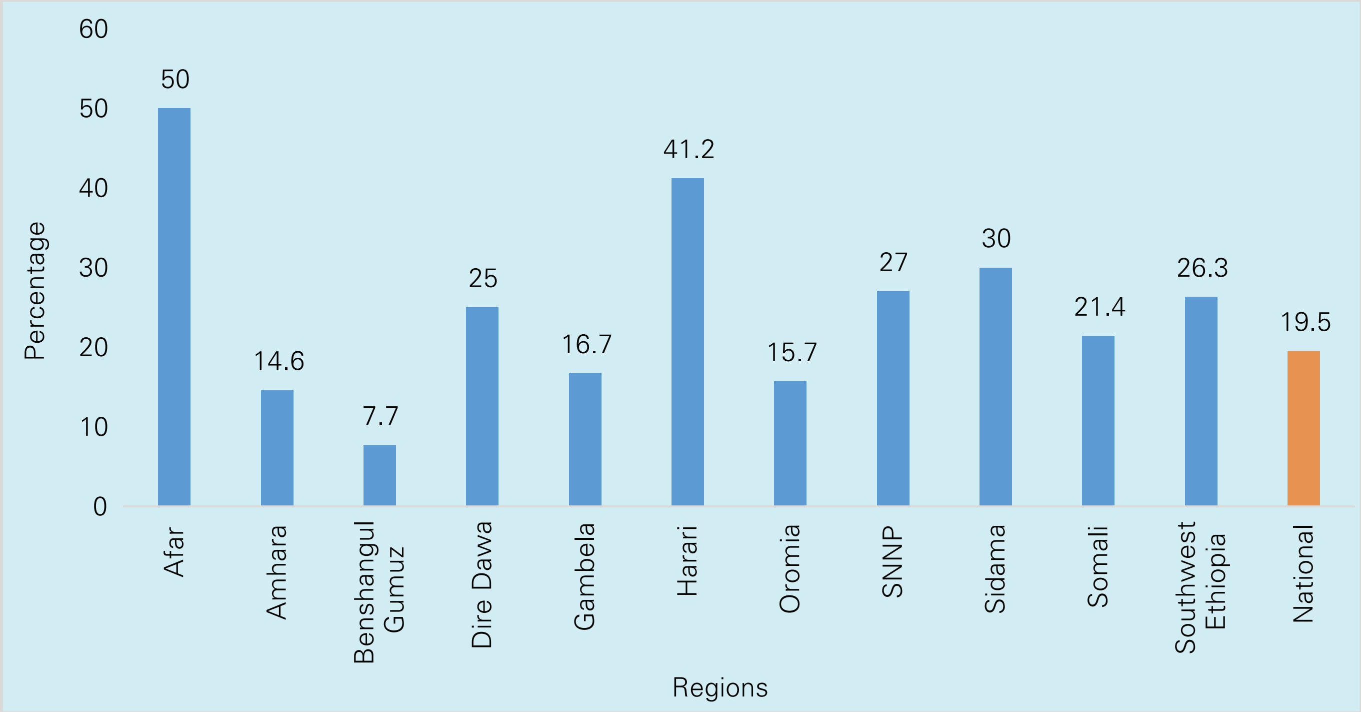

Figure 5.5: Percentage of health posts who had experience of missing scheduled vaccination sessions in Ethiopia, 2023.

Immunization integration at health posts

The assessment revealed that around 76% of health posts reported offering immunization through integrating with MCAHN and other health services. Health posts from Oromia (90%), Dire Dawa (87.5%), and regions reported integrating immunization services. The health posts also indicated integrating the immunization service with different childhood, maternal, and other health services. The immunization service is integrated with Vitamin A, deworming, growth monitoring, ICCM, and other campaign programs. The immunization service integration guideline was observed only at one in four health posts. Campaigns. The guideline was observed at above half the health posts from Afar, and 41.7% of health was from Gambela Figure 5.6.

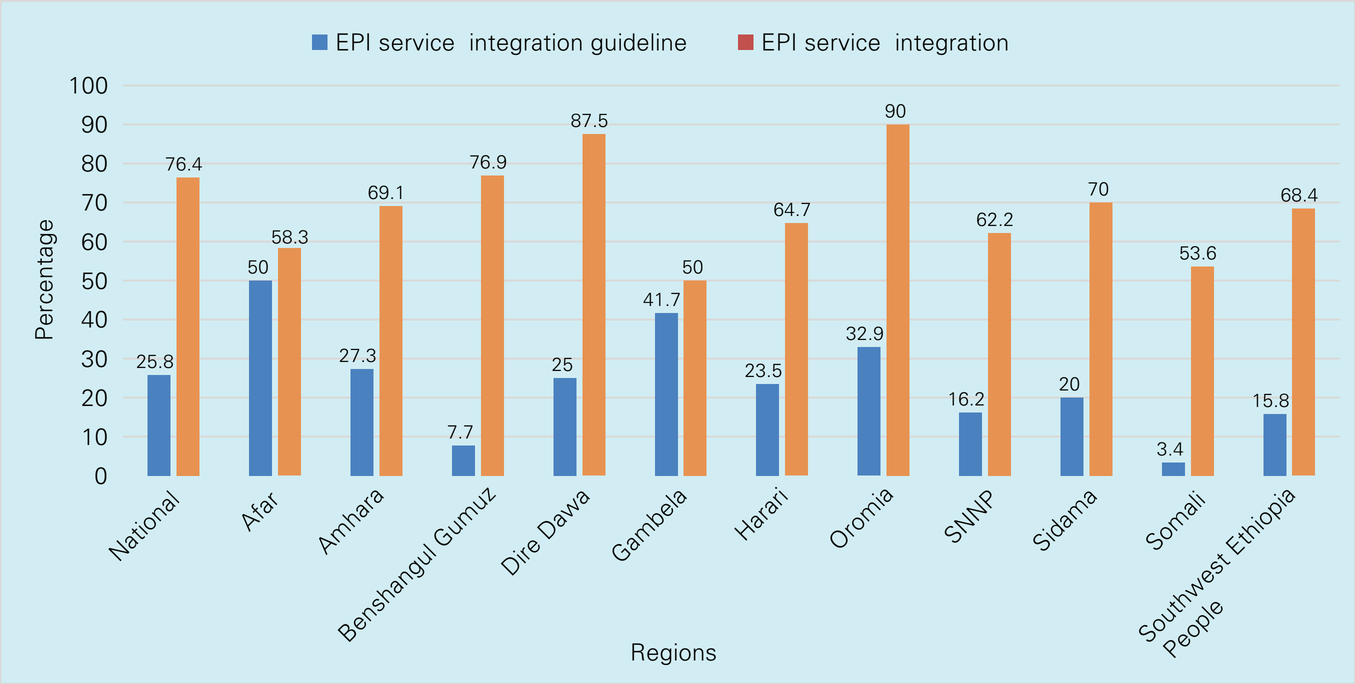

Figure 5.6: Immunization service integration and guideline availability by health posts across regions in Ethiopia, 2023.

5.4. Immunization Session Quality

Vaccination safety practice was assessed by observing the vaccinators' practice from their session preparation (pre-administration phase), injection safety practice (during administration), and post-administration practices. The session observation was done at 2954 sessions across 12 regions.

Vaccination safety practice pre-administration

The immunization pre-administration safety practice was assessed by observing the practices of vaccinators on the selected session preparation activities. The assessment was done over 2954 sessions using 21 selected activities.

Of the vaccinators observed, about 80 percent of them practiced properly: session cleanness, arranging chairs and tables, properly explaining mothers' questions, reviewing immunization cards, and discussing with the next appointment were in. 68% of the vaccinators appropriately practiced BCG and measles preparation using proper diluent and reconstitution. 91% of the vaccinators adequately explained the mothers' questions, 38% of them allowed mothers to ask questions, and only 7% of them used education materials for explanation.

Delivering all key messages was assessed using the vaccinator's discussion with mothers about the key messages. Only 6% of vaccinators deliver all key messages such as information about antigens, the purpose of each antigen, potential adverse effects, card handling, and the next appointment. Of the key messages, discussion about the next appointment was practiced by 82% of the vaccinators. At the same time, information about the purpose of the provided antigens and their potential adverse Table 5.5.

Table 5.5: Vaccination session safety during the preparation/pre-administration phase across regions, facility types and settings in Ethiopia, 2023.

Regions											Key messages						Communication			Proper infant assessment				
	Session place cleanness	Availability of table	Availability of chair	Availability of two/more vaccinators	Correct BCG diluent	Correct Measles diluent	Correct BCG reconstitution	Correct Measles reconstitution	Proper client greeting	Ensure privacy	About Antigen	Purpose	Adverse effects	card handling	Appointment	All key messages provided	Allow asking	Properly explain questions	use education materials	Review child immunization card.	History of AEFI/side effects	Medical condition	Proper infant assessment	Total number of sessions observed
Addis Ababa	100	100	100	84	80	59	76	59	26	51	60	24	35	60	82	6	49	100	0	93	51	71	2	99
Afar	68	81	97	22	89	89	86	89	81	63	55	32	48	51	56	4	56	95	34	84	11	79	1	150
Amhara	69	80	79	46	56	67	55	67	66	60	60	50	50	49	85	15	43	94	14	87	44	63	11	706
Benishangul Gumuz	71	73	77	62	41	56	53	60	81	69	77	74	59	43	77	12	32	91	5	78	25	68	5	146
Dire Dawa	70	84	84	41	37	51	37	50	32	41	13	15	18	49	87	1	4	100	1	79	0	61	0	76
Gambela	78	89	89	100	69	81	69	81	83	72	67	67	67	76	62	22	50	94	11	92	32	88	6	36
Harari	75	70	75	57	55	65	57	58	84	29	17	3	20	40	77	0	34	83	5	96	4	71	0	100
Oromia	82	93	91	44	56	68	57	68	66	52	33	34	24	53	89	4	41	96	6	85	25	64	3	713
SNNP	64	85	86	48	47	55	47	57	75	49	23	26	32	58	88	2	45	92	11	82	8	52	0	476
Sidama	69	93	93	62	75	75	76	77	46	36	2	9	22	48	97	1	44	96	13	87	48	58	4	201
Somali	64	96	90	34	51	62	46	60	72	46	65	31	36	57	97	4	46	98	11	83	10	81	2	143
Southwest Ethiopia Peoples	80	96	89	56	71	62	71	62	68	61	36	38	31	55	89	5	45	96	13	84	17	90	3	108
Setting																								
Rural	70	84	84	45	54	65	55	65	71	52	40	37	36	53	88	7	44	96	12	84	28	66	5	2232
Urban	83	94	96	60	68	68	67	68	53	55	43	33	36	51	82	6	38	91	7	88	22	69	2	722

Facility Type																							
Public Hospitals	77	91	92	54	61	63	63	67	64	62	39	32	34	54	89	7	48	95	12	85	24	68	5	510
H/Centers	74	88	87	44	57	64	56	63	68	53	40	36	35	50	85	5	42	95	11	86	27	65	3	1879
H/posts	65	76	80	56	60	75	60	75	63	42	47	40	41	57	88	12	38	95	11	81	32	69	6	525
Private HFs	100	100	100	70	28	45	43	48	75	80	21	21	35	55	83	3	55	82	3	98	12	72	5	40
Total	73	87	87	48	58	65	58	66	66	52	41	36	36	52	86	7	42	95	11	85	27	67	4	2954

Southern Nations, Nationalities and People's region, HFs=Health Facilities, H/center=Health center, H/Post= Health post

Vaccination safety practice during vaccine administration

The vaccination safety during the intra administration phase was assessed through the injection safety practice of the vaccinators. 84% of the vaccinators calm the baby during vaccination administration. Considering specific antigens provided, 98% vaccinators provided BCG correct dose, 91% correct route, 89% correct site, and 92% used a 0.05 AD syringe. 100% vaccinators provided measles correct dose, 92%, 91% correct route (correct site), and 98% used 0.5 AD syringe. Rota was provided at the correct dose (99%), correct route (93%), and correct site (91%). Pentavalent vaccine was provided at the correct dose (100%), correct route (93%), and correct site (92%), while 1% was provided without considering the above three criteria. OPV was provided at the correct dose (99%), correct route (94%), and correct site (92%). PCV was provided at the correct dose (100%), correct route (93%), and correct site (93%) Table 5.6.

Table 5.6: Vaccination safety practice during administration in Ethiopia across regions, setting and facility type, 2023

Regions	BCG (603)					Measles (645)					Rota (1548)				Pentavalent (2155)				OPV (1892)			PCV (2049)			
	Position and calm	Correct dose	Correct route	Correct site	None	Used 0.05AD syringe	Correct dose	Correct route	Correct site	None	Used 0.5 AD syringe	Correct dose	Correct route	None	Correct dose	Correct route	Correct site	None	Correct dose	Correct route	None	Correct dose	Correct route	Correct site	None
Addis Ababa	83	94	94	94	0	74	100	100	100	0	100	100	100	0	100	100	100	0	100	100	0	100	100	100	0
Afar	83	85	85	71	1	82	94	85	74	0	94	96	91	5	96	91	89	5	99	97	4	96	92	93	7
Amhara	89	87	87	89	0	99	100	93	93	1	100	99	91	1	100	89	88	1	99	89	0	100	89	88	0
Benishangul Gumuz	99	100	100	100	7	100	100	100	100	0	97	100	100	1	99	100	99	2	100	100	0	100	100	99	0
Dire Dawa	70	100	100	100	0	100	100	100	100	0	100	100	98	2	100	98	98	4	100	98	0	100	98	98	0
Gambela	97	100	100	100	0	100	100	100	100	0	100	100	100	0	100	100	100	1	100	100	0	96	100	100	0
Harari	94	100	100	62	0	100	100	95	100	0	100	100	100	0	100	100	99	0	100	100	0	100	100	97	0
Oromia	82	95	95	95	2	87	100	94	93	2	98	100	94	1	100	94	92	0	100	95	2	100	95	93	0
SNNP	72	84	84	88	1	93	100	87	87	0	91	100	91	0	100	90	90	0	100	90	0	100	90	90	0
Sidama	84	98	98	98	0	100	100	89	89	2	100	99	93	1	100	95	94	0	100	98	0	100	94	93	0
Somali	80	89	89	83	6	89	100	96	96	0	100	100	93	0	100	97	96	2	100	96	1	100	96	96	1
Southwest Ethiopia People	92	100	100	100	0	88	100	90	90	0	100	99	100	0	99	100	95	0	98	100	0	100	100	95	0
Setting																									
Rural	85	98	88	88	1	94	99	90	90	1	97	99	93	1	100	93	91	1	99	93	1	100	93	92	0
Urban	81	98	98	91	1	88	100	97	95	1	99	100	95	0	100	96	95	1	100	96	0	100	96	95	0

Facility Type																								
Public Hospitals	83	98	96	94	2	89	100	89	87	0	100	100	93	2	100	92	92	2	99	93	1	100	92	92	0
Health Centers	84	98	91	88	1	91	100	92	91	1	97	99	93	0	99	93	91	0	100	93	0	100	93	92	0
Health posts	82	97	89	87	1	98	99	95	94	1	98	99	95	0	100	95	95		99	96	0	100	94	94	0
Private HFs	100	100	67	67	0	100	100	100	100	0	100	100	100	7	100	100	100	0	100	100	0	100	100	100	0
National	84	98	91	89	1	92	100	92	91	1	98	99	93	1	100	93	92	1	99	94	0	100	93	93	0

SNNP=Southern Nations, Nationalities and People's region, HF=Health Facilities, BCG= Bacille Calmette - Guérin (BCG) vaccine, OPV=Oral Poliovirus vaccine, PCV= Pneumococcal Conjugate Vaccine

Vaccination safety practice - post-administration

The post administration practices assessed the practice of vaccinators in managing the post immunization activities like needle recapping and safety box utilization. 85% of the vaccinators practiced post-vaccination administration safety avoiding post-vaccination needle recapping. All the vaccinators in Addis Ababa (100%) avoided post-vaccination needle recapping, followed by Gambela (97%) and Dire Dawa (95%). Only 39% of the vaccinators avoided post-vaccination needle recapping in the Afar region.
Ninety percent of the vaccinators in the urban avoided post-vaccination needle recapping and followed by rural 84%. 86% of vaccinators from public hospitals, 86% health centers, 83% health posts and private health facilities practiced avoiding post-vaccination needle recap. Vaccinators at static, 86% avoided post-vaccination needle recapping whereas 82 in the outreach/mobile. 80% of vaccinators practiced proper safety box utilization: Addis Ababa (100%) followed by Sidama (90%) and Gambela (89%). Harari (44%) had the lowest proper safety box utilization. Providers from the urban setting (90%) had better proper safety box utilization than those from the rural settings (84%) Table 5.7.

Table 5.7: Post-administration vaccination safety practice by region, setting and facility type, and service delivery approach in Ethiopia, 2023.

	Avoid needle recapping	Proper safety box utilization	Total sessions observed
Regions			
Addis Ababa	100	100	95
Afar	39	69	147
Amhara	92	82	693
Benishangul Gumuz	92	69	144
Dire Dawa	95	79	76
Gambela	97	89	34
Harari	74	44	100
Oromia	81	85	708
SNNP	92	71	466
Sidama	76	90	201
Somali	83	85	142
Southwest Ethiopia Peoples	93	88	107
Setting			
Rural	84	79	2206
Urban	90	83	707
Facility Type			
Public Hospitals	86	84	469
Health Centers	86	80	1857
Health posts	83	77	521
Private HFs	85	90	39
Service delivery approach			
Static	86	83	2655
Outreach/Mobile	82	48	258
Total	85	80	2913
SNNP=South Nation, Nationalities and People region, HF=Health Facilities			

The regular staff rotation strategy held every 3, 6, or 9 months reported as one of potential barriers resulted in loss of trained and knowledgeable staff from the immunization unit. Those who joined the immunization unit by the rotation need technical and scientific training that can't be done as required. This affects the quality of vaccination sessions and decreases client satisfaction. In addition, distance and failure to store vaccines at the health posts affect vaccine quality as vaccine potency compromised during service delivery.

Maternal/caregiver satisfaction

Maternal/caregiver satisfaction towards vaccination service was assessed using 10-item questions with five Likert scales. The assessment was done among 2934 mothers who came to the health facilities for vaccination services.

The highest level of maternal satisfaction was reported among the Harari region (70%), while the lowest (23%) was from the Afar region. Service waiting time, room area and cleanness, service opening time, vaccinator availability, the respect given to clients, information sharing about antigens, information about the next appointment, and the previous child's experience following vaccination were the item questions used. On average, fifty percent of the respondents are satisfied with each dimension.
Figure 5.7 shows maternal satisfaction with vaccination services across regions, settings, and facility types. The overall satisfaction level with immunization service was 55%. 58% of mothers/caregivers from agrarian, and 55% from urban and pastoralist had better satisfaction.

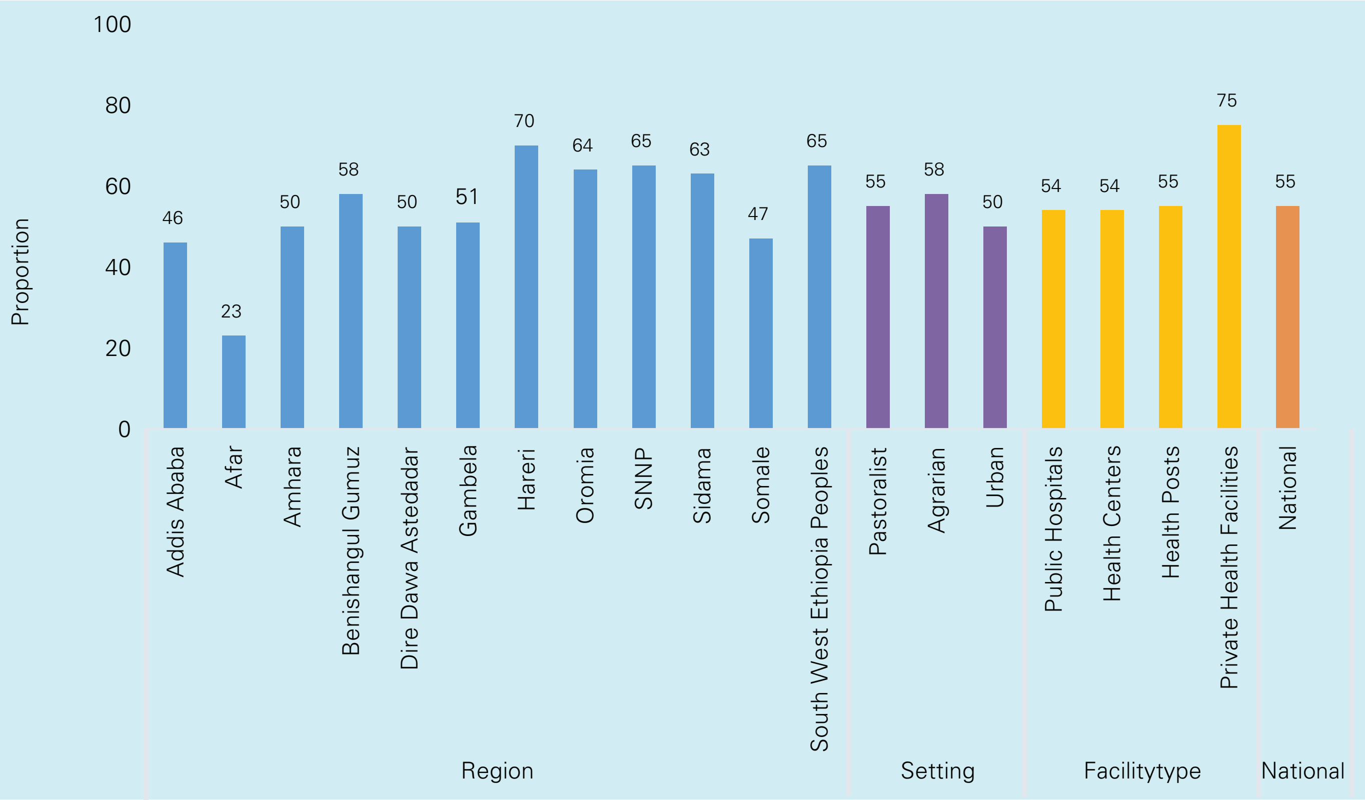

Figure 5.7: Mother's satisfaction on immunization services by regions in Ethiopia, 2023

Vaccination Service Coverage

Child vaccination coverage

The child vaccination coverage was assessed to determine the uptake of the scheduled immunization vaccines by either confirming through the mother's verbal confirmation or verifying with documents such as the immunization card and register. A weighted sample of 6146 children aged 12 to 23 months used to estimate the coverage for MCV2.

Results from the survey showed that a mere 43.9% of children between the ages of 12-23 months had received all recommended routine vaccinations. However, about 24.7% of children identified as having received none (zero dose). The overall coverage rates for the first and third doses of the pentavalent vaccine were reported at 75.3% and 64.7%, respectively. Only 60.3% of children received the first dose of the measles-containing vaccine (MCV1). About 14.1% of children dropped out before receiving their third dose of the pentavalent vaccine, while 19.9% of children failed to complete their measles vaccination series Figure 5.8.

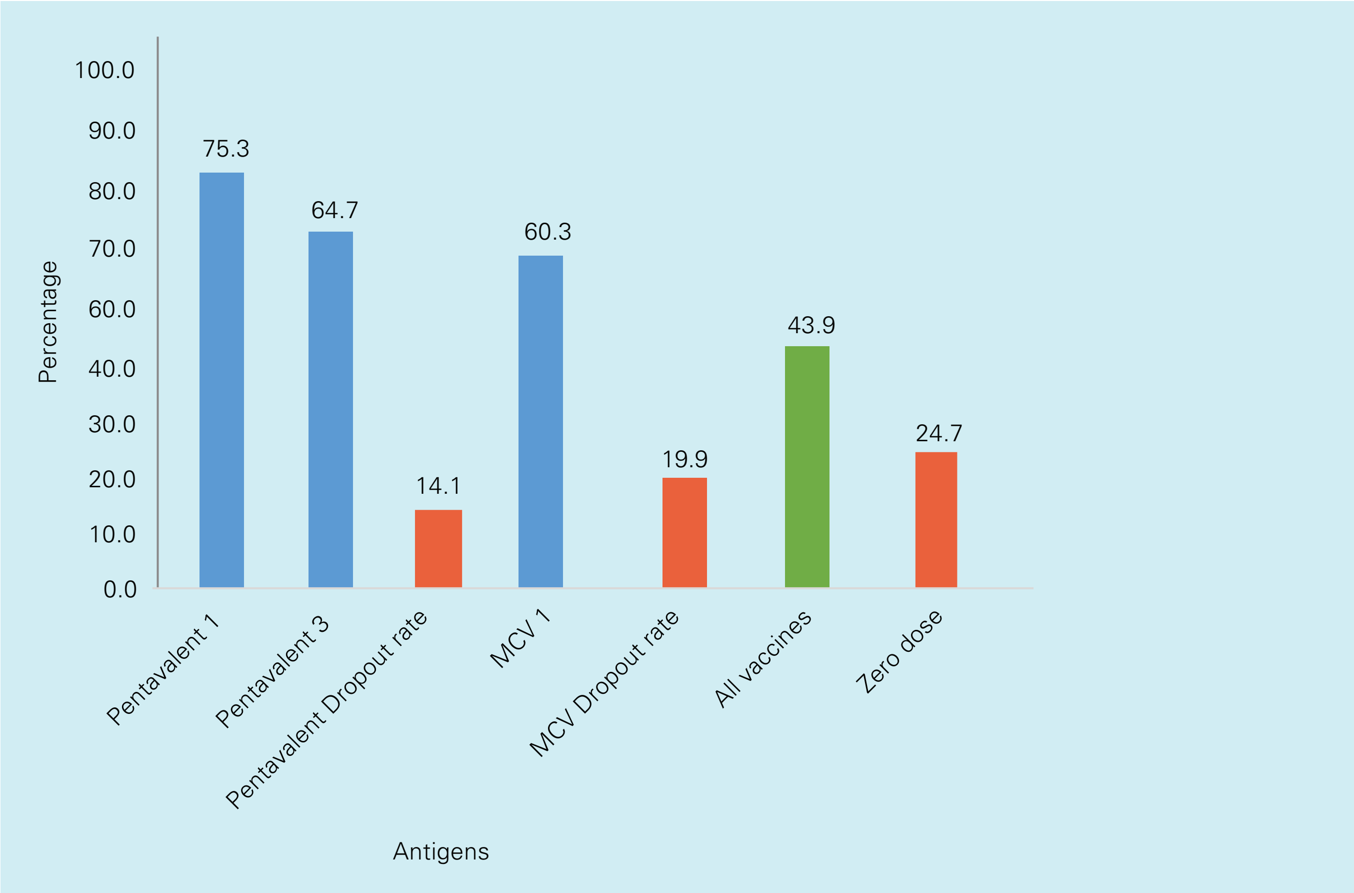

Figure 5.8: National vaccination coverage on selected antigens, dropouts and zero-dose among children aged 12 to 23 months in Ethiopia, 2023.

.

Vaccination coverage by verification means

Vaccination status was confirmed using document verification (immunization cards, registration records) and mothers/caregivers' verbal response. All vaccine antigens coverage ranges from 61.7 to 68.9% using verbal responses whereas the verified vaccine uptake ranges from 31.1to 37.4% Figure 5.9

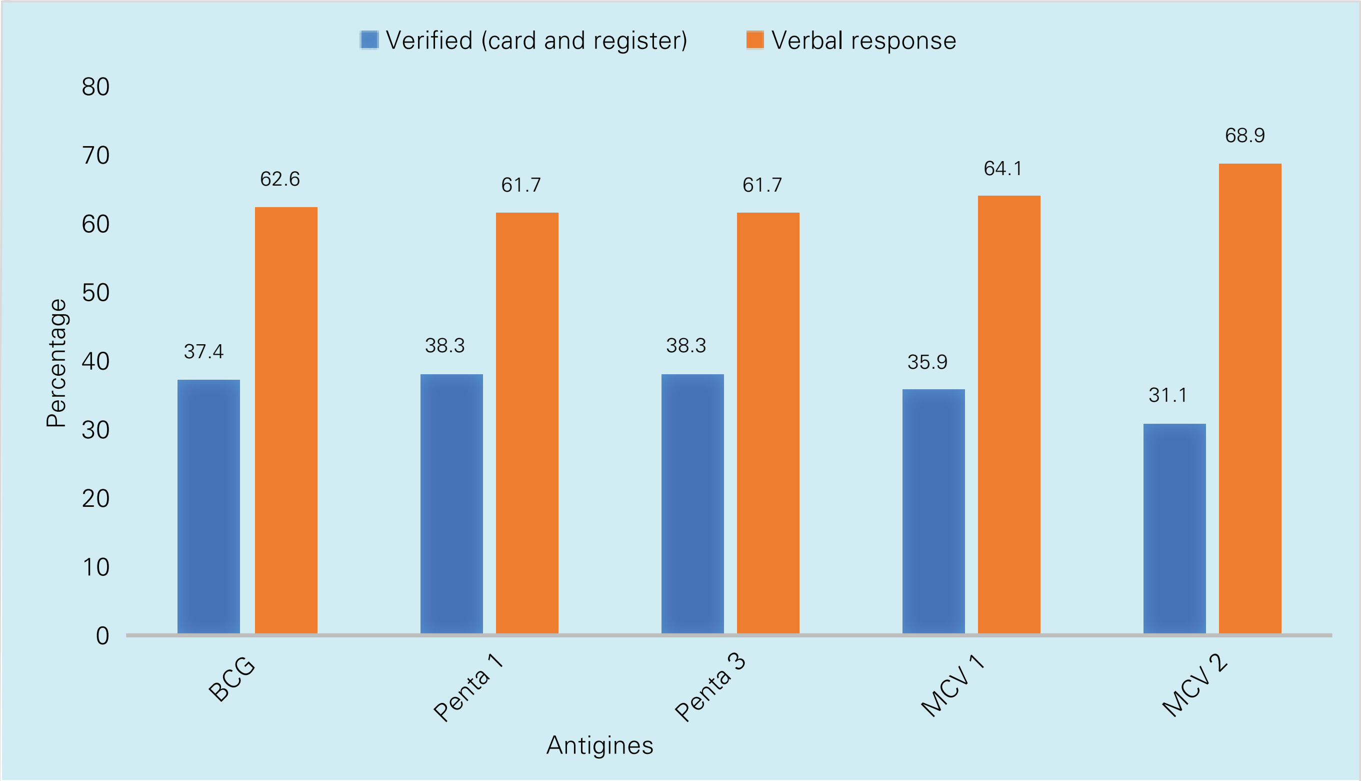

Figure 5.9: Child vaccination verification approaches by antigens in Ethiopia, 2023

Vaccination coverage for children aged 12-23 months across socio-economic and demographic variables The survey revealed that the vaccination coverage varied across regions. Vaccination rates differed based on various factors such as birth order, location of residence, mother's educational level, financial standing, history of antenatal care visits, and child place of delivery Table 5.8.

Table 5.8: Vaccination coverage aged 12-23 months across selected socio-demographic and economic characteristics in Ethiopia, 2023.

	BCG	DPT-HePB-HiB			Polio					PCV			Rotavirus		MCV	All vaccines	Zero Dose	#of Children weighted	# of Children
		1	2	3	0	1	2	3	IPV	1	2	3	1	2	1				
National	72.9	75.3	70.3	64.7	43.4	74.9	70.0	64.9	64.3	73.5	70.5	65.3	74.9	70.1	60.3	43.9	24.7	6146	5939
Region																			
Addis Ababa	97.9	99.0	98.9	97.1	82.4	98.0	96.1	93.1	93.1	99.0	98.0	97.1	99.0	98.0	97.1	74.3	1.0	102	339
Afar	53.9	60.0	44.7	33.9	33.9	58.3	43.1	32.3	29.2	59.7	44.7	33.9	60.0	44.6	24.4	12.8	40.0	65	360
Amhara	74.6	75.8	71.4	67.1	44.0	76.0	71.9	67.8	67.5	76.1	73.0	68.0	75.4	71.8	67.6	45.3	24.2	1367	1029
Benishangul Gumuz	77.1	81.9	76.3	70.3	51.0	80.7	75.9	70.3	67.9	80.7	75.9	71.5	80.7	75.1	62.3	47.0	18.5	88	249
Dire Dawa	85.9	95.2	90.3	85.9	66.5	95.2	90.3	85.9	81.1	95.2	90.3	85.9	90.3	85.9	81.1	58.3	4.9	21	206
Gambela	76.3	81.0	76.3	67.2	52.6	81.0	76.6	66.8	62.0	83.2	76.3	65.7	81.0	76.3	61.7	38.0	19.0	21	274
Harari	77.0	77.0	69.1	61.8	38.3	77.0	77.0	69.1	61.8	77.0	69.1	53.9	77.0	69.1	53.5	23.0	23.0	13	217
Oromia	69.6	72.3	68.7	61.7	34.4	71.8	68.3	62.4	62.0	68.5	68.5	62.7	72.2	68.5	55.4	40.5	27.7	2900	1563
SNNP	78.1	80.0	72.3	68.2	59.9	79.9	72.0	68.0	65.0	80.0	72.1	68.7	79.4	71.6	63.9	50.5	20.0	1109	606
Sidama	81.4	84.5	78.6	71.0	45.1	83.1	76.3	69.6	68.2	83.7	76.9	70.4	83.7	77.8	67.6	42.0	15.5	135	355
Somali	52.7	59.3	51.2	45.2	45.8	57.8	49.7	44.4	43.7	59.9	51.8	45.8	57.8	49.7	40.9	46.1	40.7	135	469
Southwest Ethiopia Peoples	81.3	82.7	76.5	70.6	53.7	81.6	74.3	68.4	67.3	81.6	75.4	69.5	81.6	74.3	63.6	39.7	17.3	190	272

Birth order																			
1st	76.0	78.5	73.6	66.6	43.5	78.3	73.2	69.2	68.5	76.3	73.9	69.2	78.4	72.8	62.1	46.4	19.4	1750	1709
2nd & 3rd	74.3	77.9	73.0	68.2	45.7	76.3	72.5	67.9	67.3	76.1	74.8	68.5	77.1	75.1	61.3	44.9	22.8	2205	2206
4th &5th	74.7	75.5	71.5	65.2	41.4	75.4	71.1	65.1	64.0	75.4	70.4	64.7	74.9	70.6	59.1	43.0	24.4	1180	1092
6th &above	62.2	63.5	58.3	52.4	40.8	65.4	57.9	52.8	51.2	60.7	55.4	52.4	64.0	53.9	46.6	38.5	38.0	1011	932
Residence																			
Urban	81.0	82.3	77.0	74.1	54.3	81.2	75.1	75.1	74.7	79.3	79.5	76.1	81.4	77.1	71.1	54.7	18.6	1457	1606
Agrarian	71.4	74.1	69.6	62.6	38.6	73.9	69.6	63.3	61.6	72.5	68.8	62.5	73.5	69.1	55.5	40.1	25.6	4235	3306
Pastoralist	60.5	63.5	57.3	52.1	53.1	63.0	57.4	51.4	55.7	64.2	57.9	56.6	66.7	56.5	48.2	43.7	36.7	454	1027
Mother's education																			
No education	66.2	70.0	64.5	56.3	39.4	69.0	63.6	57.8	57.3	68.1	64.1	56.7	68.9	62.7	51.4	38.8	33.2	2904	2888
Primary	77.0	78.2	74.1	69.1	44.6	78.6	73.3	69.2	67.6	77.0	73.7	70.6	77.8	75.2	60.7	46.0	18.8	1902	1693
Secondary	81.7	82.3	76.8	74.8	46.8	82.1	81.0	74.9	73.0	80.4	78.8	75.3	83.2	79.3	71.2	47.9	15.1	890	870
College and above	81.8	83.6	81.9	79.7	58.6	83.6	76.9	78.9	79.9	80.6	82.4	80.0	85.5	79.1	73.3	60.2	13.7	450	488

Wealth status																			
Lowest	69.9	73	66.9	61.9	42.2	72.8	66.4	60.8	60.6	71.2	66.7	60.2	71.5	66.9	58.8	43.5	27.5	1101	1261
Second	69.1	72.9	67.9	64.6	43.2	73.3	69.6	61.7	63.0	71.9	66.8	64.7	73.1	67.3	55.8	44.5	26.7	1140	1139
Middle	72.8	75.0	73.1	61.7	39.6	73.1	69.5	63.6	63.7	72.4	71.5	63.1	77.5	69.5	56.7	39.6	26.6	1319	1202
Fourth	75.5	74.6	71.4	65.9	42.9	76.9	70.8	68.3	65.2	74.1	72.0	68.2	75.2	71.6	57.1	42.1	23.1	1321	1171
Highest	76.3	80.5	72.2	68.6	49.4	77.8	73.5	71.0	68.6	77.4	74.7	69.8	76.6	74.5	65.1	50.1	20.2	1265	1166
ANC visit																			
Yes	77.8	80.3	75.9	70.1	45.6	79.7	75.3	71.0	70.1	78.2	76.2	71.0	79.9	75.6	63.7	46.6	16.9	4871	4483
No	46.5	48	41.1	34.7	28.5	48.6	41.8	35.5	32.2	47.3	39.3	33.7	47.4	40.8	33.0	26.2	63.7	807	980
Health facility																			
Health facility	79.7	80.9	76.8	72.0	48.4	81.0	76.2	73.0	72.2	79.5	77.8	73.4	80.8	77.8	65.7	48.9	17.1	4035	3795
Home	57.6	63.1	56.3	48.1	30.4	61.4	56.4	48.7	46.5	59.8	53.9	46.9	61.6	53.0	43.4	31.1	39.3	1643	1668

BCG=Bacille Calmette-Guérin vaccine, DPT -HePB-HiB= Diphtheria, Pertussis, Tetanus, Hepatitis B and Haemophilus influenza type b vaccines, PCV=Pneumococcal Conjugate Vaccine, IPV=Inactivated Poliovirus, MCV=Measles Conjugated Vaccine

Pentavalent coverage and dropout across regions

The survey revealed that pentavalent vaccination coverage varied across different regions. In Afar and Somali regions, pentavalent 3 coverage falling below 50% high issues with access to and utilization of vaccinations in these areas. Furthermore, there is a concerning dropout rate of Penta 3 vaccinations across several regions showing a high number of defaulters. Only Addis Ababa meets the national threshold for dropout rates, with others such as southwest Ethiopia Peoples, Somali, Amhara, and Harari regions reporting double-digit dropout rates. The highest rate of defaulters found in the Afar region (43.5%), where almost half of the children did not complete the vaccination schedule Figure 5.10.

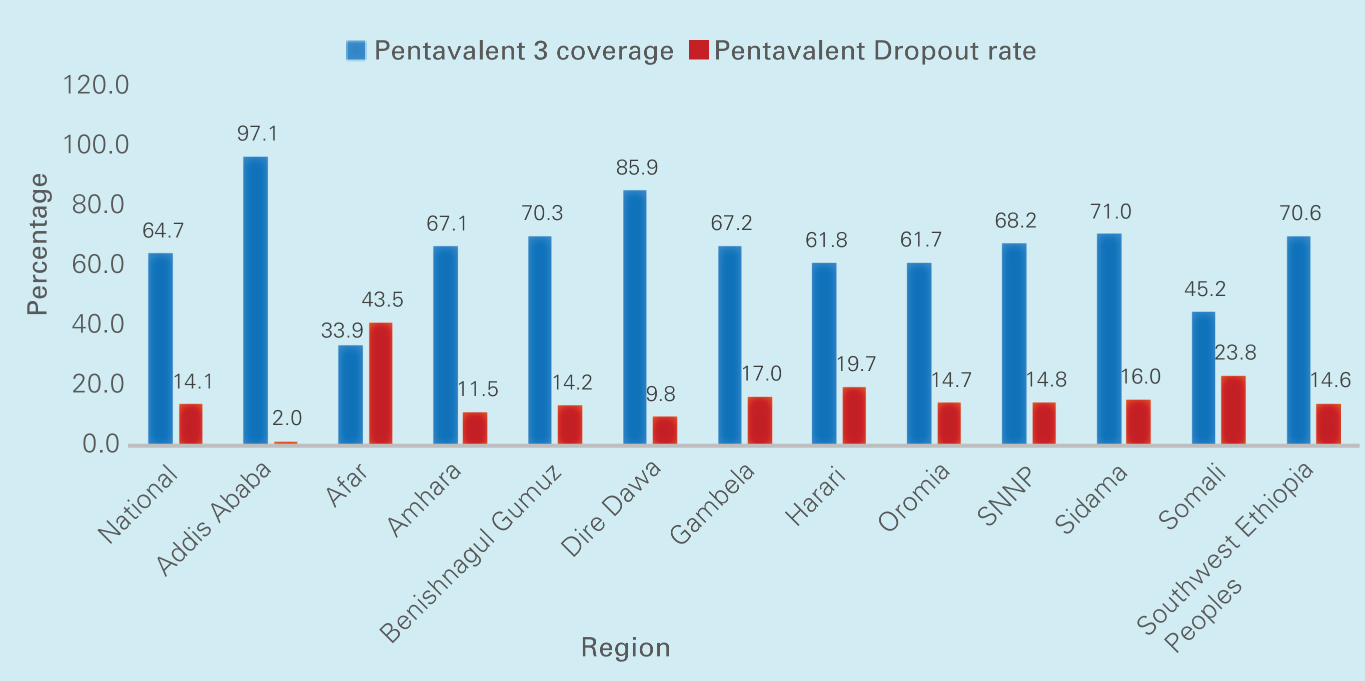

Figure 5.10: Pentavalent 3 coverage and drop-out rates by region in Ethiopia, 2023

Pentavalent coverage across demographic variables

The utilization of pentavalent 3 vaccination was also influenced by factors such as birth order, place of residence, maternal education level, attendance at antenatal care visits, and location of delivery. Higher coverage of pentavalent 3 vaccination were noted among first-born children living in urban areas, whose mothers had higher levels of education, had a history of attending antenatal care visits, and delivered at healthcare facilities Figure 5.11.

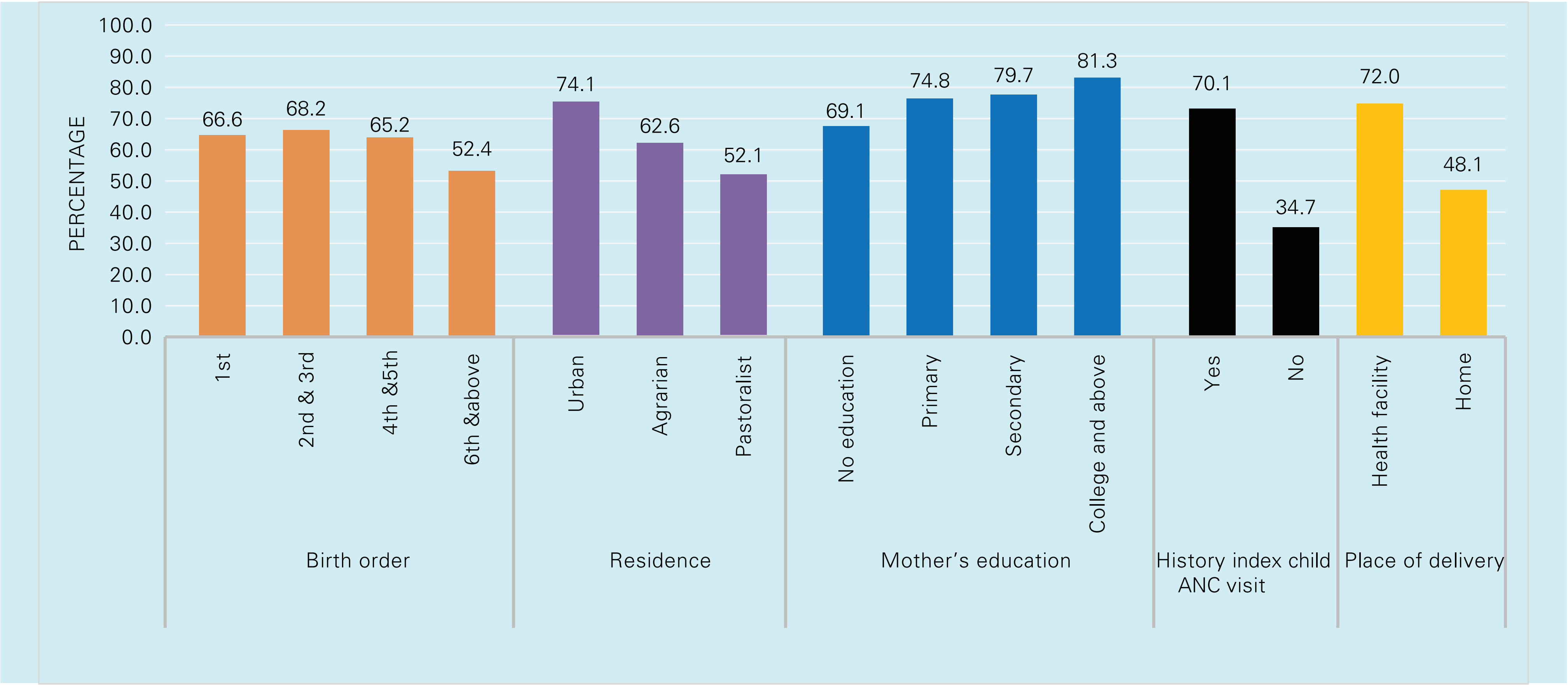

Figure 5.11: Pentavalent 3 coverage by selected characteristics in Ethiopia, 2023

All vaccines/Full immunization across regions

There was a disparity in the distribution of full vaccination coverage among different regions. The percentage of children receiving all recommended vaccines varied from 12.8% in Afar to 74.9% in Addis Ababa. Moreover, regions such as Harari, Oromia, Somali, southwest Ethiopia People, and Gambela had full vaccination coverage below the national average, 43.9% Figure 5.12.

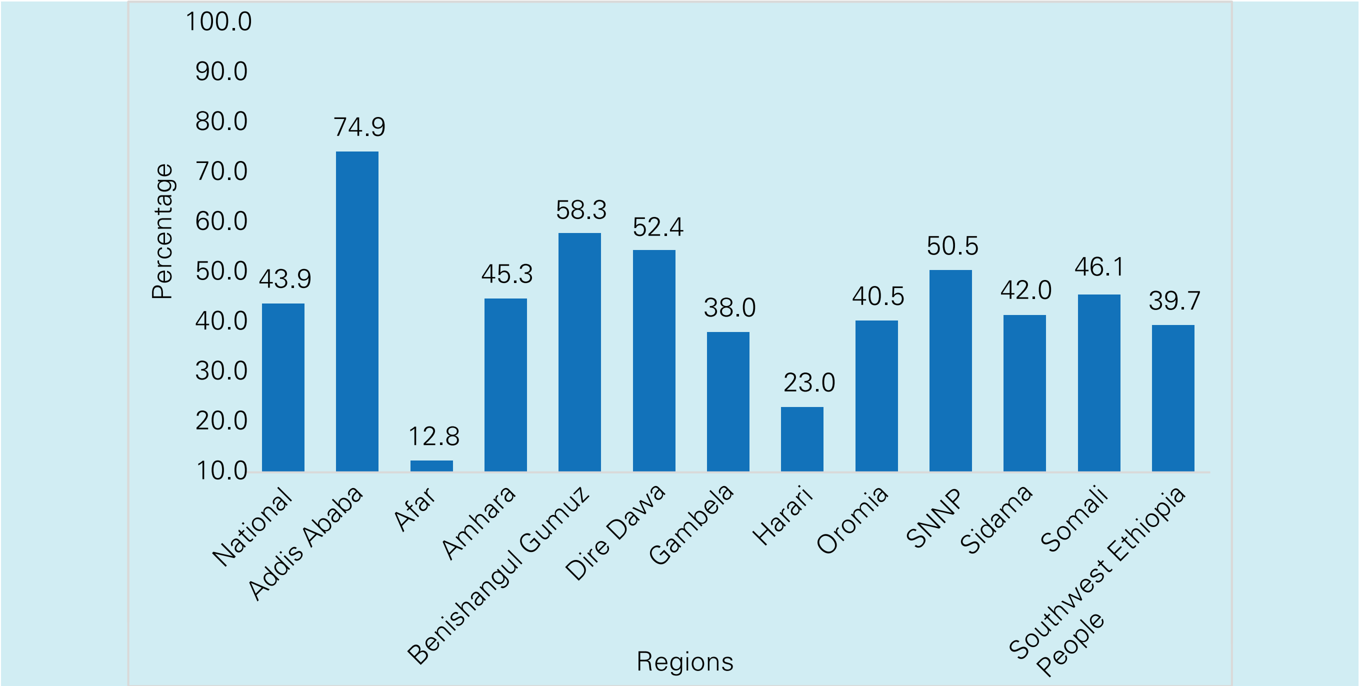

Figure 5.12: Full immunization/all vaccines coverage by regions in Ethiopia, 2023

All vaccines/full immunization across selected demographic variables

Children who were the eldest in their families, residing in pastoral areas, and whose mothers had limited education displayed lower rates of full immunization coverage compared to other children. Additionally, those who did not receive antenatal care (ANC) follow-up and were born at home had a lower uptake of all recommended vaccines in comparison to their peers Figure 5.13.

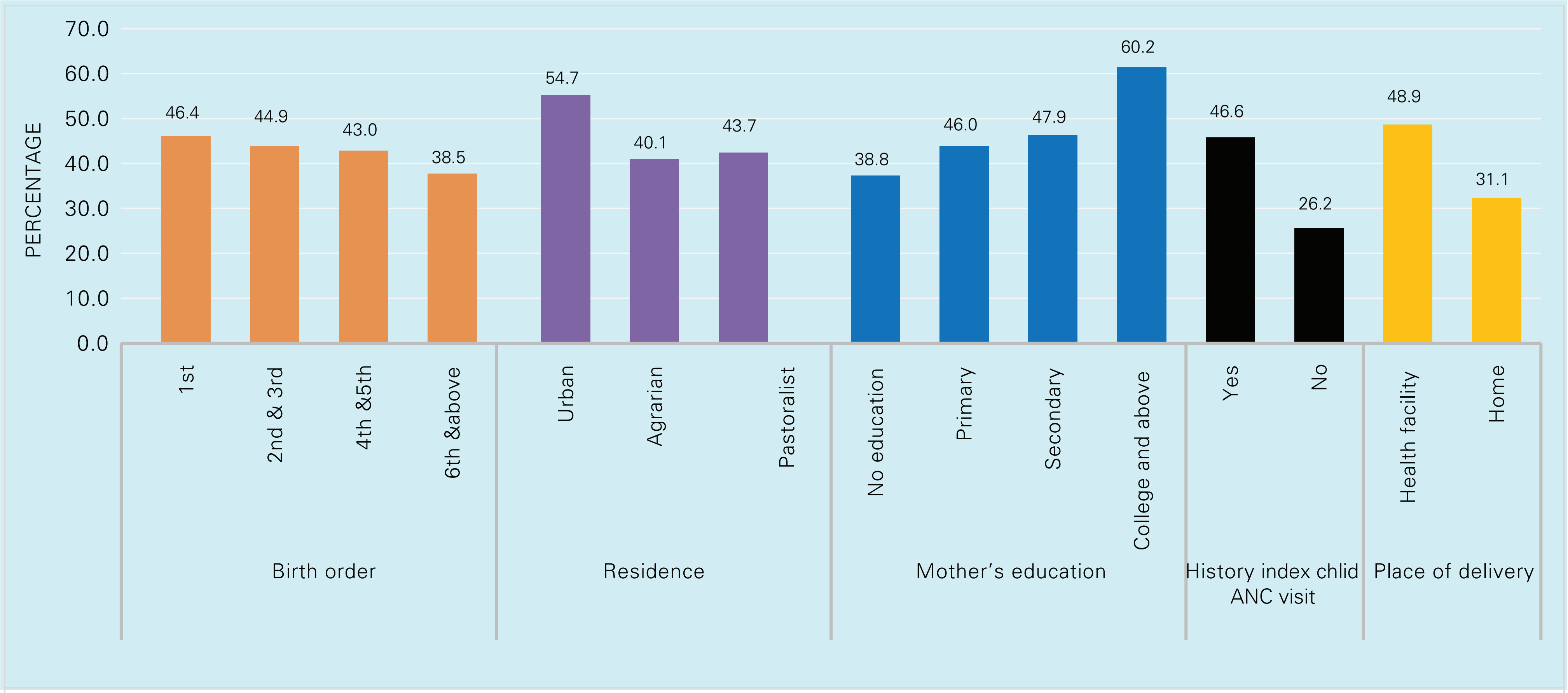

Figure 5.13: Full immunization/all vaccines coverage by selected characteristics in Ethiopia, 2023

Zero dose

Results from the survey revealed that nearly a one from four children between the ages of 12 and 23 months have not received at least a single dose of a recommended vaccine. The city of Addis Ababa had the lowest percentage of zero dose children at 0.9%, followed by Dire Dawa at 4.9%. On the other hand, a significant number of children in Amhara (24.2%), Oromia (27.7%), and even more so in Somali (40.7%) and Afar (40%) regions did not received any doses of the vaccine Figure 5.14.

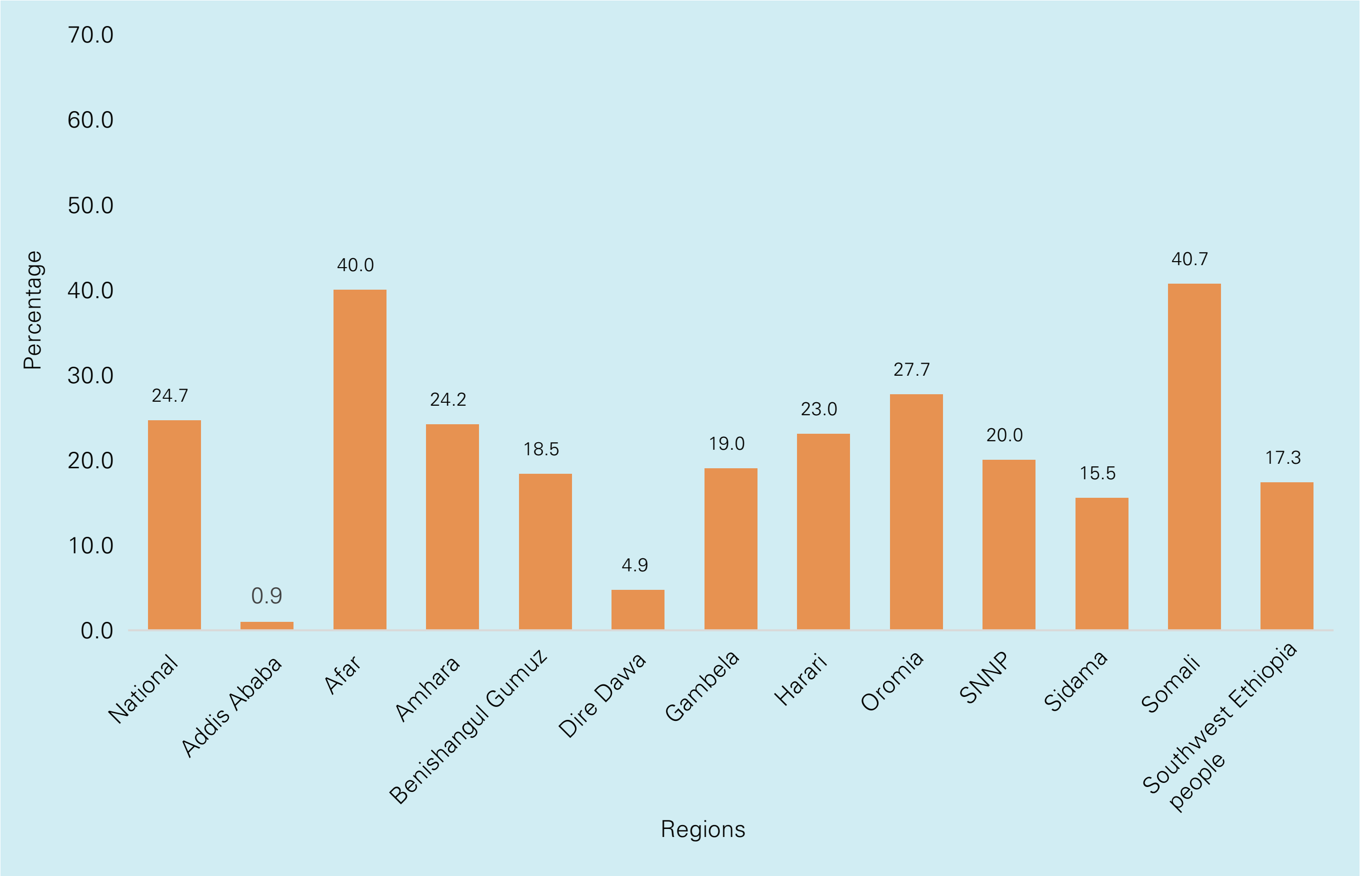

Figure 5.14: Zero dose coverage b regions in Ethiopia, 2023

Zero dose across selected demographic variables

Most children who did not receive any doses of vaccinations were older children, children in pastoral settings, and born to mothers with lower levels of education relative to their counterparts. Additionally, zero dose coverage was significantly higher among children whose mothers did not receive adequate antenatal care and gave birth at home for the index child compared to the comparators Figure 5.15.

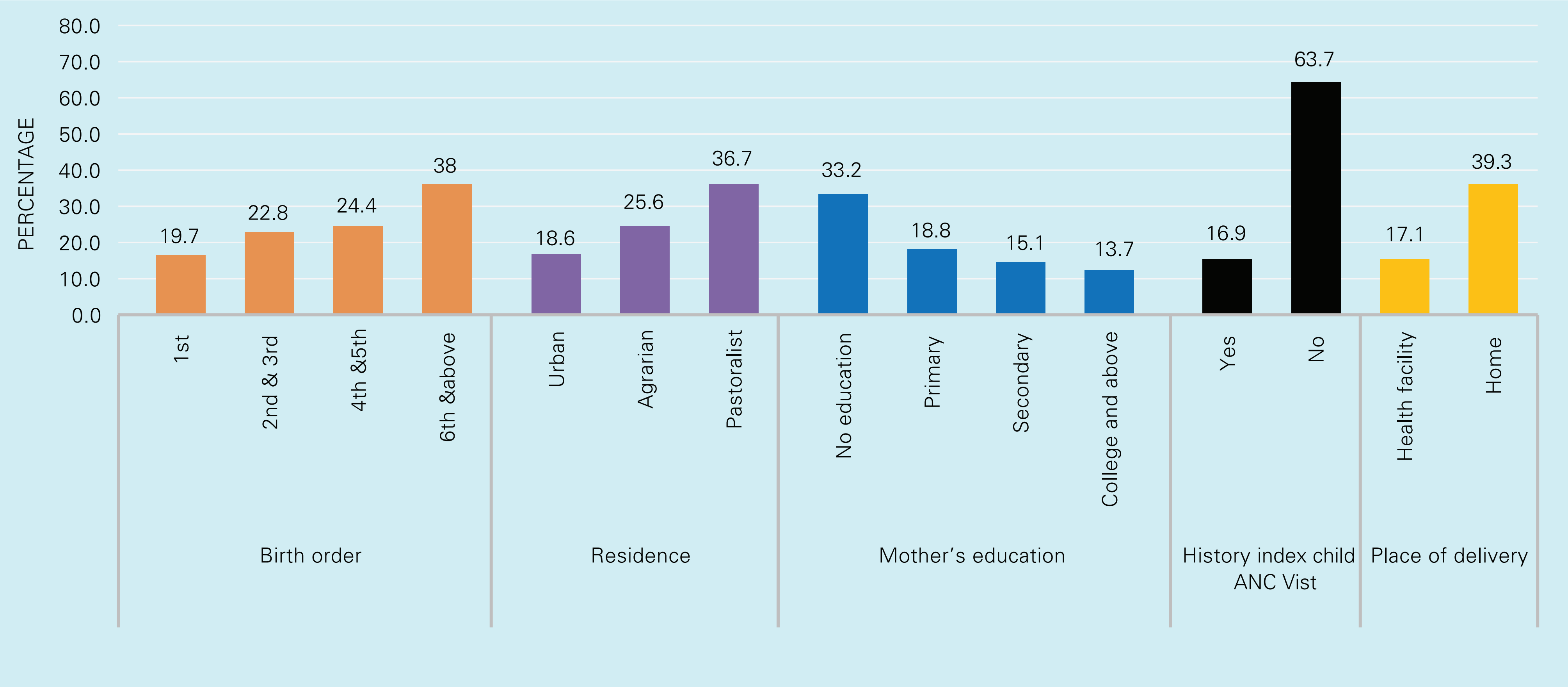

Figure 5.15: Zero dose coverage by selected characteristics in Ethiopia, 2023

MCV2 coverage

The uptake of MCV2 was assessed from children aged 23-35 months of age and the coverage was found 54.7%. The MCV2 coverage was better utilized by children from Addis Ababa (87.6%), while it was disproportionately low from Afar (25.8%) and Somali (42.2%) regions. Figure 5.16 also illustrates the MCV2 uptake/coverage across regions.

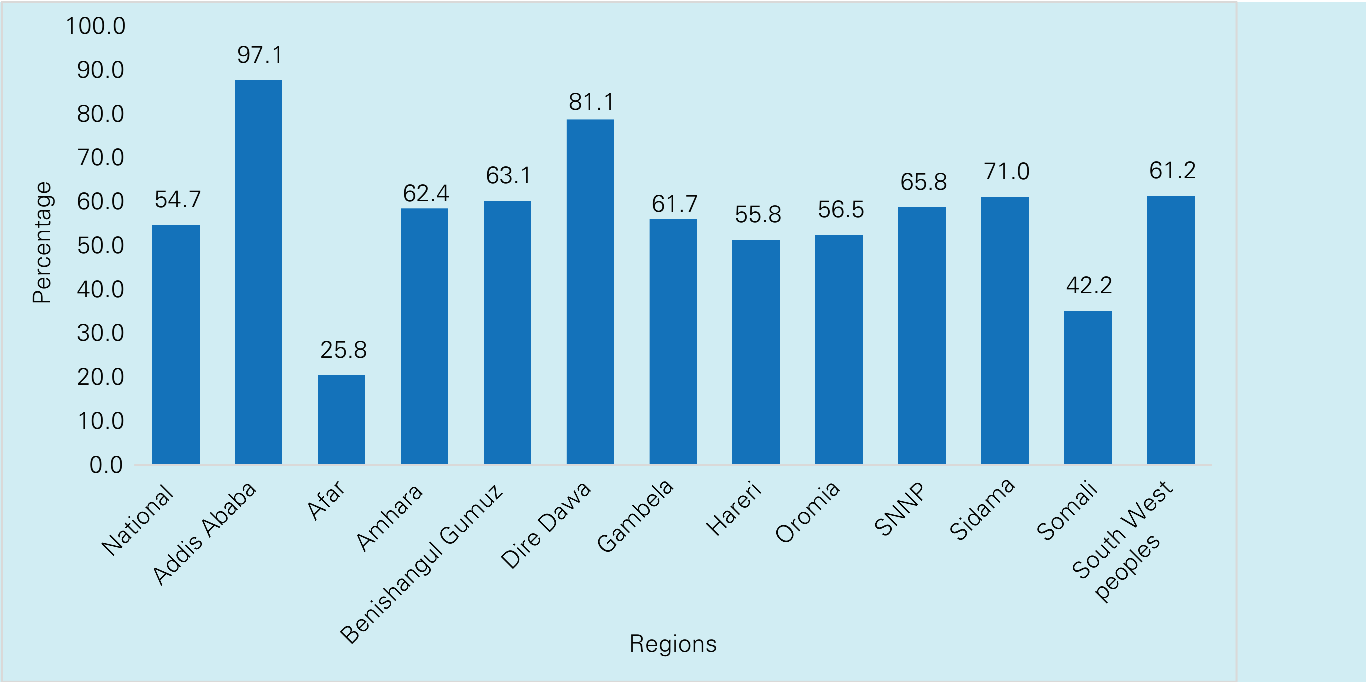

Figure 5.16: MCV 2 coverage by regions in Ethiopia, 2023.

MCV2 coverage across different variables

The MCV2 coverage variation was also observed among differences in birth order, setting, and mother's education status, history of ANC and place of delivery Table 5.9.

Table 5.9: MCV2 vaccination coverage aged 24-35 months across selected socio-demographic and economic characteristics in Ethiopia, 2023.

Characteristics	MCV2	#of Children weighted	# of Children
National	54.7	8147	7727
Region			
Addis Ababa	87.6	97	323
Afar	20.3	54	300
Amhara	54.2	1367	1158
Benishangul Gumuz	60.2	88	354
Dire Dawa	65.1	36	358
Gambela	50.2	30	277
Harari	51.4	18	294
Oromia	52.4	3764	2013
SNNP	58.7	1916	1047
Sidama	61.1	203	535
Somali	36.0	208	570
Southwest Ethiopia People	61.2	367	498
Birth order			
1st	57.5	1955	1866
2nd & 3rd	55.9	2845	2799
4th &5th	54.7	1919	1827
6th &above	48.5	1428	1235
Residence			
Urban	65.9	1818	1856
Agrarian	52.2	5747	4766
Pastoralist	46.0	582	1105
Mother's education			
No education	50.3	4235	4103
Primary	55.0	2385	2170
Secondary	67.6	1034	931
College and above	66.6	493	523
Wealth status	53.9		
Lowest	53.4	1440	1492
Second	54.6	1525	1578
Middle	54.6	1720	1539
Fourth	57.1	1760	1558
Highest	53.9	1702	1560
ANC visit			
Yes	58.8	6552	5853
No	26.2	943	1103
Place of delivery			
Health facility	62.0	5445	4846
Home	35.3	2050	2110

Maternal Td vaccination coverage

The maternal tetanus toxoid-diphtheria vaccination (Td) coverage was assessed among 13,174 childbearing mothers. 84.8% childbearing mothers received at least one dose of the Td vaccine (Figure Maternal Td vaccination coverage). Figure 5.17 shows the Td vaccine coverage with its respective doses.

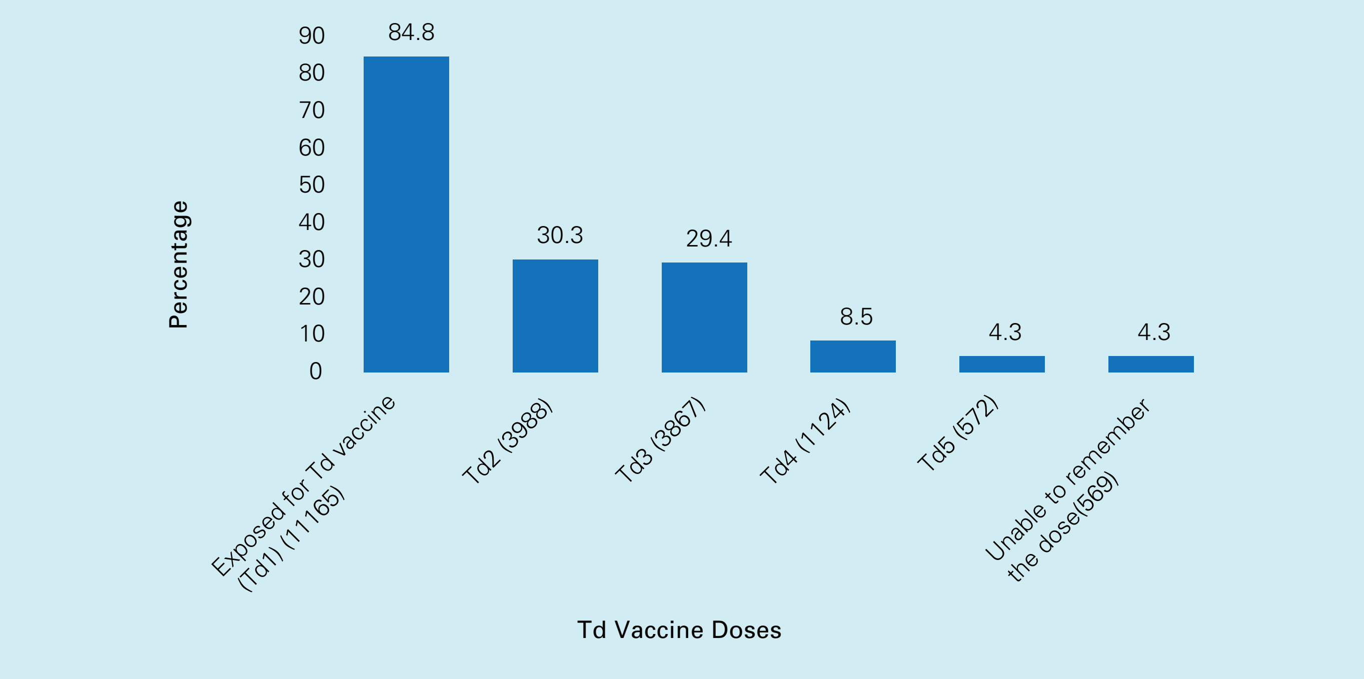

Figure 5.17: Td vaccination coverage by different doses in Ethiopia, 2023.

Maternal Td Vaccination coverage across different characteristics

Nearly one third (30.3%) of mothers received two doses (Td2), and only 4.3% of mothers received the fifth dose of the Td vaccine. Td vaccine coverage varied across regions, settings, and mothers' educational status. Td coverage was low in Afar and Somali regions, while mothers with no education (70%) compared to college and above (90%) Table 5.10.

Table 5.10: Td maternal vaccination coverage across basic characteristics in Ethiopia, 2023.

Background Characteristics	Exposed for at least Td 1 (11,165)	Td2 (3988)	Td3 (3867)	Td4 (1124)	Td5 (572)	unknown (569)	Number of mothers weighted	Number of mothers without weighted
National	84.8	30.3	29.4	8.5	4.3	4.3	13,174	12419
Region								
Addis Ababa	96.4	76.7	10.5	1.2	2.0	3.6	175	584
Afar	61.4	43.4	15.8	3.8	0.3	11.0	117	650
Amhara	83	41.1	30.4	7.5	2.7	5.2	2749	2067
Benishangul Gumuz	91.3	30.6	32.5	13.8	16.1	0.8	201	573
Dire Dawa	91.7	21.9	37.4	15.3	11.7	3.8	49	493
Gambela	86.8	31.7	40.0	12.6	5.8	1.5	52	476
Harari	80.2	26.6	40.9	11.5	7.3	4.8	27	445
Oromia	85.2	36.7	36.7	8.6	4.6	5.2	6077	3250
SNNP	90	27.0	34.9	15.5	7.8	6.0	2595	1418
Sidama	85.8	26.1	50.8	15.1	3.7	2.7	314	826
Somali	47.7	36.3	29.2	11.5	3.2	7.3	354	974
Southwest Ethiopia Peoples	85.4	35.7	34.8	10.4	8.1	1.1	464	663
Marital status								
Married and living together	84.9	35.5	34.7	10.2	5.2	5.1	12494	11740
Married However not living together	87.4	45.4	29.6	8.4	2.9	3.4	293	306
Currently not in a marital union	77.5	36.7	37.0	6.4	3.6	4.9	387	373
Religion								
Muslim	81.7	31.7	36.4	11.2	6.2	6.4	5574	5610
Orthodox	85.2	41.7	32.2	6.9	3.4	4.6	4784	4072

Protestant	89.2	33.5	35.6	13.1	5.9	3.6	2729	2665
Others	84.6	25.8	32.0	21.2	10.3	5.5	87	72
Residence								
Urban	90.6	35.6	32.6	9.9	5.0	6.9	2982	3138
Agrarian	84.2	35.9	35.5	9.9	5.5	4.1	9305	7350
Pastoralist	70.5	33.8	33.3	13.4	1.4	9.8	887	1931
Mother's education								
No education	78.6	35.0	33.9	10.4	5.1	6.0	6715	6445
Primary	90	35.5	36.7	9.6	5.0	4.0	3871	3437
Secondary	92.7	39.0	34.5	9.2	3.4	4.6	1752	1638
College and above	93.1	34.9	30.7	11.3	9.3	5.1	836	899
Occupation								
Housewife	85.1	35.5	35.3	10.0	4.6	5.3	9586	8977
Farmer	79.7	35.7	33.3	10.5	6.8	4.1	2091	1865
Merchant	87.6	36.6	32.9	11.0	4.3	6.2	569	577
Employed	92.5	34.9	32.7	9.1	9.2	6.0	734	814
Daily laborer	86.4	47.2	32.8	7.6	1.3	0.0	121	109
Other	80.7	51.1	18.6	8.2	2.0	0.3	73	77
Wealth quintile								
Lowest	82.4	32.9	33.6	11.0	6.4	6.2	2343	2547
Second	84.3	35.0	33.3	9.7	5.2	6.7	2580	2469
Middle	84.8	38.2	34.7	9.5	4.6	3.9	2773	2487
Fourth	85	38.0	35.3	8.8	4.6	4.1	2801	2458
Highest	86.9	33.9	36.1	11.4	5.0	4.9	2676	2458

SNNP=South Nation Nationalities and People region, Td=Tetanus and Diphtheria vaccine

HPV vaccination coverage

The HPV vaccination was assessed among 5,341 adolescent girls with age 15-18. 53.8% adolescent girls received at least one dose and a quarter of adolescent girls (25.9%) two doses of doses of HPV vaccine. Figure 5.18indicates the HPD vaccinations coverage by its respective doses

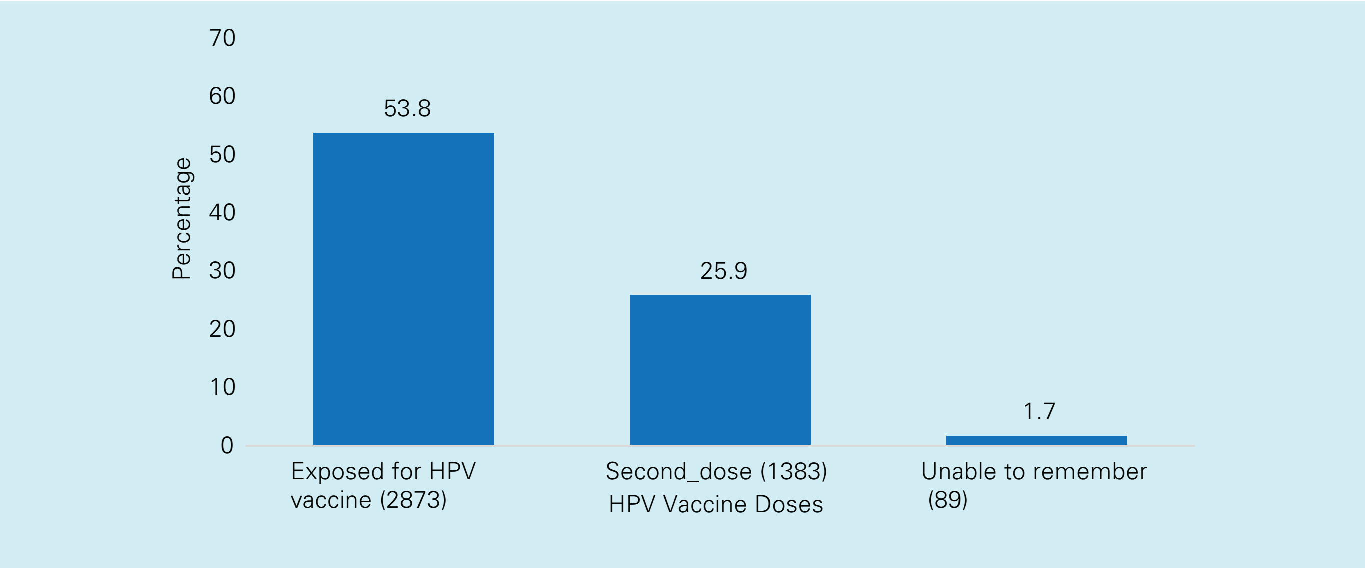

Figure 5.18: HPV vaccination coverage by doses in Ethiopia, 2023

HPV Vaccination coverage different characteristics

HPV vaccination coverage varies across regions, age, educational status, residency, and wealth status. HPV coverage at least one dose was found to be better in Benishangul Gumuz (73.6%) and Gambela (70.5%) regions, and lower in the Afar region (33.1%). 60% of adolescent girls aged 15 and 48.7% age 18-years old received least one dose of HPV vaccine. Adolescents who can read and write 60.4% and who can't read or write 18.5% received at least one dose of HPV vaccine. Adolescents attending school had 67.5% HVP vaccine uptakes, and 38.2% were non-school. Adolescents resided in urban (55.1%) and agrarian (57.3%) had better HPV vaccine uptakes, but only 36.5% of the pastoral. The Adolescents from the least poor family had 65.5% HPV vaccine uptake while only 40% were from the poorest family Table 5.11.

Table 5.11: HPV vaccination coverage across different background characteristics in Ethiopia, 2023.

	HPV exposed (at least first dose) (2873)	Two doses (1383)	Unable to remember (89)	Number of girls (weighted)	Number of girls (Un-weighted)
National	53.8	25.9	1.7	5341	5336
Region					
Addis Ababa	43.1	30.5	0.3	208	325
Afar	33.1	4.8	6.8	109	354
Amhara	59.0	27.7	1.0	1223	831
Ben/ Gumuz	73.6	41.0	0.4	65	227
Dire Dawa	41.8	27.6	0.5	29	196
Gambela	70.5	44.3	0.5	28	210
Harari	43.1	23.3	1.4	15	223
Oromia	55.8	28.5	1.8	2140	1315
SNNP	61.8	30.5	2.4	239	581
Sidama	65.3	33.5	1.2	351	343
Somali	32.8	8.0	2.4	727	451
Southwest Ethiopia Peoples	62.9	32.1	1.1	208	280
Age					
15	60	29.3	1.5	1849	1813
16	52.8	25.3	1	1396	1390
17	49.3	22.5	2.5	1086	1125
18	48.7	24.1	2.1	1010	1008
Can read and write					
Yes	60.6	29.7	1.7	4543	4557
No	15	4.4	1.3	798	779
Attending formal education					
Yes	60.4	29.5	1.8	4500	4524
No education	18.5	6.6	1.3	841	812
Highest level of education attended					
Primary	60.2	28.1	1.8	3616	3621
Secondary	61.3	32.7	1.6	884	903
Currently attending school					
Yes	67.5	33.9	1.9	3410	3458
No	38.2	15.8	1.3	1090	1066

Religion					
Muslim		3.8	0.9	1906	1679
Orthodox	46.7	18.8	2.5	2528	2441
Protestant	65	35.8	1.1	890	
Others	60.3	16.4	0.0	17	27
Residence					
Urban	55.1	30.6	1.0	1285	1411
Agrarian	57.3	28.2	1.5	3257	3059
Pastoralist	36.5	9.0	3.3	799	866
Relationship with household head					
Wife	37.3	13.4	2.7	297	288
Daughter	57.2	27.9	1.7	4173	4122
Sister	47.7	20.9	2.0	360	357
Other relatives	40.3	19.6	1.0	511	569
Mother's education					
No education	50.2	23.5	1.7	3940	3860
Primary	65.1	30.9	2.0	962	956
Secondary	66.7	43.4	1.1	245	283
College and above	63.1	36.3	0.8	104	139
don't know	45.7	19.3	0.9	90	98
Father's education					
No education	46.8	20.5	3.7	3101	3030
Primary	62.7	31.7	3.1	1391	1373
Secondary	69	39.5	1.5	496	545
College and above	67.8	37.2	1.2	197	238
don't know	47.1	24.1	2.0	156	150
Maternal Occupation					
Housewife	53.4	24.3	2.0	3404	3363
Farmer	52.6	26.7	1.0	1344	1314
Merchant	59.8	30.6	1.7	311	327
Employed	59.1	37.9	0.5	194	245
Other*	55.8	29.9	1.7	88	87
Paternal Occupation					
Farmer	52.9	24.4	1.8	3915	3776
Merchant	60	33.8	1.2	508	528
Employed	60.4	31.1	1.9	552	634
daily laborer	47.1	23.6	0.0	43	47
Others**	43.9	23.6	0.7	323	351

Living with					
parents	57	27.4	1.8	4349	4289
relative	44.1	20.9	1.3	714	737
husband	28.1	11.8	1.2	277	310
Wealth quintile					
Lowest	40.1	13.7	3.2	908	917
Second	47.7	20.8	1.0	805	816
Middle	54	27.8	0.9	872	848
Fourth	62.7	31.2	1.8	851	845
Highest	65.5	33.0	2.3	865	948

*daily laborer, religious teacher, Student

**Religious teacher, retired, unemployed

HPV=Human papillomavirus Virus, SNNP=South Nation Nationalities and People region, HH=Household

COVID-19 vaccination coverage

Among 13,812 respondents eligible for COVID19 vaccine, 55.9% received at least one dose of COVID-19 vaccine. Of these, 24.7% and 24.2% of respondents received one and two doses of COVID-19 vaccines, respectively. However, only 7% of the respondents received three or more doses of the COVID-19 vaccine. Figure 5.19 shows the coverage of COVID-19 by doses.

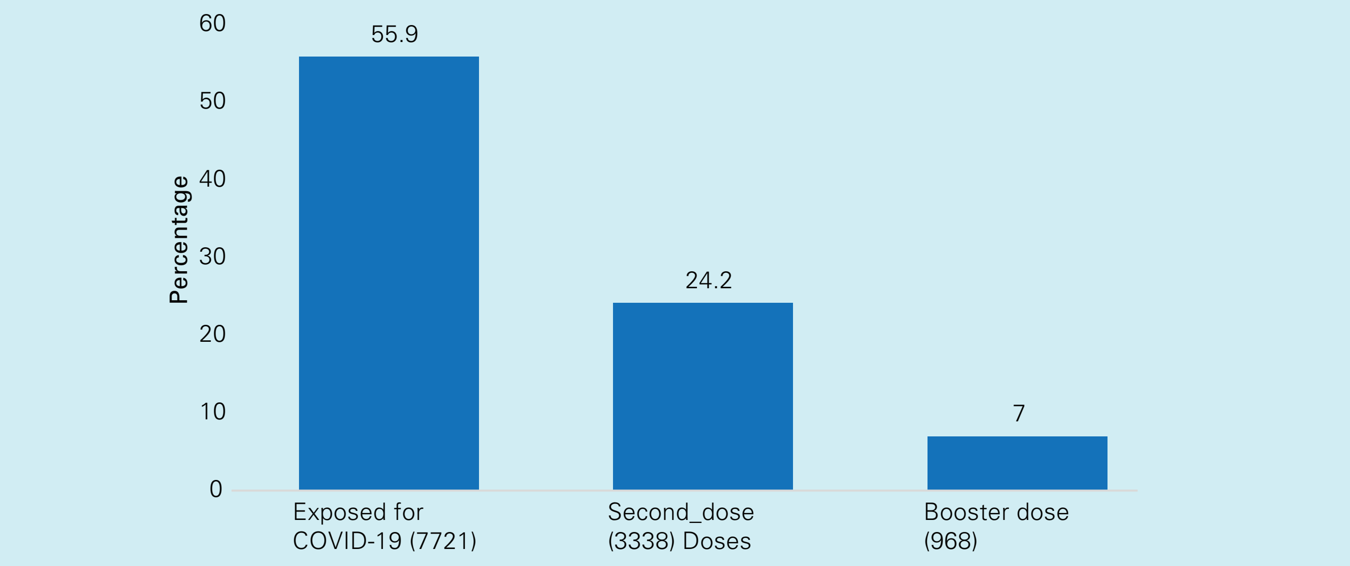

Figure 5.19: COVID-19 vaccination coverage by respective dose

COVID-19 vaccination coverage across different characteristics

The COVID-19 vaccination coverage varied across regions, settings, and sex of the respondents. The COVID-19 vaccine uptake was high at Benishangul Gumuz (78.9%), SNNP (73.5%), and southwest Ethiopia Peoples (74%) regions. However, COVID-19 vaccination uptake was low in Addis Ababa (31.8%), Harari (36.6%) and Afar (35.1%) regions. 63.3% of males and 53.3% females received at least one dose of COVID-19 vaccine Table 5.12

Table 5.12: COVID-19 vaccination coverage across different socio-demographic characteristics in Ethiopia, 2023.

Characteristics	COVID-19 vaccine (7721)	Single dose one (3415)	Two doses (3338)	Three or more (968)	# individuals weighted	# individuals without weight
National	55.9	24.7	24.2	7	13812	13843
Region						
Addis Ababa	31.8	16.4	9.8	5.6	520	732
Afar	35.1	28.7	5.8	0.6	275	655
Amhara	45.4	26.2	16.7	2.6	3082	2318
Benishangul Gumuz	79.8	13.3	41.9	24.6	164	630
Dire Dawa	65.4	17.8	28.2	19.4	74	567
Gambela	53.7	20.6	25.7	7.4	72	553
Harari	36.6	16.0	15.4	5.1	37	525
Oromia	57.4	26.5	24.7	6.2	5373	3582
SNNP	73.5	24.7	35.4	13.4	2273	1557
Sidama	58.7	23.2	27.9	7.6	614	890
Somali	43.4	17.8	20.6	5.0	886	984
Southwest Ethiopia Peoples	74.0	22.8	35.9	15.3	442	850
Sex						
Female	53.3	24.2	22.8	6.3	3463	3549
Male	63.3	26.2	28.1	9	10255	10194
Age						
20 - 29	50.2	22.4	20.6	7.3	841	866
20 - 39	55.4	24.6	24	6.8	10819	10856
40 - 59	61.2	26.9	26.5	7.8	1966	1932
>=60	54.7	18.8	24.8	11.1	186	189
Marital status						
Married and living together	56.5	25	24.5	6.9	12225	12234
Married However not living together	52	25.8	19.9	6.3	345	230
Currently not in a marital union	51.2	21.1	21.8	8.4	1192	1195
Residence						
Urban	42.9	21.3	16.8	4.8	3321	3515
Agrarian	61.3	26.7	26.8	7.7	9173	8354

Pastoralist	51.3	19.7	24	7.6	1318	1974
Educational status						
No education	55.1	24.1	24.8	6.2	6503	6523
Primary	59	27.1	24	7.9	4295	4204
Secondary	54	24	23.4	6.7	2020	1982
College and above	51.4	19.7	22.5	9.2	994	1134
Occupation						
Farmer	58.4	25.0	26.0	7.4	4571	4304
Housewife	54.1	24.6	22.8	6.7	6461	6520
Merchant	58.4	27.5	23.3	7.6	878	900
Gov employed	56.5	22.6	26.3	7.7	684	747
Private employed	47.5	14.9	26.3	6.4	237	280
Daily laborer	55	29.8	19.7	5.6	174	186
Self-employed	50.6	21.8	25.5	3.3	145	159
Other*	55.8	25.5	22.9	7.4	583	688
Wealth quintile						
Lowest	52	23.8	21.2	6.9	2587	2797
Second	58.3	25.3	26.3	6.7	2785	2760
Middle	56.7	26.4	23.1	7.2	2862	2768
Fourth	56.1	23.9	25.2	7.0	2873	2759
Highest	56.1	24.0	24.9	7.2	2705	2759

*--- unemployed, retired, religious teacher

SNNP=South Nation Nationalities and People region

5.5. Conclusions

Ninety-five percent of the health facilities provide immunization services. However, only about 38% of them offered these services on a daily basis. The assessment also discovered that the mean availability of tracer items was almost three-fourths, and only one in eight health facilities had all tracer items. This implies that the immunization service is not enough accessible and available for the users to use when and where they need.

In regards to the quality of immunization sessions, there was a lack of effective communication of important information and proper assessment of infants before vaccinating them. Moreover, many of the pre-administration activities were not carried out correctly, such as vaccine preparation and interacting with clients appropriately. This was evident in the satisfaction survey results, where only nearly four in ten of mothers reported being dissatisfied with the service. These results indicate that the immunization services are not meeting the expected standard of quality, which is hindering efforts to reach optimal immunization coverage.

The uptake of childhood immunization services was found to be suboptimal for all types of vaccines. Nationally, one out of four children did not receive any vaccines at all. In addition, only nearly four in ten

of mothers reported being dissatisfied with the service. These results indicate that the immunization services are not meeting the expected standard of quality, which is hindering efforts to reach optimal immunization coverage.

The uptake of childhood immunization services was found to be suboptimal for all types of vaccines. Nationally, one out of four children did not receive any vaccines at all. In addition, only nearly four in ten children complete the full course of recommended doses. Furthermore, among those who began vaccination services, nearly one in ten and two in ten children dropped out before reaching the pentavalent 3 and MCV 1 stages, respectively. This means that there are significant obstacles in ensuring that the target population is reached for vaccination and that there is also a risk of missing the chance to effectively deliver the service to those who have been in contact. Immunization coverage varies significantly across different regions and social determinants. This highlights the importance of implementing region-specific interventions and approaches tailored to the specific contexts and socio-demographics.

The national maternal vaccination coverage for Td was high, indicating that most mothers have received at least one dose of the Td vaccine. However, almost less than one in ten mothers completed the full dose of Td vaccine. Furthermore, approximately half of adolescent girls received at least one dose of the HPV vaccine, with roughly a quarter of adolescents had received the second dose. This implies that there is a huge gap in accessing the vaccine for the eligible population who are at risk of the cervical cancer. More than half received at least one dose of COVID-19 vaccine. One in four individuals having received both second and third doses of the COVID-19 vaccine.

5.6. Recommendation

Immunization service availability and readiness

- Only six in ten health facilities offered birth, infant, and adult vaccines. Hence, providers, partners and implementers need to give more priority in increasing immunization service availability across health facilities in order to reach all eligible children and adults

- Only nearly four in ten health facilities offered immunization services on a daily bases. Hence, maximizing immunization service availability to offered eligible children and adults received vaccine at the time of their visit in health facilities for immunization should be improved. Priority should be given for regions with their health facilities low performance of offering immunization services on daily bases.

- Nearly one in ten health facilities fulfilled all the required immunization tracer items. This affects the equity, quality and access of immunization services. So, to improve the immunization service antigen and amenity availability proper planning by applying digital supply chain management system is important.

Immunization session quality

- Since there are gaps in properly practicing the vaccination session, regular capacity building, close supportive supervision and enhancing the culture of accountability system is important.

Vaccination service coverage

- Since the coverage of zero dose and drop outs are very high, applying digital microplanning and collecting the geo tagged data of children may help to properly track zero dose children and defaulters.

- Working on vaccine availability and proper client approaching will help to enhance the service uptake of immunization services

- Design digital demand generation and communication system may also help to reach appropriate information for the users and minimize vaccines hesitancies and improve service uptake.

.

ADVERSE EVENTS FOLLOWING IMMUNIZATION (AEFI)

6. Adverse Events Following Immunization (AEFI)

This section presents types of adverse events following immunization among children, adolescent girls and adults, the measures taken by the community for AEFI, AEFI surveillance by the health system, the role of health institutions in AEFI surveillance activities, actions taken by the health system after AEFIs happened.

Although immunization is the most effective and safe intervention to prevent millions of deaths; AEFIs can put a person in life threatening conditions including hospitalization, congenital malformation, and death. Reported adverse events can either be true adverse events (that are resulting from the vaccine or the immunization process itself), or coincidental events that are not due to the vaccine or the immunization. Vaccine recipients concern on AEFI, spread rumors on immunization, and negative perception associated with its adverse effect negatively affects the uptake of immunization.

Key findings

- One in two (51%) vaccinated children; 44% of people vaccinated for COVID-19, and one in five adolescent girls vaccinated for HPV developed at least one type of AEFI
- While one-five (21.2%) AEFI cases sought care from various sources, more than a quarter of them treated it at home
- Only 14 AEFI cases were compensated with cash
- More than 11 children hospitalized because of serious AEFI
- Rural residents have limited awareness vaccine adverse events, and its management
- Children who developed AEFI drop out the subsequent vaccine schedules
- Nearly nine in ten (88%) health facilities did not report a single AEFI case in one year preceding the survey.
- One in six the facilities either had no role in the management of AEFI or they do not know their role
- AEFI surveillance activities are not considered as part of the routine activities at the health posts
- Less than one-third (31.5%) pastoral setting health facilities, 65% of urban setting health facilities, and 68% of agrarian health facilities notify and report AEFI cases
- No health facilities from pastoral areas and 21% of urban facilities conduct casualty assessment for AEFI

6.1 Adverse Event Following Immunization (AEFI)

The AEFI surveillance system is considered to be functional if at least 10 reports of AEFI are reported per 100,000 surviving infants per year according to the WHO standard. This study revealed that about 88% of facilities did not report a single AEFI case in one year preceding the survey. Of those who reported AEFI, only 4% of them reported more than 10 AEFI cases in the same time period. The regional disaagregation showed while about 40% of facilities in Addis Ababa reported one or more cases of AEFI, Dire Dawa and Southwest Ethiopian people reported zero AEFI cases.

About 2,953 mothers/caregivers coming to facilities were interviewed to assess occurrences of AEFI. Majority (56.7%) of the respondents were attending health centers and about one-third (31.36%) were

attending health posts at the time of the survey. The mean age of the mothers/care givers was 27.23 (±5.6) years. Sixty percent of the attendee were from agrarian and 14% from pastoral areas (Table 50).

Overall, a total of 29,602 individuals were approached to assess the occurrence of AEFI. For childhood vaccinations, 16,619 children (13,666 from household survey and 2953 from exit interview), 7647 individuals for COVID-19 vaccine, and 5,336 adolescent girls for HPV vaccine. More than 50% children from exit interview and 60% from household survey developed AEFI. Among adults received COVID vaccine, 44% developed AEFI, and 21% adolescent girl received HPV vaccine also developed AEFI Figure 6.1

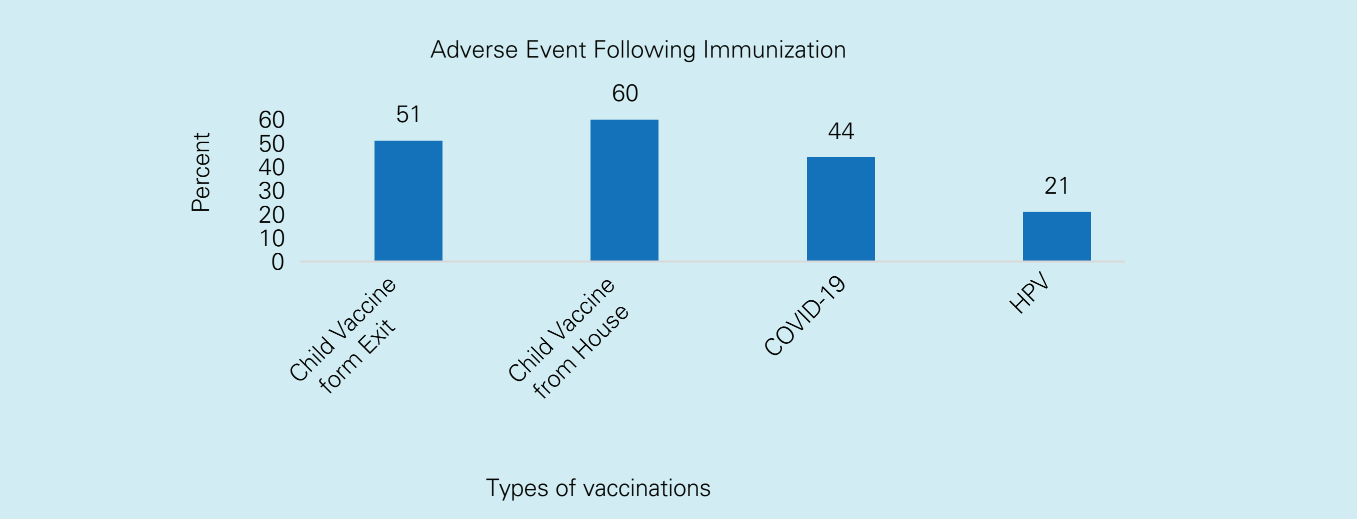

Figure 6.1: Prevalence of adverse effect following immunization based on types of vaccine in Ethiopia, 2013

6.2 Types of Adverse Events Following Immunization

Types of Adverse Event Following Routine Immunization among Children
Majority of the symptoms were non serious adverse events; fever constitute 97.7% and 57.3% of the AEFI among children observed at exit interview, and household survey respectively, followed by fatigue (8.8%) and myalgia(6.4%) Figure 6.2. About 11 children hospitalized, 68 faced life threatening illness, and 9 children permanently disabled because of AEFI.

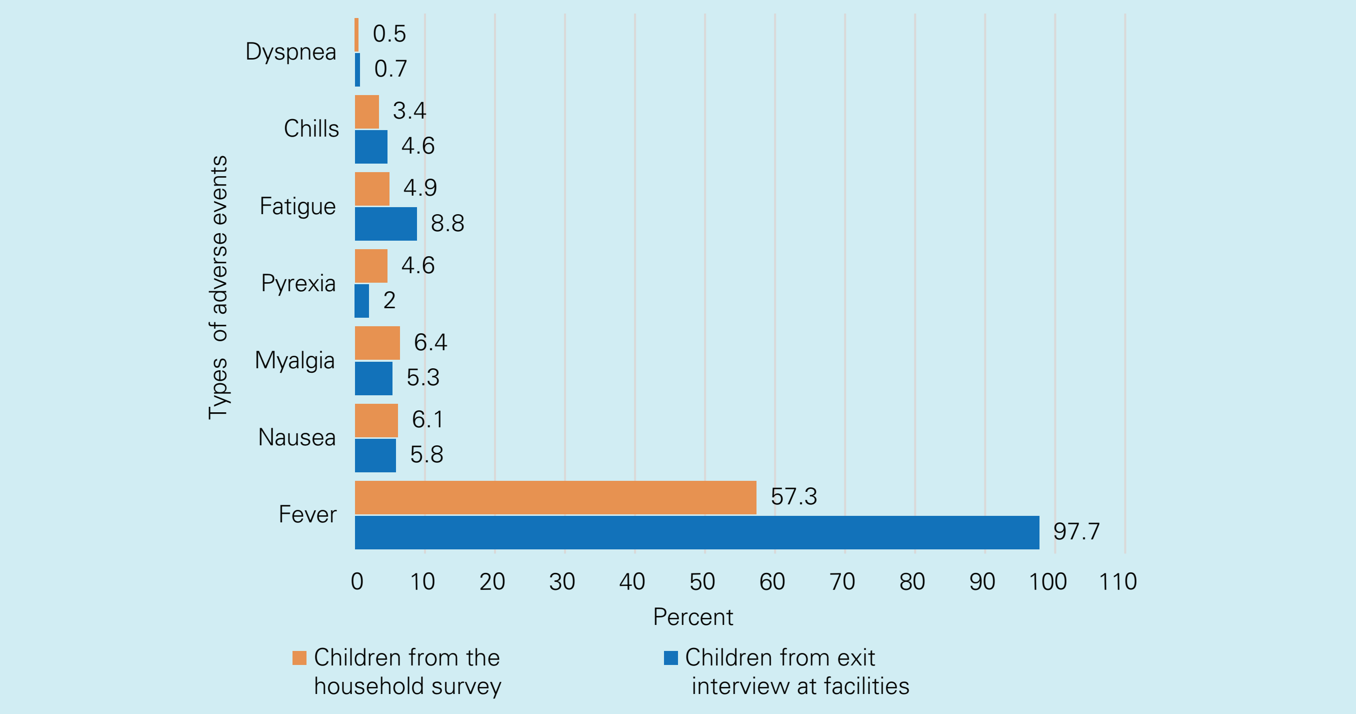

Figure 6.2: Types of adverse events following immunization among children received routine vaccine in Ethiopia, 2023

Qualitative reports from community experience indicated that most of the AEFI cases were mild and subside on their own. Commonly mentioned adverse effects include injection site pain, swelling, fever and nausea. These side effects were mentioned not serious, and perceived as the vaccine effectively working in the body of the child. Across the board, it was indicated that mothers were aware of these potential adverse events which do not result in permanent damages. Disparity of knowledge exhibited between urban and rural, the rural community had limited awareness on vaccine adverse events, what measure should be taken, and its management. In addition to vaccine hesitation, low immunization uptake, particularly in rural settings and loss of confidence on the healthcare workers.

Types of Adverse events following COVID-19 Vaccination

Among individual vaccinated for COVID-19, 44% developed at least one type of AEFI, fever (24%), injection site reaction (22%), tiredness (10%), and muscle pain(10%) were the most common reported adverse events following immunization Figure 6.3. Myocarditis reported by 46, deep vein thrombosis 43, and anaphylactic reaction 18 cases.

The qualitative study pointed out that necessary precautions were being followed during vaccination to prevent any potential complications. An in-depth interview respondent from the federal level indicated that

> *"During COVID 19 vaccination we made Adrenalin mandatory as some people shock when they get vaccinated. We also made vaccination site change because of cluster cases brought during Pfizer vaccination."* (KII, Federal)

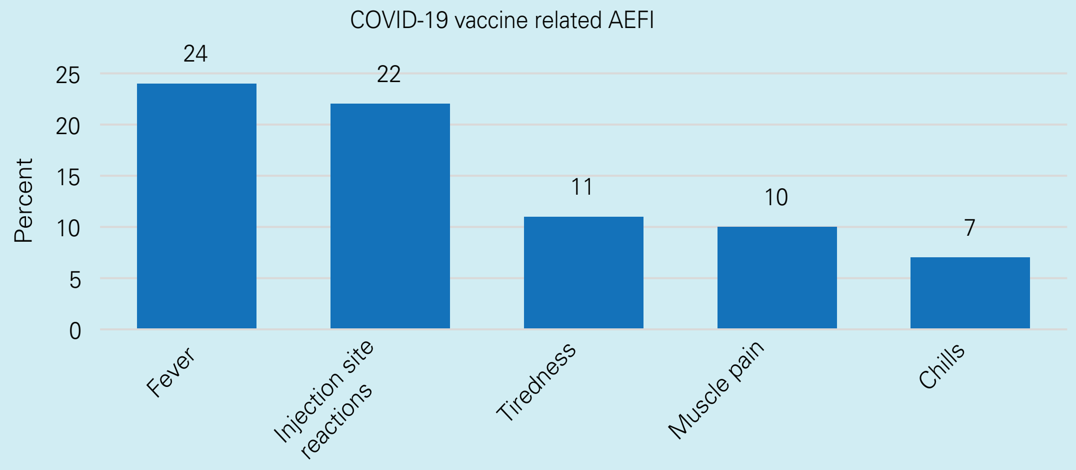

Figure 6.3: Common adverse effect following immunization among adult received COVID vaccine, in Ethiopia, 2023.

Types of Adverse Event Following HPV Vaccination among Adolescent Girls

Among HPV vaccinated 15-18 years old 21% developed some adverse events following immunization, most of it were non-serious such as injection site reaction (22%) head ache (10%), and fever (10%)

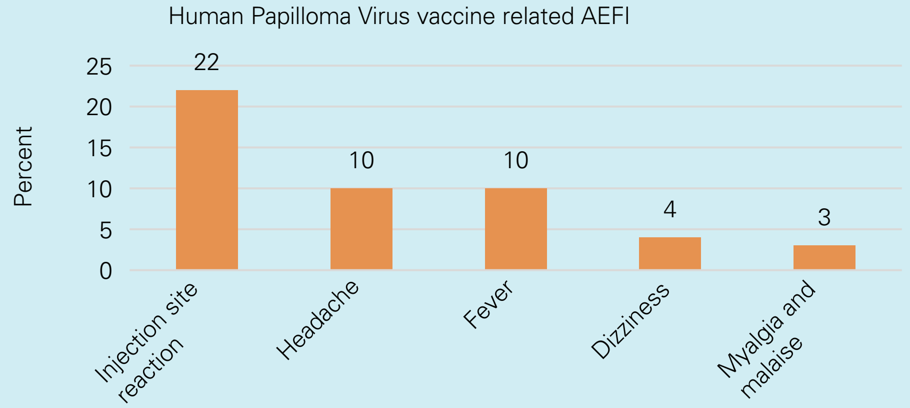

Figure 6.4: Common adverse events following immunization among adolescent girl who received Human Papilloma Virus vaccine in Ethiopia, 2023.

6.3 Measures taken for adverse effect following immunization by the community

Among the total developed AEFI 61% sough some types of treatment. More than half (50.6%) of managed the cases at home, 6.1% treated at outpatient department, 4.4% take anti pain /anti pyretic from pharmacy, 0.5% sought treatment from religious services, 9.1% compensated with cash, and 11.1% compensated with free treatment for the AEFI (Table 6.1). The in-depth interview report indicated that almost all mild AEFI cases were managed at home and only few people visited health posts or facilities, where necessary advice and treatment is provided.

"…there was a challenge in reporting of AEFI following COVID-19 vaccination. Most of the vaccinated individuals were not interested to report adverse events related to the vaccine despite experiencing them and that results in under reporting of AEFIs from the community" (KII, Health worker)

Table 6.1: Measures taken for adverse events following child immunization among children in Ethiopia 2023.

Variables (n=7750)	Response	Frequency	Percent
Have taken some measure	No	3016	38.9
	Yes	4734	61.1
Treated as an outpatient at health facility	No	7273	93.9
	Yes	477	6.1
Treated as an inpatient at health facility	No	7686	99.1
	Yes	64	0.8
Referred to the higher level hospitals	No	7729	99.7
	Yes	21	0.3
Visited traditional healers	No	7701	99.4
	Yes	49	0.6
Taken anti-pain/anti pyretic from the pharmacy	No	7409	95.6
	Yes	341	4.4
Managed at home	No	3828	49.4
	Yes	3922	50.6
Went back to the EPI clinic	No	7612	98.2
	Yes	138	1.8
Religious remedies	No	7714	99.5
	Yes	36	0.5
Compensated in Cash	No	139	90.8
	Yes	14	9.2
Compensated using free medical service	No	136	88.8
	Yes	17	11.1

6.4 Measures taken for adverse events following HPV vaccination

Among 3,387 individuals reported to have at least one adverse event, one-third (33%) of them treated the symptom at home, 7.1% treated at health facility as outpatient, 1.9% treated as inpatient at health facility, and 1.4% sought treatment from traditional healer (Table 6.2).

Table 6.2: Types of measures taken for adverse event following HPV vaccination among 15-18 girls in Ethiopia, 2023.

Actions taken for AEFI	Response	Frequency	Percent
Treated at an outpatient at health facility	No	3146	92.9
	Yes	241	7.1
Treated as an inpatient at health facility	No	3322	98.1
	Yes	65	1.9
Referred for further care to tertiary hospitals	No	3358	99.1
	Yes	29	0.8
Visited traditional healers	No	3338	98.5
	Yes	49	1.4
Taken over the counter anti-pain/anti pyretic drugs	No	3247	95.8
	Yes	140	4.1
Managed at home	No	2267	66.9
	Yes	1120	33.1
Went back to the EPI clinic	No	3355	99.1
	Yes	32	0.9
Religious remedies or practice	No	3330	98.3
	Yes	57	1.7

6.5 Compensations in place for AEFI

Fifty-eight adolescent girl received compensation for adverse event following immunization; 48 of them received free medical service, and 8 of them received cash compensation (Table 6.3).

Table 6.3: Compensation for adverse event following human papilloma various vaccination among 15-18 years old adolescent girls in Ethiopia, 2023

Type of compensation	Frequency	Percent
Cash		
No	50	86.2
Yes	8	13.8
Free medical service		
No	10	17.2
Yes	48	82.8
Others*		
No	54	93.1
Yes	4	6.9

Qualitative report highlighted free medical service provision for AEFI cases. However, absence of clear compensation scheme or policy for AEFI case was one of the barriers for AEFI identification, reporting and surveillance in the sector. Most of the participants mentioned that AEFI compensation policy and mechanism is required for those who suffer from COVID-19 and other vaccinations, despite the efforts made by health facility in supporting the cases.

> *"Compensation is given if it is related with the vaccine, but if the problem happens because of the health professional, it would be a legal issue"* (KII, MoH)

6.6 AEFI cases reported from health facilities

Health facilities are expected to routinely report AEFIs for further investigation and timely intervention. This survey indicated that 88% of facilities have not reported a single AEFI case in one year preceding the survey. Only 4% of them reported more than 10 AEFI cases in one year preceding the survey. While about 40% of facilities in Addis Ababa reported one or more cases of AEFI. Whereas Harari and Southwest Ethiopian people regions reported zero AEFI cases one year preceding the survey. Overall about 90% of the health facilities reported no AEFI cases in Ethiopia (Figure 6.5).

In line with this, the qualitative study also revealed presence of weak detection and reporting of AEFI at facility and community levels, and HEWs have low skills in detection and reporting of AEFI cases. Failing to conduct regular AEFI case detection in routine immunization, and under reporting of AEFI cases among the health workers due to fear of accountability and disrespect from the colleagues was also mentioned by key informants which results in under-reporting of cases.

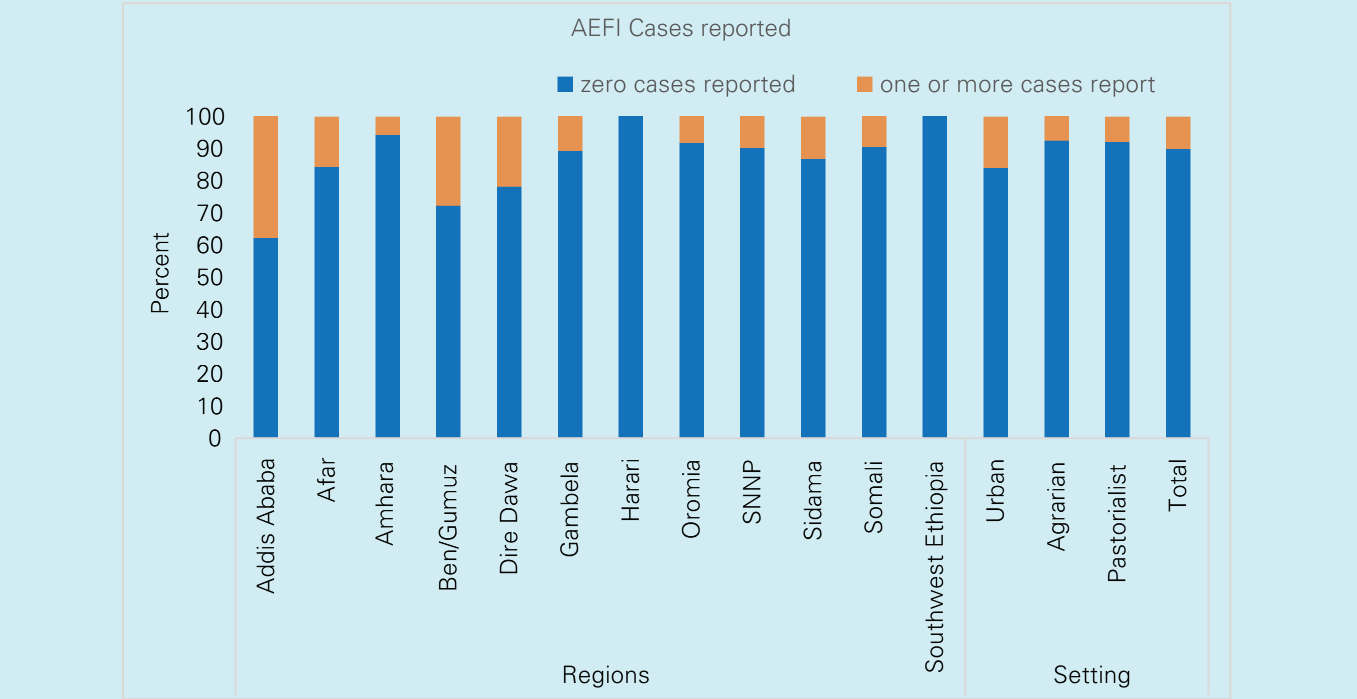

Figure 6.5: Number of AEFI cases reported from health facilities and health posts by regions in Ethiopia, 2023

6.7 Roles of health facilities in investigating and managing AEFI cases

Health facilities providing immunization services reported the facility's role in managing AEFI cases. A higher proportion of facilities (64%) had a role in AEFI reporting and notification followed by prevention and management of AEFI cases (Figure 6.6).

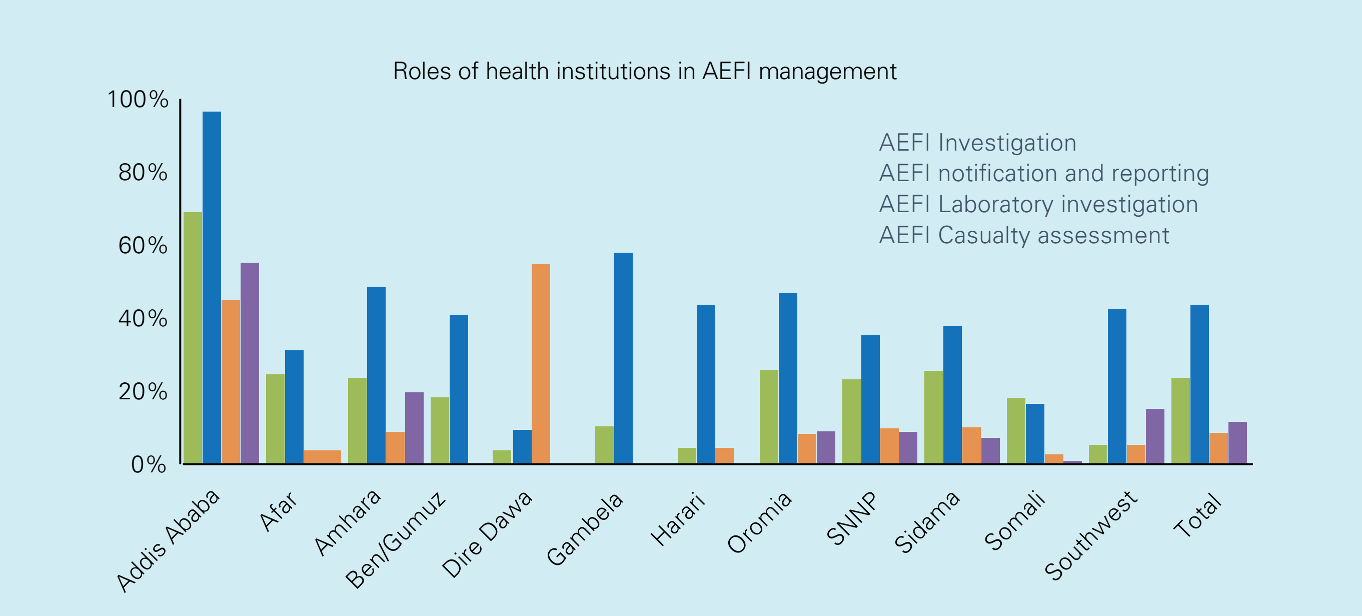

Figure 6.6: Roles of Health Facilities in AEFI investigation and Management by regions of Ethiopia, 2023.

This study further revealed that about 15% of the facilities either have had no role in the management of AEFI or they do not know their role. Looking at the disaggregation by the role of facilities by their location, across the board, facilities from pastoral areas had a lower practice of AEFI management and investigation. For instance, less than one-third of facilities from pastoral locations notify and report AEFI cases when they happen, compared to 65% and 68% in urban and agrarian facilities, respectively (Table 6.4)

Table 6.4: Roles of health facilities in managing and investigating adverse effect following immunization by type and location, in Ethiopia, 2023

Health Facility Role in AEFI Cases	Prevention & management of AEFI	AEFI Investigated	AEFI notification & reporting	AEFI Laboratory Investigation	AEFI Casualty Assessment	Don't know	None	Others
National	243.2 (63.2)	140.3 (36.5)	247.9 (64.1)	58.4(15.2)	71.2(18.5)	24.5(6.4)	37.4(9.7)	7.6(1.9)
Facility type								
Hospital	36.5(51.9)	23.0(32.1)	39.4(54.9)	9.99(13.9)	16.2(22.6)	4.91(6.8)	12.2(17.0)	1.0(1.4)
Health center	206(66.0)	117(37.4)	208(66.5)	48(15.4)	55(17.5)	19.5(6.2)	25.2(8.0)	6.9(2.2)
Health post	37.2(58.3)	21.8(34.1)	48.57(76.0)	NA	7.1(11.1)	(0)	(0)	4.2(6.6)
Location								
Urban	140.1(62.5)	80.2(35.7)	145.9(65.1)	37.95(16.9)	47.4(21.1)	12.5(5.5)	25.3(11.2)	6.5(2.9)
Agrarian	92.7 (66.1)	53.1(37.7)	95.4 (67.9)	19.77 (14.0)	23.8(17.0)	10.6(7.5)	7.4(5.2)	1.4(1.0)
Pastoral	10.4(49.9)	7.1(34.3)	6.5 (31.4)	0.69 (3.3)	0.0 (0.00)	1.3 (6.2)	4.7(22.7)	0.0(0.0)

Consistent with these findings, the qualitative study revealed that there was skill gap in detection and reporting of AEFI at the health posts. It was also pointed out that HEWs at health posts are overburdened with other workloads and have low motivation to follow children for AEFIs in the community. Similarly, a weak AEFI detection and reporting was reported at private facilities. Lack of focal person for the immunization provision and high turnover made the detection and reporting inconsistent at these facilities. On the other hand, AEFI surveillance activities are not considered as part of the routine activities of the health posts. Respondent mentioned that

> *"We don't do AEFI surveillance on a regular basis. When we have enough time or less workload we conduct house to house surveillance, to be honest, we don't consider it as a top priority. However, we advise the community to bring any AEFI cases back to us, including leg weakening and/or flush (red rash) following vaccination"* (KII, HEW)

6.8 Pharmacovigilance characteristics

In the assessment of availability of Pharmacovigilance centers, about 16% of the health facilities have a Pharmacovigilance center, with only 20% of these center having a regular budget provision and in 17% of the facilities there was inadequate skilled human resources for the center (Table 6.5)

Table 6.5: Pharmacovigilance Center Characteristics by type and location of health facilities in Ethiopia, 2023

	Pharmacovigilance Center Availability		Regular Budget for Pharmacovigilance		Human Resource for the Pharmacovigilance center	
	Yes	No	Yes	No	Yes	No
National	60.6(15.7)	324.1(84.2)	19.5 (32.2)	41.15(67.8)	50.3(82.9)	10.38(17.1)
Facility type						
Public Hospital	12.4 (17.2)	59.3 (82.8)	4.6(6.4)	67.1(93.54)	10.2(14.2)	61.5(85.7)
Health centers	48.3 (15.4)	265.1(84.6)	14.8(4.8)	298.6(95.2)	40.1(12.78)	273.4(87.2)
Location						
Urban	42.7 (19.0)	181.3(80.9)	15.6(7.0)	208.3(93.0)	29.4(13.1)	194.6(86.9)
Agrarian	14.7 (10.5)	125.6 (89.5)	3.8(2.7)	136.5(97.2)	18.6(13.3)	121.7(86.7)
Pastoral	3.2(15.5)	17.53 (84.5)	0.00 (0.0)	20.7(100.0)	2.22 (10.7)	18.54 (89.3)

The regional disaggregation indicated that about three out of four health facilities (72.3%) from Addis Ababa have a pharmacovigilance center with half of these centers having regular budget (Figure 6.7) In Somali region, almost all (98%) do not have a pharmacovigilance center. Similarly, in most regions, a significant majority of the centers do not have a regular budget and adequate human resources.

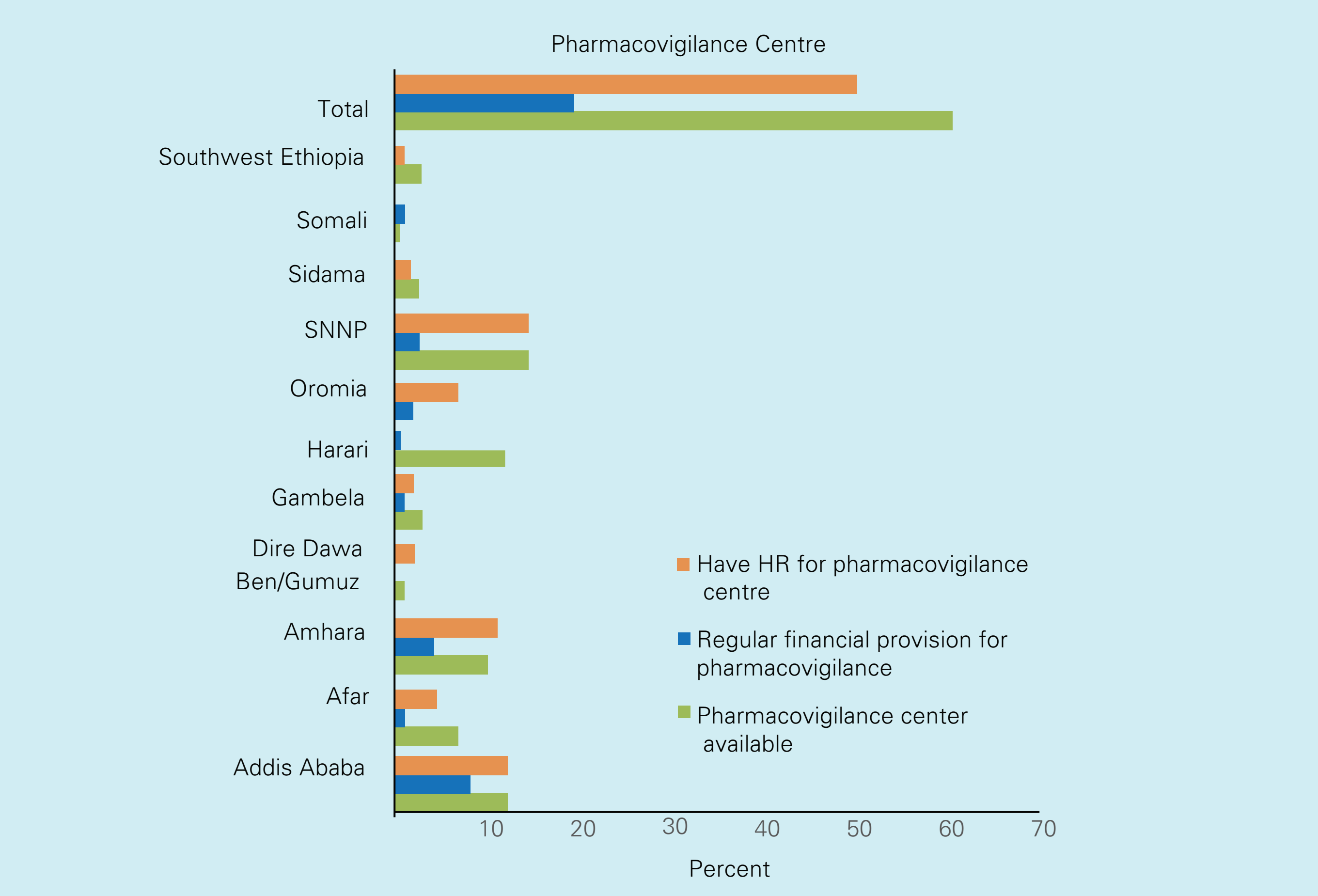

Figure 6.7: Availability of pharmacovigilance center and resource availability among health facilities in Ethiopia, 2023.

6.9 Availability of resources and inputs for AEFI surveillance and management

Overall, more than half of the facilities (52.1%) do not have AEFI investigation forms whereas more than three-quarters (77.2%) of them lack laboratory request forms. Even though all health facilities have mandate to provide vaccination, there is a huge variation on availability of resource to among level of health facilities, the primary health care facility have minimal contribution on AEFI management, though they delivery substantial immunization service (Table 6.6 and Table 6.7).

Table 6.6: Availability of inputs needed for adverse event following immunization surveillance and Management in Ethiopia, 2023.

	AEFI investigation form availability		Availability of Case definition for reportable AEFI cases		AEFI reporting form availability		AEFI laboratory request form availability	
	Yes	No	Yes	No	Yes	No	Yes	No
National	182.8(47.5)	201.9(52.5)	165.9(43.1)	218.9(56.9)	277.0(42.2)	378.4(57.7)	85.3(22.1)	299.4(77.8)
Facility type								
Public Hospital	31.5 (43.9)	40.2 (56.1)	35.31 (49.2)	36.4 (50.7)	36.4 (50.7)	35.3 (49.2)	20.2(28.2)	51.5 (71.8)
Health centers	151.2 (48.2)	162.2(51.74)	130.6 (41.6)	182.8 (58.3)	192.1 (61.3)	121.4(38.7)	65.1 (20.7)	248.4 (79.2)
Health post	27.1(10.0)	242.7(89.9)	19.4(7.2)	250.3(92.8)	48.1(17.82)	221.7(82.2)	7.1(2.6)	262.4(97.3)
Note: AEFI = Adverse events following Immunization								

Table 6.7: Availability of inputs needed for AEFI surveillance and Management in Ethiopia, 2023.

	Guideline for surveillance and respons of AEFI cases		Worksheet for causalit assessment		SOP for AEFI detectio availability		Process is in place for data recording and analysis of AEFI	
	Yes	No	Yes	No	Yes	No	Yes	No
National	145.7(37.8)	239.0(62.1)	68.55(17.8)	316.2(82.2)	79.93(20.8)	304.8(79.2)	117.2(30. 4)	267.5(69.5)
Facility type								
Public Hospital	32.7(45.7)	38.9(54.3)	12.8(17.9)	58.9(82.1)	12.5(17.5)	59.1(82.5)	22.2(30.9)	49.5(69.0)
Health centers	112.9(36.[illegible]	200.5(63.[illegible]	55.7(17.8)	257.7(82.[illegible]	67.3(21.4)	246.1(78.[illegible]	95.0(30.3)	218.4(69.[illegible]
Health post	17.2(6.4)	252.5(93.[illegible]	7.9(2.9)	261.8(97.[illegible]	5.0(1.8)	264.7(98.1	NA	NA
Locatior								
Urban	91.3(40.7)	132.7(59.2	45.24(20.2	178.8(79.[illegible]	49.1(21.9)	175.0(78.1	75.2(33.5)	148.8(66.4
Agrarian	48.2(34.3)	92.2(65.7)	19.6(13.9)	120.7(86.[illegible]	26.8(19.0)	113.6(80.[illegible]	37.9(27.0)	102.4(72.[illegible]
Pastoral	6.31(30.4)	14.4(69.6)	3.6(17.8)	17.1(82.2)	4.0(19.7)	16.7(80.3)	4.1(19.6)	16.6(80.3)
Note: AEFI = Adverse events following Immunization;								

6.10 Regulatory Actions taken by the pharmacovigilance centers

Causality assessment is the systematic evaluation of the information obtained about an AEFI to determine the likelihood that the event might have been caused by the vaccine/s received. Causality assessment does not necessarily establish whether or not a definite relationship exists, but generally ascertains a degree of association between the reported adverse events and the vaccine/vaccination. Nevertheless, causality assessment is a critical part of AEFI monitoring and enhances confidence in the national immunization program and regulation of the safety and quality of the product. Vaccine recipients want to know whether what they have experienced was due to the vaccine.

Responding to AEFI may involve immediate short-term activities or/and long-term follow-up activities. Follow-up activities should be based on findings of investigations, causality assessments and recommendations by the investigation team/AEFI committee at different levels of the health system.

Majority (91%) of health facilities reported to have not done anything when AEFI happen at their facilities. On the other hand, about 5% of them reported withdrawing the vaccine and 7.8% of them reported to put safety warnings on the vaccine (Table 6.8).

Table 6.8: Regulatory Actions taken by the pharmacovigilance center by type of facilities and region in Ethiopia, 2023.

	Product label change	Safety warnings on the vaccine	Withdrawal of vaccine	Restriction on vaccine use	None	I don't know
National	24.7(6.4)	30.1(7.81)	18.9(4.9)	20.3(20.3)	267.9(69.6)	81.3(21.1)
Facility type						
Public Hospital	0.0	0.9(1.2)	0.00	0.00	53.2(74.2)	17.6(24.5)
Health centers	24.7(7.8)	29.1(9.3)	18.9(6.1)	20.3(6.4)	214.7(68.5)	64.1(20.4)
Health posts	5.9(2.2)	11.1(4.1)	13.1(4.8)	5.2(1.9)	159.8(59.2)	95.4(35.3)
Location						
Urban	14.7(6.5)	16.8(7.5)	12.9(5.7)	17.30(7.7)	144.2(64.3)	55.5(24.7)
Agrarian	7.9(5.6)	11.2(7.9)	5.4(3.8)	1.18(0.8)	111.6(79.5)	21.5(15.3)
Pastoral	2.0(9.9)	1.9(9.5)	0.6(2.9)	1.81(8.7)	12.1(58.3)	4.7(22.9)

AEFI = Adverse events following Immunization

The AEFI surveillance system generated information are used for early detection and analysis of AEFI. In Ethiopia, the practice of managing and monitoring AEFI has been strengthened particularly after Covid-19 vaccination. Qualitative report underline the importance of AEFI, it stated as:

> *"During COVID19 vaccination, we made Adrenalin mandatory as some people collapse when they get vaccinated. We also made vaccination site change because of cluster cases brought during Pfizer vaccination."* (KII, Federal).

In most regions, AEFI surveillance is being conducted mostly during vaccination campaigns. No adverse events investigation and reporting is regularly conducted during the routine immunization, except few reports following COVID19 vaccination that improved AEFI reporting after the pandemic. There was also

low integration for AEFI monitoring and reporting at low levels in the health system including failure to joint planning and performance review. Absence of AEFI compensation policy, competency gap of health extension workers was reported to have negatively impacted the management of adverse events.

Finding from qualitative report stated as follows:

> *"….I administered the vaccine on the right side of the hand, and on both the right and left sides of the child's legs. Then, both sides the child's legs became swollen and the family of the children got into conflict with me. They said she is killing our child and I had to advise them about side effects of vaccine with one of my co-worker who has more experience"* (KII, Health extension workers)

The regional disaggregation of measures taken after AEFI indicated that most regions have not taken any regulatory actions for AEFI related problems. Withdrawal of the vaccines that were associated with the AEFI was amongst the commonest intervention followed by putting safety warnings and restricting the use of the vaccines. Figure 6.8 depicts the actions taken disaggregated by region.

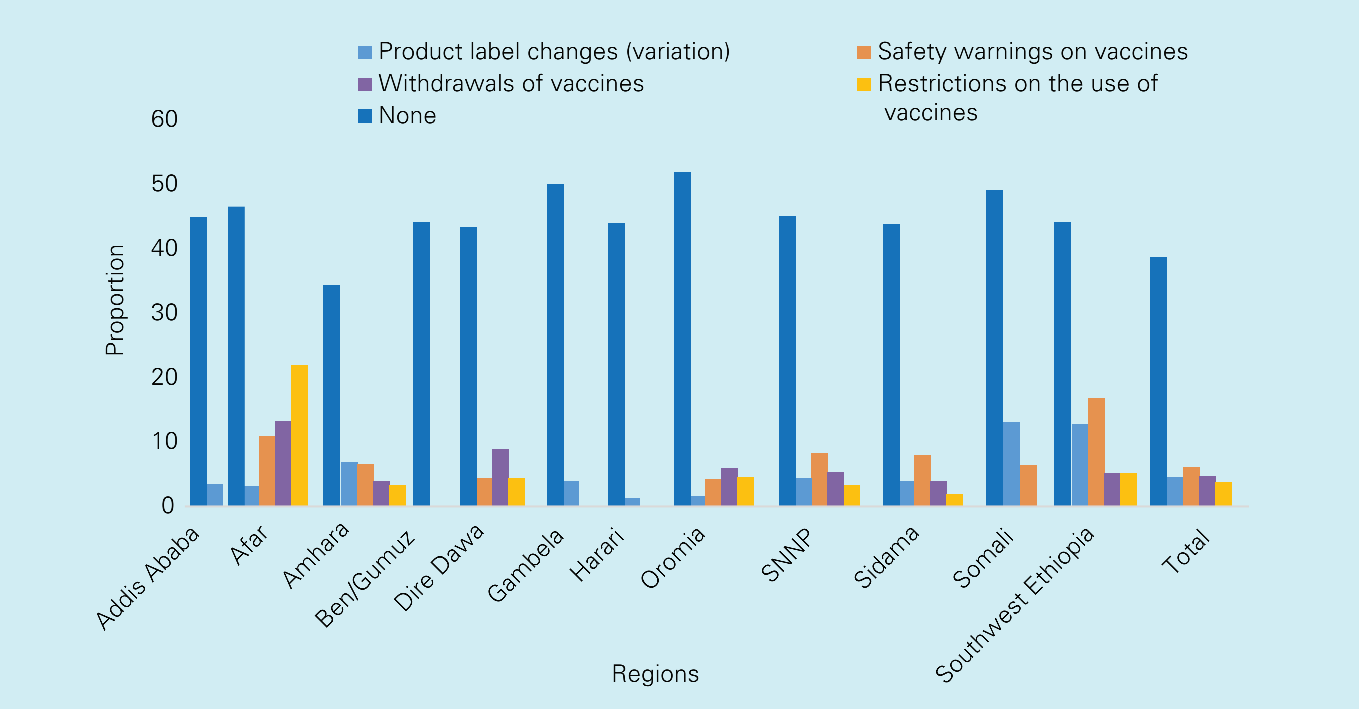

Figure 6.8: Regulatory Actions taken by health facilities for AEFI by regions of Ethiopia, 2023.

6.11 AEFI Surveillance System challenges

Despite the rapid expansion of immunization program in its scope and type of antigens provided in the country, AEFI surveillance system has faced quite several challenges. In this study, the challenges have been categorized thematically into structural, technical, and behavioral. Lack of adequate skilled human resource, high turnover of EPI focal persons, health workers skill gap in detection and reporting of AEFI at health posts, fear of reporting and accountability among health workers, workload at health posts, and low motivation of HEWs were among the major reported challenges.

Structural challenges

Ambiguity in roles and line of communication, absence of AEFI taskforce at zonal and woreda, weak coordination between stakeholders, workload at health posts, turnover of EPI focal, AEFI surveillance not integrated with HMIS, absence AEFI treatment compensation policy, sub-optimal engagement of private sectors, lack of report from private facilities, infrastructure related problems such as electric power and refrigerators. Such health posts collect vaccines from nearby health centers during vaccination dates. The respondent said that

> *"…the immunization program provided by mobile sessions is work that is done overnight for two or three days, so even at this time, we have to worry about the safety and quality of vaccination"* (KII, ZHD)

Most respondent stated that AEFI are not being conducted, though it is one of the major activities in the sector.

Technical challenges

Low reporting of AEFIs during routine vaccinations, data quality problem, absence of regular feedback, none-functional AEFI task force at regional level, weak detection and reporting at facility and community, absence of regular maintenance of faulty refrigerators were identified technical challenges.

Behavioral challenges

Fear of reporting and accountability by health workers, low staff motivation, skills gap in detection and reporting of AEFI at health post, low motivation of HEWs, low awareness in the community, negative perception and rumors, knowledge and skill gap in cold chain management among EPI service providers were among the major behavioral challenges reported. Table 6.10 shows the AEFI surveillance related structural technical and human resources related problems at all levels of the health care system, including at the community level.

Table 6.9: Summary of structural, technical and behavioral related problems of AEFI surveillance in Ethiopia 2023.

levels	Identified Challenges		
	Structural	Technical	Behavioral
Federal	• Ambiguity of roles between stakeholders working on AEFI<br>• Absence of clear compensation policy	• Safety advisory committee not mee regularly<br>• Feedback is not regularly send to facilities<br>• AEFI data accuracy and completeness<br>• Lack continuous support and monitoring,<br>• No integration of AEFI activities with other routine onsite and outreach services	• Low human resource across all levels,
Regional	• AEFI task force not fully functioning<br>• Ambiguity of the roles of EFDA, EPHI and EPI Unit<br>• Lack of human resources	• Under reporting of AEFIs<br>• Weak monitoring and follow up at lower levels due to workload<br>• Underreporting of minor AEFIs<br>• Poor AEFI reporting system<br>• No follow up of vaccinated person after routine immunization; mostly focused on vaccination campaigns<br>• No AEFI detection and report during routine immunization<br>• Low case treatmet and advise at facility level<br>• Workload to give support at lower level	• Inadequate awareness among health professionals on AEFI<br>• Inadequate counseling of mothers about AEFI after EPI service<br>• Feeling of ashamed and fear of disrespect among health professionals
Zonal	• No structure or AEFI task force at zonal and down the structure	• Gaps in reporting mechanism at zonal and woreda level following campaigns,	• No skilled EPI human resource
Woreda	• No structure or focal person and AEFI task force	• Inadequate AEFI monitoring during routine immunization	• Inadequate skilled human resource

Woreda	• Minor AEFI cases not reported to woreda, while severe case reporting to both woreda and region	• Focus only given to AEFI monitoring during vaccination campaigns overlooking routine sessions<br>• Poor AEFI reporting and underreporting of AEFI due to lack of awareness	• Identification of AEFI in the community done by HEWs only
Health facilities (Hosp, HC, clinic)	• In all regions, EFDA does not have an officer at the facility level<br>• low engagement of private sectors<br>• No budget for AEFI treatment	• Lack of AEFI report fro m private facilities providing vaccination services<br>• Suboptimal AEFI detection and notification at private facilities, except few clinics<br>• Sub optimal counseling to mothers of the child	• Less emphasis given for AEFI by EPI focal person<br>• FDA has no has no structure office<br>• Low motivation<br>• High turnover of focal person working EPI<br>• gaps in skills
Health post	• No dedicated person for AEFI monitoring and management- all the activities are assigned for the HEW	• Low skills of the health workforce in detection of cases<br>• AEFI detection poorly conducted in the community<br>• Weak work relationship between HEWs and community platforms such as WDA/HDA in detection and reporting of AEFI	• Workload of the HEW to adequately monitor and report AEFI<br>• Lack of motivation by the HEW to conduct the task<br>• Skill gap in detection, counseling and reporting of AEFI
Community	• Absence of volunteer health workers in the community	• Poor awareness of the community about AEFI<br>• Rumors, negative perceptions, and unfavorable attitude towards AEFI	• Low AEFI awareness in the community

AEFI surveillance challenges across different levels

National level

The EFDA is a federal regulatory body to monitor both vaccine and drug safety and efficacy in Ethiopia. The authority works in collaboration with MOH, partners and stakeholders to monitoring vaccine related issues. Inadequate reporting of AEFI cases, AEFI reporting channel, absence of AEFI data in some

health facilities, weak adverse event management system in the sector, low stakeholders engagement in vaccine safety monitoring and management, and weak advocacy and awareness creation on AEFI in the society.

The AEFI surveillance system generated information is used for early detection and analysis of AEFI. Respondent mentioned that

> "… now we are communicating with the manufacturers with the case of measles vaccine as asthmatic children are getting in to serious adverse effects including death when they get vaccinated. We are asking them to put "prohibited to Ethiopian asthmatic children" (KII, EFDA)

Subnational level

In most regions, there was no adverse event investigation and reporting during routine immunization, except few reports following COVID -19 vaccination. However, there was some improvement of ARFI reporting after COVID 19 pandemic. There was low integration at low levels for instance in joint planning, reviewing performance. The other challenge was there was no AEFI compensation policy.

> *"In our city, the administration health office manages and monitors Adverse Effects Following Immunization (AEFI) through several practices. Five key messages are posted at every health facility, which informs mothers about the signs and symptoms to look out for after their children receive immunizations, these signs include….."* (KII, ZHD)

AEFI activities was challenged with structural gaps, such as lack of structure at zonal and lower levels, lack of integration with the HMIS, unclear reporting channel, and ambiguity in program ownership. Other constraints include under reporting due to low detection and reporting in the facility level and weak AFEI monitoring system, including health post and in the community. Additionally, there is no compensation system for AEFI, though health facilities provide free medication services. The roles and responsibilities of the partners are not clear. There are causality analysis teams at central and regional levels. However, the expert teams are not routinely working the job due to weak AEFI surveillance system.

AEFI investigation and reporting is limited at the lower levels in the health system, where professionals are overloaded with multiple activities. AEFI reporting system is suboptimal due to inadequate awareness among health professionals and the community, though improvements have been observed after the introduction of COVID-19 vaccine. Some health professionals do not report AEFI cases due to fear of accusation and disrespect from other health professionals.

> *"Previous AEFI is not concerned as an activity done in the facility but now after the occurrence of COVID-19, the region directed health facilities to take AEFI monitoring as a routine activities."* (KII, RHB)

The introduction of COVID-19 vaccine strengthened the AEFI monitoring and response with immediate reporting if any adverse reaction is observed. However, routine immunization does not have follow up to identify and address adverse effects following immunization.

Health facilities use AEFI recording format to register any vaccine related adverse effect. Despite training of the health professionals, zones have not received adverse event report from woreda and facilities, except COVID-19 related reports.

> *"Regarding routine immunization program, there is no actively implemented monitoring and management of AEFI. During the routine vaccination, the health care providers provide health information on probability of developing AEFI and supportive management the mother/care givers of the vaccinated child can undertake at home. But during COVID-19 and mass/campaign immunization program there are active monitoring and management of AEFI"* (KII, Woreda).

It was difficult to trace adverse events in the pastoralist and remote areas. Thus, there could be under reporting of events. Lack of incentive for AEFI surveillance also mentioned as one of the constraint for the program.

> *"We provide the health service for dispersed population, where we walk long distance on foot. As a result, we do have low motivation to perform post-immunization surveillance. Additionally, due to the workload, it is impossible for me to do the AEFI surveillance in the community"* (KII, HEW)

Health facility level

Additionally, absence of AEFI format, compensation or incentive scheme for AEFI surveillance activities was mentioned as a challenge for AEFI surveillance.

One of the HEWs mention that

> *"… we have faced a problem during COVID-19 vaccination campaign: one person comes to us by complaining severe pain, swelling of hand at injection site, my hand is not working following vaccination."*

It was reported that in some health posts AEFI reporting format was not available. AEFI surveillance activities are not considered as part of the routine activities of the health post.

> *"We don't do AEFI surveillance on a regular basis. When we have enough time or less workload we conduct house to house surveillance, to be honest, we don't consider it as a top priority. However, we advise the community to bring any AEFI cases , including leg weakening and/or flush (red rash) following vaccination"*

> *"I had challenges when I was employed in 2007 EC. When I become a new employee, it is obvious that what you have learned and what you practically see are different. At that time we had given vaccines to the community such as BCG on the right arm within 45 days of birth. We traveled to one mother home with 45 days old baby to give vaccines to her child, and I gave them on the right side of the hand and on both the right and left sides of her child's legs. Then both sides of her child's legs became swollen. The family tried to manage the swollen legs, but the swelling had been exacerbated. I went to give polio vaccine to one of the village and they come here to look for me. When I return, the family of the children gets into conflict with me. They said she is killing our child and we fight. Then, I advised her about the vaccine and its side effects with one of the nurse staff members who has more experience than me. I told them not to touch the swollen legs with their hand."* (KII, HEW)

Community level

Though there is general awareness in the community, some community members have negative attitude towards vaccine and its adverse effect. As a result, mothers hesitate to vaccinate their children, particularly in the rural areas. Additionally, there was low awareness in the community about vaccine adverse event management. As a result, some people prefer not to be vaccinated or prefer to stay at home during routine immunization and campaign. In the community, adverse event related rumors and negative perceptions were among the barriers for vaccination uptake in the community. Mothers concerned why children body temperature is raising and got some minor AEFI following vaccination. It is one of the reasons not to take the subsequent doses. Thus, it could be one of the reasons for dropout rates of some antigens. In the rural community, there is a limited awareness of AEFI, though most of the reported AEFI were minor and subside on their own. However, it was mentioned that people living in urban or sub-urban areas have better awareness on the relevance of vaccination in general and adverse effect following immunization and its management.

> *"Mothers use hot water on plastic bottle (highland) to rub on the swelling to disperse the accumulated blood. The children will sleep well and the mother will not miss their sleep. The community now knows about these Minor side effects. Those mothers who used to avoid to vaccinate their children, says they will take the next doses. Vaccines are 100% medicine to children. Children who did not receive vaccines are*

100% medicine to children. Children who did not receive vaccines are infected with different diseases. All mothers know now this." (FGD)

6.12. Conclusions

More than 50% of vaccine recipients developed AEFI, however, 21% them sought care, of which majority of them managed the case at home. This finding underscore that immunization creates some adverse event on huge number of recipients, and few of them sought care. Unless put in place a strong and functional AEFI surveillance system, proactively counsel recipients and promptly manage the event its repercussion damage national EPI program.

Identification, reporting and managing AEFI case is a blurred practice among region, setting, types of health facilities. Health care workers fear to report AEFI for it may associated with accountability and blame. In the same hand, compensating AEFI cases with cash and free medical service had no clear scheme and rarely implemented (only 14 individual has composted in cash). Lack of clear direction pave a way to inconsistent practice and intervention among health facilities and health care works.

Nine in ten facilities not reported a single AEFI case in one year preceding the survey. The functionalities health facilities inequitably distributed among region, only 4% of them reported more than 10 AEFI cases in one year preceding the survey. While about 40% of facilities in Addis Ababa reported one or more cases of AEFI. Whereas Harari and Southwest Ethiopian people regions reported zero AEFI cases one year preceding the survey. Non functionalities of health facilities implies lack of coordination and monitoring, which need health programmer attention.

Pharmacovigilance center was found among 16% of the health facilities of which only 20% have a regular budget. About half of the facilities (52.1%) had no AEFI investigation forms.

Without having key center, budge and human resource it is less likely to achieve national and global target of Immunization. Despite the rapid expansion of immunization program in its scope and type of antigens provided in the country, AEFI surveillance system has faced quite several structural, technical and human resource related challenges.

6.13. Recommendations and Implications

The following recommendation are forwarded to improve AEFI surveillance and management in the country.

- Provision of frequent and targeted training for health workers to ensure vaccine safety in the point of vaccination service provision - focusing on AEFI case detection, data collection, notification and response.
- Regular and continuous supervision and mentorship of EPI service provision at lower levels to ensure proper vaccine storage – including ensuring the supply chain management and vaccine administration
- Capacity building training for EPI unite health workers on refrigerator maintenance, including training of biomedical engineers
- Strengthening collaboration and support of stakeholders at lower levels, including at community level.
- Creation of a structural system at all levels, including zonal, woreda, and health facilities to enhance the integration and coordination of AEFI surveillance
- Integration of AEFI surveillance activities with other routine health

- Integration of AEFI surveillance activities with other routine health service provision and reporting, including with HMIS/DHIS2 reporting system, which facilitate data flow and feedback.

- Establish AEFI surveillance system in the private facilities for timely detection, reporting and response.

- Advocacy and awareness creation about AEFI at community level using influential people including religios leaders and HDAs.

- Training of HEWs, HDAs and volunteers to strengthen community-based AEFI detection and reporting.

VACCINE PREVENTABLE DISEASE (VPD) SURVILLEINCE

7. Vaccine Preventable Disease (VPD) Surveillance

This section presents selected case-based surveillance data for measles, rubella and NNT, human resources and governance for VPD surveillance, surveillance data timeliness and completeness, reporting frequency of the surveillance data, and its challenges.

Vaccine-preventable diseases surveillance forms part of wider infectious and non-infectious public health surveillance – the continuous and systematic collection, analysis and interpretation of health-related data needed for the planning, implementation and evaluation of public health practice. Surveillance for VPDs provides critical, long-term data for timely detection of and response to VPDs to guide optimal use of vaccines and other disease control measures.

In Ethiopia, VPD surveillance is implemented within the framework for the Integrated Disease Surveillance and Response (IDSR) system. The IDSR was adopted as part of Public Health Emergency Management (PHEM), and VPD surveillance became a component of the PHEM core process within the Ethiopian Public Health Institute (EPHI). Currently, Ethiopia has prioritized 32 diseases for surveillance including VPDs such as Measles, Polio, Cholera, Meningitis, and Yellow fever. Indicator based surveillance starts from health posts reported in a weekly basis to the next higher level up to EPHI.

The information generated from the VPDs surveillance system is being used for early detection of outbreaks and for a timely response to detect outbreaks. It is further used for immunization related policy and strategy design including Routine Immunization Improvement Plan (RIIP), PIRI and catch-up vaccinations, preventive campaigns, and reactive vaccinations.

Key findings
• Only One-in-Three facilities (35%) conducted VPD surveillance activities
• One - in - four health facilities and half of the health posts do not have a focal person to conduct VPD surveillance activities
• Only 56% of the health centers & hospitals, and 43% of the health posts reported VPDs immediately
• Inadequate skilled human resource at lower level and shortage of necessary inputs such as lab equipment and capacity building for health workers were the major challenges.

7.1 Case-based surveillance system

Measles Surveillance

The measles case-based surveillance system has been established across the entire country to actively identify and report suspected cases of measles in a timely manner. This early detection is vital for preventing the spread of the virus and containing outbreaks before they cause serious harm. This study assessed the number of measles cases detected through routine surveillance at health facilities across

regions in one year preceding the survey. The findings revealed most cases were concentrated in Eastern, Southern and Northwestern Ethiopia. A large number of cases (>30 cases per district) cases were reported from Somali region, the southwestern and southeastern parts of Amhara, Southern Oromia, Dire Dawa and Harari regions Figure 7.1.

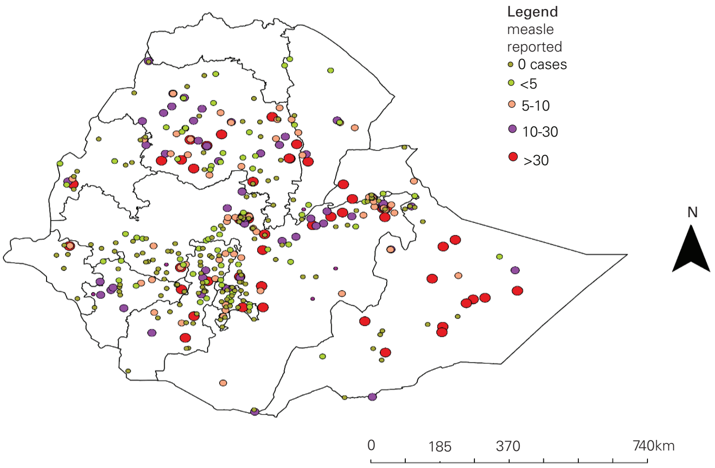

Figure 7.1: Measles cases reported by health facilities in the 12 months preceding the survey in Ethiopia, 2023.

Rubella Surveillance

The measles surveillance platform is also used to identify rubella cases and as a result of the intensive case-based surveillance for measles, rubella surveillance is also being conducted which includes laboratory testing for detection of rubella-specific IgM antibodies. In this study, the spatial distribution of rubella cases 12 months preceding the survey indicated that most of cases where reported from urban setups including Harari, Assosa zone of Gambella regions, Addis Ababa and South Wollo (Dessie Zuria) Figure 7.2.

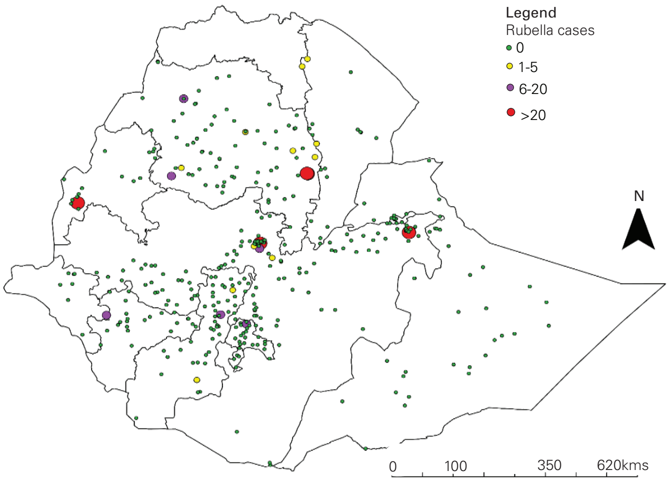

Figure 7.2: Reported rubella cases in the studied facilities 12 months preceding the survey in Ethiopia 2023.

Neonatal Tetanus Surveillance

Neonatal Tetanus (NNT) is one of the immediately reportable diseases under the national PHEM system. NNT surveillance is integrated with AFP and measles case-based surveillance activities, such as capacity building and sensitization activities for HRHs. Despite all the efforts to tackle the problem, NNT continues

Neonatal Tetanus Surveillance

Neonatal Tetanus (NNT) is one of the immediately reportable diseases under the national PHEM system. NNT surveillance is integrated with AFP and measles case-based surveillance activities, such as capacity building and sensitization activities for HRHs. Despite all the efforts to tackle the problem, NNT continues to be reported from different parts of the country. The present study revealed that more than 5 NNT cases per district has been reported from some parts of Somali, Afar and Gambela regions Figure 7.3.

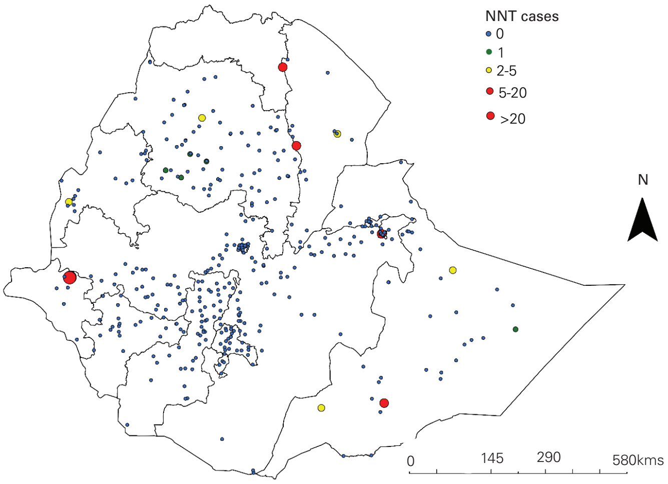

Figure 7.3: Neonatal cases reported by studied health facilities in the 12 months preceding the survey, 2023.

Neonatal Tetanus Surveillance

Neonatal Tetanus (NNT) is one of the immediately reportable diseases under the national PHEM system. NNT surveillance is integrated with AFP and measles case-based surveillance activities, such as capacity building and sensitization activities for HRHs. Despite all the efforts to tackle the problem, NNT continues

7.2. Human Resource and Governance of VPD Surveillance

More than one in four health facilities (27.1%) do not have a focal person to conduct VPD surveillance activities with significant variation among health posts whereby more than half (53.7%) not having assigned VPD focal person. In Ethiopia, VPD surveillance activities at health facilities are expected to be conducted by the public health emergency department and this study indicated the responsible unit in about three-quarters of facilities (75.3%) was the PHEM unit with around one-third (35%) reporting the EPI unit conducts the surveillance activities Table 7.1.

Table 7.1: Human Resource and Governance of VPD surveillance by type of facilities and location in Ethiopia, 2023

	Focal person for VPD surveillance		Responsible unit for conducting VPD surveillance at the facility			
	Yes	No	EPI unit	PHEM unit	IMNCI	Others
National	280.6(72.9)	104.19(27.1)	137.7(35.8)	289.77(75.3)	26.21(6.8)	18.1(4.7)
Facility type						
Public Hospital	46.1(64.3)	25.6(35.7)	17.74(24.7)	56.6(78.8)	4.1(5.7)	1.5(2.4)
Health centers	234.9(74.9)	78.6(25.1)	119.9(38.3)	233.6(74.5)	22.1(7.1)	16.6(5.3)
Health post	124.9(46.3)	144.9(53.7)	HEW			
Location						
Urban	163.7(73.1)	60.3(26.9)	78.1(34.9)	180.3(80.5)	14.6(6.5)	5.6(2.5)
Agrarian	105.9(75.5)	34.4(24.5)	49.3(35.1)	101.2(72.1)	11.0(7.8)	11.6(8.3)
Pastoral	11.31(54.5)	9.4(45.5)	10.2(49.3)	8.8(42.3)	0.7(3.32)	0.9(4.3)
VPD = Vaccine preventable disease						

7.3. VPD surveillance activities conducted by health facilities

The study also assessed the VPD surveillance activities being conducted health facilities. Table 7.2 shows what specific activities are undertaken by type of facility. Overall, about half of the study participants reported to have not received VPD surveillance related capacity building and about two-thirds of the health extension worker have not been trained on surveillance activities. Similarly, more than half of health facilities (52.57% perceive the communication and networking on VPD surveillance to be inadequate.

Table 7.2: Vaccine preventable disease surveillance capacity building, communication and networking in Ethiopia 2023.

	Training was given on VPD surveillance		SOP on VPD inves tigation		Adequate communication and network on VPD		VPD investigation forms		
	Yes	No	Yes	No	Adequate	Not adequate	Yes, observed	Yes, not observed	No
National	201.4(51.3)	191.2(48.7)	186.2(48.4)	198.6(51.6)	182.5(47.4)	202.28(52.6)	168.5(43.8)	92.7(24.1)	123.6(32.1)
Facility type									
Public Hospital	42.2(55.5)	33.9(44.5)	42.7(59.5)	29.1(40.5)	37.7(52.6)	34.0(47.4)	42.0(59.8)	8.8(12.3)	20.0(27.9)
Health centers	159.7(50.4)	157.3(49.6)	144.0(45.9)	169.5(54.1)	144.8(46.2)	168.7(53.8)	126.0(40.2)	83.9(26.8)	103.6(33.1)
Health post	89.7(33.2)	180.1(66.8)	218.2(80.9)	51.6(19.1)	141.8(52.6)	128.0(47.5)	44.6(16.5)	29.2(10.8)	196.0(72.7)
Location									
Urban	122.6(53.8)	105.5(46.2)	123.5(55.1)	100.5(44.9)	110.6(49.3)	113.5(50.7)	114.6(51.1)	50.0(22.3)	59.5(26.5)
Agrarian	68.5(48.1)	74.0(51.9)	54.7(38.9)	85.7(61.1)	68.6(48.8)	71.8(51.2)	50.5(36.0)	37.8(26.9)	52.1(37.1)
Pastoral	10.8(47.9)	11.7(52.2)	8.5(40.7)	12.3(59.3)	3.4(16.4)	17.3(83.6)	3.9(18.5)	4.88(23.5)	12.0(57.9)

VPD = Vac cine Preventable Disease; SOP = Standard Operating Procedure

7.4. VPD Surveillance Reporting

The reporting frequency of vaccine preventable diseases among the health facilities has also been assessed in this survey. Majority (65.51%) of the reports are submitted weekly according to the national schedule and about 15% of them were reported quarterly Table 7.3.

Table 7.3: Vaccine preventable disease surveillance reporting frequency by types and location of facilities in Ethiopia, 2023.

	VPD surveillance report frequency			
	Immediately	Weekly	Monthly	Quarterly
National	216.5(56.3)	252.1(65.5)	148.4(38.6)	60.5(15.7)
Facility type				
Public Hospital	44.0 (61.3)	40.8(56.9)	32.2 (44.9)	10.29(14.4)
Health centers	173.0(55.2)	211.6(67.5)	116.2(37.1)	50.22(16.0)
Health post	125.3(46.4)	182.8(67.8)	80.0(29.7)	29.46(10.9)
Location				
Urban	135.5(60.5)	143.5(64.0)	90.0(40.2)	39.17(17.5)
Agrarian	73.4(52.3)	94.4(67.3)	46.7(33.3)	18.68(13.3)
Pastoral	8.03(38.7)	14.6(70.4)	11.7(56.5)	2.67(12.9)
VPD= Vaccine Preventable Disease				

7.5. VPD Surveillance reporting frequency, timeliness and completeness

The findings on VPD surveillance reporting frequency, timelines and completeness that VPDs were reported immediately among 56% of the health facilities (health centers and hospitals) and 43% of the health posts. About 16% of the health facilities (health centers and hospitals) and 10% of the health posts reported VPD surveillance on quarterly basis. Only 19% of the health facilities (health centers and hospitals) and 8% of the health posts in Afar region reported VPDs immediately. Gambela region reported VPD immediately in all health facilities (health centers and hospitals), and only 19% of health posts in SNNP reported VPD immediately. Majority of the health facilities (66% of HC/Hospitals and 63% of the health posts) reported VPD cases on weekly basis Table 7.4

Table 7.4: Vaccine preventable diseases surveillance reporting frequency in Ethiopia, 2023.

	Hospital and health center(%)				Health post(%)			
Regions	Immediately	Weekly	Monthly	Quarterly	Immediately	Weekly	Monthly	Quarterly
Total	56	66	39	16	43	63	28	10
Addis Ababa	79	72	55	45	-	-	-	-
Afar	19	50	72	22	8	50	67	33
Amhara	75	60	29	19	62	55	31	16
Benishangul Gumuz	71	79	57	22	54	62	23	15
Dire Dawa	89	95	27	5	50	100	25	0
Gambela	100	89	35	12	40	70	10	0
Harari	74	93	67	67	56	56	31	0
Oromia	51	70	34	10	42	65	22	10
SNNP	42	64	40	4	19	46	38	3
Sidama	35	52	46	15	35	80	20	0
Somali	53	64	64	17	43	54	50	11
Southwest Ethiopia	49	95	33	18	28	67	39	11

Regarding report timeliness and completeness, from the total of surveyed health facilities, the weighted magnitude of report timeliness and completeness was 16.3% and 18.8%, respectively Figure 7.4. Report timeliness was relatively poor in the Somali region (68.18%), Afar (66.1%), and Sidama (63 %) and better report timeliness was observed in Addis Ababa (11.8%) and South-West Ethiopia (23.07%) Figure 7.5. Three out of five health facilities (57.2%) submitted report on timely basis whereas one third (34.2%) of the health posts did not submit any report 12 months preceding the survey. One-in-five facilities (18.8%), submitted incomplete report and 36.6% of public hospital submitted complete report on 12 months preceding the survey. Around 18.2% of the health posts and 13.3% did not adhere to the timeliness to report an outbreak and 16.6% of weekly submitted report were incomplete Table 7.5.

Table 7.5: Vaccine preventable disease surveillance report timeliness and completeness in Ethiopia, 2023.

	Monthly report timelines			Monthly report completeness			Outbreak report timeliness			Weekly report completeness		
	Timely	Not timely	No report	Complete	Not complete	No report	Timely	Not timely	No report	Complete	Not complete	No report
National	286.6(44.2)	105.9(16.3)	255.5(39.4)	187.1(49.5)	71.2(18.8)	120.0(31.7)	213.7(33)	96.6(14.9)	336.9(52.1)	236.2(44.0)	88.8(16.6)	211.4(39.4)
Types of facility												
Public hospitals	38.1(57.2)	6.2(9.3)	22.4(33.6)	36.6(54.9)	7.6(11.5)	22.4(33.6)	28.6(43.0)	6.2(9.3)	31.8(47.8)	38.8(58.3)	6.2(9.3)	21.6(32.5)
Health centers	156.3(50.2)	47.5(15.3)	107.7(34.6)	150.5(48.3)	63.6(20.4)	97.5(31.3)	94.3(30.3)	41.5(13.3)	175.1(56.3)	139.1(44.7)	47.5(15.3)	124.5(40.0)
Health post	92.2(34.2)	52.2(19.4)	125.4(46.5)	NA	NA	NA	90.9(33.7)	49.0(18.2)	130.0(48.2)	58.3(21.6)	35.2(13.1)	176.3(65.3)

NA – Not Applicable

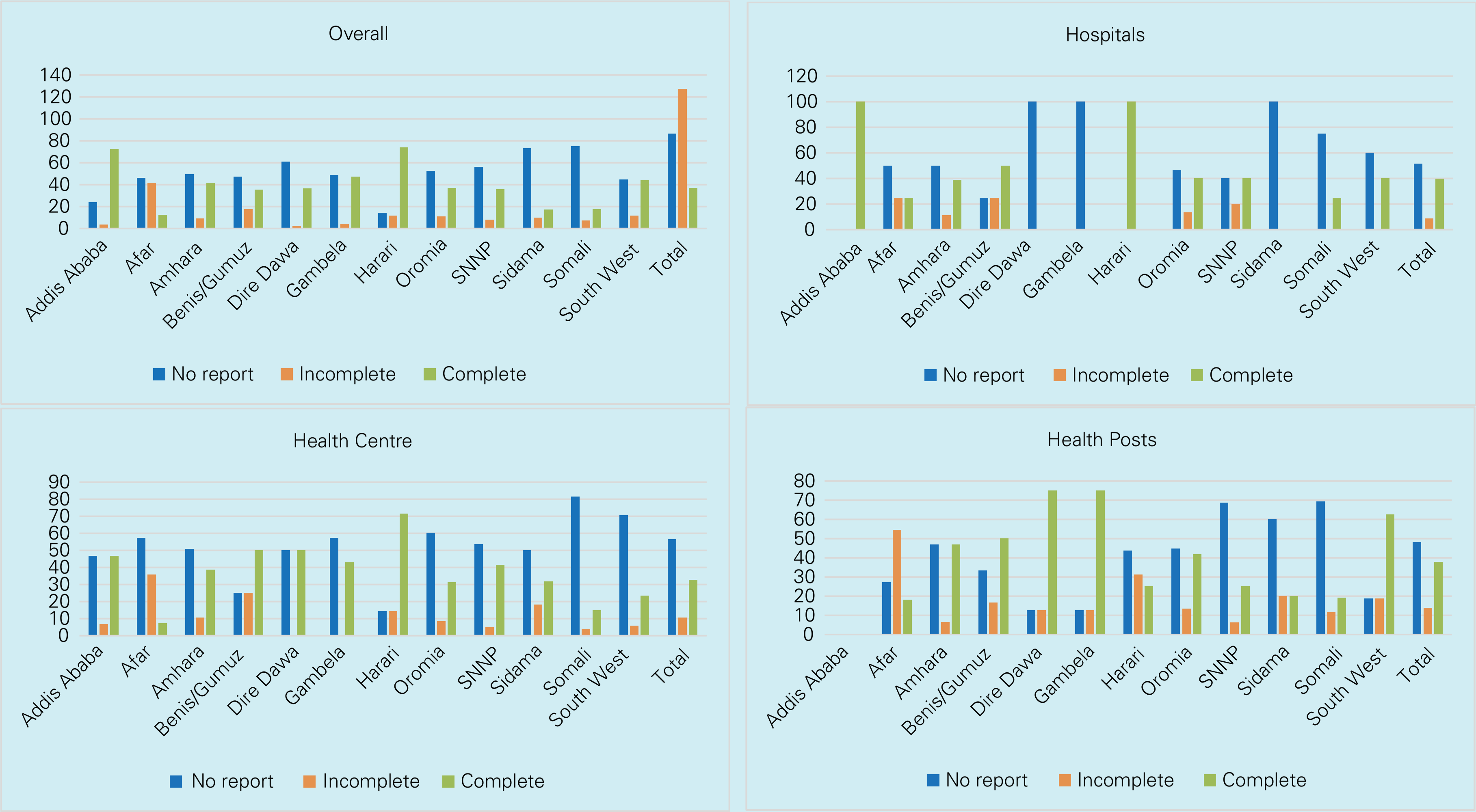

Figure 7.4: Report Completeness of VPD surveillance data among health facilities by regions of Ethiopia, 2023

7.6 Vaccine Preventable Diseases surveillance implementation challenges

surveillance implementation challenges
The status and quality of vaccine-preventable disease surveillance performance vary among regions and facilities. Though VPDs surveillance success depend on the ability of detection, notification, investigation, and laboratory confirmation of suspected cases, and response in the sector, the implementation of VPDs surveillance have still faced substantial challenges at all structural levels. This section presents the structural, technical and behavioural related challenges from in the VPD surveillance activities at different levels.

Structural challenges

Vaccine preventable disease surveillance data were collected from some woredas only of the country where most private health facilities are not well engaged in VPDs surveillance. Moreover, lack of lab equipment, insufficient capacity building to health workers working on EPI (according to one of the respondent from the federal training was not given for 1000 woredas in the country due to financial problems), absence of EPI/PHEM focal person at facility level, and shortage of reporting tools were forwarded as major structural challenges.

Technical challenges

Inadequacy of and delay in detection and reporting of VPDs was reported to be a major technical challenge. Further, delay in surveillance sample management, surveillance data quality problems (especially at lower level including health posts), inadequate advanced data analysis at national level, failure to regularly conduct VPDs surveillance in some region, VPD surveillance not considered as a routine activity in several facilities, no regular data analysis and use, weak case detection and reporting system, failure to implement active case search, weak evaluation of VPD surveillance performance, delay in reporting of suspected cases, and surveillance activities being done mainly during outbreak than routine immunization.

At woreda level it was reported that there was a persistent data quality issue within the VPD surveillance system, particularly in the reporting system. The data lacks timeliness, completeness, and accuracy. Despite multiple attempts were made so far at facility level, data quality problems continue to be a challenge. Though the VPD surveillance system includes a zero report, the qualitative results showed that there was a serious data quality and zero reporting problem at the facilities. Thus, zero reporting may not ensure the absence of the disease in the catchment areas during a specific period.

> *"…in our surveillance system, the problem is not on reporting; it is on detection. The use of zero reports is to know if facilities are working on surveillance actively or not. There is a problem in timely reporting also immediately notable diseases need to be reported within 30 minutes from each level, like woreda to zone to region. The guideline states that within two and a half hours of detection, it should be reported to the national level. But they may not report on time"* (Federal Level KI)

Behavioral challenges

Human related challenges and barriers are mention as, voluntary health workers not well engaged in VPDs detection and notification in the community, week community awareness creation and mobilization, under reporting due to lack of awareness on VPDs among community volunteers and health development army, shortage of lab professionals , lack of surveillance officers , HEWs and community health volunteers had no VPD surveillance training , low knowledge and skills of VPDs surveillance, , small number surveillance officers in lower levels, high turnover of focal persons and HEWs house to house visits decreased from time to time due to workload and low motivation.

Table 7.6: Summary of VPD surveillance implementation structural, technical and behavioral challenges in Ethiopia 2023.

Levels	Challenges			
	Structural	Technical	Behavioral	Suggested solutions
Federal	• Data quality challenges (national performance lacks report from all woredas)<br>• Shortage of lab professionals<br>• Shortage of surveillance officers<br>• HEWs and community surveillance agents are not well trained	• Delay in surveillance sample management for timely action<br>• Surveillance data management and quality problem<br>• Lack of advanced data analytics for informed decision	• HEWs and community with poor awareness about surveillance	• Work on human resource development<br>• Digitalization of the surveillance reporting system<br>• Expand the laboratory to the lower levels, especially for measles and Rota virus detection<br>• Stakeholder's engagement starts from the community to National level<br>• Strengthen feedback loop at all level to improve the surveillance system<br>• Conduct regular supportive supervision and provide feedbacks<br>• Facilitate sample transportation, testing and timely notifying the results
Region	• Shortage of inputs including laboratory capacity<br>• No system to engage stakeholders , including private facilities<br>• Voluntary health workers, private sector providers, etc are not participating in VPD surveillance<br>• Shortage of PHEM officers at some structures (especially subcity and woreda)	• No regular data analysi and use<br>• Not give technical suppo for all private and public facilities in Addis Ababa city,	• small number surveillance officers<br>• Mainly engage during outbreak,<br>• workload in Addis Ababa sub city level,	• Equipregional PHI laboratories<br>• Facilitate sample transportation, testing and timely feedback<br>• Providefocusedtraining to lower levelon VPD surveillance<br>• Conduct regular supportive supervision and provide feedbacks
Zone	• No system to engag stakeholders , includinc private facilities	• Data quality problem,<br>• Missing actual case in the community due to low case detection,<br>• False VPDs data reporting<br>• No regular data verification		• Set platform to engage all stakeholders
Woreda	• Lack of voluntary healthcare workers an a private health sector dedicated to VPD surveillance activities,	• Data quality problems and exchanging of reports<br>• Zero reporting gap	• Data analytics gap<br>• No surveillance officer only the focal person<br>• High turnover	• Capacity building on data analysi interpretation and use<br>• Strengthening data handling and documentation<br>• Community case definitions should be explained in the local language for better understandin<br>• Woreda administrators and healtl office heads should work on ensuring the availability of emergency vaccines.

Health facilities	• absence of focal person at facility level,<br>• VPD surveillance not considered as a routine activity of facilities	• weak active case detection and reporting<br>• data lacks relevant information's, child vaccination status of the child, incomplete data,<br>• data quality issues	• HWs had no VPD surveillance training<br>• lack of lab personnel and clinicians for sample collection and case detection,	• Developthe capacity of staff on data analysis and use<br>• Private sectors should use proper registration and documentation forms and submit report
Health post		• Failure to implement active case search<br>• Shortage of reporting tools<br>• weekly reporting formats,<br>• No monitoring system o[illegible] VPD surveillance activities<br>• Delay in reporting of suspected cases	• House to house visits decreased from time to time due to workload and low motivation of EHWs	• Capacitate HEWs on identificatio[illegible] listing and reporting of cases<br>• Strengthening the link between HEWs, health volunteers, and th[illegible] community<br>• Provide timely feedback from health centers to HEWs
Community	• Absence of volunteer health workers in the community	• week community awareness creation and mobilization<br>• under reporting due to lack of awareness on VPDs,	• Inadequate awareness of community platforms (WDA/HAD) case notification	• revitalizeWDA/HDA leaders and community volunteers<br>• Engage the community in the identification and notification of VPD<br>• Make the community as part of the response

VPDs surveillance implementation challenges across different levels

National level

It is evident that weak surveillance and monitoring data can lead to flawed planning and decision making for immunization activities. The national VPD surveillance system has various structural, technical and human resource related challenges in the sector. At the national level, the VPD surveillance data has quality issues such as, incomplete, accuracy and delay in reporting. Delay in reporting, low skills in detection and notifying of cases, the VPD surveillance data not integrated with the routine HMIS reporting system, lack of zero reporting and poor case detection in the community and facilities. The other gap reported is the inadequate engagement of private facilities in VPD surveillance activities including identification, reporting and management. Suboptimal advanced analysis surveillance data was also another reported challenge.

Subnational level

VPD surveillance data quality (timelines, completeness, accuracy, e.t.c.) and information use has not monitored and evaluated at regional level, it is expected to be monitored and evaluated at national level, EPHI. Private facilities were not well participating in VPD surveillance, delay in reporting and response, gap in coordination, poor case detection and reporting, false reporting, data accuracy (discrepancy between data reported by the PHEM focal and by the EPI focal), irregularities in technical support by partners, and gaps in pre-service training for health professionals. However, the direct line of the communication with EPHI regarding VPD surveillance made the reporting more effective for timely feedback.

Shortage of human resources/surveillance officers, low motivation of professionals, high turnover and transfer of focal persons, low motivation to stay in the surveillance program, health extension workers and community health volunteers having inadequate case detection skills due to lack of VPDs surveillance training. Clinicians in the facility also lack awareness and skills on VPDs surveillance. There was difference on availability of surveillance officers from facility to facility and from region to region.

Supply related challenges including shortage of lab supplies and equipment, especially at a regional laboratories, shortage of reporting formats at facility level, low finance and human resource to cascade VPDs training to lower levels.

Lack of report and data inaccuracy from health posts, no data validation work, reporting of zero cases without enough case detection the community was also a challenge at lower level of the health system.

> *"There is no system for engaging other stakeholders, including the private facilities in the VPD surveillance system, except the already available governance structure settled by Ministry of Health."* (KII, Zonal EPI focal)

There is a persistent data quality issue within the VPD surveillance system, particularly in the reporting system in some regions. Timeliness was a significant problem reported from Somali region due to shortage of transport, limited network connectivity. Lack of stakeholder and private facility engagement in VPDs surveillance was also cited to affect the service delivery.

> *"There is a persistent data quality issue in the VPD surveillance system, particularly in the reporting system. The data lacks timeliness, completeness, and accuracy. Despite multiple attempts to address this issue through reporting facilities, data quality problems continue to persist…."* (KII, Woreda EPI focal)

Weak feedback mechanism, low awareness among health professionals about the VPD surveillance activities, poor data handling and reporting using paper or phone call, work load, absence of lab technician to collect the sample from the suspected cases in the community were reported at health facility and health posts.

7.7 Conclusions

The findings indicate that measles, rubella and neonatal tetanus cases were reported from all regions of the country, though there was variation of cases among the regions. Regions need to focus on strengthening the provision of immunization service through targeted identification of unvaccinated eligible children, improving the surveillance activities through early identification, reporting and management of cases. The gap in terms of human resource to conduct VPD case identification and management has negatively impacted the early case detection and management. This gap is evident in which more than one in four health facilities do not have a focal person to conduct VPD surveillance activities with significant variation among health posts whereby more than half (53.7%) not having assigned VPD focal. Further, two-thirds of the health extension worker have not been trained on surveillance activities. The VPD surveillance activities need to be given attention and capacitated health professionals should be assigned at lower levels of the health system with particular focus for health posts.

Nationally, only half of the health facilities reported immediately reportable cases to higher levels with significant regional variation. Although the reporting problem is present in most regions, it is amplified in regions such as Afar and Somali. Unless cases are reported immediately for further investigation and management, the risk of transmission to the wider population and the human life costs implicated are very serious. The monitoring and

evaluation of the program should be strengthened through assigning and capacitating the required health professionals that can respond to the identified problem timely. Though VPDs surveillance success depend on the ability of detection, notification, investigation, and laboratory confirmation of suspected cases, and response in the sector, the implementation of VPDs surveillance have faced substantial structural, behavioural and technical challenges. Designing appropriate interventions addressing these bottlenecks requires concerted effort between ministry of health, EPHI, regional health bureaus, woredas, health facilities and other stakeholders.

7.8 Recommendations and Implications

- Regions should to focus on strengthening the provision of immunization service through targeted identification of unvaccinated eligible children, improving the surveillance activities through early identification, reporting and management of cases.
- The VPD surveillance activities need to be regularly conducted in the routine immunization program through regular follow up and monitoring.
- Capacitated health professionals should be assigned at lower levels of the health system with particular focus for health posts to effectively conduct VPD surveillance activities.
- The EPHI need to closely work with the ministry of health in surveillance activities,
- The surveillance data should be incorporated with the routine report of HMIS for timely decision making.
- The Monitoring and evaluation of the surveillance for VPDs need to be strengthened through routine reporting of surveillance data even in the absence of cases, i.e., zero case reporting, where applicable.
- The VPD surveillance should strengthen efficient and timely collection of the relevant data needed for decision making, including case-based data on age and vaccination status, pinpointing specific diseases by geographic location and affected groups to allow for risk assessment and evidence-based vaccine use
- EPHI and the ministry of health should building capacity of health professionals at all levels; for core functions including case detection, notification, investigation, reporting, and response; epidemic preparedness

DEMAND GENERATION AND COMMUNICATION

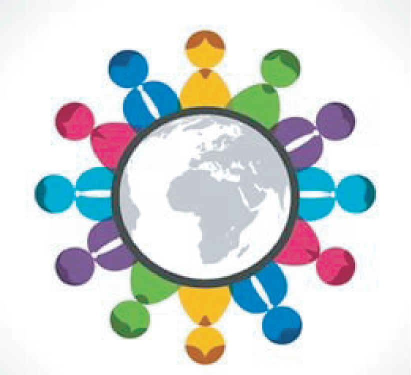

8. Demand Generation and Communication

Key findings

Childhood vaccination: awareness, attitude and acceptance

- Large majority (94.5%) of mothers ever heard about childhood immunization information and most mothers (97.3%) knew where to get immunization services.
- Open multi vial policy, issues related to accessibility and availability of services, and gaps in IPC skills of providers are impediments to vaccination uptake

COVID-19 vaccine: awareness, attitude and acceptance provide support to get COVID-19 vaccine

- Half (50.3%) reported that religious leaders
- Half (50.1%) of respondents have favorable attitude about COVID-19 vaccine
- There were diverse misperception and misinterpretation of COVID-19 vaccine,and absence of vaccine, fear of side effects and inaccessibility were major barriers for uptake

HPV vaccine: awareness, attitude and acceptance

- Majority of adolescents knew HPV vaccine helps to prevents cervical cancer (85%). However, a staggering 46% eligible adolescents have not been vaccinated for HPV
- Lack of information about vaccination site, service unavailability when needed and inaccessibility of services were main barriers reported to HPV vaccine uptake

Interpersonal communication(IPC) skills: practices and barriers

- Less than half of health workers (41%) observed, providedinformation on the type of antigens given and about one-third (36.7%) counseled on potential adverse effect with
- Regional variations were observed in IPC skills of health workers with participants from regions such as Oromia, Amhara, and Sidama performed better than Afar and Gambella.

Immunization program ACSM practices, challenges and suggestions

- A combination of platforms such as community structures, schools, public gatherings, religious institutions, and medias were employed in EPI communication and demand generation
- EPI demand generation and communication activities are commonly done for campaigns than routine immunizations
- Weakening community structures, local insensitiveness of communication materials, growing perception of demand creation as a finished agenda were the major challenges in EPI Advocacy, Communication and Social Mobilization (ACSM).

Demand generation and communication is a critical component of immunization program. It is an effective strategy that helps ensure the uptake of immunization services by the target population. This chapter presents awareness, attitude/acceptance, hesitation and mitigation strategies of childhood vaccinations, COVID-19 and HPV vaccinations. It also presents information about immunization program Advocacy, Communication and Social Mobilization (ACSM) practices, challenges and suggested strategies in Ethiopia. The information is useful in understanding the factors that determine uptake of immunization program from the demand side and design effective communication and demand generation strategies.

To explore the immunization demand generation and communication, a total of 13,666 mothers were interviewed for childhood immunization which assessed awareness about vaccination, sources of information, attitude towards vaccination as well as utilization of immunization services. For COVID -19 vaccination, a total of 13,812 adults aged 12 years and above were included and awareness about the vaccination, sources of information for vaccination, attitude towards vaccination as well as utilization of immunization services were addressed. In addition, reasons for non-immunization as well as future intention to take the vaccine were assessed. For the HPV vaccination, a total of 5336 adolescent girls aged 15-18 were interviewed.

8.1 Childhood vaccinations: Awareness, acceptance/attitude, hesitance and mitigation

Childhood vaccination information and its sources

Of the total caregivers responded to childhood vaccination survey questionnaire, 13757 (94.5%) reported that they have ever heard about immunization services. Among these, the majority 12688, (92%) mentioned that healthcare workers were their main source of information for vaccination. Moreover, 9032 (65.7%) of study participants recalled the messages they heard about immunization. Out of these, 8111 (89.8%) and 2611 (28.9%) remembered benefits of vaccination and the place where to get immunization services respectively Table 8.1.

Table 8.1: Childhood vaccination information, source of information and contents of information among caregivers in Ethiopia, 2023.

Variables	Frequency	Percent
Ever heard about immunization	13757	94.5
Source of information		
Health care Worker	12688	92
Community and religious leaders	2445	18
Neighbors	2147	16
Friends	1474	11
Television	1406	10
Radio	1241	9
Women development army (WDA)	1088	8
Social Media	616	4
IEC/SBCC	195	1
Other	185	1
SMS	173	1
Total	13757	100
Mothers who recalled any immunization message	9032	65.7
Immunization messages recalled by mothers (n=9032)		
Benefit of Vaccination	8111	89.8
Place of immunization	2611	28.9
Date of next immunization	1922	21.3
Types of vaccine	1452	16.1
Immunization schedule	1427	15.8
Side effects	693	7.7
Keeping of immunization card	602	6.7
Other	47	0.5

The qualitative findings also supported the quantitative findings that most of the respondents had positive perceived benefits of childhood vaccination. One of the participants stated,

> *"Our communities hold positive attitudes towards immunization. They recognize its benefits and do not have any negative beliefs. In the past, our children faced various illnesses; however, nowadays, thanks to immunization, they are in good health. Therefore, our communities have developed favorable attitudes towards immunization.* (Male FGD: P8)

Knowledge of childhood Vaccination

Out of 13,757 caregivers who had information about childhood vaccination, only 3615 (26.27%) reported that they knew at least one type of childhood vaccine antigen. Among those who had information about the antigens, the most commonly mentioned antigen/vaccine by the caregivers was Measles, 2271(63%), followed by Polio 2132 (59%). More than half of the respondents 9253 (53%) Knew that immunization prevents their children from vaccinate preventable diseases and 7253 (41%) mentioned that it helps to grow healthy children. Moreover, 13379 (97.3%) mothers indicated that they knew where to get vaccination services in their areas. Almost two-fifth of them mentioned that vaccination services were available in health posts and health centers Table 8.2.

The qualitative part also supported the quantitative finding in which most of the respondents were not able to differentiate the antigens and the exact age at which they are given except the 9 months' measles.

> *"Even though there were general awareness that children are eligible for vaccination, awareness of the specific age groups, for which type of vaccine and which types of disease it prevents was lacking in the community. It appears that community were not able to link each antigen with the disease it targets and age wise, except for the nine-month vaccine where they realize that it is intended to prevent measles"* (FGD).

Although most of the respondents mentioned the EPI target diseases, many respondents did not know the EPI program schedule of each vaccine and their different appointment time as well as the target groups for the antigens. Findings from qualitative data also showed knowledge about vaccination is universal but people lack specific knowledge about the target and schedule as well as the dose

> *".……the community is well oriented and they know what services is offered at the health institution which can be taken as strengths"* (KII, EPI provider)

Table 8.2: Knowledge of childhood vaccination among mothers/caregivers in Ethiopia, 2023.

Variables	Frequency	Percent
Mothers who Knew at least any of the vaccine antigens	3615	26.3
Common vaccines mentioned by the respondents		
Measles	2271	62.8
Polio (Poliomyelitis)	2132	59.0
BCG (Tuberculosis)	1055	29.2
Pertusis (whooping cough)	685	18.9
TT/Td (Tetanus) for mothers	543	15.0
Covid-19	422	11.7
Rota(Diarrhea)	409	11.3
PCV (Pneumonia)	343	9.5
Diphtheria	314	8.7
H-inf (Haemophilus influenza)	289	8.0
TD for children	242	6.7
Hep-B (Hepatitis B)	214	5.9
HPV (Human Papilloma Virus)	120	3.3
Total	3615	100
Mothers knowledge of vaccine benefits		
Mothers who knew that vaccine prevents from the disease like TB, Polio, pertussis, measles	9276	67.4
Mothers who knew that vaccine helps children to grow healthy	7253	52.7
Mothers who Knew vaccination service sites	13379	97.3
Sites mentioned by mothers (n= 13379)		
Health post	8443	39.1
Health center	8421	39.0
Out reach	2732	12.7
Hospital	1647	7.6
Private clinic	208	0.96
Pharmacy	12	0.06
Other	116	0.54
Total	13757	100

Childhood vaccination acceptance and intention to vaccinate children

Out of the total 14551 surveyed children, 6388 (43.9%) were fully vaccinated and 24.7% did not receive any kind of vaccines. Out of the total unvaccinated children, for about (70.8%) their caregivers had willingness to vaccinate their children in the future Table 8.3. The finding from the qualitative data also indicated that majority of the community members had positive attitude and accepts childhood immunization.

> *"The community members in our catchment area have generally exhibited a positive attitude and have been supportive of expanding immunization coverage within the community. However, there are instances where community members bring individuals who have discontinued their immunization schedule before completion."* (KII, EPI provider)

Table 8.3: Childhood vaccination status and willingness of mothers to get their children to be vaccinated, Ethiopia 2023

Variables	Frequency	Percent
Number of children who has taken-		
All recommended vaccines	6388	43.9
Some recommended vaccines	4569	31.4
None of recommended vaccines	3594	24.7
Total	14551	100
Mothers of unvaccinated children who have intention to vaccinate children in the future		
Yes	2545	70.8
No	1049	29.2
Total	3594	100
Mothers with partially vaccinated children who have intention to vaccinate remained antigens (n=4569)		
Yes	2518	55.1
No	2051	44.9
Total	4569	100

The main reported reasons for non-vaccination and vaccination dropouts were inaccessibility of vaccination sites (28%) followed by lack of immunization information (20%) and consideration of vaccine as not relevant (19%) Figure 8.1. The qualitative finding has also revealed many factors for non-vaccination and vaccination dropouts. The major reasons for vaccination drop outs fall in to three categories:

- Communities settlement characteristics and livelihood (mobility, hard to reach sites): In settings where the communities are mobile due to various reasons (e.g., livelihood activities such as gold mining), drop out was major problem. Key informant expressed this condition as:

"In our case, after taking penta1, significant number of children don't come for the second and third dose that may be due to long distance to reach the health center, being not able to afford transportation cost and some communities' belief that if the child gets the first dose it will be quite enough. Negligence and forgetting the child's immunization schedule is the other reason for drop out." (KII, EPI provider)

- Conflicts or security: Security problems were reported as major reason for immunization dropouts due to destruction of health system and mobility of communities.

- Beliefs and perceptions about immunizations and health concerns: In some areas communities' belief that one dose of vaccination was adequate, and as a result they don't visit health facility for immunizations. Moreover, negligence and forgetfulness of immunization dates were also reported as reasons for dropouts from immunization services.

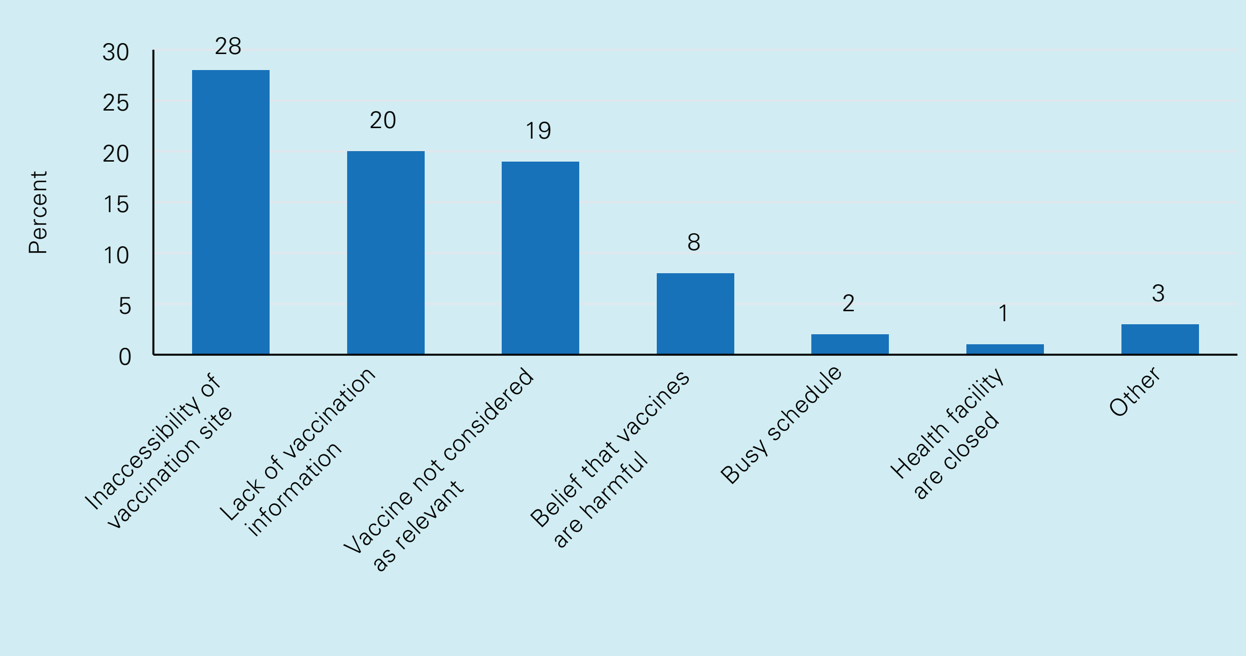

Figure 8.1: Reasons for mothers/caregivers not vaccinated their children in Ethiopia, 2023

Community participation in immunization plays a crucial role in addressing the challenge of zero doses for vaccination, which refers to children who have not received any vaccines at all. This issue often stems from various factors such as lack of awareness, misinformation, logistical barriers, and cultural beliefs. We tried to see the magnitude of zero dose and community participation in immunization. Regions like Afar and Somalia with low involvement of community had higher rate of zero dose Figure 8.2.

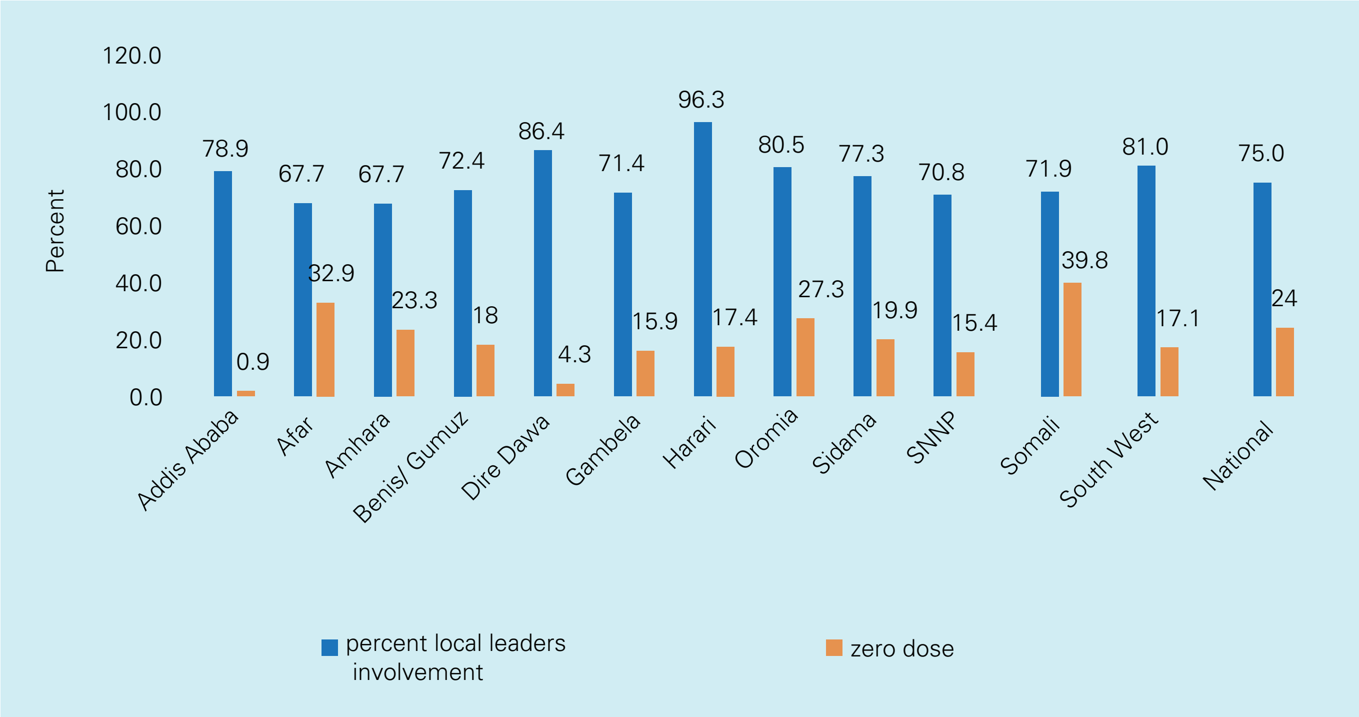

Figure 8.2: Relationship between local leaders' involvement and magnitude of Zero dose in Ethiopia

8.2 Community's perception and attitude towards childhood vaccination

Respondents were also asked to rate the importance of childhood vaccination, accordingly 11557 (84%) and 10689 (77.7%) perceived that childhood vaccination is very important and very safe for their children. Majority 10622(77.2%) of them stated that they highly trust health workers to provide vaccination for their children Table 8.4.

Table 8.4: Community perception and attitude towards childhood vaccination in Ethiopia, 2023

Variables	Response	Frequency	Percent
How important do you think vaccines are for your child's health?	Very important	11557	84.0
	Moderately important	1540	11.2
	A little important	478	3.5
	Not at all important	182	1.3
How safe do you think vaccines are for your child?	Very safe	10689	77.7
	Moderately safe	2318	16.9
	A little safe	564	4.1
	Not at all safe	186	1.4
How much do you trust the health workers who give children vaccines?	Very much	10622	77.2
	Moderately	2689	19.5
	A little	311	2.3
	Not at all	135	1.0
Total		13757	100

Perception of support and approval for childhood vaccination

Large majority 12051(82.8%) of surveyed caregivers/mothers responded that most parents they knew have vaccinated their children. With regards to support and encouragements about childhood vaccination, 12524(86.1%) mothers perceived that their close family and friends support them to vaccinate their children. Additionally, 10071(69.2%) mothers perceived that religious leaders gives supports and another 11401(78.4%) perceived that community leaders encourages them to vaccinate children(table 8.5). In support to this, FGD participants has indicated that majority of families and community leaders accept vaccination.

> *"The community views this vaccine as a good thing for children, most of the community members love child immunization above anything else" (Male FGD: P6).*

> *"Sometimes husbands remind their wives of the vaccine appointment date or the schedule. They encourage to vaccinate their children. Overall it is encouraging, people like this vacation." (Female FGD: P8)*

Nearly four in ten mothers (5710 or 39.2%) have required parents' permission to vaccinate children with only 8840(60.8%) of them have taken their children without parents' approval confirmation.

Table 8.5: Perception of support and approval for childhood vaccination in Ethiopia, 2023

Variables	Response	Frequency	Percent
Do you think most parents you know get their children vaccinated?	Yes	12051	82.8
	No	2500	17.2
Do you think most of your close family and friends want you to get your child vaccinated?	Yes	12524	86.1
	No	2027	13.9
Do you think your religious leaders want you to get your child vaccinated?	Yes	10071	69.2
	No	2007	13.8
	I don't Know	2473	17.0
Do you think your community leaders want you to get your child vaccinated?	Yes	11401	78.4
	No	1419	9.7
	I don't know	1731	11.9
Has a health worker recommended your child be vaccinated?	Yes	13176	90.6
	No	1374	9.4
Have you ever been contacted about your child being due for vaccination?	Yes	9095	62.5
	No	5456	37.5
If it was time for your child to get vaccinated, would you need permission to vaccinate your child	Yes	5710	39.2
	No	8840	60.8
Total		14551	100

8.3 Perceived access and level of satisfaction with vaccination services

Large majority 12566 (86.4%) of mothers have ever taken their index children for immunization services. Out of these, 65.5% perceived that immunization site was near to their home, and 34.5% of them perceived that the site was either far or too far. While four out of ten mothers perceived waiting time for immunization services as medium (41.8%) and short (45.3%), 12.9% perceived that waiting time for the services was too long. One-fifth of the mothers (19.2%) have ever been turned away without vaccinating their children Table 8.6.

Table 8.6: Perception and experience of mothers with childhood vaccination services in Ethiopia, 2023

Variables	Response	Frequency	Percent
Have you personally ever taken your index child to get vaccinated?	Yes	12566	86.4
	No	1985	13.6
What is your perceived distance of immunization site from your home?	Too far	1094	8.7
	Far	3244	25.8
	Near	8227	65.5
During your last visit what was your perceived waiting time for the immunization	Too long	1621	12.9
	Medium	5252	41.8
	Short	5693	45.3
Have you ever been turned away when you tried to get your child vaccinated?	Yes	2790	19.2
	No	11760	80.8
Total		14551	100

Respondents also asked about perceived access to vaccination service, the majority 11992 (87.2%) reported that access to vaccination was either very easy or moderately easy. Among mothers who perceived access to vaccination as difficult, 1039 (59%) mentioned that vaccination sites were too far or inconveniently located. With regards to caregivers' satisfaction, 93.5% were either very or moderately satisfied with vaccination services Table 8.7.

Table 8.7: Perceived access to vaccination services and level of satisfaction among mothers/caregivers in Ethiopia, 2023

Variables	Frequency	Percent
How easy is to access vaccination		
Very easy	8035	58.4
Moderately easy	3957	28.8
A little easy	985	7.2
Not at all easy	781	5.7
Total	13758	100
If not at all easy or a little easy what were the reasons		
Getting to the service is too far	1039	59
The service site opening times are inconvenient	420	24
The facility sometimes turns people away without vaccinating due lack of vaccine	400	23
The facility sometimes turns people away without vaccinating due lack of provider	272	15
The facility sometimes turns people away without vaccinating due to Multi-dose policy	124	7
Waiting time too long	175	10
Providers attitude	112	6
Others	179	10
Total	1766	100
Caregivers level satisfaction with vaccination services		
Very satisfied	8476	67.5
Moderately satisfied	3264	26.0
A little satisfied	581	4.6
Not at all satisfied	245	1.9
Total	12566	100

The main reasons for caregivers' dissatisfaction with vaccination services were vaccine unavailability whenever required (62.8%) followed by longer waiting time to get vaccination services (34.7%) and closure of service unit during service hours (24.8%) Table 8.8. Similarly, the qualitative data also revealed many factors as reason for vaccination non-use and dropout. The main factors mentioned by participants were issues related to open multivial policy, and service accessibility and availability (such as distance, shortage of HR). The participants underscored that multivial policy applied for some of antigens in the national immunization program enforces healthcare providers to wait adequate number children before opening vials. These situations create dissatisfaction among the clients as it resulted to longer waiting time and/or get away from the facility without vaccination.

> *"Some antigens (e.g., measles) included in the Ethiopia immunization program are prepared for many children and the national immunization program recommends delivery of those antigens if only sufficient number of children were available. Such situations have caused major challenges to the providers and resulted to escalated dropouts* (KII, EPI provider).

Service inaccessibility and unavailability (distance, shortage of HR) were also cited as impediments for immunization service uptake and drop outs in Ethiopia. Inaccessibility of services particularly in hard-to-reach areas coupled to the shortage of EPI providers at all level of the health system were identified as the major challenges of immunization program in Ethiopia.

"In some areas, such as hard-to-reach areas; vaccination services are inaccessible to communities. Communities travel long distances to receive the first dose of vaccination, but they will not complete the successive doses. Particularly, mothers face difficulties to carry the child long journey and vaccinate them. In areas with shortage of providers (particularly the HEWs); dropouts were higher. Such situations impede the HEWs to implement various strategies such as outreach strategies in areas that are difficult to access the community (KII: EPI provider at health center).

Table 8.8: Reasons for dissatisfaction of caregivers/mothers with immunization service in Ethiopia 2023

Reasons for dissatisfaction	Frequency	Percent
Vaccine is not always available	519	62.8
Longer waiting time	286	34.7
Service unit does not open on time	205	24.8
Lack of adequate information from provider	140	17.0
Staff are not respectful	120	14.5
Staff do not spend enough time with people	83	10.0
No comment	47	5.7
The service unit is not clean	16	1.9
Distance of the Health facility	14	1.7
Side effect of vaccine	4	0.5
Others	8	1.0
Total	826	100.0

8.4 Role of Gender in immunization service uptake/Gender inter-sectionality

Understanding of vaccine decision making process is vital to design effective SBCC strategies for EPI program. In relation to this, gender interplays significant roles. Though male involvements in child vaccinations has paramount importance, the national evaluation revealed that mothers are most commonly engaged in vaccinating children. Mothers are more important and plays key role in child immunization decision making process and facilitation of vaccination related activities at household level. Whereas, fathers/husbands play supportive roles such as reminding vaccination dates, and encouraging

mothers once the schedule is known. Experienced or senior mothers makes vaccination decisions herself but informs husband for approval whether the baby has to receive the vaccine and when to receive it. For younger and inexperienced mothers, decisions about new baby vaccinations is made and facilitated by mother-in-law. There are also some instances where close neighbors facilitate and support vaccinations for new mothers following the decisions made by parents. The study has also revealed that though male support and awareness is important for child vaccinations, and the wife/mother is expected to inform or notify the vaccination decision, it is permissible to the mother to proceed to vaccination even without the
final say or approval of father/husband. This is because child vaccination is generally believed as beneficial for children and there is no serious concern over child vaccination, as discovered in the current evaluation.

Overall, the finding indicated that gender intersects vaccination decisions. Male/husbands has less exposure to immunization information whereas female/mother had greater chance of exposure to vaccination information at multiple times such as during ANC, and delivery. Women/mother had more chance of contact with routine immunization services, and men/fathers had greater opportunity to information when the immunization program is conducted in campaign and during activities that would engage communities. Moreover, participants explained that WDA are the major source of immunization information; implying that vaccine information largely flow through women networks than men networks. On the other hand, in relation to age of the baby; it is mothers' responsibility to give care (including taking to vaccination site) during the first few months of life and this situation in itself reduces the involvement of male/fathers in child vaccinations. Mothers have better awareness, exposure and close attention to children vaccination due to various reasons because they do have better chance of exposure to vaccine information.

8.5 COVID -19 infection and vaccine: Awareness, beliefs, acceptance and hesitations

Respondents' characteristics

In the COVID-19 vaccine survey, 13812 adults age greater than 12 years were participated. Nearly two-fifth (38.9%) of respondents were from Oromia region followed by Amhara (22.3%). Majority of respondents were from agrarian setting (66.4%), in the age range of 20-39 years (78.3%) and females (74.8%). With regards to education, slightly less than half (47.1%) had no formal education and one-thirds (31%) had attended primary education. During the COVID-19 pandemic, nearly 4 in 10 (38.8%) of respondents have been farmers Table 8.9.

Table 8.9: Participants' characteristics of COVID-19 disease and vaccine survey, Ethiopia, 2023.

Variable	Category	Frequency	Percent
Region	Addis Ababa	520	3.8
	Afar	275	2.0
	Amhara	3083	22.3
	Benishangul Gumuz	164	1.2
	Dire Dawa	74	0.5
	Gambela	72	0.5
	Hareri	37	0.3
	Oromia	5373	38.9
	Sidama	614	4.4
	SNNP	2273	16.5
	Somali	886	6.4
	Southwest Ethiopia	442	3.2
Setting	Urban	3321	24.0
	Agrarian	9173	66.4
	Pastoralists	1318	9.5
Age	12-19	841	6.1
	20-39	10819	78.3
	40-59	1966	14.2
	>=60	186	1.3
Sex	Male	3463	25.2
	Female	10255	74.8
Marital status	Married and living together	12225	88.8
	Married but not living together	345	2.5
	Currently not in marital union	1192	8.7
Educational status	No formal education	6503	47.1
	Attended primary education	4295	31.1
	Attended secondary education	2020	14.6
	Attended college or university education	994	7.2
Work During Covid-19	Health care worker	394	2.9
	Teacher	227	1.6
	Merchant	1092	7.6
	Farmer	5661	41.0
	Other government and private organization employee	877	6.4
	Student	884	6.4
	Other	4677	33.9
Wealth Index	Lowest	2587	18.7
	Second	2785	20.2
	Middle	2862	20.7
	Fourth	2873	20.8
	Highest	2704	19.6
Total		13812	100

COVID-19 information and its sources

Most of surveyed participants, 13074 (94.7%) ever heard about COVID-19. Out of these, 69.2% heard about coronavirus is a deadly disease and 30.9% heard that wearing masks prevents COVID-19 Table 8.10. Insights from the qualitative participants have also showed that awareness about existence of COVID-19 was reportedly universal.

Table 8.10: Awareness about COVID-19 among study participants, Ethiopia, 2023

Variable	Frequency	Percent
Ever heard about COVID-19	13074	94.7
Information heard about COVID-19		
Coronavirus is a deadly disease	9043	69.2
Wearing of masks prevents COVID-19	4041	30.9
Close contact between peoples with the virus transmits COVID-19	3533	27.0
COVID-19 transmits during coughing	3279	25.1
COVID-19 is preventable	3099	23.7
Utilization of sanitizers prevents COVID-19	2513	19.2
COVID-19 has vaccine	2317	17.7
COVID-19 is transmitted by a virus	2243	17.2
Vaccinations prevents COVID-19	610	4.7
Other	340	2.6
Perceived causes of COVID-19		
Corona virus	3115	23.8
Bacteria	928	7.1
Unknown	668	5.1
I don't know	8348	63.9
Other	391	3.0
Total	13074	100

Regional disaggregated data showed variations in the proportion of respondents who heard about COVID-19. The proportion of respondents who heard about COVID-19 ranged from 98% in Addis Ababa and Afar regions to 86.2% in Somali region Figure 8.3 .

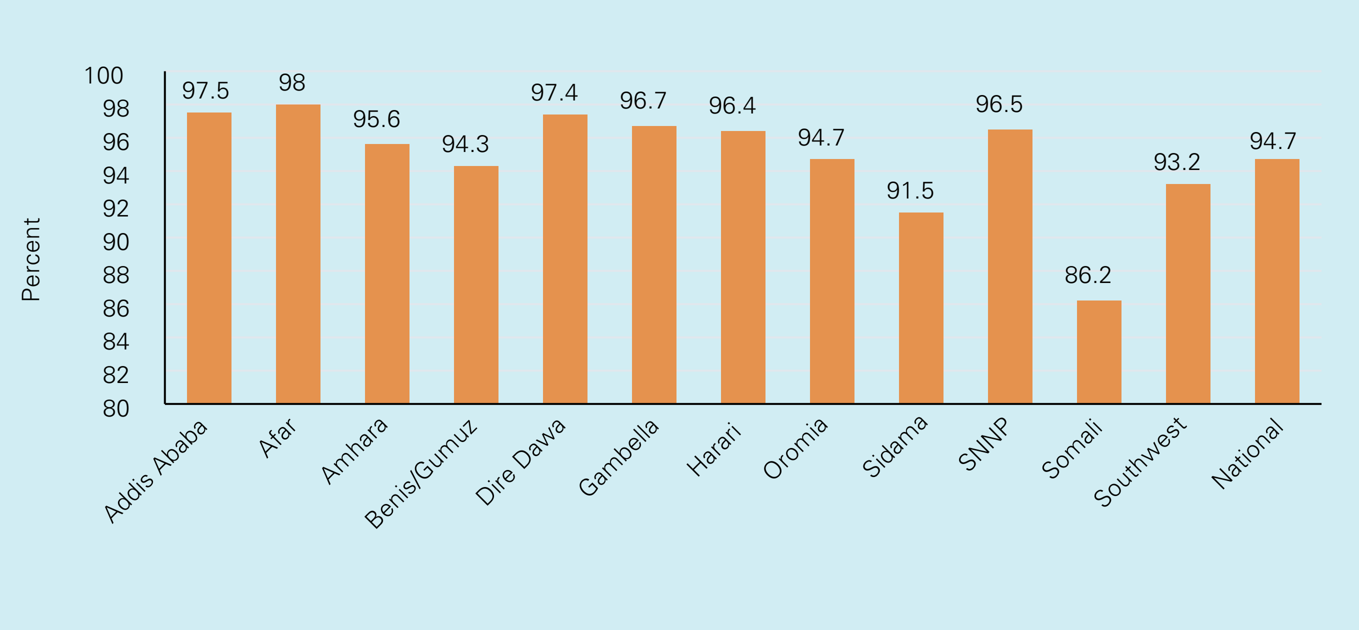

Figure 8.3: Percentage of respondents who ever heard about COVID -19 by region, Ethiopia 2023

The study revealed that audio mounted vehicle or Montarbo 6786(51.9%), healthcare workers 2909(22.2%), social media 2211(16.9%) and the religious leaders 1776(13.6%) were major sources of information about COVID-19 vaccine Figure 8.4.

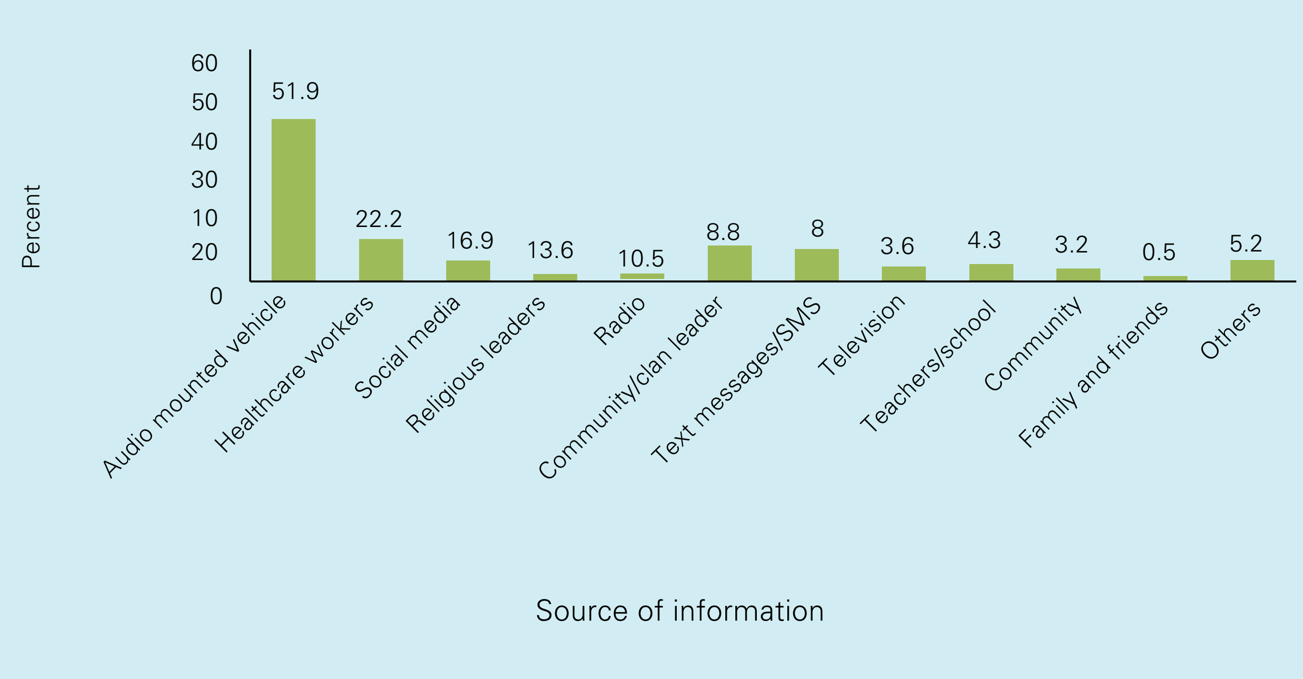

Figure 8.4: Source of information for COVID-19 vaccine among Study participants, Ethiopia, 2023

Findings from the FGDs and KIIs (especially at community level) indicated that there were huge and diverse misperceptions and misinterpretation of COVID-19 disease. Accordingly, COVID-19 was largely perceived as "fake or unreal disease", and created for political and societal manipulation purpose. There was strong belief that the COVID disease was none-existent and also some believed that the disease has already eliminated or no more COVID disease and thus, need of public health interventions such as vaccine was no needed. The analysis indicated lack of accurate perceptions and understanding of the COVID-19 disease in the community with wide spread disbelieving about existence of corona virus.

Perceptions of vulnerability of COVID-19

The study participants were asked that who contracts COVID-19 infection. Accordingly, 8273 (63.3%) of them reported all people and 1968 (15.1%), they don't know who are at risk to contract the virus. The study participants were also assessed their perception of who is at risk of severe COVID-19 infection. About 47.2% perceived that people aged 65 years old and older (elderly) were at risk of severe COVID-19 infection followed by 3033 (23.2%) of those who perceived all people were at risk of severe COVID-19 infection Table 8.11. The findings from the qualitative analysis indicated supportive findings in terms of perceived vulnerability to COVID-19 disease, as people with weak immunity, elders, people with chronic medical conditions, and urban residents were at higher risk of getting sick from the "so-called COVID-19 disease".

Table 8.11: Perception of study participants about COVID-19 infection, risk group, where to get vaccine and vaccination available in Ethiopia, 2023.

Perception of COVID-19	Frequency	Percent
Who contracts COVID-19 infection?		
All people	8273	63.3
Elders (people aged 65 and above)	2239	17.1
I don't know	1968	15.1
Children	952	7.3
Ad ults	916	7.0
Pregnant women	668	5.1
Female	577	4.4
Male	411	3.1
Others	190	1.5
Who is at risk of severe COVID-19 infection?		
People aged 65 years old and older (Elders)	6170	47.2
People with non-communicable diseases	2216	17.0
People with known respiratory diseases	1432	11.0
Male	211	1.6
Female	651	5.0
Children	2101	16.1
All people	3033	23.2
I don't know	2524	19.3
Other	308	2.4
Who are target for COVID vaccine?		
People aged 65 years old and older (Elders)	3560	27.2
People aged 12 years and above	2545	19.5
People with non-communicable diseases	765	5.9
People with known respiratory diseases	623	4.8
Male	344	2.6

	Female	671	5.1
	Children	916	7.0
	All people	4581	35.0
	I don't know	2865	21.9
	Other	488	3.7
COVID -19 can spread by an infected people without symptoms			
	Yes	5502	42.1
	No	7572	57.9
Total		13074	100

Regarding concern about COVID-19, large majority 10006(72.5%) of the respondents were either very concerned or moderately concerned during the pandemic and 2608(18.9%) of them were not concerned at all. While during the survey time, 2511(18.2%) were very or moderately concerned and the majority 81.8% had little or no concern at all Figure 8.5.

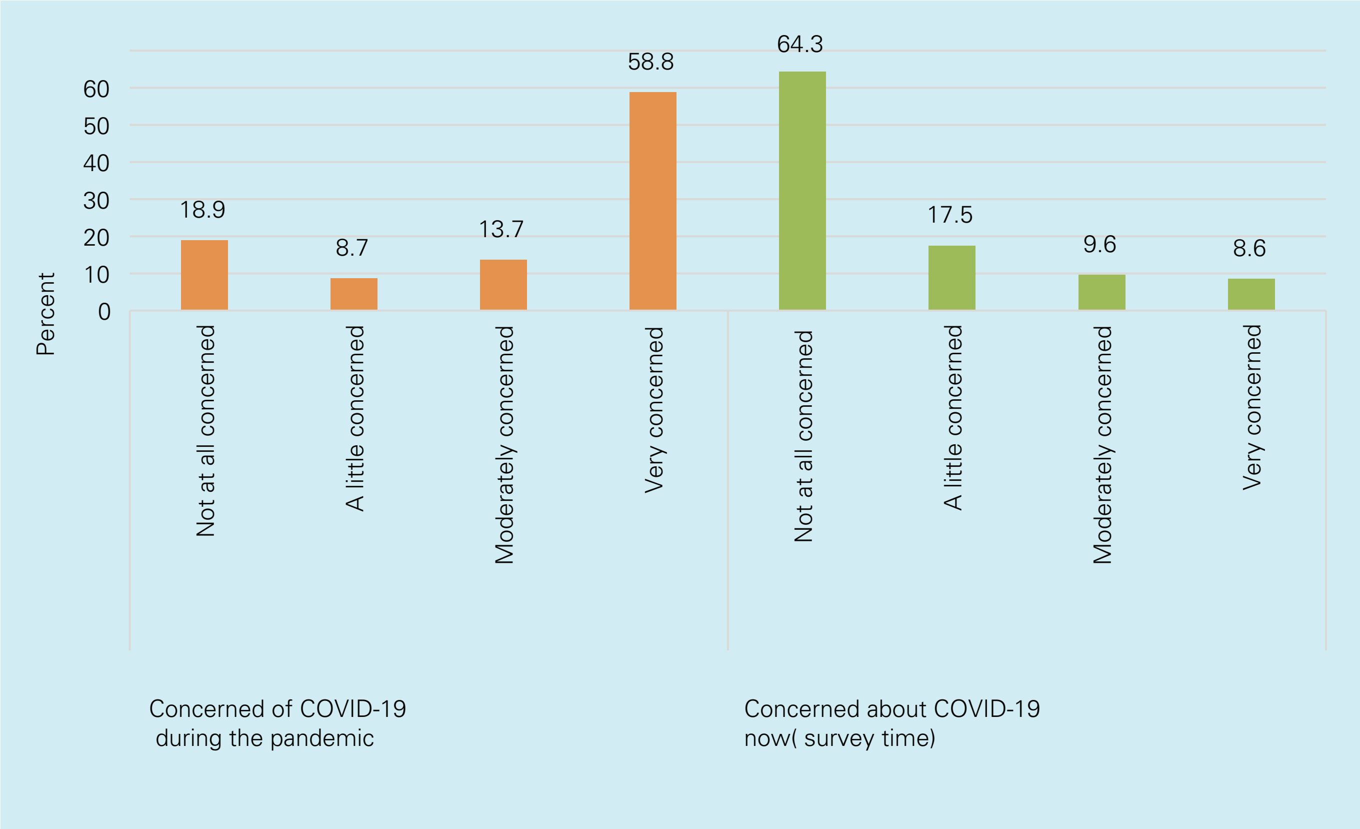

Figure 8.5: COVID-19 disease concern among study participants in Ethiopia, 2023

COVID-19 disease and vaccine awareness

Large majority of participants mentioned that Cough /sneezing /congestion /sore throat are symptoms of COVID-19 (78.1%) followed by fever (47.4%) and headache (29.7%). With regards to COVID-19 vaccine knowledge, slightly over one-thirds (4581 or 35.0%) of surveyed participants reported that all people were targets of COVID-19 vaccine and 3560(27.2%) mentioned that people aged 65 years and older (Elders) were the targets. The majority of participants knew that the recommended dose of COVID-19 vaccine was one dose (42.1%) and two doses (57.9%), with only tiny portion of them (52 or 4.0%) knew the recommended doses of COVID-19 as three or more Table 8.12.

Table 8.12: COVID-19 disease and vaccine awareness, and perception of vaccines safety and importance among study participants, Ethiopia, 2023

Variables	Frequency	Percent
Knowledge of COVID-19 sign and symptoms		
Cough / sneezing / congestion / sore throat	10210	78.1
Fever	6192	47.4
Headache	3887	29.7
Shortness of breath and difficulty breathing / chest pain	2951	22.6
Muscle aches and / orjoint pain /general body weakness	1106	8.5
Symptoms of GI (diarrhea, nausea, vomiting, abdominal pain)	782	6.0
Loss of smell and / or taste	454	3.5
I don't know	1850	14.2
Other	101	0.8
Knew where to get COVID-19 vaccine	9863	75.4
COVID-19 vaccination sites mentioned		
Health center	7181	72.8
Health post	2261	22.9
Hospital	3262	33.1
Outreach settings	33	0.3
Schools	154	1.6
Private clinics	2677	27.1
Pharmacy	370	3.8
Other	124	1.3
Recommended doses of COVID-19		
One dose	5502	42.1
Two dose	7572	57.9
Three and more dose	523	4.0
Unknown	3055	23.4
I don’t know	2612	20.0
Perceived importance of COVID-19 vaccine for health		
Not at all important	1393	10.1
A little important,	1671	12.1
Moderately important	3428	24.8
Very important	7320	53.0
Perceived safety of COVID-19 vaccine		
Not at all safe	1422	10.3
A little safe	2232	16.2
Moderately safe	4191	30.3
Very safe	5966	43.2
Total	13074	100

8.6 COVID-19 vaccine uptake

Regarding vaccine use, half 6969 (50.5%) of the survey respondents reported that their family member received COVID-19 vaccine. Regarding their willingness to get the COVID-19 vaccine, slightly over half, 7204 (52.2%) of them wanted to get the COVID-19 vaccine in the future.

More than two-fifths, 5817 (42.1%) of the participants thought that most people would receive the COVID-19 vaccine if it is recommended to them and 4256 (30.8%) of them were not sure about it. Also, nearly two-fifths, 5377 (38.9%) of them thought that most people they work or interact with would receive a COVID-19 vaccine.

With regards to supports related to COVID-19 vaccine, 5988 (43.4%) of study participants thought that most of their close family and friends wanted them to get a COVID-19 vaccine. Similarly, 6954(50.3%) thought that religious leaders want them to get vaccine and another 8977 (65.0%) thought that community leaders support them to get the vaccine. On the other hand, more than two-thirds, 9511 (68.9%) of participants reported that they didn't need permission to go and get the COVID-19 vaccine Table 8.13.

Table 8.13: Respondent perception about communities' acceptance of COVID-19 vaccines and supports in Ethiopia 2023

Variables	Response	Frequency	Percent
Most people I knew will receive COVID-19 vaccine if recommended to them	Yes	5817	42.1
	No	3739	27.1
	Not sure	4256	30.8
Most people I work or interact with will receive a COVID-19 vaccine	Yes	5377	38.9
	No	2591	18.8
	Not sure	4697	34.0
	Not currently working	1147	8.3
Most of my close family members and friends want me to get a COVID-19 vaccine	Yes	5988	43.4
	No	4199	30.4
	Not sure	3624	26.2
My religious leaders want me to get a COVID-19 vaccine	Yes	6954	50.3
	No	2800	20.3
	Not sure	4058	29.4
Other community leaders support me to get a COVID-19 vaccine	Yes	8977	65.0
	No	1811	13.1
	Not sure	3024	21.9
Healthcare worker recommended me to get a COVID-19 vaccine	Yes	11174	80.9
	No	2637	19.1
Needs permission to go and get COVID-19 vaccine	Yes	4301	31.1
	No	9511	68.9
Knew where to go and get COVID-19 vaccine	Yes	10385	75.2
	No	3427	24.8
Availability of COVID-19 vaccine at the nearest health facility or outreach sites	Yes	6875	49.8
	No	2850	20.6
	No sure	4087	29.6
Total		13812	100

With regards to factors for non-use of vaccine, the study participants mentioned that absence of vaccine 2263 (51.5%), fear of side effects 1262 (28.7%) and the vaccination site being hard to get 603 (13.7%) were among the reasons for not getting the COVID vaccine.

On the other hand, the qualitative data revealed that there were wide spread and deep rooted hesitations and concerns over the COVID-19 vaccine. Across the FGD and KIIs (community level) participants' categories there were consistent perceptions that the uptake and acceptance of COVID-19 vaccine is very weak,and people were unwilling to receive COVID-19 due to multiple hesitations and concerns over the

the vaccine. The vaccine was not acceptable and there were very strong and deep rooted native attitude and hesitations towards it. There were consistent reflections that these resistance and hesitations has been never changed overtime and still prevalent in the community. This implies that efforts should be in place to improve communities' awareness and acceptance of COVID-19 vaccine.

> *"Our community's attitude about COVID-19 vaccine is not changed yet; they dislike it very much. They believe that this vaccine may cause severe side effect and infertility. They say that these are newly introduced vaccinations Health extension worker.*

> *"People hide themselves when there is COVID-19 vaccination campaign. People are not willing to get vaccinated. So, concerned bodies should work to create community awareness about the benefit of vaccine"* Male FGD: P8

Another participant added that;

> *"There were big challenges in our woreda, even health professionals were attacked by the people… the community resists it [COVID-19 vaccine. A health worker who is providing the vaccine is told by the community to leave their kebele. The community did not want the vaccine [COVID-19 vaccine] as they believed it is against their religious value". EPI provider at health center.*

The major hesitations to COVID-19 vaccine were related to several factors including the communities' belief that: Vaccine contain microchips; vaccine is fake; it is not a vaccine rather it is the virus itself; Vaccine causes infertility, blood clot and has fatal side effects (kills people); Vaccine is related to an evil spirit (666) and Illuminati, and is against religious values; Vaccine is a political agenda; Vaccine is not well developed, immature or under experimentations; and Vaccine affects milk yield among lactating mothers.

> *"When they see us [providers] the people ran away. Communities are pre-informed and did not want to see us. They close their house, and did not want to say anything if you try to educate…-no one listens to you" EPI provider at health center*

> *"People think that this vaccine may exposed them to infertility and it is also an evil spirit (666). These are the reasons for not accepting the vaccine" Female FGD: P3)*

Access to COVID-19 vaccine and satisfaction with the service

Regarding easy access of COVID-19 vaccine, 4803 (34.8%) of study participants reported that getting a COVID-19 vaccine is very easy and 4616(44.4) of them reported as moderately easy. The main reasons for difficulty of accessing the vaccine were unavailability of the vaccine (51.5%) followed by fear of side effects (28.7%) and difficulty of easily reaching to the vaccination site (13.7%). More than two thirds (67.6%) were very satisfied and one in four (25.4%) moderately satisfied with COVID vaccine service Table 8.14.

Table 8.14: Access to COVID-19 vaccine and satisfaction with vaccination services among respondents Ethiopia 2023.

Variables	Response	Frequency	Percent
How easy is it to get a COVID-19 vaccine?	Very easy	4803	34.8
	Moderately easy	4616	33.4
	A little easy	2219	16.1
	Not at all easy	2174	15.7
	Total	13812	100
Reasons for not easily getting COVID-19 vaccine	Unavailability of vaccines	2263	51.5
	Fear of side effect	1262	28.7
	The vaccination site is hard to get to	603	13.7
	Longer waiting time	103	2.4
	I am unable to leave work duties	217	4.9
	Sometimes people are turned away without vaccination	119	2.7
	Cost of vaccination	26	0.6
	Others	796	18.1
	Total	4393	100
Satisfaction with COVID-19 vaccine service	Not at all satisfied	137	1.8
	A little satisfied,	344	4.5
	Moderately satisfied	1959	25.4
	Very satisfied	5217	67.6
	Total	7721	100
Reasons for not satisfied with the vaccination service	Vaccine is not available	278	57.8
	The vaccination site does not open on time	85	17.7
	Waiting times are long	46	9.6
	The vaccination site is not clean	19	4.1
	Providers are not respectful	31	6.4
	Provider do not spend enough time with people	43	9.0
	Other	82	17.0
	Total	481	100

Attitude towards COVID- 19 vaccination

Large majority of study participants agreed that COVID-19 vaccine is effective to control the infection (75.9%), the vaccine is safe (64.6%), use of face mask is effective in preventing COVID-19(79.6%), and use of sanitizers is effective to prevent COVID-19. Greater than two-thirds (68.5%) are willing to recommend the vaccine to others Table 8.15.

Table 8.15: Attitude toward COVID-19 vaccine and other prevention measures among Study participants in Ethiopia, 2023

Attitude statements	Response	Percent
COVID-19 vaccine is an effective way to control and prevent COVID infection	Agree	75.9
	Disagree	24.1
Vaccination decreases the risk of COVID-19 infection or its complications	Agree	75.9
	Disagree	24.1
COVID-19 is not serious a concern in my work or residential area	Agree	58.3
	Disagree	41.7
COVID-19 vaccines are safe	Agree	64.6
	Disagree	35.4
I believe the vaccine side effects are minimal and will not prevent me from receiving the vaccine	Agree	59.6
	Disagree	40.4
I am afraid that the vaccines might have unwanted side effects	Agree	33.4
	Disagree	66.6
The utilization of facemask is effective in preventing COVID-19	Agree	79.6
	Disagree	20.4
The utilization of sanitizers is effective in preventing COVID-19	Agree	78.5
	Disagree	21.5
I recommend others to take COVID-19 vaccinations	Agree	68.5
	Disagree	31.5

Greater than half of study participants have favorable attitude to COVID-19 vaccination in Southwest Ethiopia people (69.9%), Dire Dawa (60.5%), SNNP (58%), and Oromia (57.1%) regions. When coming to setting, higher proportion of adults in agrarian areas (52.3%) followed by urban (49.2%) had favorable attitude to the vaccine Table 8.16.

Table 8.16: Attitude toward COVID-19 vaccine among study participants by socio-demographic characteristics in Ethiopia, 2023

Variables	Response	Favorable attitude		Unfavorable attitude	
		Frequency	Percent	Frequency	Percent
Region	Addis Ababa	248	47.8	271	52.2
	Afar	101	36.6	174	63.4
	Amhara	1248	40.5	1835	59.5
	Benishangul Gumuz	81	49.4	83	50.6
	Dire Dawa	45	60.5	29	39.5
	Gambela	31	43.0	41	57.0
	Harari	16	44.4	20	55.6
	Oromia	3066	57.1	2307	42.9
	Sidama	246	40.0	368	60.0
	SNNP	1318	58.0	955	42.0
	Somali	214	24.2	671	75.8
	Southwest Ethiopia People	309	69.9	133	30.1
Setting	Urban	1633	49.2	1688	50.8
	Agrarian	4797	52.3	4376	47.7
	Pastoralist	492	37.4	825	62.6
Gender	Male	1915	55.3	1548	44.7
	Female	4962	48.4	5294	51.6
Age	12-19	413	49.0	429	51.0
	20-39	5471	50.6	5347	49.4
	40-59	959	48.8	1007	51.2
	>=60	80	43.1	106	56.9
Wealth Index	Lowest	1241	48.0	1346	52.0
	Second	1390	49.9	1395	50.1
	Middle	1402	49.0	1460	51.0
	Fourth	1478	51.5	1395	48.5
	Highest	1411	52.2	1293	47.8
Total		6923	50.1	6889	49.9

8.7 HPV infection and vaccine: Awareness, acceptance/attitude and hesitance/concerns

Perception and awareness related to HPV infection

Three quarters (74.9%) of adolescents didn't heard about HPV. The main source of HPV information for adolescents were health workers (72.6%) followed by teachers or school community (58.2%). With regards to adolescents' knowledge about HPV infection, large majority knew only females contracts the infection (84.9%). Tiny portion of adolescents replied that they don't know about who contracts the infection (7.7%).

In relation to HPV infection risk factors, slightly over half mentioned frequent sex partner exchange (52.9%) and regarding prevention modalities of the infection greater than two-thirds replied HPV vaccination (69.8%) Table 8.17.

Table 8.17: HPV infection information, sources of information and knowledge about the infection among adolescents in Ethiopia, 2023.

Variable	Frequency	Percent
Ever heard about HPV		
Yes	1342	25.1
No	3999	74.9
Total	5341	100
Sources of HPV information		
Health worker	974	72.6
Friends/peers	237	17.7
Relatives/parents	71	5.3
Radio	86	6.4
TV	106	7.9
Social media	60	4.5
Teachers/schools	782	58.2
Other specify	6	0.4
Total	1342	100
Who can contract HPV infection?		
Only male	8	0.6
Only female	1139	84.9
Both male and female	91	6.8
I don't know	103	7.7
Total	1342	100
HPV risk factors		
Frequent sex partner exchange	709	52.9
Skin to skin contact around the genital area	238	17.7
body fluids(blood)	152	11.3
Other, specify	6	0.4
Total	1342	100
Ways of preventing HPV infection/cervical cancer		
Vaccination	937	69.8
Practicing abstinence	484	36.1
By using condoms	180	13.4
Can't be prevented	15	1.1
Total	1342	100

Perceptions and awareness related to HPV vaccine

More than half of adolescents have heard about HPV vaccine (54.3%) and out of these, large majority of them heard the vaccine prevents cervical cancer (70.3%) and it is provided for girls (51.9%). The main source of HPV vaccine information were health workers (67.1%) followed by teachers/schools (58.4%).

Regarding adolescents' knowledge about HPV vaccine, large majority of adolescents mentioned that only women be target group of the vaccine (92.3%), the vaccine helps to prevent cervical cancer (85%), and ideal age to get the vaccine be 9-14 years (65%) Table 8.18.

Table 8.18: Perception and source of information relating to HPV vaccine

Variable	Frequency	Percent
Ever heard about HPV vaccination		
Yes	2899	54.3
No	2443	45.7
Total	5342	100
Information heard about the vaccine		
It prevents cervical cancer	2038	70.3
Provided for girls	1506	51.9
Other, specify	65	2.24
Total	2899	100
Source of information		
Health worker	1945	67.1
Teachers/schools	1694	58.4
Friends/peers	428	14.8
TV	231	8.0
Radio	131	4.5
Relatives/parents	106	3.6
Social media	62	2.1
Other specify	23	0.8
Total	2899	
Who gets the vaccine/eligible		
Only Women	2675	92.3
Men and women	77	2.7
Only Men	13	0.4
I don't know	133	4.6
Total	2898	100
HPV vaccine helps to prevent		
Cervical cancer	2464	85.0
Genital warts	210	7.26
Anal warts	60	2.08
Oropharyngeal cancer	23	0.79
Others specify	17	0.57
I don't know	407	14.04
Total	2899	100

Recommended doses of HPV vaccine		
Two doses	1407	48.5
One dose	488	16.8
I don't know	1004	34.6
Total	2899	100
Ideal/ recommended age to get HPV vaccine		
9–14 years	1897	65.4
Can be provided at any age	264	9.1
Less than 9-years	15	0.5
I don't know	722	24.9
Total	2898	100

Attitude, acceptance and beliefs towards HPV infection and vaccine

Large majority of study participants agreed that cervical cancer is a deadly disease (74.5%), and having only one sexual partner protect from HPV infection (59.6%). Regarding the vaccine, majority beliefs that vaccine side effects are minimal and don't prevent from taking (65%), HPV vaccine is safe to take (65.9%), and the vaccine save life and improve health (85.3%) Table 8.19.

Table 8.19: Attitude, acceptance and believe about HPV infection and vaccination among adolescents in Ethiopia, 2023

Attitude statements	Response	Percent
Cervical cancer is a deadly disease	Agree	74.5
	Disagree	25.5
HPV vaccine is effective in preventing cervical cancer	Agree	73.7
	Disagree	26.3
HPV vaccine is safe to take	Agree	65.9
	Disagree	34.1
I will take the vaccine because I feel at risk of getting HPV infection	Agree	52.1
	Disagree	47.9
Person who has only one sexual partner can protect himself/herself from HPV	Agree	59.6
	Disagree	40.4
It is not necessary to get HPV vaccination	Agree	20.2
	Disagree	79.8
I believe the vaccine side effects are minimal and will not prevent me from	Agree	65.1
	Disagree	34.9
I feel it is better to be vaccinated before becoming sexually active	Agree	84.0
	Disagree	16.0
More information on HPV and its vaccine will be needed before I take the vaccine	Agree	84.0
	Disagree	16.0
HPV vaccine may have long lasting negative effect on me such as infertility	Agree	21.1
	Disagree	78.9
I feel only sexually active girls should get the vaccine	Agree	26.2
	Disagree	73.8
My family allow me to get vaccinated/my parents letme to have vaccine	Agree	74.5
	Disagree	25.5
HPV vaccine saves life and improve health	Agree	85.3
	Disagree	14.7
I recommend others to take HPV vaccinations	Agree	78.6
	Disagree	21.4

Overall, more than half of study participants have favorable attitude to HPV vaccination. There is variation by sociodemographic characteristics and Region. The lowest in Afar (31.8%) and the highest in Harari 65.2%. When coming to setting, higher proportion of respondents from urban areas (58.8%) followed by agrarian (54.7%) had favorable attitude to the vaccine

Table 8.20: Attitude toward HPV vaccine among study participants by socio-demographic characteristics in Ethiopia, 2023

		Favorable attitude		Unfavorable attitude	
Variables	Response	Frequency	%	Frequency	%
Region	Addis Ababa	72	59.6	49	40.4
	Afar	13	31.8	28	68.2
	Amhara	436	54.5	364	45.5
	Benishangul Gumuz	28	63.3	17	36.7
	Dire Dawa	7	58.3	5	41.7
	Gambella	10	49.7	10	50.3
	Harari	4	65.2	2	34.8
	Oromia	649	57.6	478	42.4
	Sidama	125	56.5	96	43.5
	SNNP	80	53.1	71	46.9
	Somali	98	40.4	145	59.6
	Southwest Ethiopia People	65	58.7	46	41.3
Setting	Urban	487	58.8	341	41.2
	Agrarian	985	54.7	816	45.3
	Pastoralist	116	43.1	153	56.9
Ability to read and write	Yes	1543	55.2	1252	44.8
	No	46	43.9	58	56.1
Attended formal education	Yes	1536	55.6	1225	44.4
	No	52	37.9	85	62.1
Respondents Educational status	Primary education	1140	53.3	997	46.7
	Secondary education	396	63.4	228	36.6
Current school attendance	Yes	1318	57.0	996	43.0
	No	218	48.8	229	51.2
Religion	Muslim	696	56.9	528	43.1
	Orthodox	573	51.1	549	48.9
	Protestant	315	58.0	229	42.0
	Others	4	42.5	6	57.5
Relationship with household head	Wife	60	52.8	54	47.2
	Daughter	1318	55.0	1078	45.0
	Sister	86	48.6	91	51.4
	Other relative	105	56.9	79	43.1
	Other	20	69.5	9	30.5
Father Education	No formal education	719	50.7	699	49.3
	Primary education	511	57.5	377	42.5
	Secondary education	222	61.6	139	38.4
	College or university	87	57.8	63	42.2
	I don't know	49	60.5	32	39.5
Mother education	No formal education	1046	53.7	902	46.3
	Primary education	371	57.9	269	42.1

	Secondary education	109	60.8	70	39.2
	College or university	39	46.8	44	53.2
	I don't know	24	47.9	26	52.1
Father occupation	Farmer	1088	53.9	929	46.1
	Merchant	188	59.3	129	40.7
	Employed	208	52.7	187	47.3
	Daily laborer	13	61.5	8	38.5
	Others	62	57.8	45	42.2
Mother occupation	Housewife	1007	55.2	819	44.8
	Farmer	371	54.5	310	45.5
	Merchant	109	55.8	87	44.2
	Employed	72	49.5	73	50.5
	Daily laborer	6	80.1	2	19.9
	Others	14	74.6	5	25.4
Living with	Parents	1361	55	1107	45
	Relatives	180	52	166	48
	Husband	13	45	16	55
	Others	34	61	21	39
Wealth quantiles	Lowest	174	47	195	53
	Second	185	49	194	51
	Middle	264	55	217	45
	Fourth	292	56	228	44
	Highest	348	62	215	38
Total		1588	55	1311	45

HPV vaccine uptake, intention to use vaccine and barriers

Forty-six percent (46%) of adolescents have not been vaccinated for the HPV vaccine. This implies that an intensified effort is needed to increase vaccine uptake. Regarding future intention to get vaccinations, of the 2468 adolescents who didn't have the vaccination; 61.1 % of them are willing to take the vaccine in future.

The commonest reasons mentioned by adolescents for not taking HPV vaccination were lack of information about vaccination site (70.6 %) followed by service unavailability whenever needed (10.4%) and inaccessibility of service (9.1%) Figure 8.6.

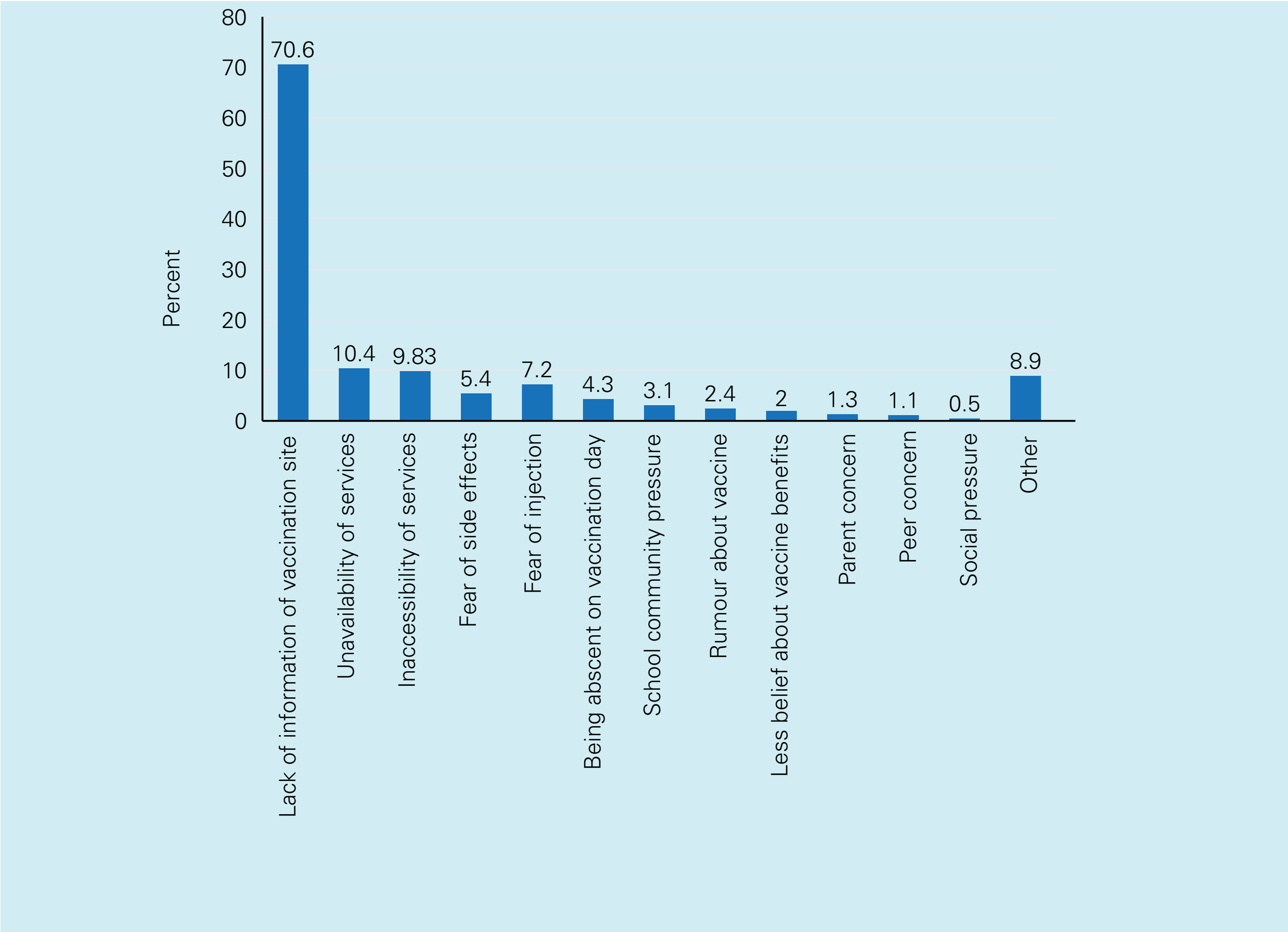

Figure 8.6: Reason for not taking HPV vaccination among adolescents in Ethiopia

The study has also revealed several concerns that impedes uptake of HPV vaccination in Ethiopia. The predominant concerns were related to needle phobia (35.2%), vaccine side effects (30.2%), vaccine safety (29.6%), and vaccine effectiveness (27.7%). Similarly, parental concern (21.7%), concern from teacher's/school community (16.9%) and concern from religious and community leaders (16.8%) were identified as barriers to immunization uptake Table 8.21.

Table 8.21: Concerns of HPV vaccination among adolescents in Ethiopia,2023

Concerns of HPV vaccine	To a Great extent	Somewhat	Not sure	Very little	Not at all
	n(%)	n(%)	n(%)	n(%)	n(%)
Concerns about possible side effects	668(12.5)	946(17.7)	1121(21.0)	664(12.4)	1942(36.4)
Concern about vaccine safety	612(11.5)	968(18.1)	1168(21.9)	712(13.3)	1881(35.2)
Concern about effectiveness of the vaccine	565(10.6)	914(17.1)	1266(23.7)	656(12.3)	1941(36.3)
Parental concern	449(8.4)	709(13.3)	1013(19.0)	637(11.9)	2532(47.4)
Teachers /school community concern	418(7.8)	487(9.1)	973(18.2)	612(11.5)	2850(53.4)
Concerns from religious and cultural leaders	371(7.0)	522(9.8)	1256(23.5)	568(10.6)	2624(49.1)
Peer concern	431(8.1)	721(13.5)	911(17.0)	680(12.7)	2599(48.7)
Needle phobia	804(15.0)	1076(20.2)	648(12.1)	806(15.1)	2008(37.6)

The qualitative finding also identified several barriers related to low uptake of HPV vaccine. The wrong communities' perception about the vaccine such as vaccine is used as contraception or birth control strategy, and vaccine causes infertility (has side effects) are cited as the main barriers to HPV vaccine uptake. Though there are some improvements in relation to communities' awareness over time, the perceptions indicated above were still the main barriers to uptake of the vaccine.

This perception and belief has resulted to significant resistances to HPV vaccine uptake and the health care providers are challenged to provide HPV vaccination to girls. The belief is related to the fact that vaccine is given to young girls only and not for boys, and the communities perceive that this may affect girls' future in relation to reproduction. The other barriers to HPV uptake are religion influence. Besides the communities, the challenges related to HPV vaccine uptake are also from the school communities; revealing that resistances are observed indifferently among all population groups.

In some areas, parents who vaccinated their children has faced influences from religious leaders and are asked to get into confessions. Similarly, the providers are asked to confess considering providing HPV vaccination as guilty practice. In most areas HPV and COVID vaccines are identified by the community as newly introduced vaccinations, and the community has bad beliefs to these vaccines by relating with the previous ones (child vaccinations). As a result of this perception, in some areas mothers showed refusals to vaccinate their children in routine EPI programs. Following the implementation of various communication and health education strategies, however, there were improvements in the acceptance of HPV vaccine among the different category of the community.

"Rumors are common; we are always in a fight; it is very common on HPV. The most rumors are like 'HPV is against fertility; why only teenager females are being vaccinated'. Misinformation is common, sometimes people may deliver message wrongly without understanding the main concept of messages" (KII, regional EPI Focal)

"Once upon a time we were in school in a certain kebele to provide HPV vaccine for school girls. When they [school community] see us, they closed the room. When we go home to home they [communities] closed their house; communities said they [providers] have brought to us something bad, that make infertile. Such belief about HPV still exists in the community. But in contrary to this there are peoples who strongly need it, even they knocked our office/health center and ask for vaccination in this year, there is different beliefs, one is positive whereas the other is negative" (KII, EPI provider at health center)

Motivators/enablers for receiving HPV

Encouragement from teachers or school community was the leading facilitator for taking HPV vaccination (53.5 %) followed by availability of service whenever needed (30%), encouragement from health worker and accessibility of the service (27.5%) Figure 8.7.

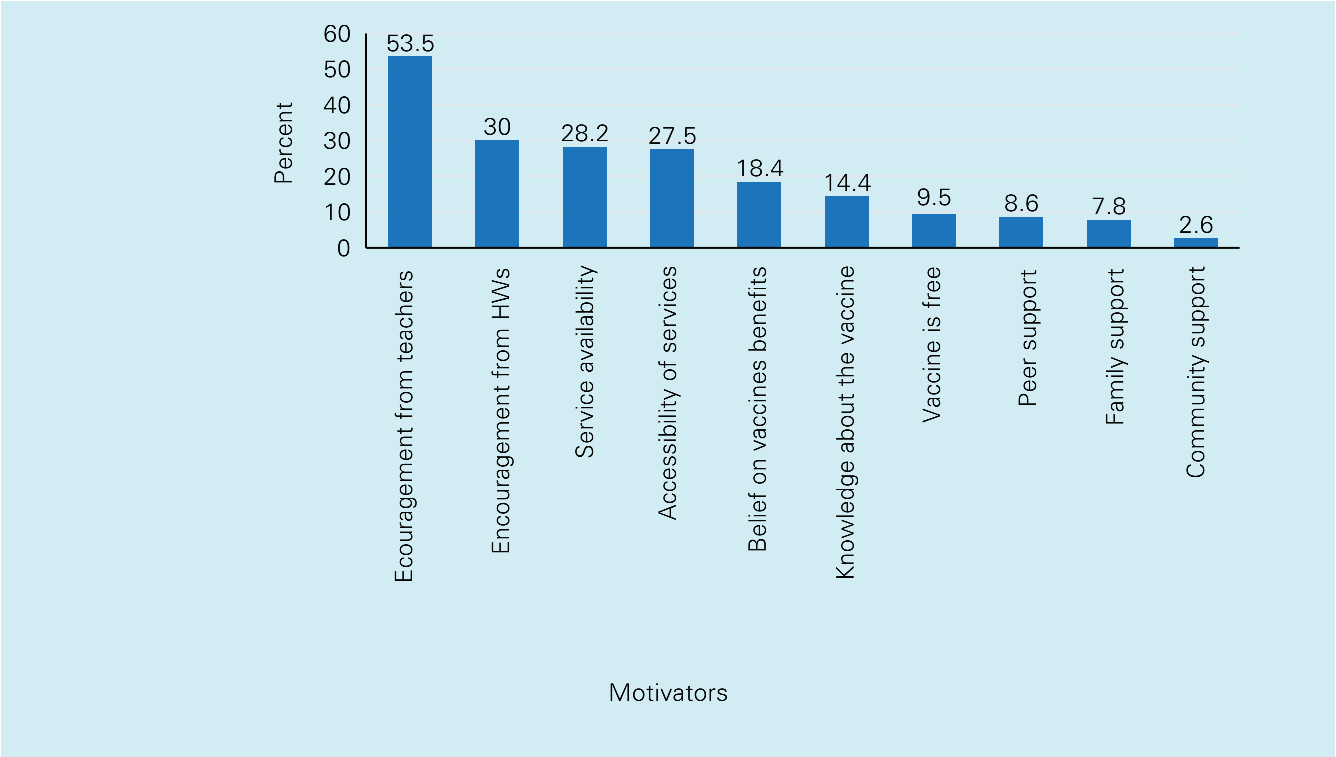

Figure 8.7: Motivators for uptake of HPV vaccination among adolescents in Ethiopia, 2023

The study result from qualitative data analysis identified almost similar finding that teachers had significant roles second to parents (mother) in HPV vaccination decisions. Commonly, girls consult mothers in such matters, and teachers influence school girls to take vaccinations. This might be the fact that girls feel comfortable when they communicate with their mother than father.

"Most of the time girls consult their mothers; however, teachers also encourage adolescent girls to take the vaccine. They say; if you refuse the vaccine you cannot attend class, teachers say like this. In this case, some girls take the vaccine in fear of their teachers" (Female FGD: P6)

In terms of HPV vaccination provisions, communities have preferred health facilities rather than schools. The reason mentioned was girls are ashamed of each other and fear to receive vaccines at schools partly due to the rumors disseminated in relation to the vaccine (i.e., HPV vaccine causes infertility).

HPV vaccine education strategies and practices

Various strategies are used to educate communities about HPV. Some of the strategies are health education by the HEWs, the development armies or groups (WDA and HAD), the community leaders, religious leaders, and health care providers. In health educations different education materials or aids are used such as banners, posters, leaflets, and radio. Usually, the HEWs primarily train the significant (influential) peoples within the community, and then this member of the community will educate and mobilize targeted people for vaccinations. The community and religious leaders are often engaged in situations of resistances in relation to vaccine uptakes. The HEWs provide HPV educations at health posts, home to home visits, and at schools. Working with schools has helped providers to effectively create demand for HPV vaccines. In addition, text messages or SMS are also used as source of information regarding HPV vaccination.

HPV vaccine resistance mitigation strategies: practices

Various strategies are used to mitigate challenges related to HPV vaccine acceptance, and uptake. The main strategy cited was EPI communication and education (or awareness creation). Awareness creation activities were implemented in various approaches and have involved different groups of the people, community structure, and various sector offices. The individuals engaged in communication and health education activities are HEWs themselves, the healthcare providers, community leaders, religious leaders, and elders. The HEWs has significant role in educating communities about HPV vaccine. From the sectors, schools, and women and child affair offices were participated. Religious leaders and influential community members are key to convincing and influencing the community. The community hears and accepts the messages disseminated by these people.

> *"The mitigation strategy is awareness creation, we work with community leaders, religious leaders, education office, women and child office to create awareness" EPI focal Regional level*

In addition, awareness creation activities are implemented at schools for girls in order to improve their knowledge about HPV and the vaccine. Trainings are provided to school community to enable the wider dissemination of HPV related messages. In the schools, Mini media clubs are used to disseminate messages about HPV vaccine. Schools are the most important sites to easily communicate HPV messages.

> *"In addition to community mobilization, we provide awareness training at the school level as girls are accessible. In schools with a Mini media club, we will aware students about the vaccine. The biggest work is done by health extension workers. The health extension workers are available in all kebeles, and engages in extensive awareness creation activities" (KII, zonal EPI focal)*

8.8 Socio-cultural factors and immunization services uptake

The national evaluation revealed that socio-cultural factors are determinants of vaccination uptake. As compared to the newly introduced vaccines, in general the communities have better awareness and good beliefs about routine immunizations with the exception of TD vaccination. This is because routine vaccines are delivered for several years, and much efforts are made to improve community awareness and attitudes. However, the study has also revealed that there are some challenges to routine vaccinations related to faith and misconceptions. For instance: in some areas there are influences from religious leaders not to vaccinate children before baptism ceremony (christening). Moreover, in some communities' child vaccination responsibilities are provided to mother and there is a belief that women/mother are supposed to stay at home at least for a month or two months.

These situations significantly resulted to non-vaccination of children, and dropouts once antigens are initiated. With regards to newly introduced vaccines (HPV and COVID-19), there are gaps in vaccine uptakes related to communities' perception and beliefs about the disease and vaccine.

- Community's belief and perceptions related to HPV vaccine and the disease: vaccine is used as form of contraception or birth control, and has side effects (causes infertility). Regarding the disease, communities' belief that girls are younger and will not be infected by the disease (cervical cancer).

- Community's belief and perceptions related to COVID-19 vaccines: There were huge misperception of COVID-19 disease. There was a strong belief that COVID-19 disease is "non-existent or fake or unreal disease", and created for political purpose. Regarding the vaccine, the communities' belief that it has side effects and causes infertility. They also associate the vaccine with evil spirit ("666"). These situations have significantly resulted to resistances to uptake of vaccinations.

8.9 IPC skills of health workers in immunization service delivery (GATHER approach)

Respondents' characteristics

A total of 1152 health workers responded to our questionnaires, about one-thirds were from Oromia region (29.3%), half were from the rural agrarian area (50.3%), 54.3% were EPI service providers and majority of them being females (70.2%) Table 8.22

Table 8.22: Demographic characteristics of IPC Skill assessment respondents , Ethiopia, 2023

	Response category	Frequency	Percent
Region	Afar	49	4.3
	Amhara	36	3.1
	Benishangul Gumuz	180	15.6
	Gambella	48	4.2
	Harari	76	6.6
	Oromia	337	29.3
	Sidama	65	5.6
	SNNP	114	9.9
	Somali	84	7.3
	Southwest	65	5.6
	Addis Ababa	59	5.1
	Dire Dawa	39	3.4
Location	Urban	464	40.3
	Rural Agrarian	578	50.3
	Rural Pastoralist	108	9.4
	Total	1150	100
Facility type	Health post	382	33.2
	Health center	626	54.3
	Hospital	144	12.5
Sex	Male	310	26.9
	Female	809	70.2
	Total	1119	100
Age	Age 20-29 years	585	50.8
	Age 30-39 years	425	36.9
	Age 40-49 years	36	3.1
	Age 50-59 years	12	1.0
	Total	1058	100
Current position in EPI unit	Provider	679	62.1
	Focal	338	30.9
	Head	76	7.0
	Total	1093	100
National		1152	100

Large majority of surveyed health workers 783(68%) received EPI focused training. Among these, 419 (37.7%) and 364 (32.8%) health workers received the training in the last two years and a year before the study is conducted. The rest 328 (29.5%) responded they did not receive training focusing on EPI.

Regarding supportive supervision, more than three-fourth (82.9%) received EPI focused supportive supervision among which 264 (23.4%) received it in the last one year before the study Figure 8.8.

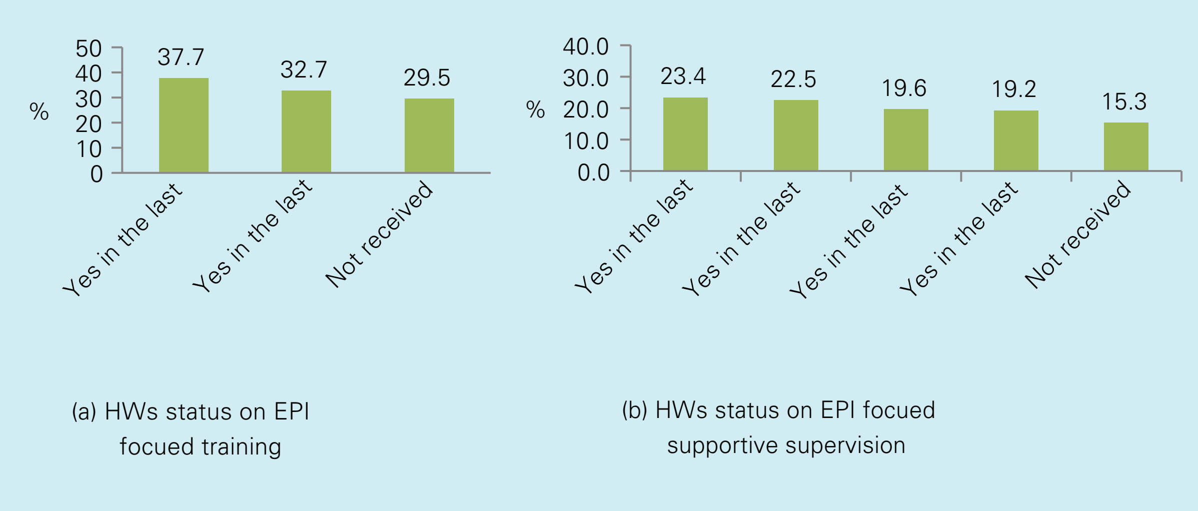

Figure 8.8: Proportion of health workers who received EPI focused training (a) and supportive supervision (b), 2023, Ethiopia

Woreda Health Office and Primary Health Care Units are among the leading health organizations to provide supportive supervision to health workers, as responded by study participants 336 (38%) and 309 (35%) respectively. Though the responses are few, health workers mentioned they have received supportive supervision from Zonal health departments (13.7%), Regional health bureaus (7.6%), Ministry of health (3.4%), and Partners/NGOs (2.4%).

IPC process and skills of health workers

Large majority of health workers responded that they communicate key immunization messages more often, including type of vaccine being given during the visit (83.5%); the possible side effects of the vaccine and what to do (84.0%); to safeguard and return with the health card (89.5%); and the date and time for the child's next doses (90.3%). However, findings of the observation session revealed a lower practice of IPC activities by health workers when compared with responses of the self-report. Hence, less than half health workers provide information on type of antigen (41.0%); purpose of each antigen (35.5%); and potential adverse effects of antigens with remedial actions (36.7%). Large majority of health workers (84%), and slightly more than half of them (53.6%) were observed while providing information on when will be the next appointment date and the proper handling of the immunization card, respectively.

Nearly six in ten of respondents (58.6%) use relevant supporting materials while explaining to caregivers more frequently. Showing respect by listening attentively (86.4%); giving credible and evidence-based vaccine and disease information (84.4%) and responding to caregivers' questions and concerns truthfully (84.6%) were among the IPC skills practiced more often by health workers. Asking caregivers to repeat what they need to do (63.9%) and using open-ended questions to seek more information about concerns or practices (61.6%) were IPC skills practiced less frequently by health workers. Table 8.23 describes IPC practice of health workers based on selected items following the GATHER (Greet, Ask, Tell, Help, Explain, and Recommend) approach.

Table 8.23: IPC process and skills practiced by health workers during immunization service delivery following GATHER approach in Ethiopia, 2023

IPC: Activities	Never<br>Frequency (%)	Seldom<br>Frequency (%)	Often<br>Frequency (%)
Welcome the caregiver	22 (1.9)	107 (9.4)	1014 (88.7)
Sincerely praise caregivers for bringing their babies for immunization	39 (3.4)	134 (11.7)	974 (84.9)
Ask the appropriate question about routine immunization	33 (2.9)	246 (21.5)	865 (75.6)
Use relevant supporting materials, including health card, to explain to caregivers	70 (6.1)	404 (35.3)	672 (58.6)
The vaccines being given during the visit	23 (2.0)	167 (14.5)	960 (83.5)
The possible side effects and what to do	27 (2.4)	156 (13.6)	963 (84.0)
To safeguard and return with the health card	25 (2.2)	96 (8.3)	1029 (89.5)
The day and time for the child's next doses	26 (2.3)	85 (7.4)	1036 (90.3)
Ask caregivers for any immunization questions or concerns they may have	25 (2.2)	287(25.0)	838 (72.9)
Respond truthfully, understandingly, and reassuringly to caregivers' questions and concerns	24 (2.1)	155 (13.5)	971 (84.4)
Ask caregivers to repeat what they need to do	39 (3.4)	376 (32.7)	735 (63.9)
Summarize key information, including the key immunization messages	57 (6.1)	204 (21.8)	675 (72.1)
Show respect by listening attentively	39 (3.4)	117 (10.2)	993 (86.4)
Give credible, evidence-based vaccine and disease information	31 (2.7)	144 (12.5)	973 (84.8)
Reflect back caregiver statements and feelings to show or check understanding and encourage dialogue	36 (3.1)	339 (29.5)	774 (67.4)
Use open-ended questions to seek more information about concerns or practices as needed	55 (4.8)	386 (33.6)	707 (61.6)
Discuss and try to correct caregivers' immunization misconceptions or rumors	42 (3.7)	190 (16.6)	915 (79.8)

Other IPC activities

There are other additional activities expected to be performed by health workers to have an effective IPC during immunization service delivery. Health workers reported that they help caregivers overcome obstacles to complete and timely immunization (73.8%) and follow with caregivers who had questions or concerns more often (69.9%) Table 8.24. Additionally, health workers practice sharing successful stories with their colleagues more frequently (69.4%). On the other hand, respondents self-report showed that

identification of useful materials and visual aids (50.4%); execution of community meetings (50.5%); and implementation of home visits (44.4%) were among the least practiced IPC activities of EPI providers frequently.

Table 8.24: Other IPC activities practiced by health workers during immunization service delivery in Ethiopia, 2023

Other IPC activities	Never Frequency (%)	Seldom Frequency (%)	Often Frequency (%)
Identify or create useful materials and visual aids	109 (9.6)	453 (39.9)	572 (50.4)
Conduct community meetings	113 (9.9)	523 (45.8)	507 (44.4)
Conduct home visits	127 (11.1)	439 (38.4)	577 (50.5)
Reach out to community leaders and encourage them to support immunization	93 (8.1)	260 (31.5)	690 (60.4)
Follow up with caregivers who had questionsor concerns	71 (6.2)	342 (29.9)	730 (63.9)
Follow up with caregivers whose children have missed a scheduled immunization	59 (5.2)	289 (25.3)	796 (69.6)
Help caregivers overcome obstacles to complete and timely immunization	47 (4.1)	252 (22.1)	842 (73.8)
Problem solving alone	123 (10.8)	393 (34.6)	621 (54.6)
Problem solving with colleagues	59 (5.2)	329 (29.0)	746 (65.8)
Seek the guidance of a supervisor to overcome a challenge	75 (6.6)	451 (39.6)	613 (53.8)
Share successful practices with colleagues	58 (5.1)	288 (25.3)	791 (69.4)

Variations of IPC practice

Larger mean score of IPC practice was observed in Amhara, Benishangul Gumuz, Harari, Oromia, Sidama, SNNP, and Southwest Ethiopia Peoples regions compared with the rest regions included in the study Table 8.25. Whereas, the lowest mean score of overall IPC practice was observed in Afar region (47.1) followed by Gambella region (52.1). In terms of IPC categories, IPC process activities have the largest mean score than IPC skill practice and other IPC activities (85.3 and 72.9, respectively). Overall, the mean score of IPC practice for all categories of IPC in all regions was found to be 59.2 (± 12.9).

Table 8.25: Mean score of IPC practice among health workers proving Immunization service by region, 2024

Region	IPC Process		IPC Skill		Other IPC activities		Overall IPC practice	
	Mean	± SD	Mean	± SD	Mean	± SD	Mean	± SD
Afar	73.6	23.30	65.78	27.47	56.99	26.71	47.1	18.8
Amhara	87.8	11.66	89.26	11.52	77.54	12.58	61.9	8.3
Benishangul Gumuz	87.97	14.97	87.22	15.08	72.31	19.27	60.4	12.1
Gambella	80.83	26.92	76.52	28.19	63.74	23.92	52.1	21.1
Harari	91.00	8.22	86.84	11.06	74.31	18.24	61.2	7.8
Oromia	87.82	12.36	88.44	12.36	74.77	18.93	61.0	9.8
Sidama	90.96	11.65	84.97	17.59	73.36	24.82	61.2	11.7
SNNP	90.47	14.13	85.09	18.47	76.18	19.65	61.2	12.6
Somali	80.37	21.70	79.96	20.24	73.91	22.16	56.0	14.5
Southwest Ethiopia Peoples	92.19	8.07	88.06	12.83	73.98	21.39	62.3	8.6
Addis Ababa	87.68	18.31	87.68	17.26	71.10	19.36	59.7	12.5
Dire Dawa	84.87	14.06	83.33	14.33	72.91	21.10	58.2	10.9
Overall (n = 1152)	87.02	16.39	85.28	17.17	72.97	20.64	59.2	12.9

Similarly, variation of IPC practice was observed for other factors including location (place of residence) and facility type. The mean score of IPC practice (overall) and all of it sub-categories (IPC process, IPC skill, and other IPC activities) varied by location (place of residence) and facility type. The mean scores for IPC process and skill were relatively high across the place of residence (location), which lies between 76.5 and 89.0, and 71.4 and 87.3, respectively. Whereas, the mean score for other IPC activities was low across the place of residence (location) - lies between 67.5 and 69.9. The average mean score for IPC practice (overall) along with its sub-categories was high among residents of the rural agrarian followed by urban and rural pastoralists. Likewise, the mean score of IPC process and skill were high across the different facility types ranging from 85.8 and 87.8 and 83.7 to 85.6, respectively. On the other hand, the mean score for other IPC practice was found to be low across facility types (65.8 to 72.3). Health posts were facility types where high mean scores were observed for IPC practice (overall) and all sub-categories of IPCs; whereas low mean scores for IPC practice were observed in hospitals Figure 8.9.

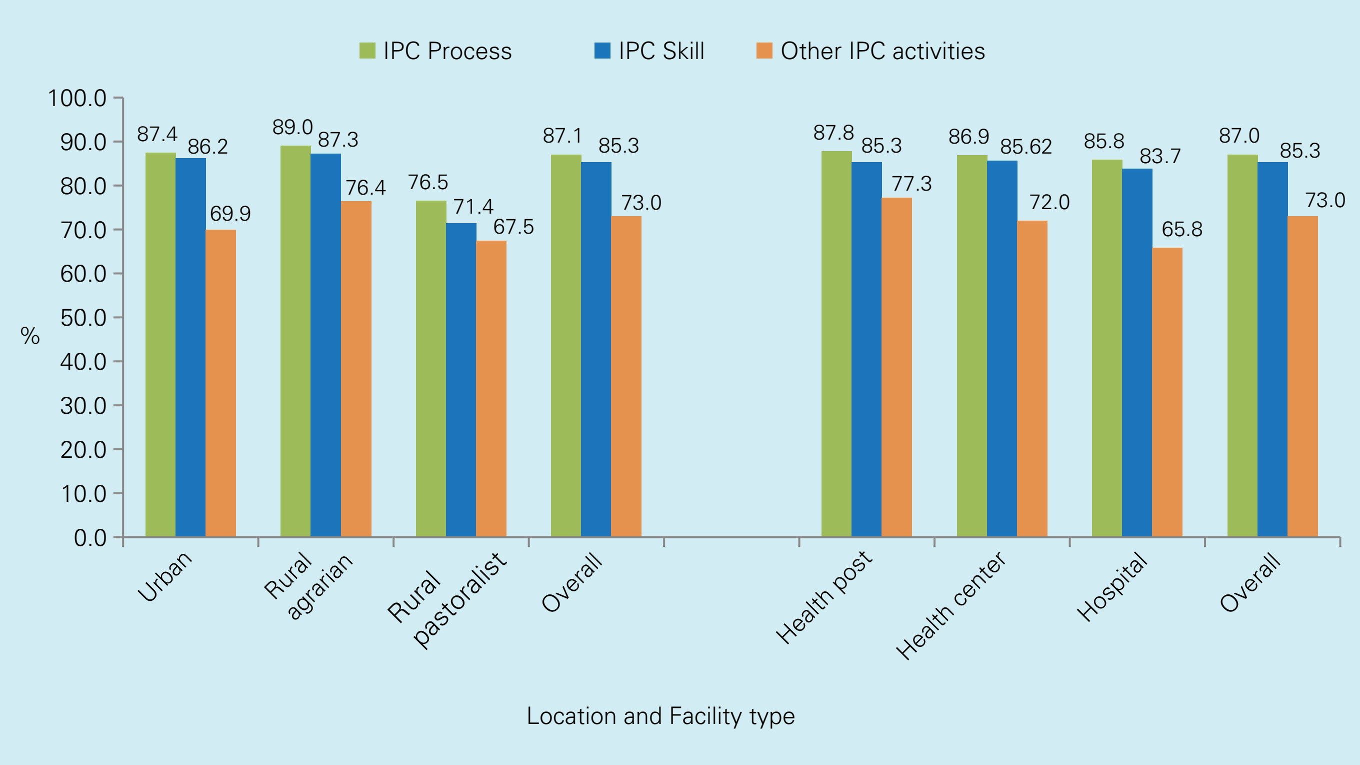

Figure 8.9: IPC practice of health workers in Immunization service delivery by location (place of residence) and facility type, Ethiopia, 2023

Variation in IPC practice was also observed among health workers with different positions in the EPI Unit of the assessed facilities. Hence, high mean score for IPC practice (overall) and all sub-categories was observed among facility heads (89.2 – IPC process; 87.5 – IPC skill; and 79.1 – other IPC activities) when compared with focal persons and service providers with no positions. Similarly, health workers with different status on EPI focused training and supportive supervision had different mean scores for IPC practices, though the magnitude of the variation was not high. With this regard, mean score for IPC process and skill was high across health workers regardless of their EPI focused training when compared with their practice on other IPC activities, which was low. However, mean score of other IPC activities was found to be high among health workers who didn't receive EPI focused training (74.1) when compared with those who received EPI focused training in the last two years and one year before the study was conducted (73.2 and 72.3, respectively). Likewise, mean scores of IPC practice (overall) and all its categories was high across health workers status on getting EPI focused supportive supervision Figure 8.10.

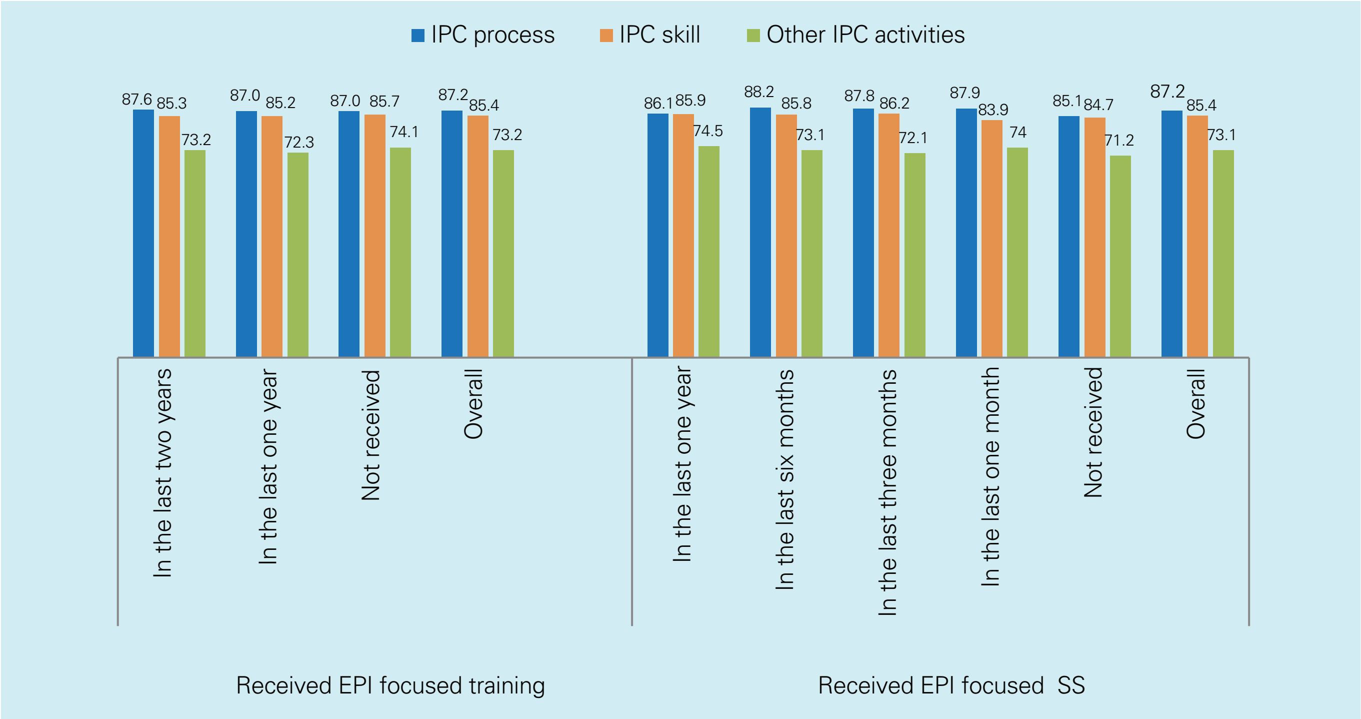

Figure 8.10: IPC practice of health workers in Immunization service delivery by status on getting EPI focused training and supportive supervision, Ethiopia, 2023

Barriers to effectively practicing IPC

Majority of health workers agreed that work overload leading to stress and fatigue (66.9%); shortage of staff at the EPI unit (60.2%); and lack /insufficient training in communication skills (59.7%) were among the barriers to practicing IPC. While nearly half of them agreed with absence of interpersonal communication job aids (52.7%) and lack of supervisions /mentorships focused on interpersonal communication (52%) as barriers that hinder them not to practice IPC effectively Table 8.26.

Table 8.26: Barriers to practicing good IPC in immunization service delivery as reported by health workers in Ethiopia, 2023

Barriers	Response	Frequency	Percent
Lack of subject knowledge required for fully explaining the modalities of diagnosis, treatment options, or prognosis	Agree	613	53.2
	Disagree	539	46.8
Long working hours/workload (leading to stress and fatigue)	Agree	771	66.9
	Disagree	381	33.1
Shortage of staffs at the EPI unit	Agree	694	60.2
	Disagree	458	39.8
Infrastructural deficits like lack of proper place for discussion, overcrowding	Agree	665	57.7
	Disagree	487	42.3
Lack /insufficient training in communication skills	Agree	688	59.7
	Disagree	464	40.3
I never felt that good communication is that much important in EPI service provision	Agree	557	48.4
	Disagree	595	51.6
Absence of interpersonal communication job aids	Agree	608	52.8
	Disagree	544	47.2
Lack of supervisions /mentorships focused on interpersonal communication	Agree	600	52.1
	Disagree	552	47.9
Difficulty in understandng the patient's language	Agree	454	39.4
	Disagree	698	60.6
Clients/caregivers usually don't want to stay longer after taking vaccination services	Agree	337	34.2
	Disagree	649	65.8

8.10 EPI Advocacy, Communication and Social Mobilization (ACSM)

ACSM Practices and Strategies

The assessment revealed that various strategies of EPI demand generations have been implemented in Ethiopia. Often a combination of setting and platforms were employed. Summary of commonly used EPI communication and education settings and platforms for demand generation were presented Table 8.27.

Table 8.27: EPI communication or education settings/platforms and participants in Ethiopia, 2023

EPI communication and education settings and platforms	Participants of EPI communications or education
Public gatherings: Social gatherings/Idir, community day, community forum/meetings, community dialogue/discussion	Women group or networks<br>Community groups/Community structure<br>Community educators'/community volunteers
Religious congregations/Religious institutions	Community PSA/ Criers
Schools	Model mother's/ model women
Women day	Health extension workers
Vaccine week	Health professionals
Women forums	Male health workers
Women conference	Community leaders/influential community
Ceremonial activities	leaders/local leaders/Gare leaders
House to house/home to home	Religious leaders
Outreach	Communication taskforce at MOH
Refugee camp	Community mobilization committee
Health facilities	Civil societies/ Charities
Mass media advocacy	Caregivers and families
Media spot messages	Politicians/district cabinets
Multimedia	Government sectors: Women and Child Affairs
Printed materials distribution	Public relations in EPI advocacy
Social media: Facebook; WhatsApp	Partners or NGOs

The different community structures (such as women development army or networks) were universally used during community mobilization for immunization services and EPI communications across regions. Similarly, local leaders (community and religious leaders) were engaged in similar activities. Usually the health professionals were engaged in EPI communications at health facilities, and the HEWs plays huge role in demand generation activities at community level. In some regions (Southwest Ethiopia region) the communities believe that health professionals were more respectful and informative. In some communities' (such as socially marginalized communities and communities located in hard to reach areas) the health professionals were engaged in EPI communication or educations at the community level than the HEWs.

"There are marginalized population (so called gossa) in the woreda: mana people and manjo gossa. These people are living in the hard to reach area and there is social stigma to reach this people" (KII, Woreda EPI focal)

In EPI communications, several strategies such as use of model mothers (mothers whose child has completed vaccinations), families with incidents of vaccine preventable diseases (VPDs), and communities with misconceptions of immunizations were employed. In the health facilities, EPI educations were given during morning sessions and by integrating with other services such as ANC.

'To increase community awareness, health education is given to all patients and clients coming to the health center (MCH, OPD). Health education is given regularly, and immunization is given as one topic. In addition, pregnant women conference is conducted every year in each kebele' (KII, Facility EPI focal)

The participants from federal level reported that advocacy, community mobilization, engagement, and conversation were used to create demand for EPI. In addition; media spots, live broadcasts, and social media were used to promote immunizations. The ministry of health has a dedicated department for this called National Health Education and Promotion, and communication team was one member of the Technical Working Group.

> *'The communication team is the sub-team of the Technical working group. This team is working proactively. We do advocacy activities before providing the vaccine. We use different formal and informal community platforms. We use trust messengers in the community. For example, religious and cultural leaders, religious unions. In regions we have ambassadors; we use them. We use civic societies, and regional development agencies'* (KII, MoH)

On the other hand, the regional level participants have mentioned that the following strategies were used for EPI communication and demand generation in their regions: public relation department to organize advocacy campaigns; religious leaders and other influential persons to deliver EPI messages; media for EPI advocacy, various event to communicate EPI (e.g., Africa vaccination week, public events) and SBCC Technical Working Groups and working with partners in EPI communications (WHO, UNICEF etc.). EPI demand generation and communication activities are commonly done for non-routine immunizations, and for routine it was conducted rarely.

8.11 Community structures for immunization demand creation

Demand creation for immunization services is more effective when addressed in the overall context of health system strengthening. The communities engage and plays crucial roles in the planning, implementing and monitoring of immunization program. In the present national assessment, the health facility heads were asked about the practices of demand assessments prior to immunization program planning, and whether the facilities coordinate with the local authorities and leaders (community & religious leaders) in EPI demand generations. The result showed that 64.7% of health facilities (including health centers and hospitals), and 55.4% of health posts conduct demand assessments before EPI planning. Regarding coordination's, 56.9% of the health facilities and 69.3% of health posts coordinates with the local authorities and leaders to implement immunization programs. Though demand assessment and coordination with local community structures are happening in the health facilities, the practice is absent in significant proportion of them. The qualitative finding also showed that the community structure and networks (such as women development army) involvement in EPI activities is getting weak in the recent years. The women health development army (HDAs) play vital role in EPI program. They assist the HEWs by coordinating and facilitating community meetings, provide education to members and communities, serve as liaison between communities and the HEWs (health system), participate as mobilizers of community and motivators of parents for vaccination, trace defaulters, remind members/neighbors on vaccination schedules, and notify vaccination side effects and any disease outbreak within the communities. However, the FGD participants and HEWs concerned that recently less attention has been given to the community networks and their role has been dropping or getting weaker.

1 for 5 or 1 for 30 platforms (WDA) were there and has taken trainings at Robit and Tikl Dingay. They have close relation with the HEW and used to communicate with HEW. The communication and integration of WDA with HEW was very close and working together for the vaccination services promotion and utilization in the community. But currently, in the previous 4 and 5 years, there is no any WDA organization and structure in this community. There is no anybody to communicate them and they are not functioning as WDA and they forgot their activities. There is no communication and mission in the woreda and region. The WDA do not conduct a meeting currently and their functions are not clear. No one supervise, monitor and mentor them to do their function in the community. Male FGD: P8

8.12 Challenges in EPI advocacy, communications and social mobilization (ACSM)

The EPI evaluation has revealed the following challenges of EPI demand generation.

A. Communication messages and materials are not adequate and insensitive to the local languages and contexts

There were concerns regarding the sensitiveness of the EPI communications messages and materials such as posters, and brochures across many settings especially in regions such as Gambella, Somali and Benishangul Gumuz. Frontline health workers and some participants in FGDs expressed their some IEC materials are not according to the local culture and context, and as a result people often do not identify themselves with it. In many areas HEWs explained that there were no adequate IEC materials (especially with illustrations) on EPI to be used for community education.

'We don't have enough pictures. I have one which I use to teach them. I don't have pictures to be given to the community. I don't have pictures to be given to each person. It is not adequate. The radio especially has all the information about vaccines. Also, the picture or leaflet we give them does have information about child vaccination' (KII, HEW)

In some regions and areas, language barriers hindered effective communication and interactions between clients and health workers. This barrier reported in many settings including Addis Ababa.

'There is also a language barrier to communicate with health providers and we fear approaching health providers. Had health centers been near our locality the community members would have been happy to visit health centers'. Female FGD: P5x

Moreover, IEC materials and messages do not have exact word translation into local languages for easy communications and understandings, and BCC is not well coordinated (is fragmented).

'Language barriers or absence of exact word in local language to educate the community is challenge to EPI communication' (KII, regional EPI focal)

B. IEC budget and resources - opportunity based IEC

There is resource and budget gaps for ACSM activities, especially at the lower level of the health care system, particularly facility and community engagements are without resources. Due to shortage or absence of budget for ACSM specially at lower levels, the communication activities often forced to wait and rely on opportunities, instead of having purposively planned and organized events in this own plan. These resource constraints led to concern that, BCC without resource is impossible and ineffective. The ACSM activities without adequate budget consideration affected mass media engagement in EPI in terms of buying airtime for public service announcements and covering other related costs. Though demand

generation activities requires resource, there was no budget dedicated for such activities.

> *'A significant challenge is the lack of budget allocated to organize community mobilization and educational events. In most cases, we rely on opportune moments to deliver our demand-generation message through meetings or events organized by other offices that have allocated budgets for such services' (EPI focal Woreda Level)*

C. Community engagement and mobilizations challenges - community network is getting weaker

ACSM activities are encountering some challenges related to community engagement, and mobilization. The key challenges as reported by HEWs and lower level key informants (district and facility) included:

- Inadequate human resources at health post level: In many settings HEWs existing at HP are not sufficient enough to engage communities in EPI, especially in highly populated and dispersed population involving remote areas. The HEWs emphasized that it is difficult to them to engage communities in EPI as they are few in numbers, in relation to the geographic area and the population size in their village.

- Lack of incentives for community volunteers: There was consistent concern among participated HEWs that community volunteers are vital in community mobilization and engagement is not motivated due to lack of incentives.

- Less acceptance of community volunteers: Community volunteers are not well accepted by some community members. According to some HEWs, some community members perceive that those volunteers are for personal benefit and then, less cooperate with them.

- Engaging marginalized communities: According to the key informants in some regions especially Southwest Ethiopia peoples' region, engaging and mobilizing marginalized and remote communities in EPI program involves multiple challenges and the key challenges are social stigma towards those community members by external people or outsiders including health workers. Importantly, certain community groups are believed to bad to the wellbeing of broader community and as result, community members do notallow mixing of their children with the stigmatized groups. Moreover, community health workers do not feel comfortable to work in and with such margined and stigmatized community groups.

- Community hesitation and misperceptions towards vaccine: Even though the vast majority of the community members (as reflected in FGDs and KIIs) have positive attitude towards vaccine (especially for the childhood vaccine), there were few people in the community who still tended to believe that vaccine is harmful or not useful. It was mentioned that those categories of people do resist vaccine uptake and also propagate misinformation or rumors that potentially negatively influence other people in the community.

- Lack of transportations and distance services and opportunity for HEWs: HEWs greatly concerned that distance and lack of transportations are key challenges to them to effectively travel in the community to undertake community mobilization and education activities.

- Community network such as women development army (WDA) are getting weak: The community networks, especially the women health development army(HDAs) are found to play vital role in community engagement in EPI program, playing roles such as assist HEWs,

coordinate and facilitate community meetings, provide education to members and communities, serve as liaison between communities and the HEWs (health system), trace defaulters, mobilizers and motivators of parents for vaccination, remind members/neighbors on vaccination schedule, and notify vaccine side effects and any disease outbreak. However, participants in FGDs and HEWs concerned that recently less attention has been given to such community networks and their role has been dropping or getting weaker
.

D. HEWs/HEP system gets weaker

There was a concern that HEW program functionality has been getting weaker or not vibrant as before in many localities in connection with several factors such insecurity situations, which led to weaken education and community engagement services as a whole.

E. A growing perception of no more demand creation is needed - demand creation is a finished agenda for routine vaccine

Generally, study participants agreed that there are very good awareness and acceptance of many immunization services, especially the childhood vaccination or routine vaccination services. Some HWs ascertained that no more demand creation effort is needed because they perceived that demand has been already created and further effort is not necessary, perceived demand generation is a finished agenda. According to them, what is most important is that, it is more important to respond to the created demand rather generating more demands which are difficult to satisfy it. This could lead to dropping of demand creation ACSM activities, thereby sudden dropping of community acceptance of vaccine.

F. Inadequacy of behavioral change enablers

HEWs concerned that community members. are well aware of the vaccine benefits and they often request it. However, lack of consistent service availability or delayed supply of vaccine at the hospital level disappoints community members who have interest to receive the services and also affect future demands and trust.

G. Weak digital communication and application for EPI ACSM

Few participants reported that digital technologies are less utilized for EPI communication and advocacy, including use of app for default tracing.

H. Health education and counseling at HFs are not considered as routine service

Many participants including HEWs and participated community members reported that health education activities are given at health facilities only occasionally when health workers are not busy, and when client flow and load is low. Likewise, client counseling during vaccination sessions such as communication of the five key messages are not consistent at facilities. When they [HEWs] gets busy, ignores or skip health education activities and counseling.

I. Motivators of immunization such as offering certificate of completion (motivational cues) stopped

8.13 Comprehensive ACSM suggested strategies to improve vaccine uptake

To address various behavioral, perceptual, and social factors that could affect the uptake of the vaccine, especially routine immunization program, participants provided several ACSM solutions for consideration in the future. Table 8.28 provides summary of finding of these suggestions.

Table 8.28: Comprehensive suggestions to improve to uptake of vaccine: ACSM

Suggested ACSM strategies for EPI	Narratives and illustrative quotes
Engage schools in EPI communication-school based EPI SBCC	The use of school platforms such as school clubs, school mini-media, peer education (e.g. For HPV uptake) was suggested to be a strong and effective approach for vaccine demand creation, addressing misconceptions, negative attitudes and social influences.
Strengthen HEWs collaborative work with communities, build positive relationship with communities	The suggestion was that the HEWs were not actively engaging schools in EPI advocacy, demand creation and education and thus, their collaboration should be strengthened.
Engage community and religious leaders in EPI communication and advocacy through dialogue, conversions and forums	Many suggested that the role of community and religious leaders are necessary specially for promoting HPV and COVID-19 vaccine acceptance and church and mosqu e focused approach to behavioral change interventions are necessary.
Engage and re-engage community networks such as women development army groups	The community structure was reportedly getting weaker, social support and social capitals are getting weaker and this has affected community and community structure roles in EPI. Thus, there were strong suggestions that it is necessary to strengthen the community networks, particularly the women development army and their functionality.
Engage multisectors in ACSM in EPI at local levels –HEAA sectors	It was strongly recommended that effective close collaboration and integration to mobilize and engage communities are possible through involving multisectors available at community level, particularly health, education, agriculture and administration (HEAA).
Setting punishment (negative reward) for non-acceptor	The first thing is to raise awareness. After they have gained awareness, the Kebele has to discuss the issues, what should they do to a person who has not been vaccinated? So, rules should be prepared to vaccinate all people. Because it is a matter of life. This is better. Male FGD: P8
Creating favorable conditions/environment for community volunteers and actors at community level	Woreda should create conducive environment during campaign (prepare shelter, waiting chair) Health extension workers

Engage mass media in EPI ACSM	In several settings and places, there was strong emphasis that mass media engagement in EPI ACSM should be strengthened. Special emphasize was laid on use of local mass media platforms such as local FMs, and local TVs stations to educate people and create awareness.
Ensure adequate budget allocation, reducing dependence of NGOs and access to EPI communications resource at the bwer level for health care system, especially facility and community level	
Harnessing social media effectively for positive change in vaccine promotion	There were suggestions that EPI program is not harnessing digital technologies for including for education and tracing defaulters and thus, future works need to harness use of these technologies. In addition, effective use of social media for positive impact by establishing control and monitoring mechanism to track rumors, misinformation and disaffirmatbn activities.
Moving away from centralized IEC message development to local based approach	It was suggested that meeting these demands requires empowering the local health system capacity and skill to develop local sensitive IEC messages for EPI, but adequate quantity/supply and availability, as well as quality.
Avoid coercive and manipulative and use of force to promote vaccine uptake	'Vaccination should not be mandatory. We should be cautious and use psychological approaches to encourage people to get vaccinated. Forcing people or using fear-based messaging is not effective and may backfire. Male FGD: P4
Integrate/strengthen routine health education and counseling services at health facilities with good interpersonal skills	In many cases and settings, health education and counseling service are considered as optional services, and there was a suggestion that health education on EPI should be undertaken as part of routine health services and every client need to be counseled on immunization with thefew key messages.
Communication skills and capacity building to community volunteers and community leaders	For the future activities, influential people, health professionals, individuals from religious institutions should be selected from the community and must be trained to teach the community. Male FGD: P4

Engage males in EPI, addressing gender inequalities in EPI	There was consistent report that immunization was perceived to be female's or women roles whereas husband's/father roles is very limited. Therefore, it was critically important that father should be involved in EPI, and assume responsibility.
Strengthen home visits with tailored health education and counseling on EPI at home settings	Strengthening home visits are important to promote vaccine uptake, address negative attitudes and concerns/hesitations, gives opportunity to identify people with special needs such as disability. Participants laid emphasize on the need to strengthen home visits by HEWs supported by WDAs.
EPI ACSM expertise for better ACSM in EPI: harmonized, integrated and expert guided EPI communications and advocacy.	There were suggestions that EPI ACSM efforts needed by guided by expertise and with adequate human resource (through recruitment of new expertise and taining of existing expertise) at the different levels of the health care system especially at the regional and zonal/district level for guiding ACSM EPI.
Ensure access behavior change enablers (resources and services)	Many participants concerned that demand creation has been done very well; community members have already well informed about immunization, especially for child immunization, and their concern is that the government and stakeholder should make resources and services needed to realize the demand into behavior.
Incentives to volunteers	There was a suggestion that community volunteers expect some incentives when they engage in EPI program such as community mobilization, campaigns organization and running. And should need to be considered

Demand generation practices, challenges and suggested strategies of new vaccines

In the Ethiopian health system, the recently introduced vaccines were HPV and COVID-19 vaccine. During the introduction of these vaccines various strategies of demand generation were implemented to improve acceptance or uptake.

- HPV vaccine: demand generation practices, challenges and suggested strategies

The strategies used to create demand for HPV vaccine were health education by HEWs, WDA, community leaders, religious leaders, and health professionals. Moreover, different education aids or IEC materials (banners, posters, leaflets, and radio) were used. In addition, text messages or SMS are also used as source of information regarding HPV vaccination. The participants mentioned that the HEWs primarily train influential peoples, and then this member of the community will educate and

mobilize targeted people for HPV vaccinations. The community and religious leaders are often engaged in situations of resistances in relation to vaccine uptakes. Religious leaders and influential community members are key to convincing and influencing the community. The HEWs provide HPV educations at health posts, home to home visits, and at schools. Working with schools has helped providers to effectively create demand for HPV vaccines. Trainings are provided to school community to enable the wider dissemination of HPV related messages. In the schools, mini media clubs are used to disseminate messages about HPV vaccine. Participants commented that schools are the most important sites to easily communicate HPV messages.

'In addition to community mobilization, we provide awareness creation trainings at the school level as girls are accessible. In schools with a mini media club, we make students aware through mini media club. The biggest work is done by health extension workers. The health extension workers are available in all kebeles, and extensive awareness creation activities are done through these health workers' EPI focal Zonal level

The suggested demand generation strategy to improve HPV vaccines uptake was strengthening engagement of community leaders, religious leaders, kebele administrators, WDAs, HDAs, and government sectors (women and child affairs office, and schools) on HPV vaccine education and communication.

COVID vaccine: demand generation practices, challenges and suggested strategies

Similarly, COVID-19 vaccine communication and education was the primary strategy used to increase demand for the vaccine uptake by the community and targeted group of people. The summary of COVID-19 hesitation strategies and suggested vaccine promotion strategies were presented in Table 8.29.

Table 8.29: COVID-19 hesitation mitigation strategies, and suggested vaccine communication strategies in Ethio-

COVID-19 Hesitation mitigation Strategies	COVID-19 Vaccine communication and advocacy strategies
Engagement of religious and community leaders at local level for serving as model and as advocate for the vaccine<br><br>Mass media awareness creation and social mobilizations<br><br>Media Advocacy activities at national and regional levels. Supported by partners (mainly UNICEF, WHO) several advocacy activities that engaged politicians and public figures helped to overcome resistance and hesitation, specially targeting urban residents. In this advocacy digital media was also utilized widely.<br><br>Engaged community and religious leaders at different levels to persuade, motivate and set role model for the public<br><br>Community dialogue and conversions in some areas<br><br>Law makers' engagement to enforce mandatory vaccination and forceful approach such as linking vaccinations with SAFETY NET program<br><br>Engaged school students in COVID-19 communication and message creation<br><br>Strengthen facility and community level IPC<br><br>Community engagement, mobilization and education such as questions and answer program<br><br>Continuous education to communities and community actors	Use multisectoral approach to social mobilization, advocacy and remobilization<br><br>Use public figures and influential for advocacy and vaccine promotion<br><br>Re-engage communities through dialogue and conversions using social networks. Many argued that mass media education is not enough, rather people need face to face interactions and discuss on this vaccine and future COVID-19 education need to address it

8.13 Conclusions

In this national survey large number of participants involved from different categories and health institutions from different level. For the demand generation and communication thematic areas, we used data from the household survey to see the community knowledge and attitude towards the different vaccines introduced in the country as well as data from the FGD and KIIs to assess the current EPI communication practices and challenges.

The study revealed that there is widespread information about the childhood immunization (94.5%) and COVID-19 vaccines (94.7%). However, we also revealed that majority of caregivers lacked adequate knowledge about the target, antigens and specific schedules of childhood immunization. The common source of information for COVID-19 were audio mounted vehicle and health workers. On the other hand, large majority of surveyed adolescents didn't hear about HPV (74.9%), and only 54.3% heard about HPV vaccination. Most common sources of information for HPV were teachers, parents and health workers. The awareness among adolescents regarding cervical cancer, HPV infection and HPV vaccine was low and this is detrimental in uptake of vaccination services.

The study identified several factors that impedes uptake of childhood, COVID-19 and HPV vaccinations. The main barriers to uptake of childhood immunization were multi vial policy, gaps in IPC skills of providers, and issues related service accessibility and availability such as distance and shortages of human resources. Though large majority of health workers received EPI focused training (68%) and supervisions (82.9%), the study indicated that there were major gaps in the IPC skills of providers in immunization service delivery. The health workers performed less in various aspects of GATHER (Greet, Ask, Tell, Help, Explain, and Recommend) approach. In hard-to-reach settings, childhood vaccination services are inaccessible to communities. Although large majority of communities accepts childhood vaccination services, still some gaps are observed associated to communities' belief and perceptions as revealed in the study. Regarding COVID-19, communities' misconception and misinformation about the disease and vaccine were widely spread, and are significant barriers to uptake of vaccine.

Almost around half of adolescents (46%) yet didn't have the HPV vaccination and this could potentially be linked to wrong perception that HPV vaccine might be used as birth control strategy, and causes infertility. But still among those who didn't have the vaccine, majority of them intend to take in near future even though they will not be eligible age group in Ethiopian context. The most common reason for not getting vaccine was lack of adequate information and teachers, parents and health care workers take the leading role of motivation for taking vaccine.

The national EPI assessment revealed that several strategies, and platforms were used to create demand for EPI. Individuals and institutions that ranged from the kebele level to the national level were engaged in various EPI advocacy, communication, and social mobilization activities. Commonly a combination of settings and platforms are used to create demand for EPI. However, the study has also indicated that the EPI ACSM activities are getting weaker recently. More commonly ACSM activities are done for non-routine immunization activities, and for routine it is rarely implemented.

The EPI demand generation activities in Ethiopia has faced several challenges. The major challenges identified in this assessment were: inadequacy and local insensitiveness of IEC/BCC materials, shortages of IEC budget and resources, weak community networks engagement in EPI communications (due to lack of incentives, less acceptance of volunteers by community, resistances from the community

etc..), inadequate HR (HEWs) at health post level particularly in the populous and hard to reach areas, lack of dedicated person that coordinates demand generation (ACSM) at lower levels, lack of transportations and distance services, weakening of HEP system due to several reasons including insecurity, a growing perception of demand creation is a finished agenda, shortages or delay of behavioral change enablers (e.g., vaccination services), and weak utilization of digital technologies. Though the community structures and networks play pivotal role in EPI demand creations, their involvements are getting lesser. This significantly affects the efforts made to improve uptake of immunization services in Ethiopia.

8.14 Recommendations and Implications

Though EPI program has been implemented for several years, the strategies that help to improve uptake of vaccines (demand generation activities) are still facing several challenges. This situation will result to and significantly affects the effort made to ensure the child survival goal through increasing coverage and uptake of the immunization services in Ethiopia. This inquires an intensified effort from MOH and its partners towards raising awareness and correct the wrong perception towards COVID and HPV vaccine using community platforms and involving parents as well as organizations like schools and religious institutions to increase vaccine uptake. Besides working with various stakeholders, there is a need to revise the open vial policy, which is mentioned as a critical issue for dropout and non-vaccination of children. Refresher training focusing on the importance of IPC activities and the correct way to communicate key immunization messages needs be organized for health workers. Additionally, regular supportive supervision of health workers' practices in this area can help identify gaps and areas for improvement, particularly on locations with low mean score of IPC practice

In summary the set of programmatic recommendations that helps to improve EPI ACSM activities are:

- Ensure that budget for EPI demand generation activities are allocated
- Ensure that a dedicated EPI ACSM personnel that coordinates EPI communication activities is available at all levels of the health system
- Ensure that adequate HEWs are available in a highly populated and marginalized settings of Ethiopia
- Revitalizing the community structures and networks through building their capacity and arranging incentive packages for community volunteers
- Strengthening the engagement of community and religious leaders in EPI communications
- Ensure that EPI IEC/BCC materials are adequately available at lower levels, and locally sensitive in terms of language and pictorial representations
- Strengthening multi-sectors engagements in EPI demand generation-The sectors including Health, Education, Agriculture and Administration (HEAA) should be actively engaged in mobilizing the community and EPI communications.
- Strengthening the use of multi-media (including mass media, social medias) in EPI communication and promotion activities

MONITORING AND EVALUATION

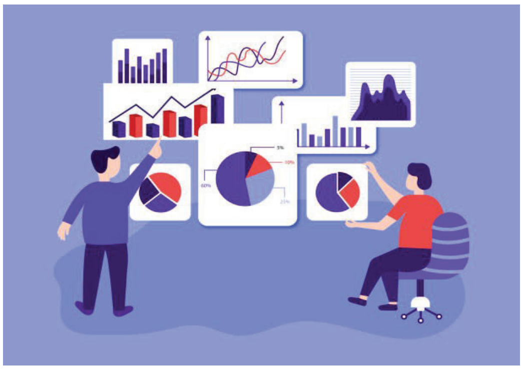

9. MONITORING AND EVALUATION

The immunization M&E structure, practice and mechanism assessment focused to assess on how the immunization services are monitored and evaluated at different level of the health system. Under this section, the EPI program M&E structure, HR arrangement, M&E practices and mechanisms, immunization data quality and use practices.

Key findings
• While the health system has a structure for monitoring and evaluation, there is a noticeable absence of a specific structure for Monitoring and Evaluation (M&E) of the Expanded Program on Immunization (EPI).<br>• Data entry staff are present in most regions, zones, districts, and facilities, but there is a shortage of staff trained in data management at all levels of the system.<br>• Only around half of the health posts use sta ndard data collection and reporting templates.<br>• Independent M&E mechanisms for EPI are present in three quarters of RHBs and 82.4% of ZHDs.<br>• RHBs and ZHDs conduct performance monitoring and review sessions at the EPI case team level.<br>• The report highlights varying levels of EPI data use culture across different health system levels, with some regions demonstrating strong practices in data utilization.<br>• Active monitoring practices for immunization performance and stock monitoring are observed at regional, zonal, and district levels.<br>• Disparities in data completeness and timeliness exist among regions, with some showing higher rates than others.<br>• Capacity -building efforts are needed to ensure regular attendance at performance assessment sessions and enhance the use of performance tracking systems for program improvement.<br>• Discrepancies in resource allocation and access to information use guidelines underscore the importance of standardized guidelines and consistent data analysis practices.

9.1 Immunization M&E Structure

Monitoring and Evaluation Unit

Even of the EPI activities are followed, monitored and evaluated using different approaches, it was report-ed that as there is no independent and separated unit designed specifically for M&E purpose. However, at all administrative levels, there is an overall M&E unit assigned to support and follow the implementation of different health programs.

Monitoring and Evaluation Mechanisms

Table 9.1 illustrates the mechanisms used to follow, monitor and evaluate the progress of the immunization related activities.

Respondents from a total of 12 RHBs and 51 ZHDs were asked about the M&E mechanisms available for EPI program at region and zonal levels. The participants in all of the regional health bureaus reported that M&E mechanisms such as review meetings and supportive supervisions were in place and in three quarters of RHBs (75%) Technical Working Group (TWG) meetings were available. An independent M&E mechanism for EPI exists in large majority (75%) of surveyed regions.

Taking a look at the M&E mechanisms at the zonal and district / sub-city level, large majority of zones and half of woreda health office/ sub-cities conducts review meeting (84.3% and 51.95) and supervisions (88.2% and 55.41), respectively. Independent M&E mechanisms for EPI was available in greater than eight out of ten of the ZHDs (82.4%) and less than half (47.1%) have partner or stakeholder forums to monitor and evaluate EPI program.

On the other hand, 81.5% of health facilities have reported implementing EPI monitoring mechanisms, indicating widespread engagement in monitoring and evaluating EPI activities. These findings highlight a structured approach to EPI program monitoring and evaluation, underscoring the importance of assessing the effectiveness and efficiency of these mechanisms in improving immunization outcomes and ensuring the delivery of essential services to target populations.

Table 9.1: Available M&E Mechanisms for EPI program monitoring and evaluation at different health system levels in Ethiopia, 2023.

M&E Activities	Regional	Zones	WorHOs
M&E mechanisms	100	100	100
Uses review meetings as an M&E mechanism	100	84.3	51.95
Uses supervision as an M&E mechanism	100	88.2	55.41
Use TWG meetings as an M&E mechanism	75	54.9	42.42
Have independent M&E mechanisms for EPI	75	82.4	91.34
Have a partner forum to monitor and evaluate EPI	91.7	47.1	46.58
Conducts partner forums			
Monthly		4.2	18.02
Quarterly	54.5	70.8	60.36
Bi-annually	27.3	25	13.53
Annually	18.2		8.11
Conducts regular review meetings to evaluate EPI performance	100	80.4	78.35
Conducts EPI performance review meeting			
Monthly		2.4	22.65
Quarterly	66.7	85.4	69.61
Bi-annually	25.0	7.3	6.63
Annually	8.3	4.9	1.10
Have performance monitoring & review sessions at EPI case team	100	74.5	40.69
Conducted EPI-focused integrated review meetings in the last 3 months			
Yes, observed	83.3	60.8	51.52
Yes, reported	8.3	21.6	26.41
No	8.3	17.6	22.08

Human resource for EPI M&E activities

In the current survey, nine out of twelve (75%) regions have a designated person responsible for recording EPI data, and 10 of the 12 regions (83.3%) have a designated person responsible for reviewing EPI data quality before submission to the federal ministry of health. Despite 7 claiming to possess Health Information Technicians (HITs), a mere 3 attest to having sufficient human resources to effectively utilize these tools.

At the Zonal level, more than three-fourths (84.3%) of the health department have a designated person responsible for reviewing immunization data quality before submission. Three-fourths (70.59%) of zones had one or more staff received training on data management. The data shows a mere 15.69% boast the

presence of a dedicated Monitoring and Evaluation (M&E) advisor, with just half of them asserting that their human resources are sufficient for the task at hand. Despite 60.7% claiming to possess Health Information Technology (HIT), a mere 22.5% attest to having sufficient human resources to effectively utilize these tools.

At the district/sub-city level, three-fourths (76.1%) and (77%) of districts/sub-cities had a designated person responsible for entering EPI data and reviewing EPI data quality before report submission. About one in ten (13.8%) of the surveyed districts and sub-cities had staff who had received training on the data management process. An impressive 85.7% report the presence of an M&E advisor, yet only 42.2% deem their staffing levels sufficient for the task at hand. Similarly, among those boasting an M&E officer, comprising 44.1% of woredas, a mere 29.4% express satisfaction with their human resource allocation.

At the health facility level (health centers and hospitals), more than half (56.5%) of them had one or more staff that received data management training. Only a quarter (27.6%) of the staff received data management training in Benishangul Gumuz. Seven in ten (70.5%) of the public health facilities received supportive supervision from the district, zone, and region. At the health post level, less than one-quarter (22.55%) of health pots had designated responsible staff for reviewing the quality of immunization data. Health posts in Afar, Benishangul Gumuz, and Harari regions didn't have designated responsible staff for reviewing the quality of immunization data. Only 19% of the health post staff received immunization data management training Table 9.2

Table 9.2: Immunization data management responsible person at different health system level in Ethiopia, 2023

		The proportion of respective health system levels that have:								
		Designated person responsible for entering EPI data		A designated person responsible for reviewing EPI data quality before submission			Received training in data management and Processing			
		Zone	District	District	HC & Hospital	Health Post	Zone	District	HC & Hospital	Health Post
	National	84.31	76.19	77.06	72.91	22.55	70.59	13.85	56.51	19.06
Setting	Urban	-	26.33	24.59	75.45	8.67	-	24.59	58.93	20.12
	Agrarian	-	19.85	16.94	75.74	25.96	-	16.94	61.76	20.40
	Pastoral	-	23.08	28.21	45.71	12.23	-	28.21	42.86	9.25
Regions										
	Addis Ababa		100	100	94.74	-	-	15.38	30.94	-
	Afar		78.57	71.43	55.56	0	-	0	46.82	9.09
	Amhara	92.31	76.19	83.33	73.33	42.55	61.54	30.95	50.27	29.79
	Benishangul Gumuz	100	100	87.50	37.50	0	100	0	27.61	25
	Dire Dawa		100	75	71.43	87.50	-	0	94.57	25
	Gambela	100	25	25	50.00	44.44	100	0	69.46	33.33
	Harari		78.13	81.25	80.00	0		17.19	100	29.41
	Oromia	81.82	100	100	85.86	20.59	72.72	16.67	65.19	23.53
	SNNP	76.92	68.75	68.75	76.92	37.50	53.85	12.50	54.25	18.75
	Sidama		60	60	83.33	15	-	8	57.36	5
	Somali	66.67	66.67	76.19	50.00	11.54	83.34	4.76	40.96	0
	Southwest Ethiopia People	100	78.57	64.29	59.09	11.70	100	0	73.53	11.76

Guidelines, formats, and log-books availability

In the current survey, around 83.3% of the region keeps the log book/electronic systems to track monthly EPI performance and 80.3% of the zone had kept log books or electronic systems to track monthly immunization performance. More than half (56.6%) of zonal health departments had written guidelines on immunization data quality assessment. Close to half (49.3%) of the districts/Sub-cities had written guidelines on EPI data quality assessment.

At the facility level, a considerable infrastructure supports monitoring and evaluation efforts, with 79.4% of healthcare centers and hospitals possessing HMIS recording and reporting manuals, 65.3% equipped with data verification checklists, and 68.3% having performance indicator reference sheets available. These resources play a crucial role in ensuring the accuracy and reliability of data collected within the facility, providing guidance for staff on recording procedures, verifying data integrity, and monitoring key performance indicators, ultimately contributing to informed decision-making and effective healthcare delivery.

Across health posts, there exists a varied landscape in the availability of standard data collection and reporting templates, with more than half (55.6%) equipped with such tools. However, notable disparities are observed among regions, with the lowest rates found in the Afar (27.2%) and Somali (38.4%) regions. Conversely, the provision of Data Quality Assessment (DQA) sheets is notably limited, with only 17.2% of health posts possessing these critical tools for evaluating the accuracy and reliability of collected data. These findings underscore the importance of addressing regional disparities and bolstering resources for comprehensive data management and quality assurance efforts across all health posts Table 9.3

Table 9.3: Guidelines, formats, and logbooks for EPI data management at all health system level, 2023.

		The proportion of respective health systems that have:								
		Written guidelines on EPI data quality assessment	Keep the logbook/electronic systems to track monthly EPI reports	Standard data collection tools and reporting template		Performance indicator reference sheets	HMIS recording and reporting manuals	Data | Verification Checklist available?	Standard data collection tools and reporting formats	Data quality assessment sheet
		Zones	Districts	Zones	Health Post	HC & Hospital	HC & Hospital	HC & Hospital	HP	HP
Region	National	56.86	49.35	80.39	56.60	68.35	79.49	65.32	56.18	17.23
	Addis Ababa		76.92			63.16	68.42	94.74		
	Afar		78.57		27.27	50.00	77.78	61.11	27.27	27.27
	Amhara	53.85	54.76	84.62	63.83	68.00	74.67	70.67	63.83	29.79
	Benishangul Gumuz	0	75	0	66.67	50.00	68.75	6.25	66.67	0.00
	Dire Dawa		50		100	71.43	85.71	64.29	100.00	25.00
	Gambela	66.67	75	66.67	55.56	70.00	90.00	50.00	55.56	11.11
	Harari		39.06		64.71	60.00	90.00	80.00	64.56	17.65
	Oromia	63.64	50	100	58.82	82.83	91.91	82.83	58.82	19.12
	SNNP	61.54	68.75	84.62	46.88	67.31	71.15	57.69	46.88	21.88
	Sidama		32		55	79.17	87.50	66.67	55.00	10.00
	Somali	33.33	42.86	33.33	38.46	50.00	69.44	36.11	38.46	0.00
	South West Ethiopia	75	21.43	100	52.94	59.09	77.27	54.55	52.94	5.88

Planning

From the total of 12 regions included in the survey, the majority (83.3%) had target number of children to be vaccinated in the calendar year. Except for one region, all regions knew the proportion of infants per vaccination type. Three-fourths (75%) of the regions had updated. catchments map, including outreach sites for 2015 EFY All regional health bureaus use the same denominators for different programs.

At the zonal level, from the total of 51 zonal health departments included in the survey, the majority, 88.2% knew the target number of children striving to be vaccinated in the calendar year, and zones in Somali regions knew their target by half. More than three-fourths (78.4%) of zones knew the proportion of infants per vaccination type (the service point). Three-fifth (60.7%), of zones used the same denominator to plan different programs. One-third of zones in Gambela and 30.7% in SNNP were using the same denominator for different programs. Less than half (45.1%) of the Zonal health departments had an updated 2015 EFY catchment map, including the outreach site. None of the zonal health departments in the Benishangul Gumuz region had updated the 2015 EFY catchments map, including the outreach site Table 9.4

Table 9.4: Planning and demographic-related information of immunization by zonal health department, 2023

		Target the number of children the health department strives to vaccinate during the calendar year	Proportion of infants per vaccination type known as per the service point	Used the same denominator for different programs	Updated 2015 E.C catchments map including outreach sites
	National	88.24	78.43	60.78	45.10
Regions	Amhara	100	92.31	69.23	53.85
	Benishangul Gumuz	100	100	100	0
	Gambela	66.67	66.67	33.33	33.33
	Oromia	90.91	81.82	90.91	54.55
	SNNP	92.31	69.23	30.77	38.46
	Somali	50	50	50	33.33
	South West Ethiopia Peoples	100	100	75	50

The finding at the district level indicates that from a total of 231 districts included in the survey, 79.22% of districts and sub-cities knew their target number of children strived to be vaccinated during the budget year. The highest practice was observed in Addis Ababa (100%), Benishangul Gumuz (100%), Dire Dawa (100%), and Oromia (100%), and the lowest in the Somali (47.5%) and Afar (50%) regions. More than half (57.5%) and (57.1%) of the districts and sub-cities used the same denominator to plan for different programs and had updated the 2015 EFY catchment map, including outreach sites in the districts and sub-cities, respectively Table 9.5

Table 9.5: Demographic and planning characteristics of Districts/Sub-cities in Ethiopia, 2023

		Target number of children strived to be vaccinated in the calendar year	Proportion of infants per vaccination type	Used the same denominator for different programs	Updated 2015 E.C catchment site map
	National	79.22	75.76	57.58	57.14
Setting	Urban	88.52	83.61	57.38	55.74
	Agrarian	84.68	83.87	65.32	63.71
	Pastoral	56.41	46.15	43.59	51.28
Regions					
	Addis Ababa	100	92.31	46.15	30.77
	Afar	50	50	42.86	35.71
	Amhara	78.57	83.33	66.67	54.76
	Ben/Gumuz	100	87.50	87.50	87.50
	Dire Dawa	100	100	75	75
	Gambela	75	100	50	0
	Hareri	84.38	81.25	53.13	65.63
	Oromia	100	100	83.33	33.33
	SNNP	75	75	56.25	68.75
	Sidama	88	84	60	64
	Somali	47.62	28.57	42.86	57.14
	South West Ethiopia	78.57	64.29	64.29	50

At the health Center and hospital level, from the total of 395 health facilities included in the evaluation survey nationally, more than nine in ten (94.7%) of the health facilities had a target number of children striving to be vaccinated in the calendar year. Close to nine in ten (89.5%) and 89.40% of immunization-delivering health facilities knew their proportions of infants per vaccination type and used the same denominator for different programs, respectively. Nearly two-thirds (62.8%) of the health facilities had an updated 2015 EFY catchment map, including outreach sites Table 9.6

Table 9.6: Immunization planning and demographic characteristics at the health facilities in Ethiopia, 2023

		Target number of children the HFs strive to vaccinate the calendar year	Proportion of infants per vaccination type known as per the service point	Used the same denominator for different programs	Updated 2015 E.C catchments map including outreach site/HF
Setting	National	94.72	89.52	89.40	62.86
	Urban	94.17	88.10	89.72	56.37
	Rural	95.49	91.50	88.95	71.88
Health facility type	Health center	96.07	92.23	88.83	70.87
	Specialized Hospital	100	59.69	92.52	11.91
	General Hospital	81.86	70.57	85.21	14.01
	Primary Hospital	88.09	86.99	94.07	39.26
Region	Addis Ababa	100	72.34	89.69	20.62
	Afar	81.31	62.52	87.48	75.05
	Amhara	91.91	86.96	79.78	61.12
	Benishangul Gumuz	91.41	91.41	91.41	59.51
	Dire Dawa	100	100	100	27.14
	Gambela	100	100	70.80	58.39
	Hareri	93.42	86.85	100	19.73
	Oromia	99.06	93.99	89.74	63.81
	SNNP	88.41	83.89	92.74	57.17
	Sidama	96.32	96.32	100	86.80
	Somali	78	78.34	96.91	56.19
	South West Ethiopia	100	95.17	100	47.05

Similar survey was conducted across 267 randomly selected health posts nationally. Accordingly, three-fourths (74.4%) of the health posts had target numbers of children for vaccination. Health posts in the Somali (34.6%) and Afar (36.3%) regions had the lowest target group. More than two-thirds (69.6%) of the survey sites had a proportion of infants per vaccination type. About one in four (26.9%) health posts reported in the Somali region. Half (50.6%) of the health posts used the same denominator for different programs. Lowest in Afar (18.1%), Gambela (33.3%), SNNP (40.6%), South West Ethiopia (47%), and Oromia (48.5%) regions. Less than half (44.7%) of the health posts had an updated 2015 EC catchment map, including the outreach site. Amhara (57.4%) and Gambela (55.5%) regions are the highest Table 9.7

Table 9.7: EPI annual plan and demographic characteristics of health Posts in Ethiopia, 2023

		Target number of children to vaccinate the calendar year	Proportion of infants per vaccination type known as per the service point	Same denominator used for different programs	Updated 2015 E.C catchments map including outreach sites
National		74.47	69.67	50.60	44.70
	Urban	81.02	65.52	47.35	27.09
Setting	Agrarian	79.01	75.22	59.97	49.36
	Pastoral	38.58	31.51	24.52	29.86
Regions					
	Addis Ababa				
	Afar	36.36	18.18	18.18	36.36
	Amhara	82.98	78.72	61.70	57.45
	Benishangul Gumuz	91.67	91.67	75	25
	Dire Dawa	100	100	87.50	25
	Gambela	66.67	44.44	33.33	55.56
	Hareri	58.82	58.82	58.82	29.41
	Oromia	83.82	79.41	48.53	47.06
	SNNP	81.25	65.63	40.63	50
	Sidama	60	55	55	35
	Somali	34.62	26.92	30.77	30.77
	South West Ethiopia	70.59	64.71	47.06	41.18

9.2 Immunization data quality

Report timeliness and completeness monitoring logbook

Regions level

According to the survey findings at the regional level, more than eight in ten (83.33%) of the regions had an updated immunization performance monitoring chart for 2015 EFY and checked report timeliness and completeness. More than half (58.33%) of the surveyed regions gave routine feedback on immunization performance. All regions used immunization data for decision-making Table 9.8

Table 9.8: Report completeness, timelines, and performance monitoring of Immunization at the regional level, Ethiopia 2023.

Variables/Areas	Responses		
	No	Yes	Yes, but not observed
Updated EPI monitoring chart /tables displayed for EPI performance for the 3rd quarter of 2015 EFY.	2(16.67%)	10(83.33%)	0
Region checks to report completeness from the EPI unit	1(8.33)	10(83.33%)	1(8.33%)
Region checks reporting timelines from EPI unit/health facilities/H.P./zone	0	10(83.33%)	2(16.67%)
Up-to-date data monitoring of the current review period (3rd quarter of 2015 EFY) immunization dropout rates	0	11(91.67%)	1(8.33%)
Monitoring of health unit's vaccine stock out at zone/region level	1(8.33%)	8(66.67%)	3(25%)
The region gives routine feedback based on the EPI performance analysis	1(8.33%)	7(58.33%)	4(33.33%)
Regular meetings with health unit workers to discuss EPI performance	1(8.33%)	8(66.67%)	3(25%)
EPI data used for decision-making	0	12(100%)	0
Region conducted a review meeting with the health units in the last two quarters (2nd& 3rd 2015 EFY)	0	9(75%)	3(25%)

Zonal level

Three-fourths (74.51%) of zonal health departments were checked for reporting completeness from immunization units and up-to-date monitoring of immunization dropout rates, respectively. Two-thirds (68.63%) of health departments were checked for reporting timelines. Less than two-thirds (59.24%) of zonal health departments monitored vaccine stock in the health unit Table 9.9

Table 9.9: Report completeness, timeless, and performance characteristics of Zonal health department, 2023

	The proportion of zonal health departments that have:				
	An updated EPI monitoring chart displaying performance for 3 months preceding the survey	Checking mechanism for EPI data reporting completeness at the EPI unit	Checking mechanism for EPI data reporting timeliness at the EPI unit	An up-to-date data monitoring of the current review period immunization dropout rates	Monitoring system for the health unit's vaccine stock
Addis Ababa*	-	-	-	-	-
Afar*	-	-	-	-	-
Amhara	30.77	10	69.23	92.31	61.54
Benshangul Gumuz	0	0	0	100	0
Dire Dawa*	-	-	-	-	-
Gambela	33.33	33.33	33.33	66.67	33.33
Harari*	-	-	-	-	-
Oromia	72.23	90.91	90.91	90.91	54.55
SNNP	69.23	61.54	61.54	69.23	46.15
Sidama*	-	-	-	-	-
Somali	50	50	66.67	16.67	33.33
Southwest Ethiopia	100	75	75	75	100
National	56.86	74.51	68.63	74.51	59.24

District/sub-city level

At the district/sub-city level, around two-thirds (66.67%) and (66.27%) of the respondents had updated immunization monitoring charts and tables for the 3rd quarter of 2015 EFY. They had complete reports from the EPI unit. Close to two-thirds (64.94%) of the districts and sub-cities had received timely reports from the immunization units. Only 42.86% of the districts and sub-cities received timely reports from the units. About seven in ten (70.13%) districts or sub-cities have up-to-date immunization dropout rates data Table 9.10

Table 9.10: Report completeness, timelines, and performance monitoring of immunization at the District/sub-city health offices, 2023

		The proportion of Districts/sub-citythat have:			
		Updated EPI monitoring chart displayed for performance of 3 months preceding the survey	Reporting completeness from the EPI unit	Reporting timelines from EPI unit/health facilities	Up-to-date data monitoring of the current review period immunization dropout rates
Setting	Urban	77.05	67.21	72.13	73.77
	Agrarian	68.55	73.39	67.74	78.23
	Pastoral	51.28	48.12	51.28	46.15
Region	Addis Ababa	84.62	84.62	92.31	92.31
	Afar	64.29	57.14	50	57.14
	Amhara	64.29	69.05	61.90	80.95
	Benshangul/gum	62.50	62.50	62.50	87.50
	Dire Dawa	50	50	75	75
	Gambela	100	25	50	50
	Harari	71.88	71.88	65.63	76.56
	Oromia	83.33	83.33	100	83.33
	SNNP	56.25	75	75	50
	Sidama	72	72	72	76
	Somali	38.10	78.10	42.86	28.57
	Southwest Ethiopia	71.43	57.14	57.14	64.29
Total		66.67	66.23	64.94	70.13

Health Facility level

At the health center and hospital level, more than three-fourths (80.45%), (78.52%), and (79.82%) updated their immunization monitoring charts for the 3rd quarter of 2015 EFY, updated immunization dropout rates, and used immunization data for decision-making, respectively. Over half (52.17%) of the health facilities were given routine feedback based on immunization performance. Less than one in three (27.61%) health facilities provided routine feedback in the Benshangul Gumuz region. Three-fourths (75.90%) of the facilities regularly met with health unit workers to discuss immunization performance. Gambela (40.27%) and Somalia (48.82%) are the regions with the lowest number of regular meetings conducted with health workers. Most (86.20%) of the health facilities were checked for reporting timelines from immunization units Table 9.11

Table 9.11: EPI report completion and performance monitoring of Health facilities in Ethiopia, 2023

		The proportion of health facilities that have:					
		Updated EPI monitoring chart displayed for performance of 3 months preceding the survey	Monitoring system to checks to report completeness from the EPI unit	Monitorig system to checks to report timeliness from the EPI unit	Up-to-date data monitoring of the current review period immunization dropout rates	Given routine feedback based on the EPI performance analysis	Regular meetings with health unit workers to discuss EPI performance
Setting	Urban	78.61	90.39	89.06	80.67	53.61	77.65
	Rural	83.02	86.49	85.41	75.54	50.16	73.48
Health facility type	Health center	85.96	88.86	85.46	81.51	53.21	76.83
	Spe/ Hospital	38.86	69.43	100	38.86	69.43	97.82
	Gen/Hospital	71.99	96.87	92.06	71.04	55.80	67.35
	Pri/Hospital	55.44	90.94	84.74	72.03	36.61	65.08
Region	Addis Ababa	51.56	75.78	96.56	41.25	55.16	96.56
	Afar	100	87.48	59.35	84.39	50	53.18
	Amhara	79.54	89.36	83	85.17	51.46	82.65
	Benshangul Gumuz	91.41	66.41	56.90	52.61	27.61	55.22
	Dire Dawa	100	89.14	89.14	94.57	83.77	94.57
	Gambela	70.80	80.54	38.93	61.07	30.54	40.27
	Harari	100	100	100	86.85	86.54	93.42
	Oromia	85.90	93.79	93.79	80.72	53.16	75.64
	SNNP	79.38	81.15	75.84	64.25	59.73	74.87
	Sidama	79.44	92.64	96.32	92.64	61.04	81.60
	Somali	69.05	75.24	55.01	59.53	34.77	48.82
	Southwest Ethiopia	67.44	75.85	85.51	55.46	36.14	56.71
National		80.45	88.76	86.20	78.52	52.17	75.90

Health post level

At the health post level, more than two-thirds (68.41%) of health posts had immunization reports for the last three quarters of 2015. EFY Somalia (36.36%) and Afar (34.62%) are regions with low availability in the last three-quarters of reports of 2015 EFY. Less than half (46.95%) of the facilities had systems for late reporting. About two in four (41.25%) health posts had the completeness of immunization reports recorded and monitored at the H.P. level. Seven in ten (70.57%) health posts had a timeliness of reports recorded and monitoring practices. Among regions, Somalia (23.08%), Gambela (44.44%), and Afar (45.43%) had the lowest report timelines in the health posts. Less than one in five (17.88%) health posts conducted LQAS before submitting the monthly report. Health posts in the Somali, Dire Dawa, and Gambela regions were not subjected to a monthly LQAS before submitting their reports Table 9.12

Table 9.12: Availability of reports, IQAS, report completeness, and timelines of health Posts in Ethiopia, 2023

		The proportion of health posts that have:				
		EPI reports for the entire period (last three-quarters of 2015 E.C)	A procedure in place for dealing with late reporting	Completeness of immunization reports recorded and monitored	Timeliness of the immunization reports recorded and monitored	Data quality assurance mechanisms (IQAS before the monthly report)
Setting	Urban	67.73	39.37	48.11	68.79	20.03
	Agrarian	73	50.79	41.62	77.46	19.05
	Pastoral	38.58	26.87	32.77	26.49	6.92
Region	Addis Ababa					
	Afar	36.36	27.27	36.36	45.45	9.09
	Amhara	82.98	57.78	51.06	74.47	27.66
	Benshangul Gumuz	50	33.33	41.67	66.67	8.33
	Dire Dawa	87.50	87.50	12.50	62.50	0
	Gambela	66.67	42.86	55.56	44.44	0
	Harari	64.71	42.86	11.76	64.71	5.88
	Oromia	76.47	55.56	50	80.88	22.06
	SNNP	62.50	40.63	43.75	59.38	15.63
	Sidama	60	31.58	20	65	10
	Somali	34.62	23.08	26.92	23.08	0
	South West Ethiopia Peoples	47.06	40	17.65	82.35	17.70
National		68.41	46.95	41.25	70.57	17.88

Reporting Accuracy

Reporting Accuracy

Reporting accuracy was assessed for two reportable data items: Penta 3 and Measles 1. The number of children who received these vaccines was recounted from source documents that health facilities used to prepare their reports. The data from recounting was then compared with data reported to the DHIS2 platform to estimate verification factors (the proportion of reported numbers of vaccinated children verified from source documents).

Verification factor (V.F.)=(Number recounted)/(Number reported)   X 100%

When there is accurate reporting, the verification factor is expected to be 100%. In line with current standards of the Ethiopian health sector, we defined acceptable reporting accuracy as one with a verification

factor between 95% and 105%. Reports with verification factors lower than 95% or higher than 105% are considered inaccurate, reflecting over-reporting and under-reporting, respectively.

We attempted to compare reported data elements with recounted data from source documents for 4,062 data elements expected to be reported from 677 health institutions, including 314 health centers, 72 public hospitals, and 291 health posts checked for three consecutive monthly reports on two child vaccination indicators. Complete reports and source documents allowing estimation of report verification factors were available only for 2,413 (59.4%) of the reportable data elements. Not submitting a report, submitting incomplete reports with missing data elements, and lacking/incomplete source documents were the reasons for inadequate documentation for the estimation of verification factors Figure 9.1

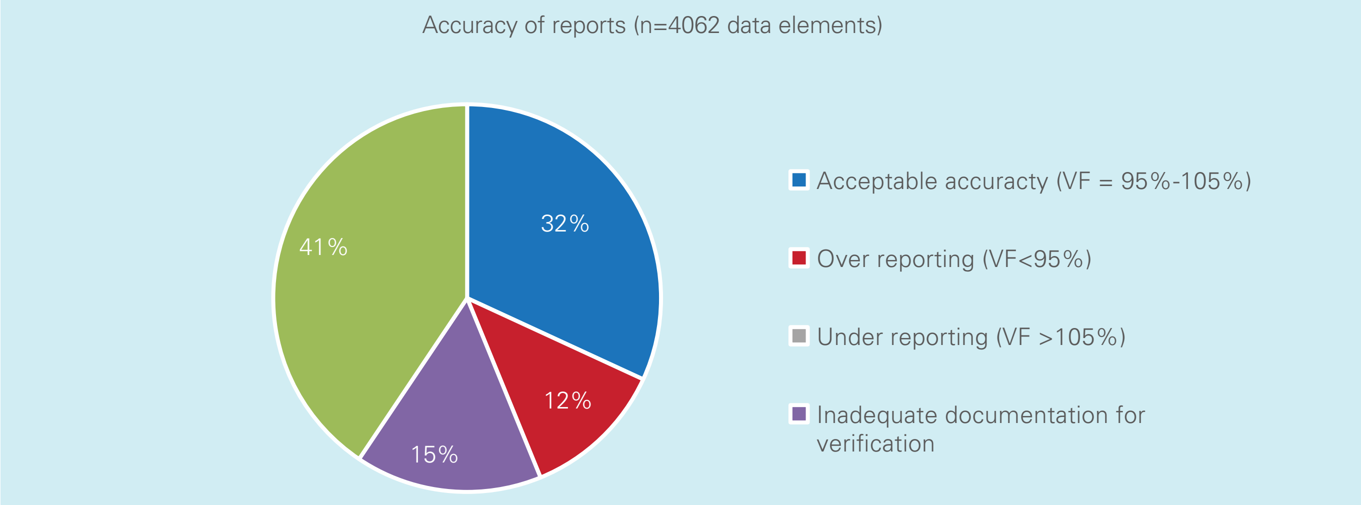

Figure 9.1: Data accuracy of reports for the E.I. data at different health service levels in Ethiopia, 2023.

In general, the availability of complete documentation and the accuracy of reported data elements was better in public hospitals than in health centers and health posts. Health posts were the worst regarding missing reports and source documents for data verification. Against our expectations, we found that under-reporting is more common than over-reporting; from the total of 4,062 data elements checked for verification purposes, 633 were under-reported, whereas 485 were over-reported Figure 9.2.

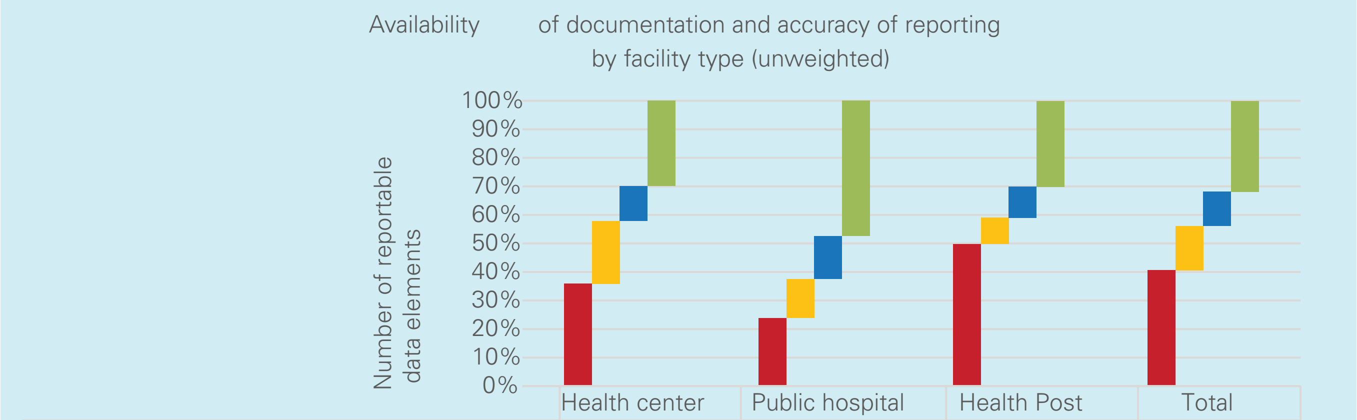

	Health center	Public hospital	Health Post	Total
Acceptable accuracty (VF = 95%-105%)	564	205	526	1295
Over reporting (VF<95%)	230	65	190	485
Under reporting (VF >105%)	413	59	161	633
Inadequate documentation for verification	677	103	869	1649

Figure 9.2: Distribution of health facilities EPI Data accuracy by facility type in Ethiopia, 2023

The proportion of health facilities with accurate reporting was generally very low, ranging from 33.4% to 38.0% for the six data points. However, it was relatively higher for public hospitals than for health centers. Health facilities in pastoral settings and the regions of Afar, Benshangul Gumuz, Somali, SNNP, and South West Ethiopia had the lowest proportions of health facilities with accurate reports. On the other hand, health facilities in urban settings and those in Dire Dawa, Harari, and Gambella had the highest rates of accurate reporting on the selected child vaccination indicators Table 9.13.

Table 9.13: The proportion of health facilities with acceptable accuracy of reports (Weighted percent) in Ethiopia, 2023

		% of health facilities with accurate reports					
		Pentavalent 3			Measles		
		Month 1	Month 2	Month 3	Month 1	Month 2	Month 3
Total		34.1	36.6	38.0	33.4	34.4	35.3
Setting	Urban	41.6	42.4	43.8	40.1	43.7	40.8
	Agrarian	25.1	29.8	32.3	26.2	22.9	30.3
	Pastoral	13.7	18.9	12.9	8.2	11.7	9.9
Health facility type	Health center	30.5	33.3	37.7	30.6	32.3	35.0
	Public hospital	49.7	50.9	38.9	45.5	43.7	36.7
Region	Addis Ababa	41.4	44.8	17.2	38.0	44.8	41.4
	Afar	18.7	18.7	21.8	21.8	28.0	12.4
	Amhara	37.8	46.1	47.5	40.4	39.0	40.1
	Benishangul Gumuz	11.2	11.2	11.2	15.5	15.5	15.5
	DD	72.9	72.9	5.4	78.3	78.3	5.4
	Gambela	50.0	70.8	9.7	51.3	70.8	0.0
	Hareri	80.3	80.3	0.0	80.3	80.3	0.0
	Oromia	40.5	38.3	46.6	37.5	37.6	45.8
	SNNP	25.1	29.5	22.2	21.4	17.9	16.1
	Sidama	25.8	22.1	29.4	25.8	25.8	22.1
	Somale	11.2	22.1	25.2	9.8	19.1	25.2
	South West Ethiopia Peoples	14.5	31.3	20.4	12.0	21.6	21.6

Accuracy of reports is also a problem among health posts. The proportion of health posts with accurate reports on the six data elements ranged between 30% and 41%. Some agrarian regions, including Amhara, Harari, Oromia, and Sidama, performed relatively better regarding reporting accuracy among health posts Table 9.14

Table 9.14: The proportion of health posts with acceptable accuracy of reports (Weighted percent) in Ethiopia, 2023

		% of health facilities with accurate reports					
		Pentavalent 3			Measles		
		Month 1	Month 2	Month 3	Month 1	Month 2	Month 3
Total		39.7	41.0	33.8	30.0	37.0	31.4
Setting	Urban	5.9	5.9	5.9	5.9	10.0	5.9
	Agrarian	51.7	53.0	43.8	39.2	47.4	41.0
	Pastoral	8.2	10.5	6.7	3.7	6.7	4.4
Region	Afar	0.0	0.0	8.3	8.3	0.0	8.3
	Amhara	52.7	56.4	52.7	47.3	52.7	45.5
	Benishangul Gumuz	15.4	23.1	30.8	15.4	15.4	30.8
	DD	25.0	25.0	37.5	25.0	37.5	25.0
	Gambela	25.0	16.7	0.0	0.0	16.7	0.0
	Hareri	58.8	52.9	23.5	41.2	35.3	17.6
	Oromia	41.4	41.4	32.9	27.1	41.4	34.3
	SNNP	27.0	29.7	21.6	13.5	16.2	10.8
	Sidama	55.0	55.0	40.0	45.0	40.0	35.0
	Somali	7.1	10.7	7.1	3.6	7.1	3.6
	South West Ethiopia Peoples	15.8	15.8	10.5	10.5	15.8	10.5

Data Completeness

Nationally, almost three-quarters (73.32%) of all assessed health institutions have at least one health post under their jurisdiction, except for Addis Ababa city administration, which does not have a health post. Most health posts (96.82%) submit reports to their respective health centers. One-fifth (20.44%) of the facilities kept a log book or electronic database to track monthly HMIS reports sent by the health posts. Similarly, 54 (19.34%) of health facilities kept both paper and electronic versions of monthly HMIS reports received from health posts.

Most respondents (92.34%) said their facility is expected to send a paper-based HMIS report (service and disease) to the corresponding health center every month. At the same time, other institutions prefer to submit their reports electronically or by mixed means. Computer system failure and power outages are not among the reasons why health posts do not submit monthly HMIS reports.

Report Timeliness

One hundred and eighty health facilities (63.25%) register receipt dates of monthly community health information system (including EPI) reports supplied by health posts, with nearly one-fifth failing to submit their monthly report on time. In the three months preceding the survey, many health posts (15.90%) covered in the national EPI program evaluation did not submit their reports on time Table 9.15.

Table 9.15: Proportion of health facilities with complete and timeliness report by background characteristics in Ethiopia, 2023.

		The proportion of health facilities that:						
		Have at least one health post under its supervision	Submitted reports to the respective Health Facility	Kept log book and electronic database t[illegible] track monthly HMIS reports sent by the H.P.	Kept both copies of monthly HMIS reports	Supposed to submit paper paperbased monthly HMIS reportto the H.C.	Record receipt dates of monthly CHIS reports	Sent reports on time in any of the review periods
Total		73.32	96.82	20.44	19.34	92.34	63.25	15.90
Setting	Urban	55.56	94.17	20.35	23.01	93.81	63.33	16.67
	Agrarian	98.52	100.00	21.80	17.29	96.24	73.68	9.77
	Pastoral	85.71	93.33	14.29	14.29	67.86	16.67	40.00
Health facility type	Public hospital	20.83	93.33	14.29	7.14	92.86	80.00	20.00
	Health center	85.35	97.01	20.77	20.00	92.31	62.31	15.67
Regions	Afar	50.00	100.00	11.11	11.11	66.67	33.33	44.44
	Amhara	72.00	96.30	19.23	3.85	100.00	61.11	9.26
	Benishangul Gumuz	68.75	100.00	36.36	9.09	81.82	81.82	27.27
	Dire Dawa	63.64	100.00	14.29	0.00	85.71	85.71	14.29
	Gambela	80.00	75.00	33.33	0.00	100.00	25.00	62.50
	Harari	75.00	100.00	16.67	0.00	83.33	83.33	16.67
	Oromia	78.57	98.70	27.63	2.63	93.42	72.73	6.49
	SNNP	88.24	100.00	15.56	2.22	97.78	80.00	17.78
	Sidama	91.67	100.00	18.18	4.55	95.45	68.18	13.64
	Somali	66.67	83.33	15.00	10.00	70.00	12.50	33.33
	SWEP	90.91	100.00	10.00	0.00	95.00	55.00	10.00

Data Quality Assurance

The current study assessed data quality by considering different dimensions at different levels. At the facility level, below a quarter of health facilities in the Gambela and Somali regions have no data quality self-assessment tools (e.g., LQAS tool, electronic data quality validation rule/system). All health facilities in the Benshangul-Gumuz, Gambela, Harari, and Sidama regions conducted LQAS in the previous three months of the survey; however, almost a tenth of health facilities in other regions (12.83%) did not. In the country, nearly two-thirds of health facilities (65.28%) kept records of feedback to staff on data quality self-assessment findings Table 9.16.

Table 9.16: Proportion of health facilities that conducted data quality assessment mechanism by to background characteristics in Ethiopia, 2023.

		The proportion of health facilities that have:			
		Data quality self-assessment tools	Conducted LQAS in the reviewed three months	Service report covered by the LQAS in the last quarter of 2015 E.C	Maintained records of feedback to staff on data quality self-assessment findings
Setting	Urban	75.46	88.34	62.96	66.26
	Agrarian	70.37	87.37	60.74	65.26
	Pastoral	20.00	57.14	8.57	42.86
Health facility type	Public hospital	76.39	92.73	63.89	67.27
	Health center	66.88	85.71	55.73	64.76
Region	Addis Ababa	100.00	94.12	94.12	58.82
	Afar	38.89	42.86	11.11	28.57
	Amhara	77.33	84.48	64.00	74.14
	Benshangul Gumuz	43.75	100.00	43.75	42.86
	Dire Dawa	90.91	70.00	63.64	70.00
	Gambela	20.00	100.00	20.00	100.00
	Harari	75.00	100.00	75.00	83.33
	Oromia	86.73	89.41	73.47	62.35
	SNNP	62.75	87.50	54.90	75.00
	Sidama	79.17	100.00	79.17	63.16
	Somali	25.00	88.89	16.67	66.67
	Southwest Ethiopia Peoples	59.09	76.92	36.36	46.15
National		68.65	87.17	57.25	65.28

9.3 Immunization Data Use

The Ethiopian Health Management Information System (HMIS) is designed to collect and disseminate critical core data for health system planning and monitoring. The HMIS information use guideline establishes procedures to ensure data quality, makes use of existing mechanisms of national-level strategic planning for the health sector, and encourages the display of data under the identified priorities to facilitate a bold display of progress and to serve as a constant reminder in the event of poor performance. The guide's overall goal is to increase the use of HMIS information for planning and management at all levels of the health system.

Performance monitoring

At various levels of the health system, the actual use of information for decision-making (or any activity) can be proven in a variety of ways. The availability of an annual plan, performance monitoring charts, and other relevant documents in a health facility/institution could indicate the presence of a data use culture.

All of the evaluated regional health bureaus had a functional performance monitoring (PMT) or management team that meets regularly to monitor the performance of health programs including EPI. However, seven out of ten zones have formed a PMT squad. Moreover, the majority of Woreda health offices (90.48%) have an active performance monitoring team. At the district level, the team met once a month for the previous three months before the survey period in the majority of districts (63.78%) Figure 9.3.

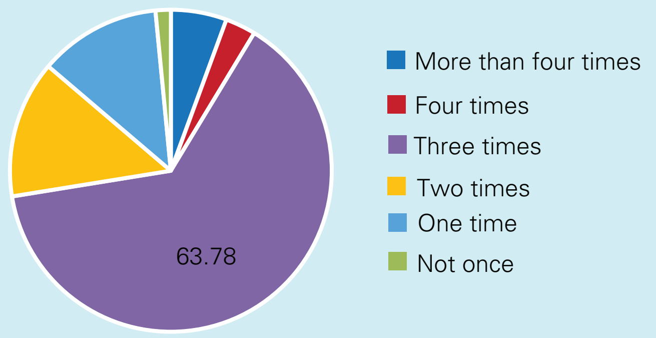

Figure 9.3: Frequency of the performance monitoring team meetings at district level held during the 3rd quarter of 2015 EFY.

The entire region retained minutes of PMT meetings held in the three months preceding the survey period. In the third quarter of the 2015 fiscal year, one-tenth of the zones (12.50%) failed to maintain the minutes. The minutes of the performance monitoring meetings for the third quarter of the 2015 fiscal year were maintained by a large percentage of Woredas (82.65%). The team focused primarily on RHIS data management, as well as EPI data quality attributes like as accuracy, completeness, and timeliness.

At woreda level, the PMT team used RHIS data to compute coverage of health services, including EPI (95.06%), to monitor health key performance indicators on the EPI program (94.44%), to identify emerging issues/epidemics related to the EPI program (71.60%), to identify vaccine stock-outs (74.07%), and to disaggregate data by sex (40.12%) Figure 9.4

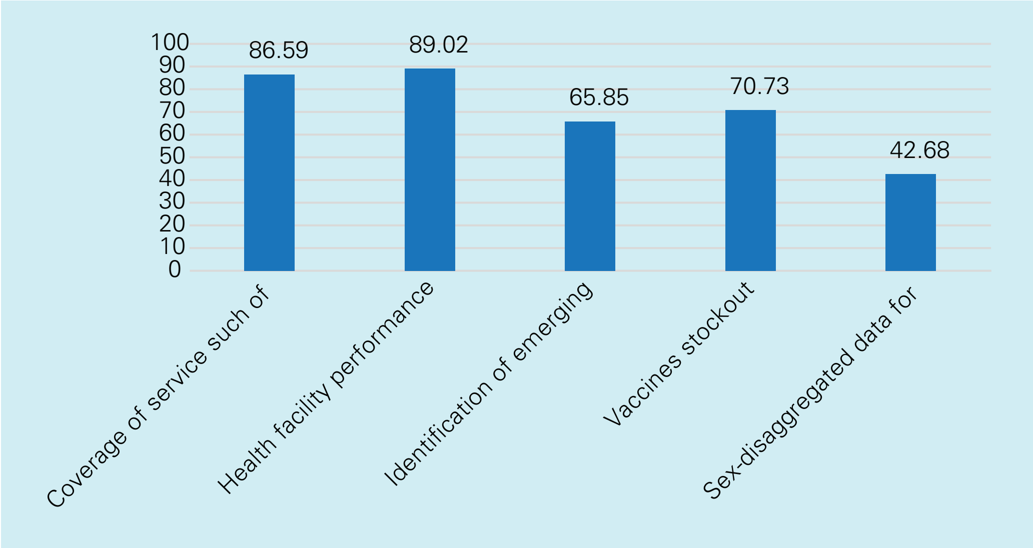

Figure 9.4: Proportion of WoHOs that conducted performance monitoring based on key performance indicators and RHIS data in Ethiopia, 2023.

The PMT team, on the other hand, used the discussion with the district/ sub-city and/or health facility's EPI program performance to make a decision primarily for plan development, vaccine supply and drug management, service quality promotion, and other relevant events Figure 9.5

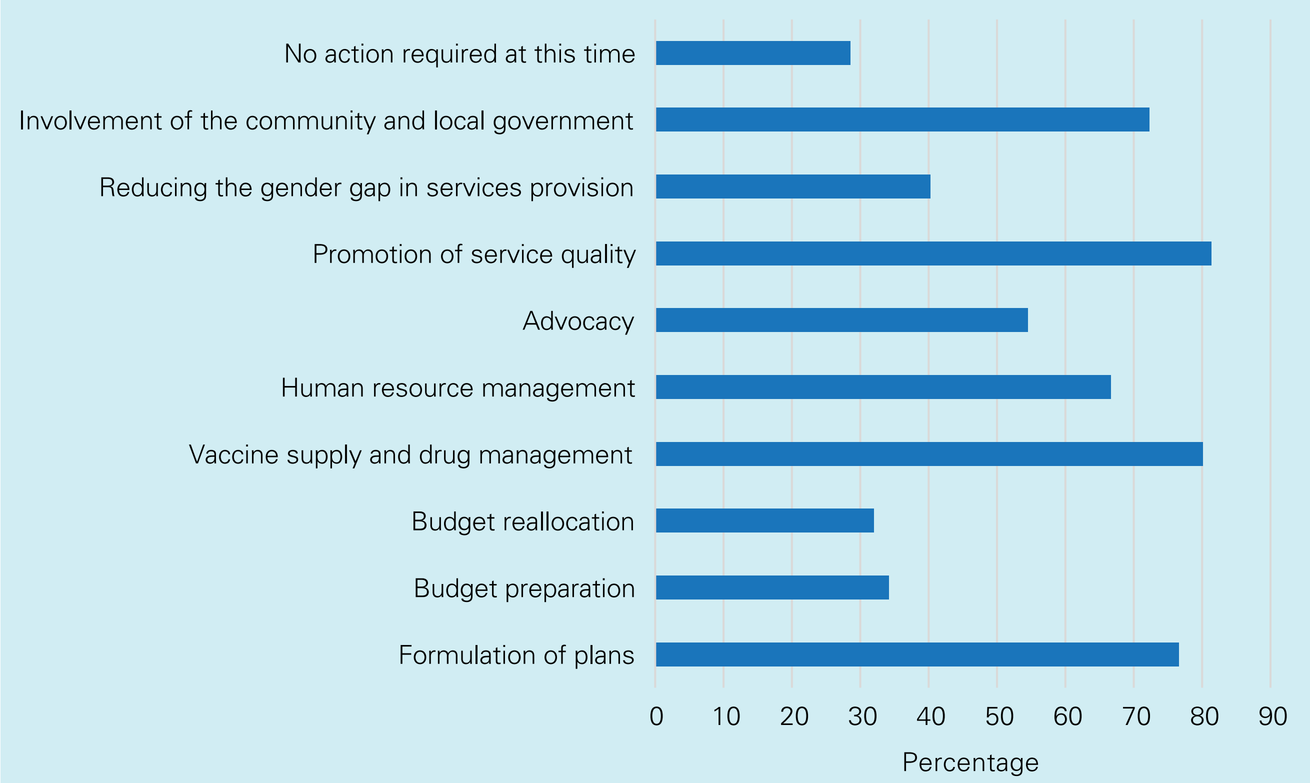

Figure 9.5: Type of decisions made based on the discussion of the district/sub-city and/or health facility's EPI program performance at district level in Ethiopia, 2023..

More than a tenth of district/sub-city heads (87.01%) did not attend any of the performance assessment sessions held in the three months before the survey Table 9.17

Table 9.17: Percentage of Woreda/sub-city that conducted performance monitoring at the district level in by region of Ethiopia, 2023.

		The proportion of Woreda/Sub-city health offices that have:				
		A performance monitoring team	Routine team meetings to discuss performance monitoring including EPI	Maintained meeting minutes three months preceding the survey	Discussed on RHIS data quality	Followup action on the decisions and identified problems
National		90.48	84.85	82.65	95.68	80.25
Region	Addis Ababa	100.00	100.00	100.00	100.00	7.69
	Afar	85.71	78.57	90.91	90.00	10.00
	Amhara	92.86	83.33	85.71	96.67	26.67
	Benishangul Gumuz	100.00	100.00	87.50	100.00	0.00
	Gambela	50.00	50.00	50.00	0.00	0.00
	Harari	75.00	75.00	100.00	66.67	66.67
	Oromia	89.06	82.81	90.57	95.83	25.00
	SNNP	96.77	93.55	96.55	100.00	14.29
	Sidama	93.75	93.75	86.67	92.31	30.77
	Somali	85.71	80.95	17.65	100.00	0.00
	SWEP	85.71	71.43	60.00	100.00	0.00

At the health post level, the performance of the EPI program is monitored by comparing the target (plan) set with the achievement at the end of each reporting period. Almost half of the health posts surveyed (49.06%) used the EPI program performance tracking system. However, based on the results of the performance tracking system, only a few health posts (16.48%) held discussions and identified feasible interventions to enhance the status of the EPI program. Concerning the immunization services session plan, only four out of ten health posts (42.32%) have prepared it based on the institution's micro plan. Nearly one-third of all health posts surveyed (34.08%) employ a defaulter tracing tickler file box or log book Table 9.18

Table 9.18: EPI performance monitoring practices at the health posts level by background characteristics, in Ethiopia 2023.

		The proportion of health posts that:					
		Have annual EPI plan for 2015 EFY	Have an EPI forecasting plan	Have updated EPI monitoring chart	Have an EPI program performance tracking system	Developed EPI services session plan	Use defaulter tracing tickler file box /log book
National		78.28%	45.32%	46.82%	49.06	42.32	34.08
Setting	Urban	73.08	26.92	30.77	46.15	38.46	11.54
	Agrarian	86.46	53.65	51.04	53.65	46.88	40.63
	Pastoral	48.98	22.45	38.78	32.65	26.53	20.41
Region	Afar	45.45	18.18	45.45	18.18	36.36	36.36
	Amhara	89.36	48.94	55.32	57.45	61.70	34.04
	Benishangul Gumuz	75.00	50.00	58.33	16.67	33.33	33.33
	Dire Dawa	87.50	37.50	50.00	100.00	62.50	62.50
	Gambela	55.56	22.22	33.33	44.44	22.22	22.22
	Harari	70.59	35.29	52.94	47.06	11.76	35.29
	Oromia	85.29	54.41	44.12	48.53	55.88	41.18
	SNNPR	84.38	56.25	46.88	56.25	31.25	31.25
	Sidama	85.00	40.00	55.00	55.00	40.00	35.00
	Somali	46.15	23.08	38.46	30.77	19.23	15.38
	South West Ethiopia Peoples	88.24	58.82	29.41	58.82	35.29	29.41

Only, 35 (13.11%) of health posts document the causes for EPI service under- or over-performance. For the 2015 Ethiopian fiscal year, all health posts in the Dire Dawa, Gambela, and Somali regions did not conduct regular analysis, including trends in EPI performance metrics over time. According to this survey, supportive supervision/mentorships focused on EPI data management, data quality, reporting, and utilization in the three months preceding the survey were less common among urban health posts than agrarian health posts. In the third quarter of the 2015 EFY, slightly more than half of the supervised health posts (56.30%) got written feedback from the appropriate supervisory team on EPI data management, data quality, reporting, and use Table 9.19

Table 9.19: EPI performance practice at the health post level by background characteristics, in Ethiopia 2023.

		The proportion of health posts that:							
		Documented procedures to ensure regular review of EPI data	Document reasons for under or over-performance of EPI services	Conducted regular EPI data analysis for 2015 EFY	Have a review meeting for M&E of EPI	Conducted EPI-specific community discussions	Received supportive supervision	Received written feedback on EPI data management	Received written feedback on EPI reports
National		23.22	13.11	16.85	20.60	18.73	44.57	56.30	55.46
Setting	Urban	19.23	7.69	23.08	15.38	19.23	26.92	57.14	57.14
	Agrarian	25.00	15.63	18.75	24.48	21.35	47.40	54.95	51.65
	Pastoral	18.37	6.12	6.12	8.16	8.16	42.86	61.90	71.43
Region	Afar	27.27	18.18	27.27	18.18	9.09	72.73	87.50	100.00
	Amhara	36.17	23.40	36.17	36.17	31.91	53.19	56.00	52.00
	Benishangul -Gumuz	16.67	0.00	8.33	16.67	25.00	50.00	33.33	33.33
	Dire Dawa	12.50	0.00	0.00	12.50	12.50	62.50	80.00	60.00
	Gambela	22.22	0.00	0.00	11.11	11.11	44.44	75.00	75.00
	Harari	5.88	29.41	29.41	29.41	23.53	82.35	57.14	57.14
	Oromia	26.47	11.76	14.71	16.18	19.12	33.82	69.57	65.22
	SNNP**	21.88	15.63	15.63	25.00	18.75	43.75	42.86	50.00
	Sidama	30.00	20.00	10.00	20.00	25.00	40.00	62.50	62.50
	Somali	11.54	0.00	0.00	0.00	0.00	23.08	33.33	33.33
	SWEP*	11.76	0.00	11.76	23.53	5.88	35.29	0.00	0.00

Performance improvement practices

The majority (94.12%) of zonal health departments used immunization data for decision-making. Of these, 93.75% of the decisions were used for service improvement. Two-thirds (66.57%) of the zonal health departments had given routine feedback on immunization performance. Almost half (49.02%) of the surveyed zones had regular meetings with health unit workers on immunization performance and half of the zones conducted review meetings with the units in the last two quarters (2nd and 3rd quarters) Table 9.20

Table 9.20: EPI data analysis and utilization on EPI data management of immunization in Zonal health department, in Ethiopia 2023.

		The proportion of zonal health departments that have:			
		Given routine feedback based on the EPI performance analysis	Regular meetings with health unit workers to discuss EPI performance	Conducted a review meeting with the health units in the last two quarters (2nd & 3rd)	Used EPI data for decision making
All Health departments		66.57	49.02	50.98	94.12
Region	Addis Ababa	-	-	-	-
	Afar	-	-	-	-
	Amhara	76.92	38.46	38.46	100
	Benishangul Gumuz	0	0	0	100
	Dire Dawa	-	-	-	-
	Gambela	33.33	66.67	66.67	66.67
	Harari	-	-	-	-
	Oromia	90.91	63.64	63.64	100
	SNNP	61.54	46.11	61.54	92.31
	Sidama	-	-	-	-
	Somali	33.33	33.33	33.33	83.33
	South West Ethiopia Peoples	75	75	50	100

At the district level, more than half (55.84%) of district /sub-city health offices monitored the health unit's vaccine stock. The highest stock-out monitoring was observed in Oromia (100%) and Benishangul Gumuz (75%), and the lowest in the Somali (28.57%) and Gambela (0%) regions. About five in eight (42.42%) districts and sub-cities conducted regular meetings with health unit workers to discuss EPI performance. Addis Ababa (92.31%) is the highest and lowest in the South West Ethiopian Peoples (21.43%), Afar (21.43%), and Somali (23.81%) regions. The majority (86.58%) of the health offices had immunization data used for decision-making. The highest in Addis Ababa (100%), Gambela (100%), and SNNP (100%) regions Table 9.21.

Table 9.21: EPI data analysis and utilization of immunization data by district/sub-city health offices in Ethiopia, 2023

		The proportion of districts/sub-city that have:				
		Vaccine stock out monitoring of health unit's	Give routine feedback based on the EPI performance analysis	Regular meetings with health unit workers to discuss EPI performance	Conducted review meetings with the health units in the last two quarters (2nd & 3rd)	EPI data used for decision-making
All District/sub city		55.84	51.08	42.42	51.52	86.58
Setting	Urban	60.66	52.46	59.02	52.46	6.56
	Agrarian	61.29	59.68	40.32	57.26	7.26
	Pastoral	41.03	30.77	25.64	35.90	30.77
Region	Addis Ababa	53.85	61.54	92.31	84.62	100
	Afar	50	42.86	21.43	50	85.71
	Amhara	59.52	54.76	38.10	47.62	92.86
	Benishangul Gumuz	75	75	50	37.50	87.50
	Dire Dawa	50	0	0	25	75
	Gambela	0	25	0	25	100
	Harari	68.75	59.38	48.44	60.94	89.06
	Oromia	100	100	100	100	83.33
	SNNP	50	62.50	50	56.25	100
	Sidama	48	40	40	48	88
	Somali	28.57	23.81	23.81	23.81	57.14
	South West Ethiopia Peoples	42.86	35.71	21.43	35.71	71.43

At the health center and hospital level, three-fourths (76.09%) of the health facilities had vaccine receipts and issues recorded in the vaccine ledger book in the last three months. Gambela (19.46%), Dire Dawa (37.97%), and Somalia (39.29%) had the lowest vaccine receipts and issues recorded in the vaccine ledger book. Close to three-fourths (73.12%), (74.48%), and (75.96%) of health facilities updated vaccine ledger books, had sufficient immunization formats, and recorded the correct appointment date in the immunization book of the last individual. More than three-fourths (78.23%) of the health facilities had a stamp or written date of report from the health facilities Table 9.22.

Table 9.22: Immunization supply recoding practice of the health facilities in Ethiopia, 2023.

		The proportion of health facilities that have:					
		Vaccine receipt and issues recorded in vaccine ledger book in the last three months	Current ledger books are up to date for all vaccines reviewed	EPI forms sufficiently available in the visited HF	The last three individual records from EPI register book data are completed for every observed vaccine.	The last three individua records from the EPI register book appointment date are correct	HF stamp or write the date of a report from the HF is received at facility level on the report
Total		76.96	73.12	74.48	78.13	75.96	78.23
Setting	Urban	79.91	75.15	77.96	80.83	77.47	76.75
	Rural	73.13	70.28	69.65	74.37	73.86	80.30
Health facility type	Health center	81.05	75.78	76.03	78.55	76.61	81.07
	Specialized Hospital	82.79	82.79	92.52	92.52	92.52	61.95
	General Hospital	55.97	57.70	78.59	87.47	85.38	56.12
	Primary Hospital	52.40	55.93	54.55	65.87	61.16	71.27
Region	Addis Ababa	100	96.56	100	96.56	89.69	55.16
	Afar	78.13	71.78	62.43	81.21	81.21	74.95
	Amhara	79.92	74.07	68.46	76.25	73.78	74.90
	Benishangul Gumuz	88.80	88.80	84.51	95.71	95.71	88.80
	Dire Dawa	37.99	32.57	43.42	94.57	83.72	83.72
	Gambela	19.46	9.73	100	59.73	30.54	50
	Harari	93.42	93.42	93.42	93.42	100	93.42
	Oromia	81.76	71.50	82.79	82.59	78.55	86
	SNNP	61.50	56.19	59.73	65.04	63.27	76.64
	Sidama	79.44	88.96	68.40	72.08	79.44	81.60
	Somali	39.29	48.57	58.10	59.53	51.67	64.05
	South West Ethiopia Peoples	61.54	61.54	65.12	91.93	85.51	63.86

At the health post level, less than half (47.27%) of the health posts had updated immunization monitoring charts. Somalia (34.62%) and Afar (36.36%) were the lowest regions in immunization monitoring. Less than two in ten (16.63%) and 13.60% of health posts had written clear instructions or guidance on how to complete immunization data, vaccine receipts, and issues recorded in the vaccine ledger book in the last three months, respectively. About one in ten (11.28%) of health posts had updated their ledger book for all vaccines. Only 7.07% of health posts had vaccine stock-out monitoring. Nearly two out of five (43.53%) health posts correctly and completely recorded individual children's immunization information Table 9.23

Table 9.23: Immunization performance monitoring and vaccine balance recording of health Posts in Ethiopia, 2023.

		The proportion of health posts that have:					
		Updated EPI monitoring chart /table display EPI performance for completing months of 2015 E.C EPI (3rd quarter)	Written clear instructions/ guidance on how to complete all EPI data	Vaccine receipt and issues recorded in vaccine ledger book in the last three months	Current Ledger books Up to date For all vaccines	Vaccine stockout monitoring	Individual (children) information is correctly and completely recorded
Total		47.27	16.63	13.60	11.28	7.07	43.53
Setting	Urban	52.30	24.63	16.47	10.02	8.69	55.97
	Agrarian	48.36	16.36	13.33	11.7	7.46	43.49
	Pastoral	35.46	10.66	12.89	9.6	3.06	32.77
Region	Addis Ababa						
	Afar	36.36	27.27	18.18	18.18	9.09	36.36
	Amhara	82.98	28.89	14.59	8.51	8.51	51.06
	Benishangul Gumuz	91.67	8.33	8.33	8.33	0	41.67
	Dire Dawa	100	0	0	0	0	12.50
	Gambela	66.67	42.86	22.20	11.11	11.11	55.56
	Harari	58.82	0	0	0	0	11.76
	Oromia	83.82	20.63	16.18	14.79	8.82	52.94
	SNNP	81.25	0	25	12.5	9.38	50
	Sidama	60	5.26	5	10	5	25
	Somali	34.62	3.85	11.54	7.69	0	26.92
	South West Ethiopia Peoples	70.59	0	11.76	0	5.88	17.65

In the three months preceding the survey, nearly half of the randomly selected districts in the country (53.25%) were supervised. However, less than four out of ten health offices (38.10%) supplied EPI-specific feedback for the woreda's lower-level units. On the other hand, within the first three months of the national EPI program assessment period, half of the woredas (48.48%) held an EPI-focused/integrated review meeting Figure 9.6

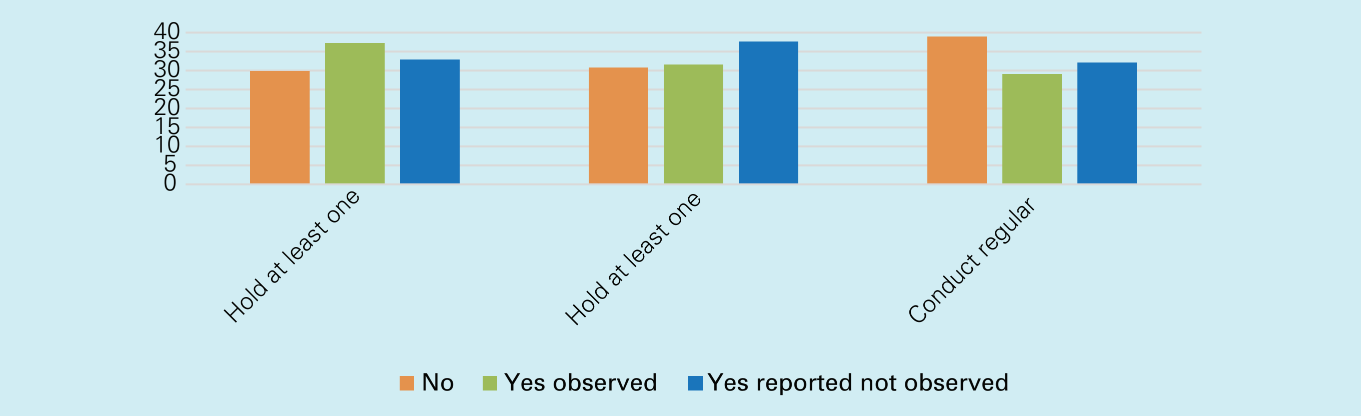

Figure 9.6: Proportions of Woredas conducted regular analysis and held data review meeting in Ethiopia, 2023.

On the contrary, slightly more than one-third of woredas (38.10%) have produced EPI-specific quality improvement/projects/action plans in the recent year. Sixty-seven (29.00%) districts analyzed HMIS/EPI data and looked at trends in performance indicators over time, disaggregated by gender and/or age, geography, and other characteristics Table 9.24.

Table 9.24: Percentage of Woreda/sub-city that practiced data use of the EPI program by region of Ethiopia, 2023.

		The proportion of Woreda/Sub city health offices that:						
		Have an established PMT for the EPI program	Conducted EPI-specific or integrated supportive supervision	Conducted EPI-focused or integrated review meeting	Developed the EPI specific QI/projects/ action plans	Provided the EPI specific feedback for the lower level units	Documented procedures to ensure regular review of EPI data	Document reasons for under- or over-performance
Total		48.48	53.25	48.48	38.10	38.10	39.83	38.96
Region	Addis Ababa	69.23	100.00	92.31	23.08	23.08	76.92	69.23
	Afar	28.57	28.57	28.57	28.57	28.57	14.29	28.57
	Amhara	52.38	61.90	57.14	38.10	38.10	45.24	52.38
	Benishangul Gumuz	37.50	62.50	62.50	62.50	62.50	50.00	25.00
	Gambela	50.00	25.00	25.00	50.00	50.00	0.00	0.00
	Harari	50.00	0.00	0.00	0.00	0.00	25.00	25.00
	Oromia	51.56	60.94	56.25	46.88	46.88	50.00	50.00
	SNNP	45.16	58.06	35.48	38.71	38.71	32.26	29.03
	Sidama	75.00	62.50	56.25	37.50	37.50	50.00	50.00
	Somali	23.81	14.29	23.81	28.57	28.57	9.52	4.76
	SWEP	42.86	28.57	35.71	28.57	28.57	28.57	14.29

At the health post level, supportive supervision/mentorships focused on EPI data management, data quality, reporting, and utilization in the three months preceding the survey were less common among urban health posts than agrarian health posts Figure 9.7.

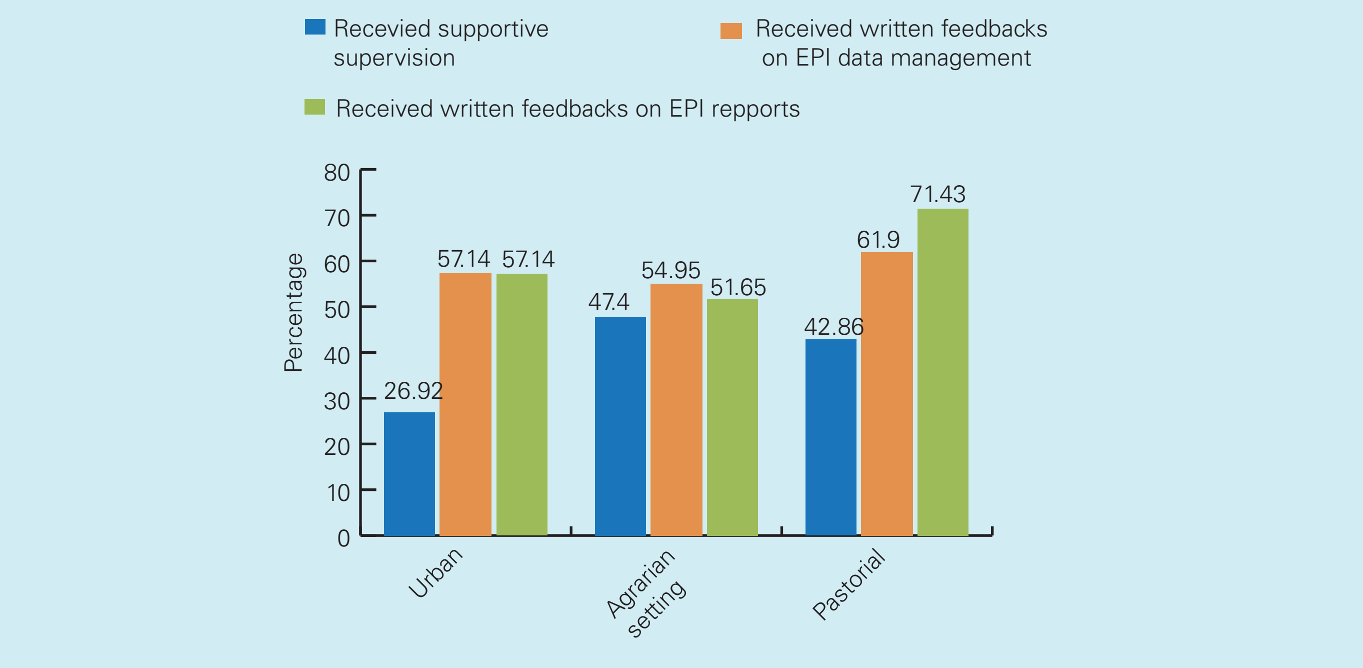

Figure 9.7: Proportion of HPs that got supportive supervision in the 3rd quarter of 2015 EFY in Ethiopia, 2023.

EPI Data Processing and Analysis

Nationally, two-thirds (66.84%) of health institutions employ an electronic database or system to manage routine health information systems including EPI data. For data entry, the majority of health institutions (63.47%) employ a national open-source data processing system (e.g., DHIS 2), followed by an Excel-based spreadsheet (11.14%). Those institutions, on the other hand, that use a national open-source data processing system for data entry also employ the same system for data analysis (63.47%)
Figure 9.8.

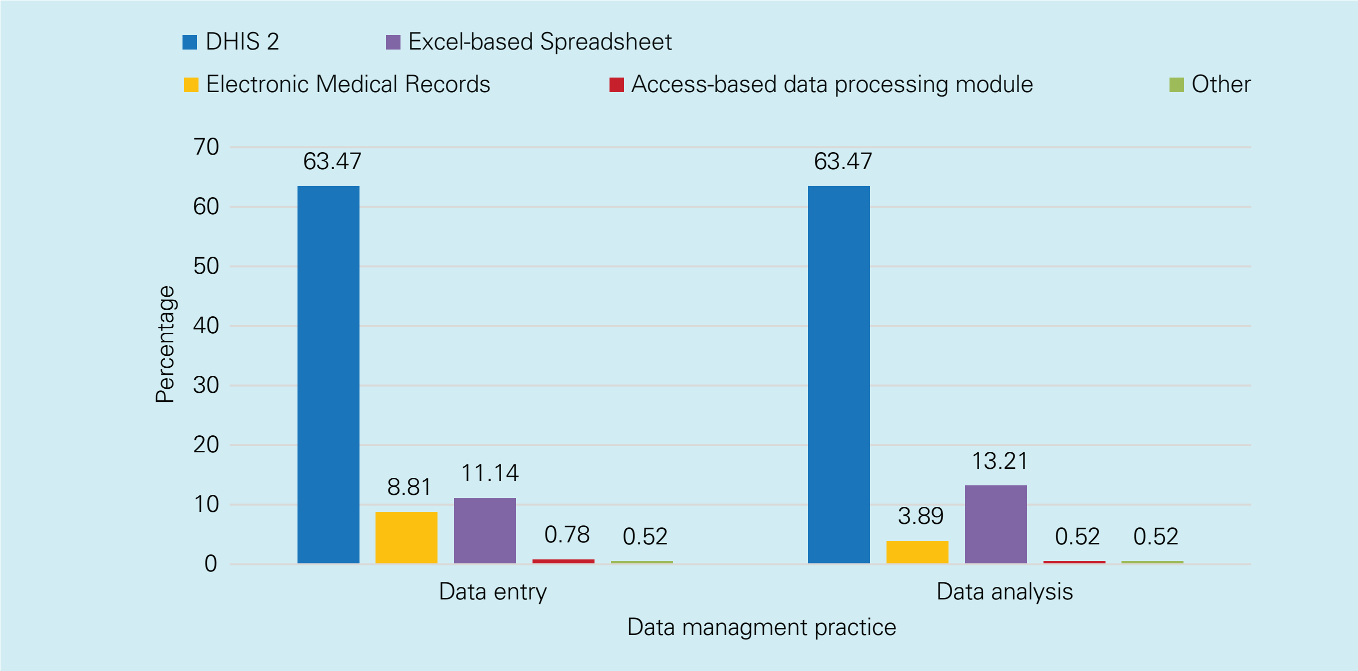

Figure 9.8: Electronic systems used by the health facilities for EPI data entry and analysis in Ethiopia, 2023.

The majority of health facilities that conducted data analysis (83.94%) had aggregated HMIS reports. The presence of demographic data on the catchment population of the health facility for calculating coverage is confirmed in the majority of health institutions (82.90%). When compared to other regions, all health facilities in the Dire Dawa and Harari regions included in the evaluation calculated indicators (e.g., Penta3 coverage) for the health facility catchment area in the third quarter of 2015 EFY

In addition, comparisons were made between health facility and woreda/national targets for immunization services given by facilities in half of the total facilities examined for the study (50.52%). Almost four out of ten health facilities (41.97%) did not do cross-time data comparisons (for example, for Penta3). Furthermore, none of the health facilities in Dire Dawa city administration and one-tenth of the health facilities in Gambela region disaggregated immunization data (for example, for Penta3) by gender, but the national coverage is less than one-third (29.79%). When comparing immunization service coverage among related immunization services (for example, PCV1 and Penta1), there is no difference between health facilities in urban and rural settings Table 9.25

Table 9.25: Proportion of health facilities that conducted data processing and analysis by background characteristics in Ethiopia, 2023

		The proportion of health facilities that:							
		Used an electronic system for routine data managemer	Aggregatec or summar HMIS report	Kept demograph data on the catchment population of the HF	Calculated EPI indicators for the HF catchment are in the third quarte (2015 E.C)	Done comparisor b/n HF & Woreda /national targets related to EPI services	Performed comparison of EPI data over time	Conducted comparisons of sex-disaggregated EPI data	Done comparisons of service coverage b/n associated EPI services
Total		66.84	83.94	82.90	76.68	50.52	58.03	29.79	59.84
Setting	Urban	73.61	86.57	84.72	75.93	49.07	60.65	31.02	62.50
	Agrarian	63.70	86.67	85.19	80.74	57.78	59.26	28.89	62.22
	Pastoral	37.14	57.14	62.86	65.71	31.43	37.14	25.71	34.29
Health facility type	Public hospital	63.69	87.50	72.22	65.28	33.33	51.39	25.00	52.78
	Health center	80.56	83.12	85.35	79.30	54.46	59.55	30.89	61.46
Region	Addis Ababa	70.59	82.35	94.12	64.71	47.06	47.06	35.29	52.94
	Afar	55.56	72.22	83.33	72.22	50.00	66.67	33.33	55.56
	Amhara	78.67	92.00	85.33	76.00	62.67	72.00	33.33	78.67
	B/Gumuz	75.00	75.00	93.75	68.75	37.50	43.75	12.50	62.50
	Dire Dawa	90.91	100.00	90.91	100.00	36.36	90.91	0.00	36.36
	Gambela	70.00	70.00	50.00	50.00	20.00	20.00	10.00	20.00
	Harari	87.50	100.00	87.50	100.00	25.00	75.00	37.50	75.00
	Oromia	80.61	93.88	91.84	83.67	61.22	66.33	36.73	66.33
	SNNP	54.90	72.55	72.55	74.51	45.10	35.29	23.53	56.86
	Sidama	45.83	91.67	83.33	75.00	58.33	62.50	29.17	58.33
	Somali	33.33	58.33	69.44	75.00	41.67	50.00	33.33	38.89
	SWEP	50.00	81.82	72.73	68.18	22.73	40.91	22.73	40.91

Immunization data presentation

Except for Benishangul Gumuz, all other regions in the country have HMIS information use guidelines. Conversely, four out of every ten zonal health departments (41.18%) do not have access to the guidelines. In general, HMIS information use guidelines were more widely available in woredas in Oromia, SNNPR, and Amhara regions than elsewhere in Ethiopia. Four out of ten woreda health offices (41.56%) have written guidelines on routine health information system (RHIS) information display, use, and feedback.

Every regional health bureau in Ethiopia has maintained copies of the national HMIS/HIS strategic plan and annual plans, but only 60% of the zones have kept them. The national RHIS strategic plans, Woreda/-sub-city yearly plans, and/or Woreda/sub-city performance targets were available in half of the district offices (54.98%), at the district level. In addition, approximately two-thirds of the zonal health department (64.71) and district offices (66.23%) created graphs, tables, maps, and other visual aids to track the immunization program's progress toward the stipulated goals.Seven of the twelve regions (58.33%) can access analyzed electronic immunization data in the form of summary tables, charts, and maps. However, about half of the zonal health departments (50.98%) do not have access to the analyzed electronic data. At the woreda level, more than half of the country's districts (55.41%) have access to analyzed immunization data, which includes summary tables, charts, and maps.

Nationally, two regions, Harari and Dire Dawa failed to provide immunization program feedback reports to health units under their supervision during the third quarter of the 2015 EFY. Similarly, less than a tenth of the country's zones (7.84%) sent feedback to their respective woreda health offices during the aforementioned quarter of the year. In addition, four out of ten district offices (41.56%) submitted immunization program feedback reports for facilities under their control Table 9.26

Table 9.26: Percentage of Woreda/sub-city that practiced data use of the EPI program by region of Ethiopia, 2023. .

		The proportion of Woreda/Sub city health offices that:					
		Have written guidelines on RHIS information display, use, and feedback	Have copies of the national RHIS strategic plans, Woreda/sub-city annual plans, and/or performance targets	Prepare data visuals showing achievements toward targets for EPI	Have access to analyzed immunization data	Produce any report or bulletin for EPI program based on an analysis of RHIS data	Send feedback reports for EPI using RHIS information to health facilities in the past three months
National		41.56	54.98	66.23	55.41	23.38	41.56
Region	Addis Ababa	69.23	84.62	84.62	30.77	61.54	84.62
	Afar	35.71	71.43	71.43	85.71	42.86	7.14
	Amhara	33.33	47.62	71.43	54.76	35.71	45.24
	Benishangul-Gumuz	37.50	50.00	87.50	50.00	12.50	50.00
	Gambela	25.00	75.00	25.00	50.00	0.00	0.00
	Harari	50.00	0.00	75.00	50.00	0.00	25.00
	Oromia	48.44	60.94	71.88	54.69	18.75	51.56
	SNNP	38.71	45.16	70.97	61.29	22.58	38.71
	Sidama	37.50	56.25	50.00	62.50	6.25	56.25
	Somali	23.81	38.10	33.33	38.10	14.29	9.52
	SWEP	57.14	64.29	57.14	64.29	7.14	28.57

Data Dissemination

In the Ethiopian health system, the Woreda health office is expected to submit its report vertically to the respective zonal health department and horizontally to the woreda council. In the current survey, all regions submitted immunization reports to their respective regional councils or administrations, although nearly one-fifth of the zonal health departments (19.61%) did not. Almost one-fifth of the Woreda health offices (19.91%) did not submit their reports to a Woreda council in the third quarter of 2015 EFY. A quarter of woredas (25.97%) shared immunization performance data with the general public through bulletin boards, chalkboards, and/or local publications. The majority of the EPI department at the Woreda level discussed EPI program performance and data quality (75.76%), but only one-quarter of the woredas (25%) in the Harari region did the same Table 9.27

Table 9.27: Percentage of Woreda/sub-city that disseminated data outside the health sector by region of Ethiopia, 2023.

		The proportion of Woreda/Sub city health offices that:						
		Submitted immunization reports to their Woreda in the last 3 months preceding the survey	Submit immunization reports to a Woreda council in the past one year	Use data from the RHIS to assess the immunization program progress	Updated a website at least annually for accessing the district EPI data	Shared woreda immunization performance data with the general public	Updated performance monitoring charts for the immunization program	Made discussion related to EPI program performance and data quality
National		80.09	98.92	91.89	17.75	25.97	70.56	75.76
Region	Addis Ababa	100.00	92.31	92.31	15.38	30.77	30.77	92.31
	Afar	50.00	100.00	100.00	28.57	35.71	35.71	78.57
	Amhara	80.95	97.06	88.24	14.29	19.05	19.05	73.81
	Benishangul Gumuz	75.00	100.00	100.00	0.00	0.00	0.00	75.00
	Gambela	100.00	100.00	100.00	25.00	25.00	25.00	75.00
	Harari	75.00	100.00	100.00	0.00	0.00	0.00	25.00
	Oromia	82.81	100.00	94.34	18.75	29.69	29.69	79.69
	SNNP	83.87	100.00	96.15	9.68	32.26	32.26	80.65
	Sidama	87.50	100.00	92.86	18.75	25.00	25.00	100.00
	Somali	66.67	100.00	71.43	33.33	19.05	19.05	47.62
	SWEP	78.57	100.00	90.91	21.43	35.71	35.71	64.29

9.4 Conclusions

The findings show that the expanded program of immunization is backed up by robust monitoring and evaluation mechanisms in all regions, as well as a strong commitment to setting vaccination targets and determining infant vaccination proportions across regions, zones, districts, and health facilities. However, there are significant gaps in updating immunization plans, ensuring uniformity in program denominators, and providing appropriate data management training. Despite the fact that most regions and zones have people dedicated to data entry and quality reviews, health posts lack specialized data quality reviewers.

The report highlights varying degrees of data utilization practices across different levels of the health system, with some regions and facilities demonstrating commendable efforts in maintaining updated monitoring charts, conducting routine feedback sessions, and utilizing immunization data for decision making.However, there are notable disparities among regions, with some facing challenges in meeting reporting timelines and maintaining data completeness.

While most regional health bureaus have active performance monitoring teams, there is room for improvement in ensuring regular attendance at performance assessment sessions and enhancing the use of performance tracking systems to identify interventions for program improvement. Disparities exist in resource allocation and access to information use guidelines across different regions and levels of the health system. Regions like Addis Ababa, Benishangul Gumuz, Oromia, and Amhara demonstrate higher levels of immunization data utilization, while regions like Somalia, Gambela, and SNNP face challenges in maintaining data quality and timely reporting

Disparities exist in resource allocation and access to information use guidelines across different regions and levels of the health system. Regions like Addis Ababa, Benishangul Gumuz, Oromia, and Amhara demonstrate higher levels of immunization data utilization, while regions like Somalia, Gambela, and SNNP face challenges in maintaining data quality and timely reporting.

9.5 Recommendations and implications

- Adapt/develop digital systems for immunization program monitoring and evaluation system (performance monitoring, supportive supervision, real time campaign monitoring)
- Address the shortage of data management staff at all levels of the health system and invest in training programs to build the skills of existing staff.
- Policy interventions should prioritize improving micro plan updates and its implementation at the district/sub-city and facility level to achieve service broader coverage which can be enhanced by solving the denominator issue through census or eCHIS based head count.
- Strengthening and ensuring the continuous implementation of EPI case team level performance monitoring and reviews
- Addressing regional disparities, strengthening data utilization practices, enhancing capacity-building efforts, and promoting standardized guidelines for information use can contribute to more effective decision-making processes and improved health outcomes for the EPI program.
- Besides, by focusing on improving data completeness, timeliness, and feedback mechanisms across all levels of the health system, stakeholders can work towards achieving more efficient resource allocation and better health service delivery
- Moreover, improving access to guidelines and ensuring consistent data analysis practices can further enhance decision-making processes within the health system.

References

1. World Health Organization, UNICEF, Ethiopia Federal Ministry of Health. Ethiopia National Expanded Programm on Immunization. 2010; (July

2. WHO G. Ethiopia Launches Measles Vaccine Second Dose (MCV2) Introduction: Over 3.3 million children will receive the vaccine annually. 2020.

3. UNICEF. Joint WHO-UNICEF-ROTARY statement on World Polio Day.

4. WHO. Immunization agenda 2030. 2022;1–58. Available from: https://www.who.int/immunization/ia2030_Draft_One_English.pdf?ua=1

5. Geweniger A, Abbas KM. Childhood vaccination coverage and equity impact in Ethiopia by socioeconomic, geographic, maternal, and child characteristics. Vaccine. 2020 Apr 29;38(20):3627–38

6. Ministry Of Health of Ethiopia. Health Sector Transformation Plan. HSTP. 2015. Available from: https://www.healthynewbornnetwork.org/hnn-content/uploads/HSDP-IV-Final-Draft-October-2010-2.pdf

7. UNICEF. Joint WHO-UNICEF-ROTARY statement on World Polio Day. Available from: https://www.unicef.org/ethiopia/press-releases/who-unicef-and-rotary-reaffirm-their-commitment-sustain-africas-polio-free-status

8. UNICEF, WHO. Ethiopia Attains Maternal and Neonatal Tetanus Elimination.

9. Central Statistical Agency. Ethiopia DHS 2000. 2001;1–299. Available from: http://www.dhsprogram.com/pubs/pdf/FR118/FR118.pdf

10. Ethiopian Public Health Institute (EPHI), ICF. Ethiopia Mini Demographic and Health Survey 2019: Final Report. 2021.

11. Central Statistical Agency (CSA), ICF. Ethiopian Demographic and Health Survey 2016. Addis Ababa, Ethiopia, and Rockville, Maryland, USA: CSA and ICF. 2016.

12. Duijzer LE, van Jaarsveld W, Dekker R. Literature review: The vaccine supply chain. Eur J Oper Res. 2018 Jul 1;268(1):174–92.

13. Ethiopian Ministry of Health. Health Sector Transformation Plan II 2020/2021-2024/2025. Ethiop Minist Heal. 2021; 25 (February):1–128.

14. Goel NK, Pathak R, Galhotra A, Dankal C, Swami HM. Status of cold-chain maintenance in Chandigarh. Indian J Public Health. 2008; 52(1):37–9.

15. Momose H, Mizukami T, Ochiai M, Hamaguchi I, Yamaguchi K. A New Method for the Evaluation of Vaccine Safety Based on Comprehensive Gene Expression Analysis. He YO, editor. J Biomed Biotechnol [Internet]. 2010;2010:361841.

16. World Health Organization. State of the world's vaccines and immunization. 2009;

17. WHO. Vaccine quality, efficacy and safety. [Cited 2023 Mar 9].

18. World Health Organization. Vaccines and Biologicals Vaccines and Biologicals Ensuring the quality of vaccines. 2002.

19. Gratton G. Monitoring and Evaluation: Systems Assessment. Initial Air worth. 2018; (August):315–35.

20. Ineza L, Bechtold K, Mwisongo A, Kwedi Nolna S, Linnander EL. Building leadership and management competencies of national immunization teams in 16 Gavi-eligible countries through the EPI leadership and management programme. Vaccine. 2022 Jun 9;40(26):3581–7.

21. UNICEF S the children. Ethiopia: Measles and cholera outbreaks pose deadly threat to already malnourished children. 2023. Available from:
https://reliefweb.int/report/ethiopia/ethiopia-measles-and-cholera-outbreaks-pose-deadly-threat-already-malnourished-children

22. WHO G. Ethiopia Launches Measles Vaccine Second Dose (MCV2) Introduction: Over 3.3 million children will receive the vaccine annually. 2020. Available from:
https://www.afro.who.int/news/ethiopia-launches-measles-vaccine-second-dose-mcv2-introduction-over-33-million-children-will

23. Tsegaye G, Gezahegn Y, Tesfaye A, Mulatu G, Bulcha GG, Berhanu N. Measles outbreak investigation in Guradamole District of bale zone, south Eastern Ethiopia, 2021. Infection and drug resistance. 2022 Jan 1:669-83.

24. Tilahun B, Teklu A, Mancuso A, Endehabtu BF, Gashu KD, Mekonnen ZA. Using health data for decision-making at each level of the health system to achieve universal health coverage in Ethiopia: the case of an immunization programme in a low-resource setting. Heal Res Policy Syst. 2021;19(2):48. Available from:
https://doi.org/10.1186/s12961-021-00694-1

25. FMoH Ethiopia. FMOH. Service Availability and Readiness Assessment (SARA) Implementation guide: An annual monitoring system for service delivery In EPHI,WHO, Geneva, Switzerland: World Health Organization. 2016.

26. WHO. Vaccine quality, efficacy and safety. [Cited 2023 Mar 9]. Available from:
https://www.who.int/europe/news-room/fact-sheets/item/vaccine-quality-efficacy-and-safety

27. Nagata JM, Epstein A, Ganson KT, Benmarhnia T, Weiser SD. Drought and child vaccination coverage in 22 countries in sub-Saharan Africa: A retrospective analysis of national survey data from 2011 to 2019. PLoS Med [Internet]. 2021;18(9):1–15. Available from: http://dx.doi.org/10.1371/journal.pmed.1003678

28. Asrade G, Alemu K, Gebeye E, Walle M, Teklu A, Mekonnen ZA, et al. The Burden of Internal Conflict on Expanded Programs on Immunization in Northwest Ethiopia: Implementation Science Study. Ethiop J Heal Dev. 2021;35(3):39–48.

29. Bangura JB, Xiao S, Qiu D, Ouyang F, Chen L. Barriers to childhood immunization in sub-Saharan Africa: A systematic review. BMC Public Health. 2020;20(1):1108. Available from:
https://doi.org/10.1186/s12889-020-09169-4

30. Feyisa D, Ejeta F, Aferu T, Kebede O. Adherence to WHO vaccine storage codes and vaccine cold chain management practices at primary healthcare facilities in Dalocha District of Silt'e Zone, Ethiopia. Trop Dis Travel Med Vaccines. 2022;8(1):10. Available from:
https://doi.org/10.1186/s40794-022-00167-5

31. WHO. Vaccine quality, efficacy and safety. [Cited 2023 Mar 9]. Available from:
https://www.who.int/europe/news-room/fact-sheets/item/vaccine-quality-efficacy-and-safety

32. Argaw MD, Desta BF, Tsegaye ZT, Mitiku AD, Atsa AA, Tefera BB, et al. Immunization data quality and decision making in pertussis outbreak management in southern Ethiopia: a cross sectional study. Arch Public Heal. 2022;80(1):49.

33. Bernardi FA, Alves D, Crepaldi NY, Yamada DB, Lima VC, Lopes Rijo RPC. Data Quality in health research: a systematic literature review. medRxiv. 2022 Jan 1;2022.05.31.22275804.

34. WHO. World Health Organization Vaccination Coverage Cluster Surveys: Reference Manual. (WHO/IVB/1809) Licence: CC BY-NC-SA 30 IGO. 2018

ADDIS ABABA UNIVERSITY

UNIVERSITY OF GONDAR

HARAMAYA UNIVERSITY

HAWASSA UNIVERSITY

JIMMA UNIVERSITY

ጤና ሚኒስቴር - ኢትዮጵያ
MINISTRY OF HEALTH-ETHIOPIA

amref health africa

World Health Organization

unicef

JSI

PATH
