# Supplementary material for: Spatial disparities in zero-dose vaccination coverage for children aged 12–23 months in Ethiopia: A geographically weighted regression analysis
Source: PLoS One. 2025 Sep 11;20(9):e0332162. doi: 10.1371/journal.pone.0332162 (PMC12425285; doi:10.1371/journal.pone.0332162)
Supplement: Attachment — Submitted filename: PLOS One Review_8April2025.docx [file pone.0332162.s002.docx]

*Spatial Disparities in Zero-Dose Vaccination Coverage for Children Aged 12-23 Months in Ethiopia: A Geographically Weighted Regression Analysis*

Synopsis: Using data from Ethiopia’s most recent national immunization survey, the authors examined the prevalence and geographic distribution of zero-dose children. Zero dose children are defined as children not having received the first dose of the diphtheria, tetanus, pertussis vaccine. Results from this study indicate that the prevalence of zero-dose children is 25% and spatial analyses identified statistically significant clusters of zero-dose children. Other results suggested that no antenatal care utilization, poor perception of benefits of immunization, no maternal tt/td vaccination and perception of the distance of the nearest facility as a big problem were significant predictors of zero-dose status. Overall, the authors highlight that contextual and social factors are crucial to consider for immunization programs, and that tailored approaches are needed.

Overall comments: The methods are sound; however, the manuscript would greatly benefit from editing for clarity and grammatical accuracy.

Major comments:

- Methods
  - The authors write that the OLS was used as a baseline model with classical assumptions of linear regression such as multicollinearity. Did the authors omit a word and in fact meant no multicollinearity? The assumptions of linear regression are linearity, homoscedasticity, independence (of error), normality and no multicollinearity.
  - How was vaccination status assessed? Was verbal report of receipt of DTP-vaccine by the caregiver accepted or was status based only on card confirmation?
- Results
  - The authors should present column percents and not row percents in Table 1
  - Please include total n in Table 1 (for yes zero-dose and no zero-dose). Additionally, please include a row for missing for the descriptive characteristics (e.g. n = 1,359 for perception of immunization benefit (poor/good) vs 1,537 for place of residency (urban/rural) among zero-dose children)

Minor comments:

- The authors write that they used data from a recent immunization program evaluation. Do the authors have information on when the immunization program evaluation was conducted? The data were accessed in 2024 but when was the survey conducted? If so, please include in the methods section.
- Line 27 – 29: The sentence is not grammatically correct and is too long. The reviewer suggests that the authors separate the sentence into two sentences. Example: “Though Ethiopia has made significant progress in childhood vaccination, many children remain unvaccinated, making it the third largest contributor to the global burden of zero-dose children. Zero-dose children are those children who have not received the first dose of diphtheria, tetanus and pertussis containing vaccine.”
- Line 82 –	There is a typo in this sentence : “highest number of for un-…”
- Line 83 – This sentence is grammatically incorrect : “…children are estimated to haven’t received…”
- Line 102 – there is a typo in this sentence (“evidences”)
- Line 102 – there is a typo in this sentence.
- Line 212 – 212 : Error – reference source not found
- Line 345 – There is a typo in this sentence
- Line 348 – there is a typo in this sentence “ the coefficients from your GWR model”
