# Supplementary material for: Spatial disparities in zero-dose vaccination coverage for children aged 12–23 months in Ethiopia: A geographically weighted regression analysis
Source: PLoS One. 2025 Sep 11;20(9):e0332162. doi: 10.1371/journal.pone.0332162 (PMC12425285; doi:10.1371/journal.pone.0332162)
Supplement: Attachment — Submitted filename: Response to Reviewers.docx [file pone.0332162.s006.docx]

**Editorial Board**

**PLOS One**

The authors would like to thank the editorial team and team of reviewers for the constructive and valuable comments. The comments of the editor and the reviewer were highly insightful and enabled us to greatly improve the quality of our manuscript. In this revised manuscript we made substantial changes to address your concerns and comments in a point-by-point response. The changes addressed by the authors are marked with track changes throughout the revised manuscript. We tried to address issues raised and the authors are pleased to submit the revised manuscript titled **‘Spatial Disparities in Zero-Dose Vaccination Coverage for Children Aged 12-23 Months in Ethiopia: A Geographically Weighted Regression Analysis’** for publication. Authors are very grateful for your absolute contributions in improving the quality of the manuscript. We are very keen to incorporate any further comments, if any, for the betterment of the final manuscript.

| **S.No** | **Comments** | **Point by point Responses** |
| --- | --- | --- |
| **Academic Editor** | |  |
| **Journal Requirements** | We note that your Data Availability Statement is currently as follows: [All relevant data are within the manuscript and its Supporting Information file.] Please confirm at this time whether or not your submission contains all raw data required to replicate the results of your study. Authors must share the “minimal data set” for their submission | Thank you so much for the comment. We addressed the comment. The dataset is included in the submission and also added as a caption in the revised manuscript. Refer on page 25, line number 610-612 of the trach version |
|  | We note that the grant information you provided in the ‘Funding Information’ and ‘Financial Disclosure’ sections do not match. | Sorry for the inconvenience. Issue addressed. |
|  | We note that the grant information you provided in the ‘Funding Information’ and ‘Financial Disclosure’ sections do not match. | Thanks for the comment. We addressed accordingly |
|  | You may seek permission from the original copyright holder of Figures 3-7 to publish the content specifically under the CC BY 4.0 license. | Thanks for your comment. The shapefiles are sourced from <https://data.humdata.org/dataset/cod-ab-eth>. which is an open platform, in which the data is publicly available for anyone to use. |
| **General comment:** | Please check every line and sentence for language clarity and typos and amend accordingly. | Thanks for your comment. We tried to address the grammatical and typos errors throughout the manuscript. |
| **Specific comments:** | Move line 115 to 121 (put it under the data sources). | Thank you for the comment. We moved line 115 to the recommended place. Refer on page 6 line number 122-123 |
| **Methods section** | Expand the data source section - who conducted the national immunization survey? When was it conducted? Who was/were the participants? How many? Did the survey use a representative sample? And include any pertinent information about the survey. Cite references for the data source. | Thank you so much for the comments. We tried to expand the data source section. Refer on page 6 line number 122-136. The number of children included in the analysis is mentioned in the main document on page 7 line number 148. |
|  | Did the survey use a representative sample? And include any pertinent information about the survey. Cite references for the data source. | Thanks for this. Yes. The survey used a representative sample conducted across all regions of Ethiopia except Tigray region.  Regarding the pertinent information, the survey included service delivery, Supply Chain Management, Adverse Events Following Immunization (AEFI), Vaccine Preventable Diseases Surveillance, Leadership, Management and Governance, Demand Generation and Communication, and monitoring and evaluation. For this study we used the service delivery data which included mothers or care givers sociodemographic and economic variables, child characteristics, mothers/care givers maternal health service utilization, perception on vaccination, and access to vaccination site were included. The variables included in the analysis are mentioned in measurement section. Referee the revised manuscript on page 7 line number 155-158. Since we requested the data and access the data from the database, we couldn’t have any reference. |
|  | Sampling and sample size - It's unclear if this was about the national immunization survey or other steps you took during the analysis or in the methods. | Thank you for the important points. The sample size for the national survey was computed based on the WHO cluster survey guideline (19) and considered the 2019 Ethiopian Demographic and Health Survey (EDHS) coverage reports for Pentavalent-3 (61.9%), zero dose (19.9%), and MCV2 (9.1%). The survey conducted with the assumption of a 95% of level of confidence, 2% margin of error, design effect of 3, 10% nonresponse rate, and the expected number of eligible children for each administrative region as 482. This gave the minimum sample size of 15,158 households with children aged 12-35 months.  A two-stage stratified cluster sampling technique was employed to select participants. Enumeration areas (EAs) which formed a cluster were randomly selected with urban-rural stratification. Sampling frames were prepared for each region and city administrations by the Ethiopian Statistical Services (ESS). The number of EAs required per region and city administration was determined based on the size within the stratum (study regions) and proportion of the Ethiopia population living in urban and rural areas (21.4% urban and 78.6% rural). A total of 468 EAs (100 EAs from urban and 368 EAs from rural areas) were randomly selected for the survey. For this analysis we used children aged 12-23 years considering the program importance of this age group. |
|  | Was this about the data source? Clarify this! Lines 181-184 are unclear. Please check it and rephrase for clarity. | This is about the data analysis to build a model to identify the predictor variables using different statistical and spatial models. |
|  | Ethical considerations - how did you handle ethical issues regarding the geographic locations of women? Explain the steps you took. | Thank you so much for the comments. The geographic location data were collected at household level. To handle the ethical issues and to make the data suitable for the spatial analysis we computed the centroid of the latitude and longitude of the geographic data. |
| **Results** | Data presentation on table 1 requires simplification. Suggestion - you can have one column for the number of participants and once column for "yes" responses, i.e. column %s. Presenting the "no" responses doesn't add any value. | Thank you for your suggestion. We have modified the table accordingly. However, one of the reviewers commented that we should include a column precents instead of row precents. If we add the total number of participants in the column and remove the zero-dose children, we would only show the proportion of zero-dose children from the total for each variable. Our intention is to highlight the contribution of each variable to the zero-dose category. Therefore, we have retained both columns and added a column total, rather than providing row totals. We welcome any further suggestions for improving the readability and simplicity of the table. Refer on page 11 |
|  | Move the methods-related results to the methods section where your presented the analysis and considerations. Present only the results related to the study question in the results section. | Thank you for your comments. We reviewed the results section in light of your feedback. While we have made revisions, we believe it is important to maintain the current structure. Moving the method-related results to the methodology section may disrupt the flow of the paper and compromise the logical coherence of the results. |
| **Discussion** | The discussion lacks focus and is too long. Please only discuss the key findings and their implications to assist the readers follow the discussion, with the focus on the key findings. | Thank you for the comments. We made a significant modification on the discussion section. Please refer on page 16-20, line number 320-407 |
| **Reviewer 1 comments** | | |
| Overall comments: | Overall comments: The methods are sound; however, the manuscript would greatly benefit from editing for clarity and grammatical accuracy. | Thanks for the positive words. We tried to address the specific comments accordingly |
| Major comments: | **Methods** The authors write that the OLS was used as a baseline model with classical assumptions of linear regression such as multicollinearity. Did the authors omit a word and in fact meant no multicollinearity? The assumptions of linear regression are linearity, homoscedasticity, independence (of error), normality and no multicollinearity. | Thank you for the comments. Indeed, the assumptions are the same with some additional assumptions. OLS equation is based on several key assumptions. First, it is assumed that the relationship between the independent and dependent variables is constant across the study area, indicating stationarity. Additionally, the residual values should have a mean of zero, meaning that any over- or under-estimations balance out. It is also important that the residuals are randomly distributed along the regression line, without any discernible pattern. Finally, the variables included in the model should not be highly correlated with one another to ensure the validity of the regression analysis. We followed the same approach. |
|  | How was vaccination status assessed? Was verbal report of receipt of DTP-vaccine by the caregiver accepted or was status based only on card confirmation? | Thank you for the comments. Yes. verbal report of receipt of DTP-vaccine by the caregiver was accepted. We used Immunization cards/health facility registration books/ mothers or caregivers’ recall for the most recent birth. If the mother reported vaccination but could not provide the card, health facility records were reviewed. As a last resort, caregivers' recall was used. To minimize recall bias, interviewers asked about the injection site, administration route (e.g., oral or injection), and the vaccination schedule. |
|  | **Results** ♣ The authors should present column percents and not row percents in Table 1 ♣ Please include total n in Table 1 (for yes zero-dose and no zero-dose). Additionally, please include a row for missing for the descriptive characteristics (e.g. n = 1,359 for perception of immunization benefit (poor/good) vs 1,537 for place of residency (urban/rural) among zero-dose children) | Thank you for your comments. We have made several adjustments in response. We modified the table to include column precent instead of row precent. Additionally, we highlighted the total sample for variables with missing values, such as the perception of immunization benefit (poor/good), since this variable was only asked of those who had heard about vaccination/immunization.  However, we could not include totals for both zero-dose and non-zero-dose participants, as this would complicate the table. Including these totals would merely show the proportion of zero-dose participants, whereas our aim is to highlight the proportion of zero-dose within each variable category.  We welcome any further suggestions for improving the readability and simplicity of the table. Refer on page 11 in the revised manuscript. |
| **Minor comments** | The authors write that they used data from a recent immunization program evaluation. Do the authors have information on when the immunization program evaluation was conducted? The data were accessed in 2024 but when was the survey conducted? If so, please include in the methods section | Yes. The survey was conducted from May to July 2023. It is now included in the revised manuscript. Refer on page 6, line number122-125 |
|  | Line 27 – 29: The sentence is not grammatically correct and is too long. The reviewer suggests that the authors separate the sentence into two sentences. Example: “Though Ethiopia has made significant progress in childhood vaccination, many children remain unvaccinated, making it the third largest contributor to the global burden of zero-dose children. Zero-dose children are those children who have not received the first dose of diphtheria, tetanus and pertussis containing vaccine.” | Thank you for your comment and suggestion. We have accepted it and made modifications based on your suggestion. Refer on page 2 line number 27-29 in the revised manuscript. |
|  | o Line 82 – There is a typo in this sentence : “highest number of for un-…” | Thank you for your comments, and I apologize for any inconvenience. It is not a typo error. The text was intended to refer to "unvaccinated" and "under-vaccinated." To improve clarity, we have written these two terms separately in the revised manuscript. Refer on page 4, line number 82 |
|  | Line 83 – This sentence is grammatically incorrect : “…children are estimated to haven’t received…” | Thank you for the comment. We tried to address the comments. page 4, line number 83 |
|  | Line 102 – there is a typo in this sentence (“evidences”) o Line 102 – there is a typo in this sentence. | Thank you for the comment. Comment addressed. Refer on page 5, line number 100-101 |
| **Minor comments** | o Line 212 – 212 : Error – reference source not found | Thank you for your comment. We were unable to identify the specific error or the missing reference. The statement, “A high proportion of zero-dose children were found among rural residents (27.2%) and mothers/caregivers with no education (30.5%). The majority (68.3%) of children whose mothers did not have an ANC visit did not receive the first dose of the pentavalent vaccine (Table 1),” is mentioned in line 212. If the comment pertains to the values, please note that these figures have been updated in the revised manuscript based on your previous suggestion |
|  | o Line 345 – There is a typo in this sentence | Thank you for the comment. Comment addressed. Refer on page 18, line number 363-364 |
|  | o Line 348 – there is a typo in this sentence “ the coefficients from your GWR model” | Thank you for the comment. Comment addressed. Refer on page 18, line number 363-364 |
|  |  |  |
| **Reviewer #2: comments** | |  |
|  | The manuscript has been highlighting a strong background and justification as Ethiopia is contributing to over 6% of zero-dose burden globally and inequities exit between various geographies. The methodologies and study design are explicitly defined. The results are well explained while the ethical approvals have also been indicated | Thank you to read and review our paper. |
