# Supplementary material for: Spatial disparities in zero-dose vaccination coverage for children aged 12–23 months in Ethiopia: A geographically weighted regression analysis
Source: PLoS One. 2025 Sep 11;20(9):e0332162. doi: 10.1371/journal.pone.0332162 (PMC12425285; doi:10.1371/journal.pone.0332162)
Supplement: Attachment — Submitted filename: Response_to_Reviewers_auresp_2.docx [file pone.0332162.s007.docx]

**Editorial Board**

**PLOS One**

The authors would like to thank the editorial team and reviewer for the constructive and valuable comments. In this revised manuscript we tried to address the concerns and comments in a point-by-point response which were not addressed in the first round of the review response. The changes addressed by the authors are marked with track changes throughout the revised manuscript. We tried to address issues raised and the authors are pleased to submit the revised manuscript titled **‘Spatial Disparities in Zero-Dose Vaccination Coverage for Children Aged 12-23 Months in Ethiopia: A Geographically Weighted Regression Analysis’** for publication. Authors are very grateful for your absolute contributions in improving the quality of the manuscript. We are very keen to incorporate any further comments, if any, for the betterment of the final manuscript.

| **S.No** | **Comments** | **Point by point Responses** |
| --- | --- | --- |
| **Academic Editor** | |  |
| **Methods** | Expand the data source section - who conducted the national immunization survey? When was it conducted? Who was/were the participants? How many? Did the survey use a representative sample? And include any pertinent information about the survey. Cite references for the data source | Apology for the inconvenience. Thank you so much again for the comments. We tried to expand the data source section. Kindly request to refer on page 6, line number 120-139 & on page 7, line number 146-149 of the revised tracked version of the manuscript. |
|  | Did the survey use a representative sample? And include any pertinent information about the survey, Sampling and sample size - It's unclear if this was about the national immunization survey or other steps you took during the analysis or in the methods. | Thanks for this. Yes. The survey used a representative. Referee the revised manuscript on page 6, line number 122-133. Regarding the sample size and sampling we removed as a section, but we tried to modify and included in the data source and type section. Refer on page 6, line number 123-133 and on page 7, line number 153-165 of the revised tracked version of the manuscript. |
|  | Include a citation for the survey report as a reference. Clarify who conducted the study, owned the data, and shared it with you. Cite references for the data source | Thank you for the comments. The survey, was conducted by five Capacity Building and Mentorship Partnership (CBMP) universities namely Addis Ababa, Jimma, Haramaya, Hawassa, and University of Gondar. In addition, The Immunization Inter-agency Coordinating Committee (ICC), the National Immunization Technical Advisory Group (NITAG), and the Immunization Technical Working Group (TWG) were involved in the study process. The owner of the data is Ministry of health and requested for this research purpose. Refer on page 6, line number 134-139 of the revised tracked version of the manuscript. |
|  | Ethical considerations - how did you handle ethical issues regarding the geographic locations of women? Explain the steps you took. | Thank you again for the comment and apology for the inconvenience. The geographic location data were collected at household level. To handle the ethical issues and to make the data suitable for the spatial analysis we computed the centroid of the latitude and longitude of the geographic data. Refer on page 11, line number 233-235 of the revised manuscript of the revised tracked version of the manuscript. |
| **Reviewer 1 comments** | | |
| Method | The authors write that the OLS was used as a baseline model with classical assumptions of linear regression such as multicollinearity. Did the authors omit a word and in fact meant no multicollinearity? The assumptions of linear regression are linearity, homoscedasticity, independence (of error), normality and no multicollinearity. | Thank you for the comments. Indeed, we tried to explain in the previous response. Know we included the word no multicollinearity rather than multicollinearity by changed the direction for the value of VIF. Refer on page 10, line number 212 of the revised tracked version of the manuscript. |
|  | How was vaccination status assessed? Was verbal report of receipt of DTP-vaccine by the caregiver accepted or was status based only on card confirmation? | Thank you for the comments. Yes. verbal report of receipt of DTP-vaccine by the caregiver was accepted. Refer on page 7, line number 146-149 of the revised tracked version of the manuscript. |
|  | o Line 212 – 212 : Error – reference source not found | Thank you for your comment and apology for the inconvenience. We addressed accordingly. Refer on page 11, line number 241 of the revised tracked version of the manuscript. |
