# Supplementary material for: Spatial disparities in zero-dose vaccination coverage for children aged 12–23 months in Ethiopia: A geographically weighted regression analysis
Source: PLoS One. 2025 Sep 11;20(9):e0332162. doi: 10.1371/journal.pone.0332162 (PMC12425285; doi:10.1371/journal.pone.0332162)
Supplement: Peer review history

# Peer review: Decision Letter 0

**DOI:** 10.1371/journal.pone.0332162.r001

**Authors:** Bereket Yakob

13 May 2025

Dear Dr. Endehabtu,

Thank you for submitting your manuscript to PLOS ONE. After careful consideration, we feel that it has merit but does not fully meet PLOS ONE’s publication criteria as it currently stands. Therefore, we invite you to submit a revised version of the manuscript that addresses the points raised during the review process.

==============================

**ACADEMIC EDITOR:**

==============================

Please submit your revised manuscript by Jun 27 2025 11:59PM. If you will need more time than this to complete your revisions, please reply to this message or contact the journal office at plosone@plos.org . When you're ready to submit your revision, log on to https://www.editorialmanager.com/pone/ and select the 'Submissions Needing Revision' folder to locate your manuscript file.

- A rebuttal letter that responds to each point raised by the academic editor and reviewer(s). You should upload this letter as a separate file labeled 'Response to Reviewers'.
- A marked-up copy of your manuscript that highlights changes made to the original version. You should upload this as a separate file labeled 'Revised Manuscript with Track Changes'.
- An unmarked version of your revised paper without tracked changes. You should upload this as a separate file labeled 'Manuscript'.

If you would like to make changes to your financial disclosure, please include your updated statement in your cover letter. Guidelines for resubmitting your figure files are available below the reviewer comments at the end of this letter.

If applicable, we recommend that you deposit your laboratory protocols in protocols.io to enhance the reproducibility of your results. Protocols.io assigns your protocol its own identifier (DOI) so that it can be cited independently in the future. For instructions see: https://journals.plos.org/plosone/s/submission-guidelines#loc-laboratory-protocols . Additionally, PLOS ONE offers an option for publishing peer-reviewed Lab Protocol articles, which describe protocols hosted on protocols.io. Read more information on sharing protocols at https://plos.org/protocols?utm_medium=editorial-email&utm_source=authorletters&utm_campaign=protocols .

We look forward to receiving your revised manuscript.

Kind regards,

Bereket Yakob, Ph.D.

Academic Editor

PLOS ONE

Journal Requirements:

When submitting your revision, we need you to address these additional requirements.

1. Please ensure that your manuscript meets PLOS ONE's style requirements, including those for file naming. The PLOS ONE style templates can be found at

https://journals.plos.org/plosone/s/file?id=wjVg/PLOSOne_formatting_sample_main_body.pdf and

https://journals.plos.org/plosone/s/file?id=ba62/PLOSOne_formatting_sample_title_authors_affiliations.pdf

2. We note that your Data Availability Statement is currently as follows: [All relevant data are within the manuscript and its Supporting Information file.]

Please confirm at this time whether or not your submission contains all raw data required to replicate the results of your study. Authors must share the “minimal data set” for their submission. PLOS defines the minimal data set to consist of the data required to replicate all study findings reported in the article, as well as related metadata and methods (https://journals.plos.org/plosone/s/data-availability#loc-minimal-data-set-definition).

For example, authors should submit the following data:

- The values behind the means, standard deviations and other measures reported;

- The values used to build graphs;

- The points extracted from images for analysis.

Authors do not need to submit their entire data set if only a portion of the data was used in the reported study.

If your submission does not contain these data, please either upload them as Supporting Information files or deposit them to a stable, public repository and provide us with the relevant URLs, DOIs, or accession numbers. For a list of recommended repositories, please see https://journals.plos.org/plosone/s/recommended-repositories.

If there are ethical or legal restrictions on sharing a de-identified data set, please explain them in detail (e.g., data contain potentially sensitive information, data are owned by a third-party organization, etc.) and who has imposed them (e.g., an ethics committee). Please also provide contact information for a data access committee, ethics committee, or other institutional body to which data requests may be sent. If data are owned by a third party, please indicate how others may request data access.

3. We note that the grant information you provided in the ‘Funding Information’ and ‘Financial Disclosure’ sections do not match.

When you resubmit, please ensure that you provide the correct grant numbers for the awards you received for your study in the ‘Funding Information’ section.

4. We note that Figures 3-7 in your submission contain [map/satellite] images which may be copyrighted. All PLOS content is published under the Creative Commons Attribution License (CC BY 4.0), which means that the manuscript, images, and Supporting Information files will be freely available online, and any third party is permitted to access, download, copy, distribute, and use these materials in any way, even commercially, with proper attribution. For these reasons, we cannot publish previously copyrighted maps or satellite images created using proprietary data, such as Google software (Google Maps, Street View, and Earth). For more information, see our copyright guidelines: http://journals.plos.org/plosone/s/licenses-and-copyright.

We require you to either (1) present written permission from the copyright holder to publish these figures specifically under the CC BY 4.0 license, or (2) remove the figures from your submission:

 a. You may seek permission from the original copyright holder of Figures 3-7 to publish the content specifically under the CC BY 4.0 license. 

We recommend that you contact the original copyright holder with the Content Permission Form (http://journals.plos.org/plosone/s/file?id=7c09/content-permission-form.pdf) and the following text:

“I request permission for the open-access journal PLOS ONE to publish XXX under the Creative Commons Attribution License (CCAL) CC BY 4.0 (http://creativecommons.org/licenses/by/4.0/). Please be aware that this license allows unrestricted use and distribution, even commercially, by third parties. Please reply and provide explicit written permission to publish XXX under a CC BY license and complete the attached form.”

Please upload the completed Content Permission Form or other proof of granted permissions as an "Other" file with your submission.

In the figure caption of the copyrighted figure, please include the following text: “Reprinted from [ref] under a CC BY license, with permission from [name of publisher], original copyright [original copyright year].”

b. If you are unable to obtain permission from the original copyright holder to publish these figures under the CC BY 4.0 license or if the copyright holder’s requirements are incompatible with the CC BY 4.0 license, please either i) remove the figure or ii) supply a replacement figure that complies with the CC BY 4.0 license. Please check copyright information on all replacement figures and update the figure caption with source information. If applicable, please specify in the figure caption text when a figure is similar but not identical to the original image and is therefore for illustrative purposes only.

The following resources for replacing copyrighted map figures may be helpful:

USGS National Map Viewer (public domain): http://viewer.nationalmap.gov/viewer/

The Gateway to Astronaut Photography of Earth (public domain): http://eol.jsc.nasa.gov/sseop/clickmap/

Maps at the CIA (public domain): https://www.cia.gov/library/publications/the-world-factbook/index.html and https://www.cia.gov/library/publications/cia-maps-publications/index.html

NASA Earth Observatory (public domain): http://earthobservatory.nasa.gov/

Landsat: http://landsat.visibleearth.nasa.gov/

USGS EROS (Earth Resources Observatory and Science (EROS) Center) (public domain): http://eros.usgs.gov/#

Natural Earth (public domain): http://www.naturalearthdata.com/

Additional Editor Comments (if provided):

Dear Endehabtu,

Thanks for submitting your manuscript to PLOS One! We have reviewed your manuscript and found that it has a merit. However, before we make the final decision on it, I kindly ask you to make amendments to the review comments or write a rebuttal letter to each of the points raised by the reviewers.

Below are my comments for your consideration.

General comment:

Please check every line and sentence for language clarity and typos and amend accordingly.

Specific comments:

Move line 115 to 121 (put it under the data sources).

Methods section:

Expand the data source section - who conducted the national immunization survey? When was it conducted? Who was/were the participants? How many? Did the survey use a representative sample? And include any pertinent information about the survey. Cite references for the data source.

Sampling and sample size - It's unclear if this was about the national immunization survey or other steps you took during the analysis or in the methods. Was this about the data source? Clarify this!

Lines 181-184 are unclear. Please check it and rephrase for clarity.

Ethical considerations - how did you handle ethical issues regarding the geographic locations of women? Explain the steps you took.

Results:

Data presentation on table 1 requires simplification. Suggestion - you can have one column for the number of participants and once column for "yes" responses, i.e. column %s. Presenting the "no" responses doesn't add any value.

Move the methods-related results to the methods section where your presented the analysis and considerations. Present only the results related to the study question in the results section.

Discussion

The discussion lacks focus and is too long. Please only discuss the key findings and their implications to assist the readers follow the discussion, with the focus on the key findings.

[Note: HTML markup is below. Please do not edit.]

Reviewers' comments:

Reviewer's Responses to Questions

**Comments to the Author**

1. Is the manuscript technically sound, and do the data support the conclusions?

Reviewer #1: Yes

Reviewer #2: Yes

**********

2. Has the statistical analysis been performed appropriately and rigorously?

Reviewer #1: Yes

Reviewer #2: Yes

**********

3. Have the authors made all data underlying the findings in their manuscript fully available?

The PLOS Data policy

Reviewer #1: Yes

Reviewer #2: Yes

**********

4. Is the manuscript presented in an intelligible fashion and written in standard English?

Reviewer #1: No

Reviewer #2: Yes

**********

Reviewer #1: Spatial Disparities in Zero-Dose Vaccination Coverage for Children Aged 12-23 Months in Ethiopia: A Geographically Weighted Regression Analysis

Synopsis: Using data from Ethiopia’s most recent national immunization survey, the authors examined the prevalence and geographic distribution of zero-dose children. Zero dose children are defined as children not having received the first dose of the diphtheria, tetanus, pertussis vaccine. Results from this study indicate that the prevalence of zero-dose children is 25% and spatial analyses identified statistically significant clusters of zero-dose children. Other results suggested that no antenatal care utilization, poor perception of benefits of immunization, no maternal tt/td vaccination and perception of the distance of the nearest facility as a big problem were significant predictors of zero-dose status. Overall, the authors highlight that contextual and social factors are crucial to consider for immunization programs, and that tailored approaches are needed.

Overall comments: The methods are sound; however, the manuscript would greatly benefit from editing for clarity and grammatical accuracy.

Major comments:

o Methods

� The authors write that the OLS was used as a baseline model with classical assumptions of linear regression such as multicollinearity. Did the authors omit a word and in fact meant no multicollinearity? The assumptions of linear regression are linearity, homoscedasticity, independence (of error), normality and no multicollinearity.

� How was vaccination status assessed? Was verbal report of receipt of DTP-vaccine by the caregiver accepted or was status based only on card confirmation?

o Results

� The authors should present column percents and not row percents in Table 1

� Please include total n in Table 1 (for yes zero-dose and no zero-dose). Additionally, please include a row for missing for the descriptive characteristics (e.g. n = 1,359 for perception of immunization benefit (poor/good) vs 1,537 for place of residency (urban/rural) among zero-dose children)

Minor comments:

o The authors write that they used data from a recent immunization program evaluation. Do the authors have information on when the immunization program evaluation was conducted? The data were accessed in 2024 but when was the survey conducted? If so, please include in the methods section.

o Line 27 – 29: The sentence is not grammatically correct and is too long. The reviewer suggests that the authors separate the sentence into two sentences. Example: “Though Ethiopia has made significant progress in childhood vaccination, many children remain unvaccinated, making it the third largest contributor to the global burden of zero-dose children. Zero-dose children are those children who have not received the first dose of diphtheria, tetanus and pertussis containing vaccine.”

o Line 82 – There is a typo in this sentence : “highest number of for un-…”

o Line 83 – This sentence is grammatically incorrect : “…children are estimated to haven’t received…”

o Line 102 – there is a typo in this sentence (“evidences”)

o Line 102 – there is a typo in this sentence.

o Line 212 – 212 : Error – reference source not found

o Line 345 – There is a typo in this sentence

o Line 348 – there is a typo in this sentence “ the coefficients from your GWR model”

Reviewer #2: The manuscript has been highlighting a strong background and justification as Ethiopia is contributing to over 6% of zero-dose burden globally and inequities exit between various geographies. The methodologies and study design are explicitly defined. The results are well explained while the ethical approvals have also been indicated.

**********

what does this mean? ). If published, this will include your full peer review and any attached files.

If you choose “no”, your identity will remain anonymous but your review may still be made public.

**Do you want your identity to be public for this peer review?** For information about this choice, including consent withdrawal, please see our Privacy Policy

Reviewer #1: No

Reviewer #2: **Yes: ** SOOFIA YUNUS

**********

[NOTE: If reviewer comments were submitted as an attachment file, they will be attached to this email and accessible via the submission site. Please log into your account, locate the manuscript record, and check for the action link "View Attachments". If this link does not appear, there are no attachment files.]

While revising your submission, please upload your figure files to the Preflight Analysis and Conversion Engine (PACE) digital diagnostic tool, https://pacev2.apexcovantage.com/ . PACE helps ensure that figures meet PLOS requirements. To use PACE, you must first register as a user. Registration is free. Then, login and navigate to the UPLOAD tab, where you will find detailed instructions on how to use the tool. If you encounter any issues or have any questions when using PACE, please email PLOS at figures@plos.org . Please note that Supporting Information files do not need this step.

Attachment Submitted filename: PLOS One Review_8April2025.docx

Attachment Submitted filename: PONE-D-24-59668 (1).pdf

# Author comment: Author response to Decision Letter 1

**DOI:** 10.1371/journal.pone.0332162.r002

21 Jun 2025

Editor’s comments

1. We note that your Data Availability Statement is currently as follows: [All relevant data are within the manuscript and its Supporting Information file.] Please confirm at this time whether or not your submission contains all raw data required to replicate the results of your study. Authors must share the “minimal data set” for their submission

Response: Thank you so much for the comment. We addressed the comment. The dataset is included in the submission and also added as a caption in the revised manuscript. Refer on page 25, line number 610-612 of the trach version

2. We note that the grant information you provided in the ‘Funding Information’ and ‘Financial Disclosure’ sections do not match.

Response: Sorry for the inconvenience. Issue addressed.

3. You may seek permission from the original copyright holder of Figures 3-7 to publish the content specifically under the CC BY 4.0 license.

Response: Thanks for your comment. The shapefiles are sourced from https://data.humdata.org/dataset/cod-ab-eth. which is an open platform, in which the data is publicly available for anyone to use.

4. Please check every line and sentence for language clarity and typos and amend accordingly.

Response: Thanks for your comment. We tried to address the grammatical and typos errors throughout the manuscript.

5. Move line 115 to 121 (put it under the data sources).

Response: Thank you for the comment. We moved line 115 to the recommended place. Refer on page 6, line number 122-123

6. Expand the data source section - who conducted the national immunization survey? When was it conducted? Who was/were the participants? How many? Did the survey use a representative sample? And include any pertinent information about the survey. Cite references for the data source

Response: Thank you so much for the comments. We tried to expand the data source section. Refer on page 6 line number 122-136. The number of children included in the analysis is mentioned in the main document on page 7 line number 148.

7. Did the survey use a representative sample? And include any pertinent information about the survey. Cite references for the data source.

Response: Thanks for this. Yes. The survey used a representative sample conducted across all regions of Ethiopia except Tigray region. Regarding the pertinent information, the survey included service delivery, Supply Chain Management, Adverse Events Following Immunization (AEFI), Vaccine Preventable Diseases Surveillance, Leadership, Management and Governance, Demand Generation and Communication, and monitoring and evaluation. For this study we used the service delivery data which included mothers or care givers sociodemographic and economic variables, child characteristics, mothers/care givers maternal health service utilization, perception on vaccination, and access to vaccination site were included. The variables included in the analysis are mentioned in measurement section. Referee the revised manuscript on page 7 line number 155-158. Since we requested the data and access the data from the database, we couldn’t have any reference.

8. Sampling and sample size - It's unclear if this was about the national immunization survey or other steps you took during the analysis or in the methods.

Response: Thank you for the important points. The sample size for the national survey was computed based on the WHO cluster survey guideline (19) and considered the 2019 Ethiopian Demographic and Health Survey (EDHS) coverage reports for Pentavalent-3 (61.9%), zero dose (19.9%), and MCV2 (9.1%). The survey conducted with the assumption of a 95% of level of confidence, 2% margin of error, design effect of 3, 10% nonresponse rate, and the expected number of eligible children for each administrative region as 482. This gave the minimum sample size of 15,158 households with children aged 12-35 months. A two-stage stratified cluster sampling technique was employed to select participants. Enumeration areas (EAs) which formed a cluster were randomly selected with urban-rural stratification. Sampling frames were prepared for each region and city administrations by the Ethiopian Statistical Services (ESS). The number of EAs required per region and city administration was determined based on the size within the stratum (study regions) and proportion of the Ethiopia population living in urban and rural areas (21.4% urban and 78.6% rural). A total of 468 EAs (100 EAs from urban and 368 EAs from rural areas) were randomly selected for the survey. For this analysis we used children aged 12-23 years considering the program importance of this age group.

9. Was this about the data source? Clarify this! Lines 181-184 are unclear. Please check it and rephrase for clarity.

Response: This is about the data analysis to build a model to identify the predictor variables using different statistical and spatial models.

10. Ethical considerations - how did you handle ethical issues regarding the geographic locations of women? Explain the steps you took.

Response: Thank you so much for the comments. The geographic location data were collected at household level. To handle the ethical issues and to make the data suitable for the spatial analysis we computed the centroid of the latitude and longitude of the geographic data.

11. Data presentation on table 1 requires simplification. Suggestion - you can have one column for the number of participants and once column for "yes" responses, i.e. column %s. Presenting the "no" responses doesn't add any value.

Response: have modified the table accordingly. However, one of the reviewers commented that we should include a column precents instead of row precents. If we add the total number of participants in the column and remove the zero-dose children, we would only show the proportion of zero-dose children from the total for each variable. Our intention is to highlight the contribution of each variable to the zero-dose category. Therefore, we have retained both columns and added a column total, rather than providing row totals. We welcome any further suggestions for improving the readability and simplicity of the table. Refer on page 11

12. Move the methods-related results to the methods section where your presented the analysis and considerations. Present only the results related to the study question in the results section.

Response: Thank you for your comments. We reviewed the results section in light of your feedback. While we have made revisions, we believe it is important to maintain the current structure. Moving the method-related results to the methodology section may disrupt the flow of the paper and compromise the logical coherence of the results.

13. The discussion lacks focus and is too long. Please only discuss the key findings and their implications to assist the readers follow the discussion, with the focus on the key findings.

Response: Thank you for the comments. We made a significant modification on the discussion section. Please refer on page 16-20, line number 320-407

Reviewer 1 comments:

1. Overall comments: The methods are sound; however, the manuscript would greatly benefit from editing for clarity and grammatical accuracy.

Response: Thanks for the positive words. We tried to address the specific comments accordingly

2. The authors write that the OLS was used as a baseline model with classical assumptions of linear regression such as multicollinearity. Did the authors omit a word and in fact meant no multicollinearity? The assumptions of linear regression are linearity, homoscedasticity, independence (of error), normality and no multicollinearity.

Response: Thank you for the comments. Indeed, the assumptions are the same with some additional assumptions. OLS equation is based on several key assumptions. First, it is assumed that the relationship between the independent and dependent variables is constant across the study area, indicating stationarity. Additionally, the residual values should have a mean of zero, meaning that any over- or under-estimations balance out. It is also important that the residuals are randomly distributed along the regression line, without any discernible pattern. Finally, the variables included in the model should not be highly correlated with one another to ensure the validity of the regression analysis. We followed the same approach.

3. How was vaccination status assessed? Was verbal report of receipt of DTP-vaccine by the caregiver accepted or was status based only on card confirmation?

Response: Thank you for the comments. Yes. verbal report of receipt of DTP-vaccine by the caregiver was accepted. We used Immunization cards/health facility registration books/ mothers or caregivers’ recall for the most recent birth. If the mother reported vaccination but could not provide the card, health facility records were reviewed. As a last resort, caregivers' recall was used. To minimize recall bias, interviewers asked about the injection site, administration route (e.g., oral or injection), and the vaccination schedule.

4. The authors should present column percents and not row percents in Table 1

Please include total n in Table 1 (for yes zero-dose and no zero-dose). Additionally, please include a row for missing for the descriptive characteristics (e.g. n = 1,359 for perception of immunization benefit (poor/good) vs 1,537 for place of residency (urban/rural) among zero-dose children)

Response: Thank you for your comments. We have made several adjustments in response. We modified the table to include column precent instead of row precent. Additionally, we highlighted the total sample for variables with missing values, such as the perception of immunization benefit (poor/good), since this variable was only asked of those who had heard about vaccination/immunization.

However, we could not include totals for both zero-dose and non-zero-dose participants, as this would complicate the table. Including these totals would merely show the proportion of zero-dose participants, whereas our aim is to highlight the proportion of zero-dose within each variable category.

We welcome any further suggestions for improving the readability and simplicity of the table. Refer on page 11 in the revised manuscript.

5. The authors write that they used data from a recent immunization program evaluation. Do the authors have information on when the immunization program evaluation was conducted? The data were accessed in 2024 but when was the survey conducted? If so, please include in the methods section

Response: Yes. The survey was conducted from May to July 2023. It is now included in the revised manuscript. Refer on page 6, line number122-125

6. Line 27 – 29: The sentence is not grammatically correct and is too long. The reviewer suggests that the authors separate the sentence into two sentences. Example: “Though Ethiopia has made significant progress in childhood vaccination, many children remain unvaccinated, making it the third largest contributor to the global burden of zero-dose children. Zero-dose children are those children who have not received the first dose of diphtheria, tetanus and pertussis containing vaccine.

Response: Thank you for your comment and suggestion. We have accepted it and made modifications based on your suggestion. Refer on page 2 line number 27-29 in the revised manuscript.

7. Line 82 – There is a typo in this sentence : “highest number of for un-…

Response: Thank you for your comments, and I apologize for any inconvenience. It is not a typo error. The text was intended to refer to "unvaccinated" and "under-vaccinated." To improve clarity, we have written these two terms separately in the revised manuscript. Refer on page 4, line number 82

8. Line 83 – This sentence is grammatically incorrect : “…children are estimated to haven’t received…”

Response: Thank you for the comment. We tried to address the comments. page 4, line number 83

9. Line 102 – there is a typo in this sentence (“evidences”)Line 102 – there is a typo in this sentence.

Response: Thank you for the comment. Comment addressed. Refer on page 5, line number 100-101

10. Line 212 – 212 : Error – reference source not found

Response: Thank you for your comment. We were unable to identify the specific error or the missing reference. The statement, “A high proportion of zero-dose children were found among rural residents (27.2%) and mothers/caregivers with no education (30.5%). The majority (68.3%) of children whose mothers did not have an ANC visit did not receive the first dose of the pentavalent vaccine (Table 1),” is mentioned in line 212. If the comment pertains to the values, please note that these figures have been updated in the revised manuscript based on your previous suggestion

11. Line 345 – There is a typo in this sentence

Response: Thank you for the comment. Comment addressed. Refer on page 18, line number 363-364

12. Line 348 – there is a typo in this sentence “ the coefficients from your GWR model”

Response: Thank you for the comment. Comment addressed. Refer on page 18, line number 363-364

Reviewer 2 comments:

1. The manuscript has been highlighting a strong background and justification as Ethiopia is contributing to over 6% of zero-dose burden globally and inequities exit between various geographies. The methodologies and study design are explicitly defined. The results are well explained while the ethical approvals have also been indicated

Response: Thank you to read and review

Attachment Submitted filename: Response to Reviewers.docx

# Editor report: Decision Letter 1

**DOI:** 10.1371/journal.pone.0332162.r003

**Authors:** Bereket Yakob

14 Aug 2025

Thank you for submitting your manuscript to PLOS ONE. After careful consideration, we feel that it has merit but does not fully meet PLOS ONE’s publication criteria as it currently stands. Therefore, we invite you to submit a revised version of the manuscript that addresses the points raised during the review process.

==============================

**ACADEMIC EDITOR:**

Please submit your revised manuscript by Sep 28 2025 11:59PM. If you will need more time than this to complete your revisions, please reply to this message or contact the journal office at plosone@plos.org . When you're ready to submit your revision, log on to https://www.editorialmanager.com/pone/ and select the 'Submissions Needing Revision' folder to locate your manuscript file.

- A rebuttal letter that responds to each point raised by the academic editor and reviewer(s). You should upload this letter as a separate file labeled 'Response to Reviewers'.
- A marked-up copy of your manuscript that highlights changes made to the original version. You should upload this as a separate file labeled 'Revised Manuscript with Track Changes'.
- An unmarked version of your revised paper without tracked changes. You should upload this as a separate file labeled 'Manuscript'.

If applicable, we recommend that you deposit your laboratory protocols in protocols.io to enhance the reproducibility of your results. Protocols.io assigns your protocol its own identifier (DOI) so that it can be cited independently in the future. For instructions see: https://journals.plos.org/plosone/s/submission-guidelines#loc-laboratory-protocols . Additionally, PLOS ONE offers an option for publishing peer-reviewed Lab Protocol articles, which describe protocols hosted on protocols.io. Read more information on sharing protocols at https://plos.org/protocols?utm_medium=editorial-email&utm_source=authorletters&utm_campaign=protocols .

We look forward to receiving your revised manuscript.

Kind regards,

Bereket Yakob, Ph.D.

Academic Editor

PLOS ONE

Journal Requirements:

If the reviewer comments include a recommendation to cite specific previously published works, please review and evaluate these publications to determine whether they are relevant and should be cited. There is no requirement to cite these works unless the editor has indicated otherwise. 

Please review your reference list to ensure that it is complete and correct. If you have cited papers that have been retracted, please include the rationale for doing so in the manuscript text, or remove these references and replace them with relevant current references. Any changes to the reference list should be mentioned in the rebuttal letter that accompanies your revised manuscript. If you need to cite a retracted article, indicate the article’s retracted status in the References list and also include a citation and full reference for the retraction notice.

Additional Editor Comments:

o Please update the section on sample size and sampling in response to my previous queries #6 and 7. Currently, it’s unclear whether this was part of the original study or if your team performed it during sampling. It appears you handled the sampling yourselves. If you want to keep the “sampling and sample size” section, I recommend describing how the national immunization survey conducted this process, rather than explaining how your team did it.

o Also, include a citation for the survey report as a reference. Clarify who conducted the study, owned the data, and shared it with you. The survey report is now available, and you can cite it in the 'Data source and type description' subsection. I have attached the survey report for your reference. Check if this was the survey you were referring to. 

o For my query #10, please indicate how you addressed it or provide justification for any modifications you chose not to make.

o In response to Reviewer 1’s comments #2 and 3, you did not specify the actions you took or modifications made. Please clearly state what changes, insertions, or omissions you implemented.

o Regarding Reviewer 1’s comment #10, the concern was valid, and the “Error – reference not found” message still appears in lines 211-212. It looks like your citation manager has not been updated recently, which causes this error in the PDF version of your manuscript. Updating your citations should resolve the issue.

[Note: HTML markup is below. Please do not edit.]

[NOTE: If reviewer comments were submitted as an attachment file, they will be attached to this email and accessible via the submission site. Please log into your account, locate the manuscript record, and check for the action link "View Attachments". If this link does not appear, there are no attachment files.]

While revising your submission, please upload your figure files to the Preflight Analysis and Conversion Engine (PACE) digital diagnostic tool, https://pacev2.apexcovantage.com/ . PACE helps ensure that figures meet PLOS requirements. To use PACE, you must first register as a user. Registration is free. Then, login and navigate to the UPLOAD tab, where you will find detailed instructions on how to use the tool. If you encounter any issues or have any questions when using PACE, please email PLOS at figures@plos.org . Please note that Supporting Information files do not need this step.

Attachment Submitted filename: National_immunization_program_evaluation 2023-ETHIOPIA.pdf

# Author comment: Author response to Decision Letter 2

**DOI:** 10.1371/journal.pone.0332162.r004

21 Aug 2025

Editor’s comments

1. Expand the data source section - who conducted the national immunization survey? When was it conducted? Who was/were the participants? How many? Did the survey use a representative sample? And include any pertinent information about the survey. Cite references for the data source

Response: Apology for the inconvenience. Thank you so much again for the comments. We tried to expand the data source section. Kindly request to refer on page 6, line number 120-139 & on page 7, line number 146-149 of the revised tracked version of the manuscript.

2. Did the survey use a representative sample? And include any pertinent information about the survey, Sampling and sample size - It's unclear if this was about the national immunization survey or other steps you took during the analysis or in the methods.

Response: Thanks for this. Yes. The survey used a representative. Referee the revised manuscript on page 6, line number 122-133. Regarding the sample size and sampling we removed as a section, but we tried to modify and included in the data source and type section. Refer on page 6, line number 123-133 and on page 7, line number 153-165 of the revised tracked version of the manuscript.

3. include a citation for the survey report as a reference. Clarify who conducted the study, owned the data, and shared it with you. Cite references for the data source

Response: Thank you for the comments. The survey, was conducted by five Capacity Building and Mentorship Partnership (CBMP) universities namely Addis Ababa, Jimma, Haramaya, Hawassa, and University of Gondar. In addition, The Immunization Inter-agency Coordinating Committee (ICC), the National Immunization Technical Advisory Group (NITAG), and the Immunization Technical Working Group (TWG) were involved in the study process. The owner of the data is Ministry of health and requested for this research purpose. Refer on page 6, line number 134-139 of the revised tracked version of the manuscript..

4. Ethical considerations - how did you handle ethical issues regarding the geographic locations of women? Explain the steps you took.

Response: Thank you again for the comment and apology for the inconvenience. The geographic location data were collected at household level. To handle the ethical issues and to make the data suitable for the spatial analysis we computed the centroid of the latitude and longitude of the geographic data. Refer on page 11, line number 233-235 of the revised tracked version of the manuscript.

Reviewer 1 comments:

1. The authors write that the OLS was used as a baseline model with classical assumptions of linear regression such as multicollinearity. Did the authors omit a word and in fact meant no multicollinearity? The assumptions of linear regression are linearity, homoscedasticity, independence (of error), normality and no multicollinearity.

Response: Thank you for the comments. Indeed, we tried to explain in the previous response. Know we included the word no multicollinearity rather than multicollinearity by changed the direction for the value of VIF. Refer on page 10, line number 212 of the revised tracked version of the manuscript.

2. How was vaccination status assessed? Was verbal report of receipt of DTP-vaccine by the caregiver accepted or was status based only on card confirmation?

Response: Thank you for the comments. Yes. verbal report of receipt of DTP-vaccine by the caregiver was accepted. Refer on page 7, line number 146-149 of the revised tracked version of the manuscript.

3. Line 212 – 212 : Error – reference source not found

Response: Thank you for your comment and apology for the inconvenience. We addressed accordingly. Refer on page 11, line number 241 of the revised tracked version of the manuscript.

Attachment Submitted filename: Response_to_Reviewers_auresp_2.docx

# Editor report: Decision Letter 2

**DOI:** 10.1371/journal.pone.0332162.r005

**Authors:** Bereket Yakob

27 Aug 2025

Spatial Disparities in Zero-Dose Vaccination Coverage for Children Aged 12-23 Months in Ethiopia: A Geographically Weighted Regression Analysis

PONE-D-24-59668R2

Dear Dr. Endehabtu,

We’re pleased to inform you that your manuscript has been judged scientifically suitable for publication and will be formally accepted for publication once it meets all outstanding technical requirements.

Within one week, you’ll receive an e-mail detailing the required amendments. When these have been addressed, you’ll receive a formal acceptance letter and your manuscript will be scheduled for publication.

An invoice will be generated when your article is formally accepted. Please note, if your institution has a publishing partnership with PLOS and your article meets the relevant criteria, all or part of your publication costs will be covered. Please make sure your user information is up-to-date by logging into Editorial Manager at Editorial Manager®  and clicking the ‘Update My Information' link at the top of the page. For questions related to billing, please contact billing support .

If your institution or institutions have a press office, please notify them about your upcoming paper to help maximize its impact. If they’ll be preparing press materials, please inform our press team as soon as possible -- no later than 48 hours after receiving the formal acceptance. Your manuscript will remain under strict press embargo until 2 pm Eastern Time on the date of publication. For more information, please contact onepress@plos.org.

Kind regards,

Bereket Yakob, Ph.D.

Academic Editor

PLOS ONE

Additional Editor Comments (optional):

Reviewers' comments:

# Editor report: Acceptance letter

**DOI:** 10.1371/journal.pone.0332162.r006

**Authors:** Bereket Yakob

PONE-D-24-59668R2

PLOS ONE

Dear Dr. Endehabtu,

I'm pleased to inform you that your manuscript has been deemed suitable for publication in PLOS ONE. Congratulations! Your manuscript is now being handed over to our production team.

At this stage, our production department will prepare your paper for publication. This includes ensuring the following:

* All references, tables, and figures are properly cited

* All relevant supporting information is included in the manuscript submission,

* There are no issues that prevent the paper from being properly typeset

You will receive further instructions from the production team, including instructions on how to review your proof when it is ready. Please keep in mind that we are working through a large volume of accepted articles, so please give us a few days to review your paper and let you know the next and final steps.

Lastly, if your institution or institutions have a press office, please let them know about your upcoming paper now to help maximize its impact. If they'll be preparing press materials, please inform our press team within the next 48 hours. Your manuscript will remain under strict press embargo until 2 pm Eastern Time on the date of publication. For more information, please contact onepress@plos.org.

You will receive an invoice from PLOS for your publication fee after your manuscript has reached the completed accept phase. If you receive an email requesting payment before acceptance or for any other service, this may be a phishing scheme. Learn how to identify phishing emails and protect your accounts at https://explore.plos.org/phishing.

If we can help with anything else, please email us at customercare@plos.org.

Thank you for submitting your work to PLOS ONE and supporting open access.

Kind regards,

PLOS ONE Editorial Office Staff

on behalf of

Dr. Bereket Yakob

Academic Editor

PLOS ONE
